# Beyond the Obesity Paradox: Analysis of New Prognostic Factors in Transcatheter Aortic Valve Implantation Procedure

**DOI:** 10.3390/jcdd11110368

**Published:** 2024-11-15

**Authors:** Francesca Ricci, Leonardo Benelli, Monia Pasqualetto, Mario Laudazi, Luca Pugliese, Maria Volpe, Cecilia Cerimele, Carlo Di Donna, Francesco Garaci, Marcello Chiocchi

**Affiliations:** Department of Biomedicine and Prevention Division of Diagnostic Imaging, University of Rome “Tor Vergata”, Viale Oxford 81, 00133 Rome, Italy; fraricci891@gmail.com (F.R.); monia.pasqua@gmail.com (M.P.); mariolauda@gmail.com (M.L.); l.pugliese88@gmail.com (L.P.); mariavolpe93mv@gmail.com (M.V.); cec.cerimele@gmail.com (C.C.); didonnacarlo@gmail.com (C.D.D.); garaci@gmail.com (F.G.); marcello.chiocchi@gmail.com (M.C.)

**Keywords:** obesity paradox, sarcopenia, fat density, BMI, CT, TAVI

## Abstract

Scope: The main purpose of our study was to collect computed tomography (CT) measurements of fat parameters that are significantly related to body mass index (BMI) and evaluate the associations of these measurements and sarcopenia with early and long-term complications after transcatheter aortic valve implantation (TAVI) in order to investigate the existence of the so-called ‘obesity paradox’ and the role of sarcopenia in this phenomenon. Materials and Methods: We analyzed the significance of fat CT measurements in 85 patients undergoing the TAVI procedure and compared these with each other, as well as with quantified CT BMI and fat density measurements. Secondly, we evaluated the associations of BMI, CT measurements of fat, and CT evaluations of skeletal muscle mass with early and long-term complications after 24 months of post-TAVI follow-up. Results: We found positive and significant relationships between fat CT measurements with each other and with BMI and a negative and significant relation between fat density and fat quantity. By comparing the CT measurements of fat and skeletal muscle mass with early and long-term complications after TAVI, we confirmed the existence of the ‘obesity paradox’ and the poor effect of sarcopenia after the TAVI procedure. Conclusions: We confirm that overweight and obesity are good prognostic factors, and sarcopenia is a poor prognostic factor for outcomes following the TAVI procedure. We focused on the scientific validation of an easy and fast way to measure fat and skeletal muscle mass using CT to better predict the outcomes of patients undergoing TAVI.

## 1. Introduction

Computed tomography (CT) is considered the gold standard for the pre-procedural study of transcatheter aortic valve implantation (TAVI) [1], the replacement of other valves [2], and it is also a valuable tool for the evaluation of post-procedural complications, with variable applicability depending on the type [3]. In particular, pre-TAVI CT provides a three-dimensional analysis of the heart, aortic valve, ascending aorta, coronary vessels, and peripheral axes and allows the evaluation of recent measurements identified as new prognostic factors for the outcomes of patients after undergoing the TAVI procedure [4,5,6]. The so-called ‘obesity paradox’ [7] and sarcopenia [8] have been recently listed among the predictive factors of post-TAVI outcomes by different authors. Regarding obesity, it is known to be a major risk factor for the development of cardiovascular disease [9]; however, several studies have revealed that having a higher body mass index (BMI) is associated with an unexpected survival advantage in patients who undergo TAVI procedures [10,11,12,13,14] and different types of surgery [15,16,17]. In contrast, regarding sarcopenia, other studies have demonstrated that patients with a low muscle volume have the poorest prognosis after surgery [18]. On the basis of previous studies that demonstrated the ability to perform an accurate quantitative analysis of adipose tissue (thicknesses and areas) using CT [19], the first aim of our research was to identify CT measurements that are significantly related to the BMI, allowing the visceral adipose tissue (VAT) and the subcutaneous adipose tissue (SAT) and their density to be quantified and differentiated. Secondly, we aimed to find a relationship between the BMI and the quantitative CT evaluation of adipose tissue and sarcopenia parameters, such as fat thicknesses and fat areas, and with the qualitative CT evaluation of fat represented by its density. The final aim of this work was to investigate the effects of the BMI, CT measurements of fat, and CT evaluations of sarcopenia on the clinical outcomes of the TAVI procedure, including their associations with early and long-term complications. In this scenario, we consider it essential to identify easy, reproducible, and fast CT measurements that allow the physician to conduct an accurate evaluation of prognostic factors associated with the TAVI procedure using CT without interfering and slowing down the daily workflow of radiologists.

## 2. Materials and Methods

This retrospective study included 85 patients who underwent transfemoral TAVI procedure at the Unit of Cardiology and Interventional Cardiology of ‘Tor Vergata University Hospital’ (Rome, Italy) between June 2019 and September 2021 and were subsequently monitored for a follow-up period of 2 years.

The research has been performed in accordance with the ethical standards laid down in the 1964 Declaration of Helsinki and its later amendments. All patients gave their informed consent for this study and their inclusion in the study. Details that might disclose the identity of the subjects under study were omitted.

**Figure 1:**
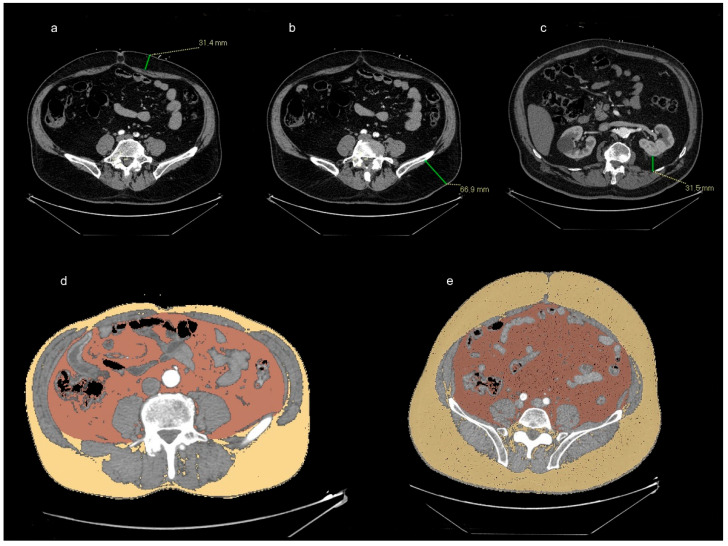
(**a**) Anterior SAT thickness, measured as the largest anterior distance on the axial slice between the rectus abdominis and the skin at the level of the umbilicus. (**b**) Posterior SAT thickness is measured as the distance between the left iliac crest and the posterior skin. (**c**) VAT thickness is obtained by calculating the retro-renal fat thickness as the distance from the left kidney to the nearest posterior inner abdominal wall on the axial slice passing through the left renal vein. (**d**,**e**) The VAT area, presented in orange, and the SAT area, presented in yellow, were segmented with 3D Slicer version 5.1.0 software using fat-specific thresholds.

Each pre-TAVI CT was performed using GE Healthcare Revolution Evo Gen 2 CT (Wauwatosa, WI, USA), and CT images were analyzed by a radiologist with 10 years of cardiovascular imaging experience. First, in order to study the effect of obesity on the patients’ outcomes after the TAVI procedure, we analyzed the fat distribution in terms of the VAT and SAT in the pre-TAVI CTs. Regarding the former (VAT), we measured the VAT thickness and the VAT area. The VAT thickness was determined by calculating the retro-renal fat thickness as the distance from the left kidney to the nearest posterior inner abdominal wall on the axial slice passing through the left renal vein [20], and the VAT area was measured with 3D Slicer version 5.1.0 software based on a semiautomatic thresholding segmentation method in which VAT is defined as fat within the peritoneal cavity. Regarding the latter (SAT), we measured the anterior SAT thickness in the abdomen as the largest anterior distance on the axial slice between the rectus abdominis and the skin at the level of the umbilicus [20], the posterior SAT thickness as the distance between the left iliac crest and the posterior skin [20], the total sum of the posterior and anterior SAT thickness, and the SAT area, defined as the fat between the skin and the underlying muscular layer, demarcated on 3D Slicer version 5.1.0 software by manually tracing the inner border of the transversus abdominis muscle. In addition, we considered the total fat area, regardless of the visceral and the subcutaneous distribution. In particular, the VAT and SAT areas can be segmented using fat-specific thresholds (−150 to −30 HU) according to previously published works [19,21,22]. With the same software, we also measured the mean density (HU) of the VAT, SAT, and total adipose tissue. As a reference, the CT measurements of the fat thicknesses and fat areas are shown in Figure 1.

Moreover, in each CT, on the axial slice, we measured the area of both common femoral arteries to verify if there is a relationship between the common femoral artery area and the fat representation that could explain eventual differences in vascular complications in the femoral site of access between patients with major fat representation and those with minor fat representation.

Secondly, in order to study the role of sarcopenia as a predictive factor in post-TAVI procedure outcomes, we evaluate the right psoas muscle diameter measured on a single axial CT slice at the level of the L3 transverse process. We considered the largest diameter perpendicular to the longest anteroposterior axis of the muscle normalized to the stature by dividing it by the height (mm/m) because this ratio (psoas/height) reflects the whole-body skeletal muscle mass, as confirmed in previous studies [23,24,25].

**Figure 2:**
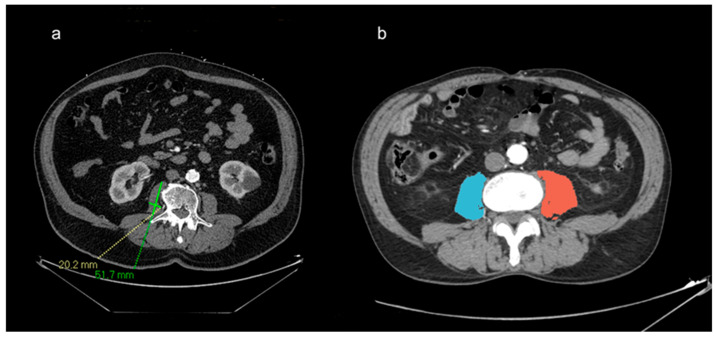
(**a**) Right psoas muscle diameter measured on a single axial CT slice at the level of the L3 transverse process by considering the largest diameter perpendicular to the longest anteroposterior axis of the muscle. (**b**) Area of both psoas muscles segmented with 3D Slicer version 5.1.0. software using muscle-specific thresholds.

We also calculated the area of both psoas muscles with 3D Slicer version 5.1.0 software. The psoas muscle areas were segmented at the same level using muscle-specific thresholds (−29 to 150 HU) in accordance with previously published works [26]. The psoas CT measurements are shown in Figure 2.

Thirdly, we compared the known BMI values of these 85 patients with the CT measurements of fat parameters, including the VAT thickness, anterior SAT thickness, posterior SAT thickness, total SAT thickness, VAT area, SAT area, and total fat area. Moreover, we analyzed the relationships between the fat thicknesses and fat areas measured on the CT.

We also studied how the fat density changed with different BMI values, fatty tissue thicknesses, and fatty tissue areas.

Finally, in order to evaluate the roles of obesity and sarcopenia as predictive factors of outcomes after the TAVI procedure, we compared the known BMI values of patients and the CT measurements (VAT thickness, VAT area, anterior SAT thickness, posterior SAT thickness, total SAT thickness, SAT area, total fat area, psoas/height, psoas area, SAT density, VAT density, and total fat density) with the long-term complications recorded during 24 months of follow-up and the early complications registered during hospitalization. Among the long-term complications, we included mortality, cardiovascular events, and cerebrovascular events, like stroke and TIA, which had occurred by the end of the 24-month follow-up period. The early complications included intrahospital mortality, peri-prosthetic endoleak, femoral stent placement, femoral bleeding, blood transfusion, prolonged hypotension, bundle branch block, atrioventricular type 1, type 2, and type 3 blocks, and PPM implantation after TAVI. In addition, the number of days of hospitalization and the number of days of intensive care were assessed.

## 3. Statistical Analysis

First, the descriptive statistics were assessed: for the qualitative variables, the absolute and percentage frequencies are reported, while for the quantitative variables, the means and standard deviation are reported. In order to compare the numerical variables between two groups, *t*-tests with independent samples were used. First, we carried out the Levene test to determine whether the classic or robust test should be used, and the Mann–Whitney U (non-parametric) test was used when at least one of the two groups had a small number (less than 30). To verify the existence of an association between two categorical variables, the Chi-Square test was used, and, in cases of significance, the Cramer V index was used to quantify the strength of the association. On the other hand, regarding the relationships among the quantitative variables, the Pearson correlation was used. Finally, the ROC curve was used to find cut-off values for some qualitative variables (two groups) with respect to the numerical variables. For all tests, the significance level considered was *p* < 0.05. The statistical analysis is conducted with IBM SPSS v28 software. The statistical analysis and full results are available as Supplementary Materials.

## 4. Results

The baseline characteristics of the study population, including gender, BMI, and frequencies of long-term and early complications, are shown in Table 1.

The average values of the CT measurements are reported in Table 2.

All the detailed results and statistical findings are collected in the Supplementary Materials document for manuscripts’ brevity requirements.

Regarding the comparison between the BMI and average values for each CT measurement, we demonstrate that the mean of each variable (VAT thickness, anterior SAT thickness, posterior SAT thickness, total SAT thickness, VAT area, SAT area, and total fat area) was higher in the BMI > 25 group than in the BMI < 25 group (all *t*-tests with *p* < 0.05).

Among the quantitative CT measurements of fat, the correlation matrix shows positive and statistically significant relationships (*p* < 0.05) among the fat variables (VAT thickness, anterior SAT thickness, posterior SAT thickness, total SAT thickness, VAT area, SAT area, and total fat area). This means that as one increases, the other one increases, and vice versa.

Our results also show higher mean density values for the VAT, SAT, and total fat in the BMI < 25 groups (the *t*-test rejects the equality of the means with *p* < 0.05). In addition, regarding the Pearson correlation between the adipose tissue density (VAT, SAT, and total fat density) and fat areas (total fat area, SAT area, and VAT area), we observed a statistically significant moderate negative relationship (*p* < 0.05); this means that as one variable decreases, the other one increases, and vice versa.

In order to identify cut-off values for the SAT and VAT density to indicate the values above which mortality is expected within the 24-month follow-up period, the ROC curve analysis was used. For females, a significant result was not obtained (*p* > 0.05) in either case, while for males, there was a significant result (*p* < 0.05) with a moderate level of accuracy in both cases (AUC for SAT density is equal to 0.785, while for VAT density, it is equal to 0.856). In particular, the cut-off of SAT density for males was −88.8750 (sensibility: 0.750; specificity: 0.769), while the cut-off of VAT density for males was −83.0905 (sensibility: 0.833; specificity: 0.885).

The statistical analysis of the association between BMI and the occurrence of long-term complications showed that BMI > 25 was significantly related to fewer adverse cardiac events, cerebrovascular events, and instances of mortality within the 24-month follow-up period (Chi-squared test *p* < 0.05).

Indeed, in the BMI > 25 group, the percentages of adverse cardiac events, cerebrovascular events, and mortality in the 24-month follow-up period were 12%, 8%, and 12%, respectively, versus 51.4%, 51.4%, and 62.9% for the adverse long-term complications registered in the BMI < 25 groups.

**Figure 3:**
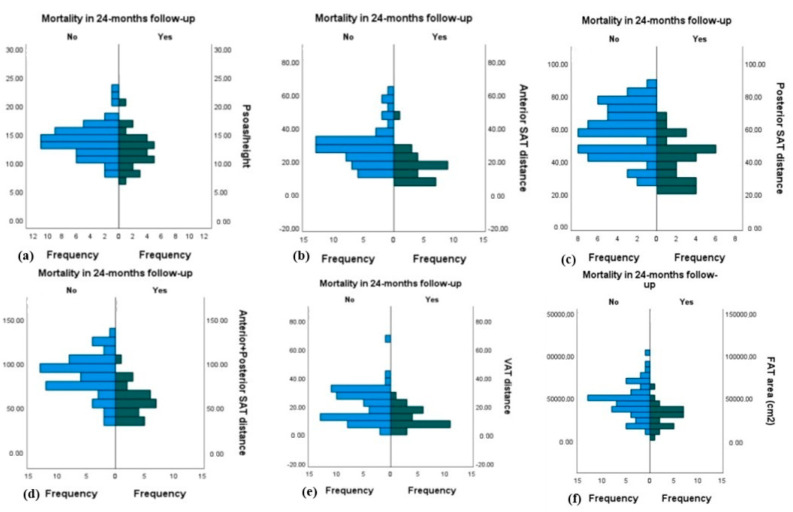
This graph shows the distributions of the psoas/height ratio (**a**), anterior SAT distance (**b**), posterior SAT distance (**c**), anterior + posterior SAT distance (**d**), VAT distance (**e**), and total fat area (**f**) in relation to the absence and presence of mortality within the 24-month follow-up period. As the diagrams show, the blue columns, which represent the pre-TAVI CT values for the psoas, VAT, and SAT measurements in patients who did not die in the 24-month follow-up period, show higher values than the green columns, which represent the same pre-TAVI CT measurements in patients who died during the 24-month follow-up period. These graphs confirm that overweight and obesity are good prognostic factors of TAVI outcomes, and sarcopenia is a poor prognostic factor.

Regarding the associations between adverse cardiac events in the 24-month follow-up period and the CT measurements, the hypothesis of equal medians (*p* < 0.05) was rejected for the variables: psoas/height, anterior SAT thickness, posterior SAT thickness, total SAT thickness, total fat area, SAT area, and both right and left psoas muscle area. We report significantly higher psoas/height ratios and larger areas of psoas muscle in patients who did not experience cardiovascular events. The other CT measurements (anterior SAT thickness, posterior SAT thickness, total SAT thickness, total fat area, and SAT area) also had significantly higher values in patients who did not experience adverse cardiac events in the 24-month follow-up period. Regarding the association between cerebrovascular events in the 24-month follow-up period and CT measurements, the hypothesis of equal medians (*p* < 0.05) was rejected for the psoas/height, anterior SAT thickness, posterior SAT thickness, total SAT thickness, VAT thickness, total fat area, SAT area, and both right and left psoas muscle areas. In all these cases, we confirm that higher values for these CT measurements were significantly associated with lower rates of cerebrovascular events. Regarding the association between mortality within the 24-month follow-up period and CT measurements, we confirm that higher psoas/height, anterior SAT thickness, posterior SAT thickness, total SAT thickness, VAT thickness, total fat area, SAT area, and VAT area values were significantly associated with a lower mortality rate (*p* < 0.05). As a result of this analysis, we confirm that sarcopenia can be considered a poor prognostic factor for outcomes following the TAVI procedure, while obesity can be considered a good prognostic factor. The results concerning the association between mortality within the 24-month follow-up period and CT measurements are shown in Figure 3.

Subsequently, we used the ROC curve to investigate the cut-off values for the total SAT thickness, VAT thickness, total fat area, SAT area, and VAT area above which long-term complications (cardiac events, cerebrovascular events, and mortality within the 24-month follow-up period) are not expected. The identified cut-off points for the CT measurements are shown in Table 3.

Regarding the statistical analysis of the association between BMI and early complications, a BMI > 25 was significantly related to a lower blood transfusion requirement (Chi-squared test *p* < 0.05). At the same time, there was a statistically significant association between the requirement for blood transfusion and some CT parameters, such as the anterior SAT thickness, posterior SAT thickness, total SAT thickness, VAT thickness, total fat area, SAT area, and VAT area (Mann–Whitney U test with *p* < 0.05). In particular, higher CT measurements were associated with a lower blood transfusion frequency. Moreover, a BMI > 25 was significantly related to the requirement for <5 days of hospitalization (Chi-squared test *p* < 0.05), while a lower BMI < 25 was related to the requirement for >5 days of hospitalization. In addition, when we compared the CT measurements of the group that required <5 days of hospitalization and the group that required >5 days of hospitalization, we found that the differences between the average CT measurement values were statistically different (*t*-test *p* < 0.05) for the posterior SAT thickness and the total (anterior + posterior) SAT thickness. In addition, regarding the correlations between the number of intensive care days required and the other CT measurements, we observed a statistically significant negative relationship (Pearson correlation *p* < 0.05) for the anterior SAT thickness variable. This means that as one variable decreases, the other increases, and vice versa. These results confirm the validity of the ‘obesity paradox’ for early complications, including the requirement for blood transfusion, days of hospitalization, and days of intensive care. Regarding the other early complications/intrahospital mortality, periprosthetic endoleak, femoral stent placement, femoral bleeding, prolonged hypotension, bundle branch block, type 1, type 2, type 3 atrioventricular blocks, and PPM implantation after TAVI, no significant correlations with the BMI or the other CT measurements were found.

## 5. Discussion

BMI is the most widely used anthropometric measurement in clinical practice to define generalized obesity [27]; however, CT and MRI are the gold standard measurement tools for the quantitative assessment of visceral adipose tissue [28]. In particular, CT allows for the accurate assessment of VAT and SAT by measuring fat distances and areas (anterior SAT thickness, posterior SAT thickness, VAT thickness, SAT area, VAT area, and the total fat area), which can be conducted easily and with good reproducibility [29]. Our study identified a significant positive correlation between the BMI and all VAT and SAT measurements performed by CT. The results of several previous studies agree with our results [26,30]. In particular, a recent Japanese study in which the CT assessment of VAT and SAT areas was performed prior to surgical implantation of the aortic valve showed significant positive correlations of the BMI with both the SAT and VAT [19]. In addition, in accordance with different studies [19,29,30], our work showed significant positive correlations between the measurements of fat areas and distances between each other. During our analysis, we also performed a qualitative assessment of the VAT and SAT densities. Our results highlight significant negative correlations of the VAT and SAT densities with the BMI and CT measurements of the VAT and SAT areas and thicknesses. This finding agrees with the results of Kenichi Shibata et al., who found negative correlations between the CT density of adipose tissue and the BMI, VAT area, and SAT area [19]. The evaluation of the ROC curve in our research also showed densities equal to −88.8750 HU and −83.0905 as the cut-off values for the SAT and VAT density, respectively, in males, above which an increased mortality rate was observed within the 24-month follow-up period. Similarly, Kenichi Shibata et al. calculated specific cut-off values for the CT density for the VAT and SAT in male and female patients undergoing surgical aortic valve replacement, above which there is an increase in the mortality rate [19]. In addition, other previous studies correlated a higher adipose tissue densitometric value with an increased all-cause mortality value and worse outcomes, especially in older patients [31]. The reason for this correlation is still unclear. The production of endocrine molecules by adipose tissue has been suggested as an explanation. Indeed, previous studies have correlated the adipose tissue density with the serum leptin level [32], and lower serum leptin levels were correlated with an increase in all-cause mortality [33]. However, the results of these studies are in contrast with the evaluation conducted by Shah R et al., who reported a negative correlation between the densitometric values of SAT and VAT with increases in markers of adipose tissue dysfunction, such as the C-reactive protein, increases in markers of insulin resistance, and reduced adiponectin levels [34]. Moreover, our study confirms the existence of the ‘obesity paradox’ by showing a significant reduction in long-term complications following the TAVI procedure in patients with a BMI > 25 compared with normal-weight patients. We also identified baseline cut-off values for the total SAT thickness, VAT thickness, SAT area, VAT area, and total fat area, above which long-term complications (adverse cardiac events, cerebrovascular events, and mortality within the 24-month follow-up period) are less likely. Our results regarding the ‘obesity paradox’ are similar to those found by Konigstein et al. [10] and Abawi et al. [11]. In contrast, Van der Boon et al. demonstrated a relationship between obesity and a reduction in 30-day mortality after TAVI but without a certain impact on long-term results [35]. In addition, regarding the effect of sarcopenia on the outcomes of TAVI candidate patients, we confirm its role as a poor prognostic factor, a result that has already been shown by previous authors [36]. Our results also suggest a reverse correlation between the BMI and some early complications after TA-VI. In particular, we recorded a significantly shorter hospitalization period (<5 days), a significantly lower average number of intensive care days, and fewer blood transfusions in the BMI > 25 group.

Our work presents some limitations. First of all, it is a retrospective study that was conducted in a single center with a limited number of patients. It would be very interesting to perform a multicenter study with more patients. Then, the same statistical analysis could be conducted with the patients divided into different groups to reflect different grades of obesity. Secondly, we did not identify a cut-off value for fat density for women above which mortality is expected. It was not possible to create a significant ROC curve because the value of fat density in women varies a lot. It would be interesting to identify whether this can be explained hormonally due to the fact that estrogens can reduce water loss, eventually changing the tissue density seen in CT measurements. Thirdly, our study did not include younger patients; it could be interesting to conduct new studies that include more younger patients who are not fragile in order to also analyze the frailty aspect that is related to sarcopenia. Future research could also benefit from exploring pre- and post-procedural dietary modifications and other parameters in relation to TAVI outcomes. Another limitation was that acute cerebrovascular events, such as aortic arch plaques/calcifications, were not evaluated in this study, but future research/multicentric collaborations with a broader patient cohort could address this.

In conclusion, we confirm that being overweight and obese are good prognostic factors, and sarcopenia is a poor prognostic factor for outcomes following the TAVI procedure. We focused on the scientific validation of an easy and fast way to measure fat and skeletal muscle mass using CT in order to better predict the outcomes of patients undergoing TAVI. Our aim was to develop a rapid and accurate evaluation method for prognostic factors that can easily be adapted into the daily workflow of radiologists.

Even if the CT measurements of fat can help to predict the outcomes of the TAVI procedure, it will still not be mandatory to add these values to the radiological report. While some parameters, such as distances and ROIs, can be measured relatively easily without the need for external software, others do indeed require post-processing techniques, which may not be feasible in all clinical settings. Moreover, even if there are radiological markers that allow radiologists to perform standard measurements of VAT and SAT, this evaluation of fat will remain patient-dependent because different patients could have different fat distributions. Over time, and with the advent of machine learning, more accurate and standardized methods for measuring fat density may be developed, which could allow the radiologist to better predict the outcomes of the TAVI procedure.

## Figures and Tables

**Table 1:** The gender, BMI < 25 or >25, and presence of long-term and early complications after TAVI.

Category		Frequency	Percentage (%)
GENDER	Female	47	55.3
	Male	38	44.7
BMI	<25	35	41.2
	≥25	50	58.8
AGE	BMI < 25	82 (mean)	-
	BMI ≥ 25	80 (mean)	-
Adverse cardiac events in 24-months follow-up	Absent	61	71.8
	Present	24	28.2
Cerebrovascular events in 24-months follow-up	Absent	63	74.1
	Present	22	25.9
Mortality in 24-months follow-up	Absent	57	67.1
	Present	28	32.9
Intrahospital mortality	Absent	79	92.9
	Present	6	7.1
Hospitalization days after TAVI	≤5	50	58.8
	>5	35	41.2
Peri-prosthetic endoleak	Absent	49	57.6
	Present	36	42.4
Femoral stent placement	Absent	77	90.6
	Present	8	9.4
Femoral bleeding	Absent	81	95.3
	Present	4	4.7
Blood transfusion	Absent	59	69.4
	Present	26	30.6
Prolonged hypotension	Absent	74	87.1
	Present	11	12.9
Bundle branch block	Absent	71	83.5
	Present	14	16.5
Atrioventricular block type 1	Absent	75	88.2
	Present	10	11.8
Atrioventricular block type 2	Absent	83	97.6
	Present	2	2.4
Atrioventricular block type 3	Absent	76	89.4
	Present	9	10.6
PPM implantation after TAVI	Absent	67	78.8
	Present	18	21.2

**Table 2:** CT measurements of the VAT, SAT, common femoral arteries, and psoas muscle are reported as their mean values in the full group of patients and in the subgroups divided by a BMI cut-off of 25.

**Fat Measurements**	**Mean**	**SD**	**BMI Subgroups**	**BMI < 25**		**BMI 25**	
Anterior SAT distance	24.9	11.98		**Mean**	**SD**	**Mean**	**SD**
Posterior SAT distance	52.16	16.74	Anterior SAT distance	15.51	5.92	31.47	10.7
Anterior+Posterior SAT distance	77.06	25.1 1	Posterior SAT distance	37.21	8.98	62.63	12.38
VAT thickness	17.68	11.02	Anterior+Posterior SAT distance	52.72	12.5	94.1	15.9
FAT area (cm^2^)	410.9	194	VAT thickness	10.69	5.72	22.43	11.25
SAT area (cm^2^)	255.5	132	FAT area (cm^2^)	263.7	116	513.9	170
VAT area (cm^2^)	154.6	81.23	SAT area (cm^2^)	163.4	73.3	320	125.8
FAT density (HI-I)	−8526	9.67	VAT area (cm^2^)	97.78	57.9	193.2	71.85
SAT density (HI-I)	−86.48	10.41	FAT mean density (HO)	−82.02	10.5	−87.52	8.45
VAT density (HI-I)	−82.76	9.86	SAT mean density (HO)	−83.69	11.3	−88.44	9.38
Right common femoral artery area (mm^2^)	56.52	21.34	VAT mean density (HO)	−79.54	10.4	−85.02	8.9
Left common femoral artery area (mm^2^)	58.62	20.18	Right common femoral artery area (mm^2^)	57.15	19.7	56.07	22.62
Right Psoas muscle area (cm^2^)	7.57	2.03	Left common femoral artery area (mm^2^)	57.91	20.3	59.1 1	20.31
Left Psoas muscle area (cm^2^)	7.74	2.18					
Psoas/height (HIJ)	13.41	3.1 1					

**Table 3:** The different CT measurement cut-off values for the total SAT thickness, VAT thickness, total fat area, SAT area, and VAT area, above which long-term complications are not expected, are shown in the table. The sensibility, specificity, and type of accur.

		Cardiac Events in 24 Months of Follow-Up	Cerebrovascular Events in 24 Months of Follow-Up	Mortality in 24 Months of Follow-Up
total SAT thickness	Specificity Sensibility	0.787 0.750	0.778 0.818	0.807 0.750
	AUC	0.759 moderate	0.763 moderate	0.836 moderate
	Cut-off	68.00	69.05	69.05
VAT thickness	Specificity Sensibility	-	0.532 0.818	0.536 0.857
	AUC	-	0.673 low	0.727 moderate
	Cut-off	-	17.30	18.95
Total fat area (cm^2^)	Specificity Sensibility	0.574 0.667	0.587 0.727	0.754 0.714
	AUC	0.654 low	0.668 low	0.776 moderate
	Cut-off	385.51	385.51	350.49
SAT area (cm^2^)	Specificity Sensibility	0.607 0.750	0.503 0.682	0.719 0.857
	AUC	0.699 low	0.668 low	0.794 moderate
	Cut-off	232.10	229.51	229.51
VAT area (cm^2^)	Specificity Sensibility	-	-	0.554 0.857
	AUC	-	-	0.701 moderate
	Cut-off	-	-	166.09

## Data Availability

The original contributions presented in this study are included in the article/Supplementary Materials.

## Supplementary Materials

The following supporting information can be downloaded at: https://www.mdpi.com/article/10.3390/jcdd11110368/s1, Detailed statistical analysis.

## Abbreviations

CT	computed tomography
BMI	body mass index
TAVI	transcatheter aortic valve implantation
VAT	visceral adipose tissue
SAT	subcutaneous adipose tissue
HU	Hounsfield units
PPM	permanent pacemaker
MRI	magnetic resonance imaging

## References

[1] Chiocchi M., Ricci F., Pasqualetto M., D’errico F., Benelli L., Pugliese L., Cavallo A.U., Forcina M., Presicce M., De Stasio V. (2020). Role of computed tomography in transcatheter aortic valve implantation and valve-in-valve implantation: Complete review of preprocedural and postprocedural imaging. J. Cardiovasc. Med..

[2] Pugliese L., Ricci F., Luciano A., De Stasio V., Presicce M., Spiritigliozzi L., Di Tosto F., Di Donna C., D’errico F., Benelli L. (2022). Role of computed tomography in transcatheter replacement of ‘other valves’: A comprehensive review of preprocedural imaging. J. Cardiovasc. Med..

[3] Chiocchi M., Pugliese L., D’errico F., Di Tosto F., Cerimele C., Pasqualetto M., De Stasio V., Presicce M., Spiritigliozzi L., Di Donna C. (2022). Transcatheter aortic valve implantation in patients with unruptured aortic root pseudoaneurysm: An observational study. J. Cardiovasc. Med..

[4] Hildebrandt H.A., Mahabadi A.A., Totzeck M., Jánosi R.A., Lind A.Y., Rassaf T., Kahlert P. (2017). Imaging for planning of transcatheter aortic valve implantation. Herz.

[5] Blanke P., Weir-McCall J.R., Achenbach S., Delgado V., Hausleiter J., Jilaihawi H., Marwan M., Nørgaard B.L., Piazza N., Schoenhagen P. (2019). Computed Tomography Imaging in the Context of Transcatheter Aortic Valve Implantation (TAVI)/Transcatheter Aortic Valve Replacement (TAVR): An Expert Consensus Document of the Society of Cardiovascular Computed Tomography. JACC Cardiovasc. Imaging.

[6] Holroyd E.W., Sirker A., Kwok C.S. (2017). British Cardiovascular Intervention Society and National Institute of Cardiovascular Outcomes Research. The Relationship of Body Mass Index to Percutaneous Coronary Intervention Outcomes: Does the Obesity Paradox Exist in Contemporary Percutaneous Coronary Intervention Cohorts? Insights From the British Cardiovascular Intervention Society Registry. JACC Cardiovasc. Interv..

[7] Gruberg L., Weissman N.J., Waksman R., Fuchs S., Deible R., Pinnow E.E., Ahmed L.M., Kent K.M., Pichard A.D., Suddath W.O. (2002). The impact of obesity on the short-term and long-term outcomes after percutaneous coronary intervention: The obesity paradox?. J. Am. Coll. Cardiol..

[8] Foldyna B., Troschel F.M., Addison D., Fintelmann F.J., Elmariah S., Furman D., Eslami P., Ghoshhajra B., Lu M.T., Murthy V.L. (2018). Computed tomography-based fat and muscle characteristics are associated with mortality after transcatheter aortic valve replacement. J. Cardiovasc. Comput. Tomogr..

[9] Riaz H., Khan M.S., Siddiqi T.J., Usman M.S., Shah N., Goyal A., Khan S.S., Mookadam F., Krasuski R.A., Ahmed H. (2018). Association Between Obesity and Cardiovascular Outcomes: A Systematic Review and Meta-analysis of Mendelian Randomization Studies. JAMA Netw. Open.

[10] Konigstein M., Havakuk O., Arbel Y., Finkelstein A., Ben-Assa E., Rubinow E.L., Abramowitz Y., Keren G., Banai S. (2015). The obesity paradox in patients undergoing transcatheter aortic valve implantation. Clin. Cardiol..

[11] Abawi M., Rozemeijer R., Agostoni P., van Jaarsveld R.C., van Dongen C.S., Voskuil M., Kraaijeveld A.O., Doevendans P.A.F.M., Stella P.R. (2017). Effect of body mass index on clinical outcome and all-cause mortality in patients undergoing transcatheter aortic valve implantation. Neth. Heart J..

[12] Yamamoto M., Mouillet G., Oguri A., Gilard M., Laskar M., Eltchaninoff H., Fajadet J., Iung B., Donzeau-Gouge P., Leprince P. (2013). Effect of body mass index on 30- and 365-day complication and survival rates of transcatheter aortic valve implantation (from the FRench Aortic National CoreValve and Edwards 2 [FRANCE 2] registry). Am. J. Cardiol..

[13] Sannino A., Schiattarella G.G., Toscano E., Gargiulo G., Giugliano G., Galderisi M., Losi M.-A., Stabile E., Cirillo P., Imbriaco M. (2017). Meta-Analysis of Effect of Body Mass Index on Outcomes After Transcatheter Aortic Valve Implantation. Am. J. Cardiol..

[14] Lv W., Li S., Liao Y., Zhao Z., Che G., Chen M., Feng Y. (2017). The ‘obesity paradox’ does exist in patients undergoing transcatheter aortic valve implantation for aortic stenosis: A systematic review and meta-analysis. Interact. Cardiovasc. Thorac. Surg..

[15] Bachmann R., Leonard D., Nachit M., Remue C., Orabi N.A., Desmet L., Faber B., Danse E., Trefois P., Kartheuser A. (2018). Comparison between abdominal fat measured by CT and anthropometric indices as prediction factors for mortality and morbidity after colorectal surgery. Acta Gastroenterol. Belg..

[16] De Santo L.S., Moscariello C., Zebele C. (2018). Implications of obesity in cardiac surgery: Pattern of referral, physiopathology, complications, prognosis. J. Thorac. Dis..

[17] Johnson A.P., Parlow J.L., Whitehead M., Xu J., Rohland S., Milne B. (2015). Body Mass Index, Outcomes, and Mortality Following Cardiac Surgery in Ontario, Canada. J. Am. Hear. Assoc..

[18] Friedman J., Lussiez A., Sullivan J., Wang S., Englesbe M. (2015). Implications of sarcopenia in major surgery. Nutr. Clin. Pr..

[19] Shibata K., Yamamoto M., Yamada S., Kobayashi T., Morita S., Kagase A., Tokuda T., Shimura T., Tsunaki T., Tada N. (2020). Clinical Outcomes of Subcutaneous and Visceral Adipose Tissue Characteristics Assessed in Patients Underwent Transcatheter Aortic Valve Replacement. CJC Open.

[20] Goldenberg L., Saliba W., Hayeq H., Hasadia R., Zeina A.-R. (2018). The impact of abdominal fat on abdominal aorta calcification measured on non-enhanced CT. Medicine.

[21] Kashihara H., Lee J.S., Kawakubo K., Tamura M., Akabayashi A. (2009). Criteria of waist circumference according to computed tomography-measured visceral fat area and the clustering of cardiovascular risk factors. Circ. J..

[22] Yoshizumi T., Nakamura T., Yamane M., Islam A.H.M.W., Menju M., Yamasaki K., Arai T., Kotani K., Funahashi T., Yamashita S. (1999). Abdominal fat: Standardized technique for measurement at CT. Radiology.

[23] Shen W., Punyanitya M., Wang Z., Gallagher D., St.-Onge M.-P., Albu J., Heymsfield S.B., Heshka S. (2004). Total body skeletal muscle and adipose tissue volumes: Estimation from a single abdominal cross-sectional image. J. Appl. Physiol..

[24] Hiraoka A., Aibiki T., Okudaira T., Toshimori A., Kawamura T., Nakahara H., Suga Y., Azemoto N., Miyata H., Miyamoto Y. (2015). Muscle atrophy as pre-sarcopenia in Japanese patients with chronic liver disease: Computed tomography is useful for evaluation. J. Gastroenterol..

[25] Kim Y.R., Park S., Han S., Ahn J.H., Kim S., Sinn D.H., Jeong W.K., Ko J.S., Gwak M.S., Kim G.S. (2018). Sarcopenia as a predictor of post-transplant tumor recurrence after living donor liver transplantation for hepatocellular carcinoma beyond the Milan criteria. Sci. Rep..

[26] Mok M., Allende R., Leipsic J., Altisent O.A.-J., del Trigo M., Campelo-Parada F., DeLarochellière R., Dumont E., Doyle D., Côté M. (2016). Prognostic Value of Fat Mass and Skeletal Muscle Mass Determined by Computed Tomography in Patients Who Underwent Transcatheter Aortic Valve Implantation. Am. J. Cardiol..

[27] Shuster A., Patlas M., Pinthus J.H., Mourtzakis M. (2012). The clinical importance of visceral adiposity: A critical review of methods for visceral adipose tissue analysis. Br. J. Radiol..

[28] Borga M., West J., Bell J.D., Harvey N.C., Romu T., Heymsfield S.B., Leinhard O.D. (2018). Advanced body composition assessment: From body mass index to body composition profiling. J. Investig. Med..

[29] Li X.-T., Tang L., Chen Y., Li Y.-L., Zhang X.-P., Sun Y.-S. (2015). Visceral and subcutaneous fat as new independent predictive factors of survival in locally advanced gastric carcinoma patients treated with neo-adjuvant chemotherapy. J. Cancer Res. Clin. Oncol..

[30] Frostberg E., Pedersen M.R., Manhoobi Y., Rahr H.B., Rafaelsen S.R. (2021). Three different computed tomography obesity indices, two standard methods, and one novel measurement, and their association with outcomes after colorectal cancer surgery. Acta Radiol..

[31] Murphy R.A., Register T.C., Shively C.A., Carr J.J., Ge Y., Heilbrun M.E., Cummings S.R., Koster A., Nevitt M.C., Satterfield S. (2013). Adipose tissue density, a novel biomarker predicting mortality risk in older adults. J. Gerontol. A Biol. Sci. Med. Sci..

[32] Kord-Varkaneh H., Fatahi S., Alizadeh S., Ghaedi E., Shab-Bidar S. (2018). Association of Serum Leptin with All-Cause and Disease Specific Mortality: A Meta-Analysis of Prospective Observational Studies. Horm. Metab. Res..

[33] Molnar M.Z., Nagy K., Remport A., Gaipov A., Fülöp T., Czira M.E., Kovesdy C.P., Mucsi I., Mathe Z. (2017). Association Between Serum Leptin Level and Mortality in Kidney Transplant Recipients. J. Ren. Nutr..

[34] Shah R.V., Allison M., Lima J., Abbasi S., Eisman A., Lai C., Jerosch-Herold M., Budoff M., Murthy V. (2016). Abdominal fat radiodensity, quantity and cardiometabolic risk: The Multi-Ethnic Study of Atherosclerosis. Nutr. Metab. Cardiovasc. Dis..

[35] Van der Boon R.M., Chieffo A., Dumonteil N., Tchetche D., Van Mieghem N.M., Buchanan G.L., Vahdat O., Marcheix B., Serruys P.W., Fajadet J. (2013). Effect of body mass index on short- and long-term outcomes after transcatheter aortic valve implantation. Am. J. Cardiol..

[36] Kärkkäinen J.M., Oderich G.S., Tenorio E.R., Pather K., Oksala N., Macedo T.A., Vrtiska T., Mees B., Jacobs M.J. (2021). Psoas muscle area and attenuation are highly predictive of complications and mortality after complex endovascular aortic repair. J. Vasc. Surg..

