# Supplementary material for: Beyond the Obesity Paradox: Analysis of New Prognostic Factors in Transcatheter Aortic Valve Implantation Procedure
Source: J Cardiovasc Dev Dis. 2024 Nov 15;11(11):368. doi: 10.3390/jcdd11110368 (PMC11594374; doi:10.3390/jcdd11110368)
Supplement: Supplementary file 1 [file jcdd-11-00368-s001.zip › jcdd-3228873-supplementary.pdf]

SUPPLEMENTARY MATERIALS

Methodology

First the descriptive statistics are created: for the qualitative variables the absolute and percentage frequencies are reported, while for the quantitative variables the main indices (mean, quartiles, minimum, maximum, variance and standard deviation) are reported and the Shapiro-Wilk test is conducted to check for normal distribution. Only continuous variables (such as densities, distances, areas, etc.) are typically appropriate for normality testing using methods like the Shapiro-Wilk test. Categorical variables (such as gender, adverse events, or mortality) are usually analyzed with frequency counts and percentages rather than tests for normal distribution.

To compare the numerical variables between variables with two groups, either the t-test with independent samples was used, first carrying out the Levene test to verify whether to use the classic or robust test, or the Mann-Whitney U (non-parametric) test, in case at least one of the two groups has a small number (less than 30).

To verify, instead, the existence of an association between two categorical variables, the Chi-Square test was used and, in case of significance, the Cramer V index was used to quantify the strength of the association.

For the relationship between the quantitative variables, on the other hand, the Pearson correlation was used. Finally, to find cut-offs of some qualitative variables (two groups) with respect to numerical variables, the ROC curve was used.

For all tests the significance level considered is $p < 0.05$.

Analyzes were conducted with IBM SPSS v28 software.

Descriptive statistics and Shapiro-Wilk test

Gender

		Frequency	Percent	Valid Percent	Cumulative Percent
Valid	F	47	55,3	55,3	55,3
	M	38	44,7	44,7	100,0
	Total	85	100,0	100,0	

Adverse cardiac events in 24-months follow-up

		Frequency	Percent	Valid Percent	Cumulative Percent
Valid	No	61	71,8	71,8	71,8
	Yes	24	28,2	28,2	100,0
	Total	85	100,0	100,0	

Cerebrovascular events in 24-months follow-up

		Frequency	Percent	Valid Percent	Cumulative Percent
Valid	No	63	74,1	74,1	74,1
	Yes	22	25,9	25,9	100,0
	Total	85	100,0	100,0	

Mortality in 24-months follow-up

		Frequency	Percent	Valid Percent	Cumulative Percent
Valid	No	57	67,1	67,1	67,1
	Yes	28	32,9	32,9	100,0
	Total	85	100,0	100,0	

Intrahospital mortality

		Frequency	Percent	Valid Percent	Cumulative Percent
Valid	No	79	92,9	92,9	92,9
	Yes	6	7,1	7,1	100,0
	Total	85	100,0	100,0	

Hospitalization days after TAVI

		Frequency	Percent	Valid Percent	Cumulative Percent
Valid	<=5	50	58,8	58,8	58,8
	>5	35	41,2	41,2	100,0
	Total	85	100,0	100,0	

c

		Frequency	Percent	Valid Percent	Cumulative Percent
Valid	No	49	57,6	57,6	57,6
	Yes	36	42,4	42,4	100,0
	Total	85	100,0	100,0	

Femoral stent placement

		Frequency	Percent	Valid Percent	Cumulative Percent
Valid	No	77	90,6	90,6	90,6
	Yes	8	9,4	9,4	100,0
	Total	85	100,0	100,0	

Femoral bleeding

		Frequency	Percent	Valid Percent	Cumulative Percent
Valid	No	81	95,3	95,3	95,3
	Yes	4	4,7	4,7	100,0
	Total	85	100,0	100,0	

Blood transfusion

		Frequency	Percent	Valid Percent	Cumulative Percent
Valid	No	59	69,4	69,4	69,4
	Yes	26	30,6	30,6	100,0
	Total	85	100,0	100,0	

Prolonged hypotension

		Frequency	Percent	Valid Percent	Cumulative Percent
Valid	No	74	87,1	87,1	87,1
	Yes	11	12,9	12,9	100,0
	Total	85	100,0	100,0	

Bundle branch block

		Frequency	Percent	Valid Percent	Cumulative Percent
Valid	No	71	83,5	83,5	83,5
	Yes	14	16,5	16,5	100,0
	Total	85	100,0	100,0	

Atrioventricular block type 1

		Frequency	Percent	Valid Percent	Cumulative Percent
Valid	No	75	88,2	88,2	88,2
	Yes	10	11,8	11,8	100,0
	Total	85	100,0	100,0	

Atrioventricular block type 2

		Frequency	Percent	Valid Percent	Cumulative Percent
Valid	No	83	97,6	97,6	97,6
	Yes	2	2,4	2,4	100,0
	Total	85	100,0	100,0	

Atrioventricular block type 3

		Frequency	Percent	Valid Percent	Cumulative Percent
Valid	No	76	89,4	89,4	89,4
	Yes	9	10,6	10,6	100,0
	Total	85	100,0	100,0	

PPM implantation after TAVI

		Frequency	Percent	Valid Percent	Cumulative Percent
Valid	No	67	78,8	78,8	78,8
	Yes	18	21,2	21,2	100,0
	Total	85	100,0	100,0	

BMI

		Frequency	Percent	Valid Percent	Cumulative Percent
Valid	<25	35	41,2	41,2	41,2
	>=25	50	58,8	58,8	100,0
	Total	85	100,0	100,0	

Statistics

		Days of intensive care	Psoas/height
N	Valid	85	85
	Missing	0	0
Mean		3,19	13,41
Std. Deviation		3,25	3,11
Variance		10,56	9,68
Minimum		1	6,69
Maximum		31	23,73
Percentiles	25	2,00	11,22
	50	3,00	13,25
	75	3,00	15,10

Statistics

		Anterior SAT distance	Posterior SAT distance	Anterior+Posterior SAT distance	VAT distance	Right common femoral artery area (mm2)	Left common femoral artery area (mm2)
N	Valid	85	85	85	84	85	85
	Missing	0	0	0	1	0	0
Mean		24,90	52,16	77,06	17,68	56,52	58,62
Std. Deviation		11,98	16,74	25,11	11,02	21,34	20,18
Variance		143,43	280,34	630,50	121,32	455,50	407,38
Minimum		6,80	23,00	32,00	2,70	13,30	11,30
Maximum		62,40	86,90	133,00	65,00	136,00	148,90
Percentiles	25	16,35	40,15	57,55	8,98	43,20	46,65
	50	24,60	49,40	76,80	15,00	56,00	58,00
	75	30,00	64,25	94,00	26,00	65,80	68,90

Statistics

		FAT area (cm2)	SAT area (cm2)	VAT area (cm2)	Right Psoas muscle area (cm2)	Left Psoas muscle area (cm2)
N	Valid	85	85	84	85	85
	Missing	0	0	1	0	0
Mean		41088,82	25552,94	15459,53	757,96	774,75
Std. Deviation		19403,60	13196,89	8123,89	203,41	218,70
Variance		376499811,05	174157889,66	65997608,92	41374,74	47830,72
Minimum		6160,51	4052,95	2107,56	429,96	320,36
Maximum		100181,00	66666,50	37125,60	1217,51	1294,13

Percentiles	25	28577,65	16409,65	8539,52	584,18	606,28
	50	38604,60	23316,60	15107,55	762,64	768,06
	75	51200,10	32161,20	21162,48	922,32	907,80

Statistics

		FAT mean density (HU)
N	Valid	85
	Missing	0
Mean		-85,26
Std. Deviation		9,67
Variance		93,51
Minimum		-102,12
Maximum		-55,93
Percentiles	25	-91,03
	50	-88,00
	75	-78,00

Statistics

		SAT mean density (HU)
N	Valid	85
	Missing	0
Mean		-86,48
Std. Deviation		10,41
Variance		108,31
Minimum		-105,15
Maximum		-54,40
Percentiles	25	-93,84
	50	-89,00
	75	-77,83

Statistics

		VAT mean density (HU)
N	Valid	85
	Missing	0
Mean		-82,76
Std. Deviation		9,86
Variance		97,20
Minimum		-100,13
Maximum		-58,86

Percentiles	25	-90,00
	50	-85,00
	75	-76,14

Statistics

		Right Psoas muscle mean density (HU)
N	Valid	85
	Missing	0
Mean		45,69
Std. Deviation		7,80
Variance		60,85
Minimum		28,87
Maximum		66,92
Percentiles	25	40,63
	50	46,89
	75	50,07

Statistics

		Left Psoas muscle mean density (HU)
N	Valid	85
	Missing	0
Mean		46,84
Std. Deviation		8,28
Variance		68,54
Minimum		26,23
Maximum		67,03
Percentiles	25	40,80
	50	47,26
	75	50,64

Grafici descrittive

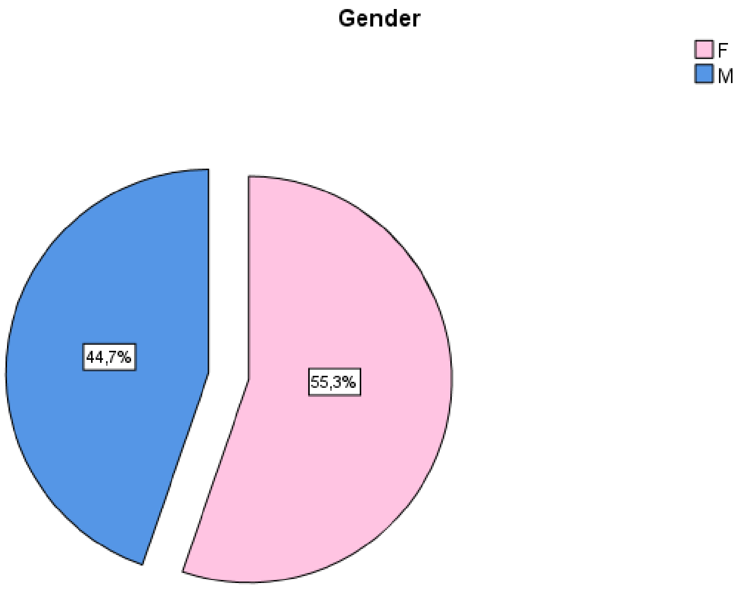

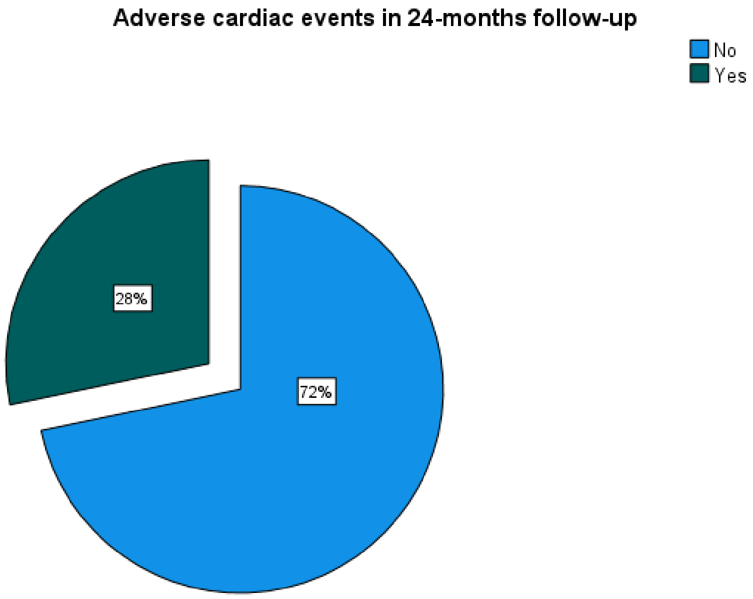

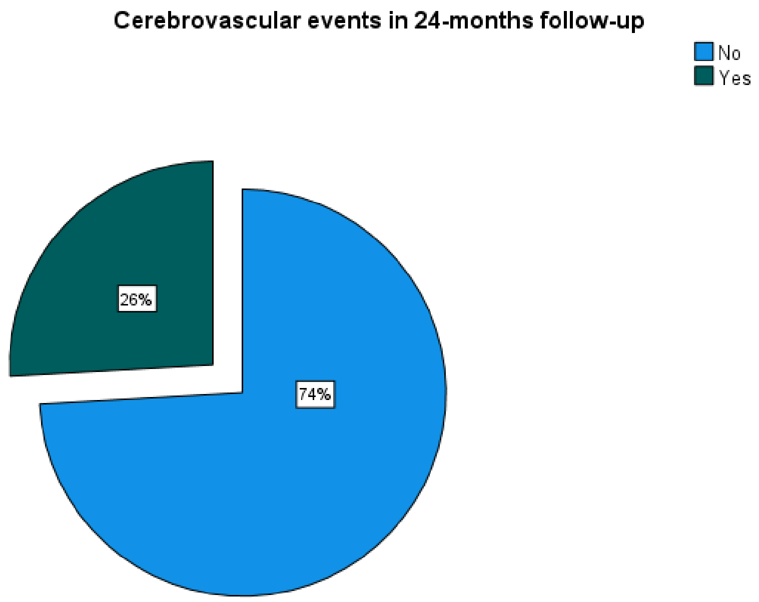
Cerebrovascular events in 24-months follow-up
No
Yes
26%
74%

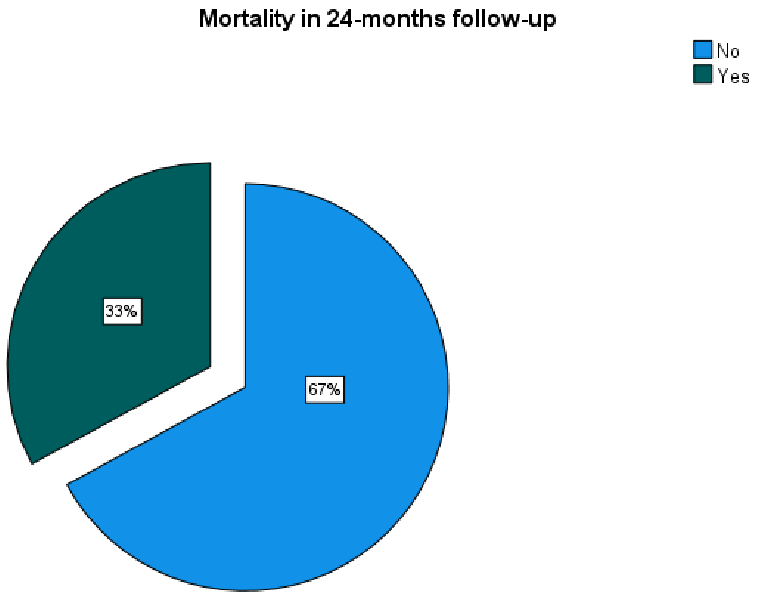
Mortality in 24-months follow-up
No
Yes
33%
67%

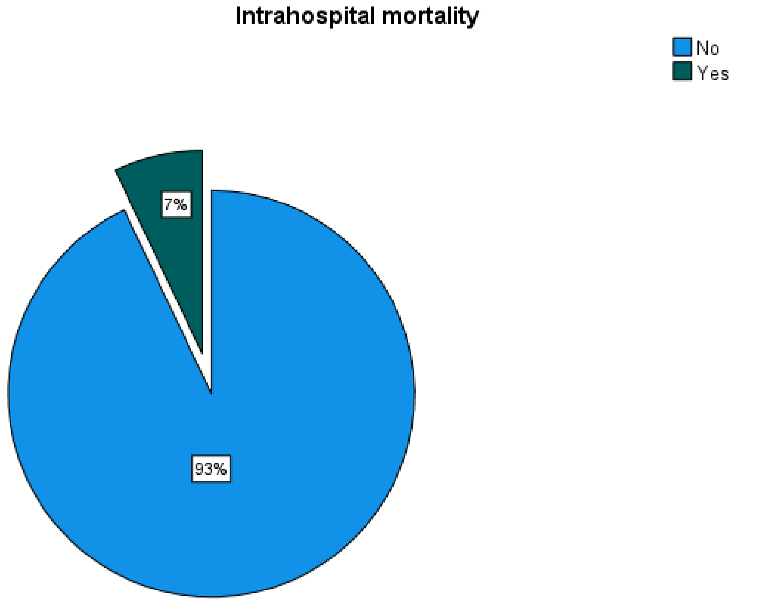
Intrahospital mortality
No
Yes
7%
93%

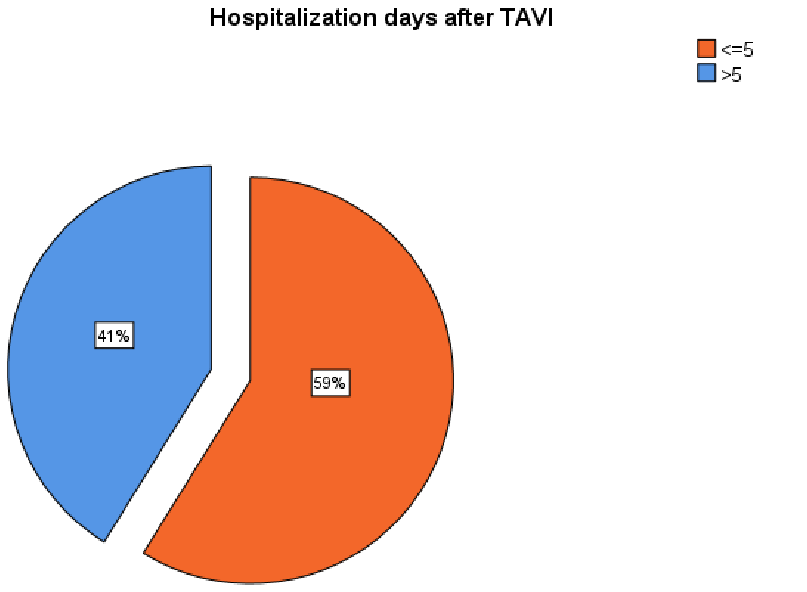
Hospitalization days after TAVI
<=5
>5
41%
59%

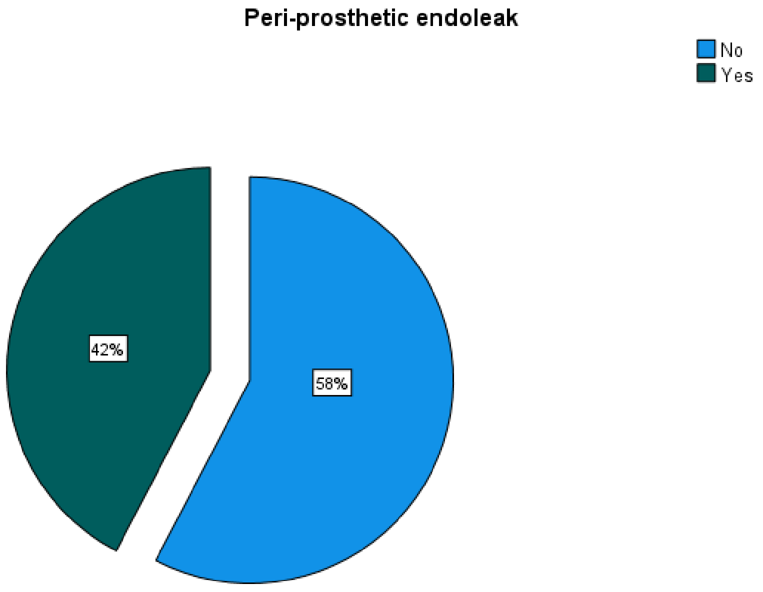
Peri-prosthetic endoleak
No
Yes
42%
58%

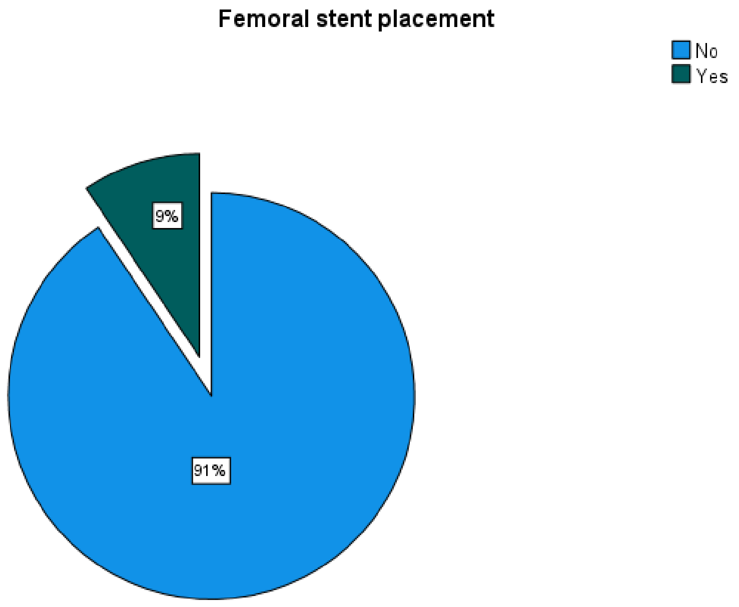
Femoral stent placement
No
Yes
9%
91%

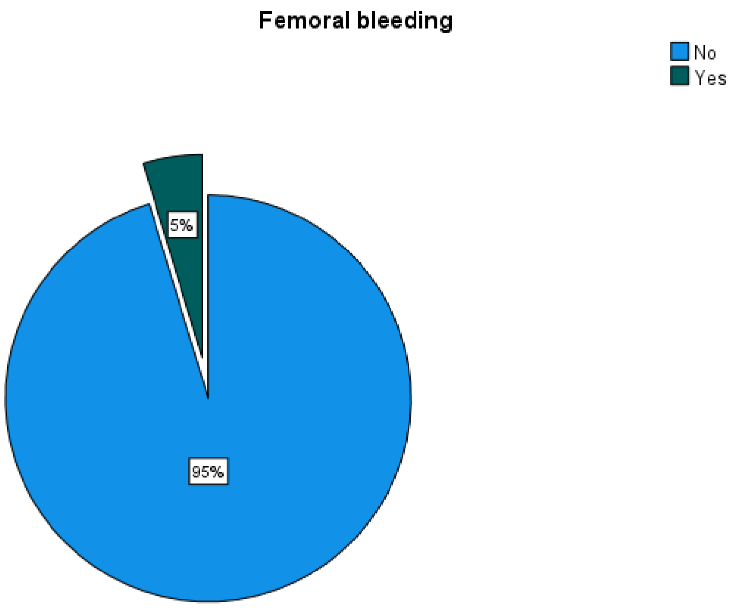
Femoral bleeding
No
Yes
5%
95%

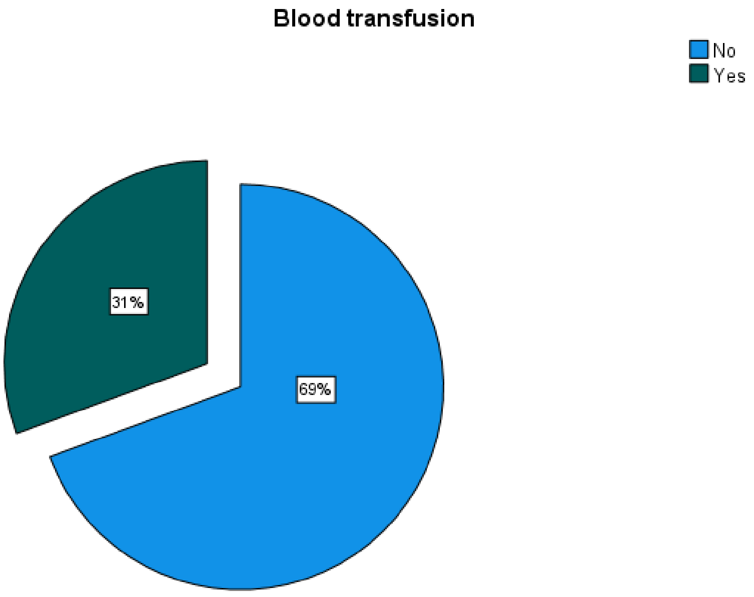
Blood transfusion
No
Yes
31%
69%

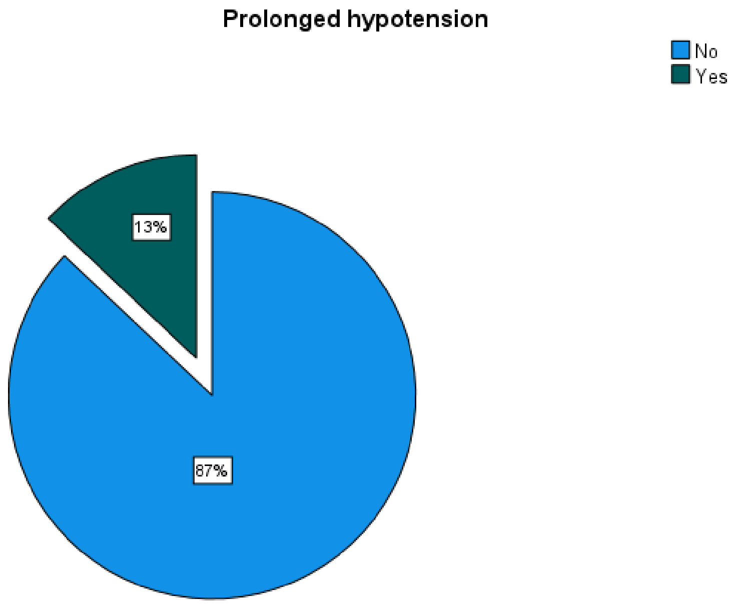
Prolonged hypotension
No
Yes
13%
87%

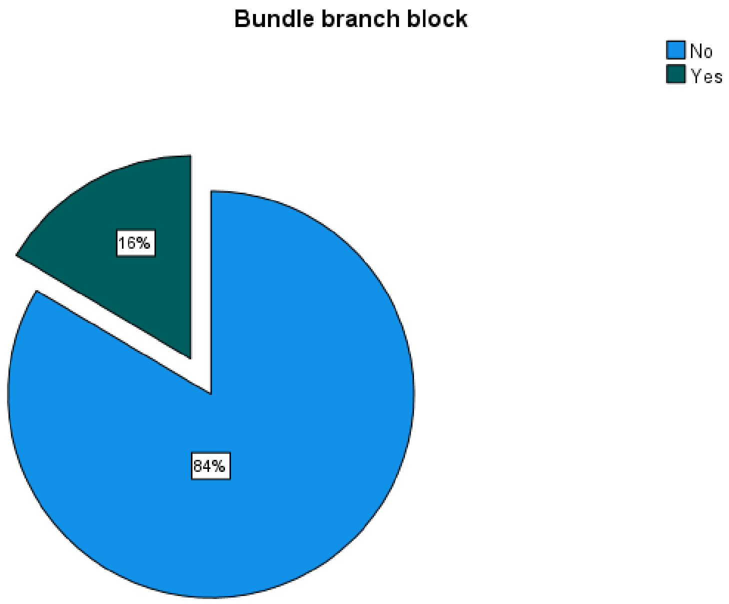
Bundle branch block
No
Yes
16%
84%

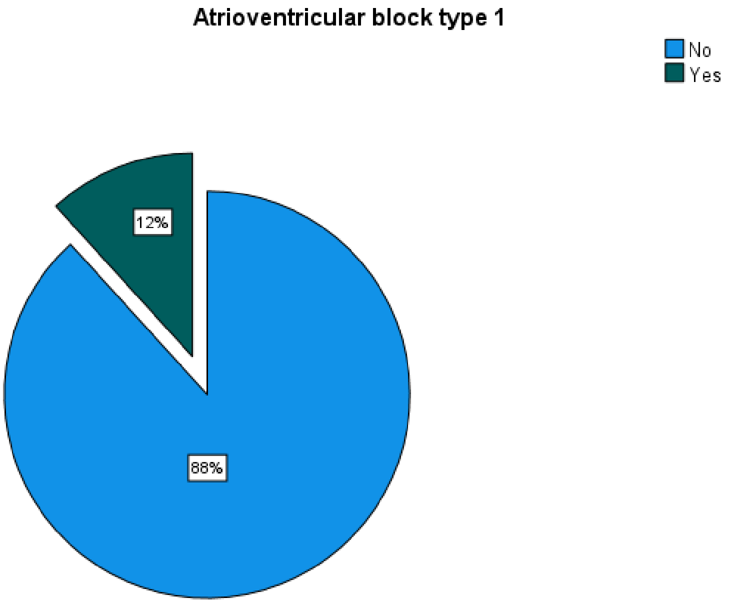
Atrioventricular block type 1
No
Yes
12%
88%

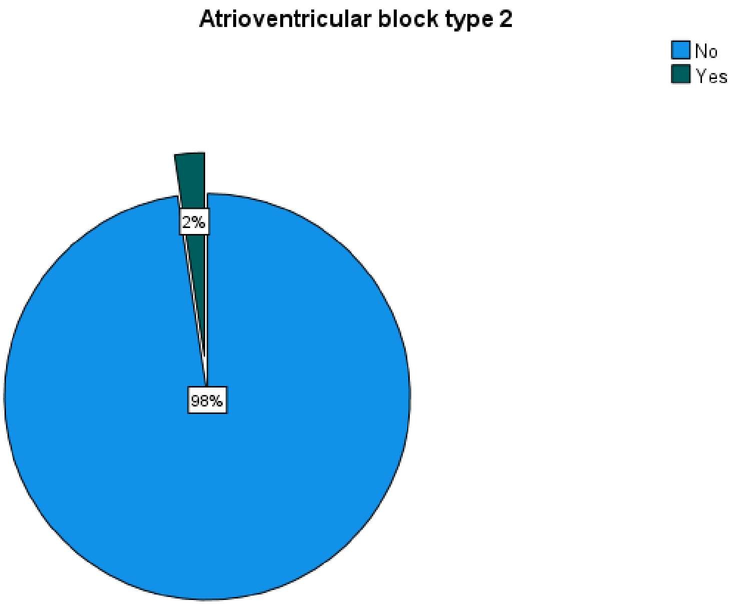
Atrioventricular block type 2
No
Yes
2%
98%

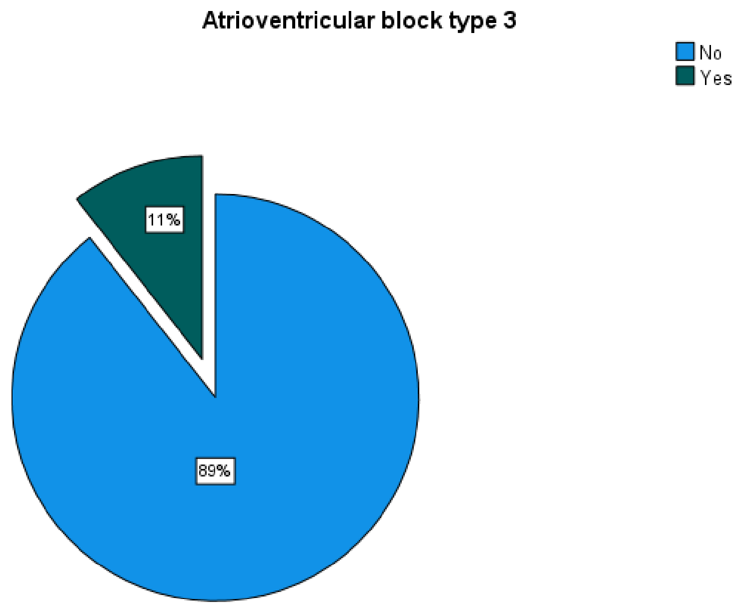
Atrioventricular block type 3
No
Yes
11%
89%

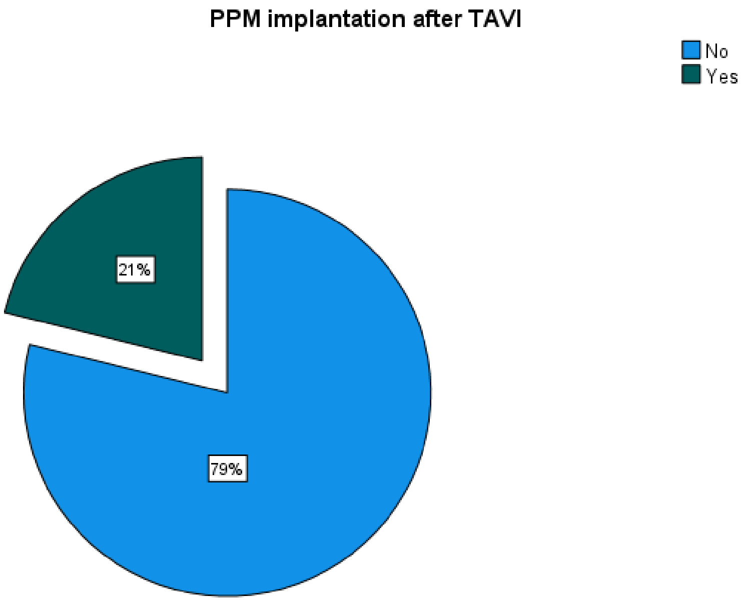
PPM implantation after TAVI
No
Yes
21%
79%

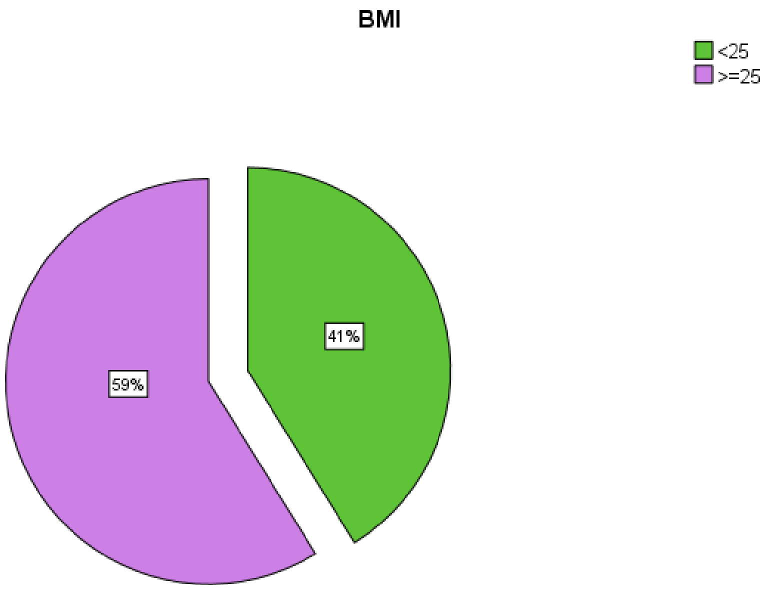

Shapiro-Wilk test for continuous variables

**Variable**	**Shapiro-Wilk W**	**p-value**
Days of Intensive Care	0,985	0,451
Psoas/Height	0,991	0,807
Anterior SAT Distance	0,991	0,818
Posterior SAT Distance	0,993	0,926
Anterior+Posterior SAT Distance	0,988	0,626
VAT Distance	0,975	0,098
Right Common Femoral Artery Area	0,986	0,503
Left Common Femoral Artery Area	0,985	0,423
FAT Area	0,978	0,158
SAT Area	0,974	0,086
VAT Area	0,993	0,946
Right Psoas Muscle Area	0,993	0,923
Left Psoas Muscle Area	0,992	0,877
FAT Mean Density	0,983	0,319
FAT Median Density	0,969	0,038
FAT Density Std Deviation	0,984	0,352
SAT Mean Density	0,993	0,935
SAT Median Density	0,992	0,901

SAT Density Std Deviation	0,991	0,843
VAT Mean Density	0,983	0,333
VAT Median Density	0,991	0,834
VAT Density Std Deviation	0,981	0,259
Right Psoas Muscle Mean Density	0,993	0,938
Left Psoas Muscle Mean Density	0,987	0,584

T-tests with independent samples with BMI as Dichotomic Variable for fat measurements

Independent sample t-tests are performed to verify if there is a statistically significant difference in the mean of the numerical variables in the two subgroups of the dichotomous variable (only two categories).
The null hypothesis of the independent-sampled t-test is that the means are the same in the two groups while the alternative hypothesis is that the means differ significantly in population.

The null hypothesis of equal averages is rejected, concluding that the averages are significantly different, if the p-value is less than 0.05.

Note: before the t-test, the Levene test is performed to verify the null hypothesis of equal variances in the two subgroups. If this assumption is violated (p <0.05), the robust t-test is carried out, i.e. the results are commented on in the second line of the output.

Group Statistics

	BMI	N	Mean	Std. Deviation	Std. Error Mean
Anterior SAT distance	<25	35	15,51	5,92	1,00
	>=25	50	31,47	10,70	1,51
Posterior SAT distance	<25	35	37,21	8,98	1,52
	>=25	50	62,63	12,38	1,75
Anterior+Posterior SAT distance	<25	35	52,72	12,52	2,12
	>=25	50	94,10	15,90	2,25
VAT distance	<25	34	10,69	5,72	,98
	>=25	50	22,43	11,25	1,59
FAT area (cm2)	<25	35	26372,94	11610,65	1962,56
	>=25	50	51389,94	16997,82	2403,85
SAT area (cm2)	<25	35	16340,77	7329,60	1238,93
	>=25	50	32001,46	12578,95	1778,93

VAT area (cm2)	<25	34	9778,04	5785,05	992,13
	>=25	50	19322,94	7185,02	1016,12

Independent Samples Test

		Levene's Test for Equality of Variances		t-test for Equality of Means							
						Significance				95% Confidence Interval of the Difference	
		F	Sig.	t	df	One-Sided p	Two-Sided p	Mean Difference	Std. Error Difference	Lower	Upper
Anterior SAT distance	Equal variances assumed	4,641	,034	-8,002	83	<,001	<,001	-15,96	1,99	-19,93	-11,99
	Equal variances not assumed			-8,800	79,364	<,001	<,001	-15,96	1,81	-19,58	-12,35
Posterior SAT distance	Equal variances assumed	1,742	,191	-10,376	83	<,001	<,001	-25,41	2,45	-30,29	-20,54

	Equal variances not assumed			-10,967	82,867	<,001	<,001	-25,41	2,32	-30,02	-20,81
Anterior+Posterior SAT distance	Equal variances assumed	1,390	,242	-12,852	83	<,001	<,001	-41,38	3,22	-47,78	-34,98
	Equal variances not assumed			-13,402	81,779	<,001	<,001	-41,38	3,09	-47,52	-35,24
VAT distance	Equal variances assumed	10,089	,002	-5,606	82	<,001	<,001	-11,74	2,10	-15,91	-7,57
	Equal variances not assumed			-6,282	76,844	<,001	<,001	-11,74	1,87	-15,46	-8,02
FAT area (cm2)	Equal variances assumed	2,737	,102	-7,554	83	<,001	<,001	-25016,99	3311,66	-31603,76	-18430,24
	Equal variances not assumed			-8,062	82,968	<,001	<,001	-25016,99	3103,25	-31189,28	-18844,72
SAT area (cm2)	Equal variances assumed	6,729	,011	-6,614	83	<,001	<,001	-15660,69	2367,72	-20369,99	-10951,39

	Equal variances not assumed			-7,224	80,700	<,001	<,001	-15660,69	2167,84	-19974,26	-11347,12
VAT area (cm2)	Equal variances assumed	1,885	,174	-6,450	82	<,001	<,001	-9544,90	1479,79	-12488,68	-6601,12
	Equal variances not assumed			-6,721	79,574	<,001	<,001	-9544,90	1420,14	-12371,31	-6718,49

The Levene test is accepted ($p > 0.05$) for the Posterior SAT distance, Anterior + Posterior SAT distance, FAT area cm2 and VAT area cm2 variables, mentre per le altre viene rifiutato ($p < 0.05$).

Regarding the means, regardless of the classic or robust test, the null hypothesis of equality is rejected in all cases ($p < 0.05$), i.e. the means of each variable are higher in the BMI group> = 25 than in the group < 25.

Pearson’s correlations between fat measurements

Pearson's correlation (r) is an index that varies between -1 and +1: the closer, in absolute value, to 1, the stronger the linear relationship between the two numerical variables. The test relating to this index has linear independence as a null hypothesis (r = 0), while the alternative hypothesis is that there is linear dependence (r ≠ 0); the test will be accepted at level (typically p> 0.05 or 0.10).
The correlation matrix of the variables in question is shown below. Remember that the correlation matrix is built on quantitative variables.

Correlations

		Anterior SAT distance	Posterior SAT distance	Anterior+Posterior SAT distance	VAT distance	FAT area (cm2)	SAT area (cm2)	VAT area (cm2)
Anterior SAT distance	Pearson Correlation	--						
	N	85						
Posterior SAT distance	Pearson Correlation	,515**	--					
	Sig. (2-tailed)	<,001						
	N	85	85					
Anterior+Posterior SAT distance	Pearson Correlation	,821**	,913**	--				
	Sig. (2-tailed)	<,001	<,001					
	N	85	85	85				
VAT distance	Pearson Correlation	,582**	,288**	,470**	--			
	Sig. (2-tailed)	<,001	,008	<,001				
	N	84	84	84	84			
FAT area (cm2)	Pearson Correlation	,804**	,637**	,808**	,634**	--		
	Sig. (2-tailed)	<,001	<,001	<,001	<,001			
	N	85	85	85	84	85		
SAT area (cm2)	Pearson Correlation	,826**	,669**	,840**	,497**	,947**	--	
	Sig. (2-tailed)	<,001	<,001	<,001	<,001	<,001		
	N	85	85	85	84	85	85	
VAT area (cm2)	Pearson Correlation	,586**	,431**	,567**	,709**	,850**	,638**	--
	Sig. (2-tailed)	<,001	<,001	<,001	<,001	<,001	<,001	
	N	84	84	84	84	84	84	84

**. Correlation is significant at the 0.01 level (2-tailed).

From the correlation matrix it can be observed that there is a positive and statistically significant relationship ($p < 0.05$) between the variable Anterior SAT distance and each of the variables taken into consideration: as one increases, the other increases and vice versa. The relationship with Anterior SAT distance is moderate as regards the Posterior SAT distance, VAT distance and VAT area (cm2) variables, while for the others it is of a strong type.

There is a positive and statistically significant relationship ($p < 0.05$) between the variable Posterior SAT distance and each of the other variables: as one increases, the other increases and vice versa.
The relationship with Posterior SAT distance is moderate as regards the Posterior SAT distance, FAT area (cm2), SAT area (cm2) and VAT area (cm2) variables; is low with VAT distance and is strong with Anterior+Posterior SAT distance.

There is a positive and statistically significant relationship ($p < 0.05$) between the variable Anterior+ Posterior SAT distance and each of the other variables: as one increases, the other increases and vice versa.
The relationship with Anterior+Posterior SAT distance is moderate as regards VAT distance and VAT area (cm2) variables, while for the others it is of a strong type.

There is a positive and statistically significant relationship ($p < 0.05$) between the variable VAT distance and each of the other variables: as one increases, the other increases and vice versa.
The relationship with VAT distance is strong as regards VAT area (cm2) variables, while for the others it is of a moderate type.

There is a positive, strong and statistically significant relationship ($p < 0.05$) between the variable FAT area (cm2) and each of the other variables: as one increases, the other increases and vice versa.

Finally, there is a positive, moderate and statistically significant relationship ($p < 0.05$) between SAT area (cm2) and VAT area (cm2).

T-tests with indipendent samples – BMI with fat measurements

Group Statistics

	BMI	N	Mean	Std. Deviation	Std. Error Mean
FAT mean density (HU)	<25	35	-82,02	10,48	1,77
	>=25	50	-87,52	8,45	1,20
FAT median density (HU)	<25	35	-84,74	12,29	2,08
	>=25	50	-90,92	9,48	1,34

FAT density standard deviation	<25	35	24,37	2,72	,46
	>=25	50	25,79	2,91	,41
SAT mean density (HU)	<25	35	-83,69	11,27	1,91
	>=25	50	-88,44	9,38	1,33
SAT median density (HU)	<25	34	-87,21	13,38	2,29
	>=25	49	-88,10	28,49	4,07
SAT density standard deviation	<25	35	24,01	3,12	,53
	>=25	49	25,37	3,09	,44
VAT mean density (HU)	<25	35	-79,54	10,39	1,76
	>=25	50	-85,02	8,90	1,26
VAT median density (HU)	<25	35	-81,29	12,96	2,19
	>=25	50	-84,58	27,01	3,82
VAT density standard deviation	<25	35	25,62	2,81	,48
	>=25	49	25,18	2,79	,40

Independent Samples Test

		Levene's Test for Equality of Variances		t-test for Equality of Means							
						Significance				95% Confidence Interval of the Difference	
		F	Sig.	T	df	One-Sided p	Two-Sided p	Mean Difference	Std. Error Difference	Lower	Upper
FAT mean density (HU)	Equal variances assumed	3,258	,075	2,676	83	,004	,009	5,51	2,06	1,41	9,60
	Equal variances not assumed			2,577	62,936	,006	,012	5,51	2,14	1,24	9,78
FAT median density (HU)	Equal variances assumed	3,554	,063	2,614	83	,005	,011	6,18	2,36	1,48	10,88
	Equal variances not assumed			2,499	60,924	,008	,015	6,18	2,47	1,23	11,12

FAT density standard deviation	Equal variances assumed	,079	,780	-2,287	83	,012	,025	-1,43	,63	-2,67	-,19
	Equal variances not assumed			-2,315	76,302	,012	,023	-1,43	,62	-2,66	-,20
SAT mean density (HU)	Equal variances assumed	2,480	,119	2,113	83	,019	,038	4,75	2,25	,28	9,22
	Equal variances not assumed			2,046	64,448	,022	,045	4,75	2,32	,11	9,39
SAT median density (HU)	Equal variances assumed	,136	,713	,171	81	,432	,865	,90	5,25	-9,55	11,35
	Equal variances not assumed			,192	72,683	,424	,848	,90	4,67	-8,42	10,21
SAT density standard deviation	Equal variances assumed	,238	,627	-1,978	82	,026	,051	-1,36	,69	-2,72	,01
	Equal variances not assumed			-1,975	72,883	,026	,052	-1,36	,70	-2,73	,01
VAT mean density (HU)	Equal variances assumed	2,567	,113	2,605	83	,005	,011	5,47	2,10	1,29	9,66
	Equal variances not assumed			2,534	65,858	,007	,014	5,47	2,16	1,16	9,79
VAT median density (HU)	Equal variances assumed	,153	,697	,669	83	,253	,505	3,29	4,93	-6,50	13,09
	Equal variances not assumed			,748	74,863	,228	,457	3,29	4,40	-5,48	12,07

VAT density standard deviation	Equal variances assumed	,321	,573	,713	82	,239	,478	,44	,62	-,79	1,68
	Equal variances not assumed			,712	73,138	,239	,479	,44	,62	-,80	1,68

In this case the Levene test is accepted in all cases ($p > 0.05$).

The test to be observed is, therefore, the classic one and the test rejects the equality of the means ($p < 0.05$) for the variables FAT mean density (HU), FAT median density (HU), FAT density standard deviation, SAT mean density (HU) and VAT mean density (HU).

The averages that are statistically higher in the BMI group <25 are those of the FAT mean density (HU), FAT median density (HU), SAT mean density (HU) and VAT mean density (HU) variables, while for the FAT density variable standard deviation (HU) appears to be higher in the BMI group> = 25.

For the other variables the t-test is rejected, so the means are not significantly different ($p > 0.05$).

Pearson’s correlations FAT area with fat measurements

Correlations

		FAT area (cm2)	FAT mean density (HU)	FAT median density (HU)	FAT density standard deviation	SAT mean density (HU)	SAT median density (HU)	SAT density standard deviation	VAT mean density (HU)	VAT median density (HU)	VAT density standard deviation
FAT area (cm2)	Pearson Correlation	--									
	N	85									
FAT mean density (HU)	Pearson Correlation	-,491**	--								
	Sig. (2-tailed)	<,001									
	N	85	85								
FAT median density (HU)	Pearson Correlation	-,481**	,991**	--							
	Sig. (2-tailed)	<,001	<,001								
	N	85	85	85							
FAT density standard deviation	Pearson Correlation	,372**	-,563**	-,518**	--						
	Sig. (2-tailed)	<,001	<,001	<,001							
	N	85	85	85	85						
SAT mean density (HU)	Pearson Correlation	-,426**	,947**	,940**	-,530**	--					
	Sig. (2-tailed)	<,001	<,001	<,001	<,001						
	N	85	85	85	85	85					
SAT median density (HU)	Pearson Correlation	-,162	,531**	,511**	-,313**	,591**	--				
	Sig. (2-tailed)	,144	<,001	<,001	,004	<,001					
	N	83	83	83	83	83	83				
SAT density standard deviation	Pearson Correlation	,283**	-,446**	-,409**	,845**	-,454**	-,160	--			
	Sig. (2-tailed)	,009	<,001	<,001	<,001	<,001	,150				
	N	84	84	84	84	84	82	84			
VAT mean	Pearson Correlation	-,453**	,852**	,845**	-,524**	,822**	,399**	-,553**	--		

density (HU)	Sig. (2-tailed)	<,001	<,001	<,001	<,001	<,001	<,001	<,001			
	N	85	85	85	85	85	83	84	85		
VAT median density (HU)	Pearson Correlation	-,009	,303**	,327**	-,138	,321**	,175	-,269*	,493**	--	
	Sig. (2-tailed)	,938	,005	,002	,209	,003	,113	,013	<,001		
	N	85	85	85	85	85	83	84	85	85	
VAT density standard deviation	Pearson Correlation	-,004	-,327**	-,283**	,605**	-,313**	-,223*	,609**	-,484**	-,263*	--
	Sig. (2-tailed)	,968	,002	,009	<,001	,004	,043	<,001	<,001	,016	
	N	84	84	84	84	84	83	83	84	84	84

**. Correlation is significant at the 0.01 level (2-tailed).

*. Correlation is significant at the 0.05 level (2-tailed).

From the correlation matrix it can be observed that there is a negative, statistically significant and moderate relationship ($p < 0.05$) between the variable FAT area (cm2) and the variables FAT mean density (HU), SAT mean density (HU) and VAT mean density (HU): as one decreases, the other increases and vice versa.

There is a positive and statistically significant ($p < 0.05$) relationship between the FAT area (cm2) variable and the FAT density standard deviation (moderate) and SAT density standard deviation (low) variables, so as one increases the other increases. The other relationships with FAT area (cm2) are not significant ($p > 0.05$), so they are linearly independent, i.e. it is as if the correlation were 0.

Pearson's correlations SAT area with fat measurements

Correlations

		SAT area (cm2)	FAT mean density (HU)	FAT median density (HU)	FAT density standard deviation	SAT mean density (HU)	SAT median density (HU)	SAT density standard deviation	VAT mean density (HU)	VAT median density (HU)	VAT density standard deviation
SAT area (cm2)	Pearson Correlation	--									
	N	85									
FAT mean density (HU)	Pearson Correlation	-,416**	--								
	Sig. (2-tailed)	<,001									
	N	85	85								
FAT median density (HU)	Pearson Correlation	-,414**	,991**	--							
	Sig. (2-tailed)	<,001	<,001								
	N	85	85	85							
FAT density standard deviation	Pearson Correlation	,296**	-,563**	-,518**	--						
	Sig. (2-tailed)	,006	<,001	<,001							
	N	85	85	85	85						
SAT mean density (HU)	Pearson Correlation	-,350**	,947**	,940**	-,530**	--					
	Sig. (2-tailed)	,001	<,001	<,001	<,001						
	N	85	85	85	85	85					
SAT median density (HU)	Pearson Correlation	-,145	,531**	,511**	-,313**	,591**	--				
	Sig. (2-tailed)	,192	<,001	<,001	,004	<,001					
	N	83	83	83	83	83	83				
SAT density standard deviation	Pearson Correlation	,152	-,446**	-,409**	,845**	-,454**	-,160	--			
	Sig. (2-tailed)	,168	<,001	<,001	<,001	<,001	,150				
	N	84	84	84	84	84	82	84			
VAT mean	Pearson Correlation	-,307**	,852**	,845**	-,524**	,822**	,399**	-,553**	--		

density (HU)	Sig. (2-tailed)	,004	<,001	<,001	<,001	<,001	<,001	<,001			
	N	85	85	85	85	85	83	84	85		
VAT median density (HU)	Pearson Correlation	,083	,303**	,327**	-,138	,321**	,175	-,269*	,493**	--	
	Sig. (2-tailed)	,452	,005	,002	,209	,003	,113	,013	<,001		
	N	85	85	85	85	85	83	84	85	85	
VAT density standard deviation	Pearson Correlation	-,040	-,327**	-,283**	,605**	-,313**	-,223*	,609**	-,484**	-,263*	--
	Sig. (2-tailed)	,715	,002	,009	<,001	,004	,043	<,001	<,001	,016	
	N	84	84	84	84	84	83	83	84	84	84

**. Correlation is significant at the 0.01 level (2-tailed).

*. Correlation is significant at the 0.05 level (2-tailed).

From the correlation matrix it can be observed that there is a negative, statistically significant and moderate relationship ($p < 0.05$) between the variable SAT area (cm2) and the variables FAT mean density (HU), FAT median density (HU), SAT mean density (HU) and VAT mean density (HU): as one decreases, the other increases and vice versa.

There is a positive, low and statistically significant ($p < 0.05$) relationship between the SAT area (cm2) variable and the FAT density standard deviation. The other relationships with SAT area (cm2) are not significant ($p > 0.05$), so they are linearly independent, i.e. it is as if the correlation were 0.

Pearson's correlations VAT area with fat measurements

Correlations

		VAT area (cm2)	FAT mean density (HU)	FAT median density (HU)	FAT density standard deviation	SAT mean density (HU)	SAT median density (HU)	SAT density standard deviation	VAT mean density (HU)	VAT median density (HU)	VAT density standard deviation
VAT area (cm2)	Pearson Correlation	--									
	N	84									
FAT mean density (HU)	Pearson Correlation	-,508**	--								
	Sig. (2-tailed)	<,001									
	N	84	85								
FAT median density (HU)	Pearson Correlation	-,488**	,991**	--							
	Sig. (2-tailed)	<,001	<,001								
	N	84	85	85							
FAT density standard deviation	Pearson Correlation	,416**	-,563**	-,518**	--						
	Sig. (2-tailed)	<,001	<,001	<,001							
	N	84	85	85	85						
SAT mean density (HU)	Pearson Correlation	-,454**	,947**	,940**	-,530**	--					
	Sig. (2-tailed)	<,001	<,001	<,001	<,001						
	N	84	85	85	85	85					
SAT median density (HU)	Pearson Correlation	-,151	,531**	,511**	-,313**	,591**	--				
	Sig. (2-tailed)	,175	<,001	<,001	,004	<,001					
	N	82	83	83	83	83	83				
SAT density standard deviation	Pearson Correlation	,421**	-,446**	-,409**	,845**	-,454**	-,160	--			
	Sig. (2-tailed)	<,001	<,001	<,001	<,001	<,001	,150				
	N	83	84	84	84	84	82	84			
VAT mean	Pearson Correlation	-,596**	,852**	,845**	-,524**	,822**	,399**	-,553**	--		

density (HU)	Sig. (2-tailed)	<,001	<,001	<,001	<,001	<,001	<,001	<,001			
	N	84	85	85	85	85	83	84	85		
VAT median density (HU)	Pearson Correlation	-,159	,303**	,327**	-,138	,321**	,175	-,269*	,493**	--	
	Sig. (2-tailed)	,149	,005	,002	,209	,003	,113	,013	<,001		
	N	84	85	85	85	85	83	84	85	85	
VAT density standard deviation	Pearson Correlation	,062	-,327**	-,283**	,605**	-,313**	-,223*	,609**	-,484**	-,263*	--
	Sig. (2-tailed)	,576	,002	,009	<,001	,004	,043	<,001	<,001	,016	
	N	83	84	84	84	84	83	83	84	84	84

**. Correlation is significant at the 0.01 level (2-tailed).

*. Correlation is significant at the 0.05 level (2-tailed).

From the correlation matrix it can be observed that there is a negative, statistically significant and moderate relationship ($p < 0.05$) between the variable VAT area (cm2) and the variables FAT mean density (HU), FAT median density (HU), SAT mean density (HU) and VAT mean density (HU): as one decreases, the other increases and vice versa.

There is a positive, moderate and statistically significant ($p < 0.05$) relationship between the VAT area (cm2) variable and the FAT density standard deviation and SAT density standard deviation. The other relationships with VAT area (cm2) are not significant ($p > 0.05$), so they are linearly independent, i.e. it is as if the correlation were 0.

ROC analysis

The ROC curve is used in the presence of:

- A dichotomous variable that represents the outcome of interest (e.g. healthy / sick patient)
- A Scale variable that represents the test variable with respect to which you want to search for an optimal cut-off above which (or below which) assign the patient to one of the two categories (healthy / sick patient) making the least possible error.

To evaluate the 'goodness' in terms of classification error we will use the 'classic' indices, namely: -

- Sensitivity, that is the proportion of true positives with respect to all positives;
- Specificity, i.e. the proportion of true negatives to all negatives.

Therefore, the ROC curve will allow us to identify the optimal threshold value (the so-called best cut-off), ie the threshold value of the test beyond which it is 'convenient' to predict a positive outcome, in order to maximize Sensitivity and Specificity (via Youden's index).

The ROC curve is constructed, therefore, considering all the possible values of the test (i.e. of the Scale variable) and, for each of them, the software calculates the Sensitivity and the proportion of false positives (i.e.: 1 - Specificity).

By joining the points that relate Sensitivity and 1-Specificity, a curve called the ROC curve is obtained.

The area below the ROC curve (AUC, acronym ofEnglish terms “Area Under the Curve”) is a measure of accuracy of the Scale variable chosen as a test.

The area under the curve can assume values between 0.5 and 1.0. The greater the area under the curve, the greater the ability of the test variable to correctly discriminate the two groups.

For the interpretation of the values of the area below the ROC curve it is possible to refer to the classification proposed by Swets:

1) AUC = 0.5 the test is not informative;

2) 0.5 <AUC≤0.7 the test is not very accurate;

3) 0.7 <AUC≤0.9 the test is moderately accurate;

4) 0.9 <AUC <1.0 the test is highly accurate;

5) AUC = 1 perfect test.

Furthermore, the hypothesis test is carried out which verifies the null hypothesis of AUC = 0.5 or of non-informative test variable (ie not capable of discriminating at all).

Therefore, if the p-value is <0.05, the null hypothesis of non-informative test will be rejected, thus concluding that the chosen Scale variable is accurate, or, at least, significantly better than the non-informative test situation.

Cut off SAT

To find a SAT cut-off able to predict the mortality variable, the sample was divided into Males and Females.

Case Processing Summary[b]

Gender	Mortality in 24-months follow-up	Valid N (listwise)
F	Positive[a]	16
	Negative	31
M	Positive[a]	12
	Negative	26

Larger values of the test result variable(s) indicate stronger evidence for a positive actual state.

a. The positive actual state is Yes.

b. For split file Gender = M, the test variable(s): SAT mean density (HU) has at least one tie between the positive actual state group and the negative actual state group.

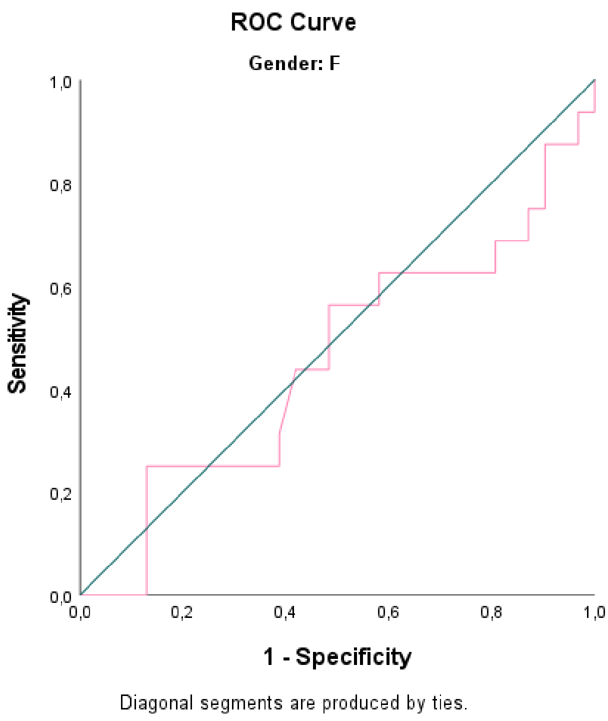

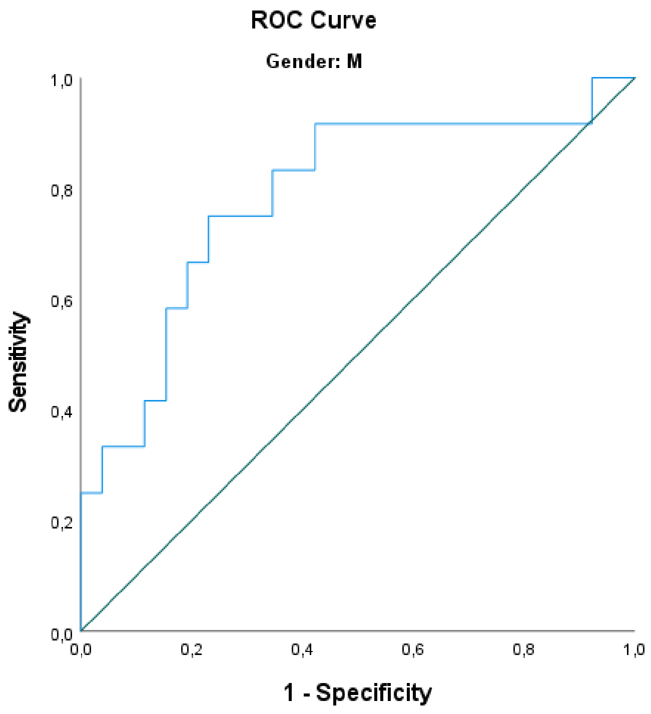

Area Under the Curve[a]

Test Result Variable(s): SAT mean density (HU)

Gender	Area
F	,456

M	,785

a. For split file Gender = F, the test result variable(s): SAT mean density (HU) has at least one tie between the positive actual state group and the negative actual state group. Statistics may be biased.

Area Under the Curve[a]

Test Result Variable(s): SAT mean density (HU)

Area	Std. Error[b]	Asymptotic Sig.[c]	Asymptotic 95% Confidence Interval	
			Lower Bound	Upper Bound
,456	,093	,621	,274	,638

The test result variable(s): SAT mean density (HU) has at least one tie between the positive actual state group and the negative actual state group. Statistics may be biased.

a. Gender = F

b. Under the nonparametric assumption

c. Null hypothesis: true area = 0.5

Youden Index F: 1.079; cut-off: -87.9079; sensibility: 0.563; specificity: 0.516

Area Under the Curve[a]

Test Result Variable(s): SAT mean density (HU)

Area	Std. Error[b]	Asymptotic Sig.[c]	Asymptotic 95% Confidence Interval	
			Lower Bound	Upper Bound
,785	,085	,005	,619	,951

a. Gender = M

b. Under the nonparametric assumption

c. Null hypothesis: true area = 0.5

Youden Index M: 1.519; cut-off: -88.8750; sensibility: 0.750; specificity: 0.769

For females, a significant result is not obtained ($p > 0.05$), therefore the SAT variable does not discriminate well between the two groups, while in males there is a significant result ($p < 0.05$) and the AUC is equal to 0.785, therefore the test is moderately accurate. The cut-off found indicates the value above which the 1's are expected.

Cut off VAT

To find a VAT cut-off able to predict the mortality variable, the sample was divided into Males and Females.

Case Processing Summary[b]

Gender	Mortality in 24-months follow-up	Valid N (listwise)
F	Positive[a]	16
	Negative	31
M	Positive[a]	12
	Negative	26

Larger values of the test result variable(s) indicate stronger evidence for a positive actual state.

a. The positive actual state is Yes.

b. For split file Gender = M, the test variable(s): VAT mean density (HU) has at least one tie between the positive actual state group and the negative actual state group.

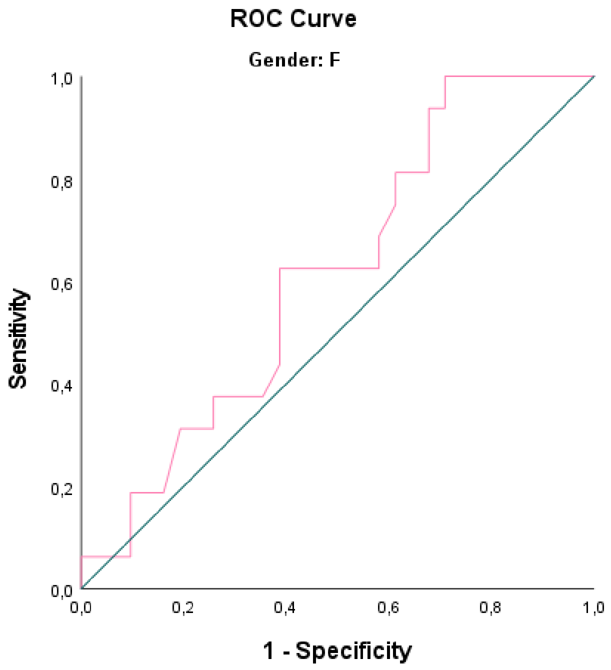

Diagonal segments are produced by ties.

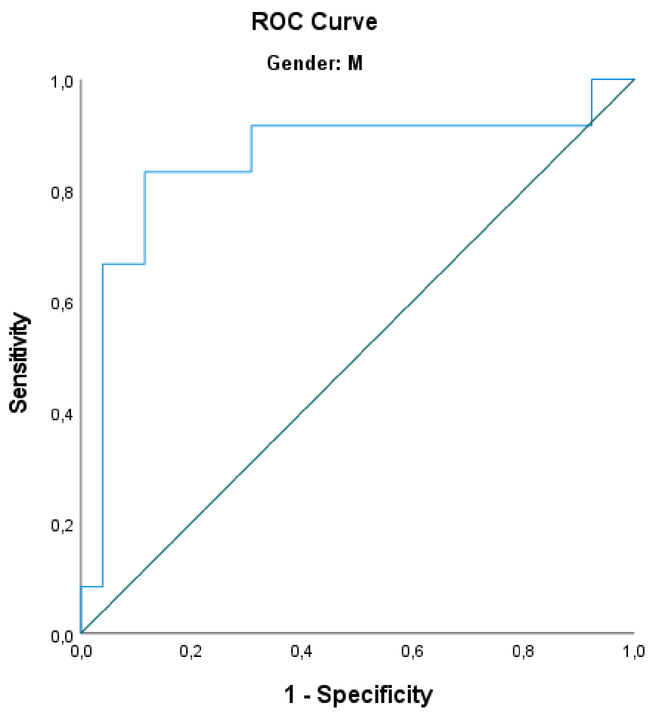

Area Under the Curve[a]

Test Result Variable(s): VAT mean density (HU)

Gender	Area
F	,613
M	,856

a. For split file Gender = F, the test result variable(s): VAT mean density (HU) has at least one tie between the positive actual state group and the negative actual state group. Statistics may be biased.

Area Under the Curve[a]

Test Result Variable(s): VAT mean density (HU)

Area	Std. Error[b]	Asymptotic Sig.[c]	Asymptotic 95% Confidence Interval	
			Lower Bound	Upper Bound
,613	,083	,209	,450	,775

The test result variable(s): VAT mean density (HU) has at least one tie between the positive actual state group and the negative actual state group. Statistics may be biased.

a. Gender = F

b. Under the nonparametric assumption

c. Null hypothesis: true area = 0.5

Youden Index F: 0.980; cut-off: -87.0310; sensibility: 0.625; specificity: 0.355

Area Under the Curve[a]

Test Result Variable(s): VAT mean density (HU)

Area	Std. Error[b]	Asymptotic Sig.[c]	Asymptotic 95% Confidence Interval	
			Lower Bound	Upper Bound
,856	,080	,000	,700	1,000

a. Gender = M

b. Under the nonparametric assumption

c. Null hypothesis: true area = 0.5

Youden Index M: 1.718; cut-off: -83.0905; sensibility: 0.833; specificity: 0.885

For females, a significant result is not obtained ($p > 0.05$), therefore the SAT variable does not discriminate well between the two groups, while in males there is a significant result ($p < 0.05$) and the AUC is equal to 0.856, therefore the test is moderately accurate. The cut-off found indicates the value above which the 1's are expected.

T-tests with indipendent samples – BMI with Right common femoral artery area and Left common femoral artery area

Group Statistics

	BMI	N	Mean	Std. Deviation	Std. Error Mean
Right common femoral artery area (mm2)	<25	35	57,15	19,68	3,33
	>=25	50	56,07	22,62	3,20
Left common femoral artery area (mm2)	<25	35	57,91	20,27	3,43
	>=25	50	59,11	20,31	2,87

Independent Samples Test

		Levene's Test for Equality of Variances		t-test for Equality of Means							
						Significance				95% Confidence Interval of the Difference	
		F	Sig.	T	df	One-Sided p	Two-Sided p	Mean Difference	Std. Error Difference	Lower	Upper
Right common femoral artery area (mm2)	Equal variances assumed	,664	,418	,228	83	,410	,820	1,08	4,73	-8,33	10,49
	Equal variances not assumed			,233	79,063	,408	,816	1,078	4,62	-8,108	10,26
Left common femoral artery area (mm2)	Equal variances assumed	,327	,569	-,269	83	,394	,789	-1,20	4,47	-10,10	7,69
	Equal variances not assumed			-,269	73,425	,394	,789	-1,20	4,47	-10,11	7,71

In this case the Levene test is accepted in both cases ($p > 0.05$).

The test to be observed is, therefore, the classic one and the test accepts the equality of the means ($p > 0.05$) in both cases, so there so statistically difference between the means in the two groups.

Pearson's correlations of Right common femoral artery area

Correlations

		Right common femoral artery area (mm2)	Anterior SAT distance	Posterior SAT distance	Anterior+Posterior SAT distance	VAT distance	FAT area (cm2)	SAT area (cm2)	VAT area (cm2)
Right common femoral artery area (mm2)	Pearson Correlation	--							
	N	85							
Anterior SAT distance	Pearson Correlation	-,146	--						
	Sig. (2-tailed)	,182							
	N	85	85						
Posterior SAT distance	Pearson Correlation	-,005	,515**	--					
	Sig. (2-tailed)	,962	<,001						
	N	85	85	85					
Anterior+Posterior SAT distance	Pearson Correlation	-,073	,821**	,913**	--				
	Sig. (2-tailed)	,506	<,001	<,001					
	N	85	85	85	85				
VAT distance	Pearson Correlation	,113	,582**	,288**	,470**	--			
	Sig. (2-tailed)	,306	<,001	,008	<,001				
	N	84	84	84	84	84			
FAT area (cm2)	Pearson Correlation	-,034	,804**	,637**	,808**	,634**	--		
	Sig. (2-tailed)	,756	<,001	<,001	<,001	<,001			
	N	85	85	85	85	84	85		
SAT area (cm2)	Pearson Correlation	-,072	,826**	,669**	,840**	,497**	,947**	--	
	Sig. (2-tailed)	,510	<,001	<,001	<,001	<,001	<,001		
	N	85	85	85	85	84	85	85	

VAT area (cm2)	Pearson Correlation	,040	,586**	,431**	,567**	,709**	,850**	,638**	--
	Sig. (2-tailed)	,719	<,001	<,001	<,001	<,001	<,001	<,001	
	N	84	84	84	84	84	84	84	84

**. Correlation is significant at the 0.01 level (2-tailed).

There are not statistically significant ($p>0.05$) relationships with Right common femoral artery area (mm2) and the other variables, so they are linearly independent, i.e. it is as if the correlation were 0.

Pearson’s correlations of Left common femoral artery area

Correlations

		Left common femoral artery area (mm2)	Anterior SAT distance	Posterior SAT distance	Anterior+Posterior SAT distance	VAT distance	FAT area (cm2)	SAT area (cm2)	VAT area (cm2)
Left common femoral artery area (mm2)	Pearson Correlation	--							
	N	85							
Anterior SAT distance	Pearson Correlation	-,091	--						
	Sig. (2-tailed)	,408							
	N	85	85						
Posterior SAT distance	Pearson Correlation	,066	,515**	--					
	Sig. (2-tailed)	,551	<,001						
	N	85	85	85					
Anterior+Posterior SAT distance	Pearson Correlation	,000	,821**	,913**	--				
	Sig. (2-tailed)	,998	<,001	<,001					
	N	85	85	85	85				
VAT distance	Pearson Correlation	,175	,582**	,288**	,470**	--			
	Sig. (2-tailed)	,111	<,001	,008	<,001				
	N	84	84	84	84	84			
FAT area (cm2)	Pearson Correlation	,044	,804**	,637**	,808**	,634**	--		
	Sig. (2-tailed)	,692	<,001	<,001	<,001	<,001			
	N	85	85	85	85	84	85		
SAT area (cm2)	Pearson Correlation	,014	,826**	,669**	,840**	,497**	,947**	--	
	Sig. (2-tailed)	,897	<,001	<,001	<,001	<,001	<,001		
	N	85	85	85	85	84	85	85	

VAT area (cm2)	Pearson Correlation	,088	,586**	,431**	,567**	,709**	,850**	,638**	--
	Sig. (2-tailed)	,423	<,001	<,001	<,001	<,001	<,001	<,001	
	N	84	84	84	84	84	84	84	84

**. Correlation is significant at the 0.01 level (2-tailed).

There are not statistically significant ($p>0.05$) relationships with Left common femoral artery area (mm2) and the other variables, so they are linearly independent, i.e. it is as if the correlation were 0.

PUNTO 4

Chi-Square and Cramer's V test BMI with Adverse cardiac and cerebrovascular events and mortality at follow-up

Contingency tables are constructed to assess whether or not there is a statistically significant association between two variables taken into consideration. The term association is used when we want to study the link between two qualitative variables (not to be confused with the correlation used for quantitative variables).

To test the association, the chi-squared test will then be used on each contingency table analyzed, which is based on the following set of hypotheses:

- Null hypothesis: independence of the variables under consideration;
- Alternative hypothesis: the two variables under consideration are not independent and there is a statistically significant association.

If the test is significant ($p < 0.05$), the null hypothesis of independence between the variables is rejected and it can be concluded that there is a statistically significant form of association between the variables under consideration. Otherwise if ($p > 0.05$) the null hypothesis of independence between the variables is accepted, concluding that there is no statistically significant association.

Crosstab

			Adverse cardiac events in 24-months follow-up		
			No	Yes	Total
BMI	<25	Count	17	18	35
		% within BMI	48,6%	51,4%	100,0%
	>=25	Count	44	6	50
		% within BMI	88,0%	12,0%	100,0%
Total		Count	61	24	85
		% within BMI	71,8%	28,2%	100,0%

It is noted that 17 patients do not have the cardiac event and have a BMI <25, 18 have had the cardiac event and have a BMI <25, 44 have not had the cardiac event and have a BMI> = 25 and 6 people have had the cardiac event and have a BMI> = 25.

Among those who have a BMI <25, 48.6% did not have the cardiac event, while 51.4% did; among those who have a BMI> = 25, 88% did not have the cardiac event, while 12% did.

Chi-Square Tests

	Value	df	Asymptotic Significance (2-sided)	Exact Sig. (2-sided)	Exact Sig. (1-sided)

Pearson Chi-Square	15,796[a]	1	<,001		
Continuity Correction[b]	13,910	1	<,001		
Likelihood Ratio	15,993	1	<,001		
Fisher's Exact Test				<,001	<,001
Linear-by-Linear Association	15,610	1	<,001		
N of Valid Cases	85				

a. 0 cells (0,0%) have expected count less than 5. The minimum expected count is 9,88.

b. Computed only for a 2x2 table

The chi-squared test is statistically significant ($p < 0.05$), therefore the null hypothesis of independence between the variables is rejected and it can be said that there is a statistically significant association between the variables.
Since the test is statistically significant, Cramer's V is interpreted. This value defines the "Strength of the association" and can be interpreted as follows:

- If the V is between 0 and 0.33, the association is of low intensity
- If the V is between 0.33 and 0.66, the association is of moderate intensity
- If the V is between 0.66 and 1, the association is of strong intensity.

Symmetric Measures

		Value	Approximate Significance
Nominal by Nominal	Phi	-,431	<,001
	Cramer's V	,431	<,001
N of Valid Cases		85	

In this case, Cramer's V, equal to 0.431, shows us that this association is of moderate intensity.

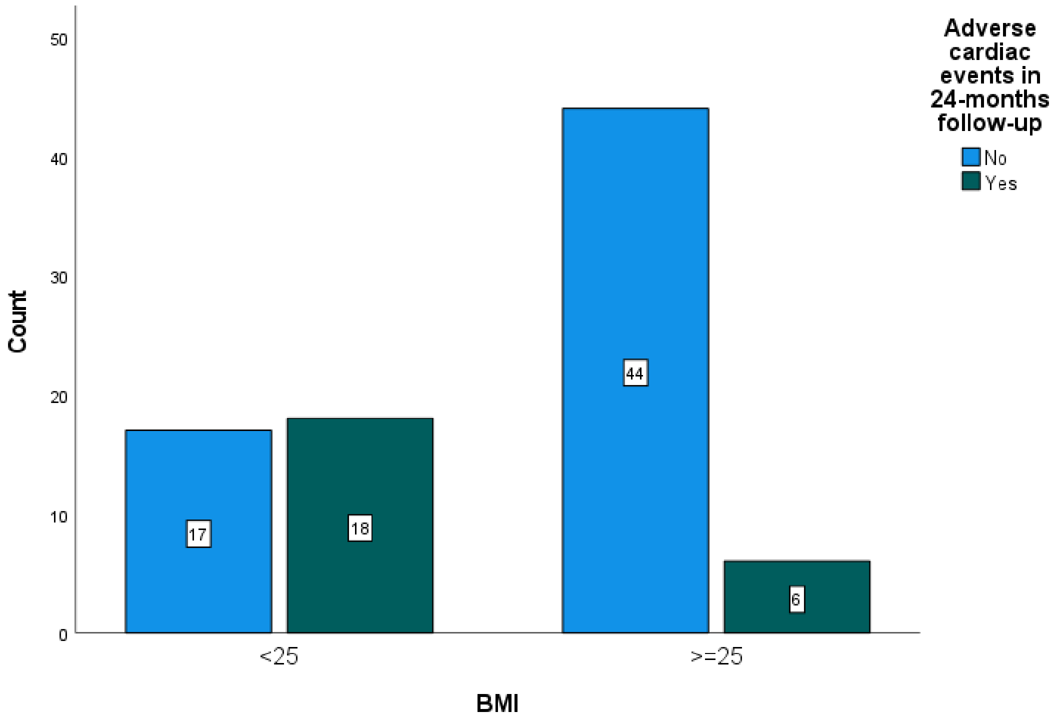
Adverse cardiac events in 24-months follow-up
No
Yes
Count
50
40
30
20
10
0
17
18
44
6
<25
>=25
BMI

Crosstab

			Cerebrovascular events in 24-months follow-up		
			No	Yes	Total
BMI	<25	Count	17	18	35
		% within BMI	48,6%	51,4%	100,0%
	>=25	Count	46	4	50
		% within BMI	92,0%	8,0%	100,0%
Total		Count	63	22	85
		% within BMI	74,1%	25,9%	100,0%

It is noted that 17 patients do not have the cerebrovascular event and have a BMI <25, 18 have had the cerebrovascular event and have a BMI <25, 46 have not had the cerebrovascular event and have a BMI> = 25 and 4 people have had the cerebrovascular event and have a BMI> = 25.

Among those who have a BMI <25, 48.6% did not have the cerebrovascular event, while 51.4% did; among those who have a BMI> = 25, 92% did not have the cerebrovascular event, while 8% did.

Chi-Square Tests

	Value	df	Asymptotic Significance (2-sided)	Exact Sig. (2-sided)	Exact Sig. (1-sided)
Pearson Chi-Square	20,242[a]	1	<,001		
Continuity Correction[b]	18,041	1	<,001		
Likelihood Ratio	20,841	1	<,001		
Fisher's Exact Test				<,001	<,001
Linear-by-Linear Association	20,003	1	<,001		
N of Valid Cases	85				

a. 0 cells (0,0%) have expected count less than 5. The minimum expected count is 9,06.

b. Computed only for a 2x2 table

The chi-squared test is statistically significant ($p <0.05$), therefore the null hypothesis of independence between the variables is rejected and it can be said that there is a statistically significant association between the variables.

Since the test is statistically significant, Cramer's V is interpreted.

Symmetric Measures

		Value	Approximate Significance
Nominal by Nominal	Phi	-,488	<,001

Cramer's V	,488	<,001
N of Valid Cases	85	

In this case, Cramer's V, equal to 0.488, shows us that this association is of moderate intensity.

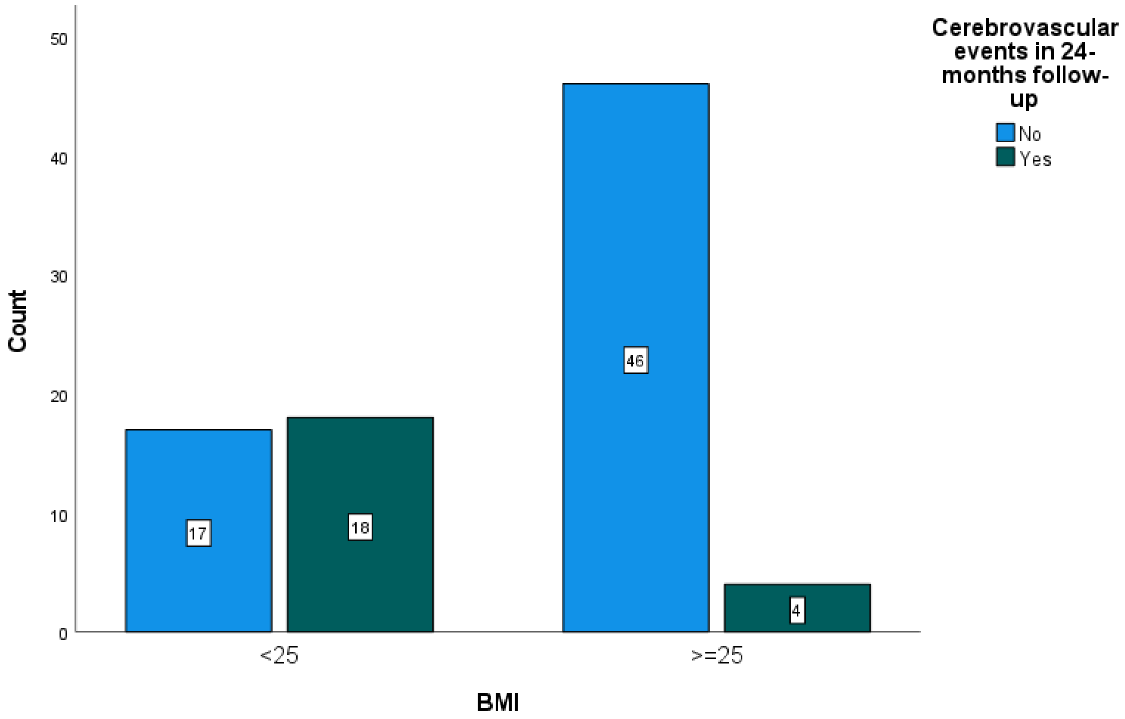

Crosstab

			Mortality in 24-months follow-up		
			No	Yes	Total
BMI	<25	Count	13	22	35
		% within BMI	37,1%	62,9%	100,0%
	>=25	Count	44	6	50
		% within BMI	88,0%	12,0%	100,0%
Total		Count	57	28	85
		% within BMI	67,1%	32,9%	100,0%

It is noted that 13 patients do not have the mortality event and have a BMI <25, 22 have had the cardiac event and have a BMI <25, 44 have not had the mortality event and have a BMI> = 25 and 6 people have had the mortality event and have a BMI> = 25.
Among those who have a BMI <25, 37.1% did not have the mortality event, while 62.9% did; among those who have a BMI> = 25, 88% did not have the mortality event, while 12% did.

Chi-Square Tests

	Value	df	Asymptotic Significance (2-sided)	Exact Sig. (2-sided)	Exact Sig. (1-sided)
Pearson Chi-Square	24,106[a]	1	<,001		
Continuity Correction[b]	21,859	1	<,001		
Likelihood Ratio	24,867	1	<,001		
Fisher's Exact Test				<,001	<,001
Linear-by-Linear Association	23,823	1	<,001		
N of Valid Cases	85				

a. 0 cells (0,0%) have expected count less than 5. The minimum expected count is 11,53.

b. Computed only for a 2x2 table

The chi-squared test is statistically significant ($p < 0.05$), therefore the null hypothesis of independence between the variables is rejected and it can be said that there is a statistically significant association between the variables.
Since the test is statistically significant, Cramer's V is interpreted.

Symmetric Measures

		Value	Approximate Significance
Nominal by Nominal	Phi	-,533	<,001
	Cramer's V	,533	<,001
N of Valid Cases		85	

In this case, Cramer's V, equal to 0.533, shows us that this association is of moderate intensity.

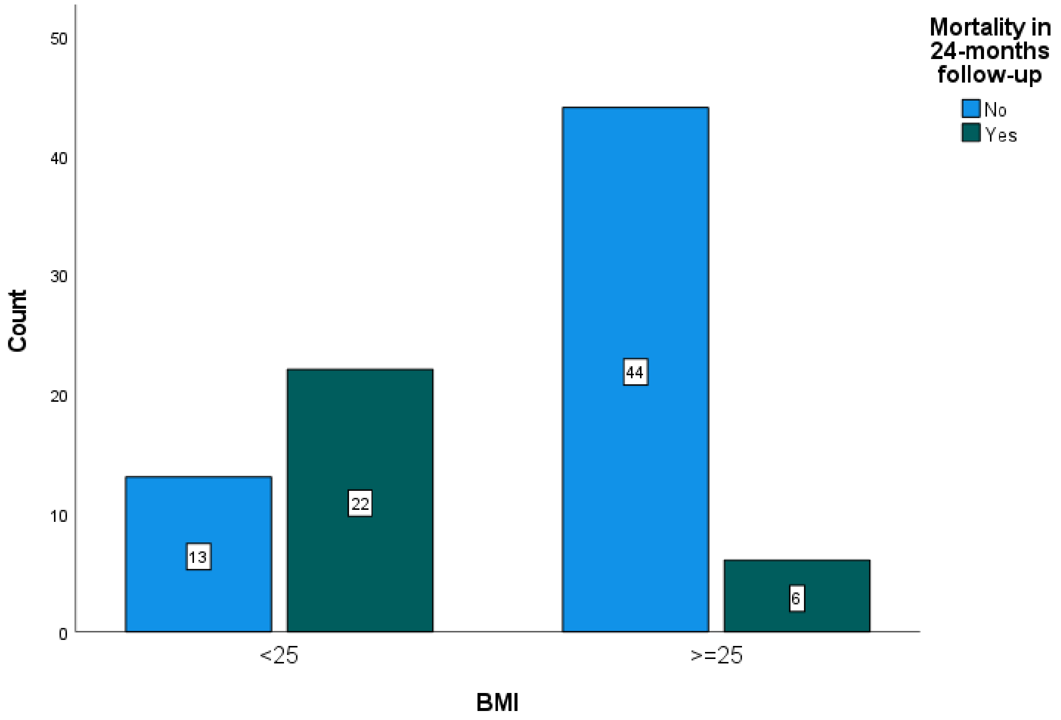

Independent-Samples Mann-Whitney U Test for Adverse cardiac events, cerebrovascular events and mortality in 24-months follow-up

A Mann-Whitney U is performed to verify if there is a statistically significant difference in the median of the numerical variable in the two subgroups of the dichotomous variable.

The null hypothesis of the test is that the medians are the same in the two groups while the alternative hypothesis is that the medians differ significantly in population.

The null hypothesis of equal medians is rejected, concluding that the medians are significantly different, if the p-value is less than 0.05. (it is the same as the t-test with independent samples, but it is used when at least one of the two groups has a small number, generally less than 30 units. The Levene test in this case is not necessary).

Adverse cardiac events in 24-months follow-up with other variables

Nonparametric Tests

Hypothesis Test Summary

	Null Hypothesis	Test	Sig.[a,b]	Decision
1	The distribution of Psoas/height is the same across categories of Adverse cardiac events in 24-months follow-up.	Independent-Samples Mann-Whitney U Test	<,001	Reject the null hypothesis.
2	The distribution of Anterior SAT distance is the same across categories of Adverse cardiac events in 24-months follow-up.	Independent-Samples Mann-Whitney U Test	<,001	Reject the null hypothesis.
3	The distribution of Posterior SAT distance is the same across categories of Adverse cardiac events in 24-months follow-up.	Independent-Samples Mann-Whitney U Test	<,001	Reject the null hypothesis.
4	The distribution of Anterior+Posterior SAT distance is the same across categories of Adverse cardiac events in 24-months follow-up.	Independent-Samples Mann-Whitney U Test	<,001	Reject the null hypothesis.

5	The distribution of VAT distance is the same across categories of Adverse cardiac events in 24-months follow-up.	Independent-Samples Mann-Whitney U Test	,074	Retain the null hypothesis.
6	The distribution of Right common femoral artery area (mm2) is the same across categories of Adverse cardiac events in 24-months follow-up.	Independent-Samples Mann-Whitney U Test	,423	Retain the null hypothesis.
7	The distribution of Left common femoral artery area (mm2) is the same across categories of Adverse cardiac events in 24-months follow-up.	Independent-Samples Mann-Whitney U Test	,278	Retain the null hypothesis.
8	The distribution of FAT area (cm2) is the same across categories of Adverse cardiac events in 24-months follow-up.	Independent-Samples Mann-Whitney U Test	,028	Reject the null hypothesis.
9	The distribution of SAT area (cm2) is the same across categories of Adverse cardiac events in 24-months follow-up.	Independent-Samples Mann-Whitney U Test	,004	Reject the null hypothesis.
10	The distribution of VAT area (cm2) is the same across categories of Adverse cardiac events in 24-months follow-up.	Independent-Samples Mann-Whitney U Test	,191	Retain the null hypothesis.
11	The distribution of Right Psoas muscle area (cm2) is the same across categories of Adverse cardiac events in 24-months follow-up.	Independent-Samples Mann-Whitney U Test	,015	Reject the null hypothesis.
12	The distribution of Left Psoas muscle area (cm2) is the same across categories of Adverse cardiac events in 24-months follow-up.	Independent-Samples Mann-Whitney U Test	,049	Reject the null hypothesis.
13	The distribution of FAT mean density (HU) is the same across categories of Adverse cardiac events in 24-months follow-up.	Independent-Samples Mann-Whitney U Test	,449	Retain the null hypothesis.

14	The distribution of FAT median density (HU) is the same across categories of Adverse cardiac events in 24-months follow-up.	Independent-Samples Mann-Whitney U Test	,356	Retain the null hypothesis.
15	The distribution of FAT density standard deviation is the same across categories of Adverse cardiac events in 24-months follow-up.	Independent-Samples Mann-Whitney U Test	,981	Retain the null hypothesis.
16	The distribution of SAT mean density (HU) is the same across categories of Adverse cardiac events in 24-months follow-up.	Independent-Samples Mann-Whitney U Test	,125	Retain the null hypothesis.
17	The distribution of SAT median density (HU) is the same across categories of Adverse cardiac events in 24-months follow-up.	Independent-Samples Mann-Whitney U Test	,387	Retain the null hypothesis.
18	The distribution of SAT density standard deviation is the same across categories of Adverse cardiac events in 24-months follow-up.	Independent-Samples Mann-Whitney U Test	,578	Retain the null hypothesis.
19	The distribution of VAT mean density (HU) is the same across categories of Adverse cardiac events in 24-months follow-up.	Independent-Samples Mann-Whitney U Test	,500	Retain the null hypothesis.
20	The distribution of VAT median density (HU) is the same across categories of Adverse cardiac events in 24-months follow-up.	Independent-Samples Mann-Whitney U Test	,491	Retain the null hypothesis.
21	The distribution of VAT density standard deviation is the same across categories of Adverse cardiac events in 24-months follow-up.	Independent-Samples Mann-Whitney U Test	,434	Retain the null hypothesis.
22	The distribution of Right Psoas muscle mean density (HU) is the same across categories of Adverse cardiac events in 24-months follow-up.	Independent-Samples Mann-Whitney U Test	,364	Retain the null hypothesis.

23	The distribution of Right Psoas muscle median density (HU) is the same across categories of Adverse cardiac events in 24-months follow-up.	Independent-Samples Mann-Whitney U Test	,467	Retain the null hypothesis.
24	The distribution of Right Psoas muscle density standard deviation is the same across categories of Adverse cardiac events in 24-months follow-up.	Independent-Samples Mann-Whitney U Test	,303	Retain the null hypothesis.
25	The distribution of Left Psoas muscle mean density (HU) is the same across categories of Adverse cardiac events in 24-months follow-up.	Independent-Samples Mann-Whitney U Test	,235	Retain the null hypothesis.
26	The distribution of Left Psoas muscle median density (HU) is the same across categories of Adverse cardiac events in 24-months follow-up.	Independent-Samples Mann-Whitney U Test	,206	Retain the null hypothesis.
27	The distribution of Left Psoas muscle density standard deviation is the same across categories of Adverse cardiac events in 24-months follow-up.	Independent-Samples Mann-Whitney U Test	,354	Retain the null hypothesis.

a. The significance level is ,050.

b. Asymptotic significance is displayed.

In this case, the hypothesis of equal medians ($p < 0.05$) is rejected for the variables Psoas/height, Anterior SAT distance, Posterior SAT distance, Anterior + Posterior SAT distance, FAT area (cm2), SAT area (cm2), Right Psoas muscle area (cm2) and Left Psoas muscle area (cm2), while for the rest we accept the null hypothesis ($p > 0.05$).

Psoas/height across Adverse cardiac events in 24-months follow-up

Independent-Samples Mann-Whitney U Test Summary

Total N	85
Mann-Whitney U	355,000
Wilcoxon W	655,000
Test Statistic	355,000
Standard Error	102,430
Standardized Test Statistic	-3,681
Asymptotic Sig.(2-sided test)	<,001

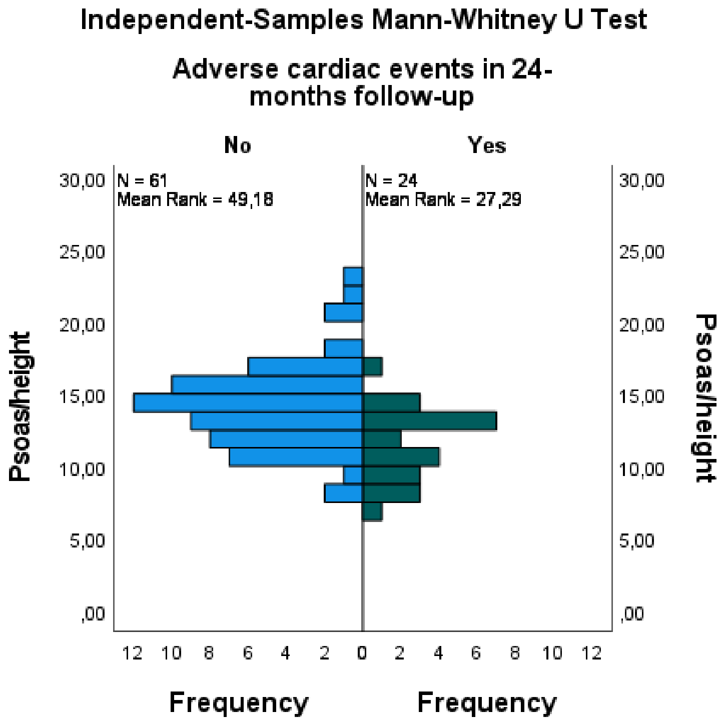

Anterior SAT distance across Adverse cardiac events in 24-months follow-up

Independent-Samples Mann-Whitney U Test Summary

Total N	85
Mann-Whitney U	392,000
Wilcoxon W	692,000
Test Statistic	392,000
Standard Error	102,416
Standardized Test Statistic	-3,320
Asymptotic Sig.(2-sided test)	<,001

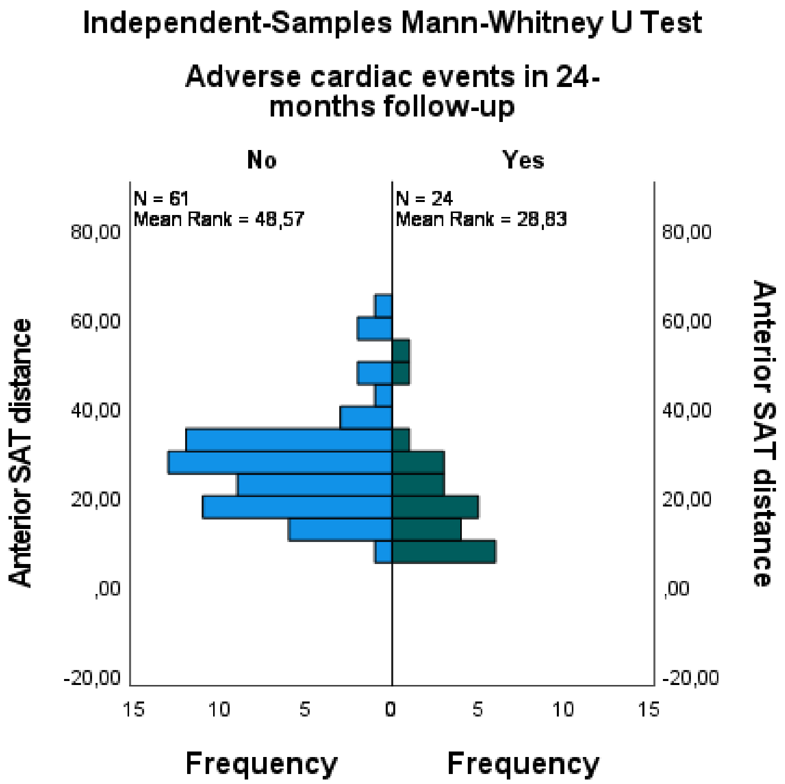

Posterior SAT distance across Adverse cardiac events in 24-months follow-up

Independent-Samples Mann-Whitney U Test Summary

Total N	85
Mann-Whitney U	350,500
Wilcoxon W	650,500
Test Statistic	350,500
Standard Error	102,419
Standardized Test Statistic	-3,725
Asymptotic Sig.(2-sided test)	<,001

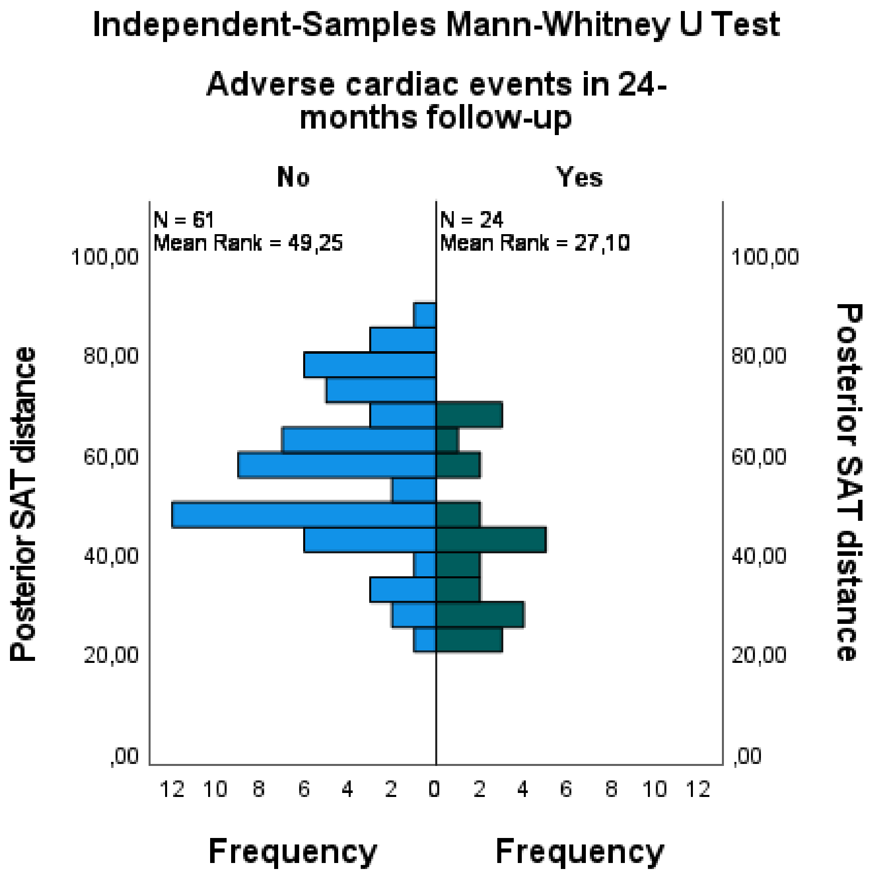

Anterior+Posterior SAT distance across Adverse cardiac events in 24-months follow-up

Independent-Samples Mann-Whitney U Test Summary

Total N	85
Mann-Whitney U	352,500
Wilcoxon W	652,500
Test Statistic	352,500
Standard Error	102,426
Standardized Test Statistic	-3,705
Asymptotic Sig.(2-sided test)	<,001

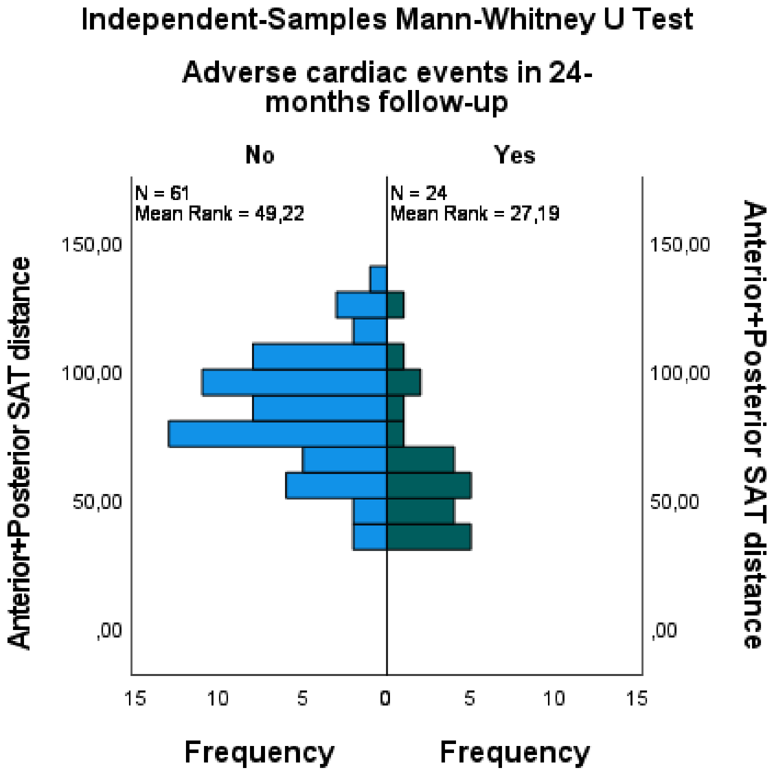

VAT distance across Adverse cardiac events in 24-months follow-up

Independent-Samples Mann-Whitney U Test Summary

Total N	84
Mann-Whitney U	523,500
Wilcoxon W	799,500
Test Statistic	523,500
Standard Error	99,669
Standardized Test Statistic	-1,786
Asymptotic Sig.(2-sided test)	,074

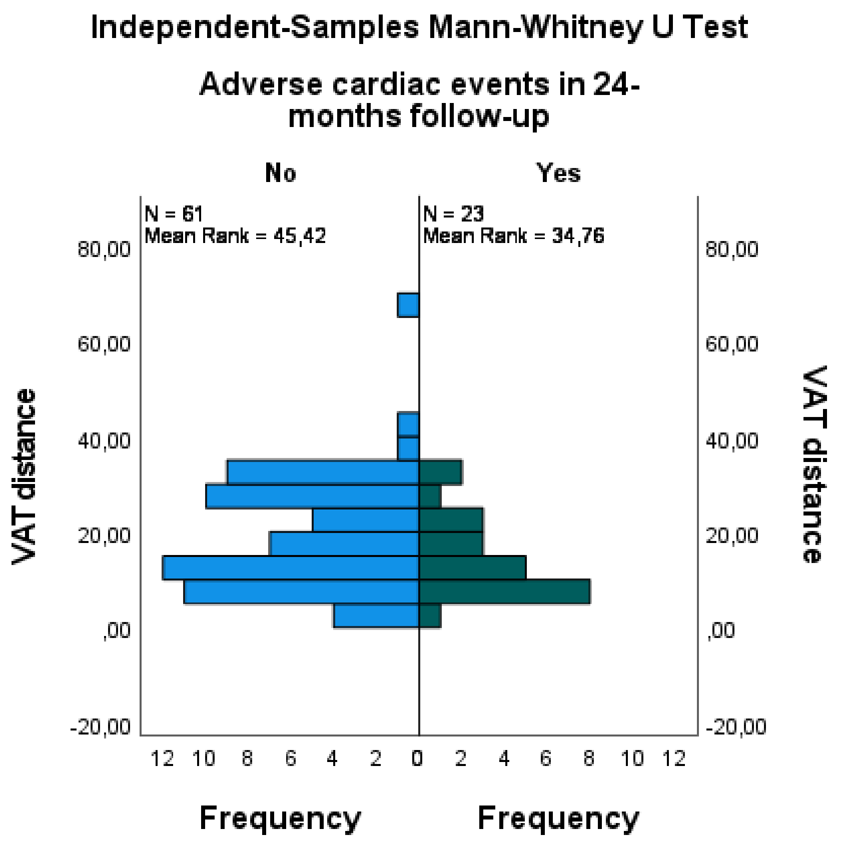

Right common femoral artery area (mm2) across Adverse cardiac events in 24-months follow-up

Independent-Samples Mann-Whitney U Test Summary

Total N	85
Mann-Whitney U	814,000
Wilcoxon W	1114,000
Test Statistic	814,000
Standard Error	102,413
Standardized Test Statistic	,801
Asymptotic Sig.(2-sided test)	,423

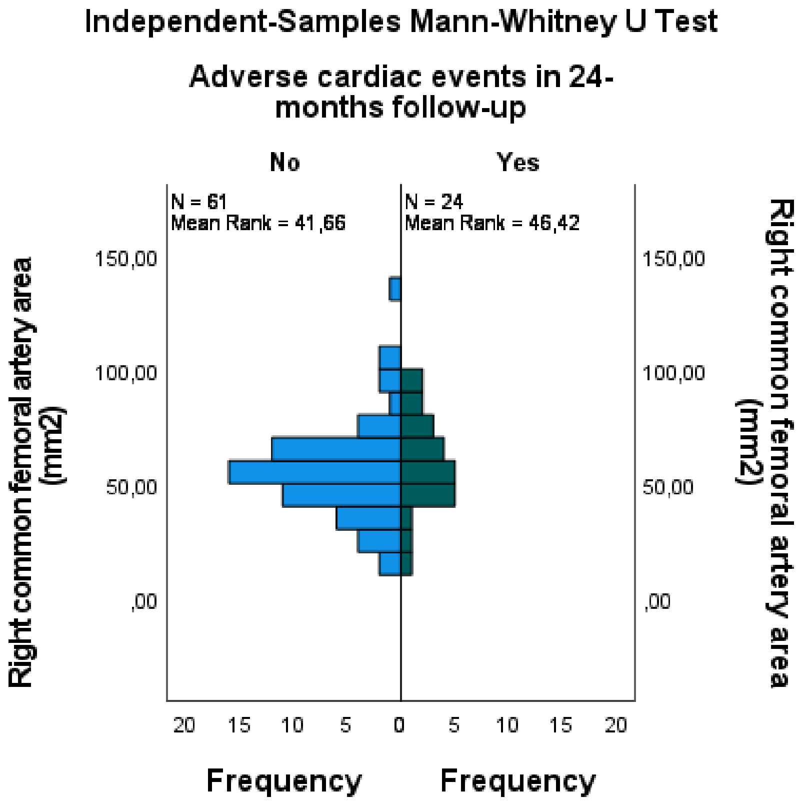

Left common femoral artery area (mm2) across Adverse cardiac events in 24-months follow-up

Independent-Samples Mann-Whitney U Test Summary

Total N	85
Mann-Whitney U	843,000
Wilcoxon W	1143,000
Test Statistic	843,000
Standard Error	102,405
Standardized Test Statistic	1,084
Asymptotic Sig.(2-sided test)	,278

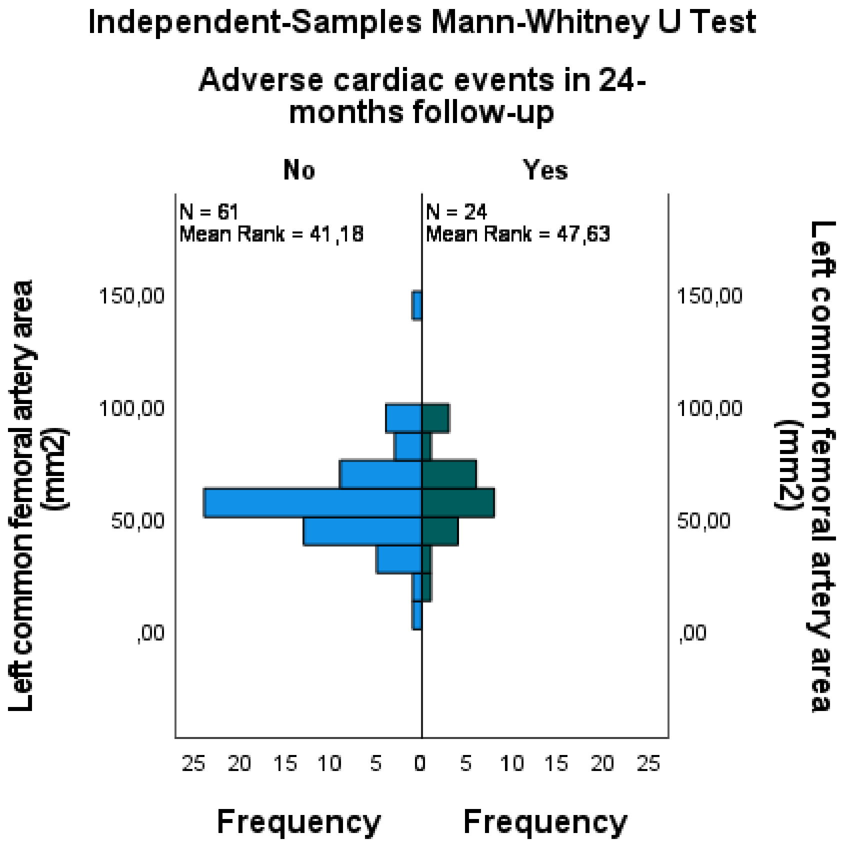

FAT area (cm2) across Adverse cardiac events in 24-months follow-up

Independent-Samples Mann-Whitney U Test Summary

Total N	85
Mann-Whitney U	507,000
Wilcoxon W	807,000
Test Statistic	507,000
Standard Error	102,430
Standardized Test Statistic	-2,197
Asymptotic Sig.(2-sided test)	,028

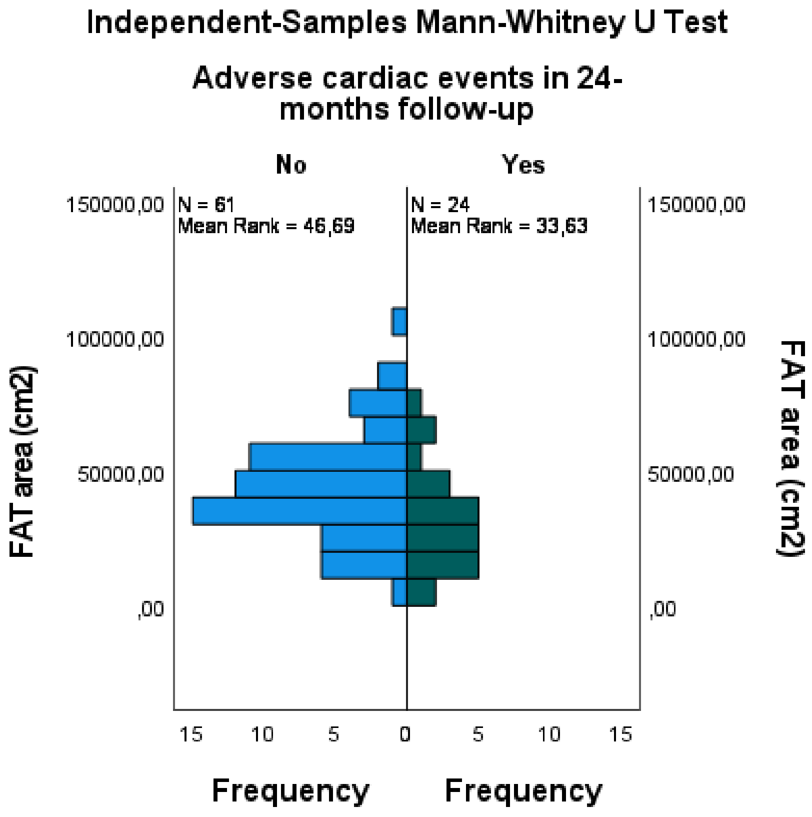

SAT area (cm2) across Adverse cardiac events in 24-months follow-up

Independent-Samples Mann-Whitney U Test Summary

Total N	85
Mann-Whitney U	440,000
Wilcoxon W	740,000
Test Statistic	440,000
Standard Error	102,430
Standardized Test Statistic	-2,851
Asymptotic Sig.(2-sided test)	,004

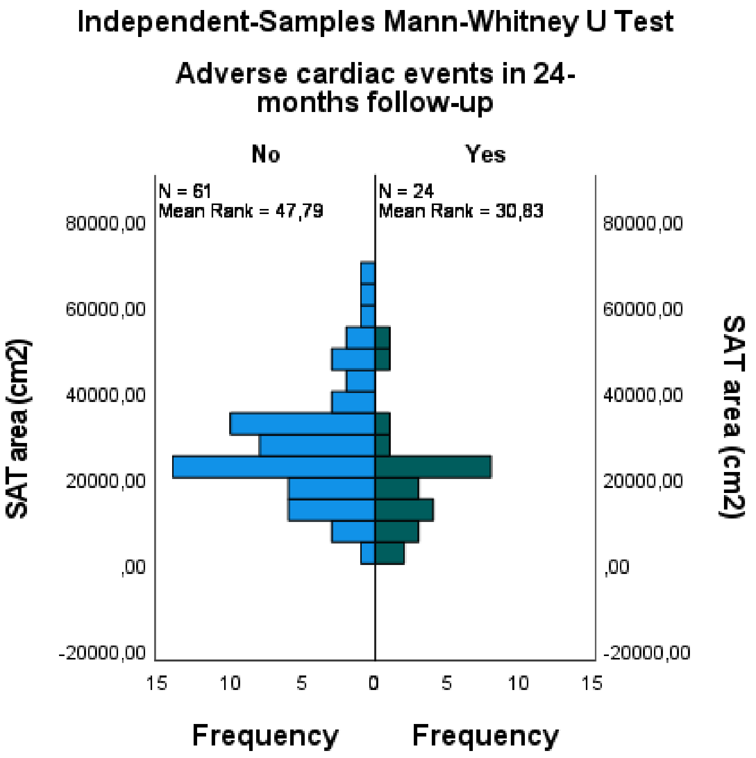

VAT area (cm2) across Adverse cardiac events in 24-months follow-up

Independent-Samples Mann-Whitney U Test Summary

Total N	84
Mann-Whitney U	571,000
Wilcoxon W	847,000
Test Statistic	571,000
Standard Error	99,689
Standardized Test Statistic	-1,309
Asymptotic Sig.(2-sided test)	,191

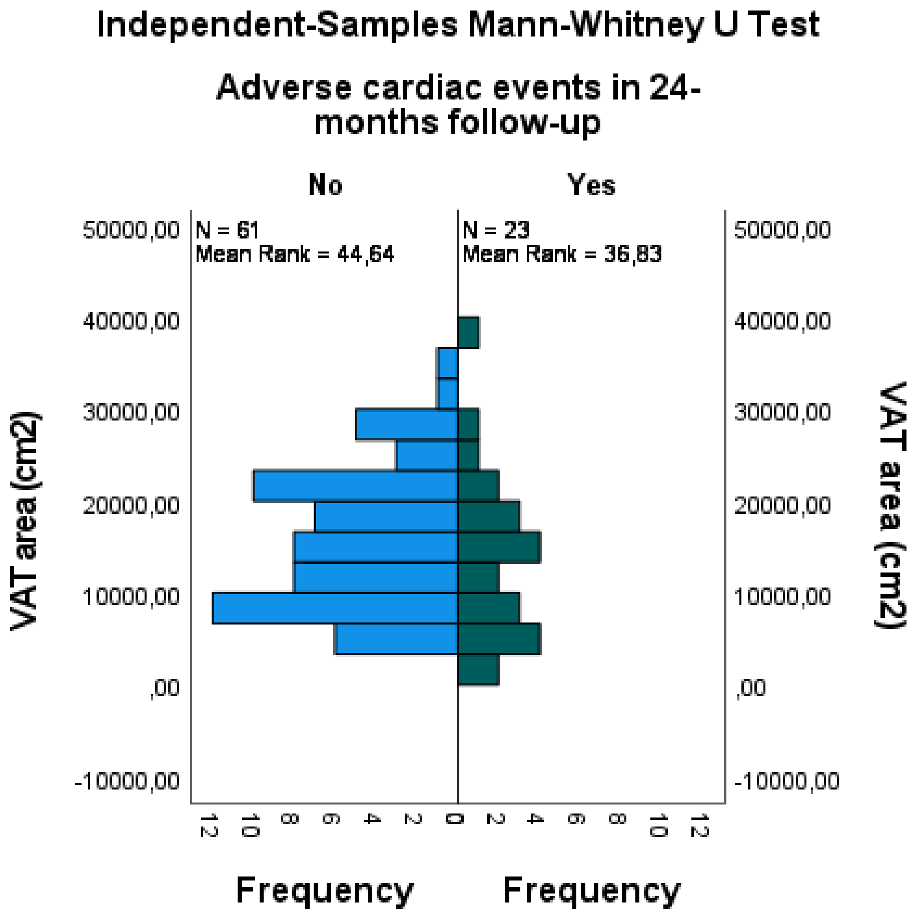

Right Psoas muscle area (cm2) across Adverse cardiac events in 24-months follow-up

Independent-Samples Mann-Whitney U Test Summary

Total N	85
Mann-Whitney U	483,000
Wilcoxon W	783,000
Test Statistic	483,000
Standard Error	102,427
Standardized Test Statistic	-2,431
Asymptotic Sig.(2-sided test)	,015

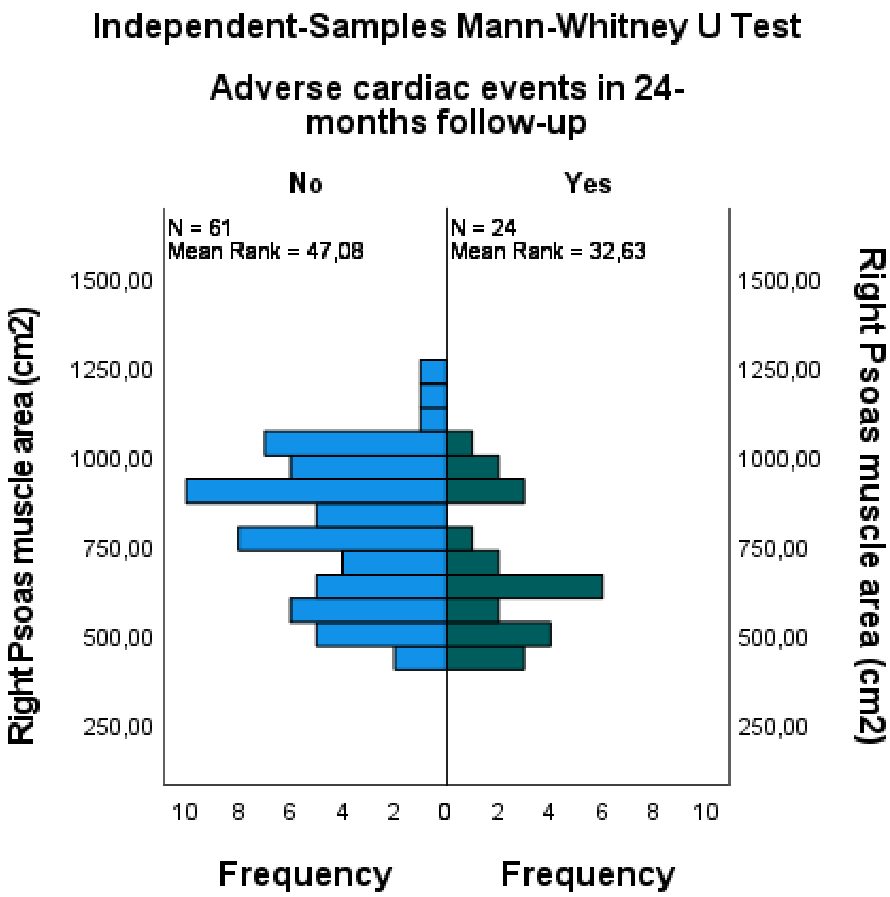

Left Psoas muscle area (cm2) across Adverse cardiac events in 24-months follow-up

Independent-Samples Mann-Whitney U Test Summary

Total N	85
Mann-Whitney U	531,000
Wilcoxon W	831,000
Test Statistic	531,000
Standard Error	102,429
Standardized Test Statistic	-1,962

Asymptotic Sig.(2-sided test)	,049

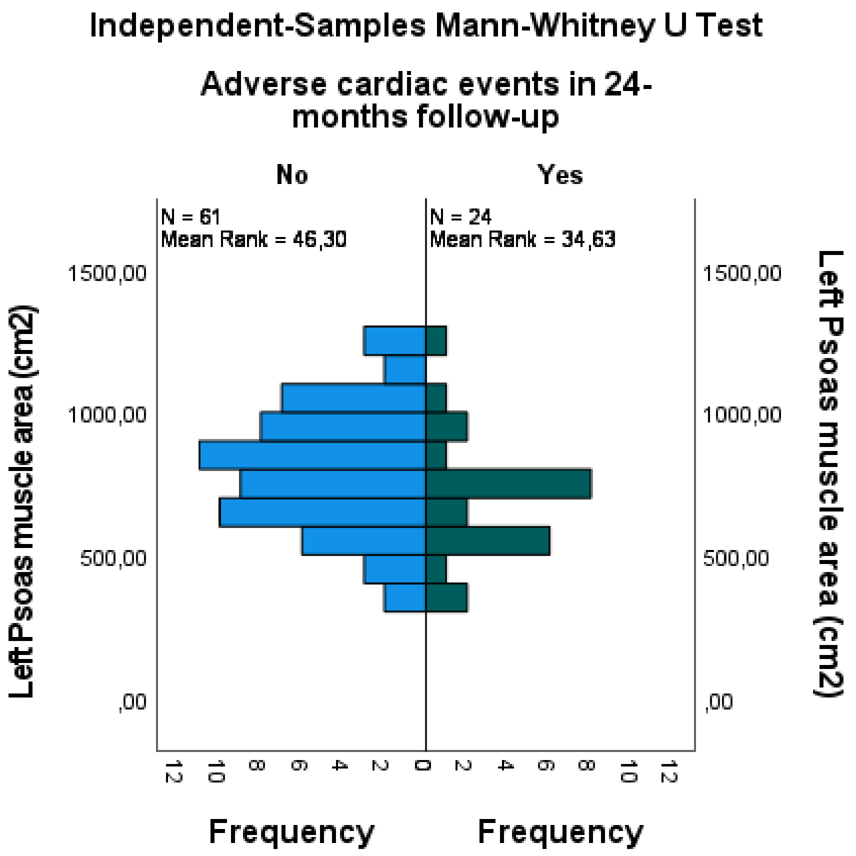

FAT mean density (HU) across Adverse cardiac events in 24-months follow-up

Independent-Samples Mann-Whitney U Test Summary	
Total N	85
Mann-Whitney U	809,500
Wilcoxon W	1109,500

Test Statistic	809,500
Standard Error	102,413
Standardized Test Statistic	,757
Asymptotic Sig.(2-sided test)	,449

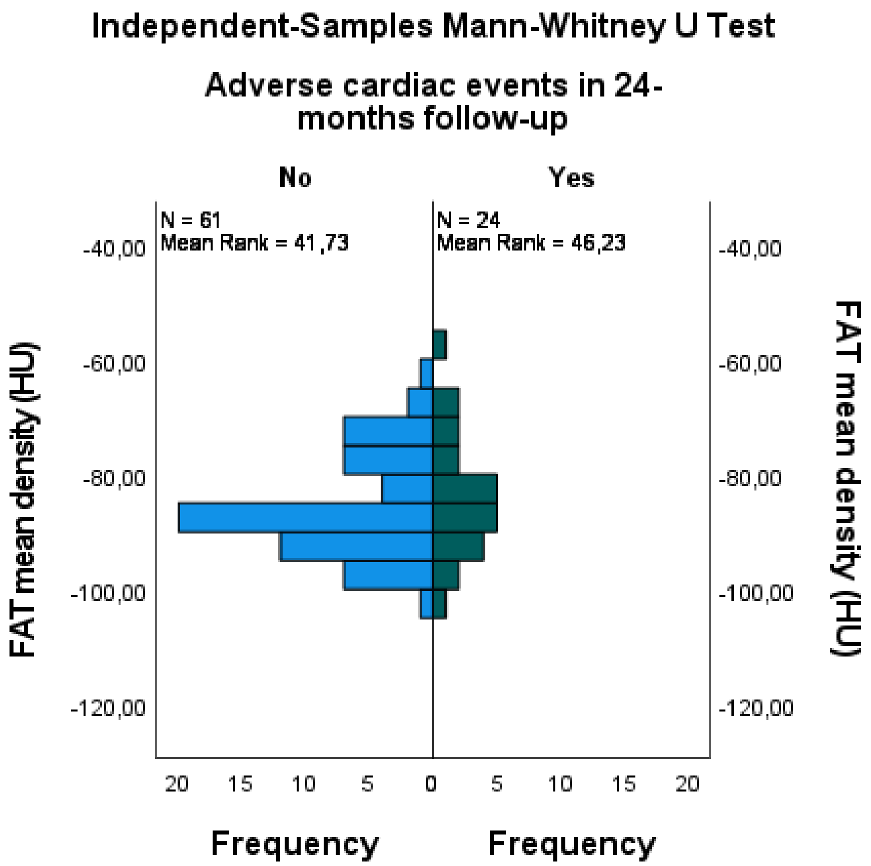

FAT median density (HU) across Adverse cardiac events in 24-months follow-up

Independent-Samples Mann-Whitney U Test Summary

Total N	85
Mann-Whitney U	826,500
Wilcoxon W	1126,500

Test Statistic	826,500
Standard Error	102,307
Standardized Test Statistic	,924
Asymptotic Sig.(2-sided test)	,356

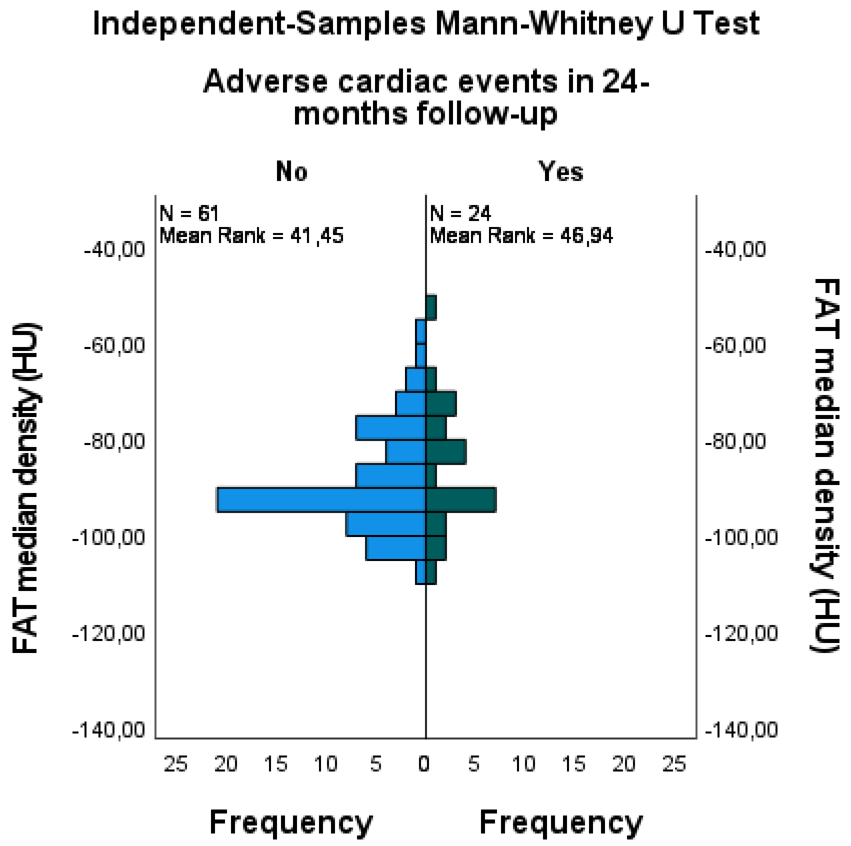

FAT density standard deviation across Adverse cardiac events in 24-months follow-up

Independent-Samples Mann-Whitney U Test Summary

Total N	85

Mann-Whitney U	734,500
Wilcoxon W	1034,500
Test Statistic	734,500
Standard Error	102,430
Standardized Test Statistic	,024
Asymptotic Sig.(2-sided test)	,981

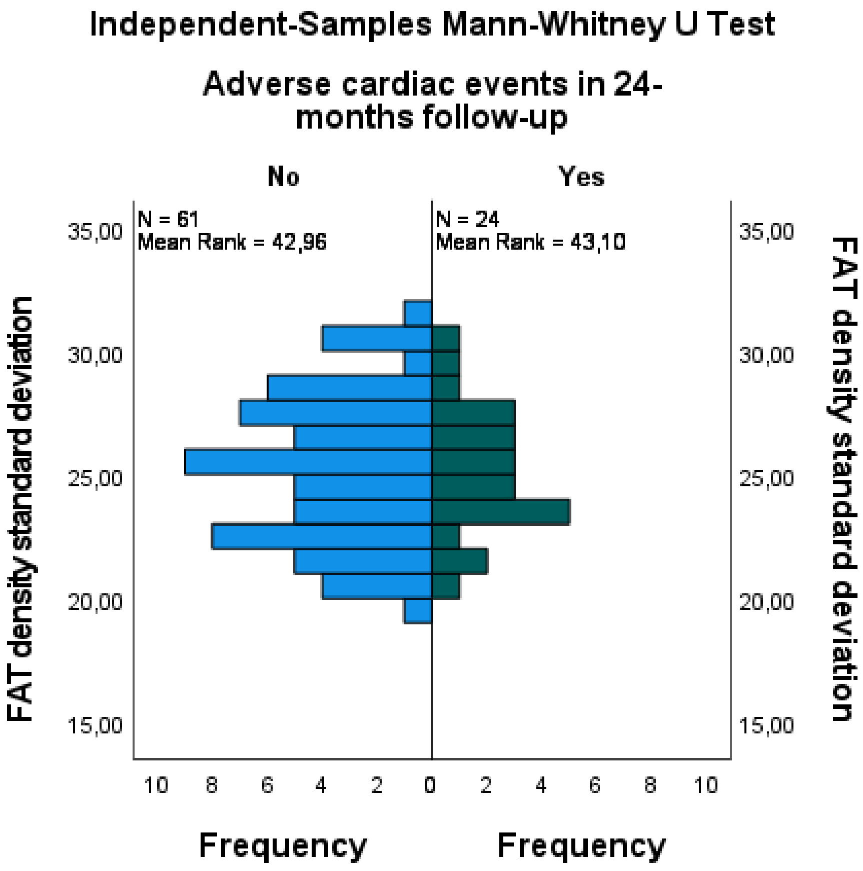

SAT mean density (HU) across Adverse cardiac events in 24-months follow-up

Independent-Samples Mann-Whitney U Test Summary

Total N	85
Mann-Whitney U	889,000
Wilcoxon W	1189,000
Test Statistic	889,000
Standard Error	102,418
Standardized Test Statistic	1,533
Asymptotic Sig.(2-sided test)	,125

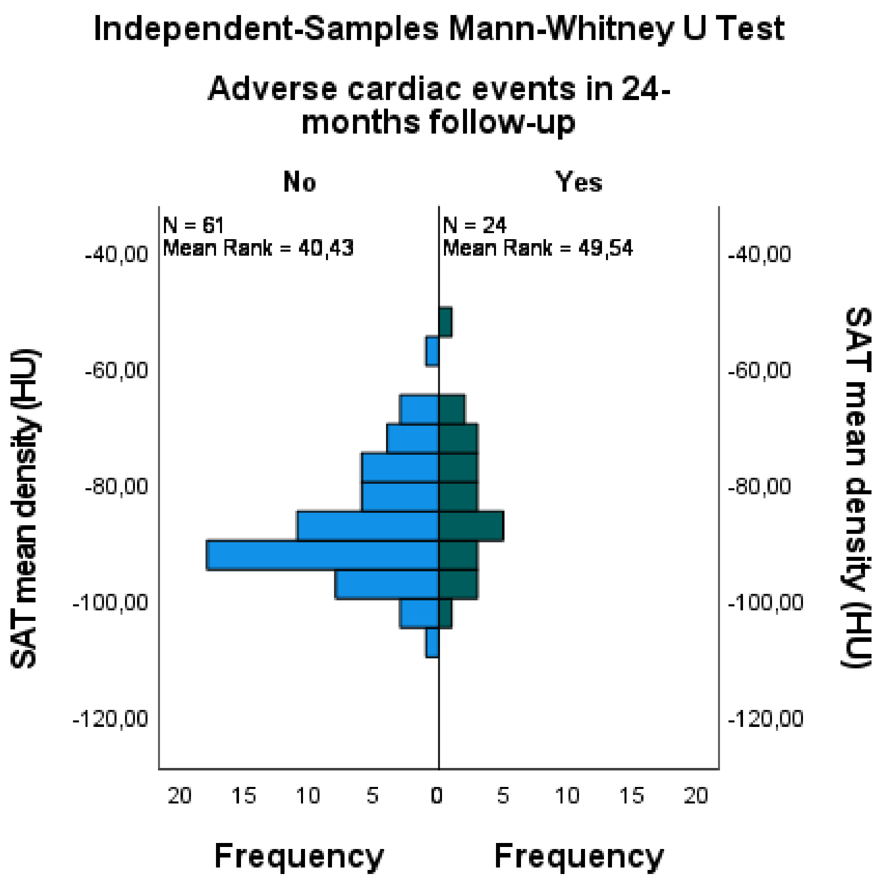

SAT median density (HU) across Adverse cardiac events in 24-months follow-up

Independent-Samples Mann-Whitney U Test Summary

Total N	83
Mann-Whitney U	775,000
Wilcoxon W	1051,000
Test Statistic	775,000
Standard Error	98,209
Standardized Test Statistic	,866
Asymptotic Sig.(2-sided test)	,387

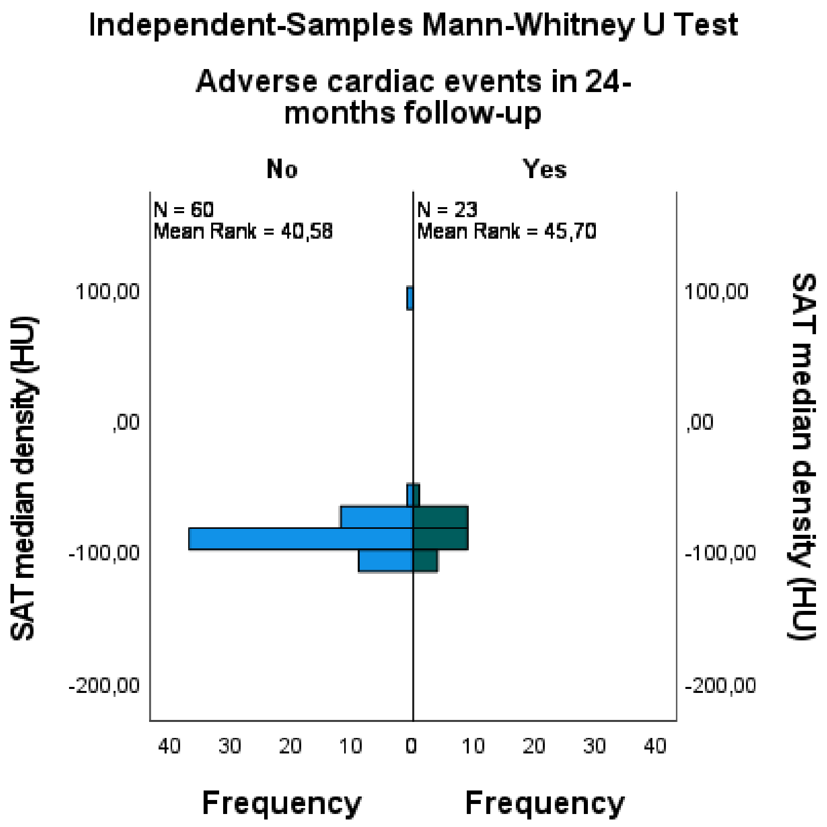

SAT density standard deviation across Adverse cardiac events in 24-months follow-up

Independent-Samples Mann-Whitney U Test Summary

Total N	84
Mann-Whitney U	646,000
Wilcoxon W	922,000
Test Statistic	646,000
Standard Error	99,689
Standardized Test Statistic	-,557
Asymptotic Sig.(2-sided test)	,578

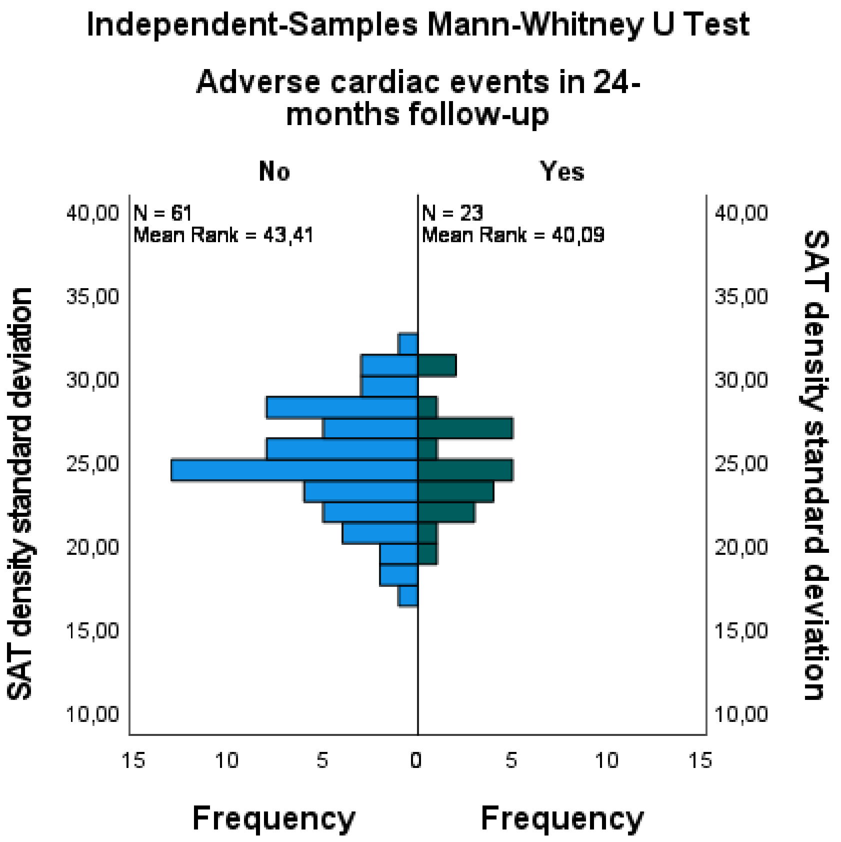

VAT mean density (HU) across Adverse cardiac events in 24-months follow-up

Independent-Samples Mann-Whitney U Test Summary

Total N	85
Mann-Whitney U	801,000
Wilcoxon W	1101,000
Test Statistic	801,000
Standard Error	102,412
Standardized Test Statistic	,674
Asymptotic Sig.(2-sided test)	,500

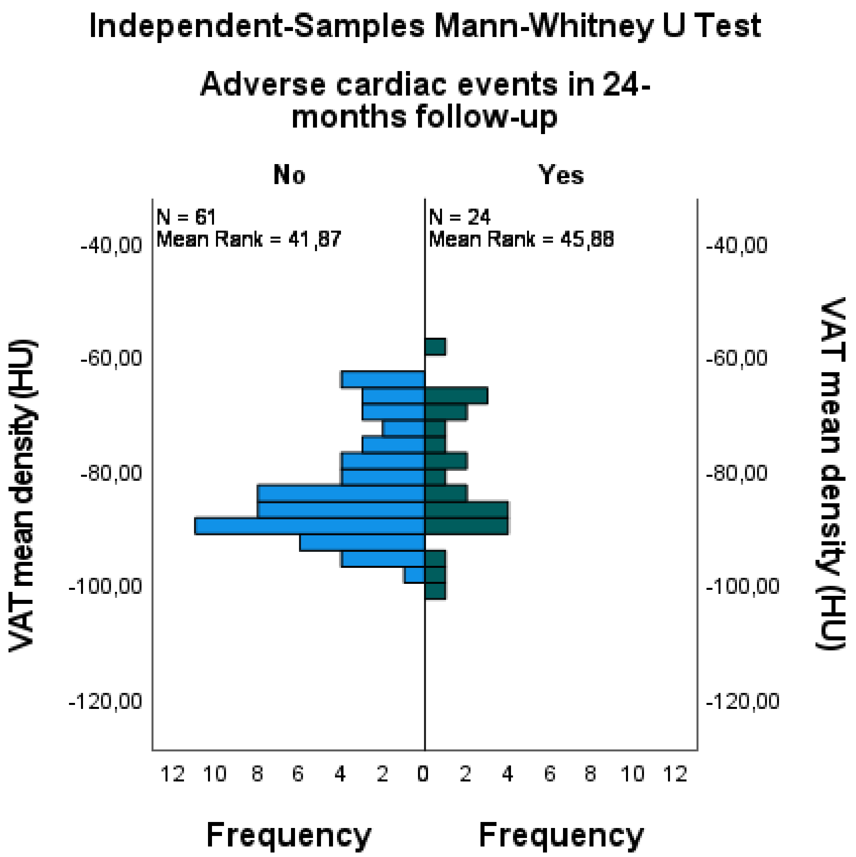

VAT median density (HU) across Adverse cardiac events in 24-months follow-up

Independent-Samples Mann-Whitney U Test Summary

Total N	85
Mann-Whitney U	802,500
Wilcoxon W	1102,500
Test Statistic	802,500
Standard Error	102,302
Standardized Test Statistic	,689
Asymptotic Sig.(2-sided test)	,491

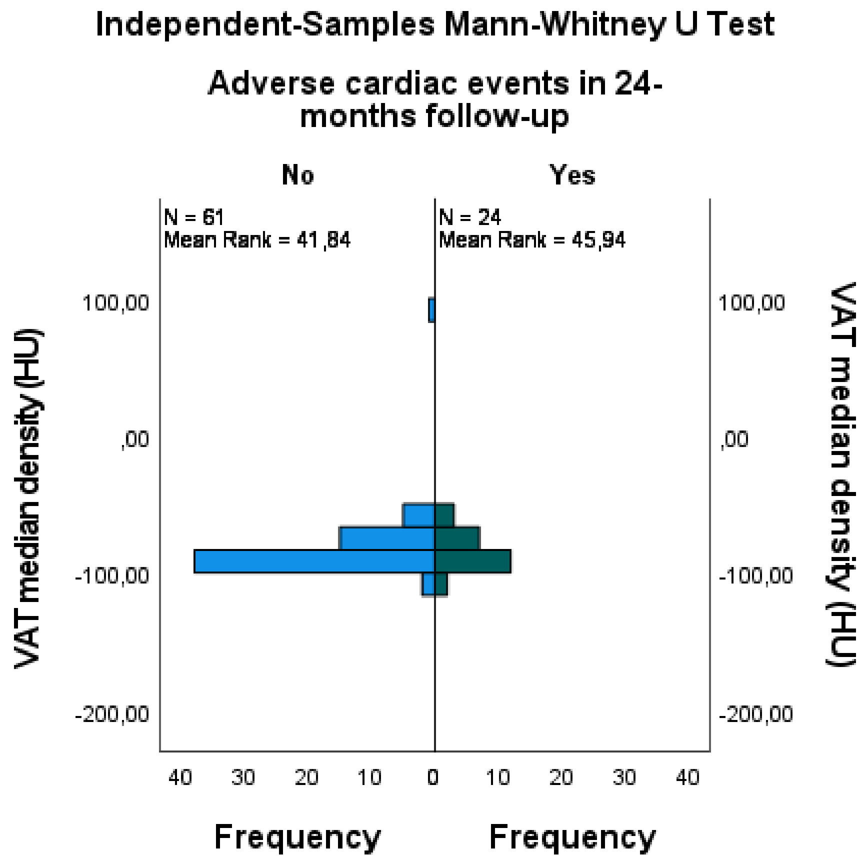

VAT density standard deviation across Adverse cardiac events in 24-months follow-up

Independent-Samples Mann-Whitney U Test Summary

Total N	84
Mann-Whitney U	799,000
Wilcoxon W	1099,000
Test Statistic	799,000
Standard Error	100,995
Standardized Test Statistic	,782
Asymptotic Sig.(2-sided test)	,434

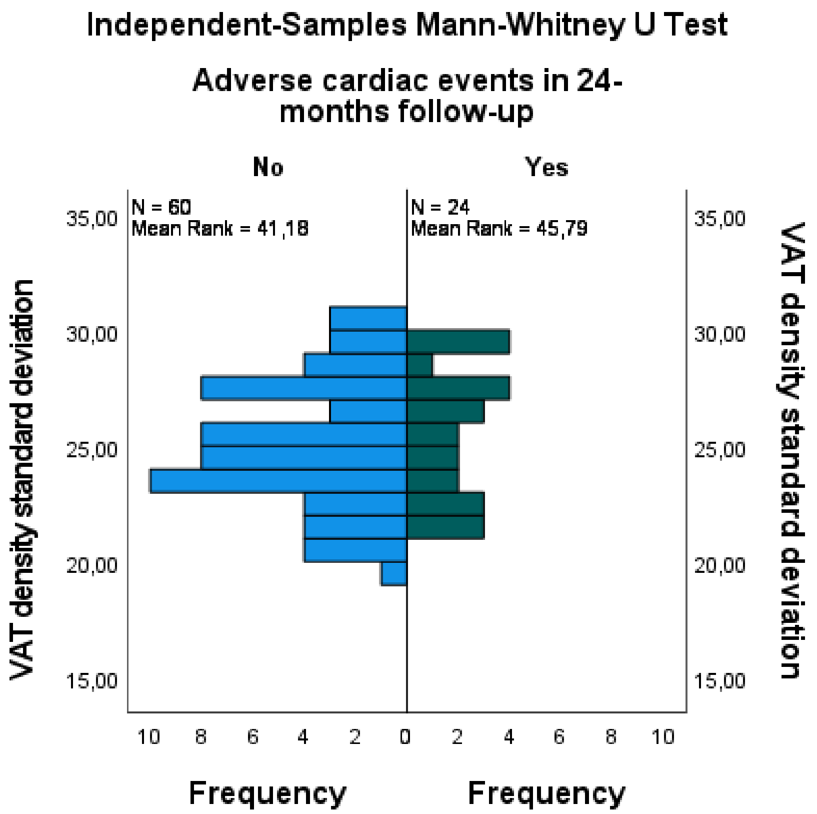

Right Psoas muscle mean density (HU) across Adverse cardiac events in 24-months follow-up

Independent-Samples Mann-Whitney U Test Summary

Total N	85
Mann-Whitney U	639,000
Wilcoxon W	939,000
Test Statistic	639,000
Standard Error	102,408
Standardized Test Statistic	-,908
Asymptotic Sig.(2-sided test)	,364

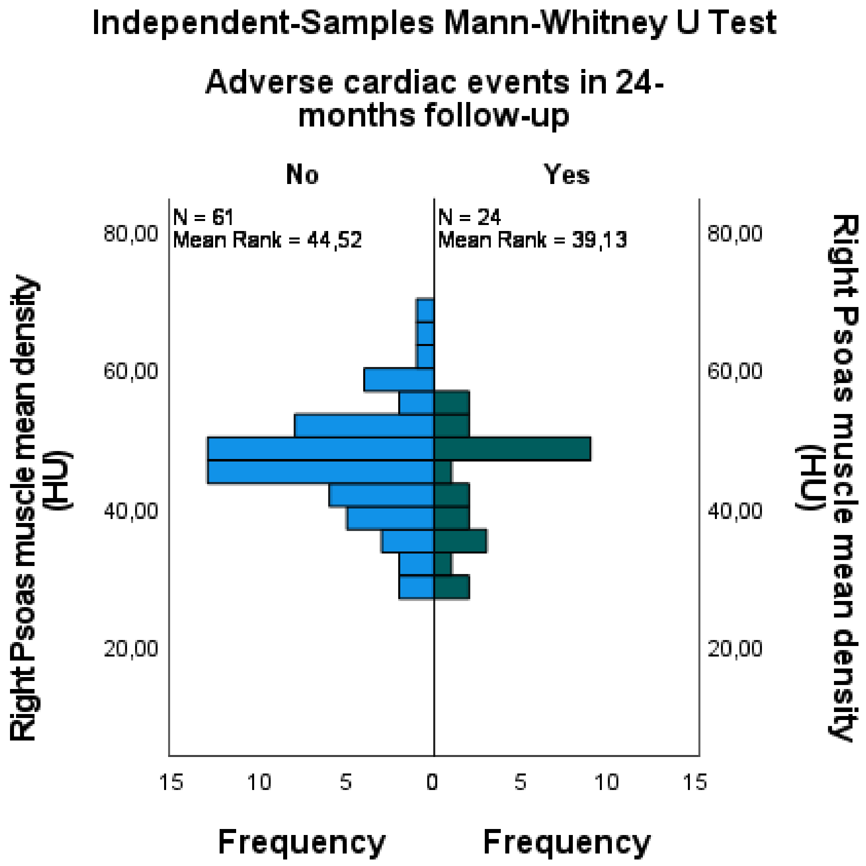

Right Psoas muscle median density (HU) across Adverse cardiac events in 24-months follow-up

Independent-Samples Mann-Whitney U Test Summary

Total N	85
Mann-Whitney U	657,500
Wilcoxon W	957,500
Test Statistic	657,500
Standard Error	102,333
Standardized Test Statistic	-,728
Asymptotic Sig.(2-sided test)	,467

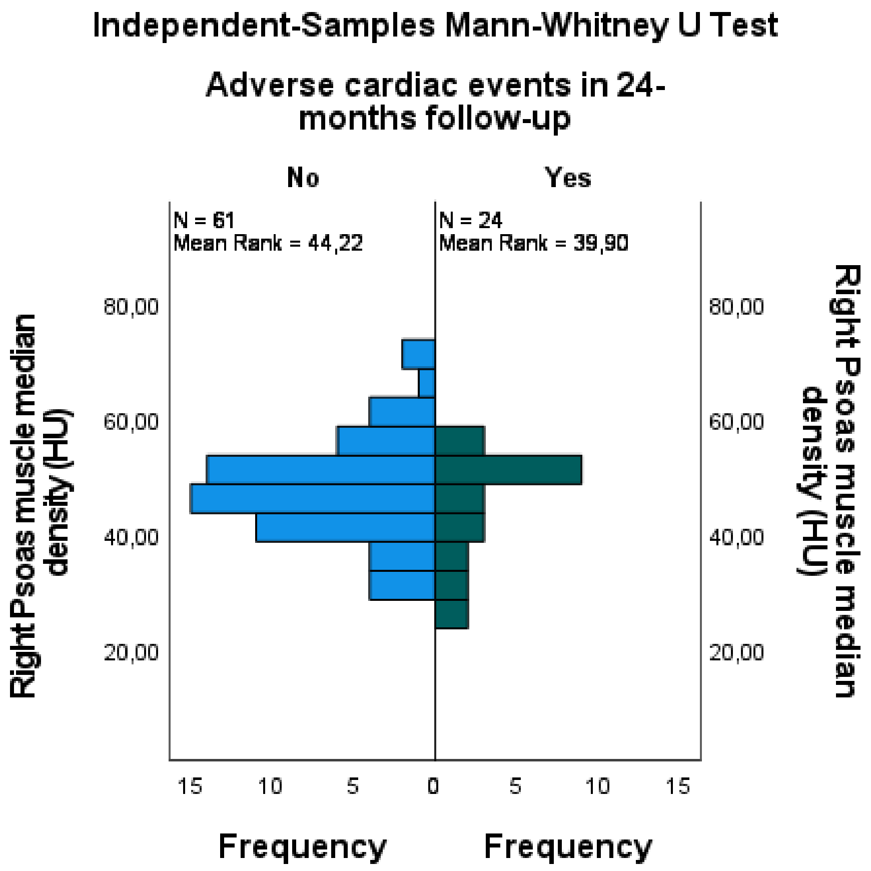

Right Psoas muscle density standard deviation across Adverse cardiac events in 24-months follow-up

Independent-Samples Mann-Whitney U Test Summary

Total N	85
Mann-Whitney U	626,500
Wilcoxon W	926,500
Test Statistic	626,500
Standard Error	102,429
Standardized Test Statistic	-1,030
Asymptotic Sig.(2-sided test)	,303

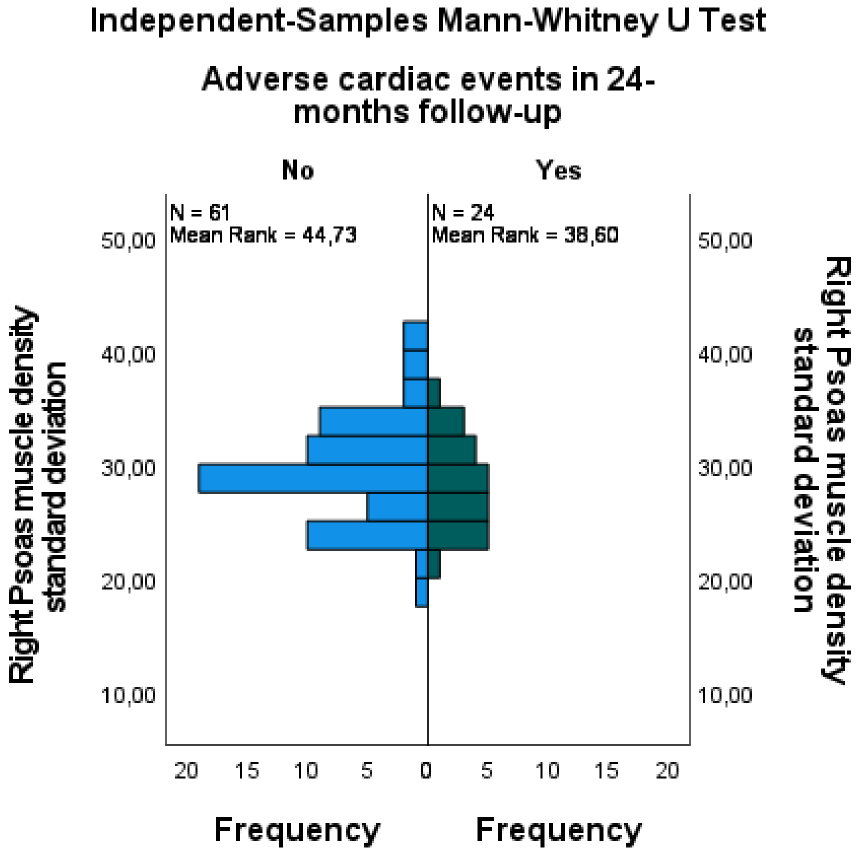

Left Psoas muscle mean density (HU) across Adverse cardiac events in 24-months follow-up

Independent-Samples Mann-Whitney U Test Summary

Total N	85
Mann-Whitney U	610,500
Wilcoxon W	910,500
Test Statistic	610,500
Standard Error	102,409
Standardized Test Statistic	-1,186
Asymptotic Sig.(2-sided test)	,235

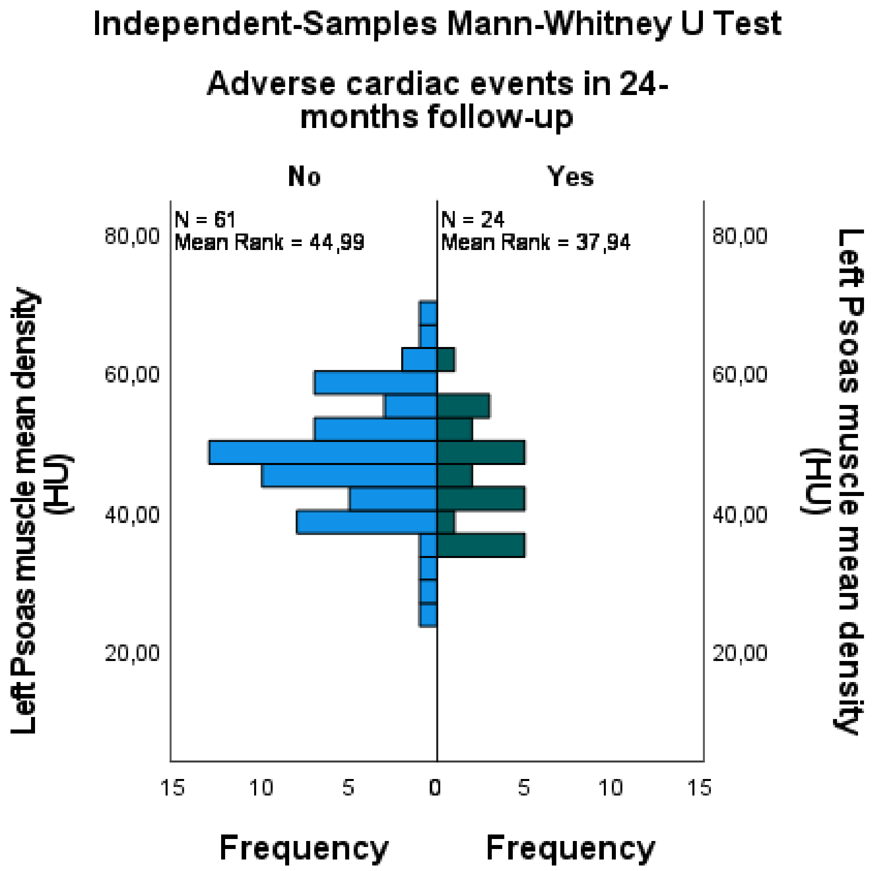

Left Psoas muscle median density (HU) across Adverse cardiac events in 24-months follow-up

Independent-Samples Mann-Whitney U Test Summary

Total N	85
Mann-Whitney U	602,500
Wilcoxon W	902,500
Test Statistic	602,500
Standard Error	102,300
Standardized Test Statistic	-1,266
Asymptotic Sig.(2-sided test)	,206

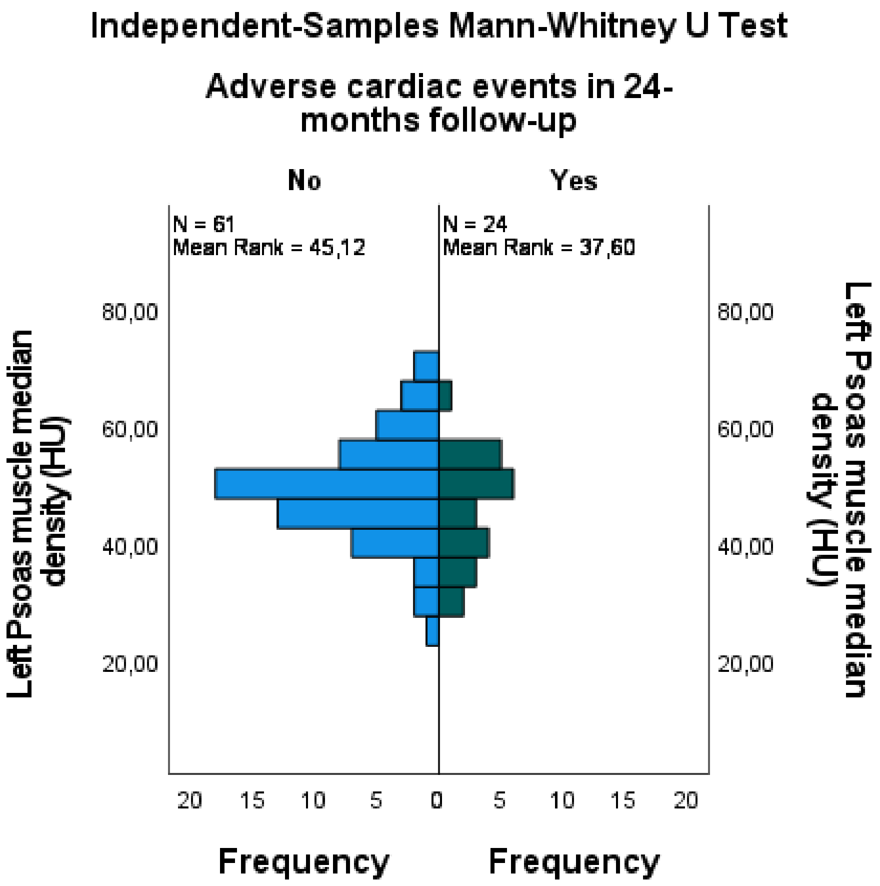

Left Psoas muscle density standard deviation across Adverse cardiac events in 24-months follow-up

Independent-Samples Mann-Whitney U Test Summary

Total N	85
Mann-Whitney U	637,000
Wilcoxon W	937,000
Test Statistic	637,000
Standard Error	102,430
Standardized Test Statistic	-,927
Asymptotic Sig.(2-sided test)	,354

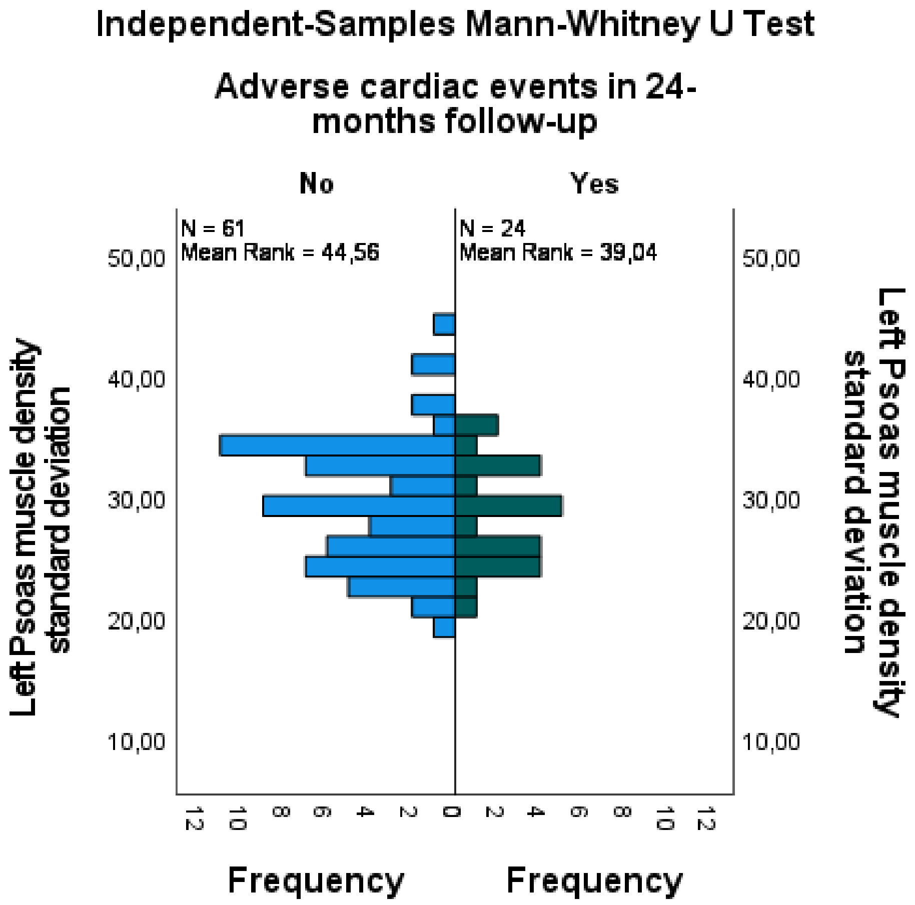

Independent-Samples Mann-Whitney U Test
Adverse cardiac events in 24-months follow-up
No
Yes
N = 61
Mean Rank = 44,56
N = 24
Mean Rank = 39,04
50,00
40,00
30,00
20,00
10,00
Left Psoas muscle density standard deviation
12 10 8 6 4 2 0 2 4 6 8 10 12
Frequency
Frequency

Cerebrovascular events in 24-months follow-up with other variables

Hypothesis Test Summary

	Null Hypothesis	Test	Sig.[a,b]	Decision
1	The distribution of Psoas/height is the same across categories of Cerebrovascular events in 24-months follow-up.	Independent-Samples Mann-Whitney U Test	<,001	Reject the null hypothesis.
2	The distribution of Anterior SAT distance is the same across categories of Cerebrovascular events in 24-months follow-up.	Independent-Samples Mann-Whitney U Test	,001	Reject the null hypothesis.
3	The distribution of Posterior SAT distance is the same across categories of Cerebrovascular events in 24-months follow-up.	Independent-Samples Mann-Whitney U Test	<,001	Reject the null hypothesis.
4	The distribution of Anterior+Posterior SAT distance is the same across categories of Cerebrovascular events in 24-months follow-up.	Independent-Samples Mann-Whitney U Test	<,001	Reject the null hypothesis.
5	The distribution of VAT distance is the same across categories of Cerebrovascular events in 24-months follow-up.	Independent-Samples Mann-Whitney U Test	,016	Reject the null hypothesis.
6	The distribution of Right common femoral artery area (mm2) is the same across categories of Cerebrovascular events in 24-months follow-up.	Independent-Samples Mann-Whitney U Test	,257	Retain the null hypothesis.
7	The distribution of Left common femoral artery area (mm2) is the same across categories of Cerebrovascular events in 24-months follow-up.	Independent-Samples Mann-Whitney U Test	,684	Retain the null hypothesis.
8	The distribution of FAT area (cm2) is the same across categories of Cerebrovascular events in 24-months follow-up.	Independent-Samples Mann-Whitney U Test	,019	Reject the null hypothesis.
9	The distribution of SAT area (cm2) is the same across categories of Cerebrovascular events in 24-months follow-up.	Independent-Samples Mann-Whitney U Test	,019	Reject the null hypothesis.

10	The distribution of VAT area (cm2) is the same across categories of Cerebrovascular events in 24-months follow-up.	Independent-Samples Mann-Whitney U Test	,066	Retain the null hypothesis.
11	The distribution of Right Psoas muscle area (cm2) is the same across categories of Cerebrovascular events in 24-months follow-up.	Independent-Samples Mann-Whitney U Test	,009	Reject the null hypothesis.
12	The distribution of Left Psoas muscle area (cm2) is the same across categories of Cerebrovascular events in 24-months follow-up.	Independent-Samples Mann-Whitney U Test	,007	Reject the null hypothesis.
13	The distribution of FAT mean density (HU) is the same across categories of Cerebrovascular events in 24-months follow-up.	Independent-Samples Mann-Whitney U Test	,361	Retain the null hypothesis.
14	The distribution of FAT median density (HU) is the same across categories of Cerebrovascular events in 24-months follow-up.	Independent-Samples Mann-Whitney U Test	,436	Retain the null hypothesis.
15	The distribution of FAT density standard deviation is the same across categories of Cerebrovascular events in 24-months follow-up.	Independent-Samples Mann-Whitney U Test	,779	Retain the null hypothesis.
16	The distribution of SAT mean density (HU) is the same across categories of Cerebrovascular events in 24-months follow-up.	Independent-Samples Mann-Whitney U Test	,343	Retain the null hypothesis.
17	The distribution of SAT median density (HU) is the same across categories of Cerebrovascular events in 24-months follow-up.	Independent-Samples Mann-Whitney U Test	,197	Retain the null hypothesis.
18	The distribution of SAT density standard deviation is the same across categories of Cerebrovascular events in 24-months follow-up.	Independent-Samples Mann-Whitney U Test	,935	Retain the null hypothesis.

19	The distribution of VAT mean density (HU) is the same across categories of Cerebrovascular events in 24-months follow-up.	Independent-Samples Mann-Whitney U Test	,353	Retain the null hypothesis.
20	The distribution of VAT median density (HU) is the same across categories of Cerebrovascular events in 24-months follow-up.	Independent-Samples Mann-Whitney U Test	,305	Retain the null hypothesis.
21	The distribution of VAT density standard deviation is the same across categories of Cerebrovascular events in 24-months follow-up.	Independent-Samples Mann-Whitney U Test	,157	Retain the null hypothesis.
22	The distribution of Right Psoas muscle mean density (HU) is the same across categories of Cerebrovascular events in 24-months follow-up.	Independent-Samples Mann-Whitney U Test	,837	Retain the null hypothesis.
23	The distribution of Right Psoas muscle median density (HU) is the same across categories of Cerebrovascular events in 24-months follow-up.	Independent-Samples Mann-Whitney U Test	,952	Retain the null hypothesis.
24	The distribution of Right Psoas muscle density standard deviation is the same across categories of Cerebrovascular events in 24-months follow-up.	Independent-Samples Mann-Whitney U Test	,086	Retain the null hypothesis.
25	The distribution of Left Psoas muscle mean density (HU) is the same across categories of Cerebrovascular events in 24-months follow-up.	Independent-Samples Mann-Whitney U Test	,165	Retain the null hypothesis.
26	The distribution of Left Psoas muscle median density (HU) is the same across categories of Cerebrovascular events in 24-months follow-up.	Independent-Samples Mann-Whitney U Test	,234	Retain the null hypothesis.
27	The distribution of Left Psoas muscle density standard deviation is the same across categories of Cerebrovascular events in 24-months follow-up.	Independent-Samples Mann-Whitney U Test	,292	Retain the null hypothesis.

a. The significance level is ,050.

b. Asymptotic significance is displayed.

In this case, the hypothesis of equal medians ($p < 0.05$) is rejected for the variables Psoas/height, Anterior SAT distance, Posterior SAT distance, Anterior + Posterior SAT distance, VAT distance, FAT area (cm2), SAT area (cm2), Right Psoas muscle area (cm2) and Left Psoas muscle area (cm2), while for the rest we accept the null hypothesis ($p > 0.05$).

Psoas/height across Cerebrovascular events in 24-months follow-up

Independent-Samples Mann-Whitney U Test Summary

Total N	85
Mann-Whitney U	298,500
Wilcoxon W	551,500
Test Statistic	298,500
Standard Error	99,664
Standardized Test Statistic	-3,958
Asymptotic Sig.(2-sided test)	<,001

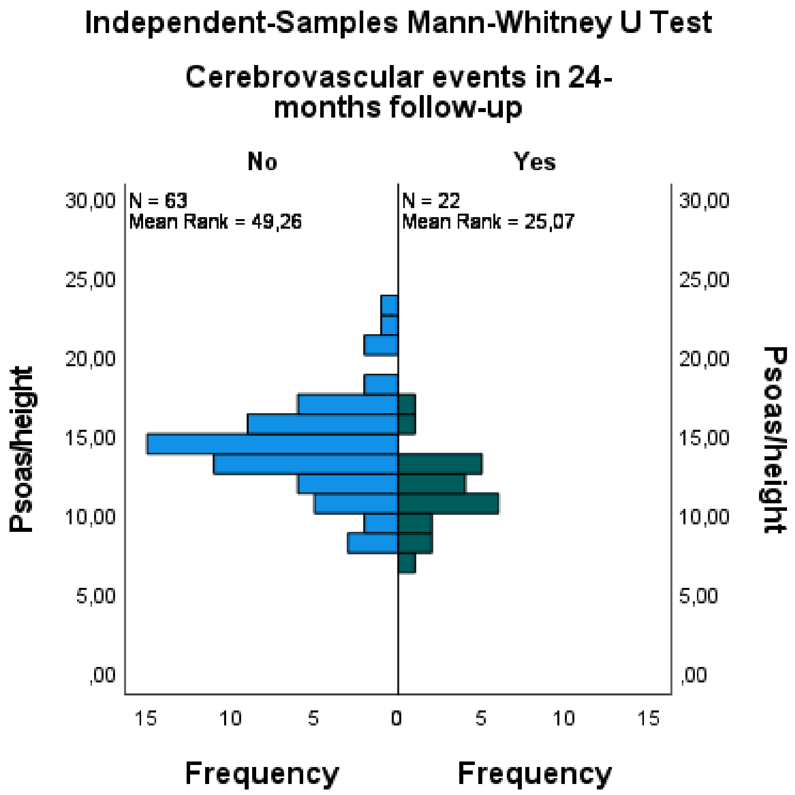

Anterior SAT distance across Cerebrovascular events in 24-months follow-up

Independent-Samples Mann-Whitney U Test Summary

Total N	85
Mann-Whitney U	365,500
Wilcoxon W	618,500
Test Statistic	365,500
Standard Error	99,650
Standardized Test Statistic	-3,286
Asymptotic Sig.(2-sided test)	,001

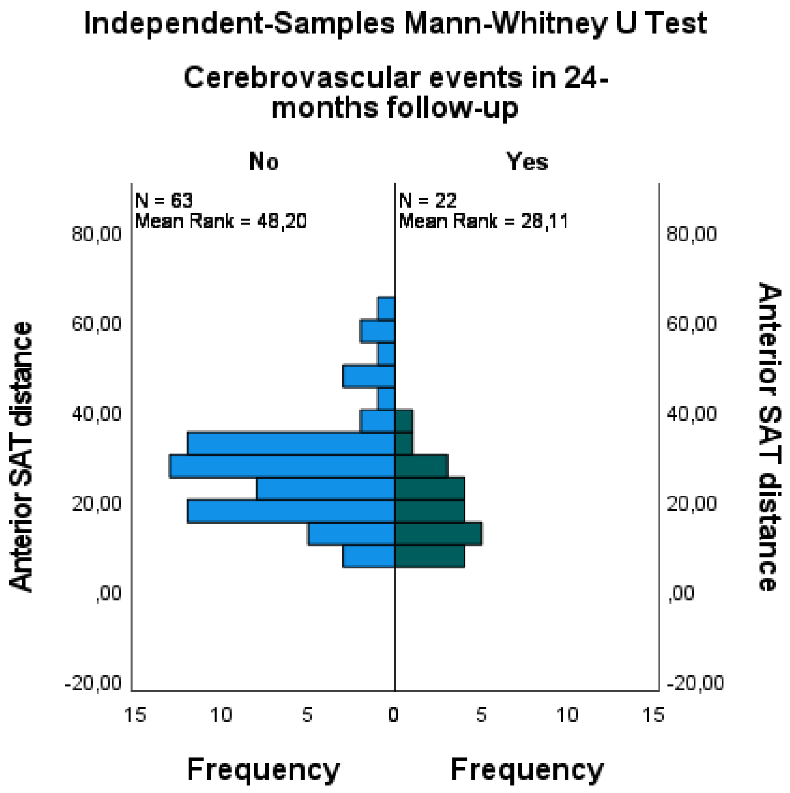

Posterior SAT distance across Cerebrovascular events in 24-months follow-up

Independent-Samples Mann-Whitney U Test Summary

Total N	85
Mann-Whitney U	361,000
Wilcoxon W	614,000
Test Statistic	361,000
Standard Error	99,653
Standardized Test Statistic	-3,332
Asymptotic Sig.(2-sided test)	<,001

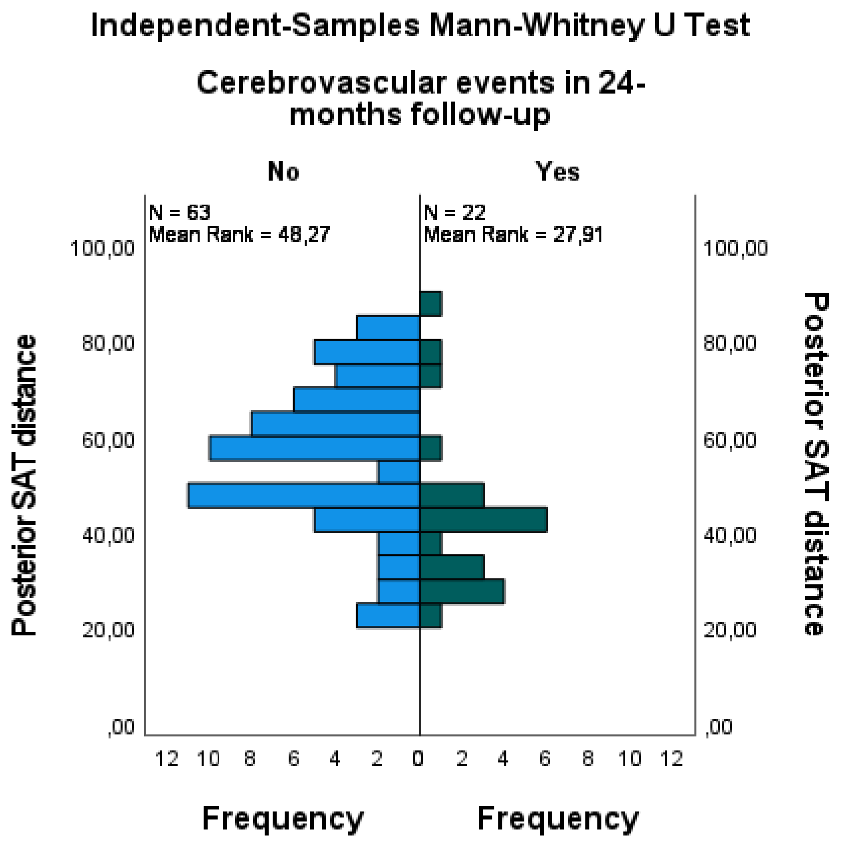

Anterior+Posterior SAT distance across Cerebrovascular events in 24-months follow-up

Independent-Samples Mann-Whitney U Test Summary

Total N	85
Mann-Whitney U	328,500
Wilcoxon W	581,500
Test Statistic	328,500
Standard Error	99,660
Standardized Test Statistic	-3,657
Asymptotic Sig.(2-sided test)	<,001

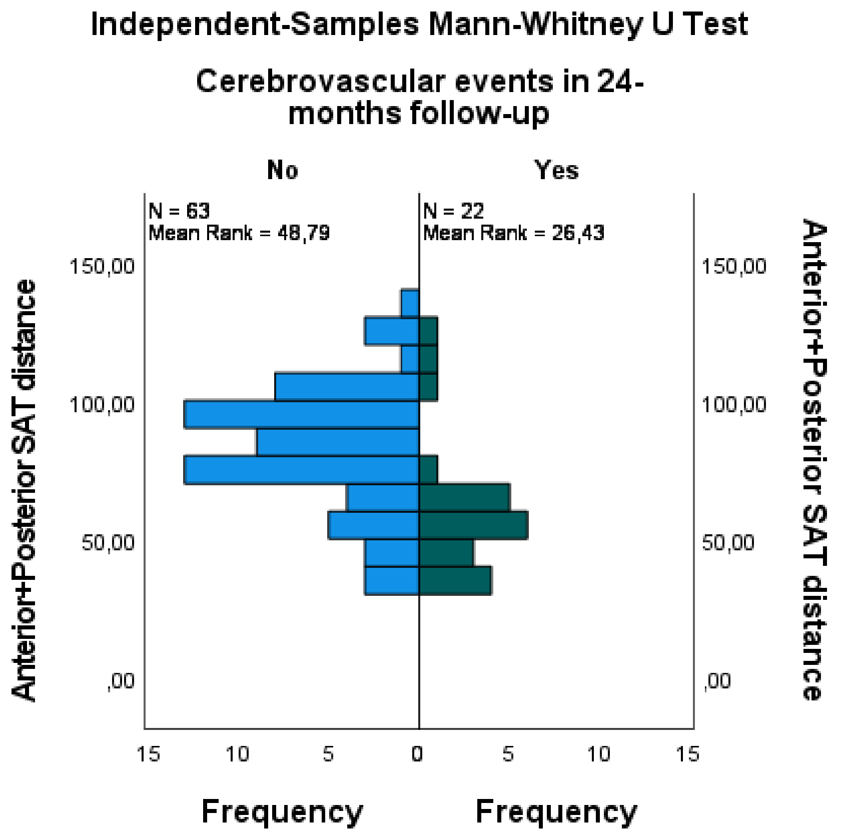

VAT distance across Cerebrovascular events in 24-months follow-up

Independent-Samples Mann-Whitney U Test Summary

Total N	84
Mann-Whitney U	445,500
Wilcoxon W	698,500
Test Statistic	445,500
Standard Error	98,274
Standardized Test Statistic	-2,407
Asymptotic Sig.(2-sided test)	,016

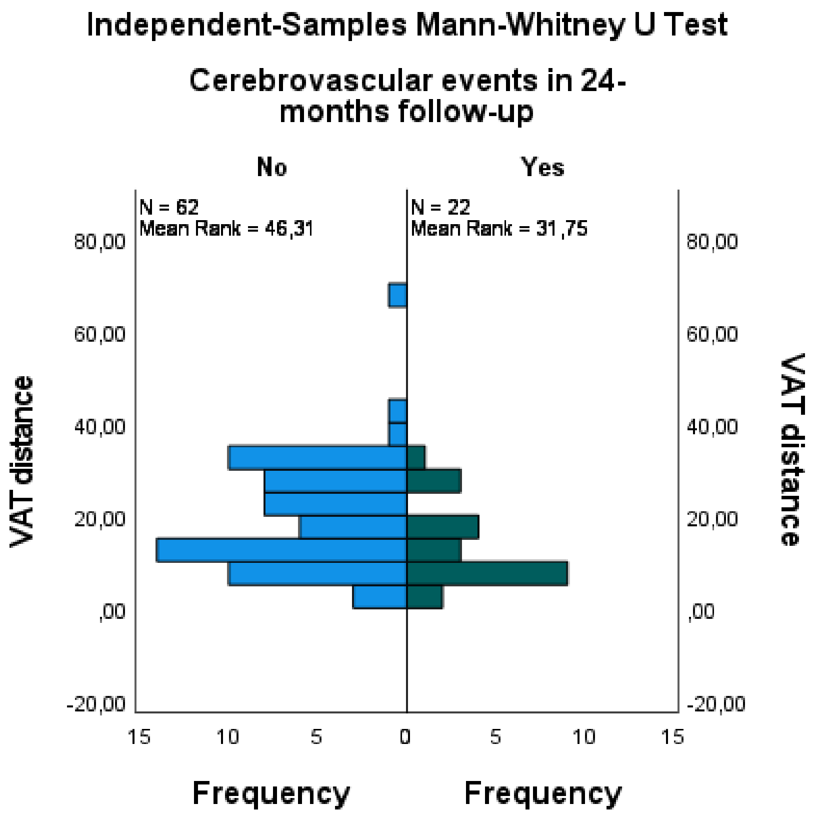

Right common femoral artery area (mm2) across Cerebrovascular events in 24-months follow-up

Independent-Samples Mann-Whitney U Test Summary

Total N	85
Mann-Whitney U	806,000
Wilcoxon W	1059,000
Test Statistic	806,000
Standard Error	99,648
Standardized Test Statistic	1,134
Asymptotic Sig.(2-sided test)	,257

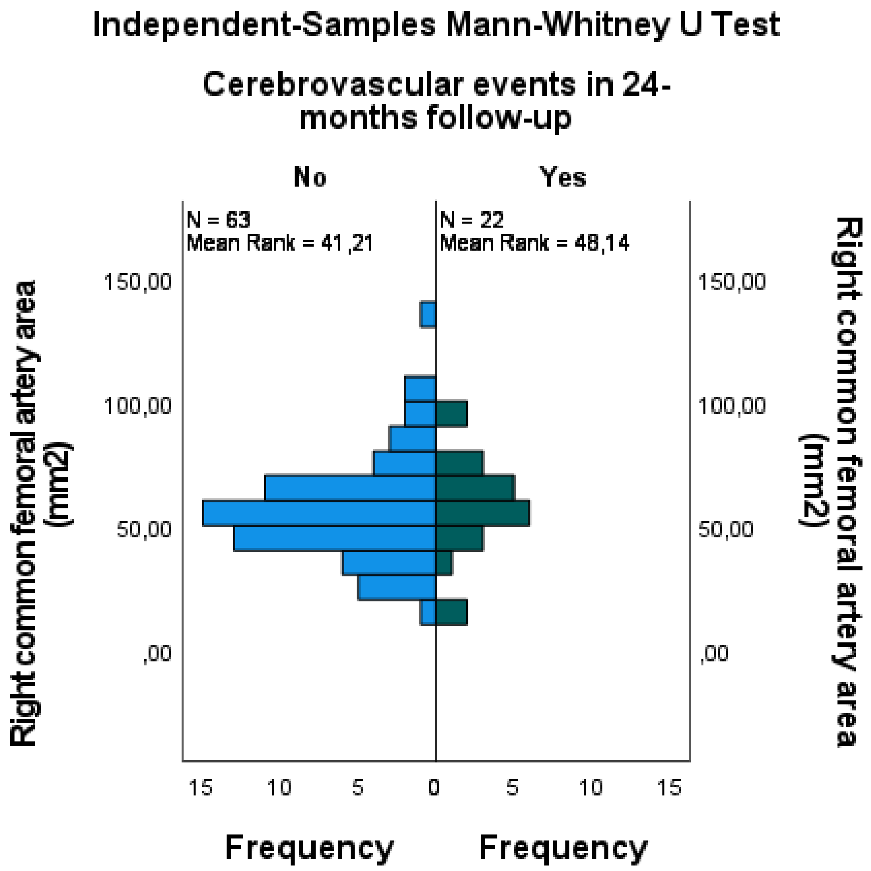

Left common femoral artery area (mm2) across Cerebrovascular events in 24-months follow-up

Independent-Samples Mann-Whitney U Test Summary

Total N	85
Mann-Whitney U	733,500
Wilcoxon W	986,500
Test Statistic	733,500
Standard Error	99,640
Standardized Test Statistic	,406
Asymptotic Sig.(2-sided test)	,684

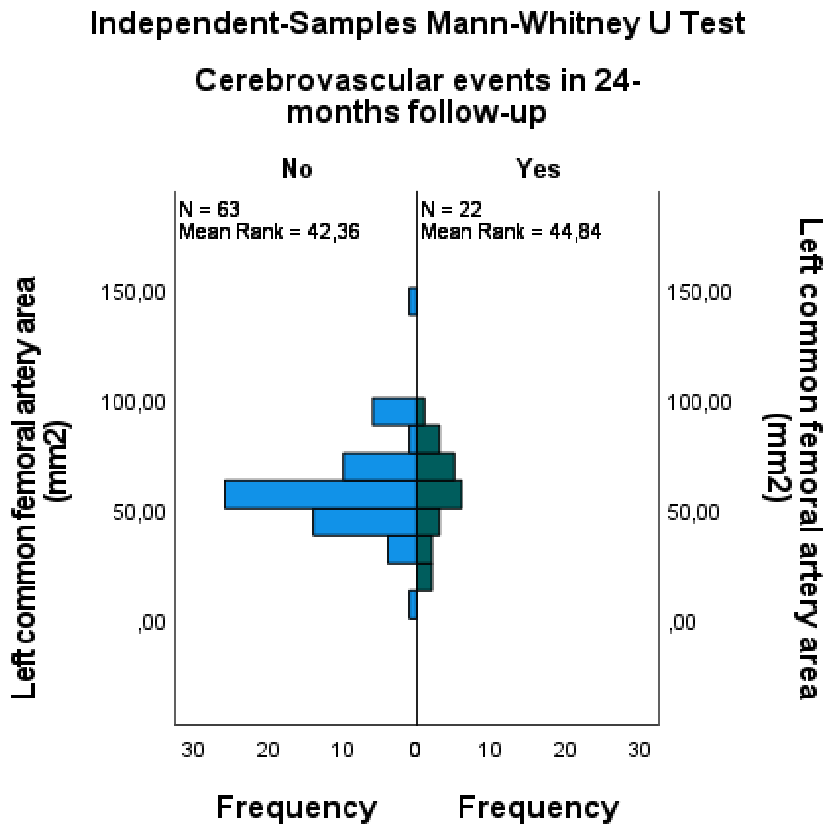

FAT area (cm2) across Cerebrovascular events in 24-months follow-up

Independent-Samples Mann-Whitney U Test Summary

Total N	85
Mann-Whitney U	460,000
Wilcoxon W	713,000
Test Statistic	460,000
Standard Error	99,664
Standardized Test Statistic	-2,338
Asymptotic Sig.(2-sided test)	,019

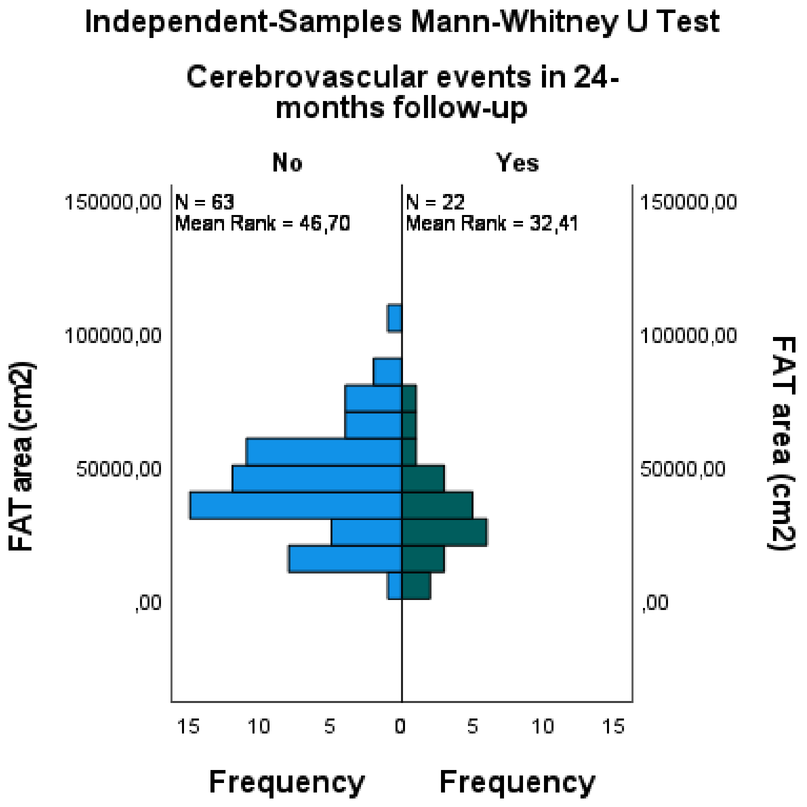

SAT area (cm2) across Cerebrovascular events in 24-months follow-up

Independent-Samples Mann-Whitney U Test Summary

Total N	85
Mann-Whitney U	459,500
Wilcoxon W	712,500
Test Statistic	459,500
Standard Error	99,664
Standardized Test Statistic	-2,343
Asymptotic Sig.(2-sided test)	,019

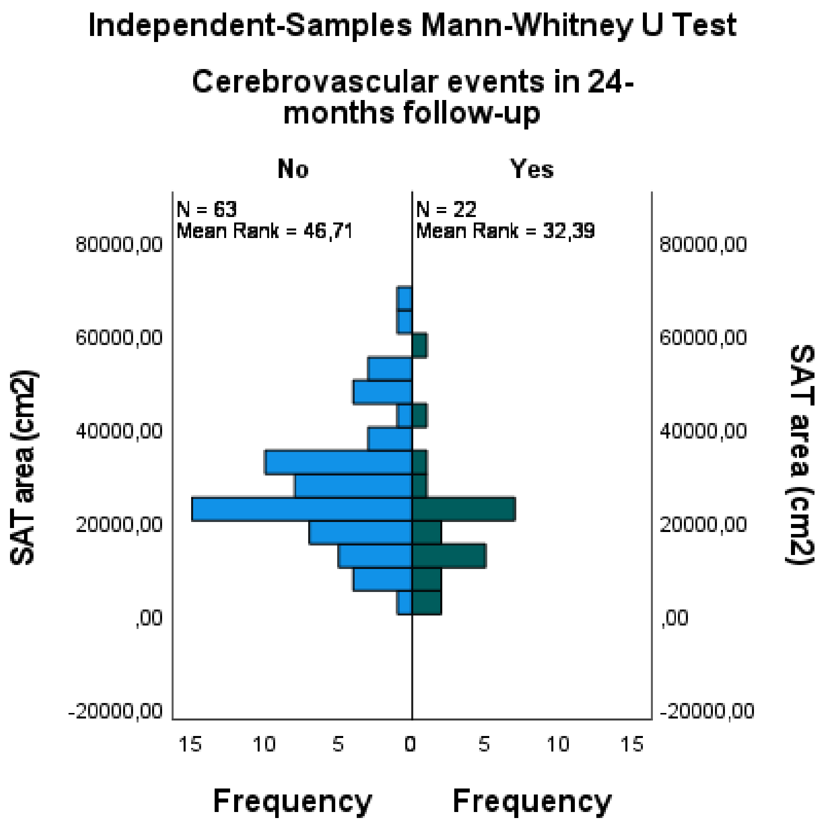

VAT area (cm2) across Cerebrovascular events in 24-months follow-up

Independent-Samples Mann-Whitney U Test Summary

Total N	84
Mann-Whitney U	501,000
Wilcoxon W	754,000
Test Statistic	501,000
Standard Error	98,294
Standardized Test Statistic	-1,841
Asymptotic Sig.(2-sided test)	,066

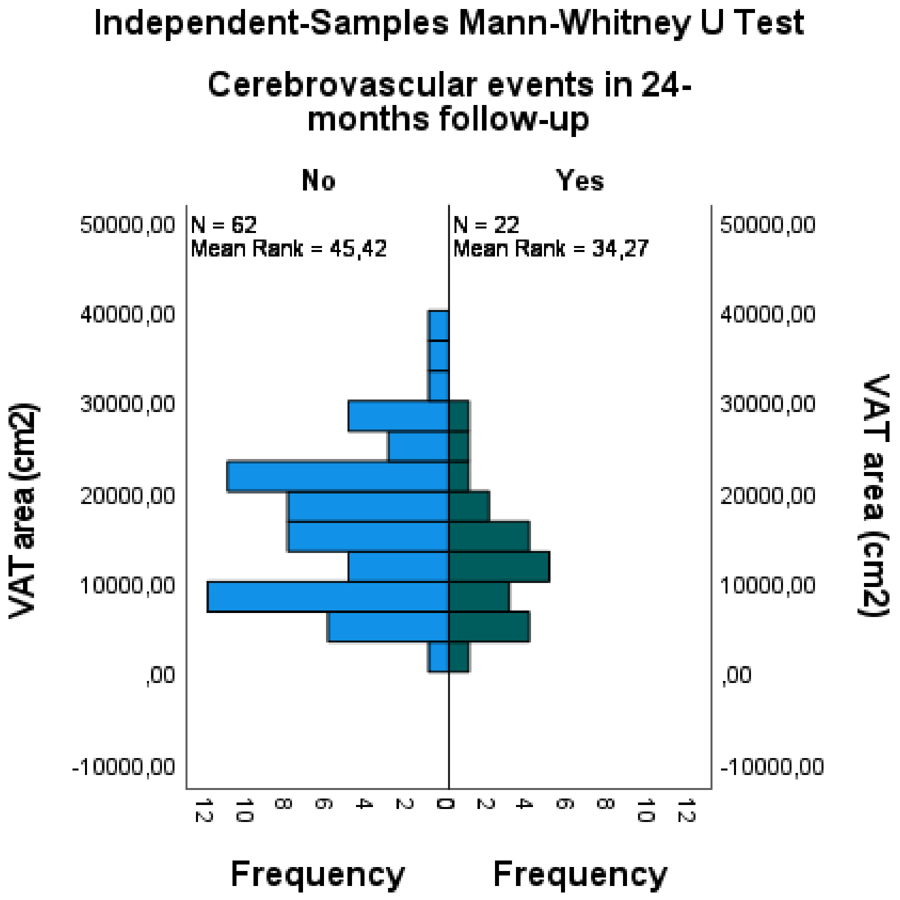

Right Psoas muscle area (cm2) across Cerebrovascular events in 24-months follow-up

Independent-Samples Mann-Whitney U Test Summary

Total N	85
Mann-Whitney U	433,000
Wilcoxon W	686,000
Test Statistic	433,000
Standard Error	99,662
Standardized Test Statistic	-2,609
Asymptotic Sig.(2-sided test)	,009

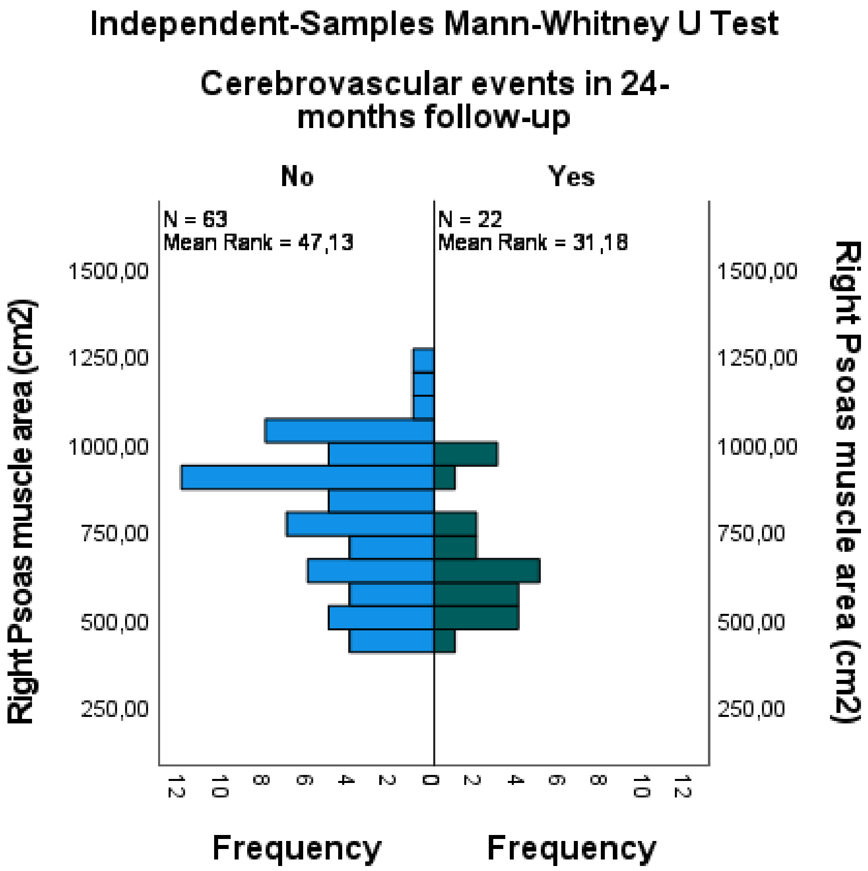

Left Psoas muscle area (cm2) across Cerebrovascular events in 24-months follow-up

Independent-Samples Mann-Whitney U Test Summary

Total N	85
Mann-Whitney U	424,000
Wilcoxon W	677,000
Test Statistic	424,000
Standard Error	99,663
Standardized Test Statistic	-2,699

Asymptotic Sig.(2-sided test)	,007

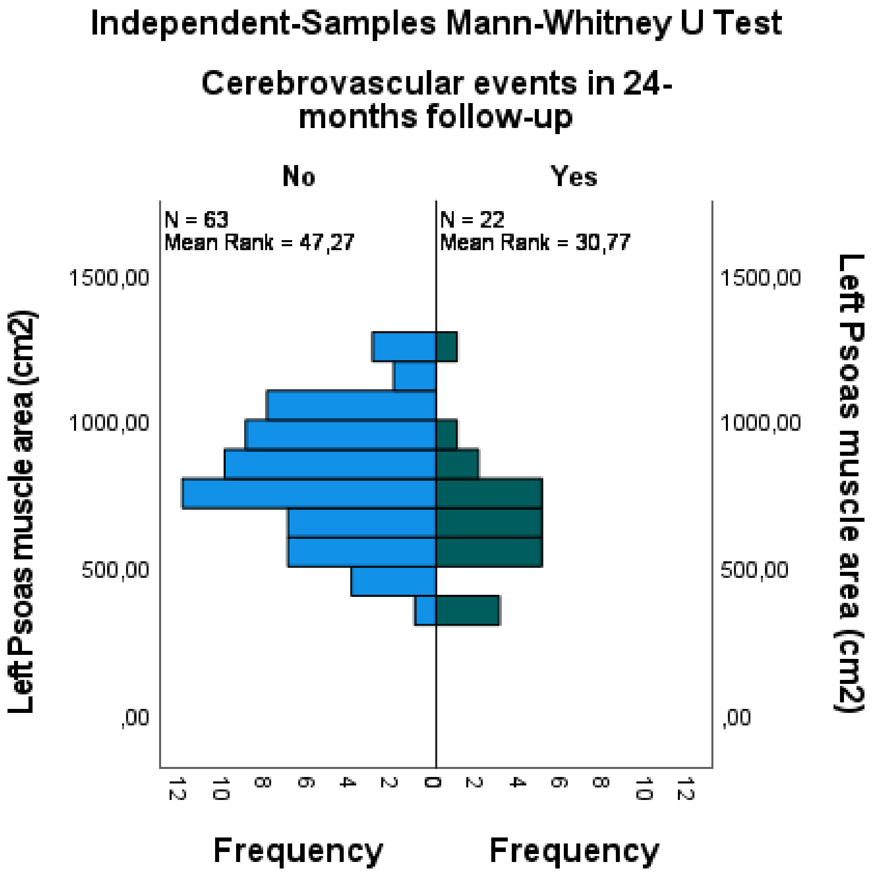

FAT mean density (HU) across Cerebrovascular events in 24-months follow-up

Independent-Samples Mann-Whitney U Test Summary

Total N	85
Mann-Whitney U	602,000
Wilcoxon W	855,000
Test Statistic	602,000

Standard Error	99,648
Standardized Test Statistic	-,913
Asymptotic Sig.(2-sided test)	,361

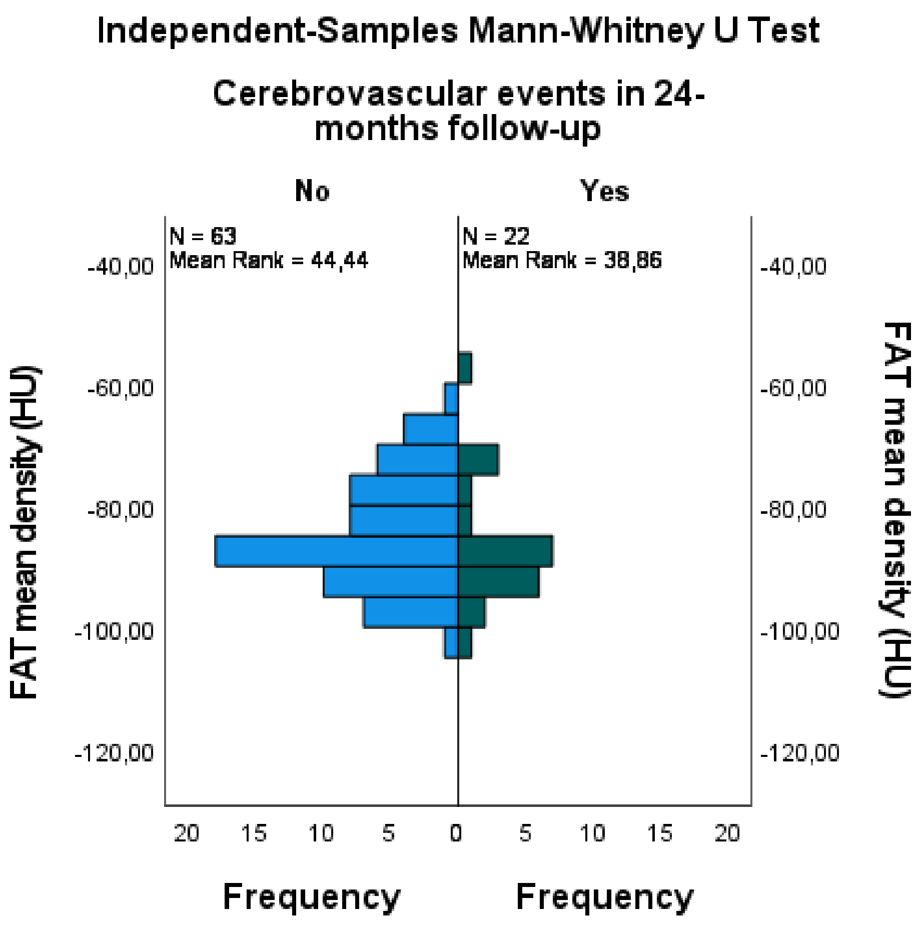

FAT median density (HU) across Cerebrovascular events in 24-months follow-up

Independent-Samples Mann-Whitney U Test Summary

Total N	85
Mann-Whitney U	615,500
Wilcoxon W	868,500

Test Statistic	615,500
Standard Error	99,544
Standardized Test Statistic	-,779
Asymptotic Sig.(2-sided test)	,436

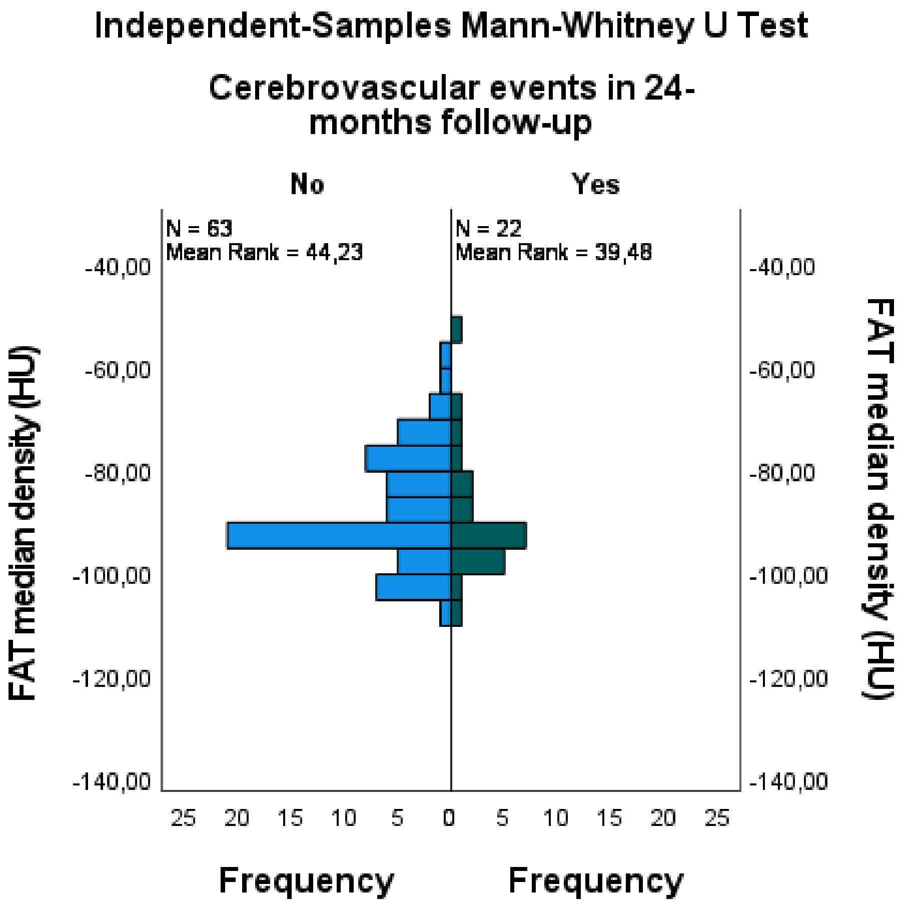

FAT density standard deviation across Cerebrovascular events in 24-months follow-up

Independent-Samples Mann-Whitney U Test Summary

Total N	85
Mann-Whitney U	665,000

Wilcoxon W	918,000
Test Statistic	665,000
Standard Error	99,664
Standardized Test Statistic	-,281
Asymptotic Sig.(2-sided test)	,779

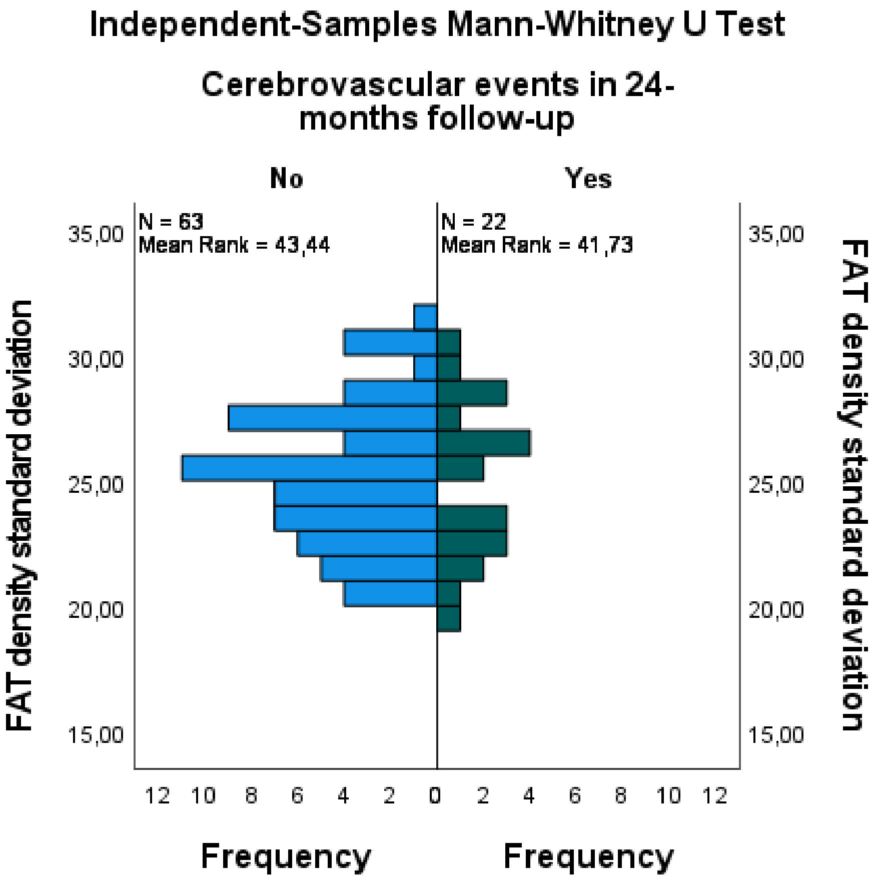

SAT mean density (HU) across Cerebrovascular events in 24-months follow-up

Independent-Samples Mann-Whitney U Test Summary

Total N	85

Mann-Whitney U	598,500
Wilcoxon W	851,500
Test Statistic	598,500
Standard Error	99,653
Standardized Test Statistic	-,948
Asymptotic Sig.(2-sided test)	,343

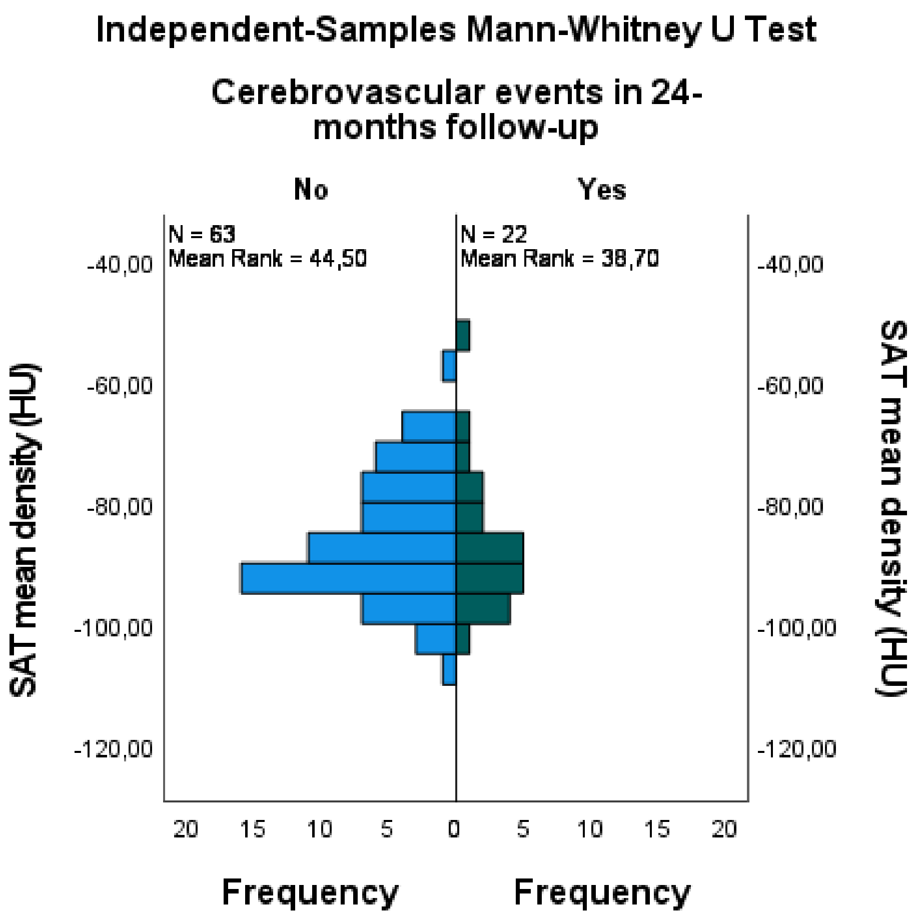

SAT median density (HU) across Cerebrovascular events in 24-months follow-up

Independent-Samples Mann-Whitney U Test Summary

Total N	83
Mann-Whitney U	528,000
Wilcoxon W	759,000
Test Statistic	528,000
Standard Error	95,393
Standardized Test Statistic	-1,289
Asymptotic Sig.(2-sided test)	,197

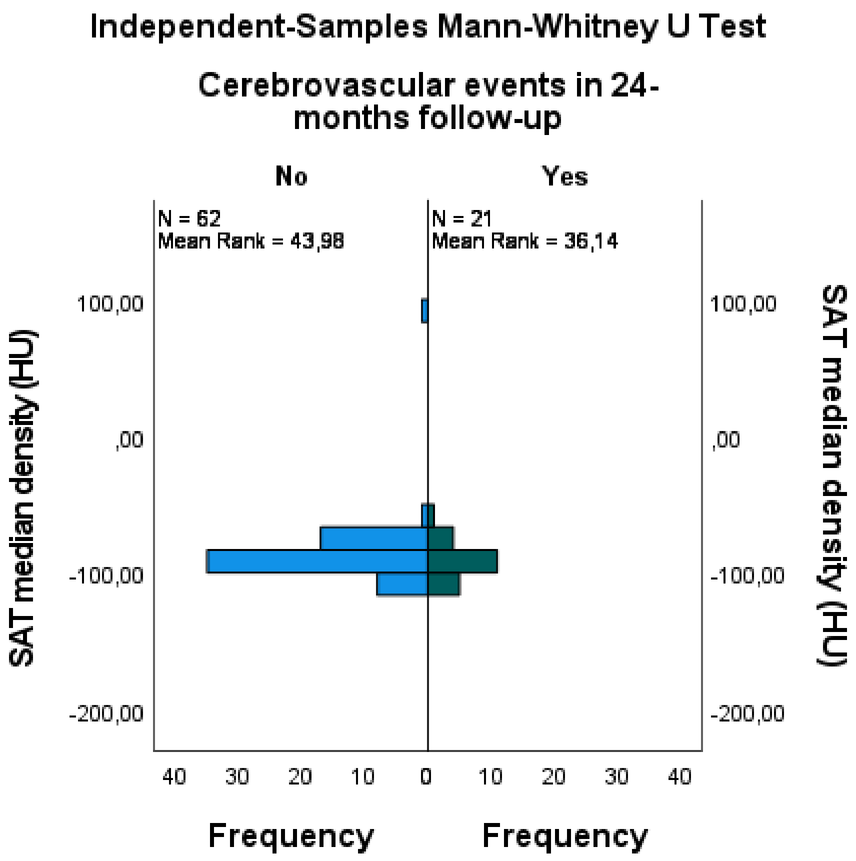

SAT density standard deviation across Cerebrovascular events in 24-months follow-up

Independent-Samples Mann-Whitney U Test Summary

Total N	84
Mann-Whitney U	690,000
Wilcoxon W	943,000
Test Statistic	690,000
Standard Error	98,294
Standardized Test Statistic	,081
Asymptotic Sig.(2-sided test)	,935

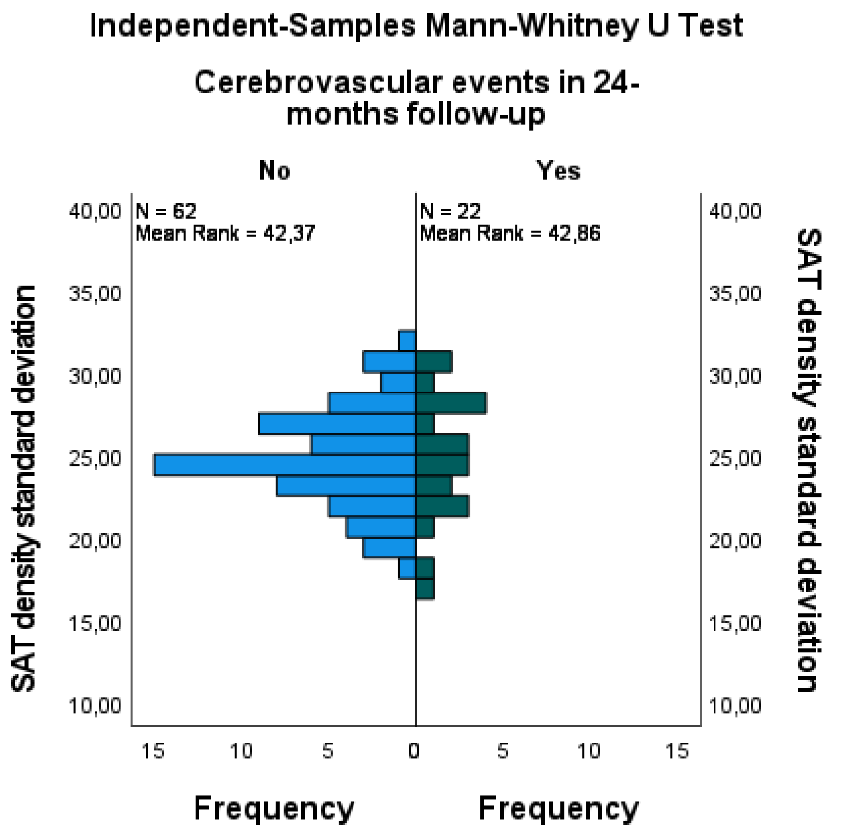

VAT mean density (HU) across Cerebrovascular events in 24-months follow-up

Independent-Samples Mann-Whitney U Test Summary

Total N	85
Mann-Whitney U	600,500
Wilcoxon W	853,500
Test Statistic	600,500
Standard Error	99,646
Standardized Test Statistic	-,928
Asymptotic Sig.(2-sided test)	,353

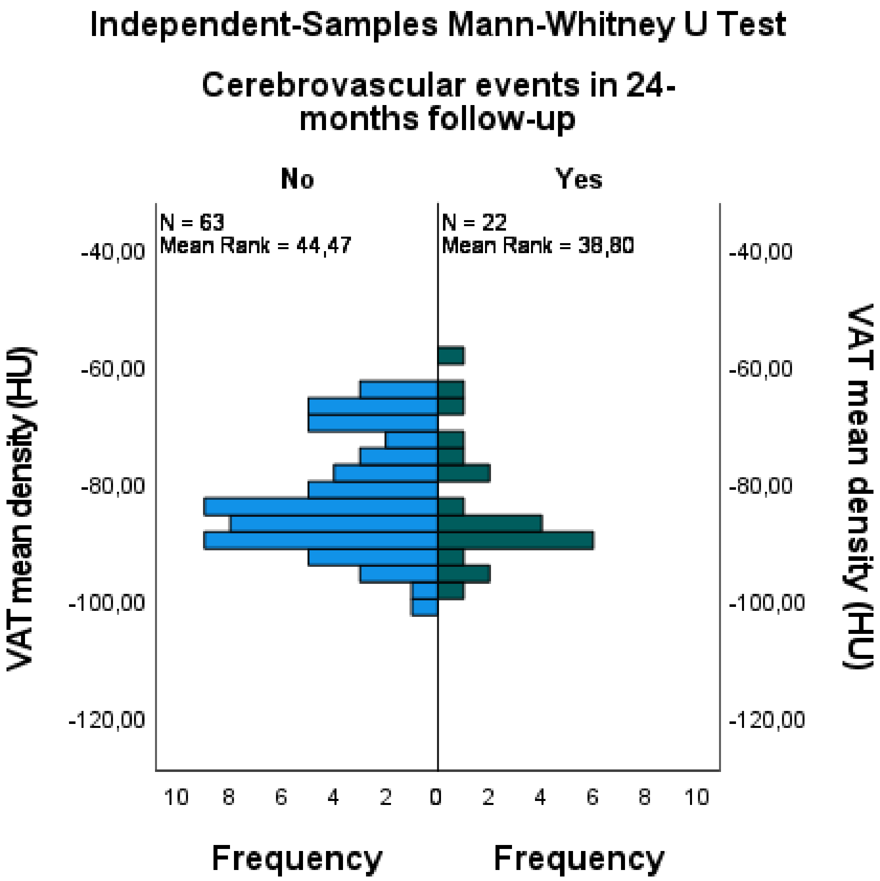

VAT median density (HU) across Cerebrovascular events in 24-months follow-up

Independent-Samples Mann-Whitney U Test Summary

Total N	85
Mann-Whitney U	591,000
Wilcoxon W	844,000
Test Statistic	591,000
Standard Error	99,540
Standardized Test Statistic	-1,025
Asymptotic Sig.(2-sided test)	,305

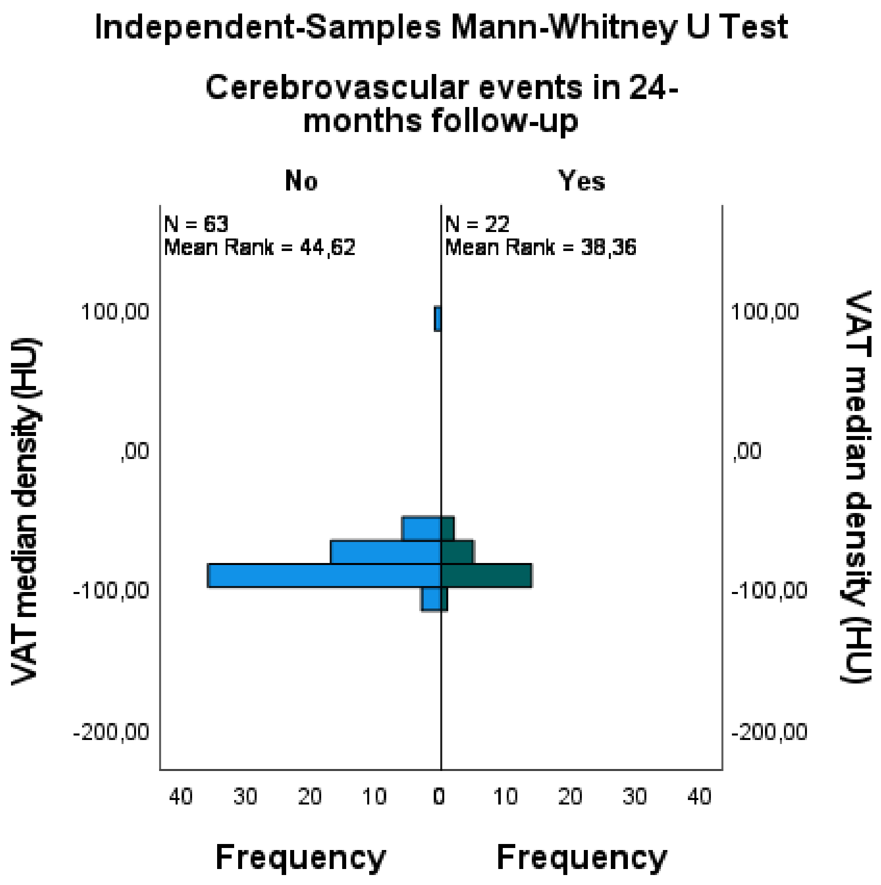

VAT density standard deviation across Cerebrovascular events in 24-months follow-up

Independent-Samples Mann-Whitney U Test Summary

Total N	84
Mann-Whitney U	821,000
Wilcoxon W	1074,000
Test Statistic	821,000
Standard Error	98,294
Standardized Test Statistic	1,414
Asymptotic Sig.(2-sided test)	,157

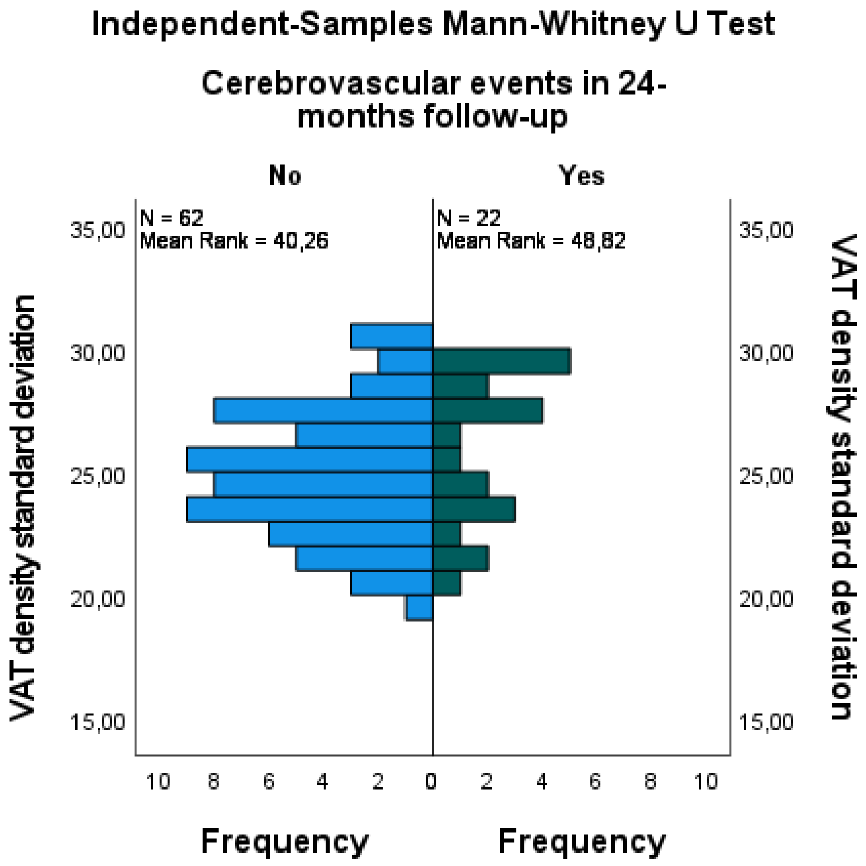

Right Psoas muscle mean density (HU) across Cerebrovascular events in 24-months follow-up

Independent-Samples Mann-Whitney U Test Summary

Total N	85
Mann-Whitney U	672,500
Wilcoxon W	925,500
Test Statistic	672,500
Standard Error	99,643
Standardized Test Statistic	-,206
Asymptotic Sig.(2-sided test)	,837

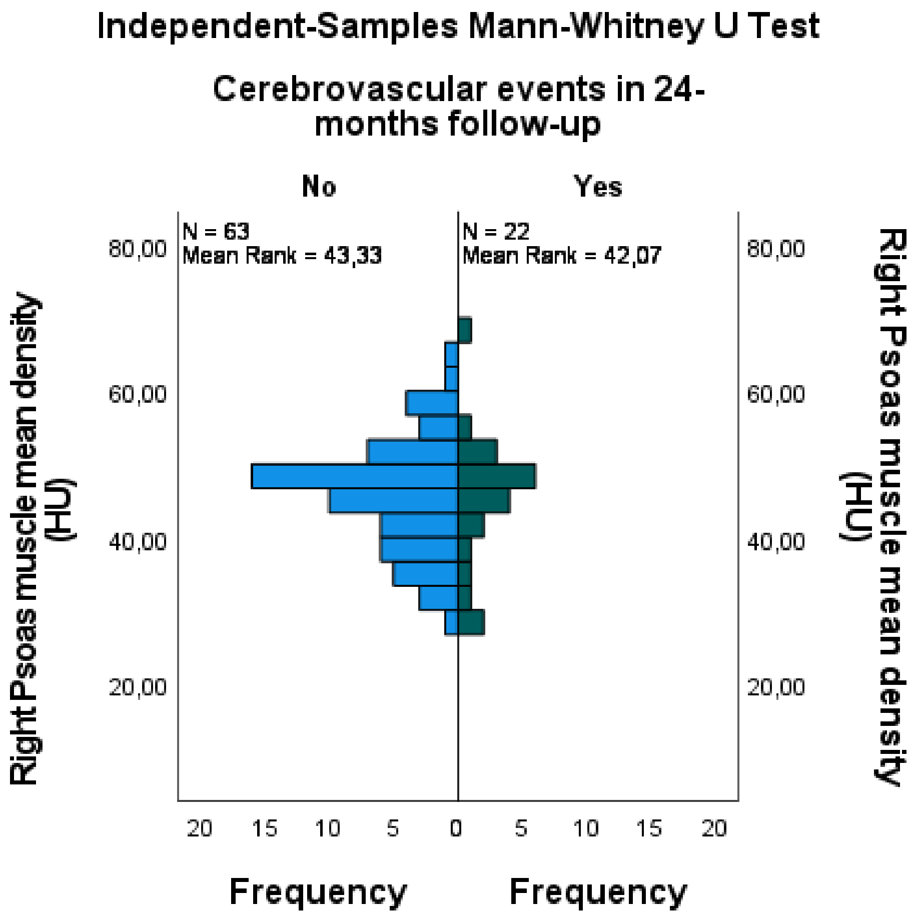

Right Psoas muscle median density (HU) across Cerebrovascular events in 24-months follow-up

Independent-Samples Mann-Whitney U Test Summary

Total N	85
Mann-Whitney U	687,000
Wilcoxon W	940,000
Test Statistic	687,000
Standard Error	99,570
Standardized Test Statistic	-,060
Asymptotic Sig.(2-sided test)	,952

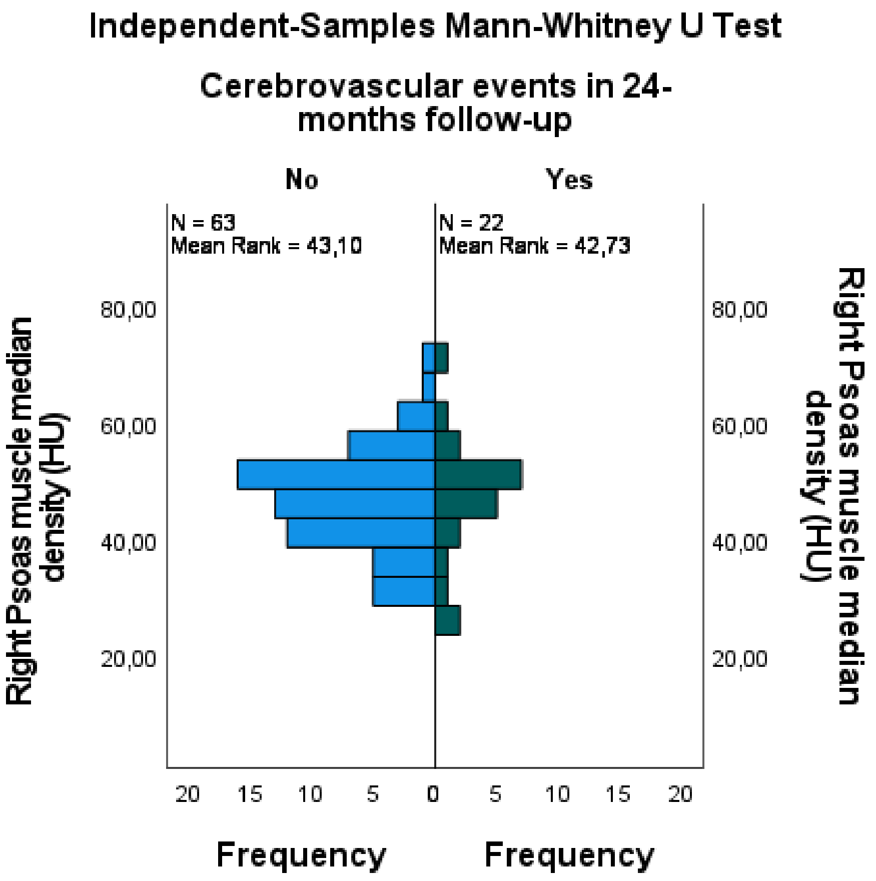

Right Psoas muscle density standard deviation across Cerebrovascular events in 24-months follow-up

Independent-Samples Mann-Whitney U Test Summary

Total N	85
Mann-Whitney U	522,000
Wilcoxon W	775,000
Test Statistic	522,000
Standard Error	99,663
Standardized Test Statistic	-1,716
Asymptotic Sig.(2-sided test)	,086

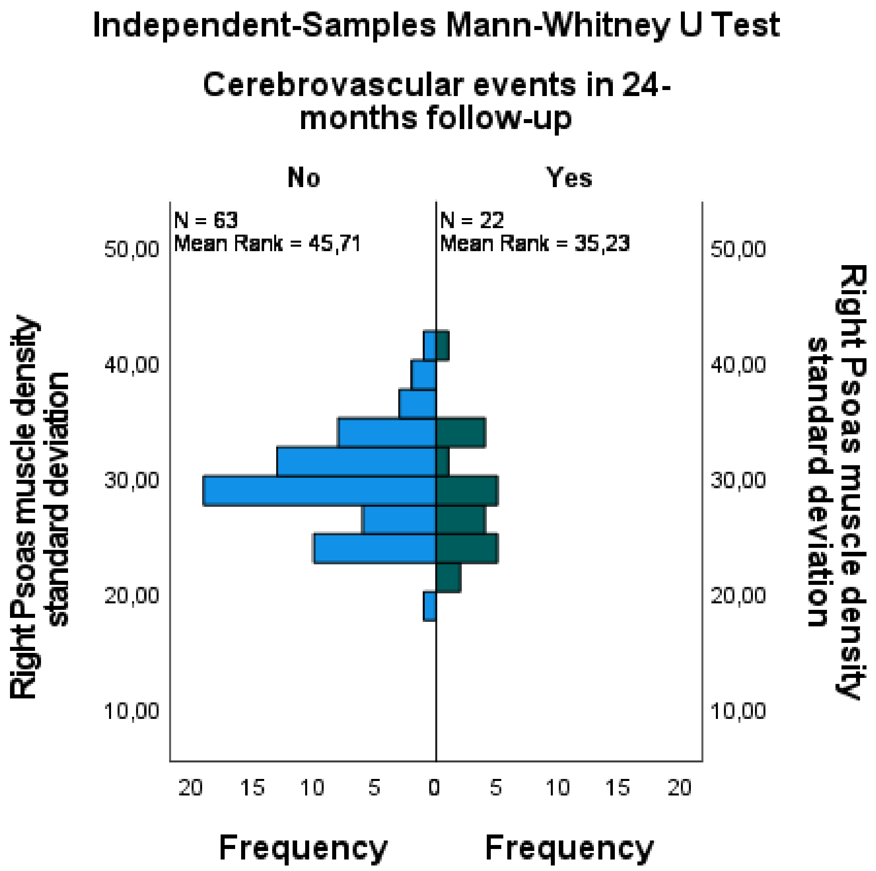

Left Psoas muscle mean density (HU) across Cerebrovascular events in 24-months follow-up

Independent-Samples Mann-Whitney U Test Summary

Total N	85
Mann-Whitney U	554,500
Wilcoxon W	807,500
Test Statistic	554,500
Standard Error	99,643
Standardized Test Statistic	-1,390
Asymptotic Sig.(2-sided test)	,165

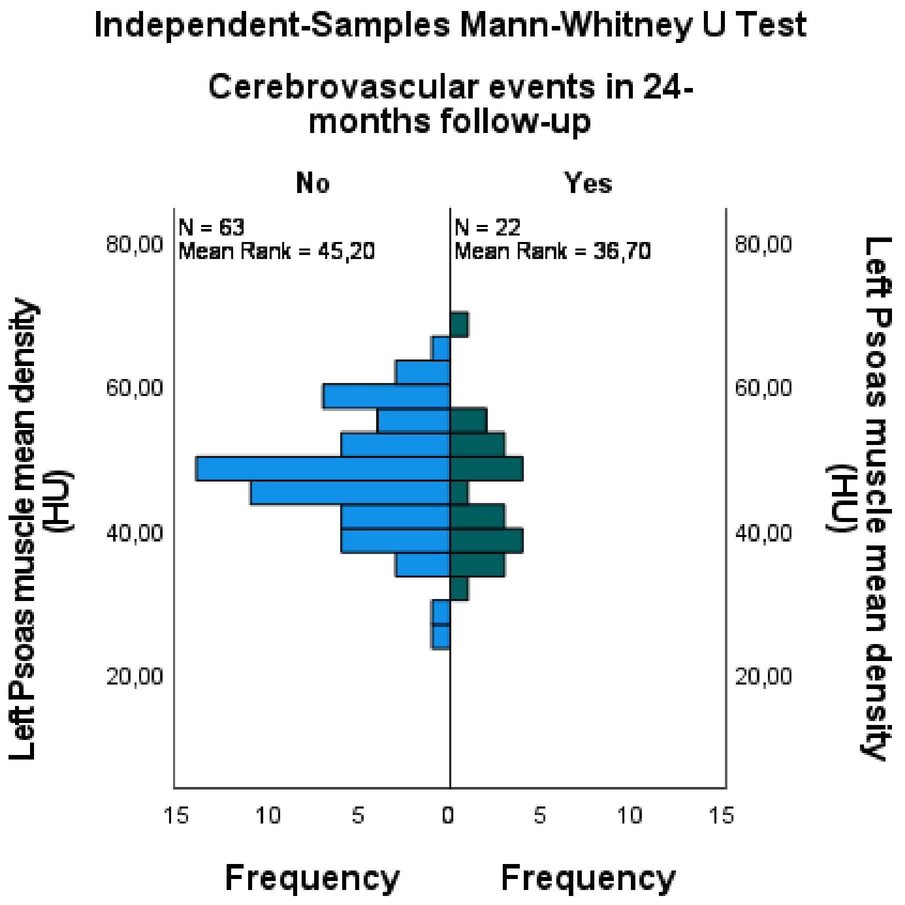

Left Psoas muscle median density (HU) across Cerebrovascular events in 24-months follow-up

Independent-Samples Mann-Whitney U Test Summary

Total N	85
Mann-Whitney U	574,500
Wilcoxon W	827,500
Test Statistic	574,500
Standard Error	99,537
Standardized Test Statistic	-1,191
Asymptotic Sig.(2-sided test)	,234

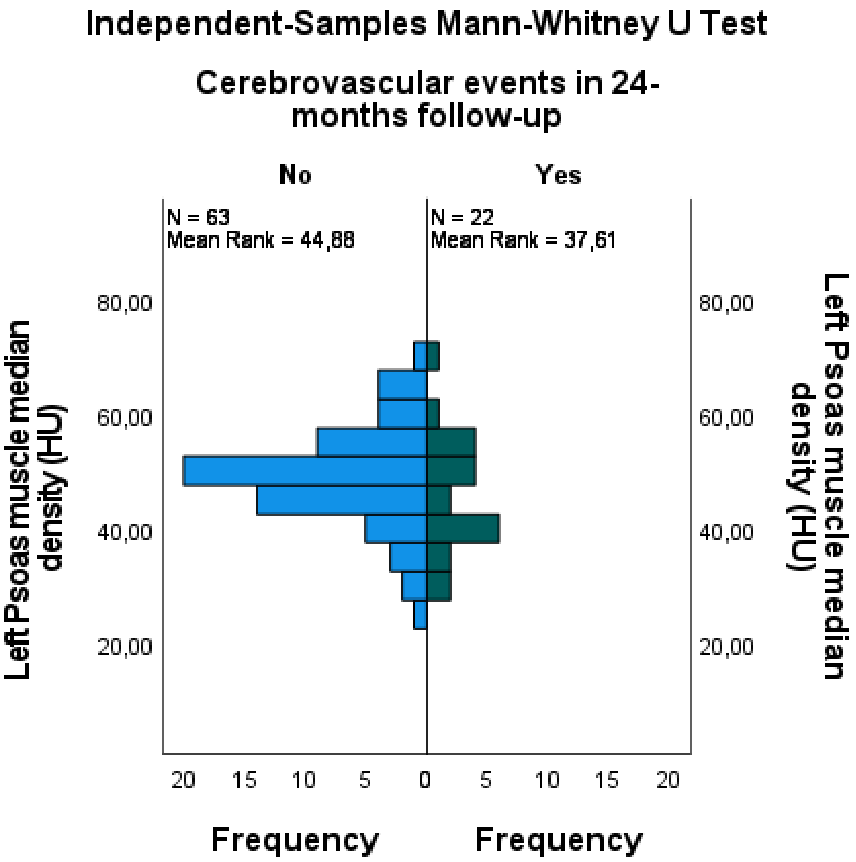

Left Psoas muscle density standard deviation across Cerebrovascular events in 24-months follow-up

Independent-Samples Mann-Whitney U Test Summary

Total N	85
Mann-Whitney U	588,000
Wilcoxon W	841,000
Test Statistic	588,000
Standard Error	99,664
Standardized Test Statistic	-1,054
Asymptotic Sig.(2-sided test)	,292

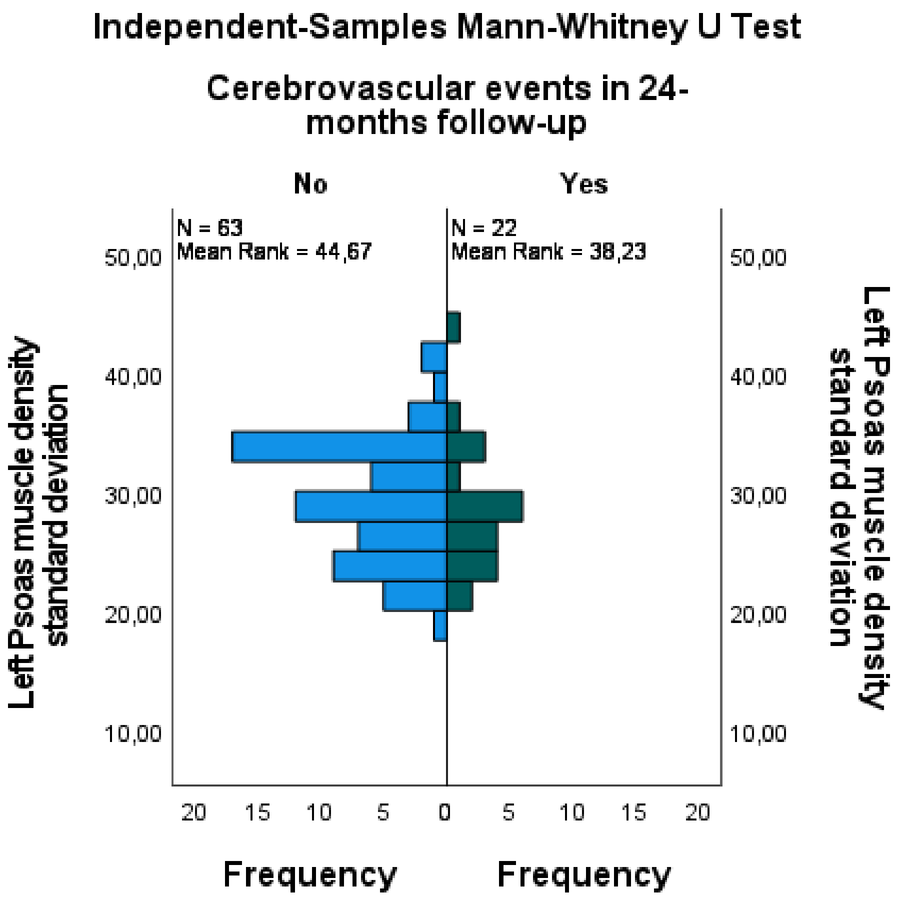

Mortality in 24-months follow-up con altre variabili in verde

Hypothesis Test Summary

	Null Hypothesis	Test	Sig.[a,b]	Decision
1	The distribution of Psoas/height is the same across categories of Mortality in 24-months follow-up.	Independent-Samples Mann-Whitney U Test	,008	Reject the null hypothesis.
2	The distribution of Anterior SAT distance is the same across categories of Mortality in 24-months follow-up.	Independent-Samples Mann-Whitney U Test	<,001	Reject the null hypothesis.
3	The distribution of Posterior SAT distance is the same across categories of Mortality in 24-months follow-up.	Independent-Samples Mann-Whitney U Test	<,001	Reject the null hypothesis.
4	The distribution of Anterior+Posterior SAT distance is the same across categories of Mortality in 24-months follow-up.	Independent-Samples Mann-Whitney U Test	<,001	Reject the null hypothesis.
5	The distribution of VAT distance is the same across categories of Mortality in 24-months follow-up.	Independent-Samples Mann-Whitney U Test	<,001	Reject the null hypothesis.
6	The distribution of Right common femoral artery area (mm2) is the same across categories of Mortality in 24-months follow-up.	Independent-Samples Mann-Whitney U Test	,058	Retain the null hypothesis.
7	The distribution of Left common femoral artery area (mm2) is the same across categories of Mortality in 24-months follow-up.	Independent-Samples Mann-Whitney U Test	,143	Retain the null hypothesis.

8	The distribution of FAT area (cm2) is the same across categories of Mortality in 24-months follow-up.	Independent-Samples Mann-Whitney U Test	<,001	Reject the null hypothesis.
9	The distribution of SAT area (cm2) is the same across categories of Mortality in 24-months follow-up.	Independent-Samples Mann-Whitney U Test	<,001	Reject the null hypothesis.
10	The distribution of VAT area (cm2) is the same across categories of Mortality in 24-months follow-up.	Independent-Samples Mann-Whitney U Test	,003	Reject the null hypothesis.
11	The distribution of Right Psoas muscle area (cm2) is the same across categories of Mortality in 24-months follow-up.	Independent-Samples Mann-Whitney U Test	,178	Retain the null hypothesis.
12	The distribution of Left Psoas muscle area (cm2) is the same across categories of Mortality in 24-months follow-up.	Independent-Samples Mann-Whitney U Test	,210	Retain the null hypothesis.
13	The distribution of FAT mean density (HU) is the same across categories of Mortality in 24-months follow-up.	Independent-Samples Mann-Whitney U Test	,106	Retain the null hypothesis.
14	The distribution of FAT median density (HU) is the same across categories of Mortality in 24-months follow-up.	Independent-Samples Mann-Whitney U Test	,099	Retain the null hypothesis.
15	The distribution of FAT density standard deviation is the same across categories of Mortality in 24-months follow-up.	Independent-Samples Mann-Whitney U Test	,073	Retain the null hypothesis.
16	The distribution of SAT mean density (HU) is the same across categories of Mortality in 24-months follow-up.	Independent-Samples Mann-Whitney U Test	,127	Retain the null hypothesis.
17	The distribution of SAT median density (HU) is the same across categories of Mortality in 24-months follow-up.	Independent-Samples Mann-Whitney U Test	,368	Retain the null hypothesis.

18	The distribution of SAT density standard deviation is the same across categories of Mortality in 24-months follow-up.	Independent-Samples Mann-Whitney U Test	,021	Reject the null hypothesis.
19	The distribution of VAT mean density (HU) is the same across categories of Mortality in 24-months follow-up.	Independent-Samples Mann-Whitney U Test	,084	Retain the null hypothesis.
20	The distribution of VAT median density (HU) is the same across categories of Mortality in 24-months follow-up.	Independent-Samples Mann-Whitney U Test	,105	Retain the null hypothesis.
21	The distribution of VAT density standard deviation is the same across categories of Mortality in 24-months follow-up.	Independent-Samples Mann-Whitney U Test	,769	Retain the null hypothesis.
22	The distribution of Right Psoas muscle mean density (HU) is the same across categories of Mortality in 24-months follow-up.	Independent-Samples Mann-Whitney U Test	,775	Retain the null hypothesis.
23	The distribution of Right Psoas muscle median density (HU) is the same across categories of Mortality in 24-months follow-up.	Independent-Samples Mann-Whitney U Test	,354	Retain the null hypothesis.
24	The distribution of Right Psoas muscle density standard deviation is the same across categories of Mortality in 24-months follow-up.	Independent-Samples Mann-Whitney U Test	,004	Reject the null hypothesis.
25	The distribution of Left Psoas muscle mean density (HU) is the same across categories of Mortality in 24-months follow-up.	Independent-Samples Mann-Whitney U Test	,591	Retain the null hypothesis.
26	The distribution of Left Psoas muscle median density (HU) is the same across categories of Mortality in 24-months follow-up.	Independent-Samples Mann-Whitney U Test	,974	Retain the null hypothesis.

27	The distribution of Left Psoas muscle density standard deviation is the same across categories of Mortality in 24-months follow-up.	Independent-Samples Mann-Whitney U Test	,002	Reject the null hypothesis.

a. The significance level is ,050.

b. Asymptotic significance is displayed.

In this case, the hypothesis of equal medians ($p <0.05$) is rejected for the variables Psoas/height, Anterior SAT distance, Posterior SAT distance, Anterior + Posterior SAT distance, VAT distance, FAT area (cm2), SAT area (cm2), VAT area (cm2), Right Psoas muscle density standard deviation, SAT density standard deviation and Left Psoas muscle density standard deviation, while for the rest we accept the null hypothesis ($p> 0.05$).

(The tables and graphs below are the details of the tests in this table: I have highlighted what things you should eventually report, namely test statistic and pvalue).

Psoas/height across Mortality in 24-months follow-up

Independent-Samples Mann-Whitney U Test Summary

Total N	85
Mann-Whitney U	516,000
Wilcoxon W	922,000
Test Statistic	516,000
Standard Error	106,948
Standardized Test Statistic	-2,637
Asymptotic Sig.(2-sided test)	,008

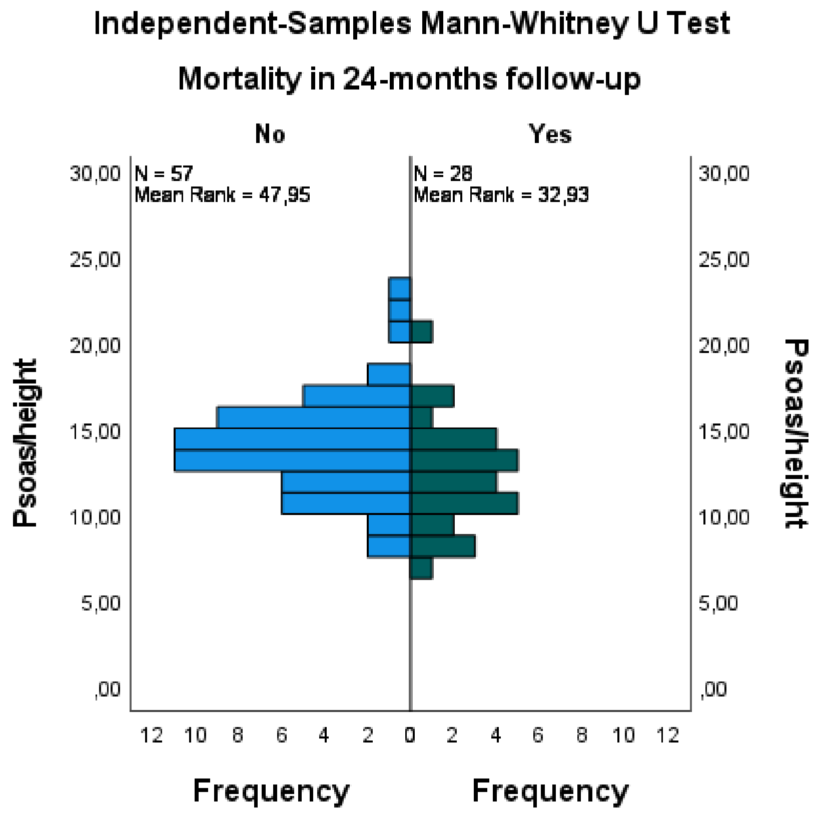

Anterior SAT distance across Mortality in 24-months follow-up

Independent-Samples Mann-Whitney U Test Summary

Total N	85
Mann-Whitney U	291,000
Wilcoxon W	697,000
Test Statistic	291,000
Standard Error	106,933
Standardized Test Statistic	-4,741
Asymptotic Sig.(2-sided test)	<,001

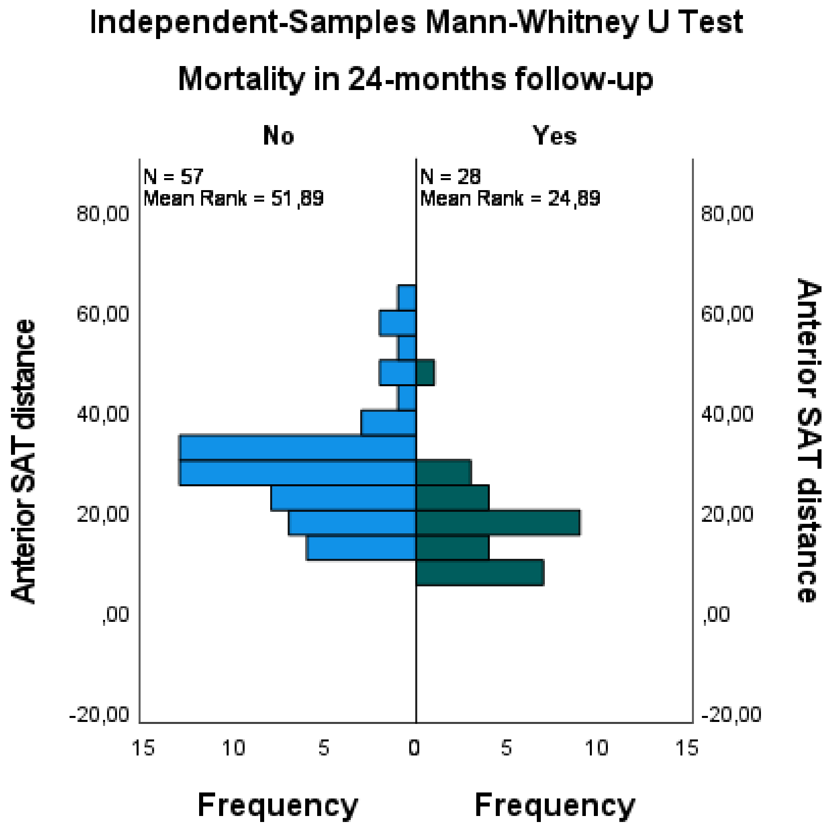

Posterior SAT distance across Mortality in 24-months follow-up

Independent-Samples Mann-Whitney U Test Summary

Total N	85
Mann-Whitney U	331,500
Wilcoxon W	737,500
Test Statistic	331,500
Standard Error	106,937
Standardized Test Statistic	-4,362
Asymptotic Sig.(2-sided test)	<,001

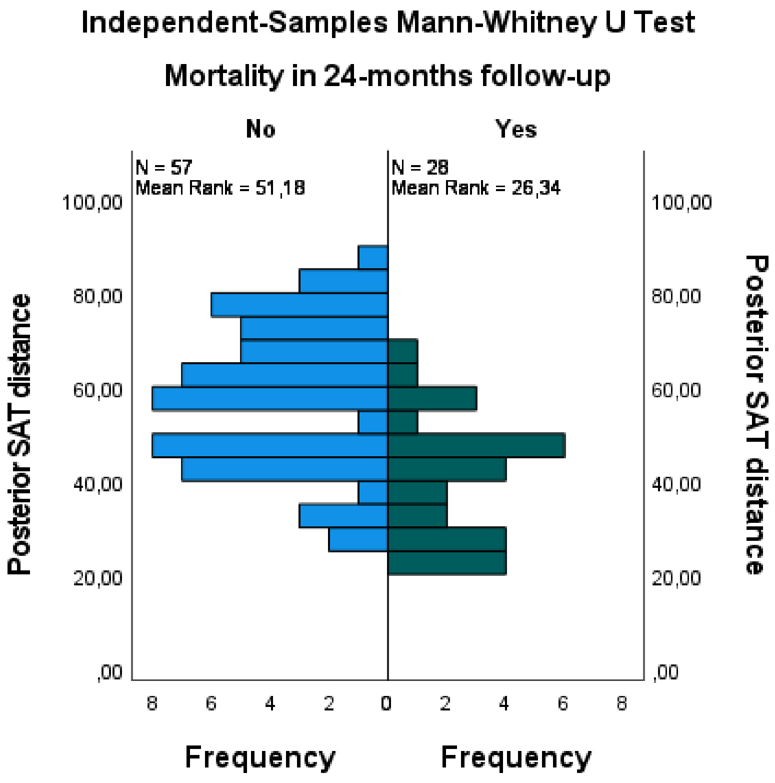

Anterior+Posterior SAT distance across Mortality in 24-months follow-up

Independent-Samples Mann-Whitney U Test Summary

Total N	85
Mann-Whitney U	262,000
Wilcoxon W	668,000
Test Statistic	262,000
Standard Error	106,944
Standardized Test Statistic	-5,012
Asymptotic Sig.(2-sided test)	<,001

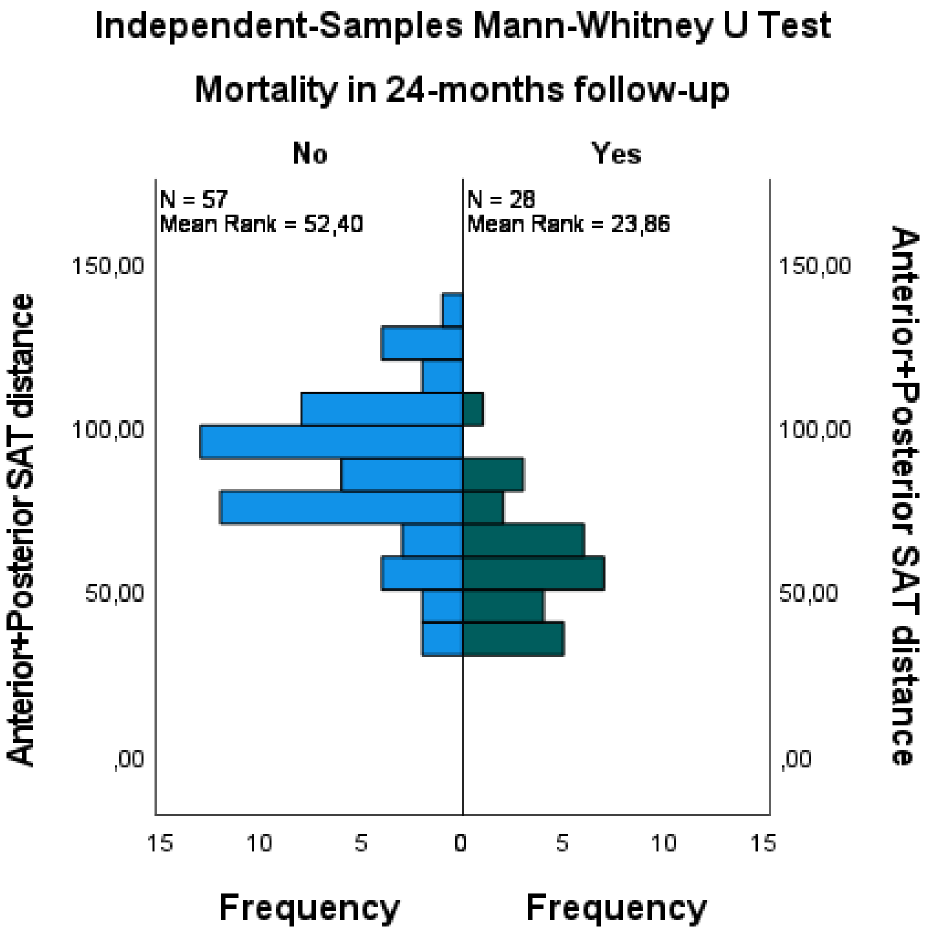

VAT distance across Mortality in 24-months follow-up

Independent-Samples Mann-Whitney U Test Summary

Total N	84
Mann-Whitney U	428,500
Wilcoxon W	834,500
Test Statistic	428,500
Standard Error	105,367
Standardized Test Statistic	-3,374
Asymptotic Sig.(2-sided test)	<,001

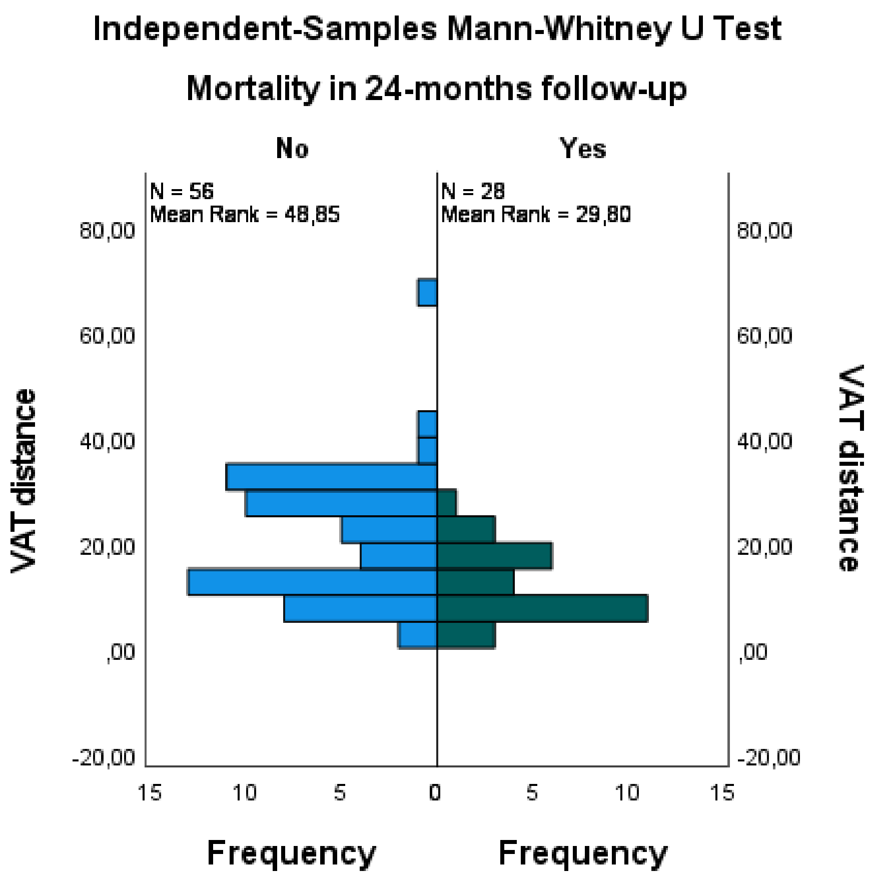

Right common femoral artery area (mm2) across Mortality in 24-months follow-up

Independent-Samples Mann-Whitney U Test Summary

Total N	85
Mann-Whitney U	1000,500
Wilcoxon W	1406,500
Test Statistic	1000,500
Standard Error	106,931
Standardized Test Statistic	1,894
Asymptotic Sig.(2-sided test)	,058

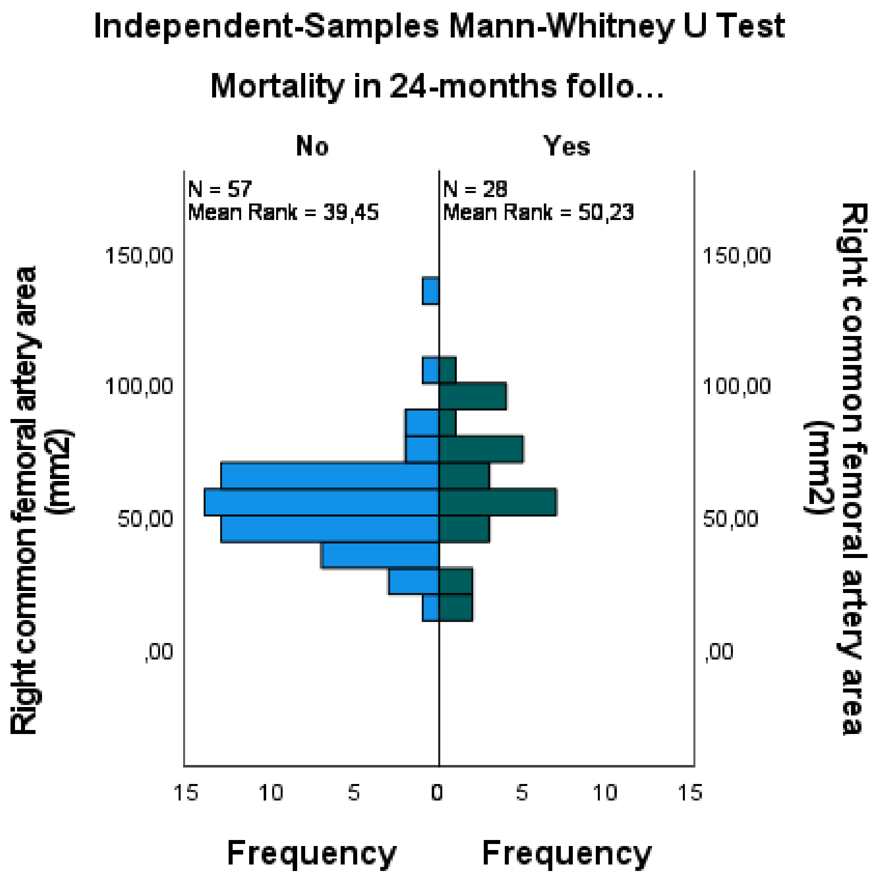

Left common femoral artery area (mm2) across Mortality in 24-months follow-up

Independent-Samples Mann-Whitney U Test Summary

Total N	85
Mann-Whitney U	954,500
Wilcoxon W	1360,500
Test Statistic	954,500
Standard Error	106,922
Standardized Test Statistic	1,464
Asymptotic Sig.(2-sided test)	,143

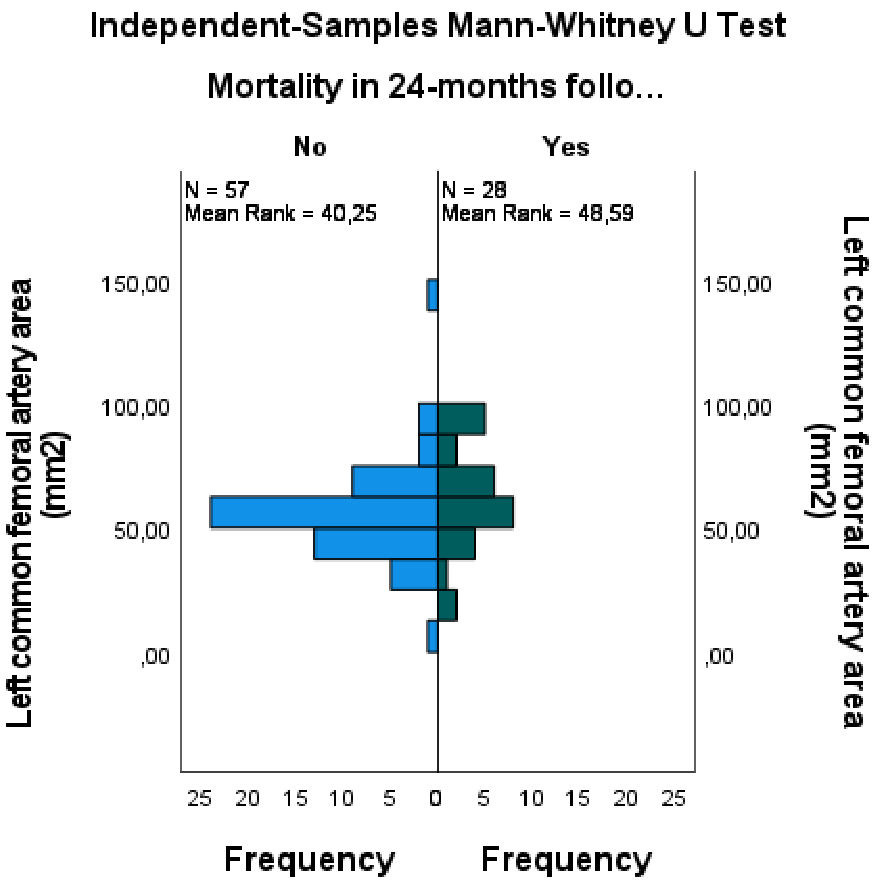

FAT area (cm2) across Mortality in 24-months follow-up

Independent-Samples Mann-Whitney U Test Summary

Total N	85
Mann-Whitney U	357,000
Wilcoxon W	763,000
Test Statistic	357,000
Standard Error	106,948
Standardized Test Statistic	-4,123

Asymptotic Sig.(2-sided test)	<,001

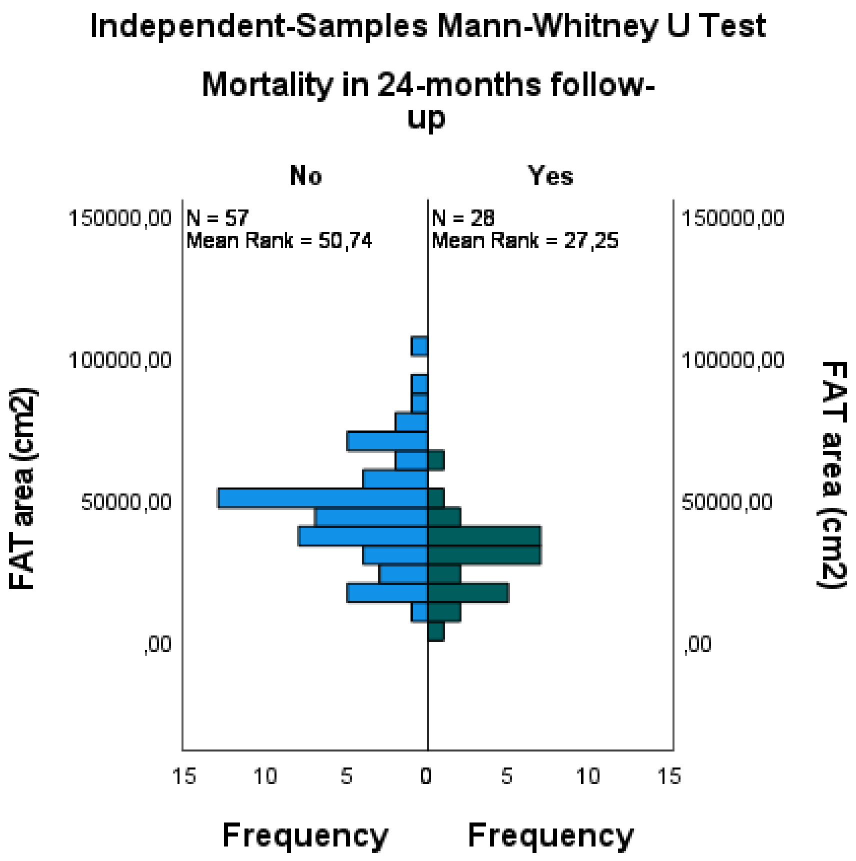

SAT area (cm2) across Mortality in 24-months follow-up

Independent-Samples Mann-Whitney U Test Summary

Total N	85
Mann-Whitney U	329,000
Wilcoxon W	735,000
Test Statistic	329,000
Standard Error	106,948
Standardized Test Statistic	-4,385

Asymptotic Sig.(2-sided test)	<,001

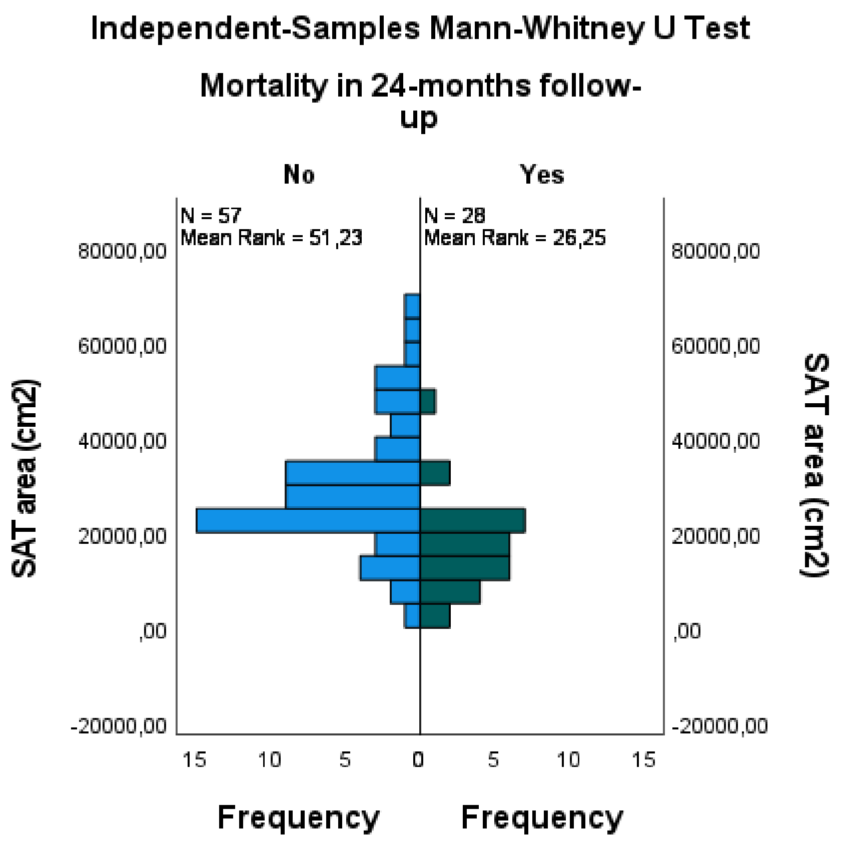

VAT area (cm2) across Mortality in 24-months follow-up

Independent-Samples Mann-Whitney U Test Summary

Total N	84
Mann-Whitney U	469,000
Wilcoxon W	875,000
Test Statistic	469,000
Standard Error	105,388

Standardized Test Statistic	-2,989
Asymptotic Sig.(2-sided test)	,003

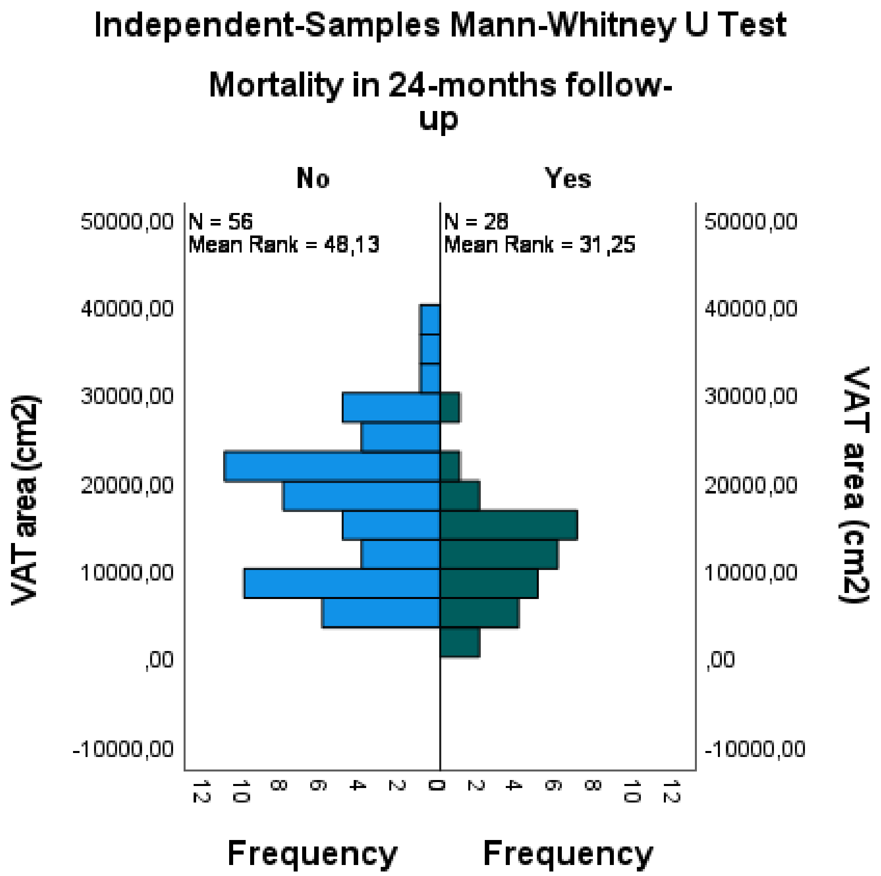

Right Psoas muscle area (cm2) across Mortality in 24-months follow-up

Independent-Samples Mann-Whitney U Test Summary

Total N	85
Mann-Whitney U	654,000
Wilcoxon W	1060,000
Test Statistic	654,000

Standard Error	106,945
Standardized Test Statistic	-1,346
Asymptotic Sig.(2-sided test)	,178

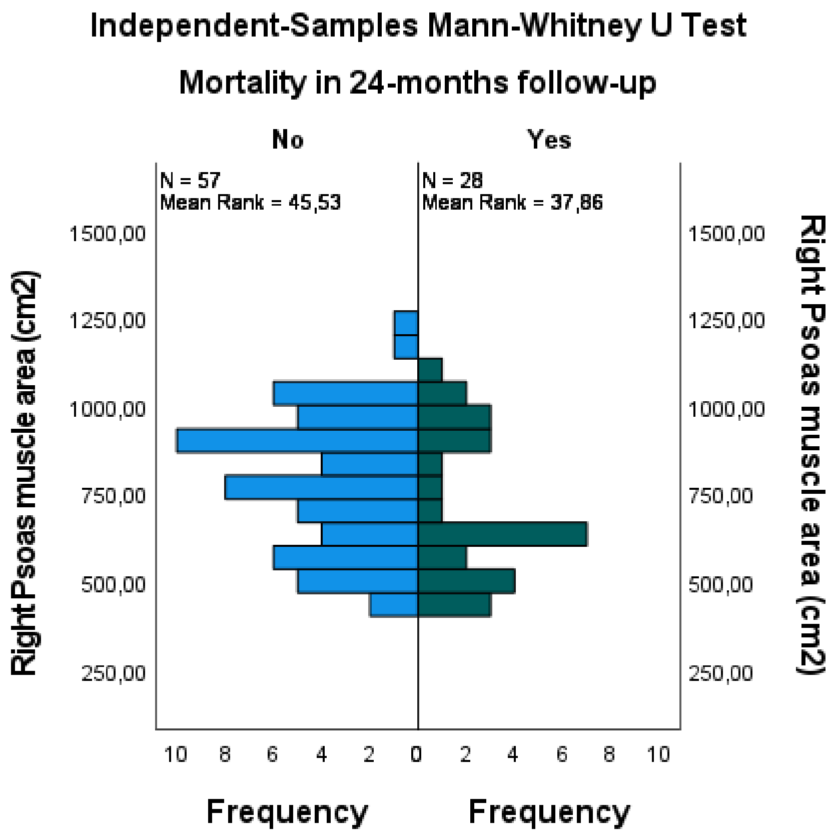

Left Psoas muscle area (cm2) across Mortality in 24-months follow-up

Independent-Samples Mann-Whitney U Test Summary

Total N	85
Mann-Whitney U	664,000
Wilcoxon W	1070,000

Test Statistic	664,000
Standard Error	106,948
Standardized Test Statistic	-1,253
Asymptotic Sig.(2-sided test)	,210

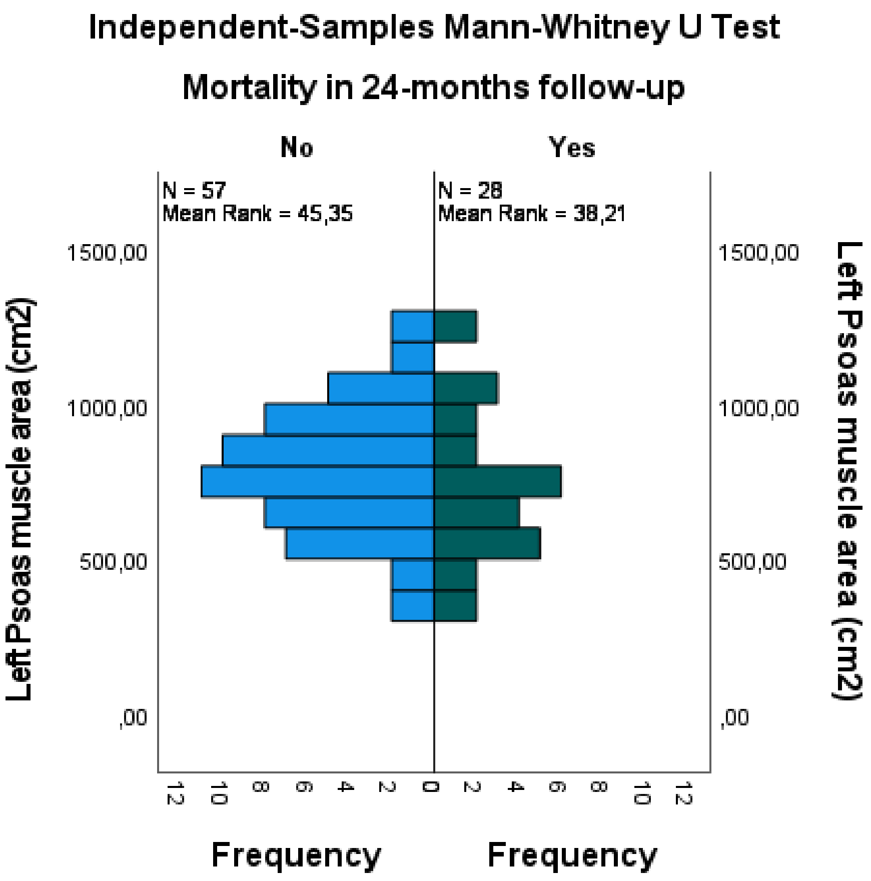

FAT mean density (HU) across Mortality in 24-months follow-up

Independent-Samples Mann-Whitney U Test Summary

Total N	85
Mann-Whitney U	971,000

Wilcoxon W	1377,000
Test Statistic	971,000
Standard Error	106,931
Standardized Test Statistic	1,618
Asymptotic Sig.(2-sided test)	,106

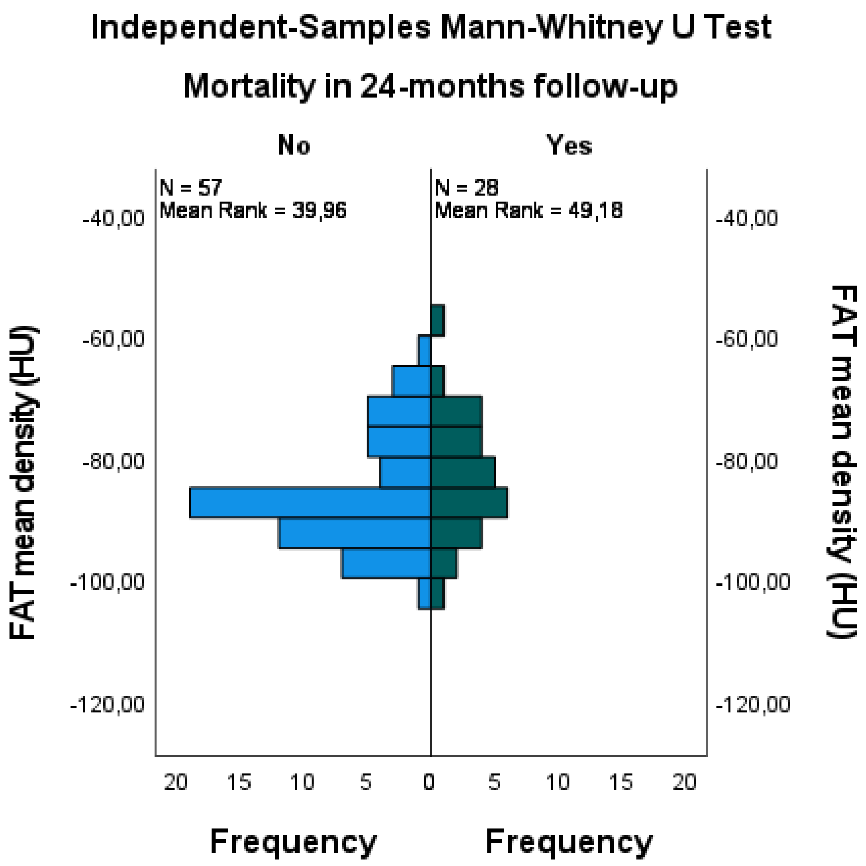

FAT median density (HU) across Mortality in 24-months follow-up

Independent-Samples Mann-Whitney U Test Summary

Total N	85
Mann-Whitney U	974,000
Wilcoxon W	1380,000
Test Statistic	974,000
Standard Error	106,819
Standardized Test Statistic	1,648
Asymptotic Sig.(2-sided test)	,099

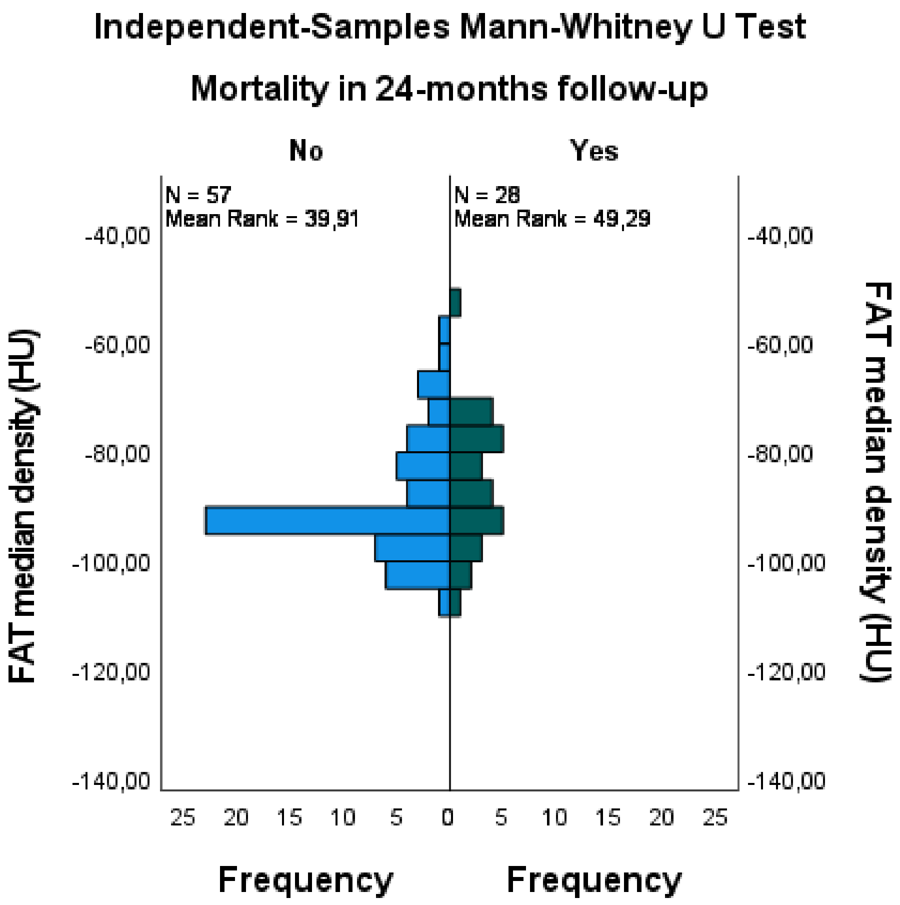

FAT density standard deviation across Mortality in 24-months follow-up

Independent-Samples Mann-Whitney U Test Summary

Total N	85
Mann-Whitney U	606,000
Wilcoxon W	1012,000
Test Statistic	606,000
Standard Error	106,948
Standardized Test Statistic	-1,795
Asymptotic Sig.(2-sided test)	,073

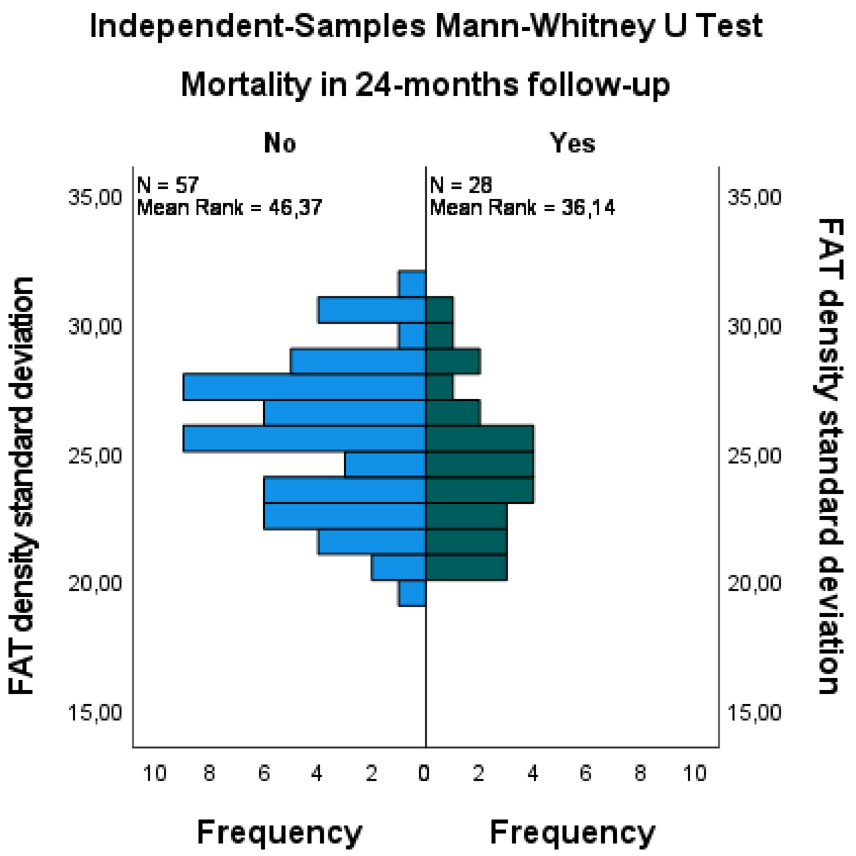

SAT mean density (HU) across Mortality in 24-months follow-up

Independent-Samples Mann-Whitney U Test Summary

Total N	85
Mann-Whitney U	961,000
Wilcoxon W	1367,000
Test Statistic	961,000
Standard Error	106,936
Standardized Test Statistic	1,524
Asymptotic Sig.(2-sided test)	,127

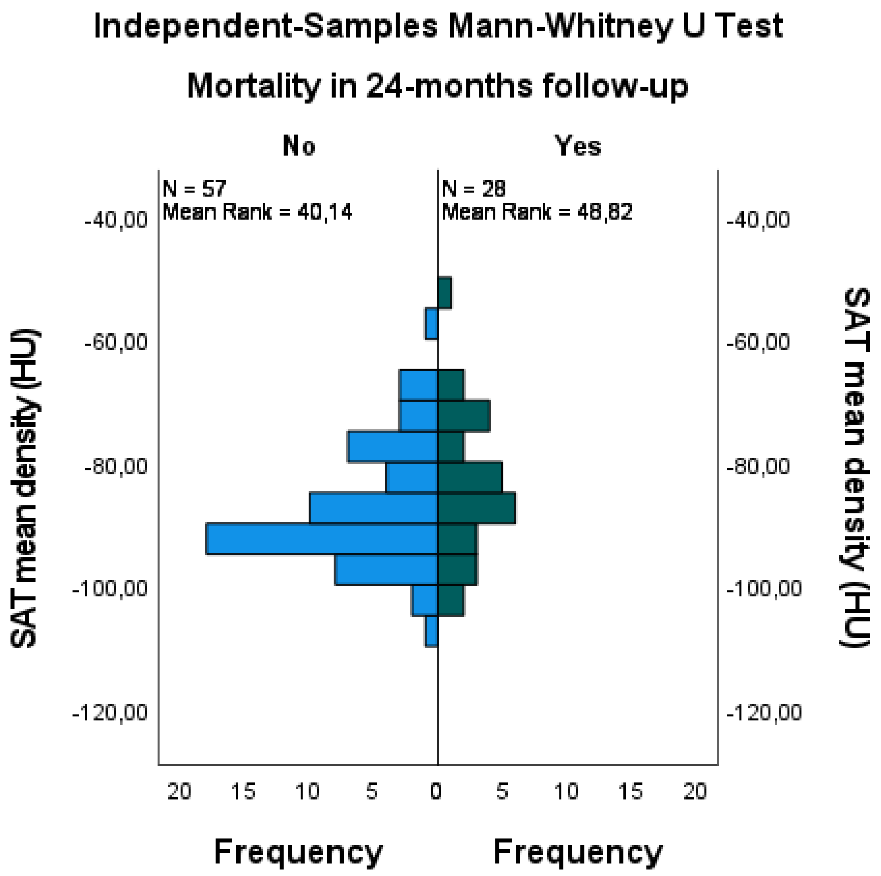

SAT median density (HU) across Mortality in 24-months follow-up

Independent-Samples Mann-Whitney U Test Summary

Total N	83
Mann-Whitney U	848,500
Wilcoxon W	1226,500
Test Statistic	848,500
Standard Error	102,799
Standardized Test Statistic	,900
Asymptotic Sig.(2-sided test)	,368

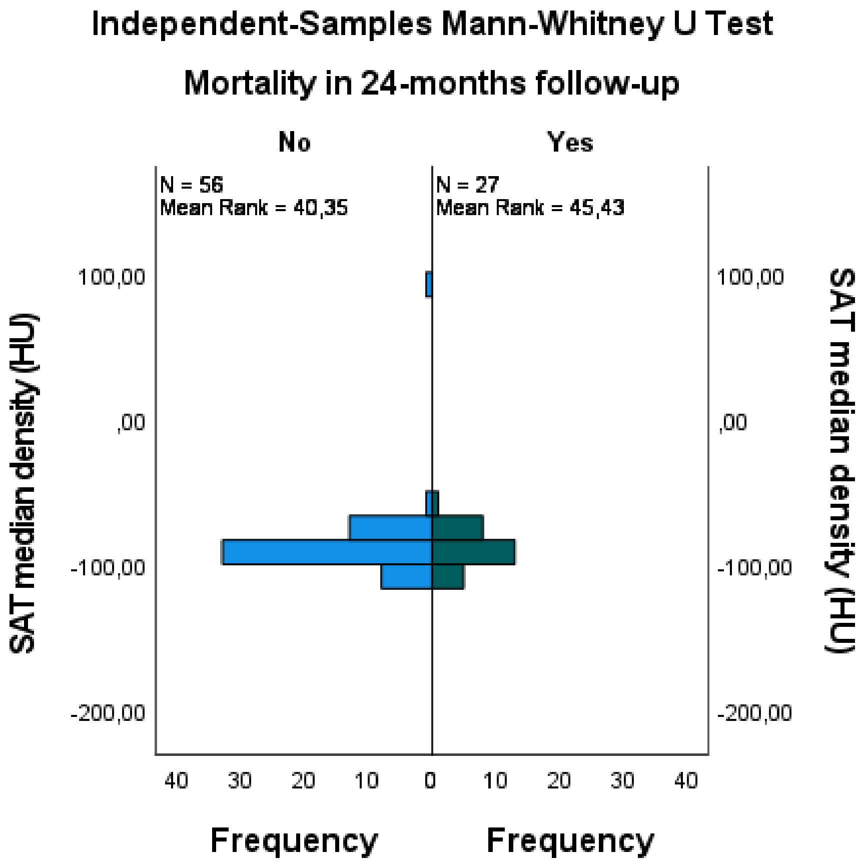

SAT density standard deviation across Mortality in 24-months follow-up

Independent-Samples Mann-Whitney U Test Summary

Total N	84
Mann-Whitney U	541,000
Wilcoxon W	947,000
Test Statistic	541,000
Standard Error	105,388
Standardized Test Statistic	-2,306
Asymptotic Sig.(2-sided test)	,021

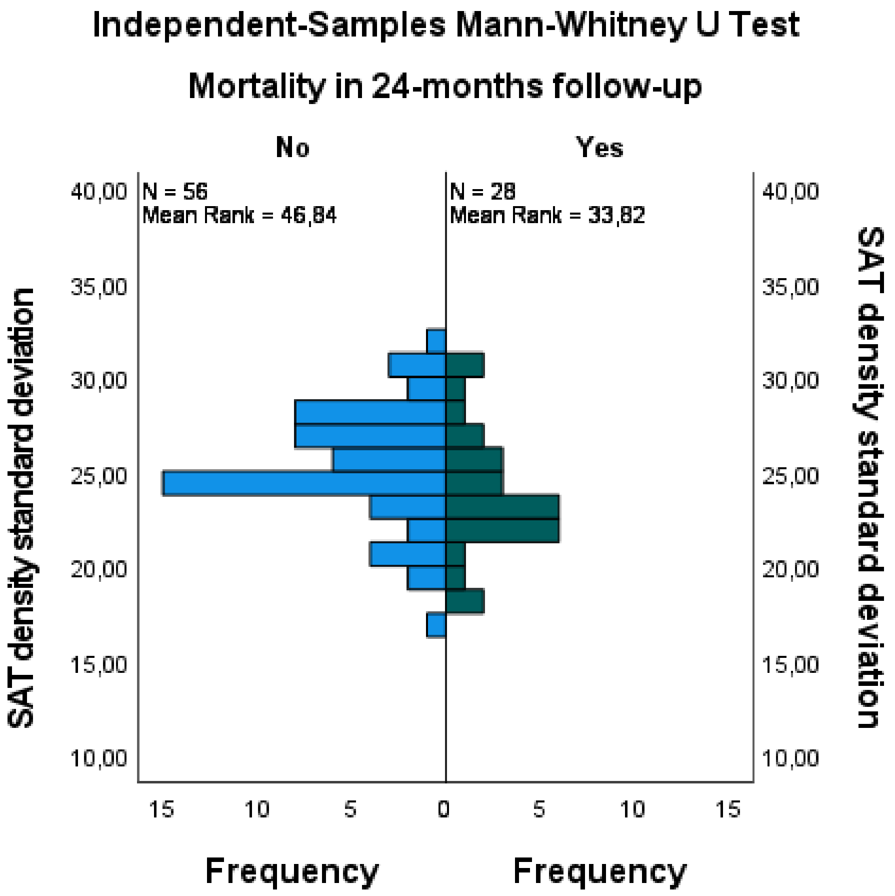

VAT mean density (HU) across Mortality in 24-months follow-up

Independent-Samples Mann-Whitney U Test Summary

Total N	85
Mann-Whitney U	982,500
Wilcoxon W	1388,500
Test Statistic	982,500
Standard Error	106,929
Standardized Test Statistic	1,725
Asymptotic Sig.(2-sided test)	,084

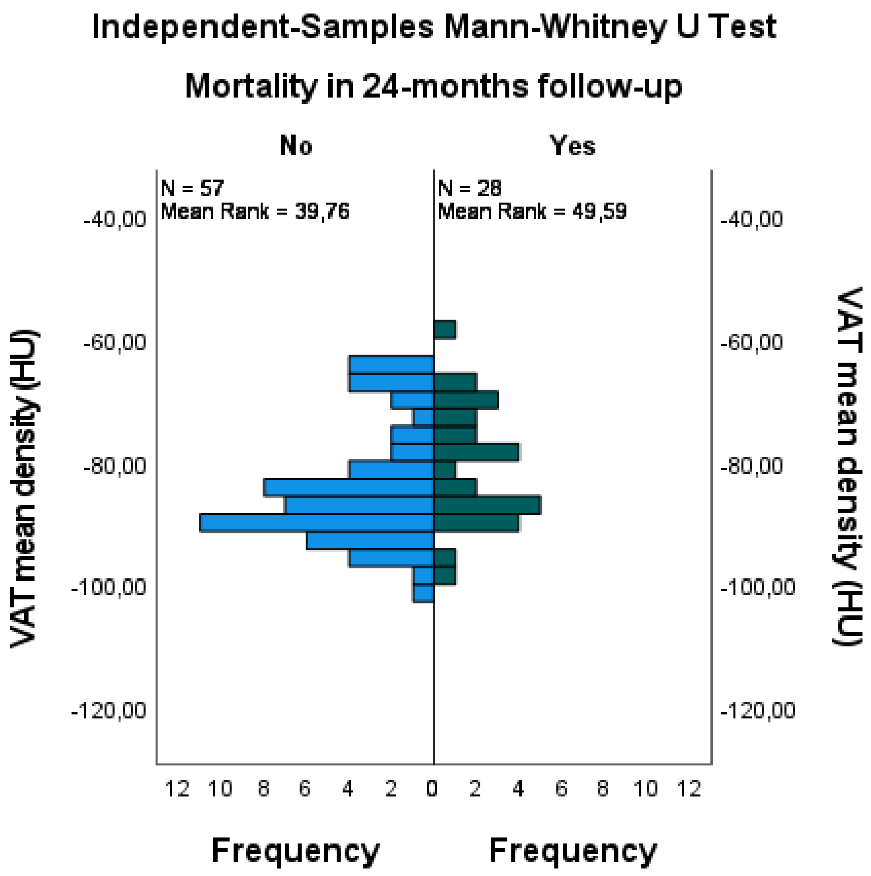

VAT median density (HU) across Mortality in 24-months follow-up

Independent-Samples Mann-Whitney U Test Summary

Total N	85
Mann-Whitney U	971,000
Wilcoxon W	1377,000
Test Statistic	971,000
Standard Error	106,815
Standardized Test Statistic	1,620
Asymptotic Sig.(2-sided test)	,105

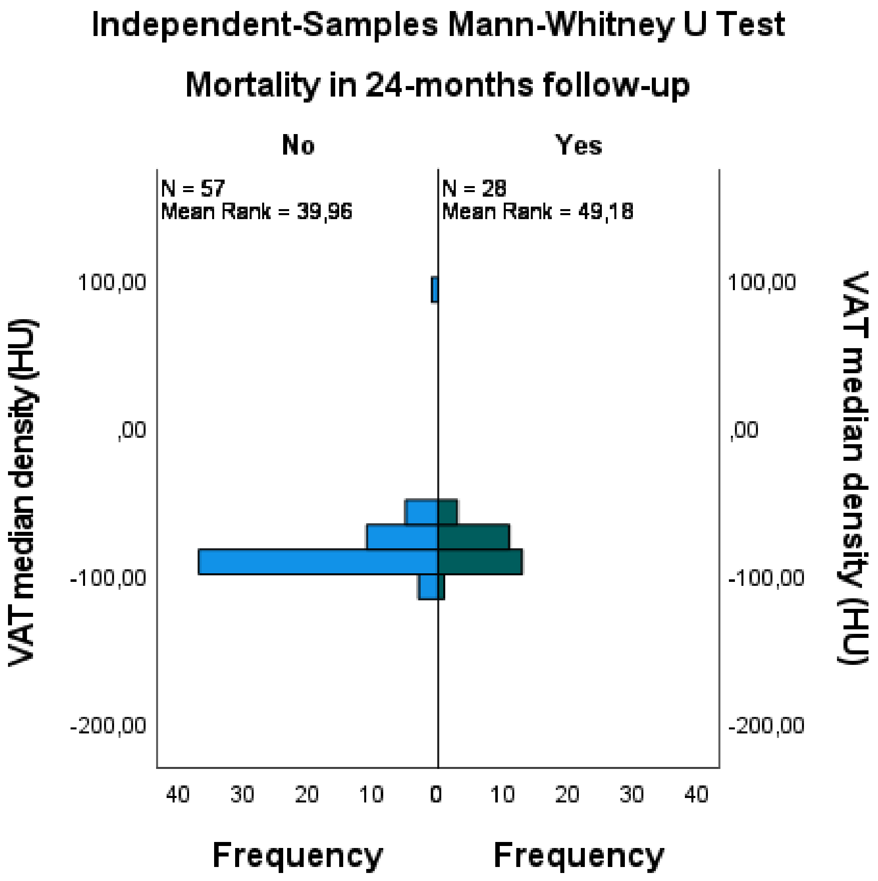

VAT density standard deviation across Mortality in 24-months follow-up

Independent-Samples Mann-Whitney U Test Summary

Total N	84
Mann-Whitney U	815,000
Wilcoxon W	1221,000
Test Statistic	815,000
Standard Error	105,388
Standardized Test Statistic	,294
Asymptotic Sig.(2-sided test)	,769

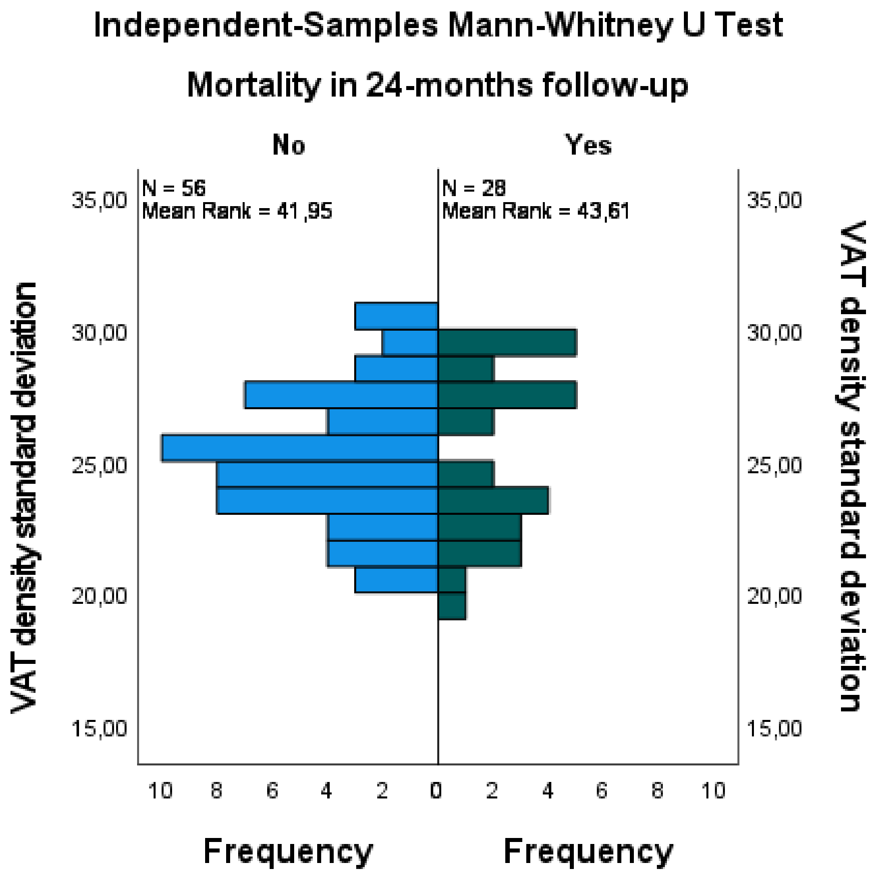

Right Psoas muscle mean density (HU) across Mortality in 24-months follow-up

Independent-Samples Mann-Whitney U Test Summary

Total N	85
Mann-Whitney U	828,500
Wilcoxon W	1234,500
Test Statistic	828,500
Standard Error	106,925
Standardized Test Statistic	,285
Asymptotic Sig.(2-sided test)	,775

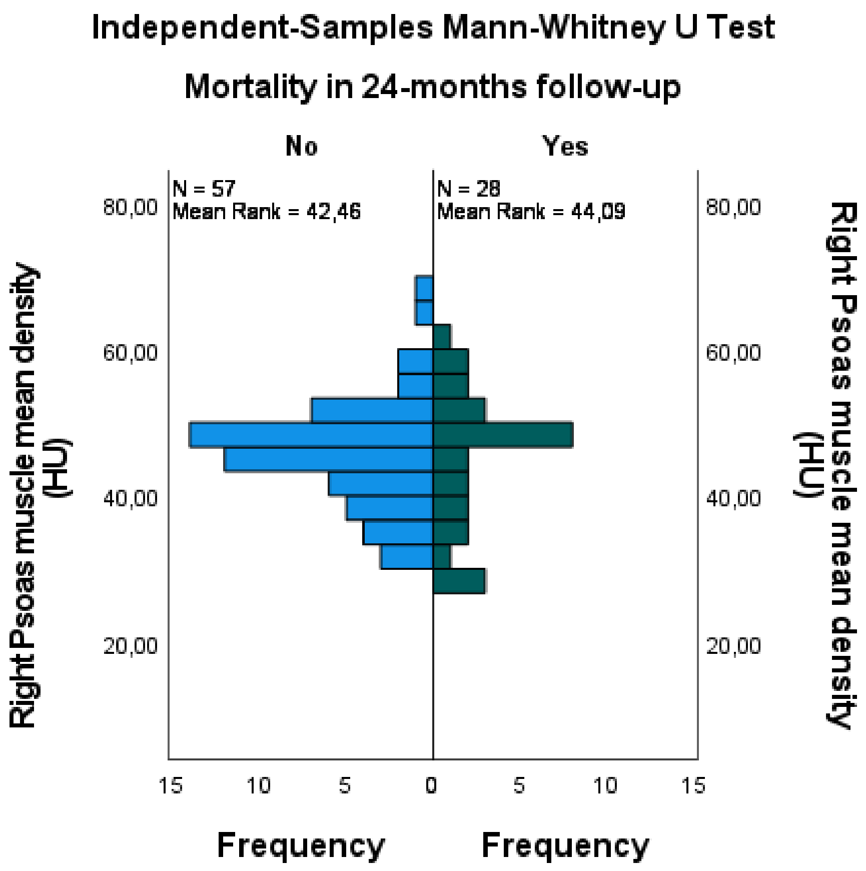

Right Psoas muscle median density (HU) across Mortality in 24-months follow-up

Independent-Samples Mann-Whitney U Test Summary

Total N	85
Mann-Whitney U	897,000
Wilcoxon W	1303,000
Test Statistic	897,000
Standard Error	106,847
Standardized Test Statistic	,927
Asymptotic Sig.(2-sided test)	,354

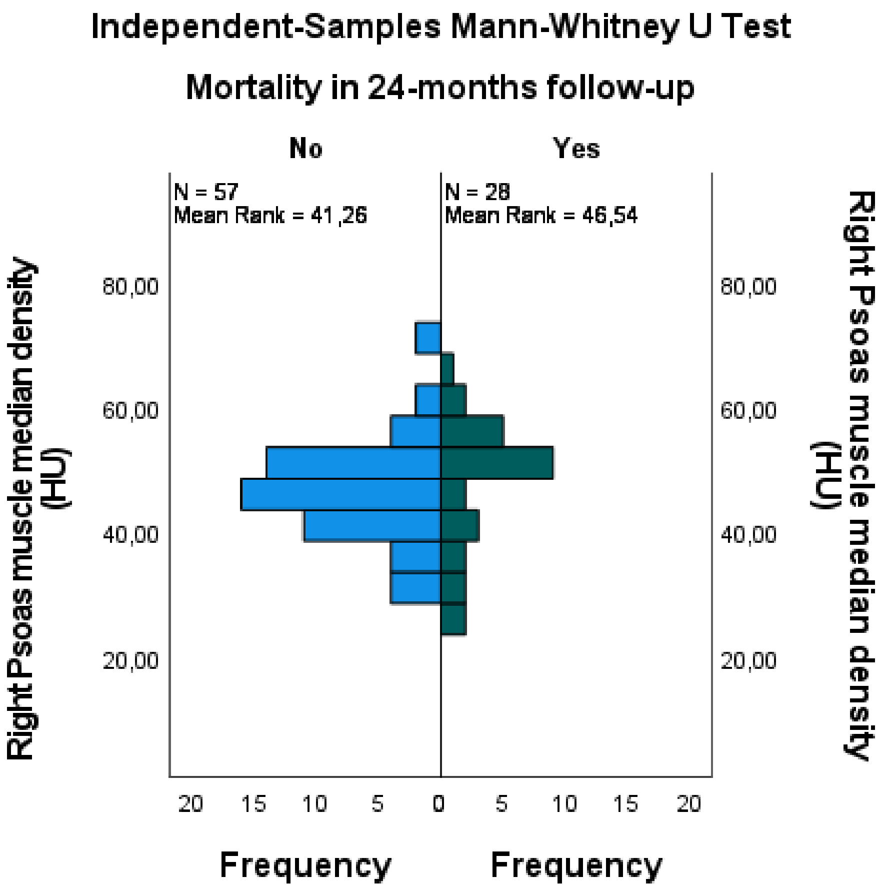

Right Psoas muscle density standard deviation across Mortality in 24-months follow-up

Independent-Samples Mann-Whitney U Test Summary

Total N	85
Mann-Whitney U	493,000
Wilcoxon W	899,000
Test Statistic	493,000
Standard Error	106,947
Standardized Test Statistic	-2,852
Asymptotic Sig.(2-sided test)	,004

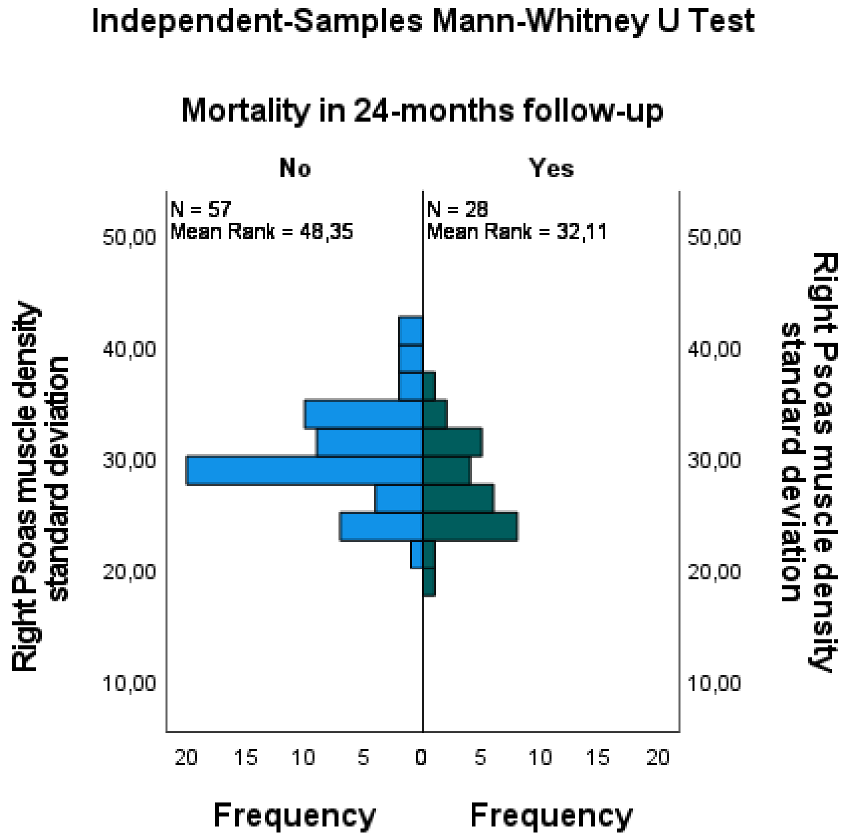

Left Psoas muscle mean density (HU) across Mortality in 24-months follow-up

Independent-Samples Mann-Whitney U Test Summary

Total N	85
Mann-Whitney U	740,500
Wilcoxon W	1146,500
Test Statistic	740,500
Standard Error	106,926
Standardized Test Statistic	-,538
Asymptotic Sig.(2-sided test)	,591

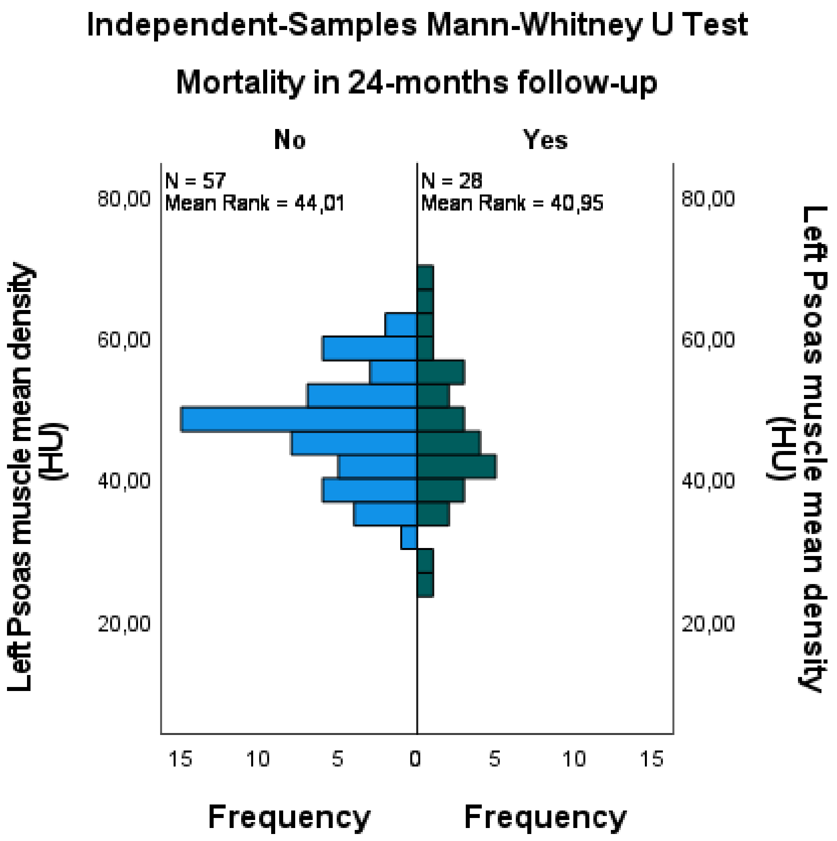

Left Psoas muscle median density (HU) across Mortality in 24-months follow-up

Independent-Samples Mann-Whitney U Test Summary

Total N	85
Mann-Whitney U	794,500
Wilcoxon W	1200,500
Test Statistic	794,500
Standard Error	106,812
Standardized Test Statistic	-,033
Asymptotic Sig.(2-sided test)	,974

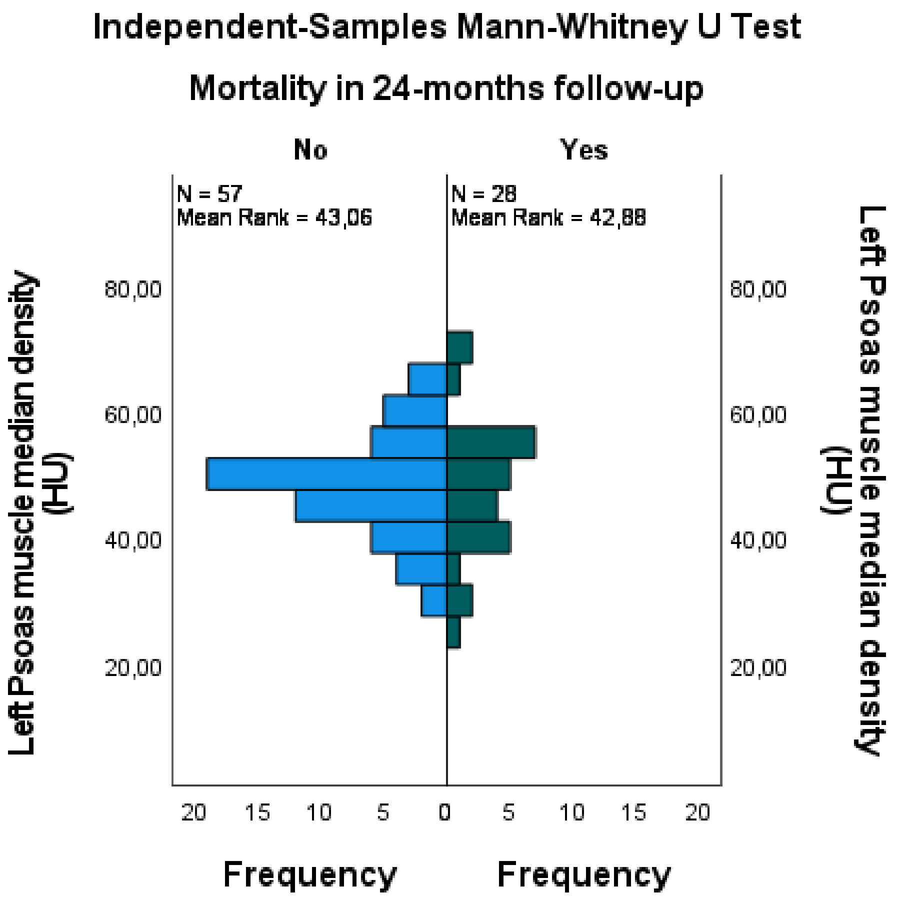

Left Psoas muscle density standard deviation across Mortality in 24-months follow-up

Independent-Samples Mann-Whitney U Test Summary

Total N	85
Mann-Whitney U	473,000
Wilcoxon W	879,000
Test Statistic	473,000
Standard Error	106,949
Standardized Test Statistic	-3,039

Asymptotic Sig.(2-sided test)	,002

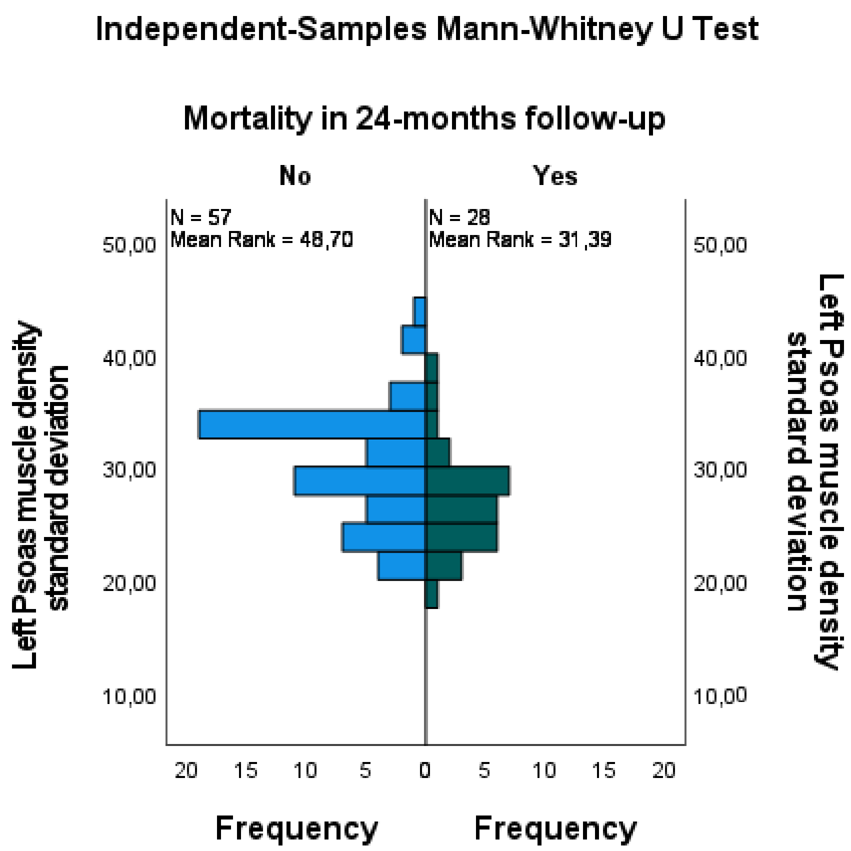

ROC Analysis

Cut-off SAT ant+post

- **Adverse cardiac events in 24-months follow-up**

Case Processing Summary

Adverse cardiac events in 24-months follow-up	Valid N (listwise)
Positive[a]	24
Negative	61

Smaller values of the test result variable(s) indicate stronger evidence for a positive actual state.

a. The positive actual state is Yes.

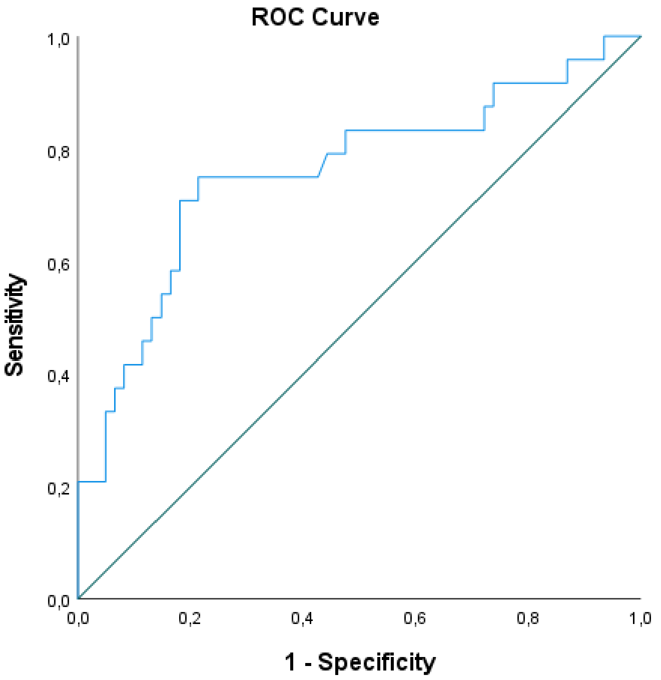

Diagonal segments are produced by ties.

Area Under the Curve

Test Result Variable(s): Anterior+Posterior SAT distance

Area	Std. Error[a]	Asymptotic Sig.[b]	Asymptotic 95% Confidence Interval	
			Lower Bound	Upper Bound
,759	,064	,000	,634	,884

The test result variable(s): Anterior+Posterior SAT distance has at least one tie between the positive actual state group and the negative actual state group. Statistics may be biased.

a. Under the nonparametric assumption

b. Null hypothesis: true area = 0.5

Youden Index: 1.537; cut-off: 68.00; sensibility: 0.750; specificity: 0.787

There is a significant result ($p < 0.05$) and the AUC is equal to 0.759, therefore the test is moderately accurate. The cut-off found indicates the value above which the 0's are expected.

- **Cerebrovascular events in 24-months follow-up**

Case Processing Summary

Cerebrovascular events in 24-months follow-up	Valid N (listwise)
Positive[a]	22
Negative	63

Smaller values of the test result variable(s) indicate stronger evidence for a positive actual state.

a. The positive actual state is Yes.

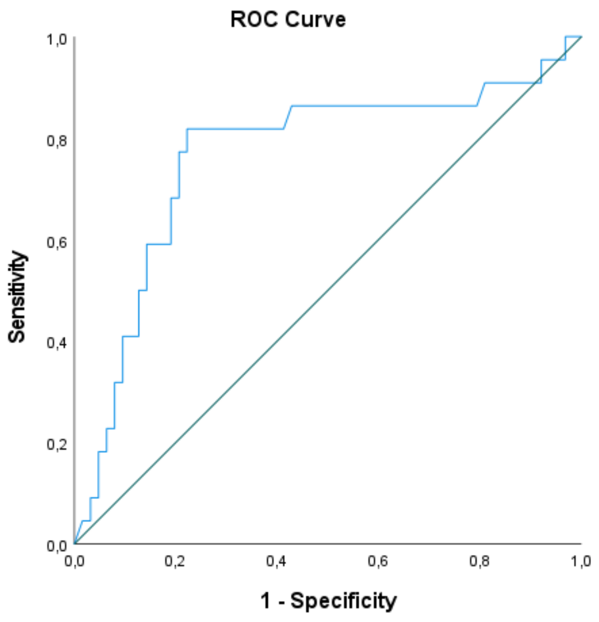

Diagonal segments are produced by ties.

Area Under the Curve

Test Result Variable(s): Anterior+Posterior SAT distance

Area	Std. Error[a]	Asymptotic Sig.[b]	Asymptotic 95% Confidence Interval	
			Lower Bound	Upper Bound
,763	,066	,000	,633	,893

The test result variable(s): Anterior+Posterior SAT distance has at least one tie between the positive actual state group and the negative actual state group. Statistics may be biased.

a. Under the nonparametric assumption

b. Null hypothesis: true area = 0.5

Youden Index: 1.596; cut-off: 69.05; sensibility: 0.818; specificity: 0.778

There is a significant result ($p < 0.05$) and the AUC is equal to 0.763, therefore the test is moderately accurate. The cut-off found indicates the value above which the 0's are expected.

- **Mortality in 24-months follow-up**

Case Processing Summary

Mortality in 24-months follow-up	Valid N (listwise)
Positive[a]	28
Negative	57

Smaller values of the test result variable(s) indicate stronger evidence for a positive actual state.

a. The positive actual state is Yes.

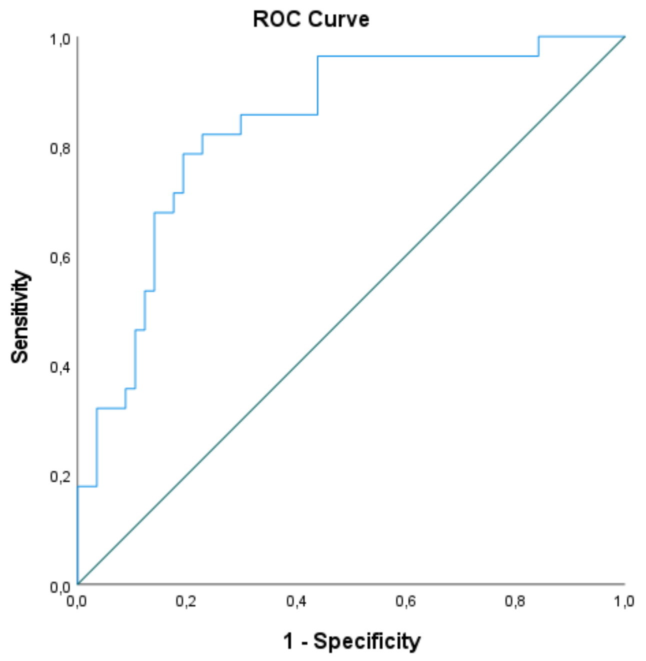

Area Under the Curve

Test Result Variable(s): Anterior+Posterior SAT distance

Area	Std. Error[a]	Asymptotic Sig.[b]	Asymptotic 95% Confidence Interval Lower Bound	Upper Bound
,836	,046	,000	,746	,925

a. Under the nonparametric assumption

b. Null hypothesis: true area = 0.5

Youden Index: 1.557; cut-off: 69.05; sensibility: 0.750; specificity: 0.807

There is a significant result ($p < 0.05$) and the AUC is equal to 0.836, therefore the test is moderately accurate. The cut-off found indicates the value above which the 0's are expected.

Cut-off VAT thickness

- Adverse cardiac events in 24-months follow-up

Case Processing Summary

Adverse cardiac events in 24-months follow-up	Valid N (listwise)
Positive[a]	23
Negative	61
Missing	1

Smaller values of the test result variable(s) indicate stronger evidence for a positive actual state.

a. The positive actual state is Yes.

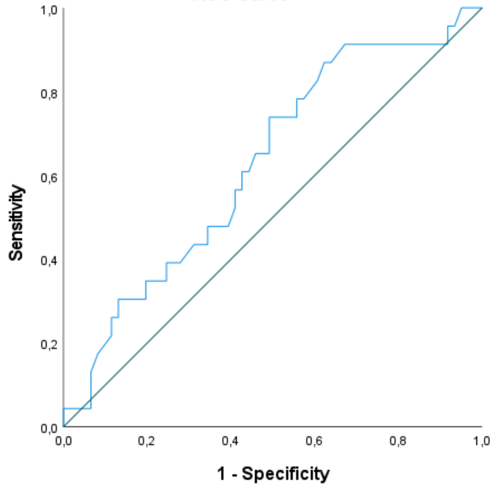

Diagonal segments are produced by ties.

Area Under the Curve

Test Result Variable(s): VAT distance

Area	Std. Error[a]	Asymptotic Sig.[b]	Asymptotic 95% Confidence Interval	
			Lower Bound	Upper Bound
,627	,067	,074	,496	,757

The test result variable(s): VAT distance has at least one tie between the positive actual state group and the negative actual state group. Statistics may be biased.

a. Under the nonparametric assumption

b. Null hypothesis: true area = 0.5

Youden Index: 1.193; cut-off: 15.05; sensibility: 0.652; specificity: 0.541

A significant result is not obtained ($p > 0.05$), therefore the variable does not discriminate well between the two groups.

- **Cerebrovascular events in 24-months follow-up**

Case Processing Summary

Cerebrovascular events in 24-months follow-up	Valid N (listwise)
Positive[a]	22
Negative	62
Missing	1

Smaller values of the test result variable(s) indicate stronger evidence for a positive actual state.

a. The positive actual state is Yes.

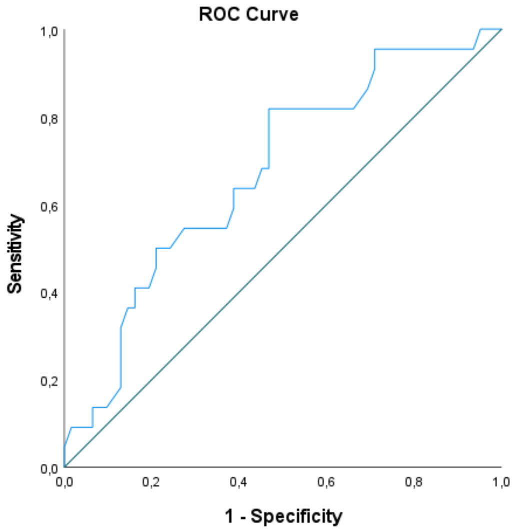

Diagonal segments are produced by ties.

Area Under the Curve

Test Result Variable(s): VAT distance

Area	Std. Error[a]	Asymptotic Sig.[b]	Asymptotic 95% Confidence Interval	
			Lower Bound	Upper Bound
,673	,065	,016	,545	,801

The test result variable(s): VAT distance has at least one tie between the positive actual state group and the negative actual state group. Statistics may be biased.

a. Under the nonparametric assumption

b. Null hypothesis: true area = 0.5

Youden Index: 1.350; cut-off: 17.30; sensibility: 0.818; specificity: 0.532

There is a significant result ($p < 0.05$) and the AUC is equal to 0.673, therefore the test is not very accurate. The cut-off found indicates the value above which the 0's are expected**.**

- **Mortality in 24-months follow-up**

Case Processing Summary

Mortality in 24-months follow-up	Valid N (listwise)
Positive[a]	28
Negative	56
Missing	1

Smaller values of the test result variable(s) indicate stronger evidence for a positive actual state.

a. The positive actual state is Yes.

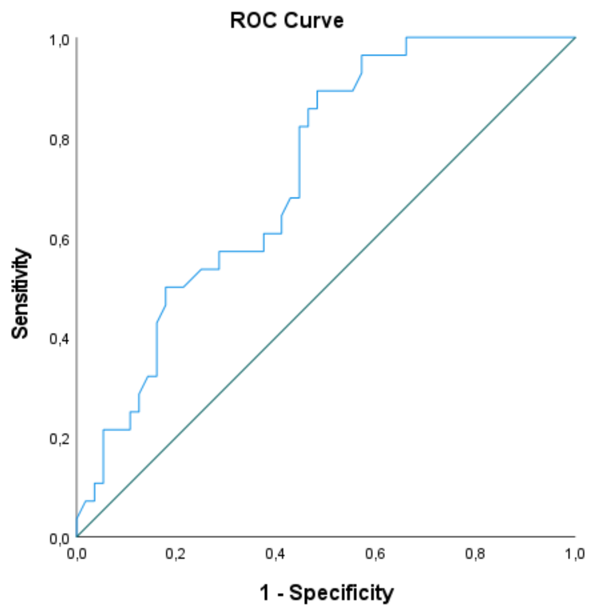

Diagonal segments are produced by ties.

Area Under the Curve

Test Result Variable(s): VAT distance

Area	Std. Error[a]	Asymptotic Sig.[b]	Asymptotic 95% Confidence Interval	
			Lower Bound	Upper Bound
,727	,055	,001	,620	,834

The test result variable(s): VAT distance has at least one tie between the positive actual state group and the negative actual state group. Statistics may be biased.

a. Under the nonparametric assumption

b. Null hypothesis: true area = 0.5

Youden Index: 1.393; cut-off: 18.95; sensibility: 0.857; specificity: 0.536

There is a significant result (p <0.05) and the AUC is equal to 0.727, therefore the test is moderately accurate. The cut-off found indicates the value above which the 0's are expected.

Cut-off FAT area

- **Adverse cardiac events in 24-months follow-up**

Case Processing Summary

Adverse cardiac events in 24-months follow-up	Valid N (listwise)
Positive[a]	24
Negative	61

Smaller values of the test result variable(s) indicate stronger evidence for a positive actual state.

a. The positive actual state is Yes.

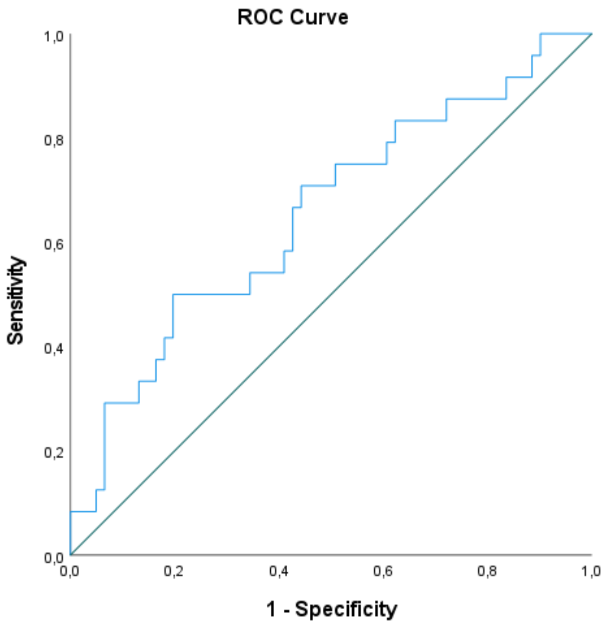

Area Under the Curve

Test Result Variable(s): FAT area (cm2)

Area	Std. Error[a]	Asymptotic Sig.[b]	Asymptotic 95% Confidence Interval	
			Lower Bound	Upper Bound
,654	,068	,028	,521	,786

a. Under the nonparametric assumption

b. Null hypothesis: true area = 0.5

Youden Index: 1.240; cut-off: 38551.35; sensibility: 0.667; specificity: 0.574

There is a significant result ($p < 0.05$) and the AUC is equal to 0.654, therefore the test is not very accurate. The cut-off found indicates the value above which the 0's are expected**.**

- **Cerebrovascular events in 24-months follow-up**

Case Processing Summary

Cerebrovascular events in 24-months follow-up	Valid N (listwise)
Positive[a]	22
Negative	63

Smaller values of the test result variable(s) indicate stronger evidence for a positive actual state.

a. The positive actual state is Yes.

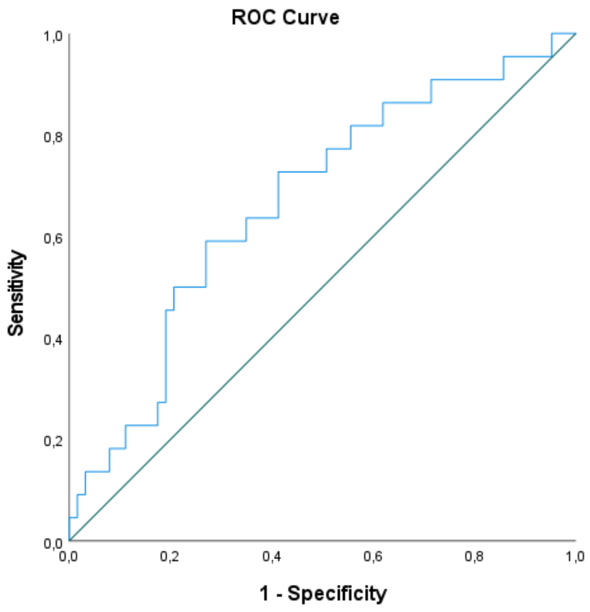

Area Under the Curve

Test Result Variable(s): FAT area (cm2)

Area	Std. Error[a]	Asymptotic Sig.[b]	Asymptotic 95% Confidence Interval	
			Lower Bound	Upper Bound
,668	,067	,019	,538	,799

a. Under the nonparametric assumption

b. Null hypothesis: true area = 0.5

Youden Index: 1.315; cut-off: 38551.35; sensibility: 0.727; specificity: 0.587

There is a significant result ($p < 0.05$) and the AUC is equal to 0.668, therefore the test is not very accurate. The cut-off found indicates the value above which the 0's are expected.

- **Mortality in 24-months follow-up**

Case Processing Summary

Mortality in 24-months follow-up	Valid N (listwise)
Positive[a]	28
Negative	57

Smaller values of the test result variable(s) indicate stronger evidence for a positive actual state.

a. The positive actual state is Yes.

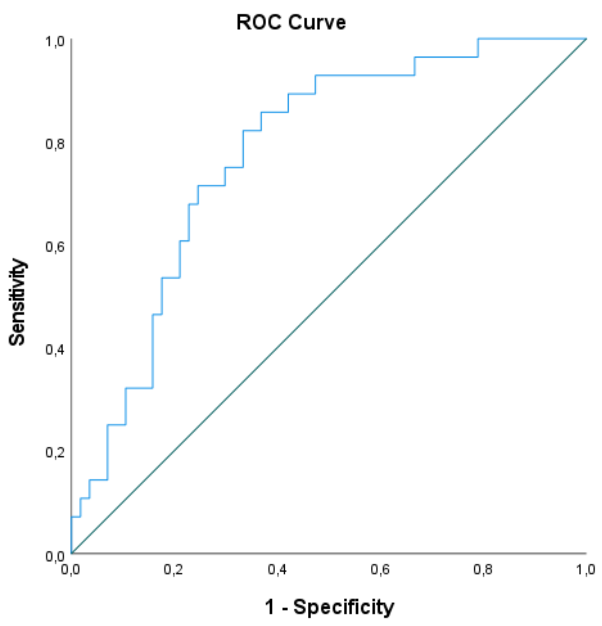

Area Under the Curve

Test Result Variable(s): FAT area (cm2)

Area	Std. Error[a]	Asymptotic Sig.[b]	Asymptotic 95% Confidence Interval	
			Lower Bound	Upper Bound
,776	,051	,000	,676	,877

a. Under the nonparametric assumption

b. Null hypothesis: true area = 0.5

Youden Index: 1.469; cut-off: 35049.45; sensibility: 0.714; specificity: 0.754

There is a significant result ($p < 0.05$) and the AUC is equal to 0.776, therefore the test is moderately accurate. The cut-off found indicates the value above which the 0's are expected.

Cut-off SAT area

- **Adverse cardiac events in 24-months follow-up**

Case Processing Summary

Adverse cardiac events in 24-months follow-up	Valid N (listwise)
Positive[a]	24
Negative	61

Smaller values of the test result variable(s) indicate stronger evidence for a positive actual state.

a. The positive actual state is Yes.

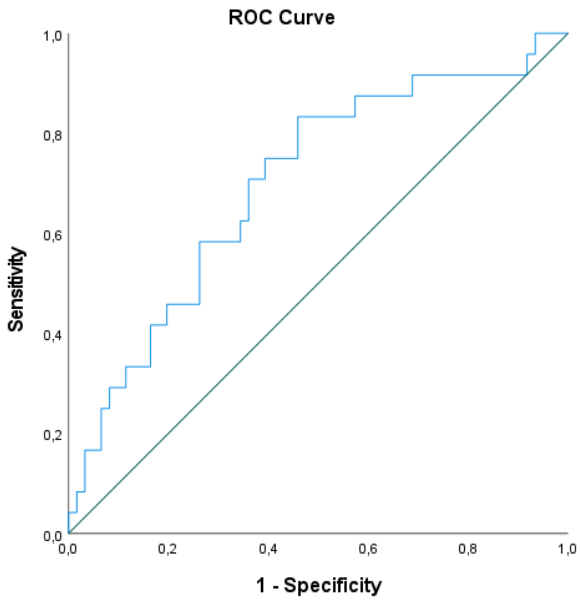

Area Under the Curve

Test Result Variable(s): SAT area (cm2)

Area	Std. Error[a]	Asymptotic Sig.[b]	Asymptotic 95% Confidence Interval	
			Lower Bound	Upper Bound
,699	,063	,004	,575	,824

a. Under the nonparametric assumption

b. Null hypothesis: true area = 0.5

Youden Index: 1.357; cut-off: 23210.35; sensibility: 0.750; specificity: 0.607

There is a significant result ($p < 0.05$) and the AUC is equal to 0.699, therefore the test is not very accurate. The cut-off found indicates the value above which the 0's are expected.

- **Cerebrovascular events in 24-months follow-up**

Case Processing Summary

Cerebrovascular events in 24-months follow-up	Valid N (listwise)
Positive[a]	22
Negative	63

Smaller values of the test result variable(s) indicate stronger evidence for a positive actual state.

a. The positive actual state is Yes.

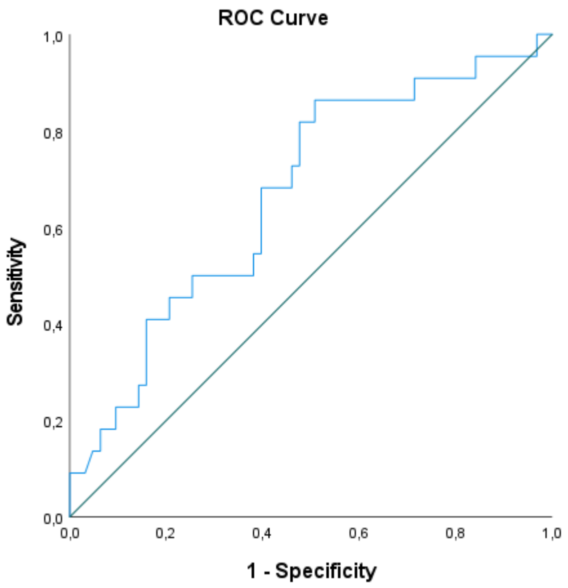

Diagonal segments are produced by ties.

Area Under the Curve

Test Result Variable(s): SAT area (cm2)

Area	Std. Error[a]	Asymptotic Sig.[b]	Asymptotic 95% Confidence Interval	
			Lower Bound	Upper Bound
,668	,066	,019	,539	,798

The test result variable(s): SAT area (cm2) has at least one tie between the positive actual state group and the negative actual state group. Statistics may be biased.

a. Under the nonparametric assumption

b. Null hypothesis: true area = 0.5

Youden Index: 1.285; cut-off: 22951.45; sensibility: 0.682; specificity: 0.503

There is a significant result ($p < 0.05$) and the AUC is equal to 0.668, therefore the test is not very accurate. The cut-off found indicates the value above which the 0's are expected.

- **Mortality in 24-months follow-up**

Case Processing Summary

Mortality in 24-months follow-up	Valid N (listwise)
Positive[a]	28
Negative	57

Smaller values of the test result variable(s) indicate stronger evidence for a positive actual state.

a. The positive actual state is Yes.

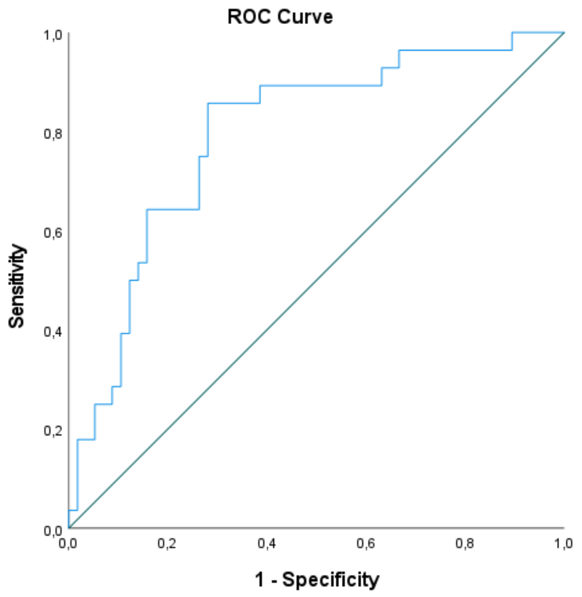

Area Under the Curve

Test Result Variable(s): SAT area (cm2)

Area	Std. Error[a]	Asymptotic Sig.[b]	Asymptotic 95% Confidence Interval	
			Lower Bound	Upper Bound
,794	,052	,000	,692	,895

a. Under the nonparametric assumption

b. Null hypothesis: true area = 0.5

Youden Index: 1.576; cut-off: 22951.45; sensibility: 0.857; specificity: 0.719

There is a significant result ($p < 0.05$) and the AUC is equal to 0.794, therefore the test is moderately accurate. The cut-off found indicates the value above which the 0's are expected.

Cut-off VAT area

- **Adverse cardiac events in 24-months follow-up**

Case Processing Summary

Adverse cardiac events in 24-months follow-up	Valid N (listwise)
Positive[a]	23
Negative	61
Missing	1

Smaller values of the test result variable(s) indicate stronger evidence for a positive actual state.

a. The positive actual state is Yes.

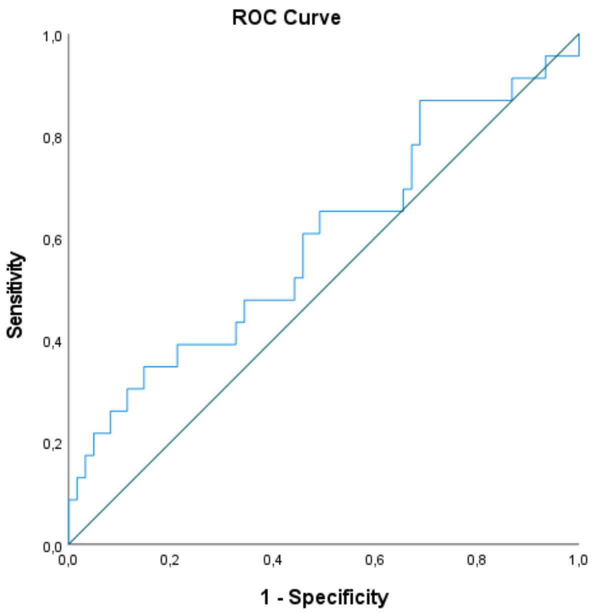

Area Under the Curve

Test Result Variable(s): VAT area (cm2)

Area	Std. Error[a]	Asymptotic Sig.[b]	Asymptotic 95% Confidence Interval	
			Lower Bound	Upper Bound
,593	,073	,191	,449	,737

a. Under the nonparametric assumption

b. Null hypothesis: true area = 0.5

Youden Index: 1.160; cut-off: 15590.65; sensibility: 0.652; specificity: 0.508

A significant result is not obtained ($p > 0.05$), therefore the variable does not discriminate well between the two groups.

- **Cerebrovascular events in 24-months follow-up**

Case Processing Summary

Cerebrovascular events in 24-months follow-up	Valid N (listwise)
Positive[a]	22
Negative	62
Missing	1

Smaller values of the test result variable(s) indicate stronger evidence for a positive actual state.

a. The positive actual state is Yes.

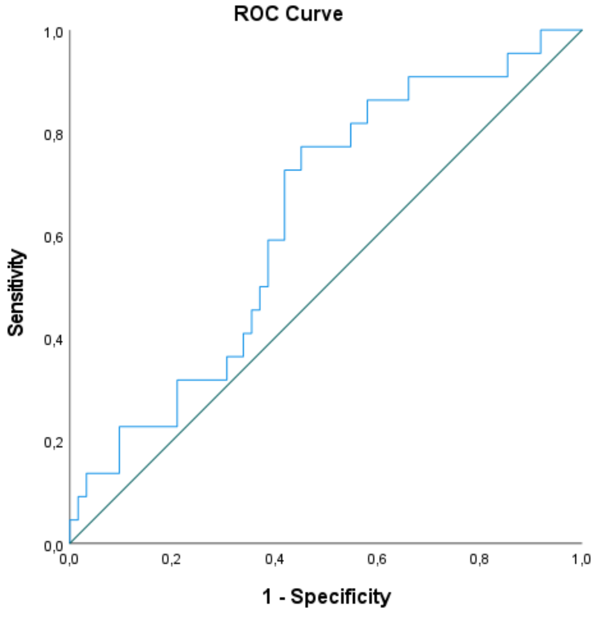

Area Under the Curve

Test Result Variable(s): VAT area (cm2)

Area	Std. Error[a]	Asymptotic Sig.[b]	Asymptotic 95% Confidence Interval	
			Lower Bound	Upper Bound
,633	,066	,066	,504	,761

a. Under the nonparametric assumption

b. Null hypothesis: true area = 0.5

Youden Index: 1.308; cut-off: 15107.55; sensibility: 0.727; specificity: 0.581

A significant result is not obtained ($p > 0.05$), therefore the variable does not discriminate well between the two groups.

- **Mortality in 24-months follow-up**

Case Processing Summary

Mortality in 24-months follow-up	Valid N (listwise)
Positive[a]	28
Negative	56
Missing	1

Smaller values of the test result variable(s) indicate stronger evidence for a positive actual state.

a. The positive actual state is Yes.

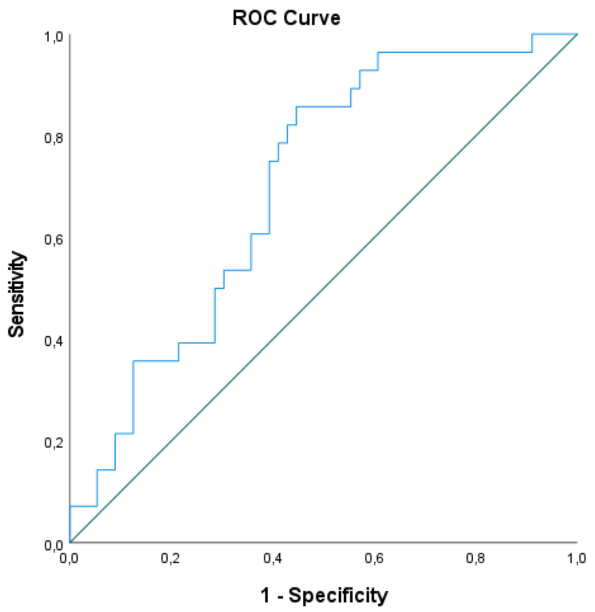

Area Under the Curve

Test Result Variable(s): VAT area (cm2)

Area	Std. Error[a]	Asymptotic Sig.[b]	Asymptotic 95% Confidence Interval	
			Lower Bound	Upper Bound
,701	,057	,003	,588	,813

a. Under the nonparametric assumption

b. Null hypothesis: true area = 0.5

Youden Index: 1.411; cut-off: 16609.60; sensibility: 0.857; specificity: 0.554

There is a significant result ($p < 0.05$) and the AUC is equal to 0.701, therefore the test is moderately accurate. The cut-off found indicates the value above which the 0's are expected.

Chi-Square and Cramer's V test BMI with post-TAVI complications

BMI * Intrahospital mortality

Crosstab

			Intrahospital mortality		
			No	Yes	Total
BMI	<25	Count	31	4	35
		% within BMI	88,6%	11,4%	100,0%
	>=25	Count	48	2	50
		% within BMI	96,0%	4,0%	100,0%
Total		Count	79	6	85
		% within BMI	92,9%	7,1%	100,0%

It is noted that 31 patients do not have the Intrahospital mortality and have a BMI <25, 4 have had the Intrahospital mortality and have a BMI <25, 48 have not had the Intrahospital mortality and have a BMI> = 25 and 2 people have had the Intrahospital mortality and have a BMI> = 25.

Among those who have a BMI <25, 88.6% did not have the Intrahospital mortality, while 11.4% did; among those who have a BMI> = 25, 96% did not have the Intrahospital mortality, while 6% did.

Chi-Square Tests

	Value	df	Asymptotic Significance (2-sided)	Exact Sig. (2-sided)	Exact Sig. (1-sided)
Pearson Chi-Square	1,732[a]	1	,188		
Continuity Correction[b]	,785	1	,376		
Likelihood Ratio	1,706	1	,192		
Fisher's Exact Test				,224	,187
Linear-by-Linear Association	1,711	1	,191		
N of Valid Cases	85				

a. 2 cells (50,0%) have expected count less than 5. The minimum expected count is 2,47.

b. Computed only for a 2x2 table

The chi-squared test is statistically insignificant ($p > 0.05$), therefore the null hypothesis of independence between the variables is accepted and it can be stated that the two variables are not significantly associated.

(In this case there is no need to enter Cramer's V).

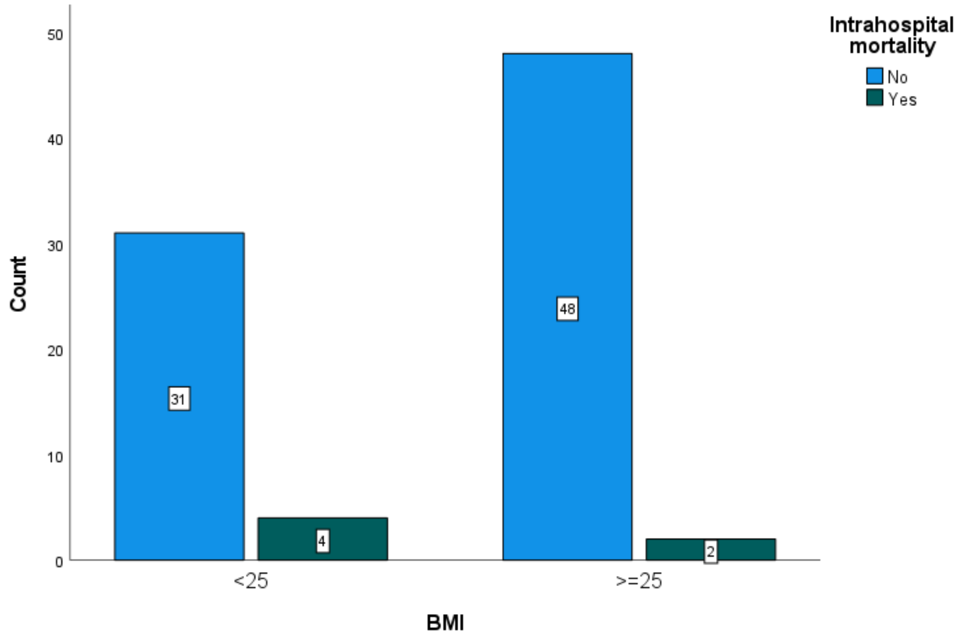

Count
50
40
30
20
10
0
31
4
48
2
<25
>=25
BMI
Intrahospital mortality
No
Yes

BMI * Hospitalization days after TAVI

Crosstab

			Hospitalization days after TAVI		
			<=5	>5	Total
BMI	<25	Count	14	21	35
		% within BMI	40,0%	60,0%	100,0%
	>=25	Count	36	14	50
		% within BMI	72,0%	28,0%	100,0%
Total		Count	50	35	85
		% within BMI	58,8%	41,2%	100,0%

It is noted that 14 patients do have the Hospitalization <= 5 days and have a BMI <25, 21 have had the Hospitalization > days and have a BMI <25, 36 have had the Hospitalization <= 5 days and have a BMI> = 25 and 14 people have had the Hospitalization > 5 days and have a BMI> = 25.

Among those who have a BMI <25, 40% had the Hospitalization <= 5 days, while 60% >5; among those who have a BMI> = 25, 72% had the Hospitalization <= 5 days, while 28% >5.

Chi-Square Tests

	Value	Df	Asymptotic Significance (2-sided)	Exact Sig. (2-sided)	Exact Sig. (1-sided)
Pearson Chi-Square	8,704[a]	1	,003		
Continuity Correction[b]	7,433	1	,006		
Likelihood Ratio	8,768	1	,003		
Fisher's Exact Test				,004	,003
Linear-by-Linear Association	8,602	1	,003		
N of Valid Cases	85				

a. 0 cells (0,0%) have expected count less than 5. The minimum expected count is 14,41.

b. Computed only for a 2x2 table

The chi-squared test is statistically significant ($p < 0.05$), therefore the null hypothesis of independence between the variables is rejected and it can be said that there is a statistically significant association between the variables.

Since the test is statistically significant, Cramer's V is interpreted.

Symmetric Measures

		Value	Approximate Significance
Nominal by Nominal	Phi	-,320	,003
	Cramer's V	,320	,003
N of Valid Cases		85	

In this case, Cramer's V, equal to 0.320, shows us that this association is of low intensity.

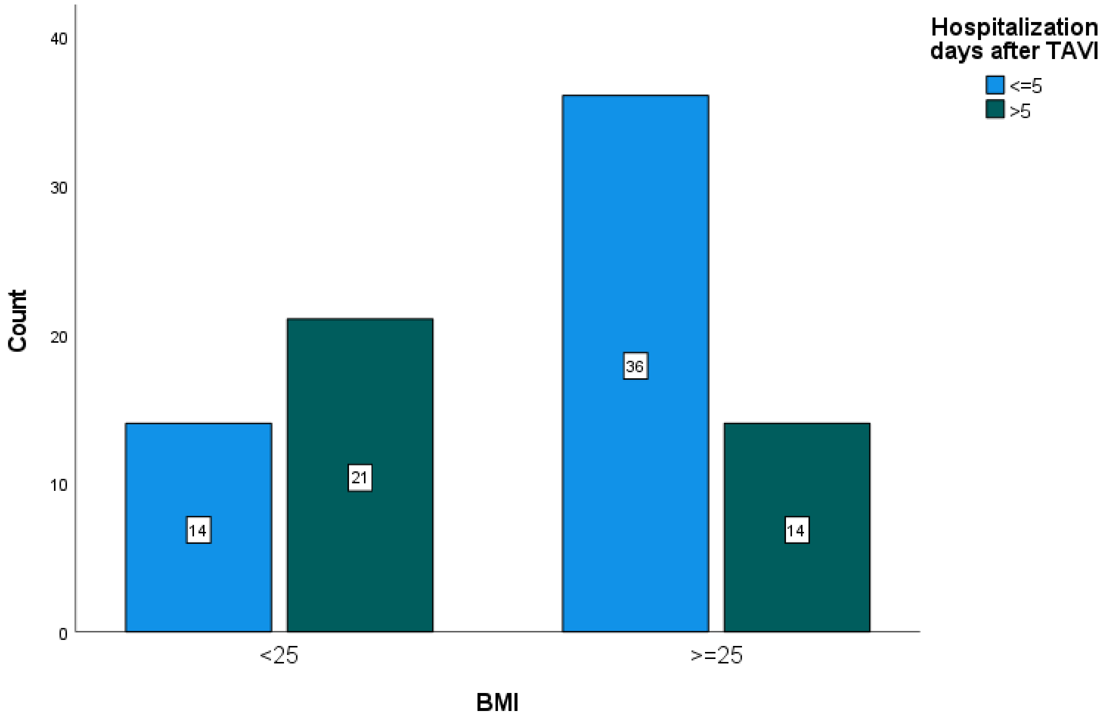

BMI * Peri-prosthetic endoleak

Crosstab

			Peri-prosthetic endoleak		Total
			No	Yes	
BMI	<25	Count	18	17	35
		% within BMI	51,4%	48,6%	100,0%
	>=25	Count	31	19	50
		% within BMI	62,0%	38,0%	100,0%
Total		Count	49	36	85
		% within BMI	57,6%	42,4%	100,0%

It is noted that 18 patients do not have the Peri-prosthetic endoleak and have a BMI <25, 17 have had the Peri-prosthetic endoleak and had have a BMI <25, 31 have not had Peri-prosthetic endoleak and have a BMI> = 25 and 19 people had have the Peri-prosthetic endoleak and have a BMI> = 25.

Among those who have a BMI <25, 51.4% did not have the Peri-prosthetic endoleak, while 48.6% did; among those who have a BMI> = 25, 62% did not have the Peri-prosthetic endoleak, while 38% did.

Chi-Square Tests

	Value	Df	Asymptotic Significance (2-sided)	Exact Sig. (2-sided)	Exact Sig. (1-sided)
Pearson Chi-Square	,942[a]	1	,332		
Continuity Correction[b]	,559	1	,455		
Likelihood Ratio	,941	1	,332		
Fisher's Exact Test				,377	,227
Linear-by-Linear Association	,931	1	,335		
N of Valid Cases	85				

a. 0 cells (0,0%) have expected count less than 5. The minimum expected count is 14,82.

b. Computed only for a 2x2 table

The chi-squared test is statistically insignificant ($p > 0.05$), therefore the null hypothesis of independence between the variables is accepted and it can be stated that the two variables are not significantly associated.

(In this case there is no need to enter Cramer's V).

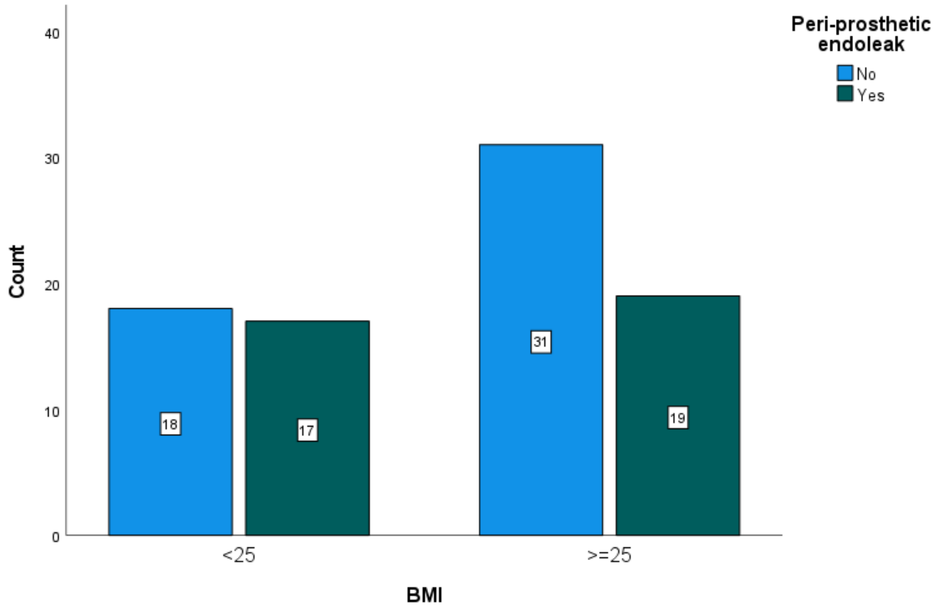

Peri-prosthetic endoleak
No
Yes
Count
40
30
20
10
0
18
17
31
19
<25
>=25
BMI

BMI * Femoral stent placement

Crosstab

			Femoral stent placement		
			No	Yes	Total
BMI	<25	Count	32	3	35
		% within BMI	91,4%	8,6%	100,0%
	>=25	Count	45	5	50
		% within BMI	90,0%	10,0%	100,0%
Total		Count	77	8	85
		% within BMI	90,6%	9,4%	100,0%

It is noted that 32 patients do not have the Femoral stent placement and have a BMI <25, 3 have had the Femoral stent placement and had have a BMI <25, 45 have not had Femoral stent placement and have a BMI> = 25 and 5 people had have the Femoral stent placement and have a BMI> = 25.

Among those who have a BMI <25, 91.4% did not have the Femoral stent placement, while 8.6% did; among those who have a BMI> = 25, 90% did not have the Femoral stent placement, while 10% did.

Chi-Square Tests

	Value	df	Asymptotic Significance (2-sided)	Exact Sig. (2-sided)	Exact Sig. (1-sided)
Pearson Chi-Square	,049[a]	1	,824		
Continuity Correction[b]	,000	1	1,000		
Likelihood Ratio	,050	1	,824		
Fisher's Exact Test				1,000	,568
Linear-by-Linear Association	,049	1	,825		
N of Valid Cases	85				

a. 2 cells (50,0%) have expected count less than 5. The minimum expected count is 3,29.

b. Computed only for a 2x2 table

The chi-squared test is statistically insignificant ($p > 0.05$), therefore the null hypothesis of independence between the variables is accepted and it can be stated that the two variables are not significantly associated.

(In this case there is no need to enter Cramer's V).

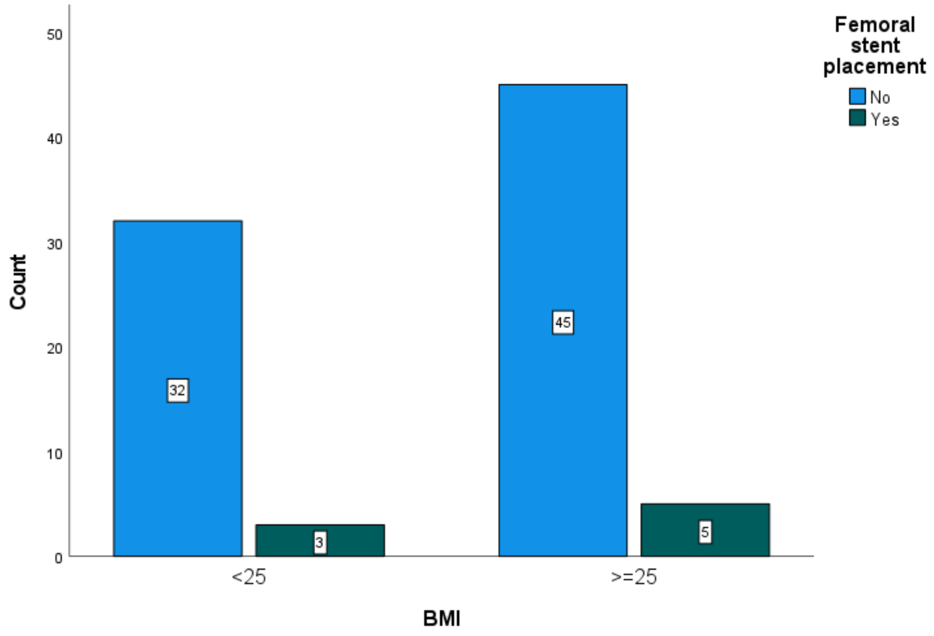

Femoral stent placement
No
Yes
Count
50
40
30
20
10
0
32
3
45
5
<25
>=25
BMI

BMI * Femoral bleeding

Crosstab

			Femoral bleeding		
			No	Yes	Total
BMI	<25	Count	32	3	35
		% within BMI	91,4%	8,6%	100,0%
	>=25	Count	49	1	50
		% within BMI	98,0%	2,0%	100,0%
Total		Count	81	4	85
		% within BMI	95,3%	4,7%	100,0%

It is noted that 32 patients do not have the Femoral bleending and have a BMI <25, 3 have had the Femoral bleending and had have a BMI <25, 49 have not had Femoral bleending and have a BMI> = 25 and 1 people had have the Femoral bleending and have a BMI> = 25. Among those who have a BMI <25, 91.4% did not have the Femoral bleending, while 8.6% did; among those who have a BMI> = 25, 98% did not have the Femoral bleending, while 2% did.

Chi-Square Tests

	Value	df	Asymptotic Significance (2-sided)	Exact Sig. (2-sided)	Exact Sig. (1-sided)
Pearson Chi-Square	1,983[a]	1	,159		
Continuity Correction[b]	,788	1	,375		
Likelihood Ratio	1,980	1	,159		
Fisher's Exact Test				,301	,187
Linear-by-Linear Association	1,959	1	,162		
N of Valid Cases	85				

a. 2 cells (50,0%) have expected count less than 5. The minimum expected count is 1,65.

b. Computed only for a 2x2 table

The chi-squared test is statistically insignificant ($p > 0.05$), therefore the null hypothesis of independence between the variables is accepted and it can be stated that the two variables are not significantly associated.

(In this case there is no need to enter Cramer's V).

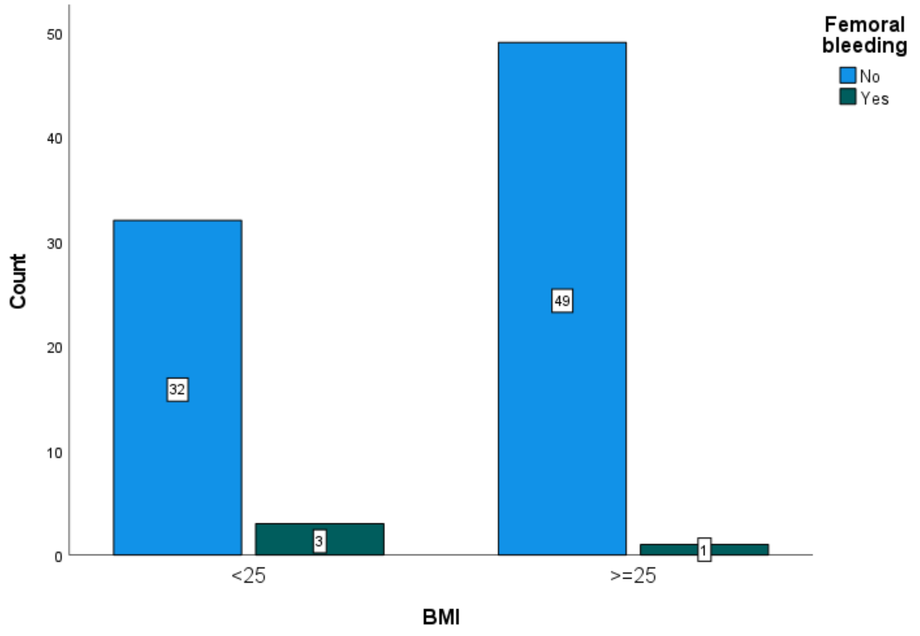

Femoral bleeding
No
Yes
Count
BMI
<25
>=25
0
10
20
30
40
50
32
3
49
1

BMI * Blood transfusion

Crosstab

			Blood transfusion		
			No	Yes	Total
BMI	<25	Count	12	23	35
		% within BMI	34,3%	65,7%	100,0%
	>=25	Count	47	3	50
		% within BMI	94,0%	6,0%	100,0%
Total		Count	59	26	85
		% within BMI	69,4%	30,6%	100,0%

It is noted that 12 patients do not have the Blood transfusion and have a BMI <25, 23 have had the Blood transfusion and had have a BMI <25, 47 have not had Blood transfusion and have a BMI> = 25 and 3 people had have the Blood transfusion and have a BMI> = 25. Among those who have a BMI <25, 34.3% did not have the Blood transfusion, while 65.7% did; among those who have a BMI> = 25, 94% did not have the Blood transfusion, while 6% did.

Chi-Square Tests

	Value	df	Asymptotic Significance (2-sided)	Exact Sig. (2-sided)	Exact Sig. (1-sided)
Pearson Chi-Square	34,577[a]	1	<,001		
Continuity Correction[b]	31,822	1	<,001		
Likelihood Ratio	36,980	1	<,001		
Fisher's Exact Test				<,001	<,001
Linear-by-Linear Association	34,170	1	<,001		
N of Valid Cases	85				

a. 0 cells (0,0%) have expected count less than 5. The minimum expected count is 10,71.

b. Computed only for a 2x2 table

The chi-squared test is statistically significant ($p < 0.05$), therefore the null hypothesis of independence between the variables is rejected and it can be said that there is a statistically significant association between the variables.
Since the test is statistically significant, Cramer's V is interpreted.

Symmetric Measures

		Value	Approximate Significance
Nominal by Nominal	Phi	-,638	<,001

Cramer's V	,638	<,001
N of Valid Cases	85	

In this case, Cramer's V, equal to 0.638, shows us that this association is of moderate intensity.

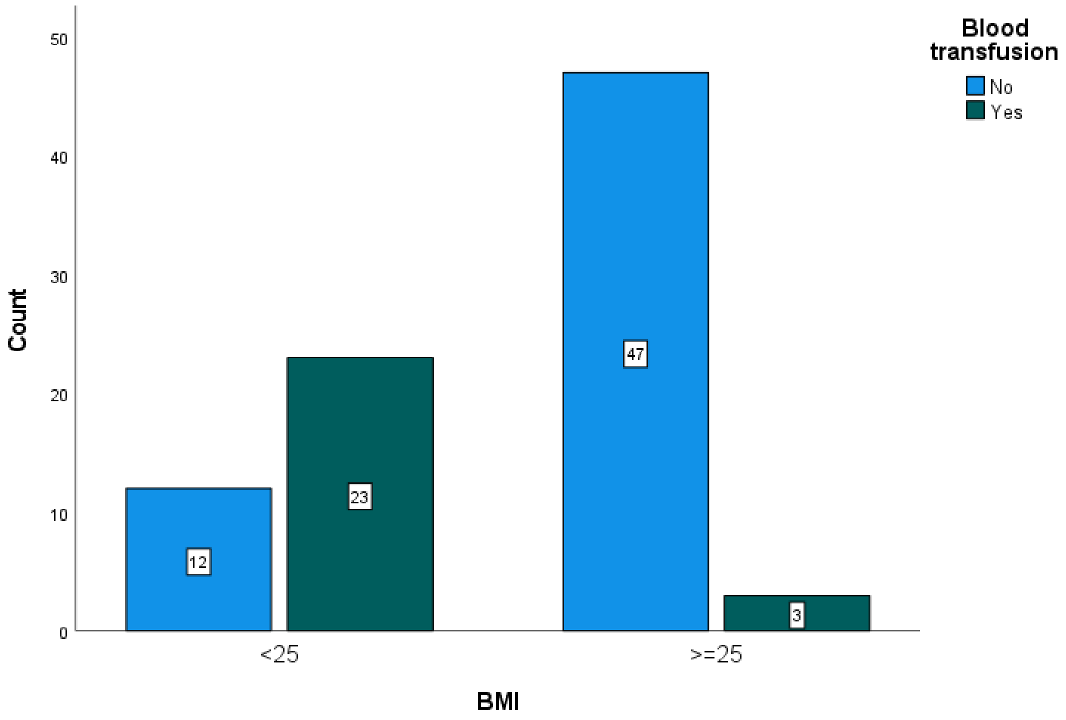

BMI * Prolonged hypotension

Crosstab

			Prolonged hypotension		
			No	Yes	Total
BMI	<25	Count	32	3	35
		% within BMI	91,4%	8,6%	100,0%
	>=25	Count	42	8	50
		% within BMI	84,0%	16,0%	100,0%
Total		Count	74	11	85
		% within BMI	87,1%	12,9%	100,0%

It is noted that 32 patients do not have the Prolonged hypotension and have a BMI <25, 3 have had the Prolonged hypotension and had have a BMI <25, 42 have not had Prolonged hypotension and have a BMI> = 25 and 8 people had have the Prolonged hypotension and have a BMI> = 25.

Among those who have a BMI <25, 91.4% did not have the Prolonged hypotension, while 8.6% did; among those who have a BMI> = 25, 84% did not have the Prolonged hypotension, while 16% did.

Chi-Square Tests

	Value	df	Asymptotic Significance (2-sided)	Exact Sig. (2-sided)	Exact Sig. (1-sided)
Pearson Chi-Square	1,008[a]	1	,315		
Continuity Correction[b]	,457	1	,499		
Likelihood Ratio	1,053	1	,305		
Fisher's Exact Test				,513	,253
Linear-by-Linear Association	,997	1	,318		
N of Valid Cases	85				

a. 1 cells (25,0%) have expected count less than 5. The minimum expected count is 4,53.

b. Computed only for a 2x2 table

The chi-squared test is statistically insignificant ($p > 0.05$), therefore the null hypothesis of independence between the variables is accepted and it can be stated that the two variables are not significantly associated.

(In this case there is no need to enter Cramer's V).

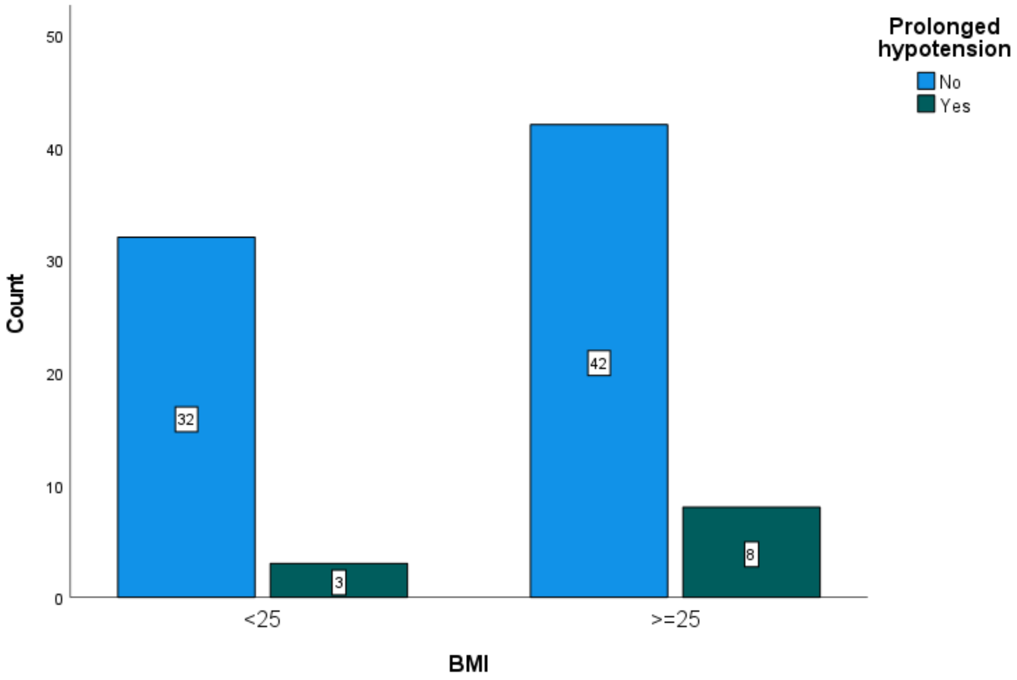

Prolonged hypotension
No
Yes
Count
50
40
30
20
10
0
32
3
42
8
<25
>=25
BMI

BMI * Bundle branch block

Crosstab

			Bundle branch block		
			No	Yes	Total
BMI	<25	Count	29	6	35
		% within BMI	82,9%	17,1%	100,0%
	>=25	Count	42	8	50
		% within BMI	84,0%	16,0%	100,0%
Total		Count	71	14	85
		% within BMI	83,5%	16,5%	100,0%

It is noted that 29 patients do not have the Bundle branch block and have a BMI <25, 6 have had the Bundle branch block and had have a BMI <25, 42 have not had Bundle branch block and have a BMI> = 25 and 8 people had have the Bundle branch block and have a BMI> = 25.
Among those who have a BMI <25, 82.9% did not have the Bundle branch block, while 17.1% did; among those who have a BMI> = 25, 84% did not have the Bundle branch block, while 16% did.

Chi-Square Tests

	Value	df	Asymptotic Significance (2-sided)	Exact Sig. (2-sided)	Exact Sig. (1-sided)
Pearson Chi-Square	,020[a]	1	,889		
Continuity Correction[b]	,000	1	1,000		
Likelihood Ratio	,019	1	,889		
Fisher's Exact Test				1,000	,558
Linear-by-Linear Association	,019	1	,889		
N of Valid Cases	85				

a. 0 cells (0,0%) have expected count less than 5. The minimum expected count is 5,76.

b. Computed only for a 2x2 table

The chi-squared test is statistically insignificant ($p > 0.05$), therefore the null hypothesis of independence between the variables is accepted and it can be stated that the two variables are not significantly associated.
(In this case there is no need to enter Cramer's V).

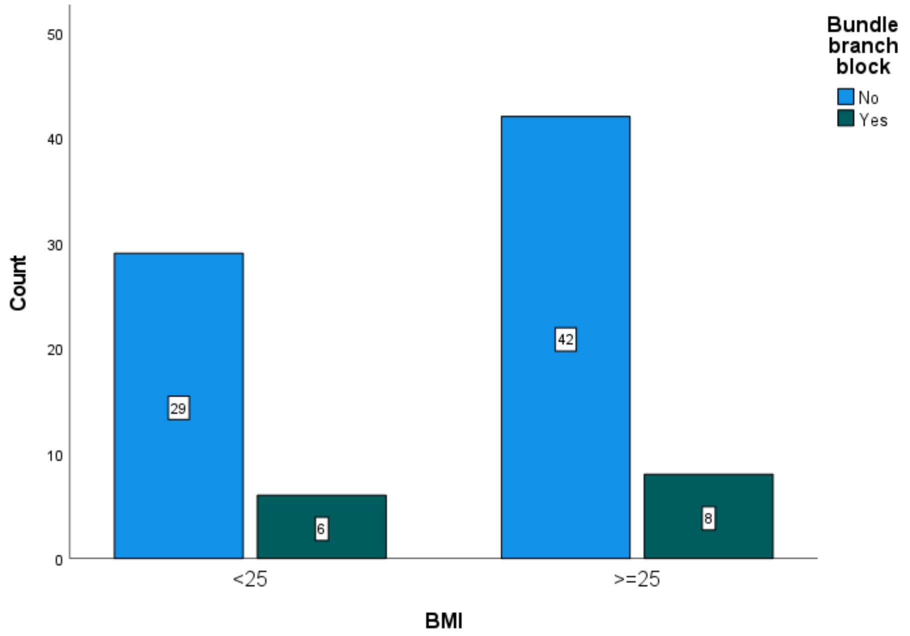

Bundle branch block
No
Yes
Count
50
40
30
20
10
0
29
6
42
8
<25
>=25
BMI

BMI * Atrioventricular block type 1

Crosstab

			Atrioventricular block type 1		
			No	Yes	Total
BMI	<25	Count	32	3	35
		% within BMI	91,4%	8,6%	100,0%
	>=25	Count	43	7	50
		% within BMI	86,0%	14,0%	100,0%
Total		Count	75	10	85
		% within BMI	88,2%	11,8%	100,0%

It is noted that 32 patients do not have the Atrioventricular block type 1 and have a BMI <25, 3 have had the Atrioventricular block type 1 and had have a BMI <25, 43 have not had Atrioventricular block type 1 and have a BMI> = 25 and 7 people had have the Atrioventricular block type 1 and have a BMI> = 25.
Among those who have a BMI <25, 91.4% did not have the Atrioventricular block type 1, while 8.6% did; among those who have a BMI> = 25, 86% did not have the Atrioventricular block type 1, while 14% did.

Chi-Square Tests

	Value	df	Asymptotic Significance (2-sided)	Exact Sig. (2-sided)	Exact Sig. (1-sided)
Pearson Chi-Square	,584[a]	1	,445		
Continuity Correction[b]	,179	1	,673		
Likelihood Ratio	,604	1	,437		
Fisher's Exact Test				,514	,342
Linear-by-Linear Association	,578	1	,447		
N of Valid Cases	85				

a. 1 cells (25,0%) have expected count less than 5. The minimum expected count is 4,12.

b. Computed only for a 2x2 table

The chi-squared test is statistically insignificant ($p > 0.05$), therefore the null hypothesis of independence between the variables is accepted and it can be stated that the two variables are not significantly associated.
(In this case there is no need to enter Cramer's V).

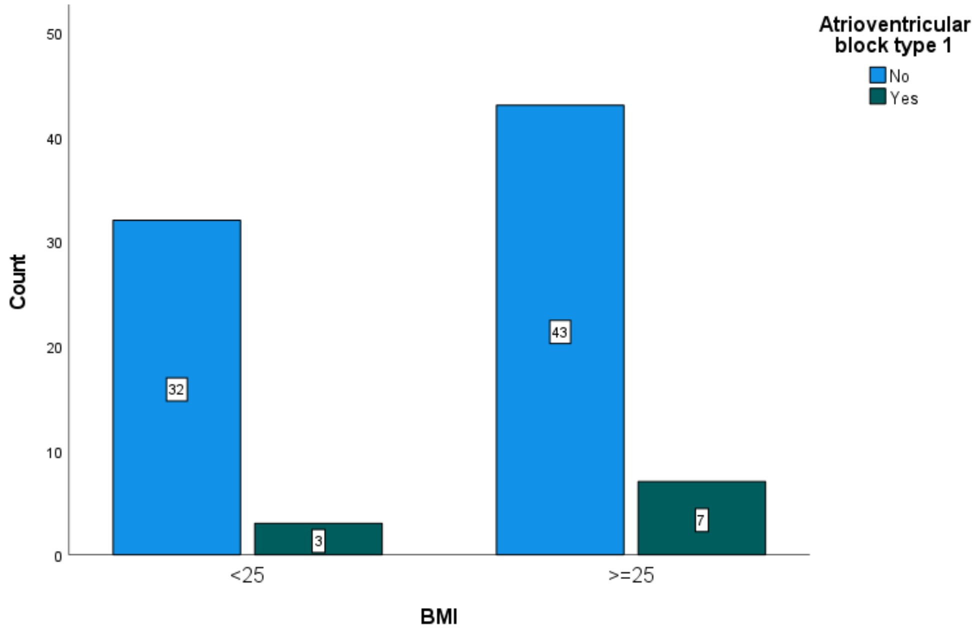
Atrioventricular block type 1
No
Yes
Count
50
40
30
20
10
0
32
3
43
7
<25
>=25
BMI

BMI * Atrioventricular block type 2

Crosstab

			Atrioventricular block type 2		
			No	Yes	Total
BMI	<25	Count	34	1	35
		% within BMI	97,1%	2,9%	100,0%
	>=25	Count	49	1	50
		% within BMI	98,0%	2,0%	100,0%
Total		Count	83	2	85
		% within BMI	97,6%	2,4%	100,0%

It is noted that 34 patients do not have the Atrioventricular block type 2 and have a BMI <25, 1 have had the Atrioventricular block type 2 and had have a BMI <25, 49 have not had Atrioventricular block type 2 and have a BMI> = 25 and 1 people had have the Atrioventricular block type 2 and have a BMI> = 25.

Among those who have a BMI <25, 97.1% did not have the Atrioventricular block type 2, while 2.9% did; among those who have a BMI> = 25, 98% did not have the Atrioventricular block type 2, while 2% did.

Chi-Square Tests

	Value	df	Asymptotic Significance (2-sided)	Exact Sig. (2-sided)	Exact Sig. (1-sided)
Pearson Chi-Square	,066[a]	1	,798		
Continuity Correction[b]	,000	1	1,000		
Likelihood Ratio	,065	1	,799		
Fisher's Exact Test				1,000	,657
Linear-by-Linear Association	,065	1	,799		
N of Valid Cases	85				

a. 2 cells (50,0%) have expected count less than 5. The minimum expected count is ,82.

b. Computed only for a 2x2 table

The chi-squared test is statistically insignificant ($p > 0.05$), therefore the null hypothesis of independence between the variables is accepted and it can be stated that the two variables are not significantly associated.

(In this case there is no need to enter Cramer's V).

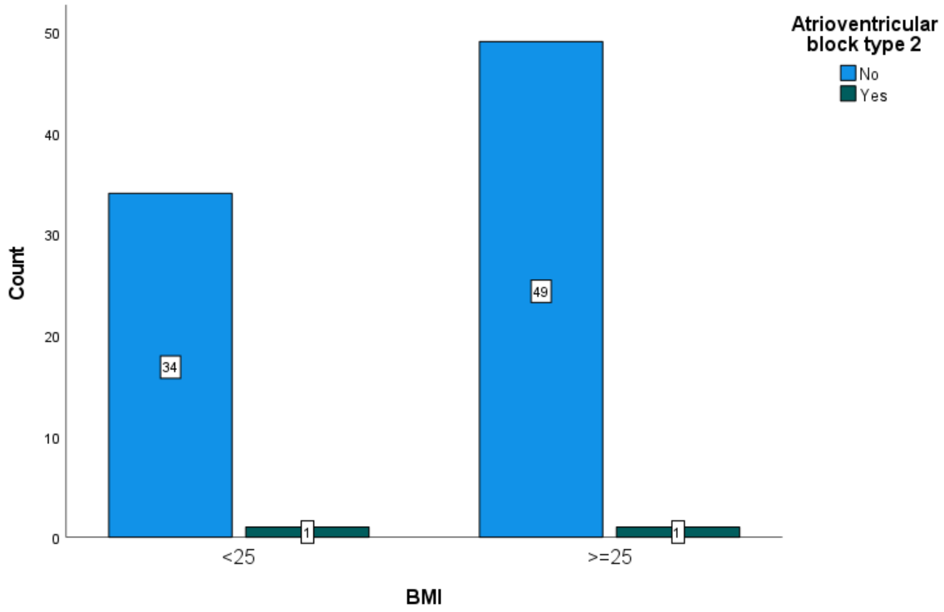

Atrioventricular block type 2
No
Yes
Count
50
40
30
20
10
0
34
1
49
1
<25
>=25
BMI

BMI * Atrioventricular block type 3

Crosstab

			Atrioventricular block type 3		
			No	Yes	Total
BMI	<25	Count	31	4	35
		% within BMI	88,6%	11,4%	100,0%
	>=25	Count	45	5	50
		% within BMI	90,0%	10,0%	100,0%
Total		Count	76	9	85
		% within BMI	89,4%	10,6%	100,0%

It is noted that 31 patients do not have the Atrioventricular block type 3 and have a BMI <25, 4 have had the Atrioventricular block type 3 and had have a BMI <25, 45 have not had Atrioventricular block type 3 and have a BMI> = 25 and 5 people had have the Atrioventricular block type 3 and have a BMI> = 25.

Among those who have a BMI <25, 88.6% did not have the Atrioventricular block type 3, while 11.4% did; among those who have a BMI> = 25, 90% did not have the Atrioventricular block type 3, while 10% did.

Chi-Square Tests

	Value	df	Asymptotic Significance (2-sided)	Exact Sig. (2-sided)	Exact Sig. (1-sided)
Pearson Chi-Square	,044[a]	1	,833		
Continuity Correction[b]	,000	1	1,000		
Likelihood Ratio	,044	1	,834		
Fisher's Exact Test				1,000	,551
Linear-by-Linear Association	,044	1	,834		
N of Valid Cases	85				

a. 1 cells (25,0%) have expected count less than 5. The minimum expected count is 3,71.

b. Computed only for a 2x2 table

The chi-squared test is statistically insignificant ($p > 0.05$), therefore the null hypothesis of independence between the variables is accepted and it can be stated that the two variables are not significantly associated.

(In this case there is no need to enter Cramer's V).

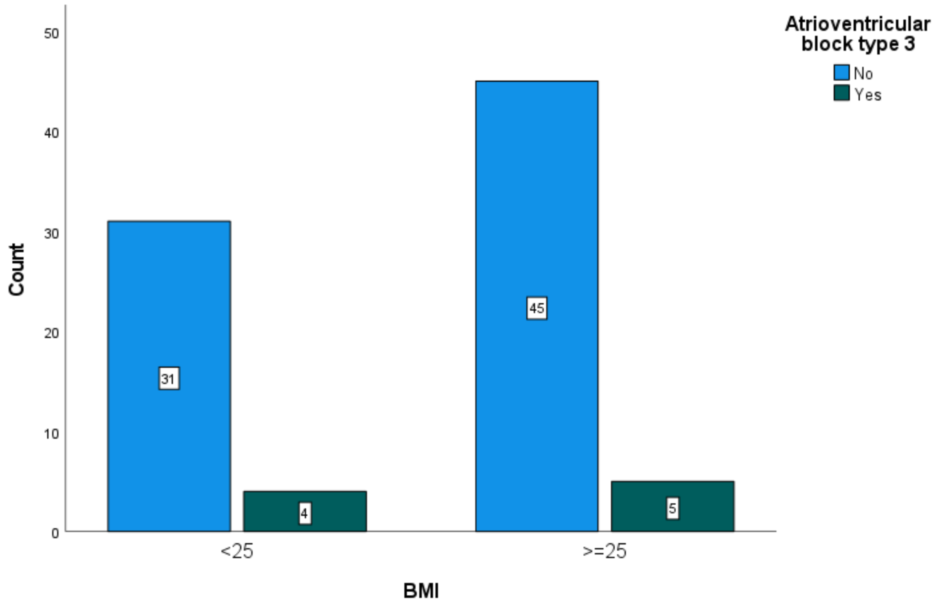

Atrioventricular block type 3
No
Yes
Count
50
40
30
20
10
0
31
4
45
5
<25
>=25
BMI

BMI * PPM implantation after TAVI

Crosstab

			PPM implantation after TAVI		
			No	Yes	Total
BMI	<25	Count	30	5	35
		% within BMI	85,7%	14,3%	100,0%
	>=25	Count	37	13	50
		% within BMI	74,0%	26,0%	100,0%
Total		Count	67	18	85
		% within BMI	78,8%	21,2%	100,0%

It is noted that 30 patients do not have the PPM implantation after TAVI and have a BMI <25, 5 have had the PPM implantation after TAVI and had have a BMI <25, 37 have not had PPM implantation after TAVI and have a BMI> = 25 and 13 people had have the PPM implantation after TAVI and have a BMI> = 25.

Among those who have a BMI <25, 85.7% did not have the PPM implantation after TAVI, while 14.3% did; among those who have a BMI> = 25, 74% did not have the PPM implantation after TAVI while 26% did.

Chi-Square Tests

	Value	df	Asymptotic Significance (2-sided)	Exact Sig. (2-sided)	Exact Sig. (1-sided)
Pearson Chi-Square	1,693[a]	1	,193		
Continuity Correction[b]	1,064	1	,302		
Likelihood Ratio	1,755	1	,185		
Fisher's Exact Test				,281	,151
Linear-by-Linear Association	1,673	1	,196		
N of Valid Cases	85				

a. 0 cells (0,0%) have expected count less than 5. The minimum expected count is 7,41.

b. Computed only for a 2x2 table

The chi-squared test is statistically insignificant ($p > 0.05$), therefore the null hypothesis of independence between the variables is accepted and it can be stated that the two variables are not significantly associated.

(In this case there is no need to enter Cramer's V).

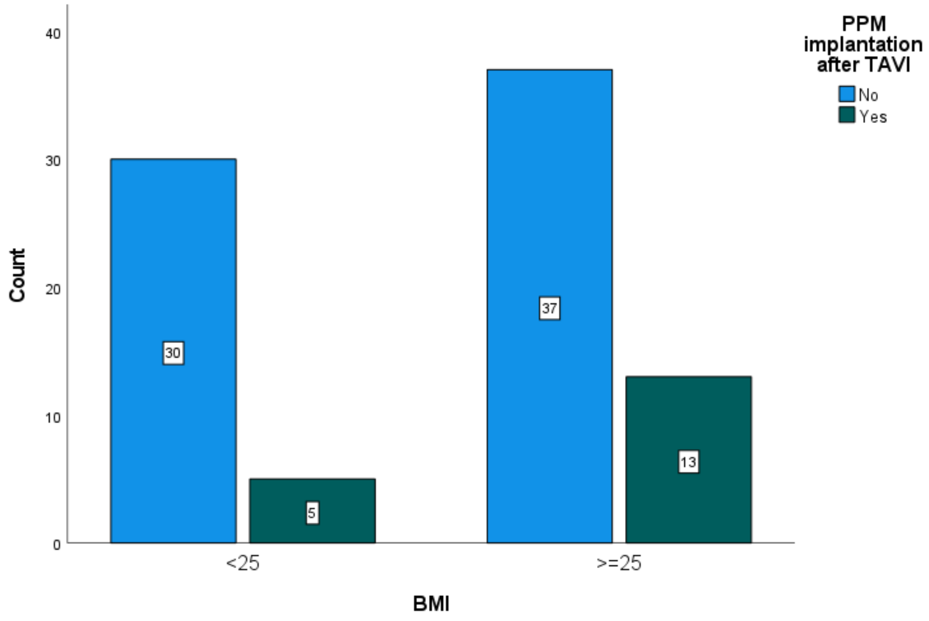
PPM implantation after TAVI
No
Yes
Count
40
30
20
10
0
30
5
37
13
<25
>=25
BMI

Independent-Samples Mann-Whitney U Test for Days of intensive care

Group Statistics

	BMI	N	Mean	Std. Deviation	Std. Error Mean
Days of intensive care	<25	35	4,29	4,787	,809
	>=25	50	2,42	,859	,122

Independent Samples Test

		Levene's Test for Equality of Variances		t-test for Equality of Means							
						Significance				95% Confidence Interval of the Difference	
		F	Sig.	t	df	One-Sided p	Two-Sided p	Mean Difference	Std. Error Difference	Lower	Upper
Days of intensive care	Equal variances assumed	4,267	,042	2,701	83	,004	,008	1,87	,691	,49	3,24
	Equal variances not assumed			2,280	35,538	,014	,029	1,87	,818	,21	3,53

The Levene test is rejected ($p < 0.05$) and the robust t test is looked at, the qaule rejects the null hypothesis of equality between means ($p < 0.05$). It is therefore concluded that the mean is significantly higher in the BMI <25 group.

Independent-Samples Mann-Whitney U Test for intrahospital mortality

Hypothesis Test Summary

	Null Hypothesis	Test	Sig.[a,b]	Decision
1	The distribution of Psoas/height is the same across categories of Intrahospital mortality.	Independent-Samples Mann-Whitney U Test	,938	Retain the null hypothesis.
2	The distribution of Anterior SAT distance is the same across categories of Intrahospital mortality.	Independent-Samples Mann-Whitney U Test	,311	Retain the null hypothesis.
3	The distribution of Posterior SAT distance is the same across categories of Intrahospital mortality.	Independent-Samples Mann-Whitney U Test	,368	Retain the null hypothesis.
4	The distribution of Anterior+Posterior SAT distance is the same across categories of Intrahospital mortality.	Independent-Samples Mann-Whitney U Test	,295	Retain the null hypothesis.
5	The distribution of VAT distance is the same across categories of Intrahospital mortality.	Independent-Samples Mann-Whitney U Test	,058	Retain the null hypothesis.
6	The distribution of Right common femoral artery area (mm2) is the same across categories of Intrahospital mortality.	Independent-Samples Mann-Whitney U Test	,142	Retain the null hypothesis.
7	The distribution of Left common femoral artery area (mm2) is the same across categories of Intrahospital mortality.	Independent-Samples Mann-Whitney U Test	,213	Retain the null hypothesis.
8	The distribution of FAT area (cm2) is the same across categories of Intrahospital mortality.	Independent-Samples Mann-Whitney U Test	,345	Retain the null hypothesis.

9	The distribution of SAT area (cm2) is the same across categories of Intrahospital mortality.	Independent-Samples Mann-Whitney U Test	,681	Retain the null hypothesis.
10	The distribution of VAT area (cm2) is the same across categories of Intrahospital mortality.	Independent-Samples Mann-Whitney U Test	,106	Retain the null hypothesis.
11	The distribution of Right Psoas muscle area (cm2) is the same across categories of Intrahospital mortality.	Independent-Samples Mann-Whitney U Test	,303	Retain the null hypothesis.
12	The distribution of Left Psoas muscle area (cm2) is the same across categories of Intrahospital mortality.	Independent-Samples Mann-Whitney U Test	,363	Retain the null hypothesis.
13	The distribution of FAT mean density (HU) is the same across categories of Intrahospital mortality.	Independent-Samples Mann-Whitney U Test	,103	Retain the null hypothesis.
14	The distribution of FAT median density (HU) is the same across categories of Intrahospital mortality.	Independent-Samples Mann-Whitney U Test	,078	Retain the null hypothesis.
15	The distribution of FAT density standard deviation is the same across categories of Intrahospital mortality.	Independent-Samples Mann-Whitney U Test	,136	Retain the null hypothesis.
16	The distribution of SAT mean density (HU) is the same across categories of Intrahospital mortality.	Independent-Samples Mann-Whitney U Test	,145	Retain the null hypothesis.
17	The distribution of SAT median density (HU) is the same across categories of Intrahospital mortality.	Independent-Samples Mann-Whitney U Test	,157	Retain the null hypothesis.
18	The distribution of SAT density standard deviation is the same across categories of Intrahospital mortality.	Independent-Samples Mann-Whitney U Test	,181	Retain the null hypothesis.

19	The distribution of VAT mean density (HU) is the same across categories of Intrahospital mortality.	Independent-Samples Mann-Whitney U Test	,008	Reject the null hypothesis.
20	The distribution of VAT median density (HU) is the same across categories of Intrahospital mortality.	Independent-Samples Mann-Whitney U Test	,010	Reject the null hypothesis.
21	The distribution of VAT density standard deviation is the same across categories of Intrahospital mortality.	Independent-Samples Mann-Whitney U Test	,165	Retain the null hypothesis.
22	The distribution of Right Psoas muscle mean density (HU) is the same across categories of Intrahospital mortality.	Independent-Samples Mann-Whitney U Test	,170	Retain the null hypothesis.
23	The distribution of Right Psoas muscle median density (HU) is the same across categories of Intrahospital mortality.	Independent-Samples Mann-Whitney U Test	,172	Retain the null hypothesis.
24	The distribution of Right Psoas muscle density standard deviation is the same across categories of Intrahospital mortality.	Independent-Samples Mann-Whitney U Test	,265	Retain the null hypothesis.
25	The distribution of Left Psoas muscle mean density (HU) is the same across categories of Intrahospital mortality.	Independent-Samples Mann-Whitney U Test	,548	Retain the null hypothesis.
26	The distribution of Left Psoas muscle median density (HU) is the same across categories of Intrahospital mortality.	Independent-Samples Mann-Whitney U Test	,429	Retain the null hypothesis.
27	The distribution of Left Psoas muscle density standard deviation is the same across categories of Intrahospital mortality.	Independent-Samples Mann-Whitney U Test	,154	Retain the null hypothesis.

a. The significance level is ,050.

b. Asymptotic significance is displayed.

In this case, the hypothesis of equal medians ($p < 0.05$) is rejected only for the variables VAT mean density (HU) and VAT median density (HU), while for the others the null hypothesis is accepted ($p > 0.05$).
(The tables and graphs below are the details of the tests in this table: I have highlighted what things you should eventually report, namely test statistic and pvalue).

Psoas/height across Intrahospital mortality

Independent-Samples Mann-Whitney U Test Summary

Total N	85
Mann-Whitney U	241,500
Wilcoxon W	262,500
Test Statistic	241,500
Standard Error	58,284
Standardized Test Statistic	,077
Asymptotic Sig.(2-sided test)	,938

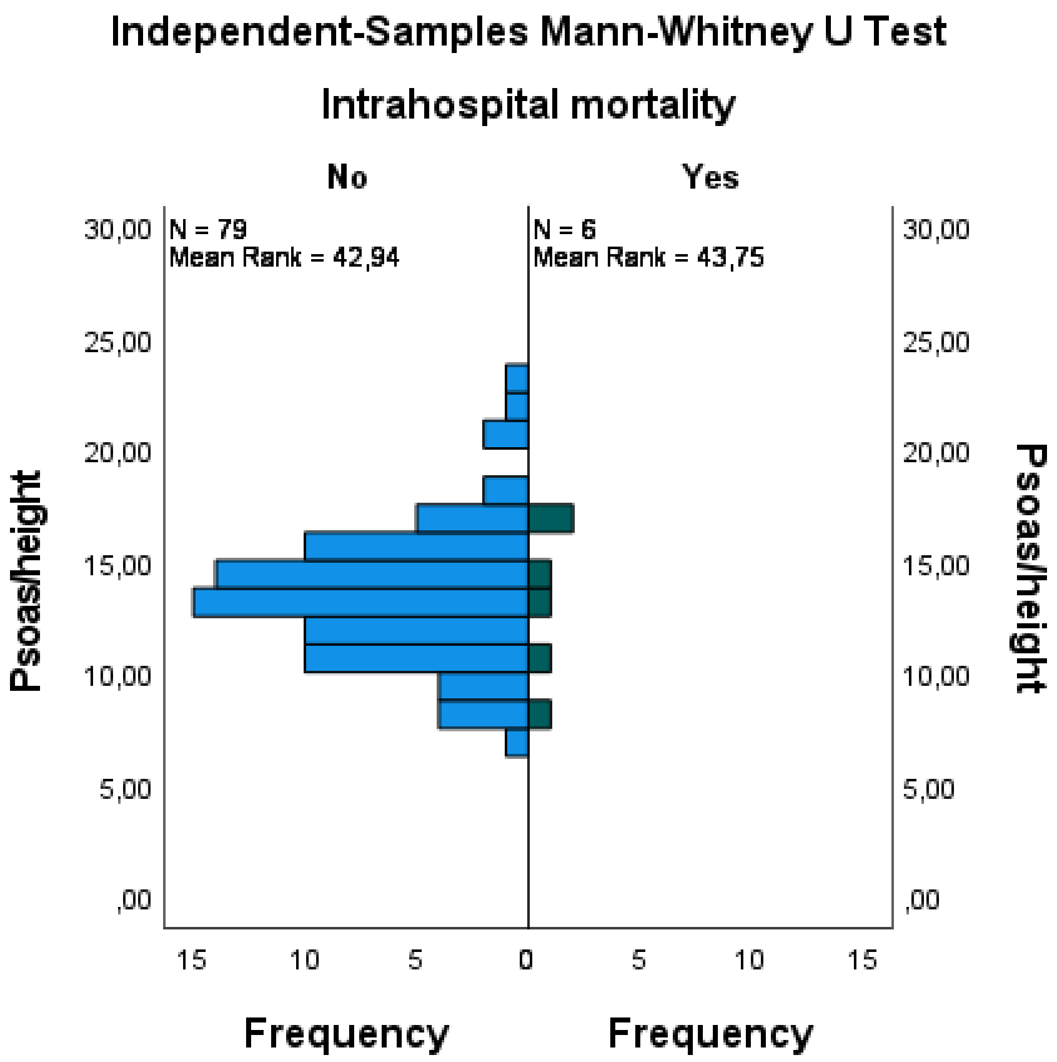

Anterior SAT distance across Intrahospital mortality

Independent-Samples Mann-Whitney U Test Summary

Total N	85
Mann-Whitney U	178,000
Wilcoxon W	199,000
Test Statistic	178,000
Standard Error	58,276
Standardized Test Statistic	-1,012
Asymptotic Sig.(2-sided test)	,311

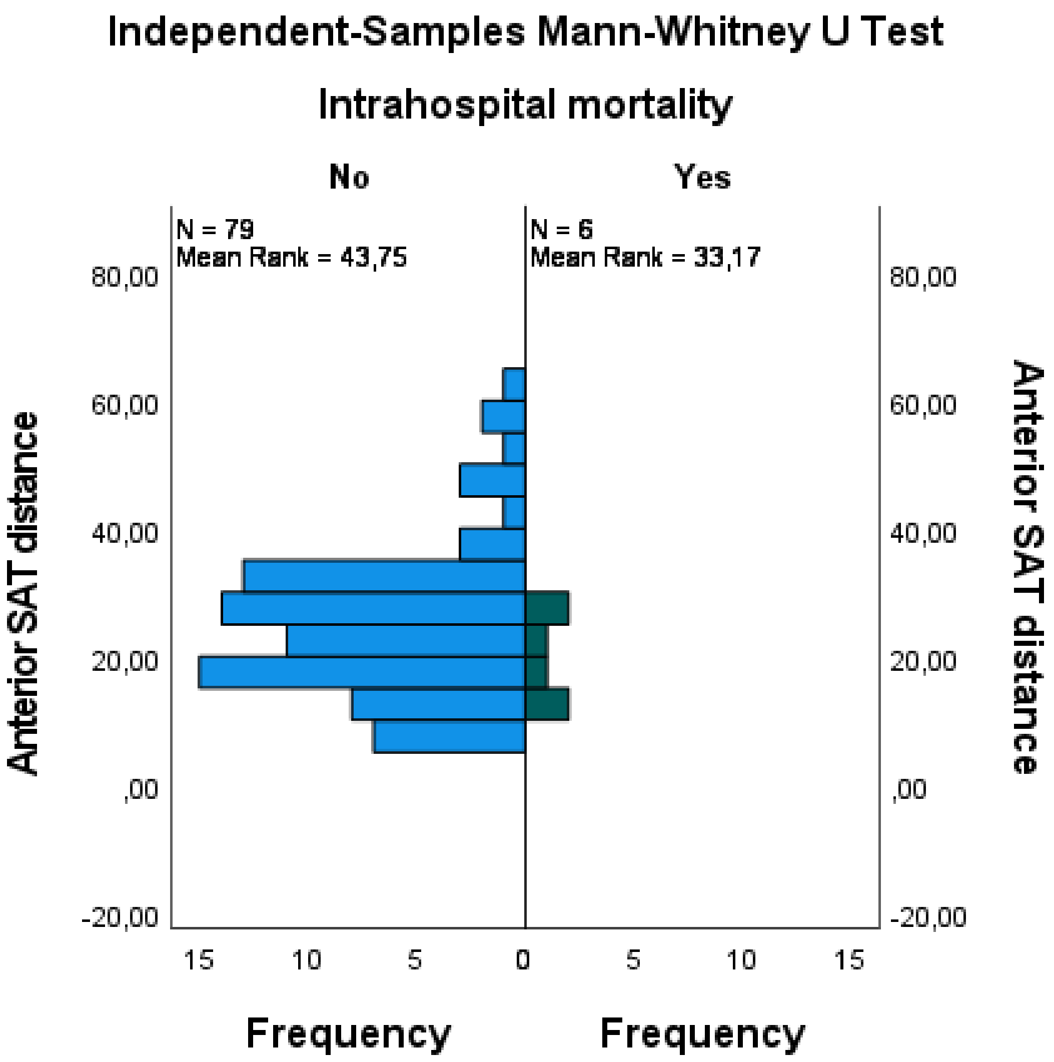

Posterior SAT distance across Intrahospital mortality

Independent-Samples Mann-Whitney U Test Summary

Total N	85
Mann-Whitney U	184,500
Wilcoxon W	205,500
Test Statistic	184,500
Standard Error	58,277
Standardized Test Statistic	-,901

Asymptotic Sig.(2-sided test)	,368

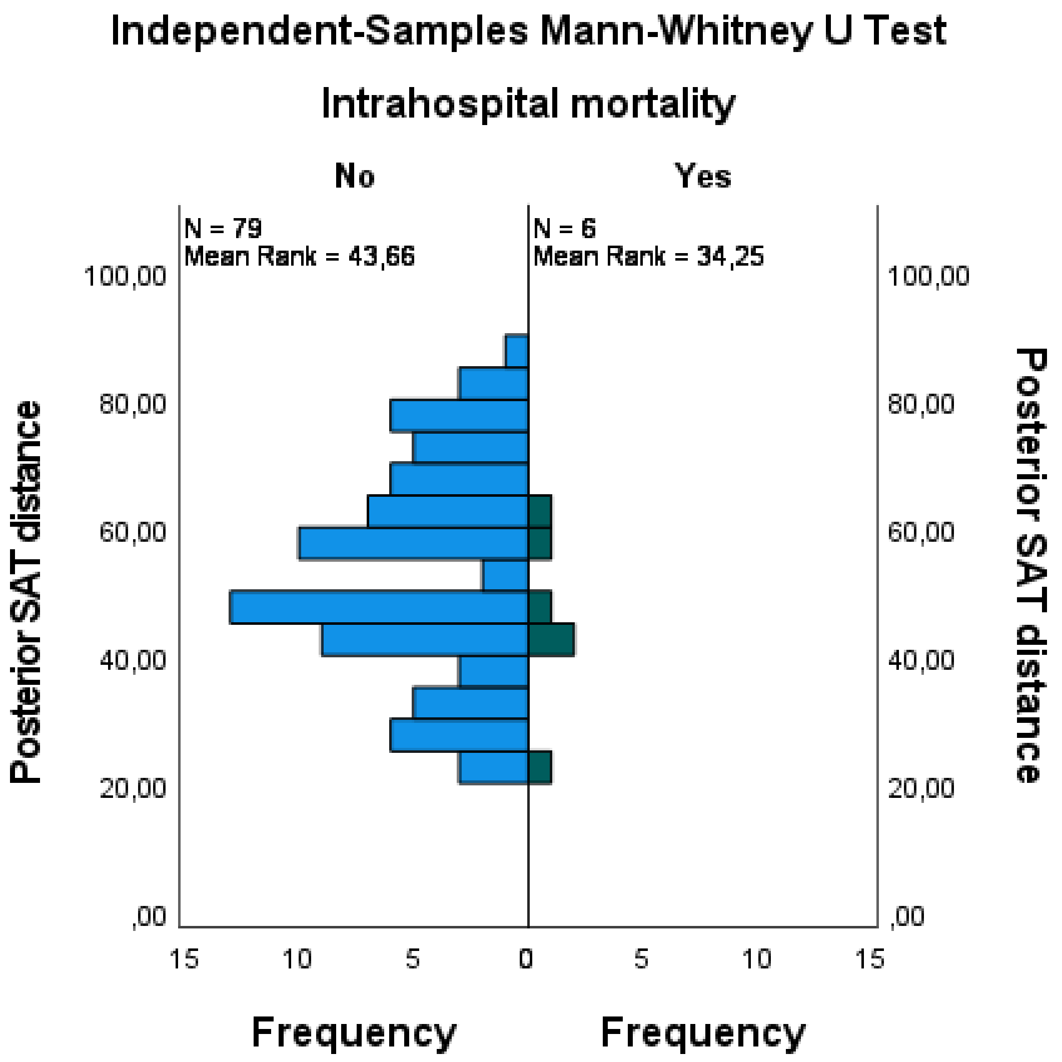

Anterior+Posterior SAT distance across Intrahospital mortality

Independent-Samples Mann-Whitney U Test Summary

Total N	85
Mann-Whitney U	176,000
Wilcoxon W	197,000
Test Statistic	176,000
Standard Error	58,281
Standardized Test Statistic	-1,047

Asymptotic Sig.(2-sided test)	,295

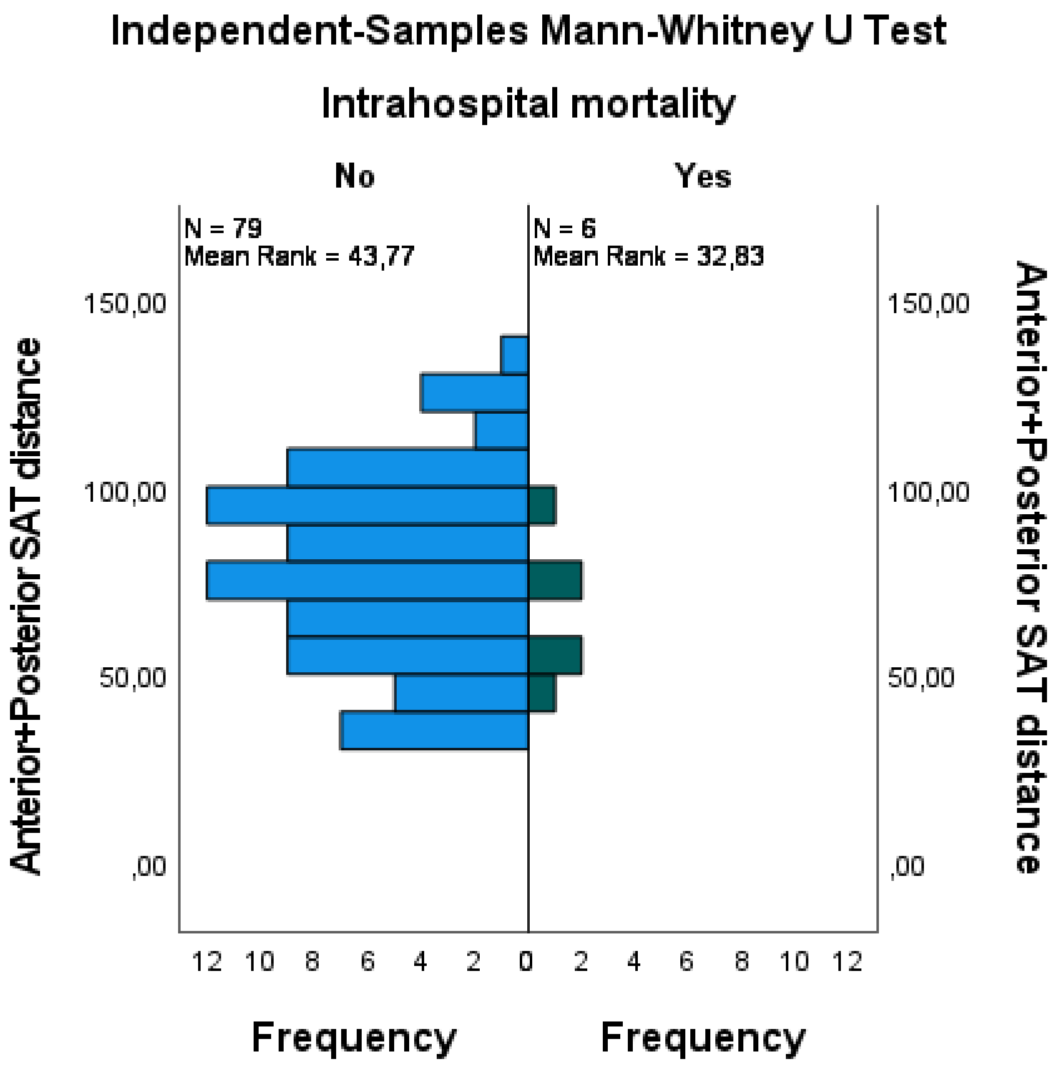

VAT distance across Intrahospital mortality

Independent-Samples Mann-Whitney U Test Summary

Total N	84
Mann-Whitney U	125,000
Wilcoxon W	146,000
Test Statistic	125,000
Standard Error	57,564
Standardized Test Statistic	-1,894

Asymptotic Sig.(2-sided test)	,058

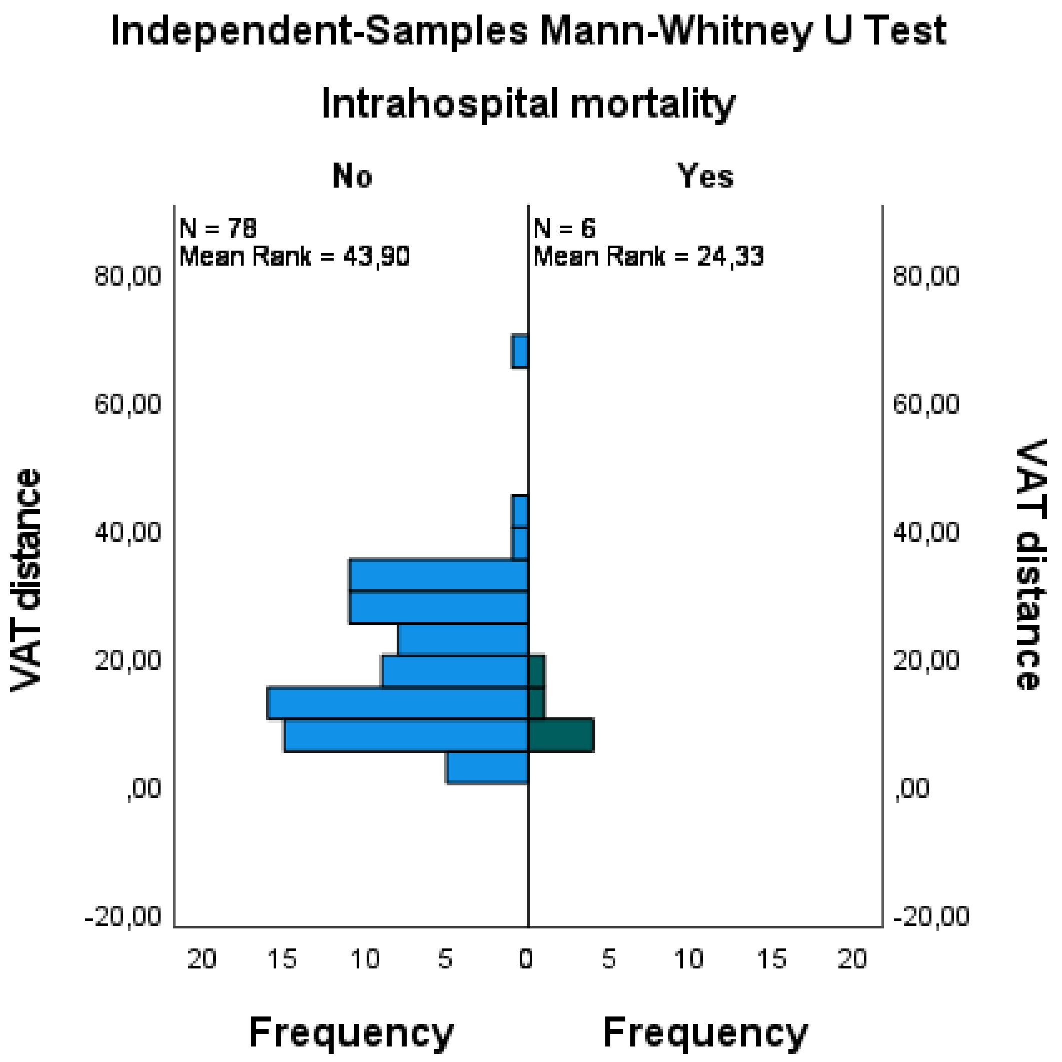

Right common femoral artery area (mm2) across Intrahospital mortality

Independent-Samples Mann-Whitney U Test Summary

Total N	85
Mann-Whitney U	151,500
Wilcoxon W	172,500
Test Statistic	151,500
Standard Error	58,274
Standardized Test Statistic	-1,467

Asymptotic Sig.(2-sided test)	,142

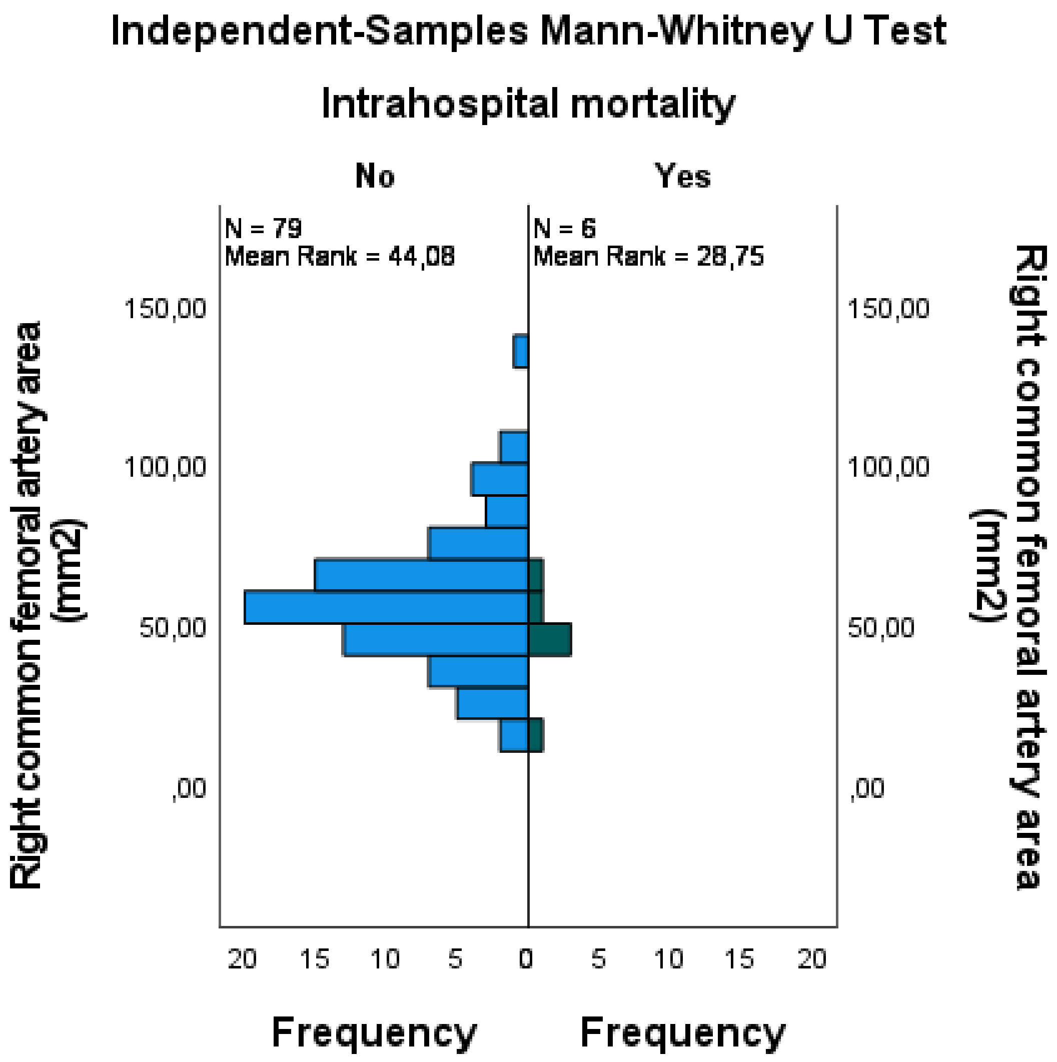

Left common femoral artery area (mm2) across Intrahospital mortality

Independent-Samples Mann-Whitney U Test Summary

Total N	85
Mann-Whitney U	164,500
Wilcoxon W	185,500
Test Statistic	164,500
Standard Error	58,270
Standardized Test Statistic	-1,244

Asymptotic Sig.(2-sided test)	,213

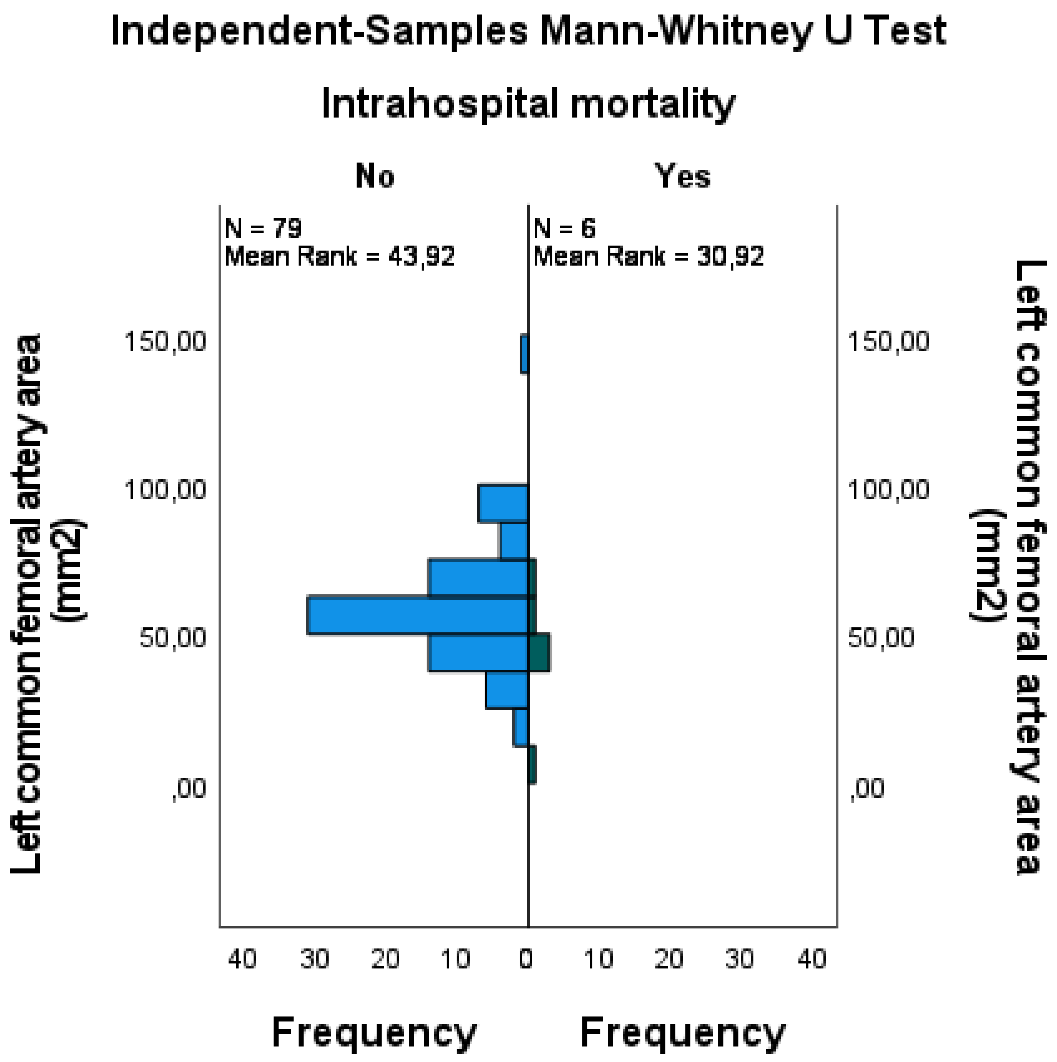

FAT area (cm2) across Intrahospital mortality

Independent-Samples Mann-Whitney U Test Summary

Total N	85
Mann-Whitney U	182,000
Wilcoxon W	203,000
Test Statistic	182,000
Standard Error	58,284
Standardized Test Statistic	-,944

Asymptotic Sig.(2-sided test)	,345

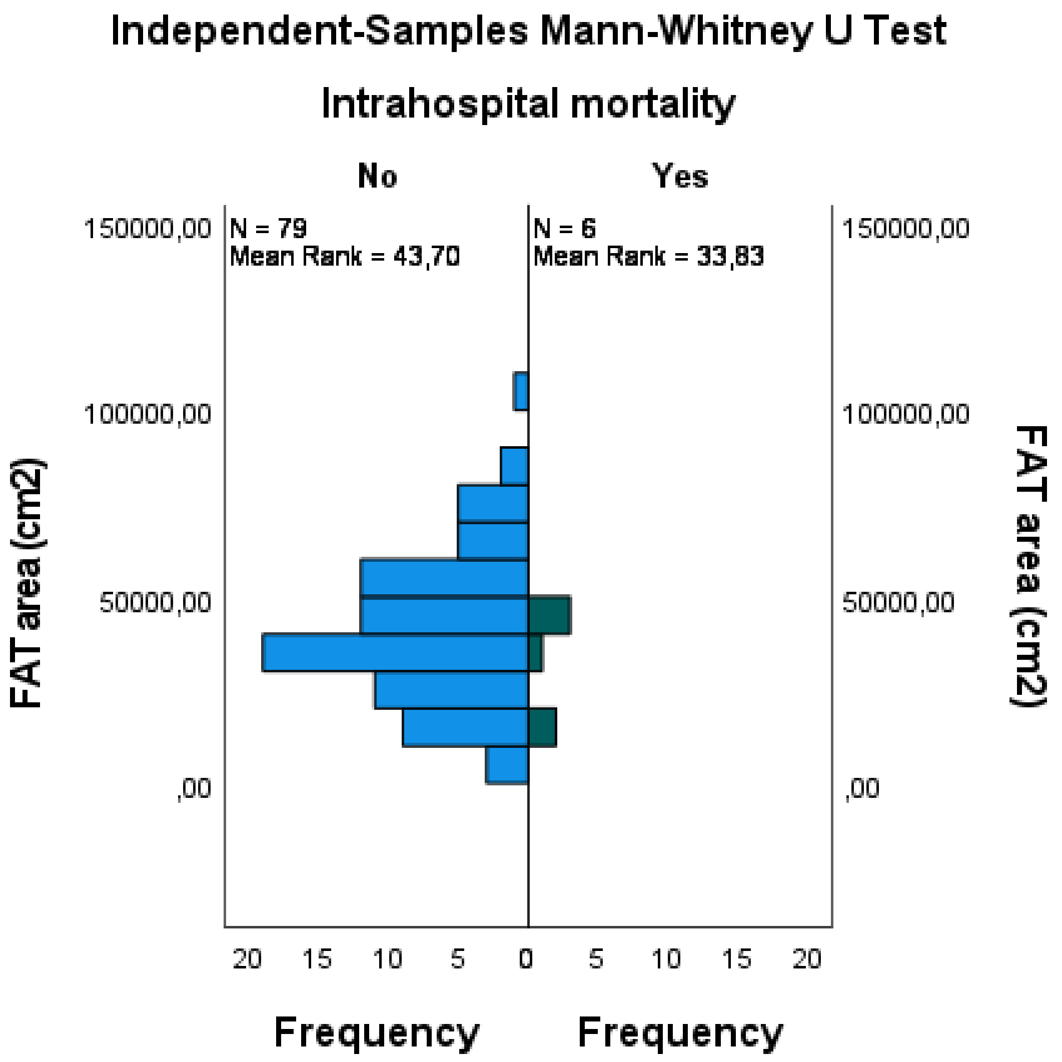

SAT area (cm2) across Intrahospital mortality

Independent-Samples Mann-Whitney U Test Summary

Total N	85
Mann-Whitney U	213,000
Wilcoxon W	234,000
Test Statistic	213,000
Standard Error	58,284
Standardized Test Statistic	-,412

Asymptotic Sig.(2-sided test)	,681

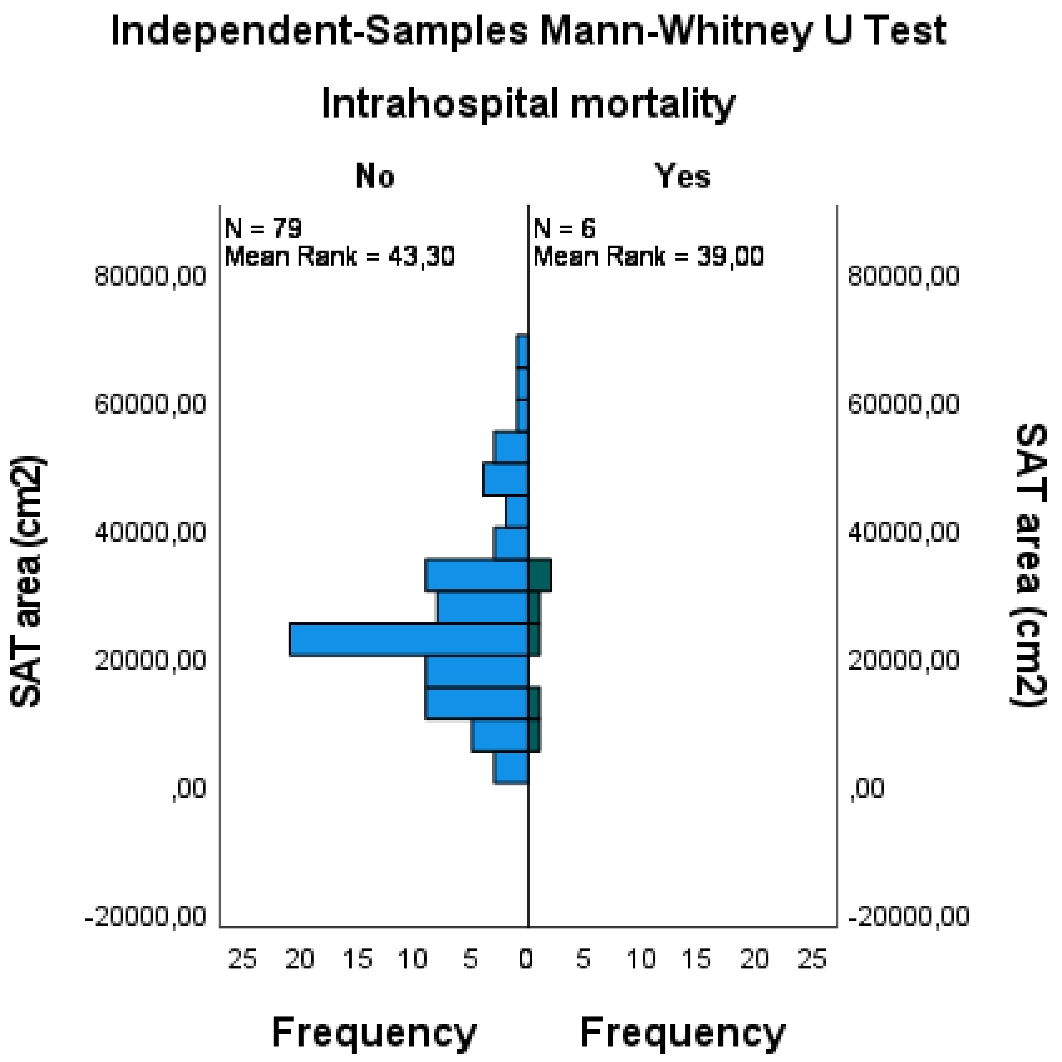

VAT area (cm2) across Intrahospital mortality

Independent-Samples Mann-Whitney U Test Summary

Total N	84
Mann-Whitney U	141,000
Wilcoxon W	162,000
Test Statistic	141,000
Standard Error	57,576
Standardized Test Statistic	-1,615

Asymptotic Sig.(2-sided test)	,106

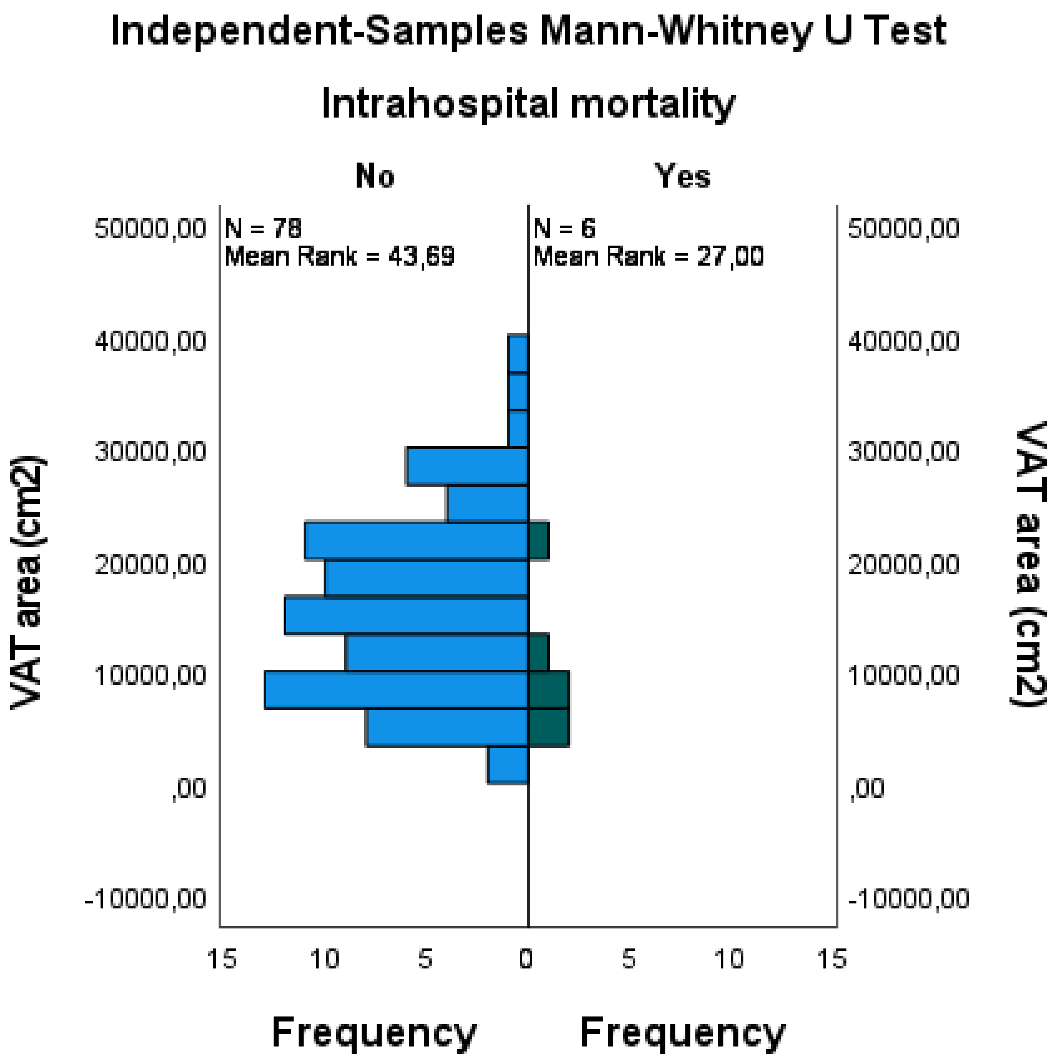

Right Psoas muscle area (cm2) across Intrahospital mortality

Independent-Samples Mann-Whitney U Test Summary

Total N	85
Mann-Whitney U	177,000
Wilcoxon W	198,000
Test Statistic	177,000
Standard Error	58,282

Standardized Test Statistic	-1,029
Asymptotic Sig.(2-sided test)	,303

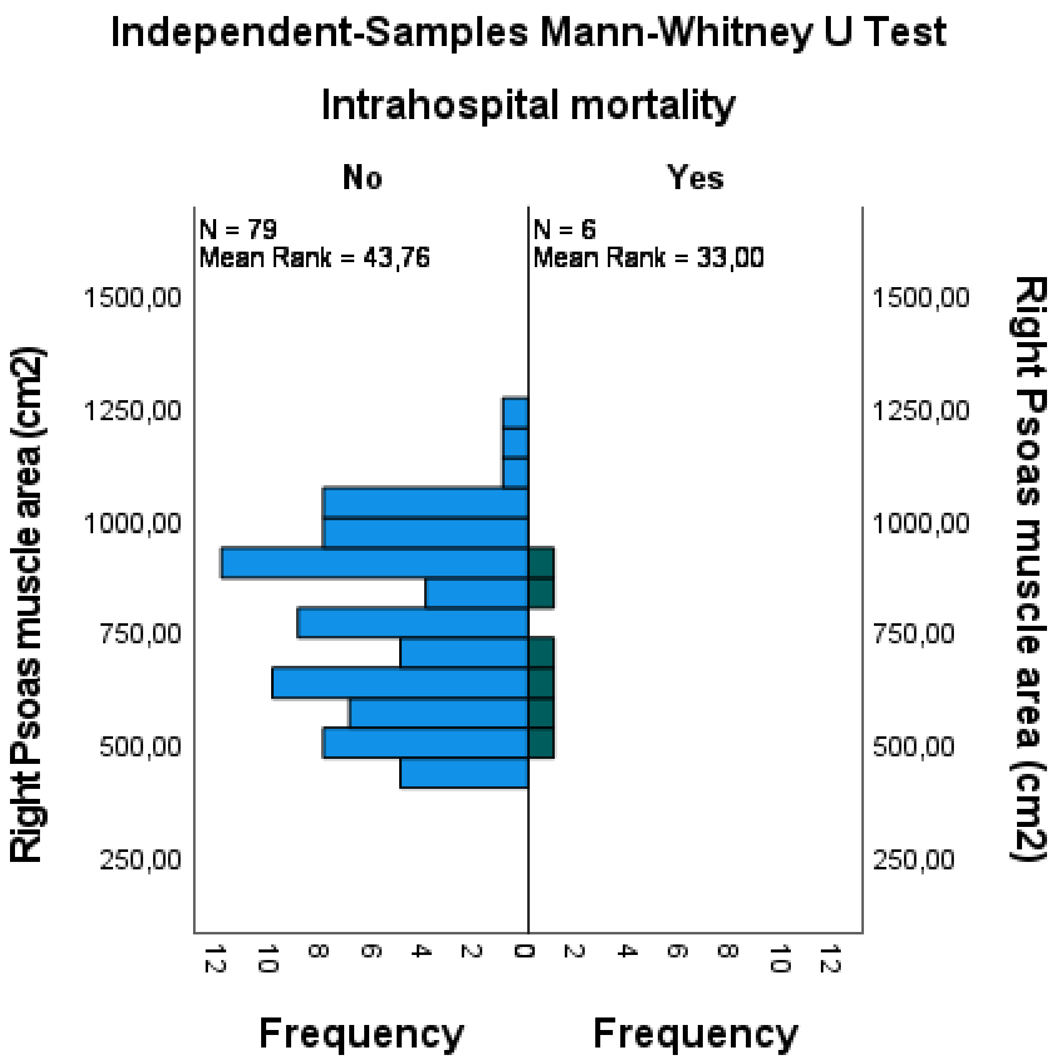

Left Psoas muscle area (cm2) across Intrahospital mortality

Independent-Samples Mann-Whitney U Test Summary

Total N	85
Mann-Whitney U	184,000
Wilcoxon W	205,000
Test Statistic	184,000
Standard Error	58,283

Standardized Test Statistic	-,909
Asymptotic Sig.(2-sided test)	,363

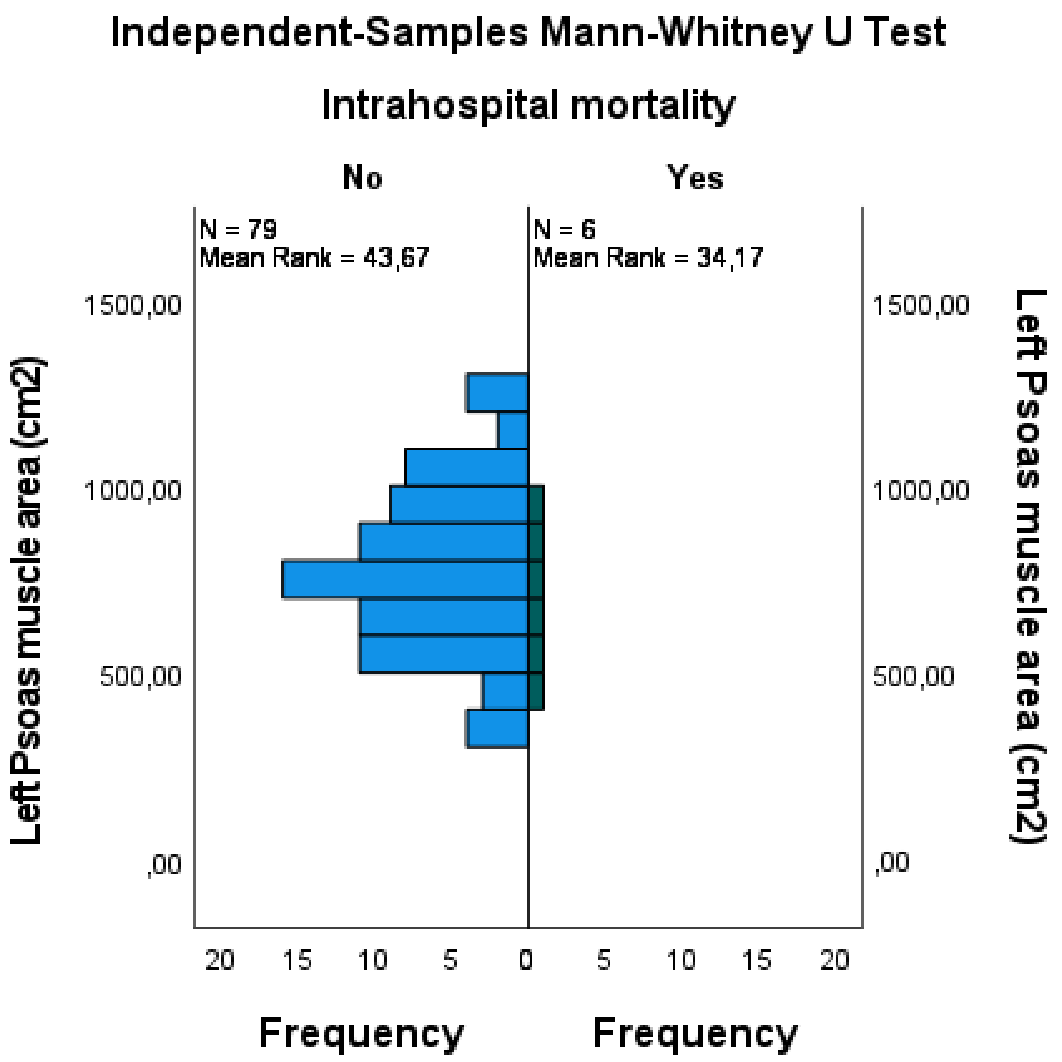

FAT mean density (HU) across Intrahospital mortality

Independent-Samples Mann-Whitney U Test Summary

Total N	85
Mann-Whitney U	332,000
Wilcoxon W	353,000
Test Statistic	332,000
Standard Error	58,274

Standardized Test Statistic	1,630
Asymptotic Sig.(2-sided test)	,103

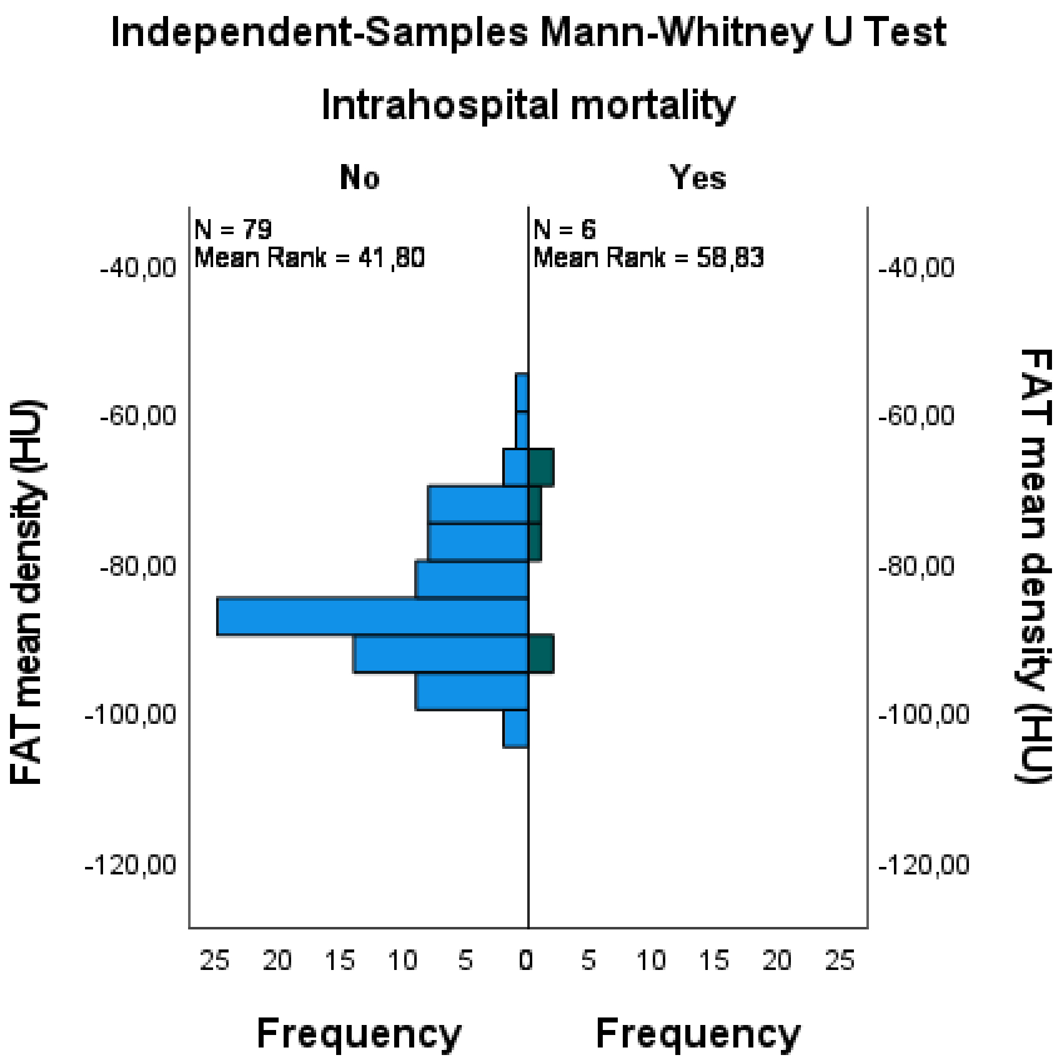

FAT median density (HU) across Intrahospital mortality

Independent-Samples Mann-Whitney U Test Summary

Total N	85
Mann-Whitney U	339,500
Wilcoxon W	360,500
Test Statistic	339,500
Standard Error	58,213

Standardized Test Statistic	1,761
Asymptotic Sig.(2-sided test)	,078

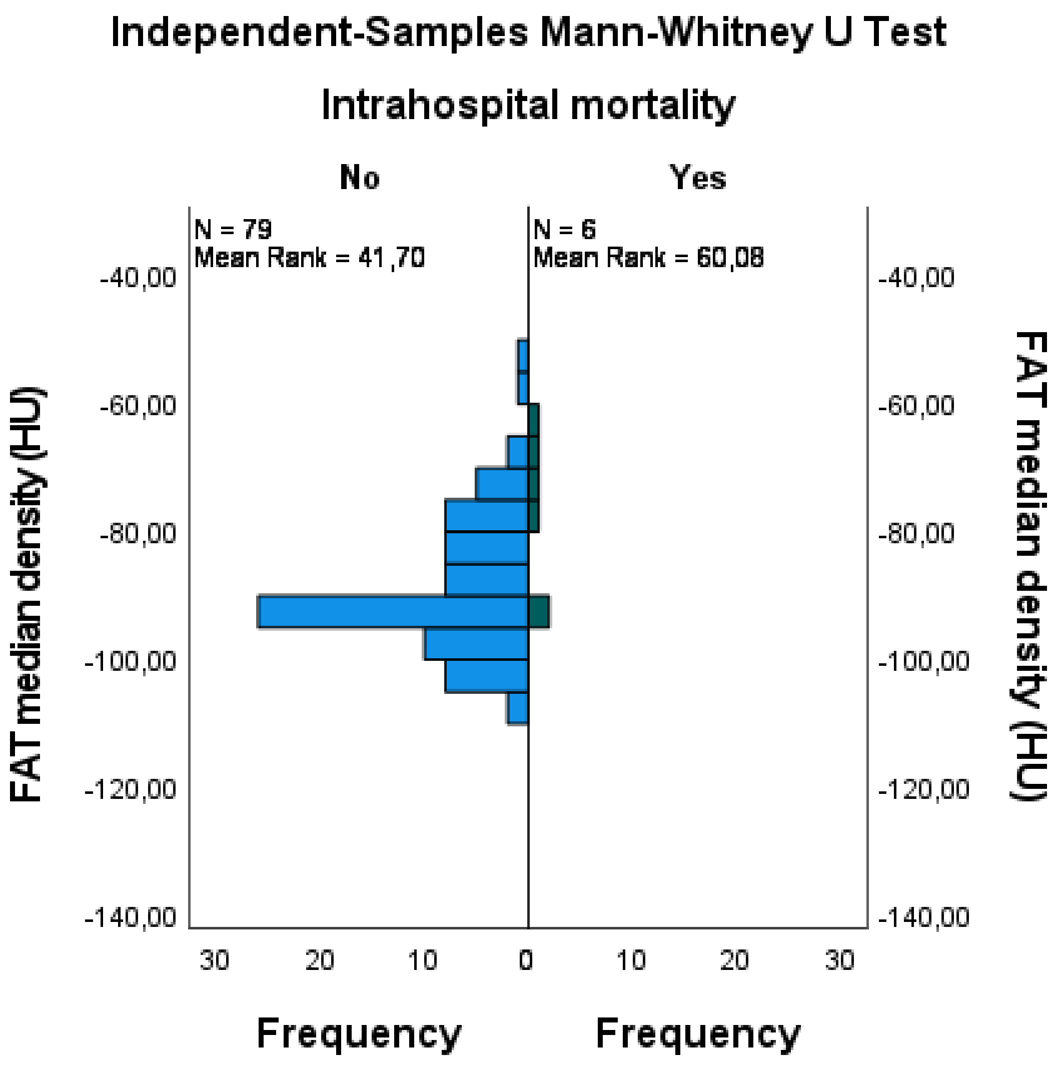

FAT density standard deviation across Intrahospital mortality

Independent-Samples Mann-Whitney U Test Summary

Total N	85
Mann-Whitney U	150,000
Wilcoxon W	171,000
Test Statistic	150,000
Standard Error	58,284

Standardized Test Statistic	-1,493
Asymptotic Sig.(2-sided test)	,136

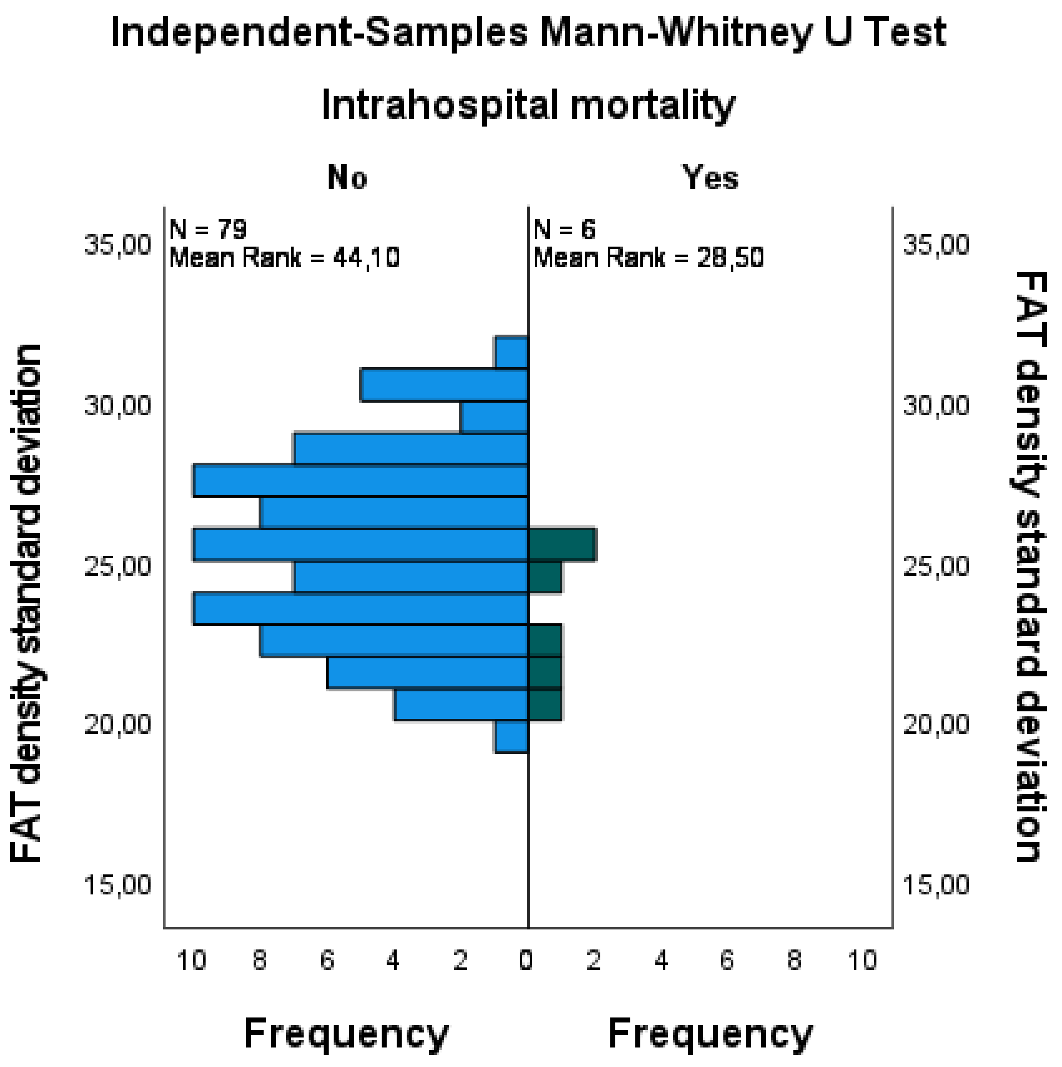

SAT mean density (HU) across Intrahospital mortality

Independent-Samples Mann-Whitney U Test Summary

Total N	85
Mann-Whitney U	322,000
Wilcoxon W	343,000
Test Statistic	322,000
Standard Error	58,277

Standardized Test Statistic	1,459
Asymptotic Sig.(2-sided test)	,145

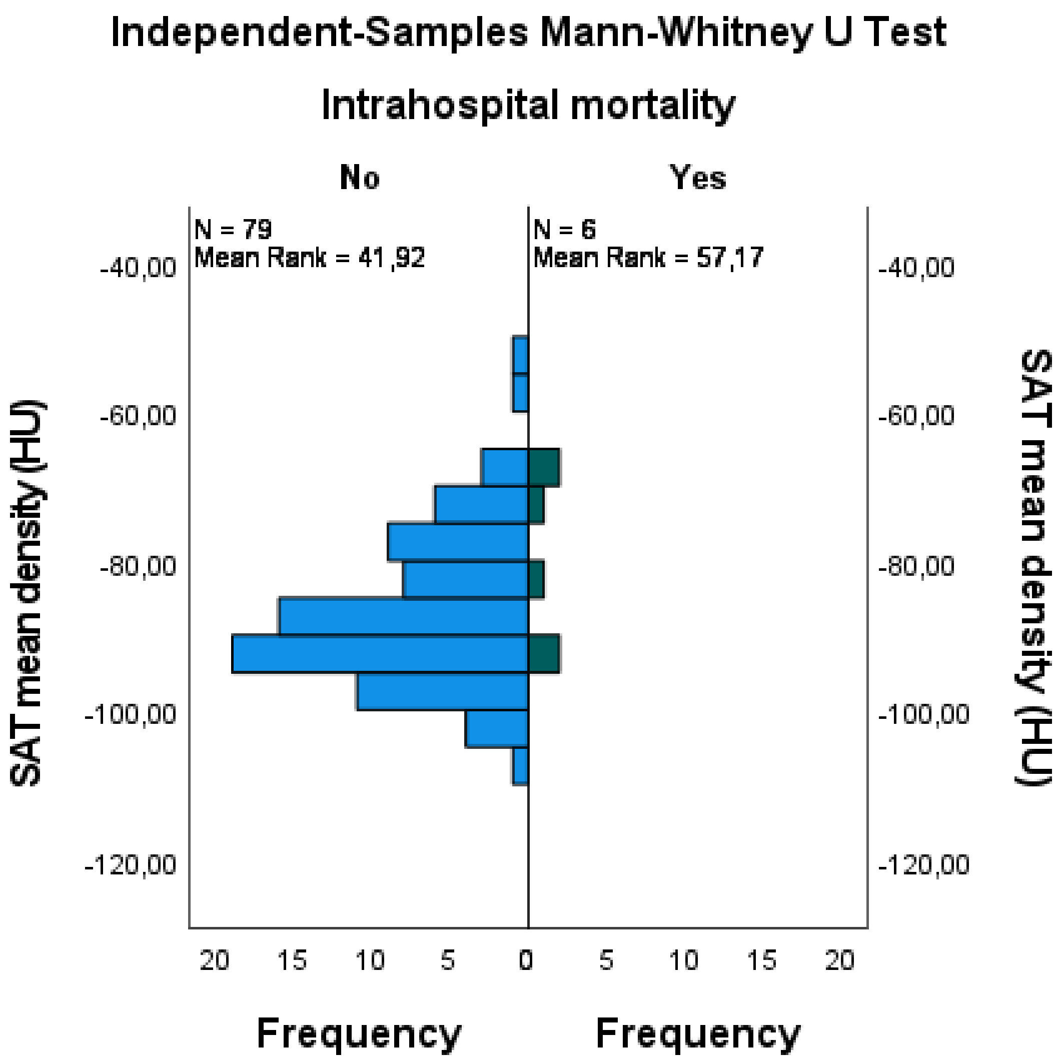

SAT median density (HU) across Intrahospital mortality

Independent-Samples Mann-Whitney U Test Summary

Total N	83
Mann-Whitney U	311,500
Wilcoxon W	332,500
Test Statistic	311,500
Standard Error	56,824

Standardized Test Statistic	1,417
Asymptotic Sig.(2-sided test)	,157

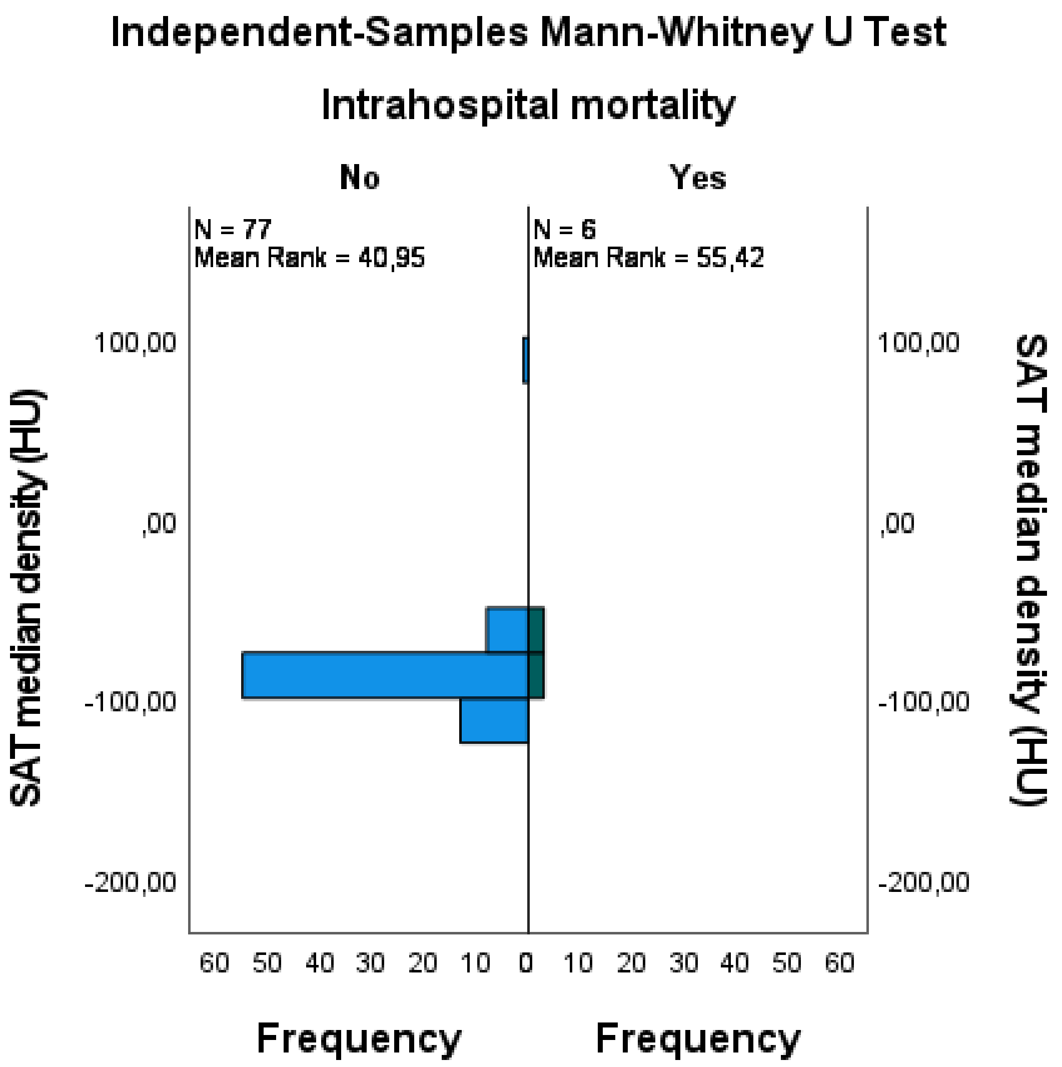

SAT density standard deviation across Intrahospital mortality

Independent-Samples Mann-Whitney U Test Summary

Total N	84
Mann-Whitney U	157,000
Wilcoxon W	178,000
Test Statistic	157,000
Standard Error	57,576

Standardized Test Statistic	-1,337
Asymptotic Sig.(2-sided test)	,181

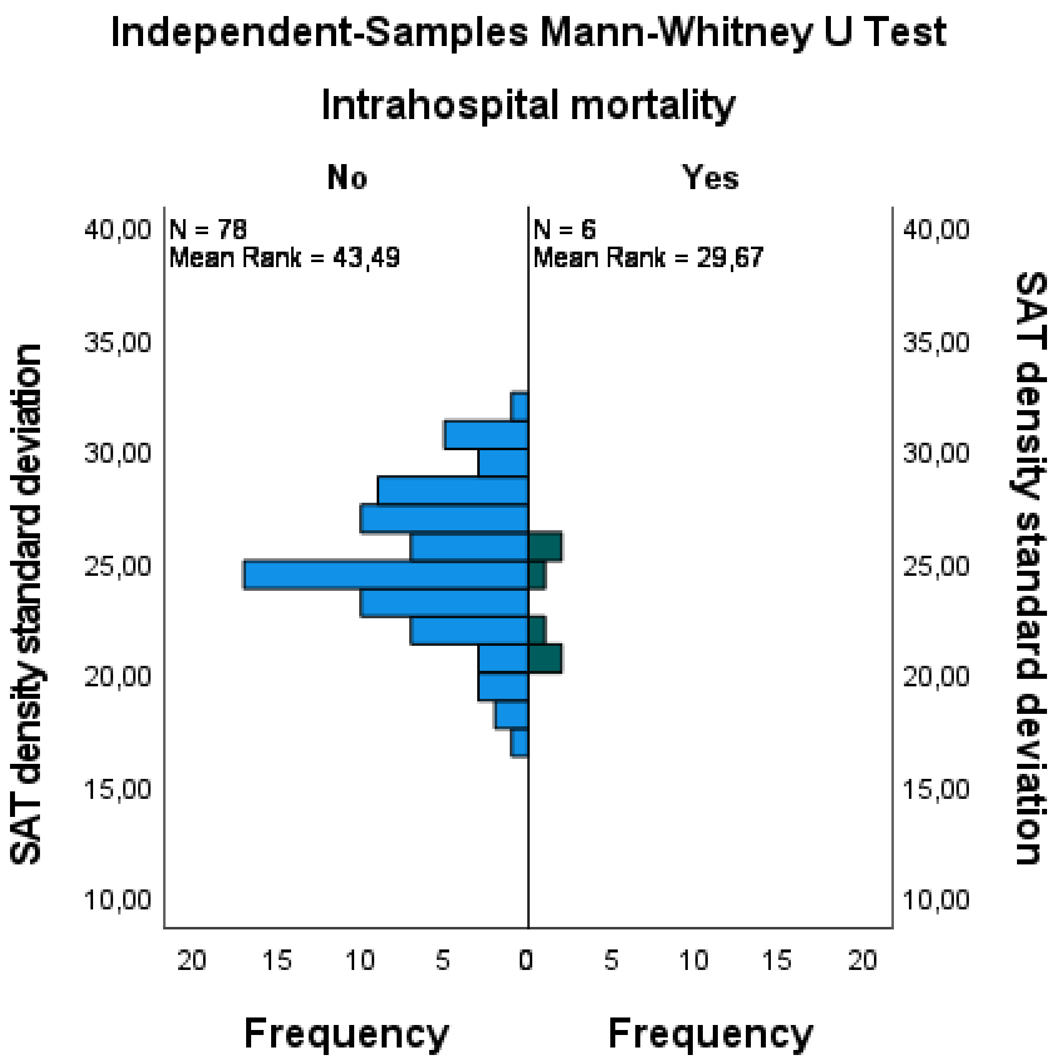

VAT mean density (HU) across Intrahospital mortality

Independent-Samples Mann-Whitney U Test Summary

Total N	85
Mann-Whitney U	391,000
Wilcoxon W	412,000
Test Statistic	391,000
Standard Error	58,273

Standardized Test Statistic	2,643
Asymptotic Sig.(2-sided test)	,008

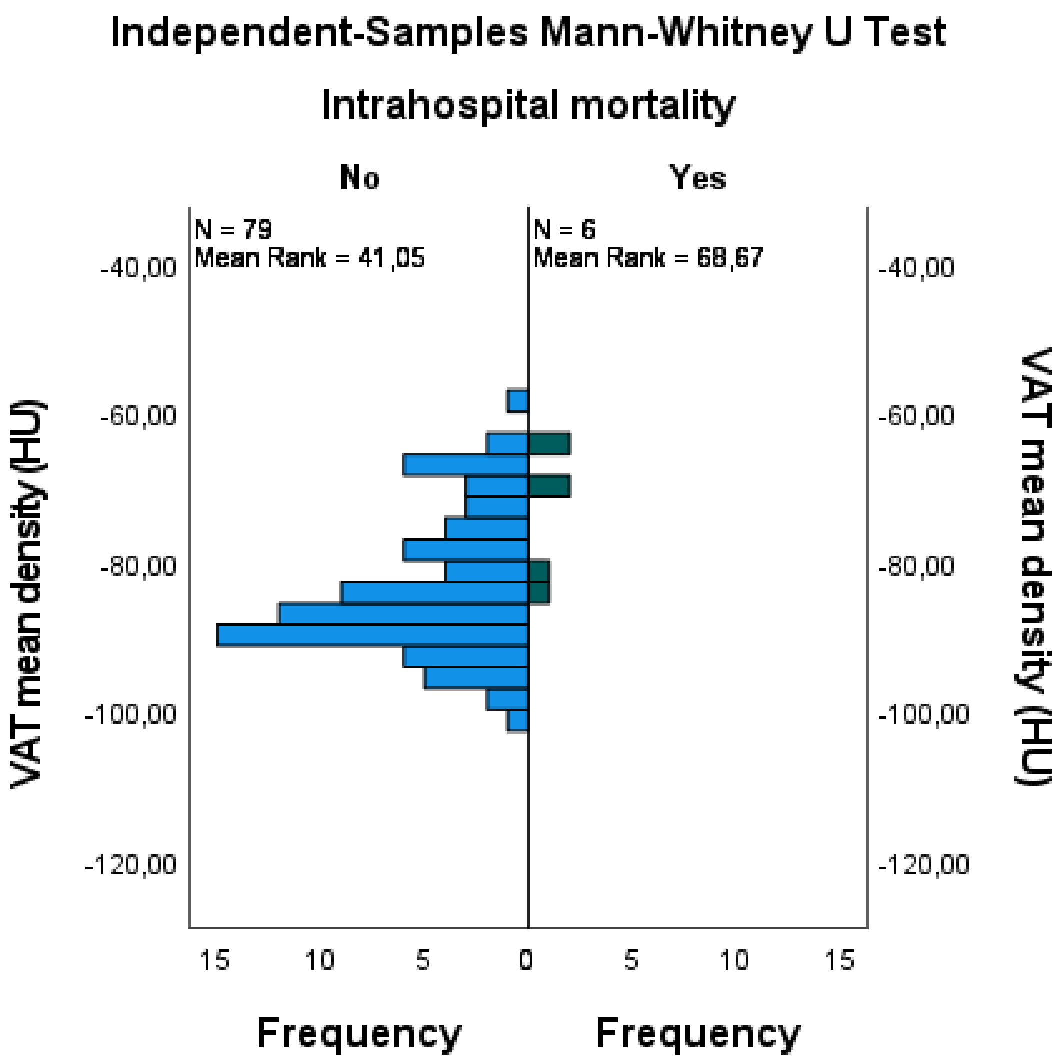

VAT median density (HU) across Intrahospital mortality

Independent-Samples Mann-Whitney U Test Summary

Total N	85
Mann-Whitney U	386,000
Wilcoxon W	407,000
Test Statistic	386,000
Standard Error	58,211

Standardized Test Statistic	2,560
Asymptotic Sig.(2-sided test)	,010

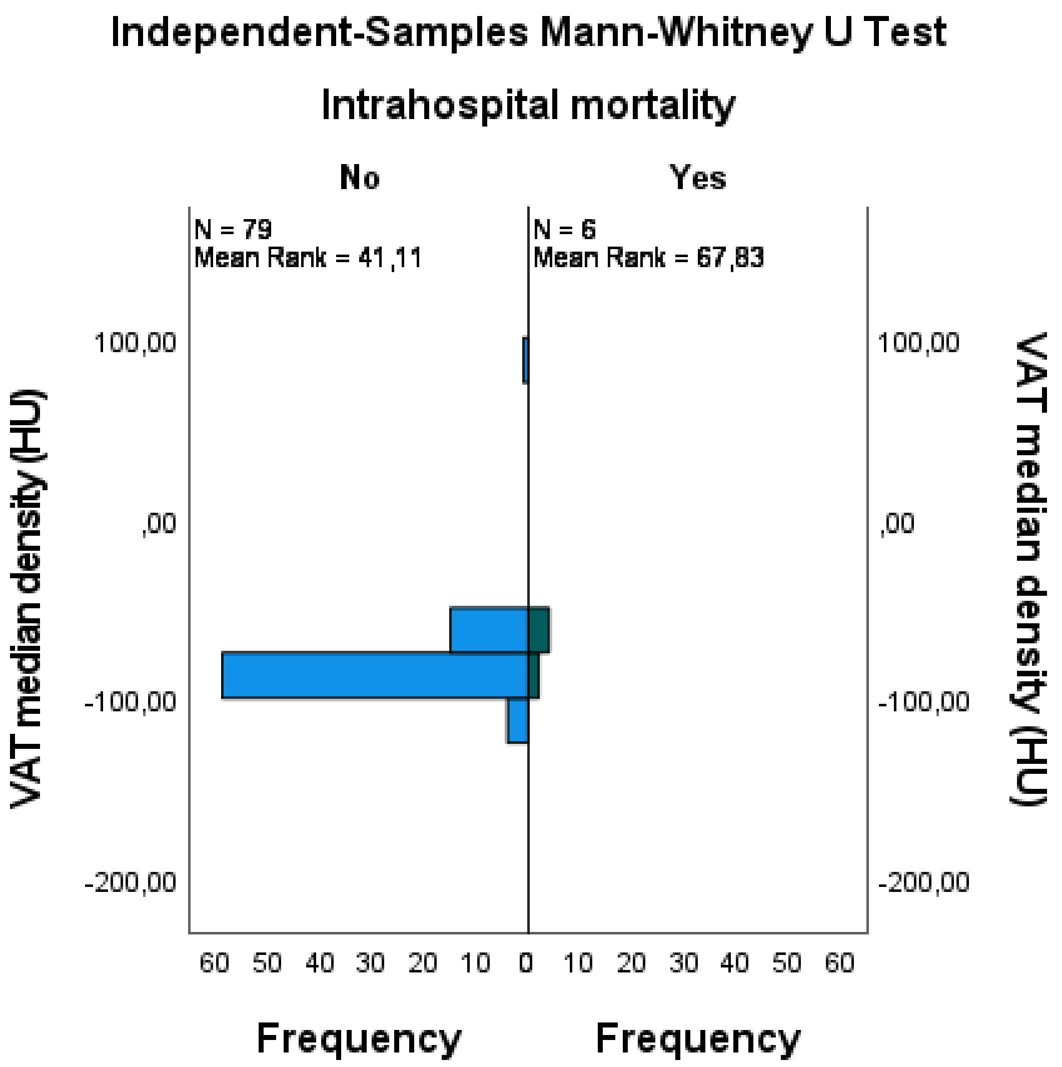

VAT density standard deviation across Intrahospital mortality

Independent-Samples Mann-Whitney U Test Summary	
Total N	84
Mann-Whitney U	154,000
Wilcoxon W	175,000
Test Statistic	154,000
Standard Error	57,576

Standardized Test Statistic	-1,389
Asymptotic Sig.(2-sided test)	,165

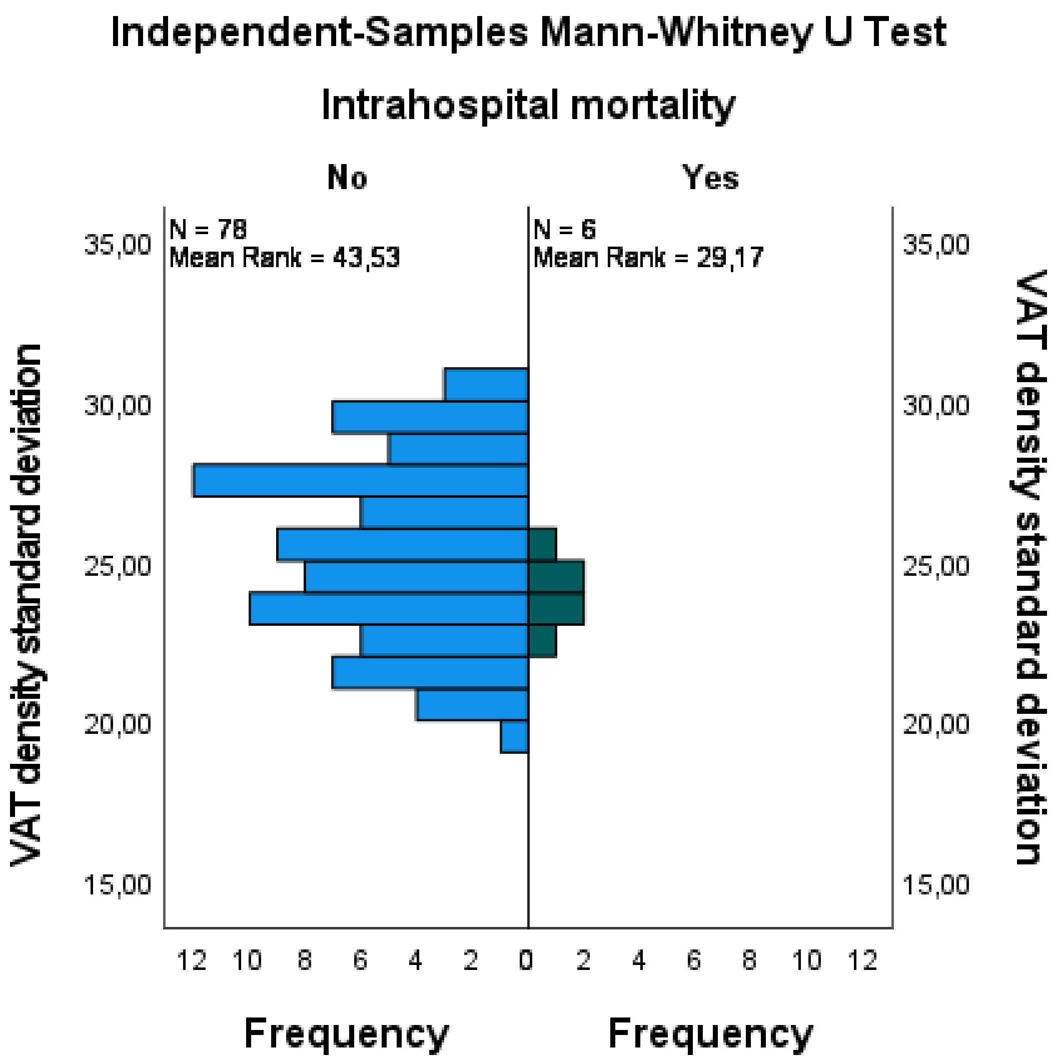

Right Psoas muscle mean density (HU) across Intrahospital mortality

Independent-Samples Mann-Whitney U Test Summary

Total N	85
Mann-Whitney U	317,000
Wilcoxon W	338,000
Test Statistic	317,000
Standard Error	58,271

Standardized Test Statistic	1,373
Asymptotic Sig.(2-sided test)	,170

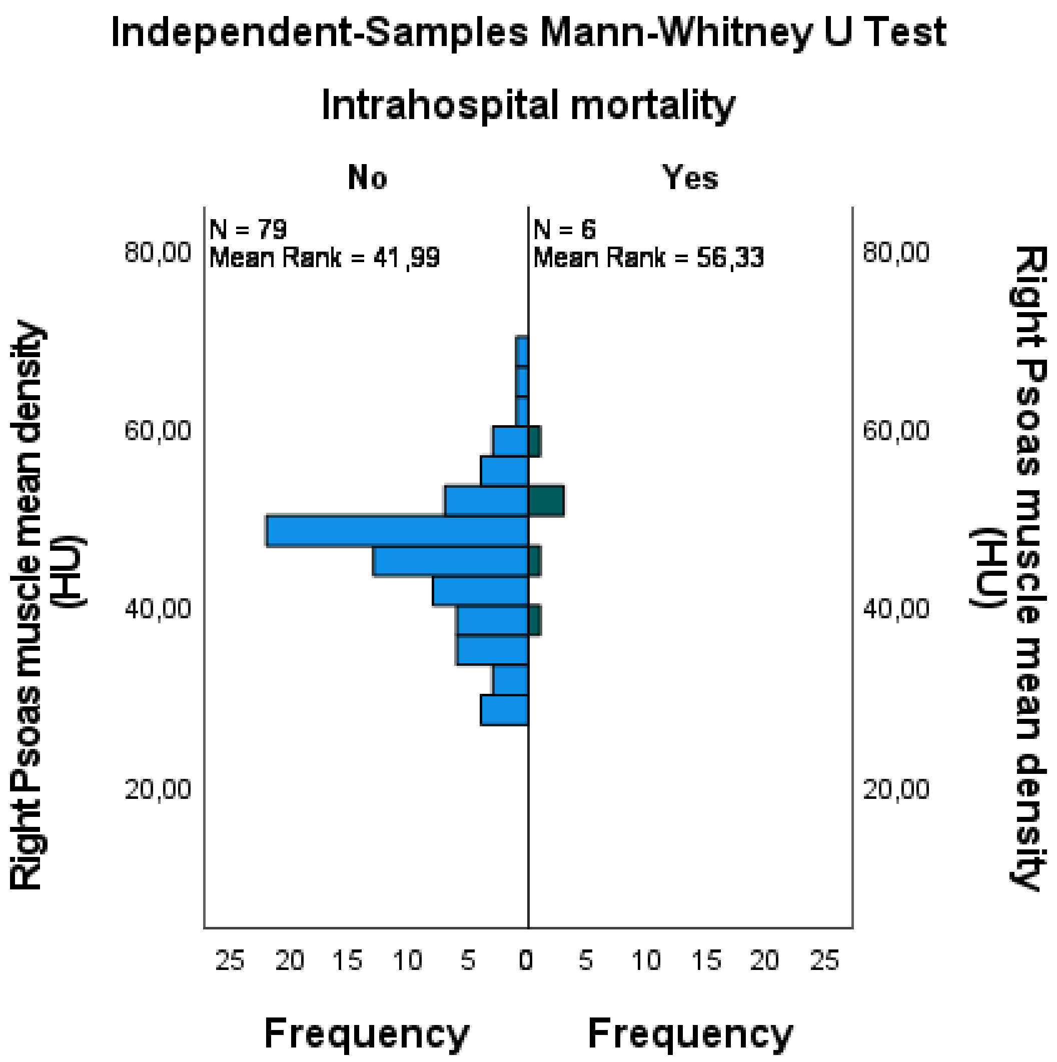

Right Psoas muscle median density (HU) across Intrahospital mortality

Independent-Samples Mann-Whitney U Test Summary

Total N	85
Mann-Whitney U	316,500
Wilcoxon W	337,500
Test Statistic	316,500
Standard Error	58,229

Standardized Test Statistic	1,365
Asymptotic Sig.(2-sided test)	,172

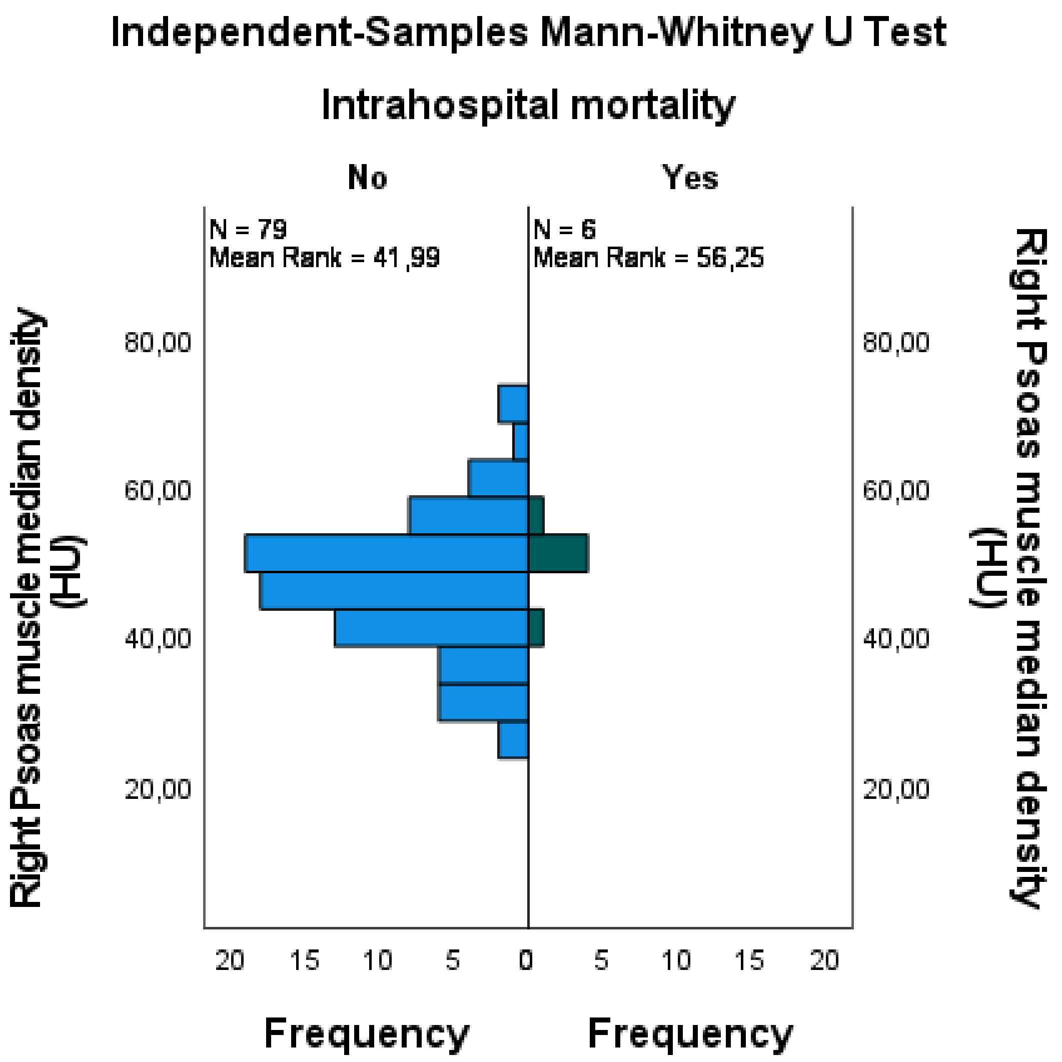

Right Psoas muscle density standard deviation across Intrahospital mortality

Independent-Samples Mann-Whitney U Test Summary

Total N	85
Mann-Whitney U	172,000
Wilcoxon W	193,000
Test Statistic	172,000
Standard Error	58,283

Standardized Test Statistic	-1,115
Asymptotic Sig.(2-sided test)	,265

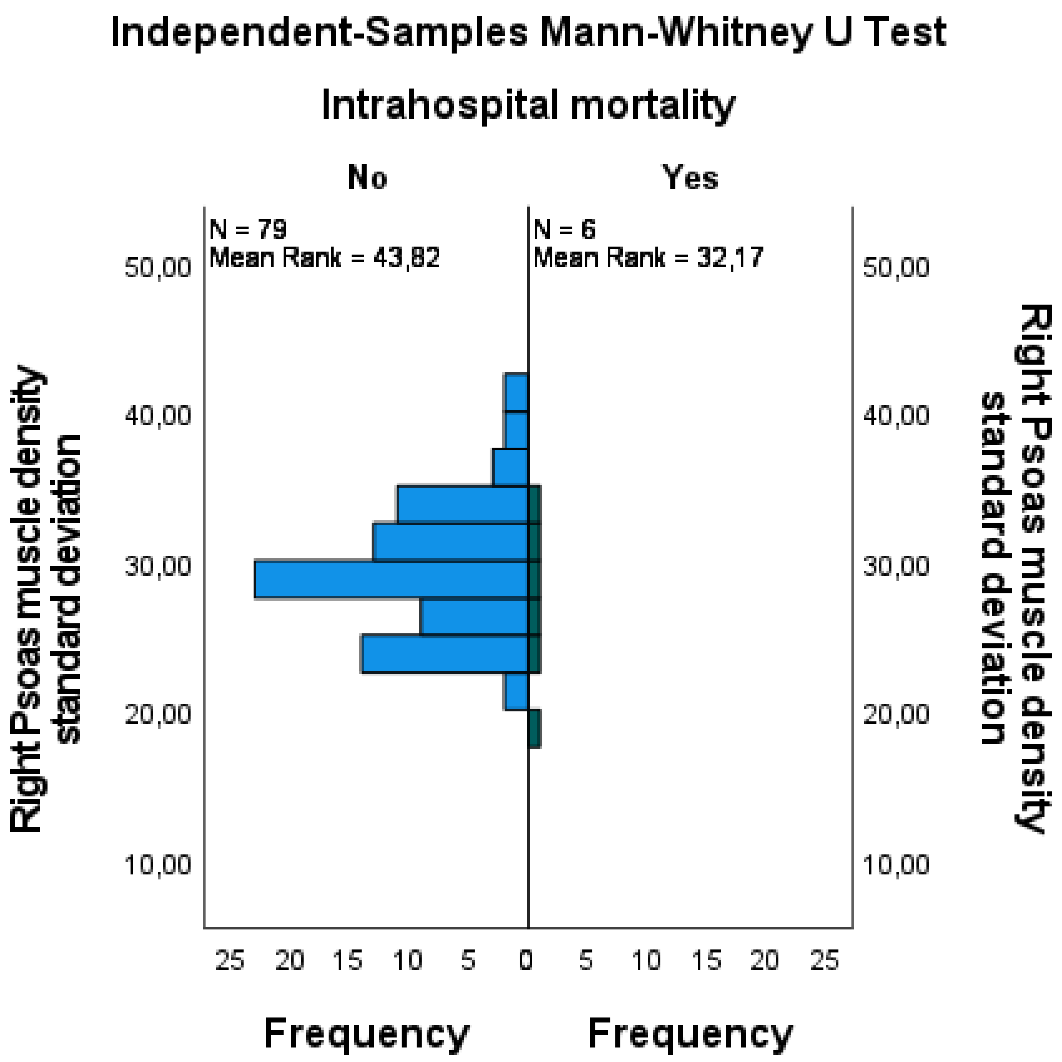

Left Psoas muscle mean density (HU) across Intrahospital mortality

Independent-Samples Mann-Whitney U Test Summary

Total N	85
Mann-Whitney U	272,000
Wilcoxon W	293,000
Test Statistic	272,000
Standard Error	58,272

Standardized Test Statistic	,601
Asymptotic Sig.(2-sided test)	,548

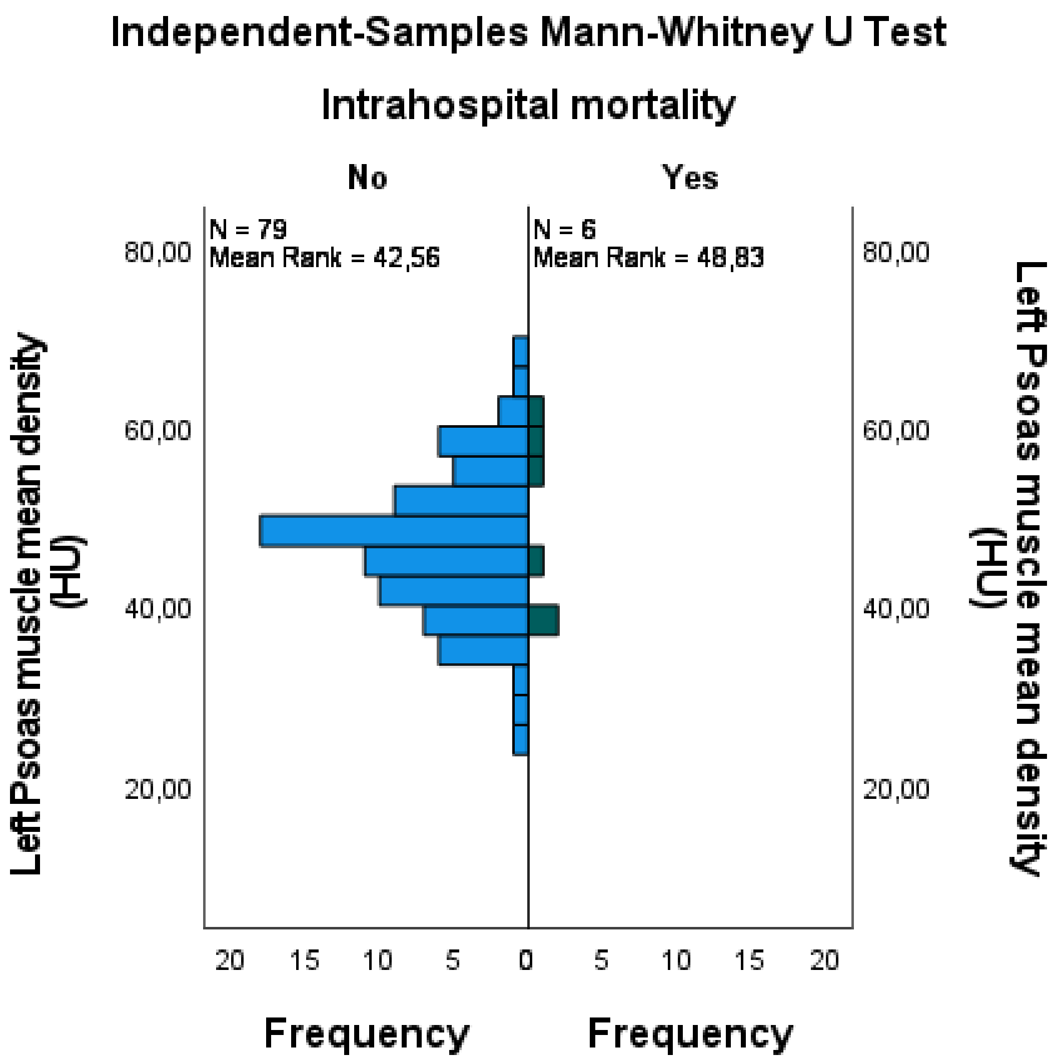

Left Psoas muscle median density (HU) across Intrahospital mortality

Independent-Samples Mann-Whitney U Test Summary

Total N	85
Mann-Whitney U	283,000
Wilcoxon W	304,000
Test Statistic	283,000
Standard Error	58,209

Standardized Test Statistic	,790
Asymptotic Sig.(2-sided test)	,429

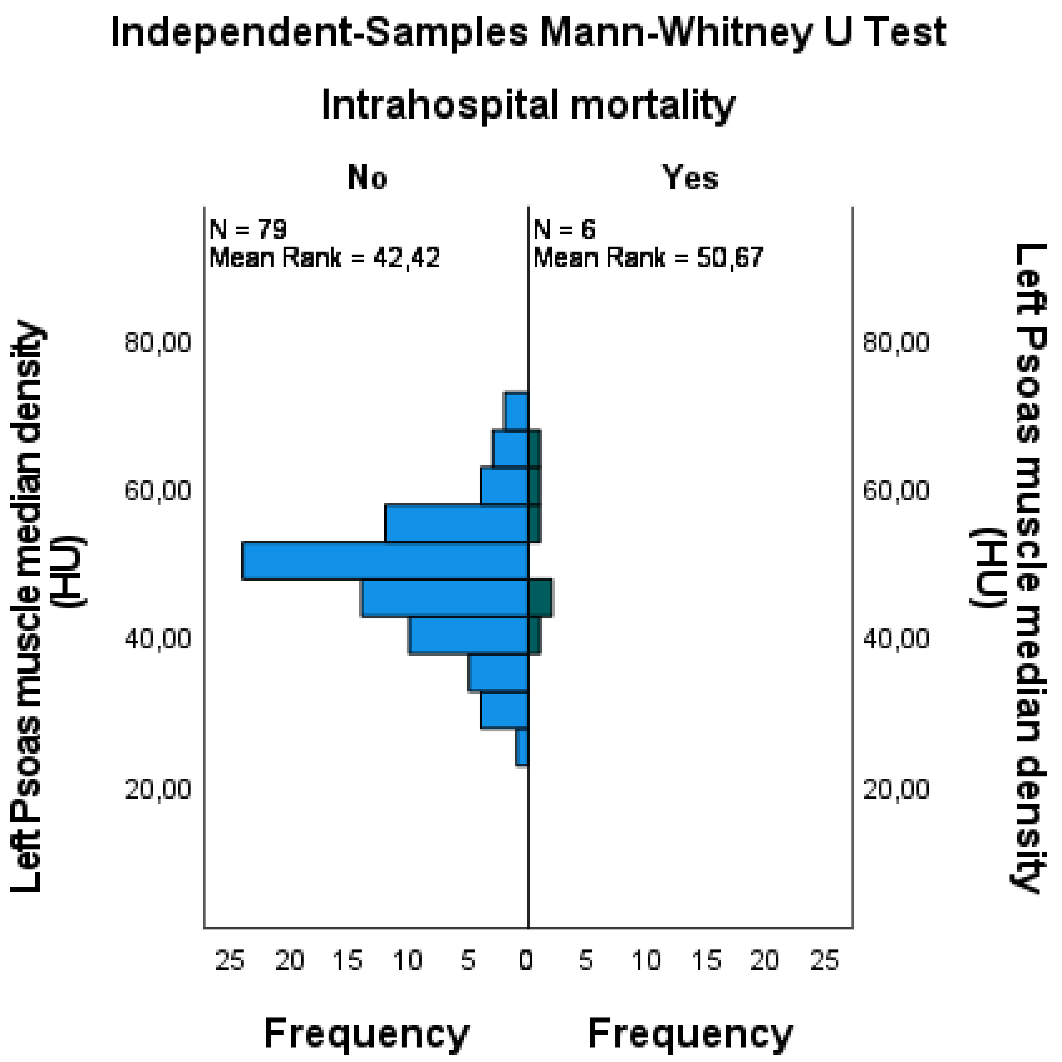

Left Psoas muscle density standard deviation across Intrahospital mortality

Independent-Samples Mann-Whitney U Test Summary

Total N	85
Mann-Whitney U	154,000
Wilcoxon W	175,000
Test Statistic	154,000
Standard Error	58,284

Standardized Test Statistic	-1,424
Asymptotic Sig.(2-sided test)	,154

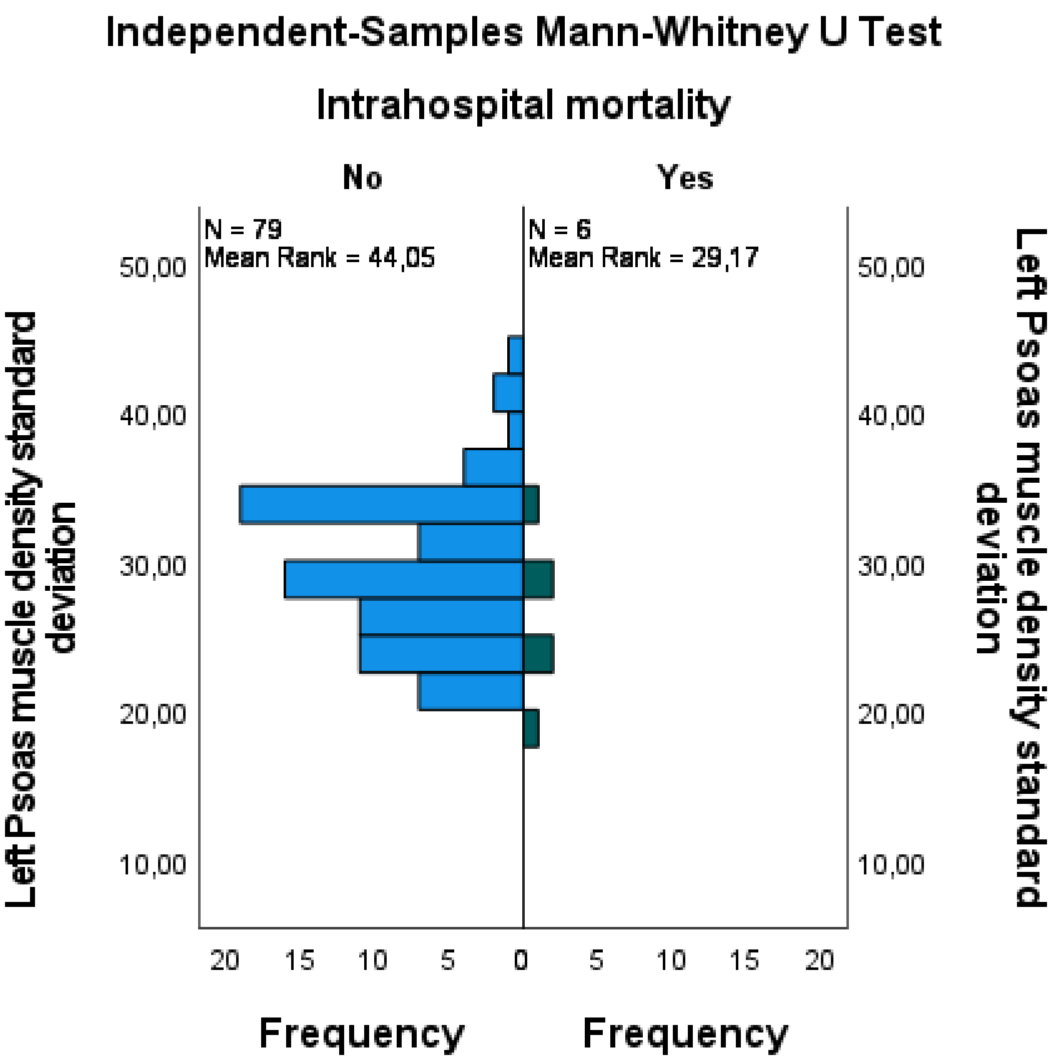

Independent-Samples Mann-Whitney U Test for femoral stent placement

Hypothesis Test Summary

	Null Hypothesis	Test	Sig.[a,b]	Decision
1	The distribution of Psoas/height is the same across categories of Femoral stent placement.	Independent-Samples Mann-Whitney U Test	,489	Retain the null hypothesis.
2	The distribution of Anterior SAT distance is the same across categories of Femoral stent placement.	Independent-Samples Mann-Whitney U Test	,547	Retain the null hypothesis.
3	The distribution of Posterior SAT distance is the same across categories of Femoral stent placement.	Independent-Samples Mann-Whitney U Test	,246	Retain the null hypothesis.
4	The distribution of Anterior+Posterior SAT distance is the same across categories of Femoral stent placement.	Independent-Samples Mann-Whitney U Test	,412	Retain the null hypothesis.
5	The distribution of VAT distance is the same across categories of Femoral stent placement.	Independent-Samples Mann-Whitney U Test	,493	Retain the null hypothesis.
6	The distribution of Right common femoral artery area (mm2) is the same across categories of Femoral stent placement.	Independent-Samples Mann-Whitney U Test	,157	Retain the null hypothesis.
7	The distribution of Left common femoral artery area (mm2) is the same across categories of Femoral stent placement.	Independent-Samples Mann-Whitney U Test	,220	Retain the null hypothesis.
8	The distribution of FAT area (cm2) is the same across categories of Femoral stent placement.	Independent-Samples Mann-Whitney U Test	,810	Retain the null hypothesis.

9	The distribution of SAT area (cm2) is the same across categories of Femoral stent placement.	Independent-Samples Mann-Whitney U Test	,583	Retain the null hypothesis.
10	The distribution of VAT area (cm2) is the same across categories of Femoral stent placement.	Independent-Samples Mann-Whitney U Test	,604	Retain the null hypothesis.
11	The distribution of Right Psoas muscle area (cm2) is the same across categories of Femoral stent placement.	Independent-Samples Mann-Whitney U Test	,054	Retain the null hypothesis.
12	The distribution of Left Psoas muscle area (cm2) is the same across categories of Femoral stent placement.	Independent-Samples Mann-Whitney U Test	,098	Retain the null hypothesis.
13	The distribution of FAT mean density (HU) is the same across categories of Femoral stent placement.	Independent-Samples Mann-Whitney U Test	,976	Retain the null hypothesis.
14	The distribution of FAT median density (HU) is the same across categories of Femoral stent placement.	Independent-Samples Mann-Whitney U Test	,946	Retain the null hypothesis.
15	The distribution of FAT density standard deviation is the same across categories of Femoral stent placement.	Independent-Samples Mann-Whitney U Test	,821	Retain the null hypothesis.
16	The distribution of SAT mean density (HU) is the same across categories of Femoral stent placement.	Independent-Samples Mann-Whitney U Test	,851	Retain the null hypothesis.
17	The distribution of SAT median density (HU) is the same across categories of Femoral stent placement.	Independent-Samples Mann-Whitney U Test	,902	Retain the null hypothesis.
18	The distribution of SAT density standard deviation is the same across categories of Femoral stent placement.	Independent-Samples Mann-Whitney U Test	,648	Retain the null hypothesis.

19	The distribution of VAT mean density (HU) is the same across categories of Femoral stent placement.	Independent-Samples Mann-Whitney U Test	,976	Retain the null hypothesis.
20	The distribution of VAT median density (HU) is the same across categories of Femoral stent placement.	Independent-Samples Mann-Whitney U Test	,862	Retain the null hypothesis.
21	The distribution of VAT density standard deviation is the same across categories of Femoral stent placement.	Independent-Samples Mann-Whitney U Test	,044	Reject the null hypothesis.
22	The distribution of Right Psoas muscle mean density (HU) is the same across categories of Femoral stent placement.	Independent-Samples Mann-Whitney U Test	,032	Reject the null hypothesis.
23	The distribution of Right Psoas muscle median density (HU) is the same across categories of Femoral stent placement.	Independent-Samples Mann-Whitney U Test	,096	Retain the null hypothesis.
24	The distribution of Right Psoas muscle density standard deviation is the same across categories of Femoral stent placement.	Independent-Samples Mann-Whitney U Test	,718	Retain the null hypothesis.
25	The distribution of Left Psoas muscle mean density (HU) is the same across categories of Femoral stent placement.	Independent-Samples Mann-Whitney U Test	,073	Retain the null hypothesis.
26	The distribution of Left Psoas muscle median density (HU) is the same across categories of Femoral stent placement.	Independent-Samples Mann-Whitney U Test	,128	Retain the null hypothesis.
27	The distribution of Left Psoas muscle density standard deviation is the same across categories of Femoral stent placement.	Independent-Samples Mann-Whitney U Test	,578	Retain the null hypothesis.

a. The significance level is ,050.

b. Asymptotic significance is displayed.

In this case, the hypothesis of equal medians ($p < 0.05$) is rejected only for the variables

VAT density standard deviation and Right Psoas muscle mean density (HU), while for the others the null hypothesis is accepted ($p > 0.05$).
(The tables and graphs below are the details of the tests in this table: I have highlighted what things you should eventually report, namely test statistic and pvalue).

Psoas/height across Femoral stent placement

Independent-Samples Mann-Whitney U Test Summary

Total N	85
Mann-Whitney U	262,000
Wilcoxon W	298,000
Test Statistic	262,000
Standard Error	66,443
Standardized Test Statistic	-,692
Asymptotic Sig.(2-sided test)	,489

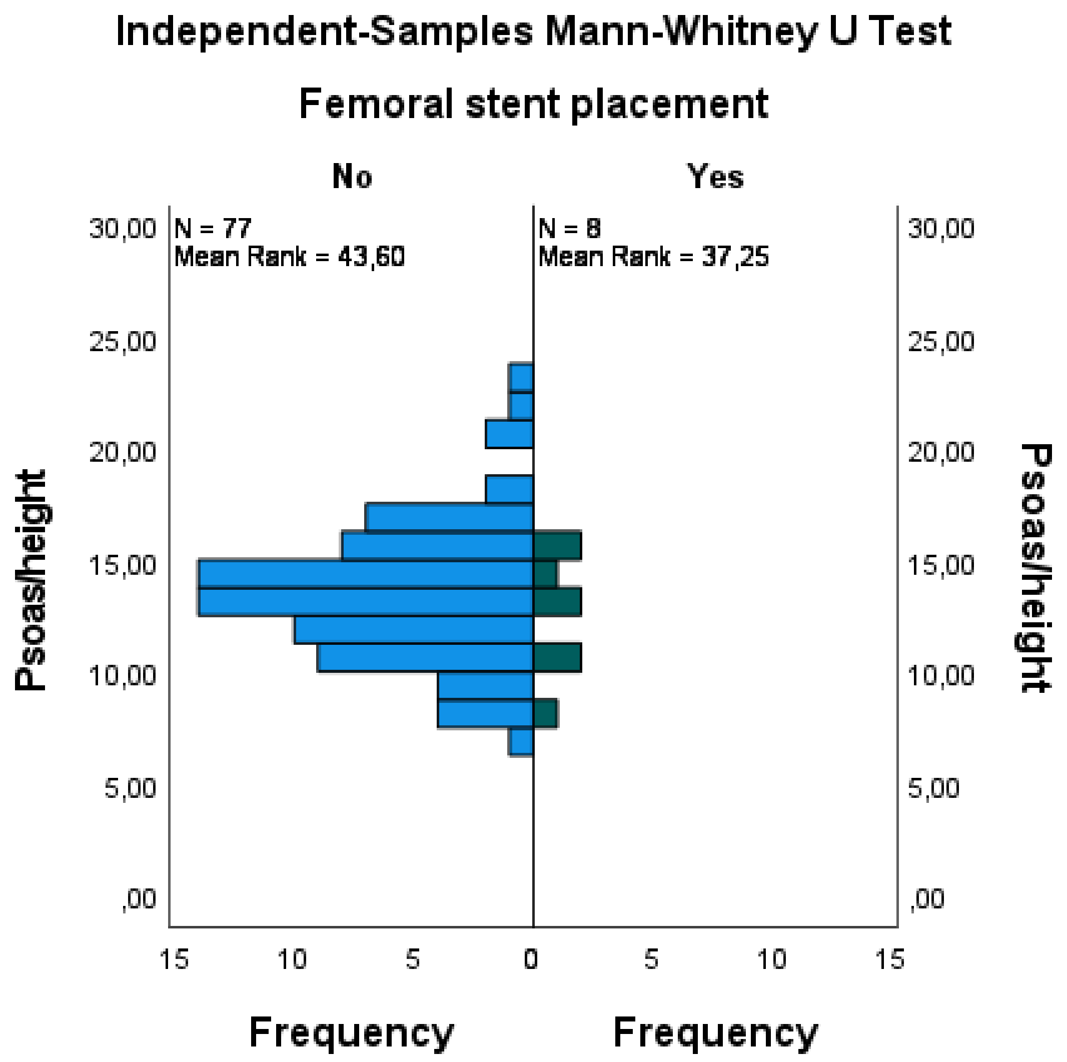

Anterior SAT distance across Femoral stent placement

Independent-Samples Mann-Whitney U Test Summary

Total N	85
Mann-Whitney U	268,000
Wilcoxon W	304,000
Test Statistic	268,000
Standard Error	66,434
Standardized Test Statistic	-,602
Asymptotic Sig.(2-sided test)	,547

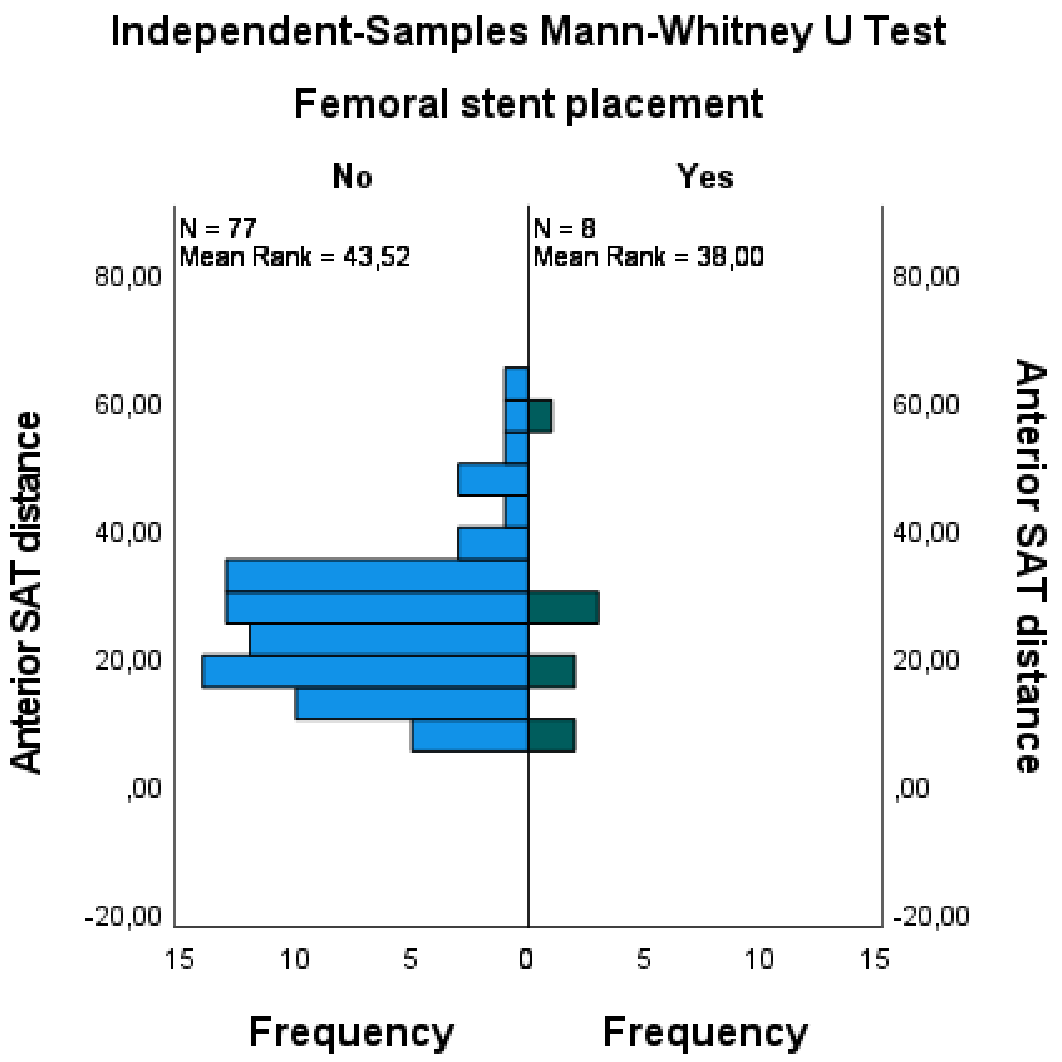

Posterior SAT distance across Femoral stent placement

Independent-Samples Mann-Whitney U Test Summary

Total N	85
Mann-Whitney U	231,000
Wilcoxon W	267,000
Test Statistic	231,000
Standard Error	66,435
Standardized Test Statistic	-1,159
Asymptotic Sig.(2-sided test)	,246

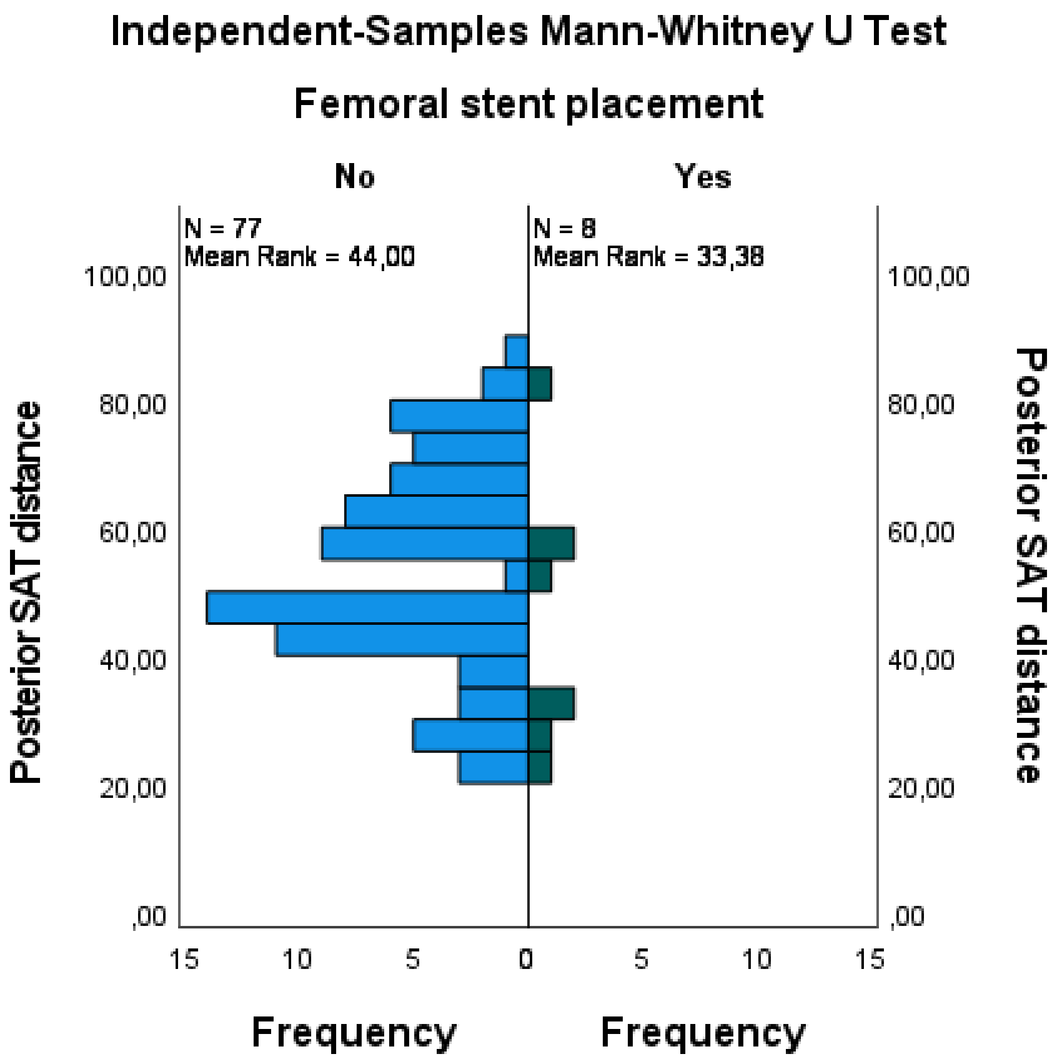

Anterior+Posterior SAT distance across Femoral stent placement

Independent-Samples Mann-Whitney U Test Summary

Total N	85
Mann-Whitney U	253,500
Wilcoxon W	289,500
Test Statistic	253,500
Standard Error	66,440
Standardized Test Statistic	-,820
Asymptotic Sig.(2-sided test)	,412

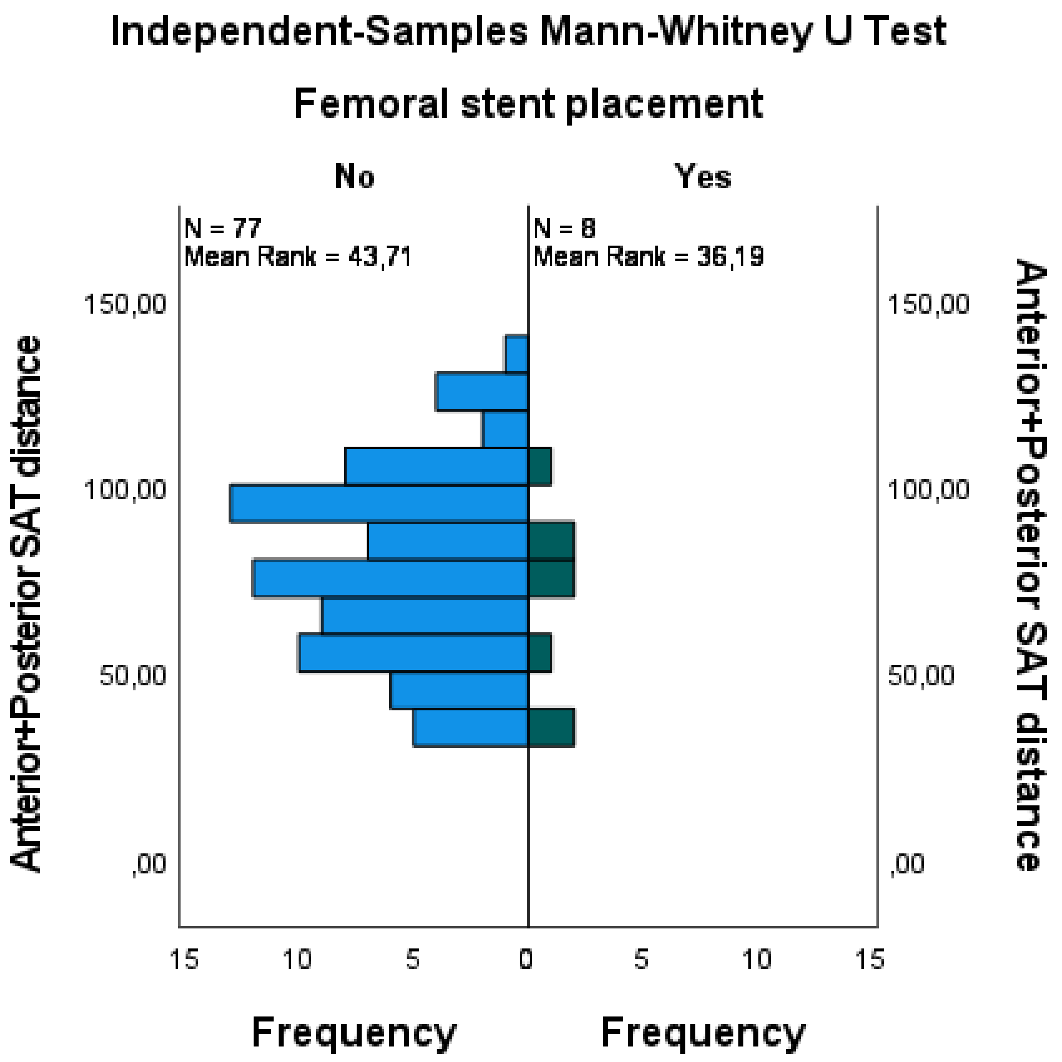

VAT distance across Femoral stent placement

Independent-Samples Mann-Whitney U Test Summary

Total N	84
Mann-Whitney U	349,000
Wilcoxon W	385,000
Test Statistic	349,000
Standard Error	65,612
Standardized Test Statistic	,686
Asymptotic Sig.(2-sided test)	,493

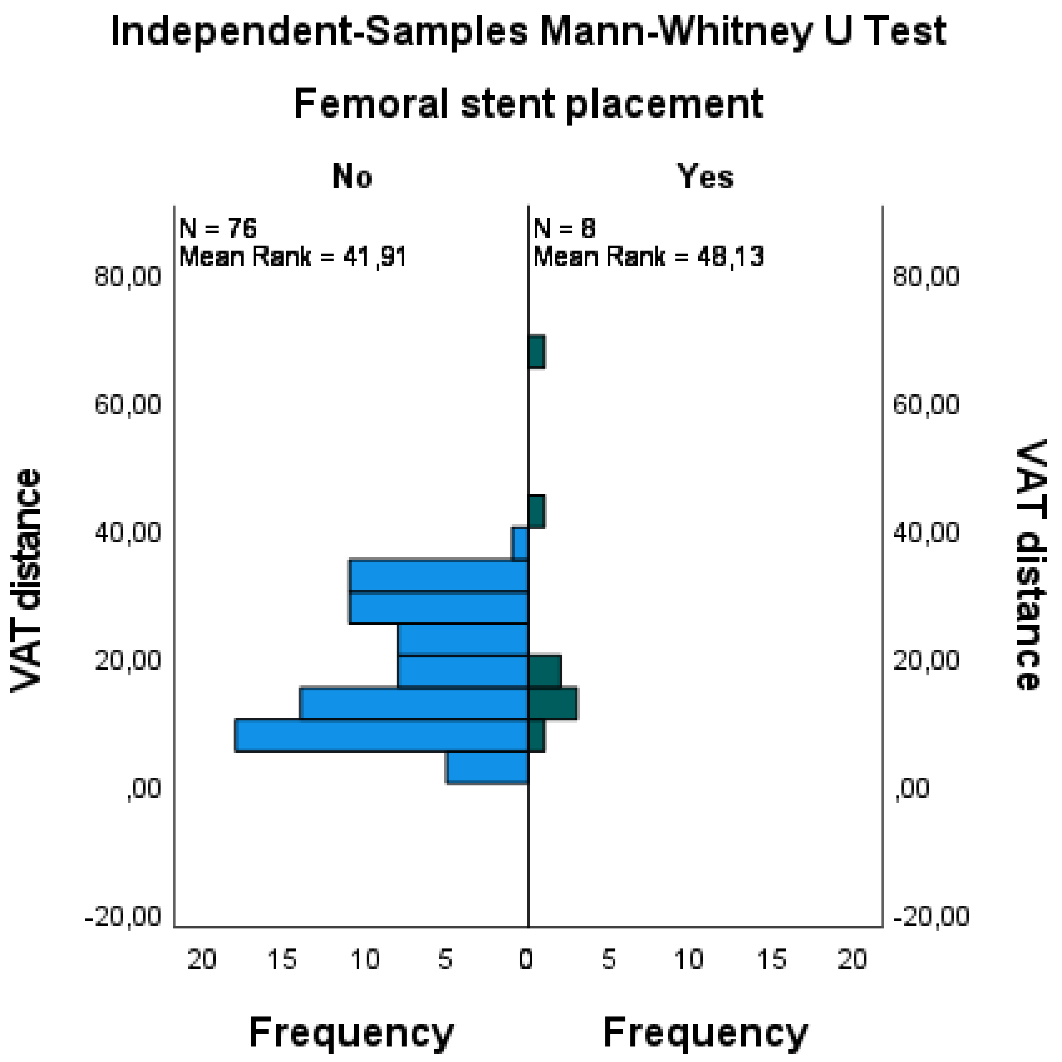

Right common femoral artery area (mm2) across Femoral stent placement

Independent-Samples Mann-Whitney U Test Summary

Total N	85
Mann-Whitney U	214,000
Wilcoxon W	250,000
Test Statistic	214,000
Standard Error	66,432
Standardized Test Statistic	-1,415
Asymptotic Sig.(2-sided test)	,157

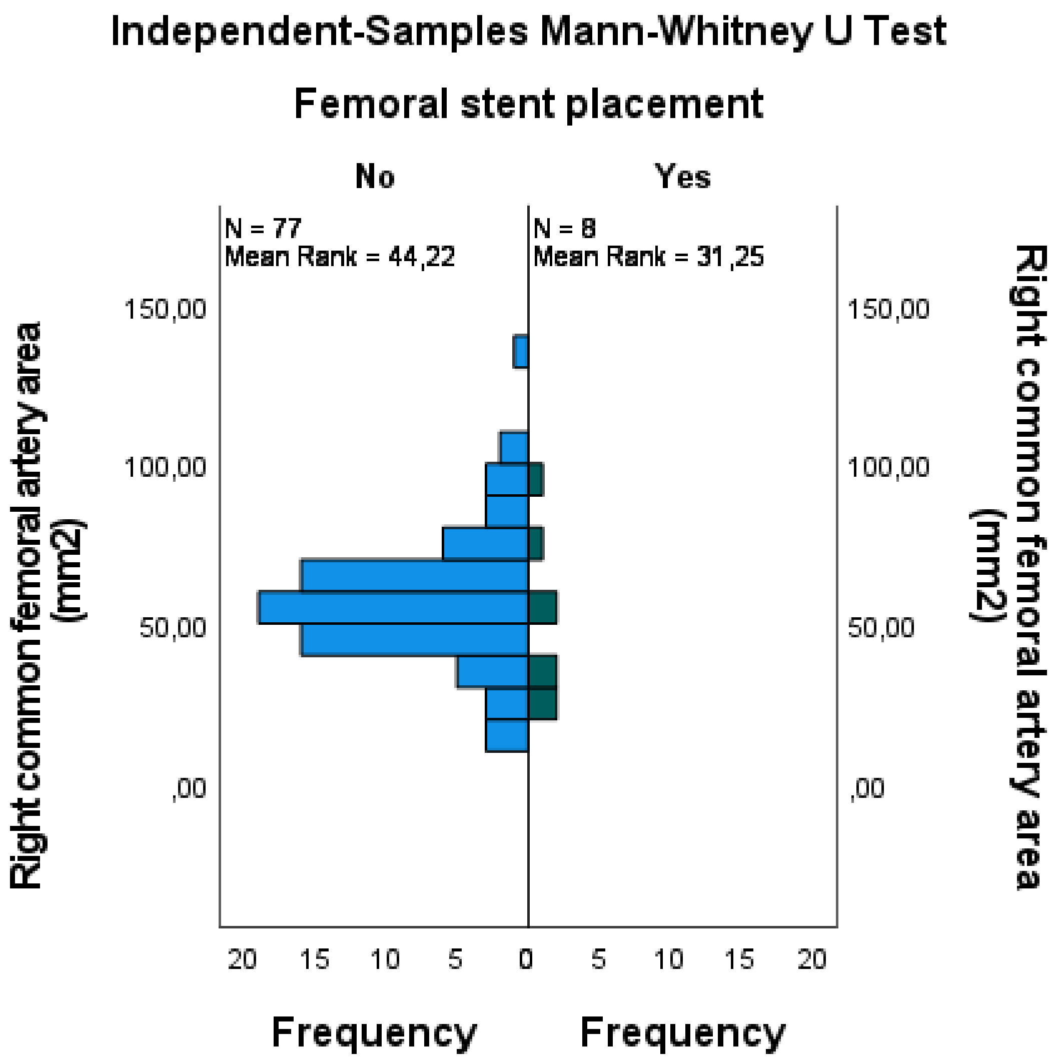

Left common femoral artery area (mm2) across Femoral stent placement

Independent-Samples Mann-Whitney U Test Summary

Total N	85
Mann-Whitney U	226,500
Wilcoxon W	262,500
Test Statistic	226,500
Standard Error	66,427
Standardized Test Statistic	-1,227
Asymptotic Sig.(2-sided test)	,220

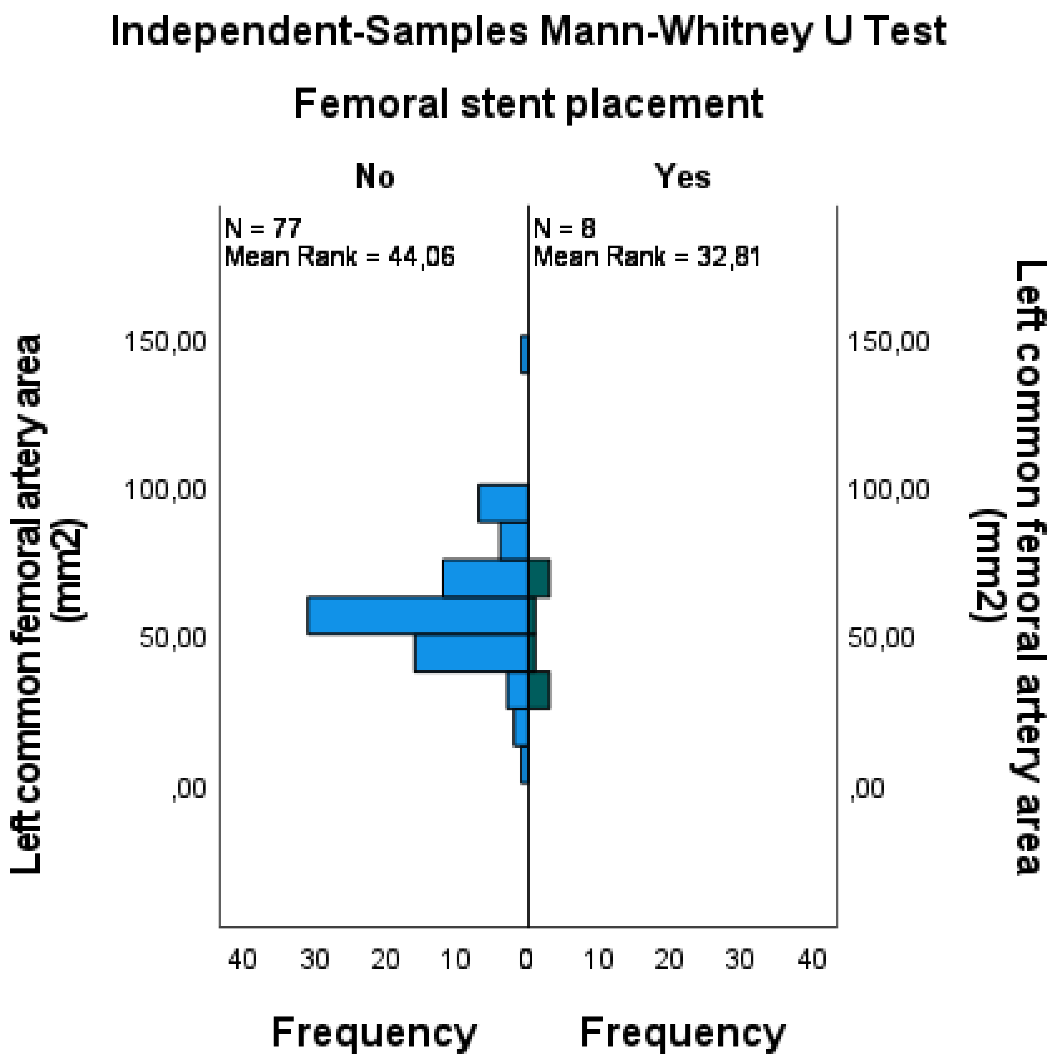

FAT area (cm2) across Femoral stent placement

Independent-Samples Mann-Whitney U Test Summary

Total N	85
Mann-Whitney U	292,000
Wilcoxon W	328,000
Test Statistic	292,000
Standard Error	66,443
Standardized Test Statistic	-,241
Asymptotic Sig.(2-sided test)	,810

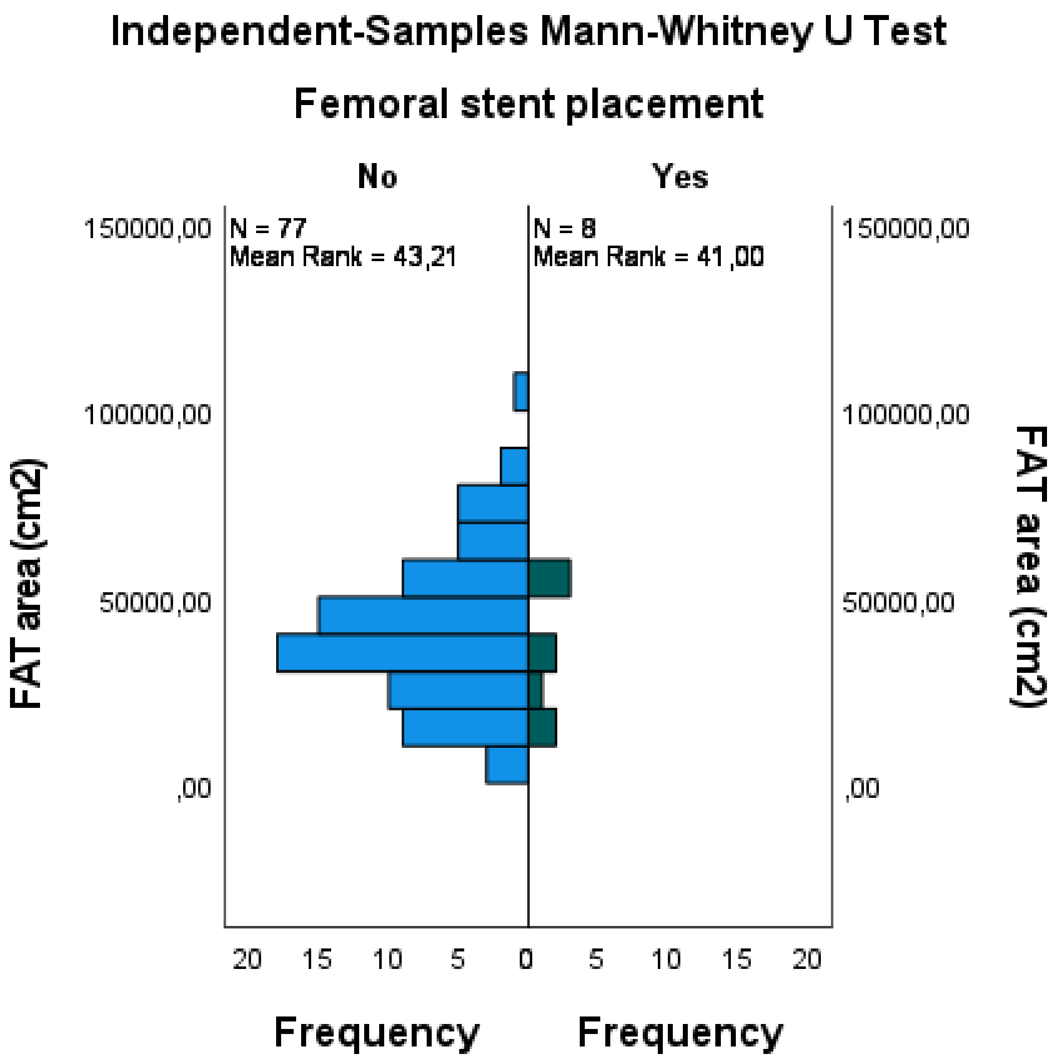

SAT area (cm2) across Femoral stent placement

Independent-Samples Mann-Whitney U Test Summary

Total N	85
Mann-Whitney U	271,500
Wilcoxon W	307,500
Test Statistic	271,500
Standard Error	66,443
Standardized Test Statistic	-,549
Asymptotic Sig.(2-sided test)	,583

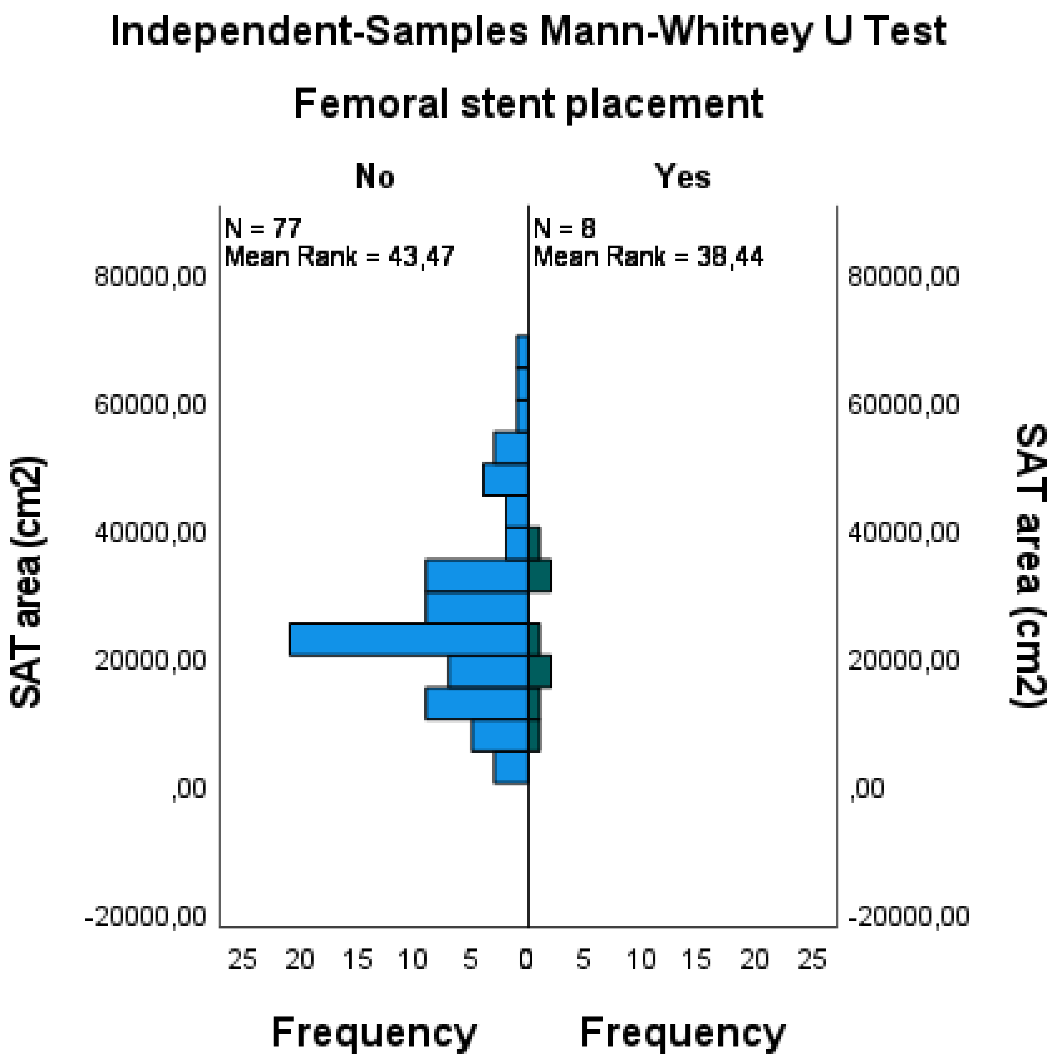

VAT area (cm2) across Femoral stent placement

Independent-Samples Mann-Whitney U Test Summary

Total N	84
Mann-Whitney U	270,000
Wilcoxon W	306,000
Test Statistic	270,000
Standard Error	65,625
Standardized Test Statistic	-,518
Asymptotic Sig.(2-sided test)	,604

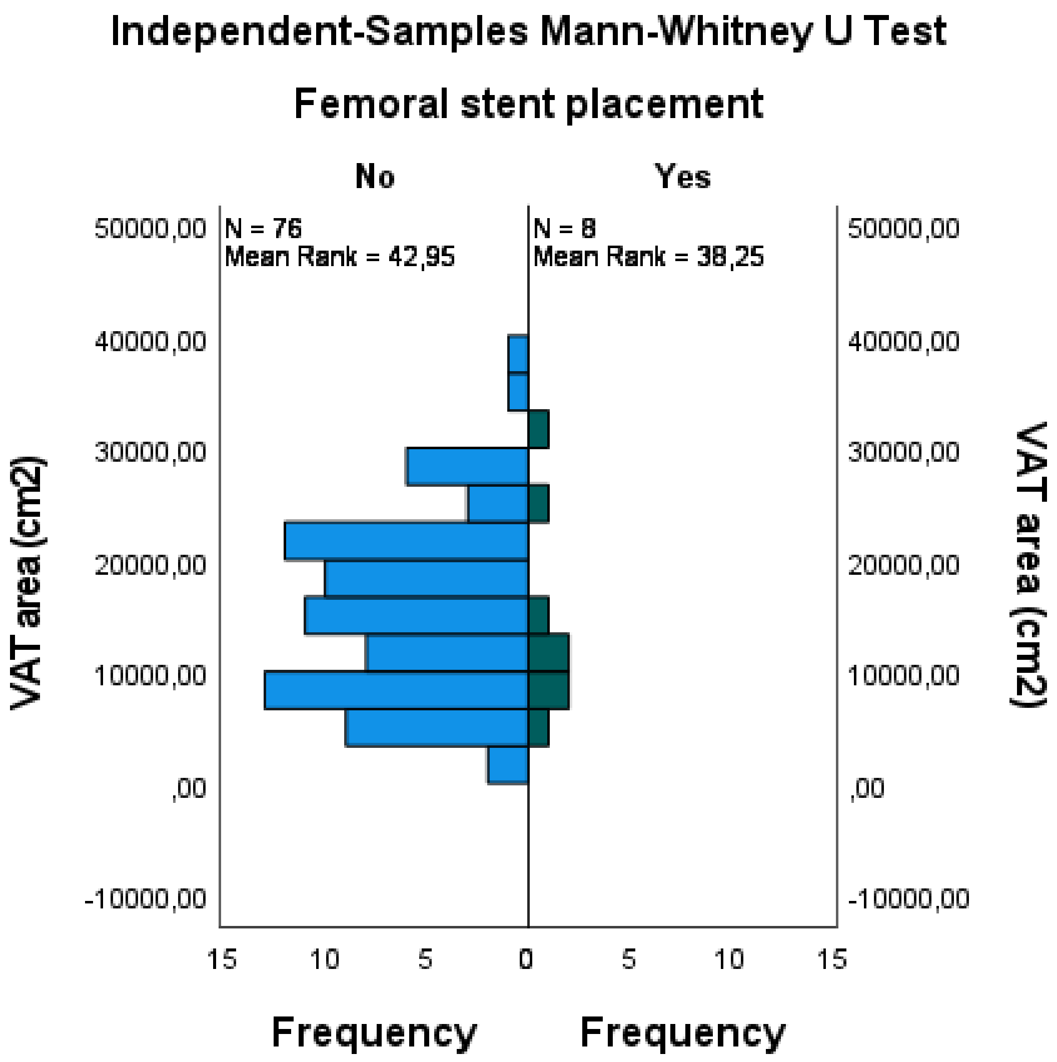

Right Psoas muscle area (cm2) across Femoral stent placement

Independent-Samples Mann-Whitney U Test Summary

Total N	85
Mann-Whitney U	180,000
Wilcoxon W	216,000
Test Statistic	180,000
Standard Error	66,441
Standardized Test Statistic	-1,927
Asymptotic Sig.(2-sided test)	,054

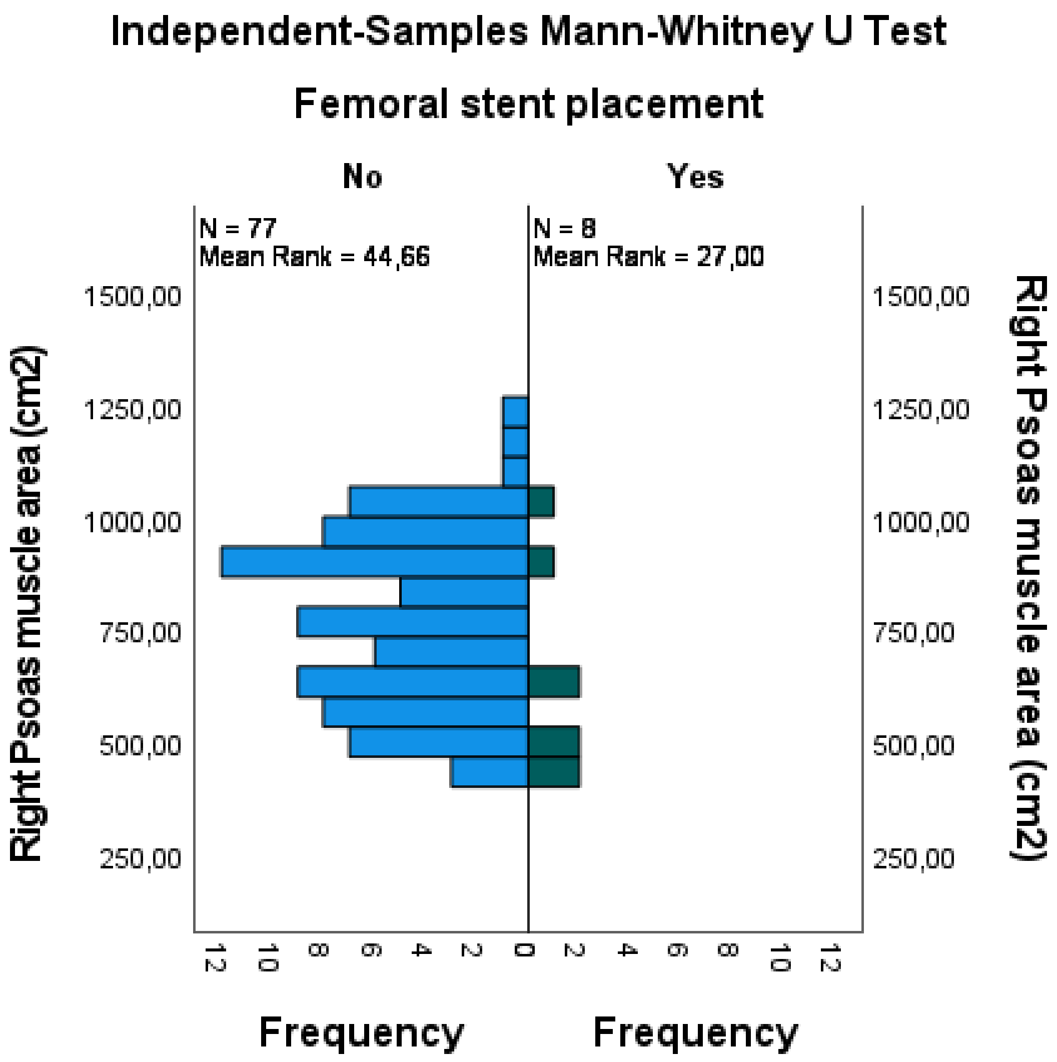

Left Psoas muscle area (cm2) across Femoral stent placement

Independent-Samples Mann-Whitney U Test Summary

Total N	85
Mann-Whitney U	198,000
Wilcoxon W	234,000
Test Statistic	198,000
Standard Error	66,442
Standardized Test Statistic	-1,656
Asymptotic Sig.(2-sided test)	,098

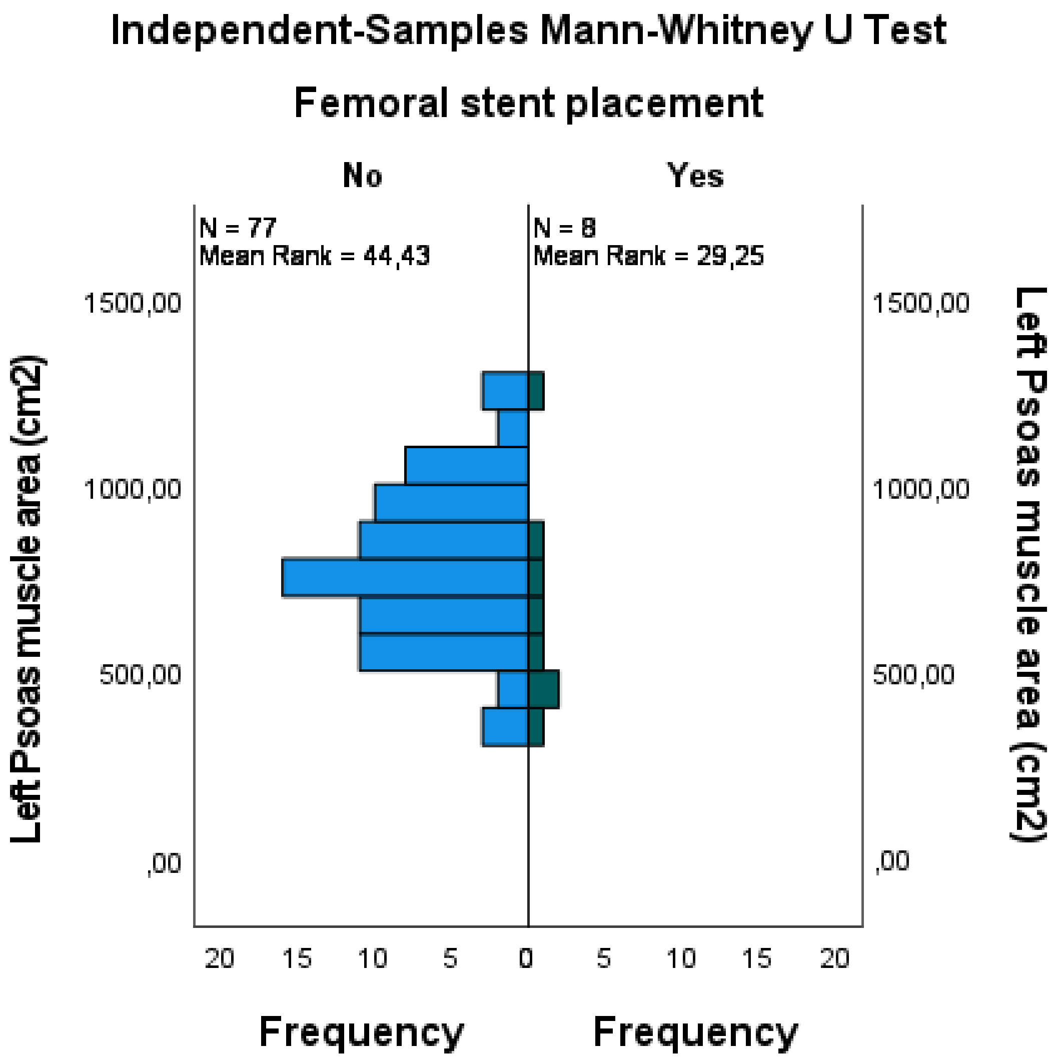

FAT mean density (HU) across Femoral stent placement

Independent-Samples Mann-Whitney U Test Summary

Total N	85
Mann-Whitney U	306,000
Wilcoxon W	342,000
Test Statistic	306,000
Standard Error	66,432
Standardized Test Statistic	-,030
Asymptotic Sig.(2-sided test)	,976

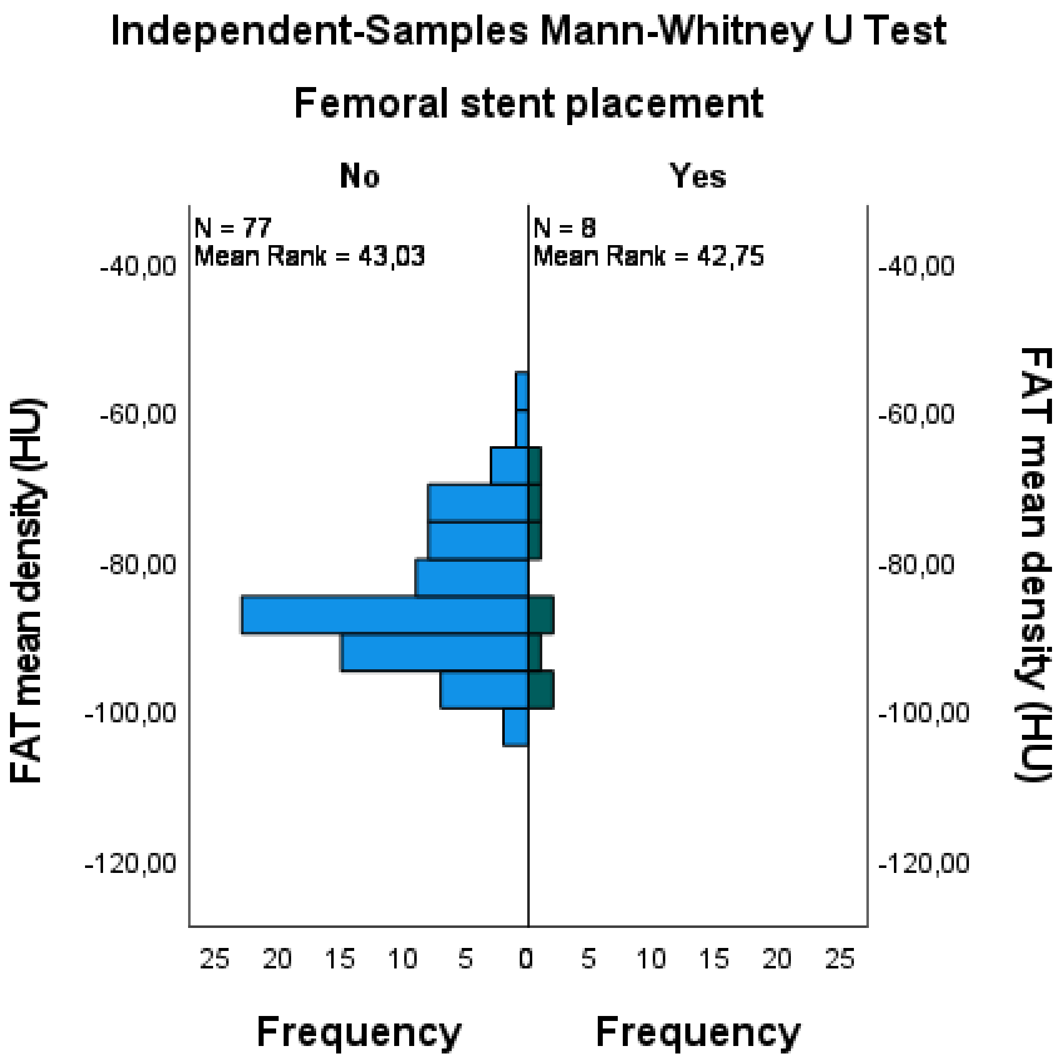

FAT median density (HU) across Femoral stent placement

Independent-Samples Mann-Whitney U Test Summary

Total N	85
Mann-Whitney U	312,500
Wilcoxon W	348,500
Test Statistic	312,500
Standard Error	66,363
Standardized Test Statistic	,068
Asymptotic Sig.(2-sided test)	,946

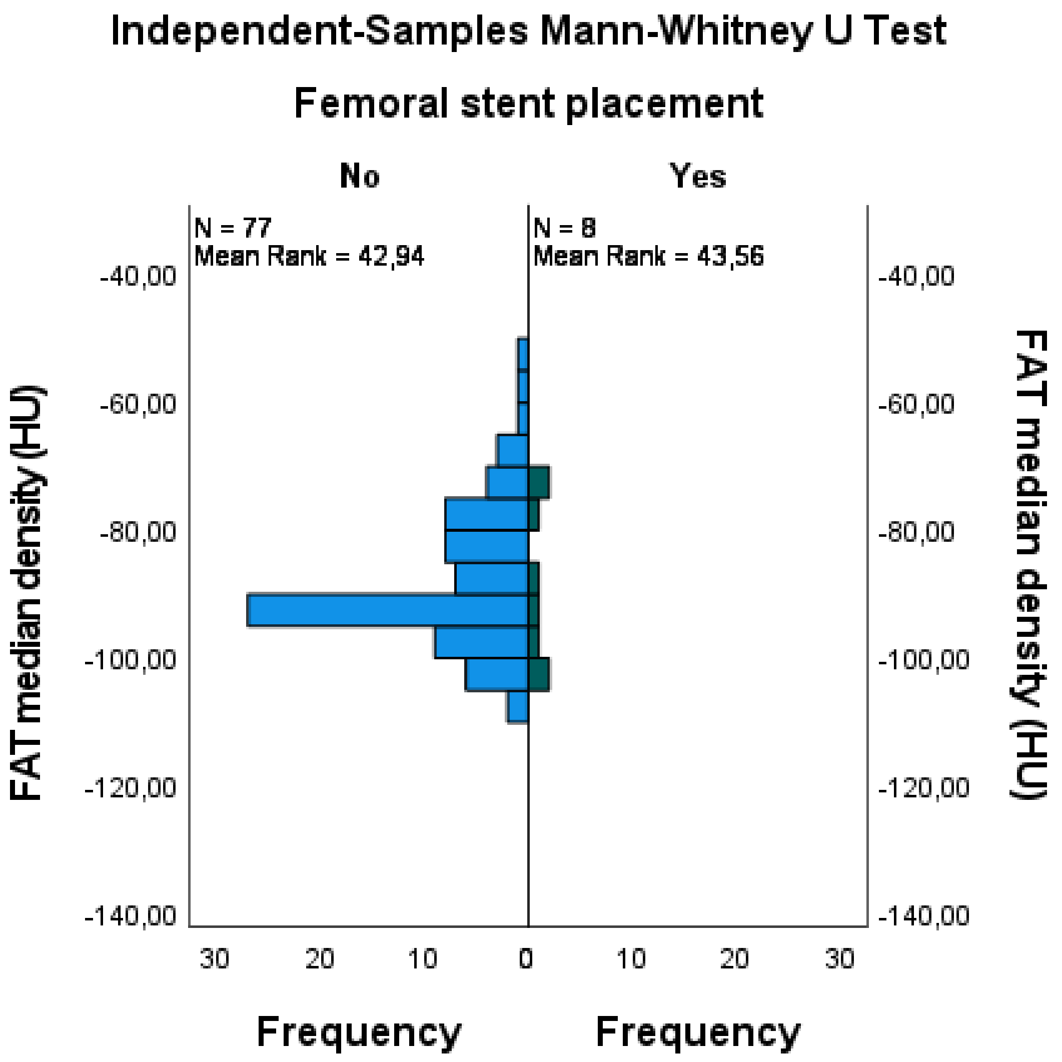

FAT density standard deviation across Femoral stent placement

Independent-Samples Mann-Whitney U Test Summary

Total N	85
Mann-Whitney U	323,000
Wilcoxon W	359,000
Test Statistic	323,000
Standard Error	66,443
Standardized Test Statistic	,226
Asymptotic Sig.(2-sided test)	,821

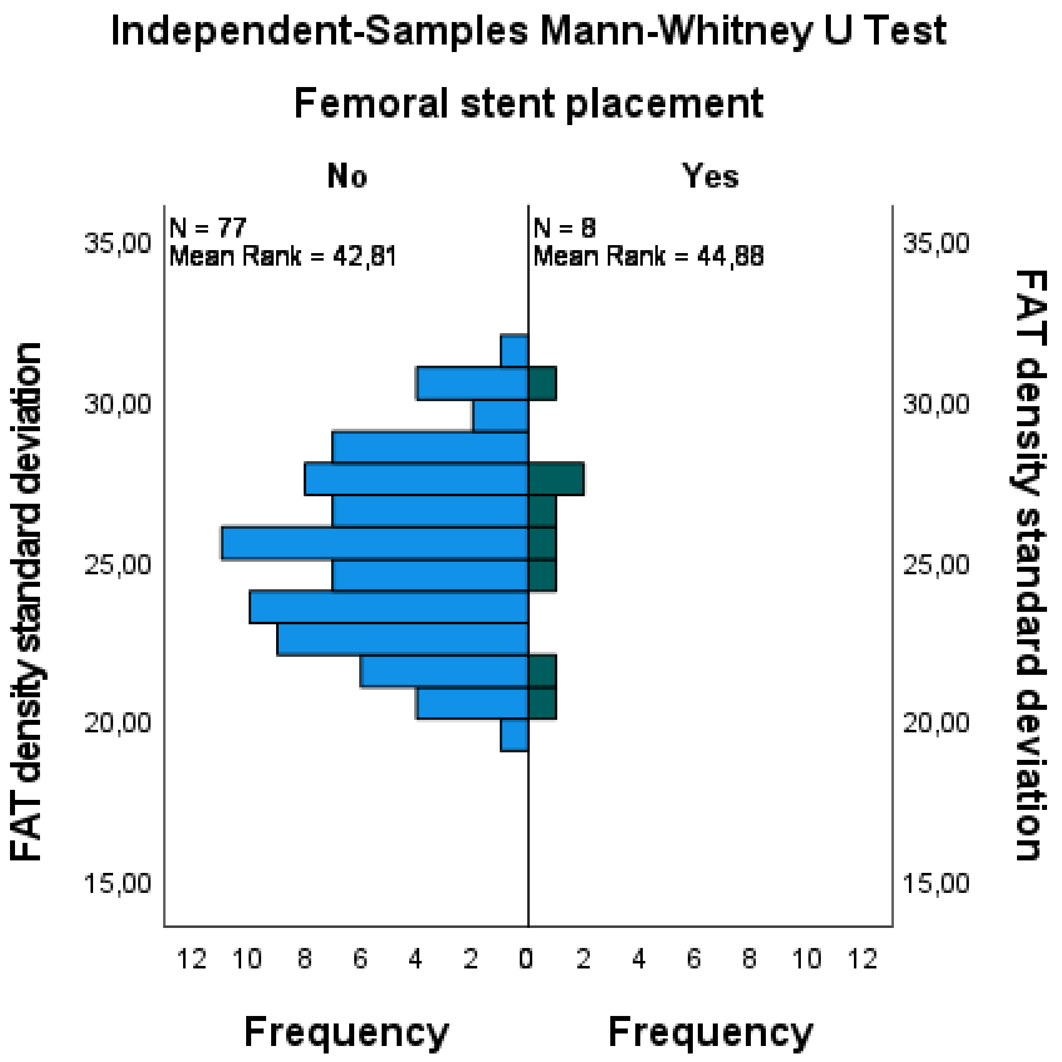

SAT mean density (HU) across Femoral stent placement

Independent-Samples Mann-Whitney U Test Summary

Total N	85
Mann-Whitney U	295,500
Wilcoxon W	331,500
Test Statistic	295,500
Standard Error	66,435
Standardized Test Statistic	-,188
Asymptotic Sig.(2-sided test)	,851

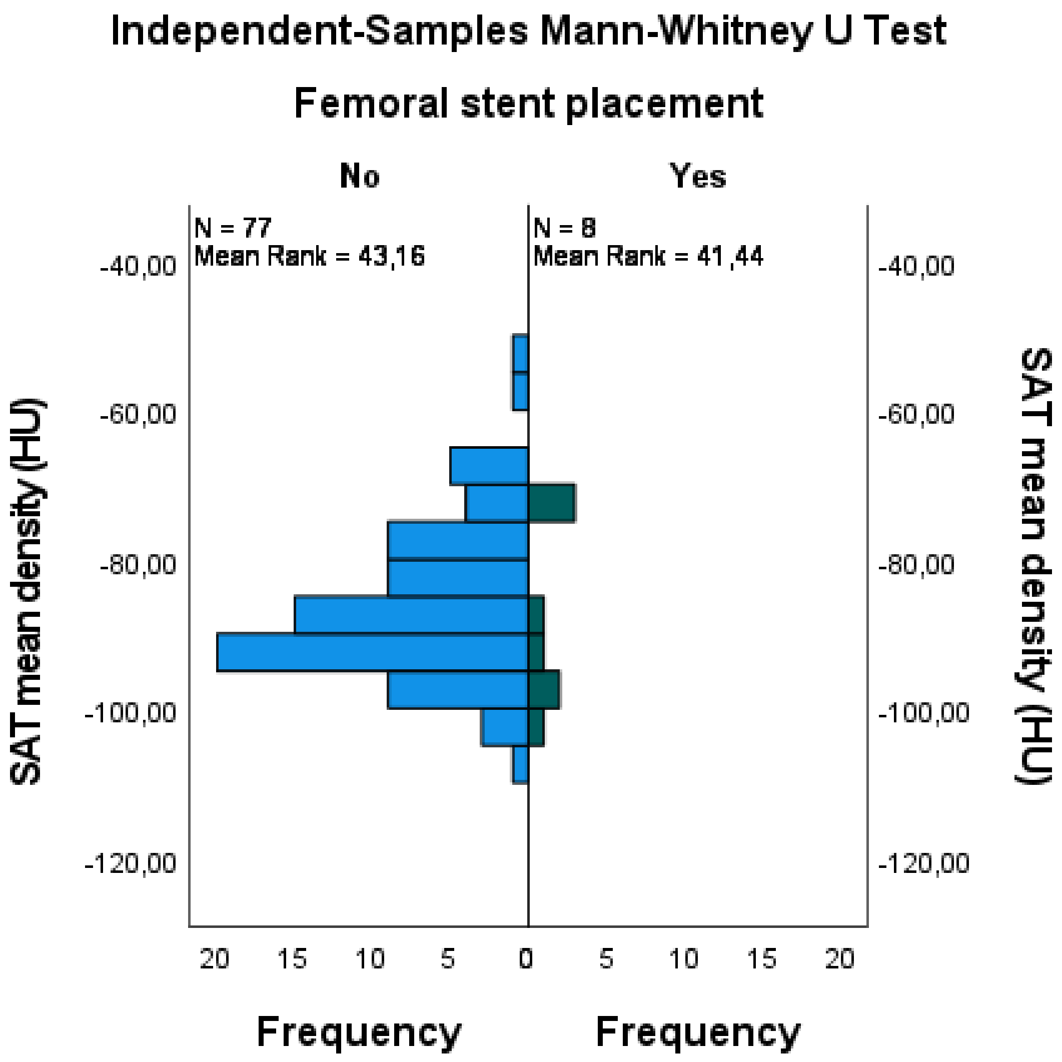

SAT median density (HU) across Femoral stent placement

Independent-Samples Mann-Whitney U Test Summary

Total N	83
Mann-Whitney U	292,000
Wilcoxon W	328,000
Test Statistic	292,000
Standard Error	64,757
Standardized Test Statistic	-,124
Asymptotic Sig.(2-sided test)	,902

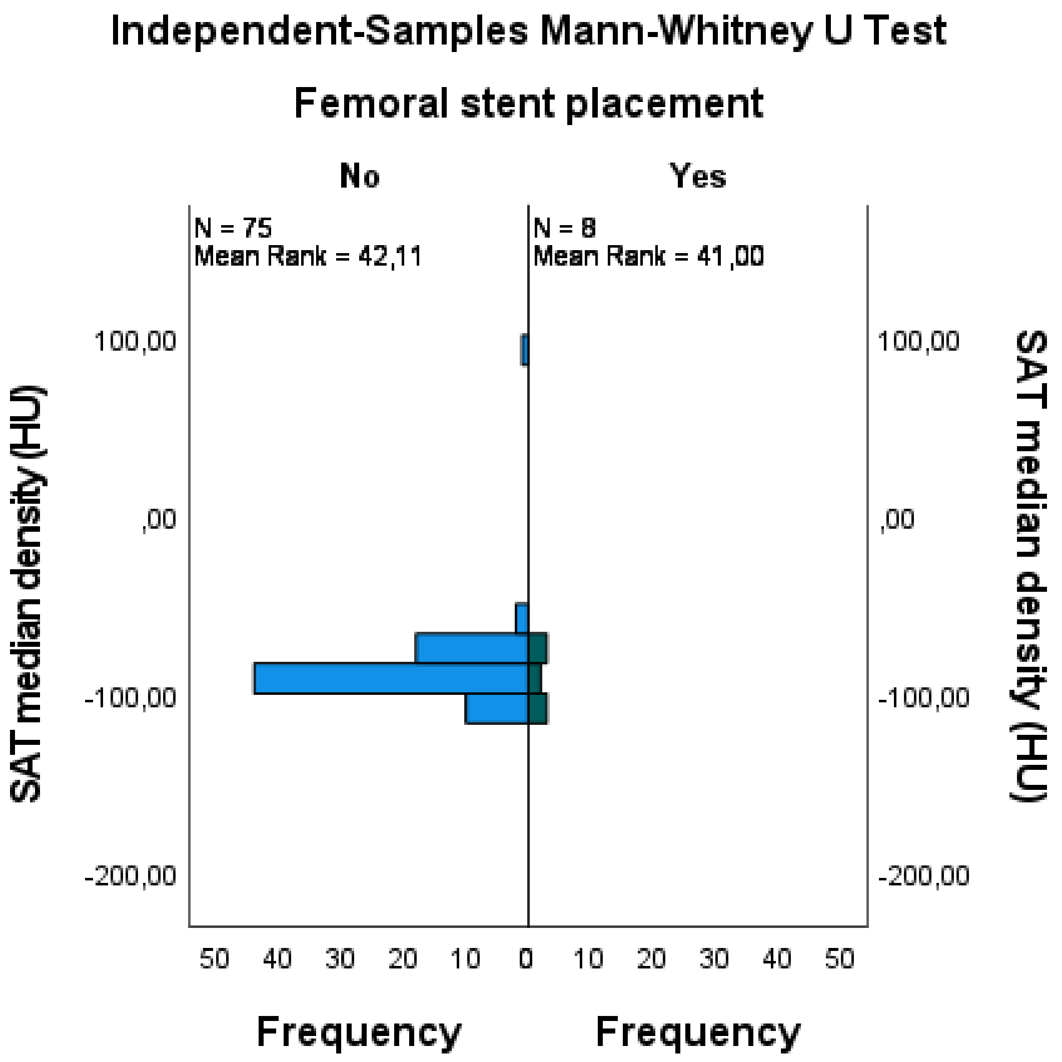

SAT density standard deviation across Femoral stent placement

Independent-Samples Mann-Whitney U Test Summary

Total N	84
Mann-Whitney U	334,000
Wilcoxon W	370,000
Test Statistic	334,000
Standard Error	65,625
Standardized Test Statistic	,457
Asymptotic Sig.(2-sided test)	,648

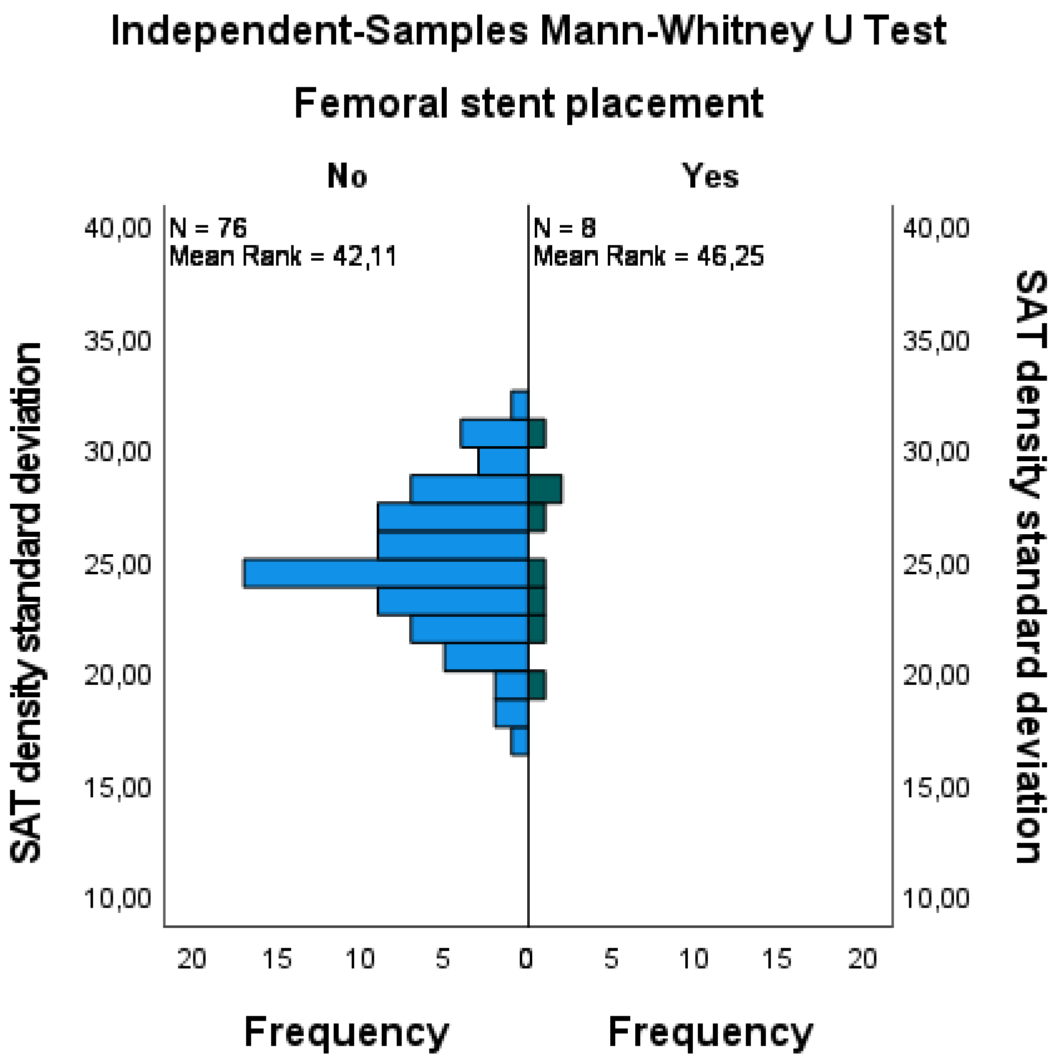

VAT mean density (HU) across Femoral stent placement

Independent-Samples Mann-Whitney U Test Summary

Total N	85
Mann-Whitney U	310,000
Wilcoxon W	346,000
Test Statistic	310,000
Standard Error	66,431
Standardized Test Statistic	,030
Asymptotic Sig.(2-sided test)	,976

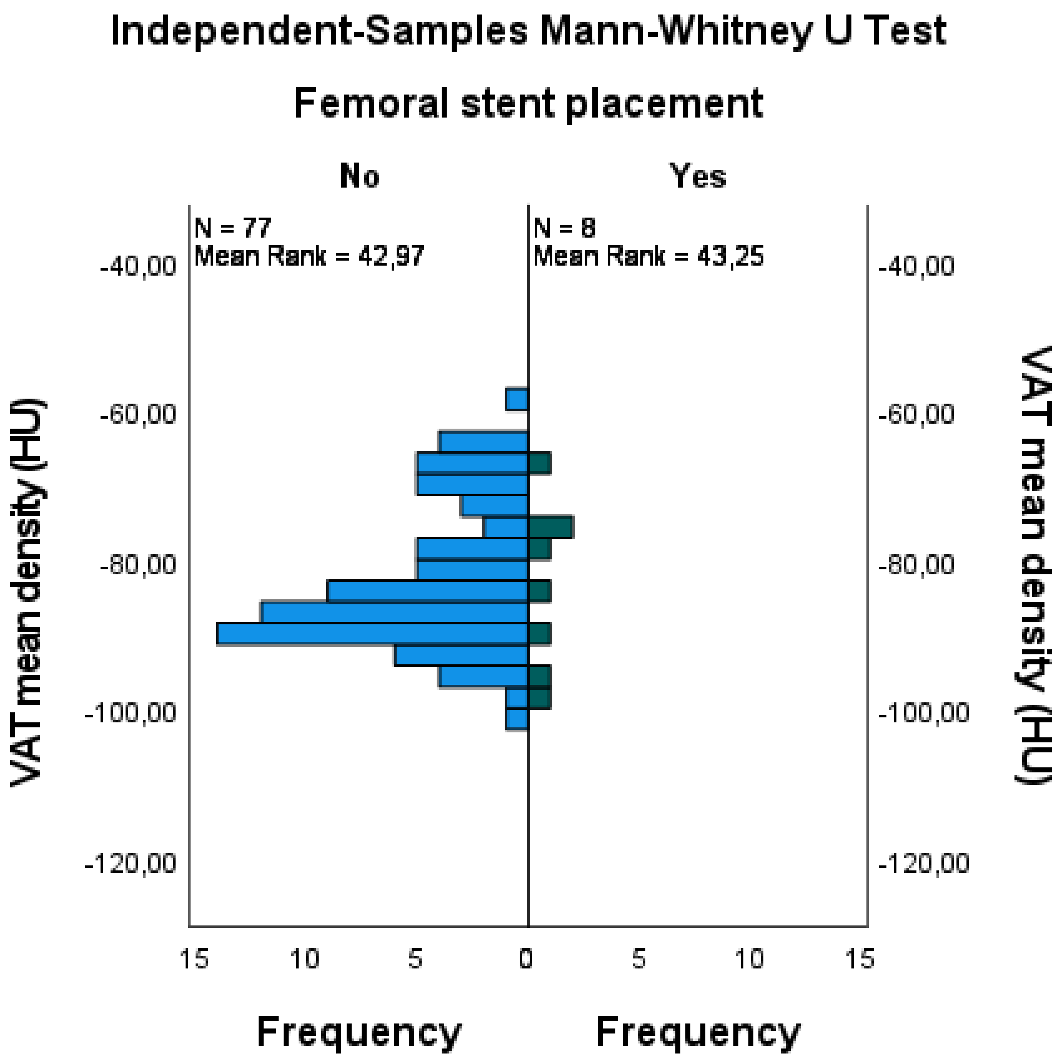

VAT median density (HU) across Femoral stent placement

Independent-Samples Mann-Whitney U Test Summary

Total N	85
Mann-Whitney U	319,500
Wilcoxon W	355,500
Test Statistic	319,500
Standard Error	66,360
Standardized Test Statistic	,173
Asymptotic Sig.(2-sided test)	,862

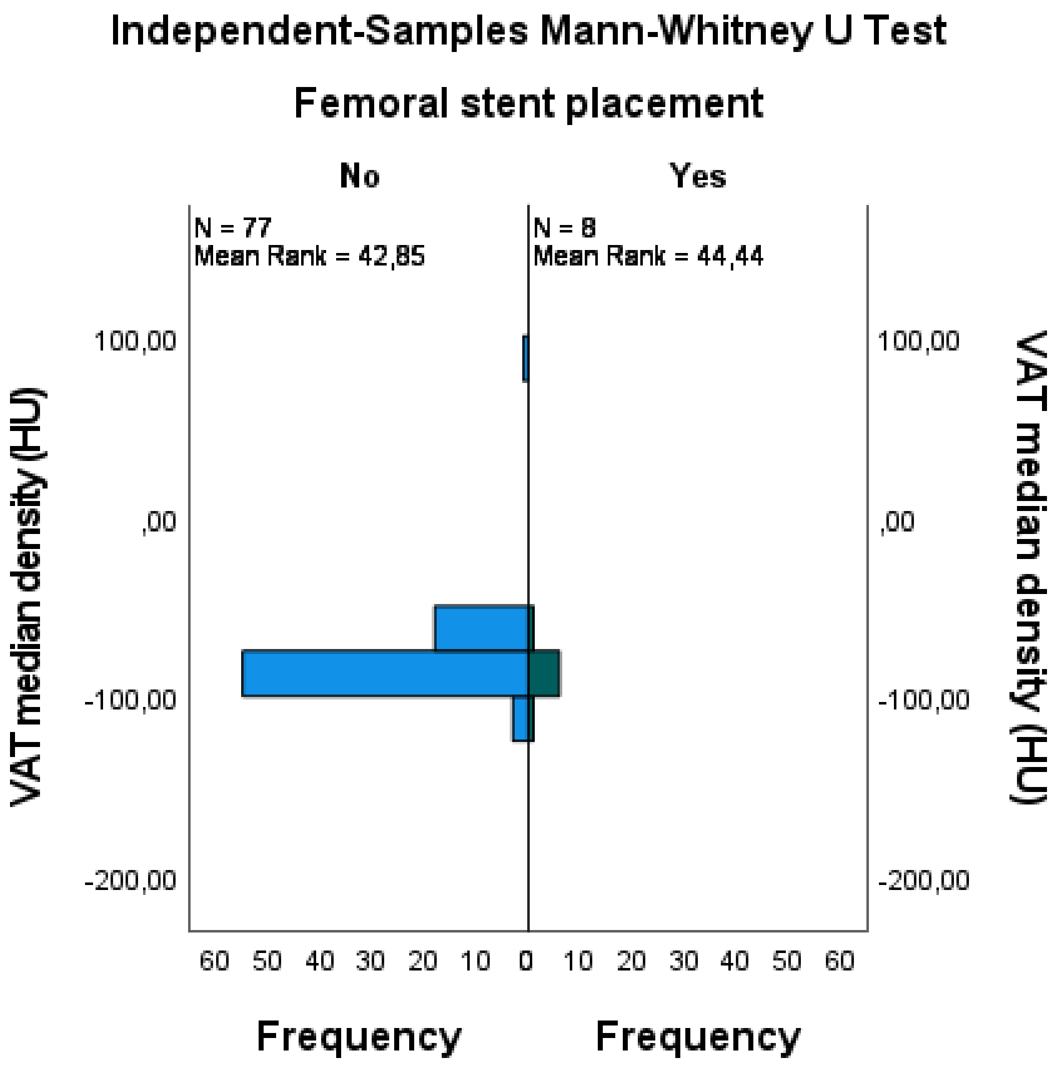

VAT density standard deviation across Femoral stent placement

Independent-Samples Mann-Whitney U Test Summary

Total N	84
Mann-Whitney U	436,000
Wilcoxon W	472,000
Test Statistic	436,000
Standard Error	65,625
Standardized Test Statistic	2,011
Asymptotic Sig.(2-sided test)	,044

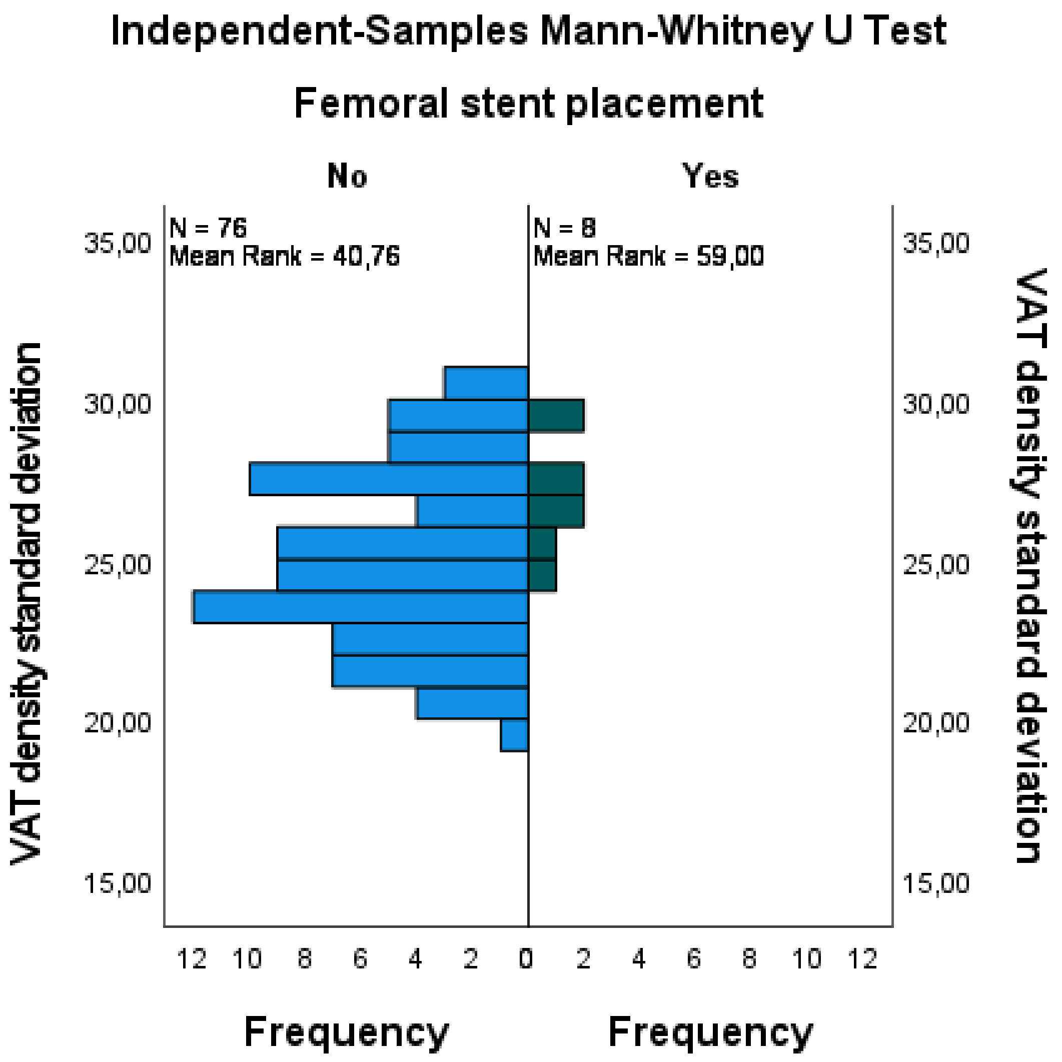

Right Psoas muscle mean density (HU) across Femoral stent placement

Independent-Samples Mann-Whitney U Test Summary

Total N	85
Mann-Whitney U	165,500
Wilcoxon W	201,500
Test Statistic	165,500
Standard Error	66,428
Standardized Test Statistic	-2,145
Asymptotic Sig.(2-sided test)	,032

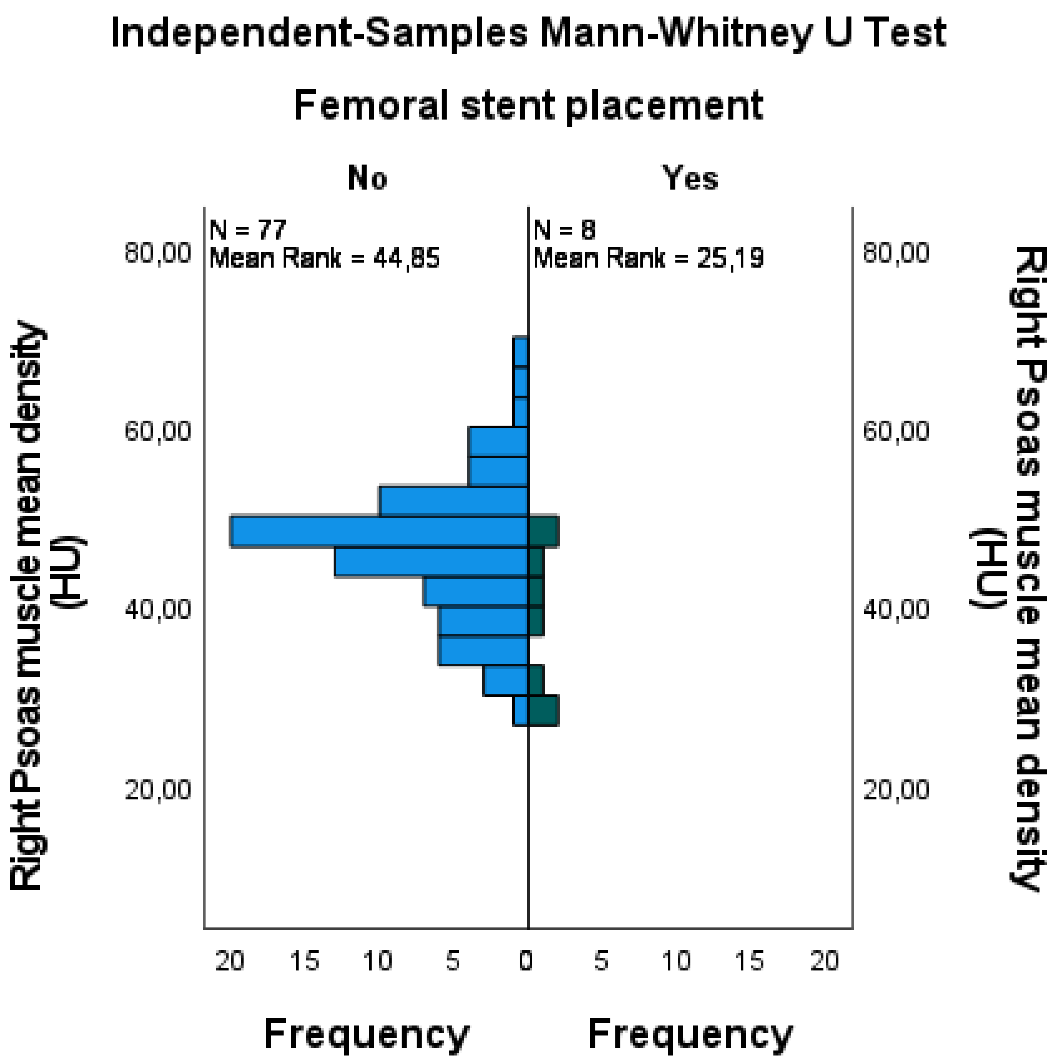

Right Psoas muscle median density (HU) across Femoral stent placement

Independent-Samples Mann-Whitney U Test Summary

Total N	85
Mann-Whitney U	197,500
Wilcoxon W	233,500
Test Statistic	197,500
Standard Error	66,380
Standardized Test Statistic	-1,665
Asymptotic Sig.(2-sided test)	,096

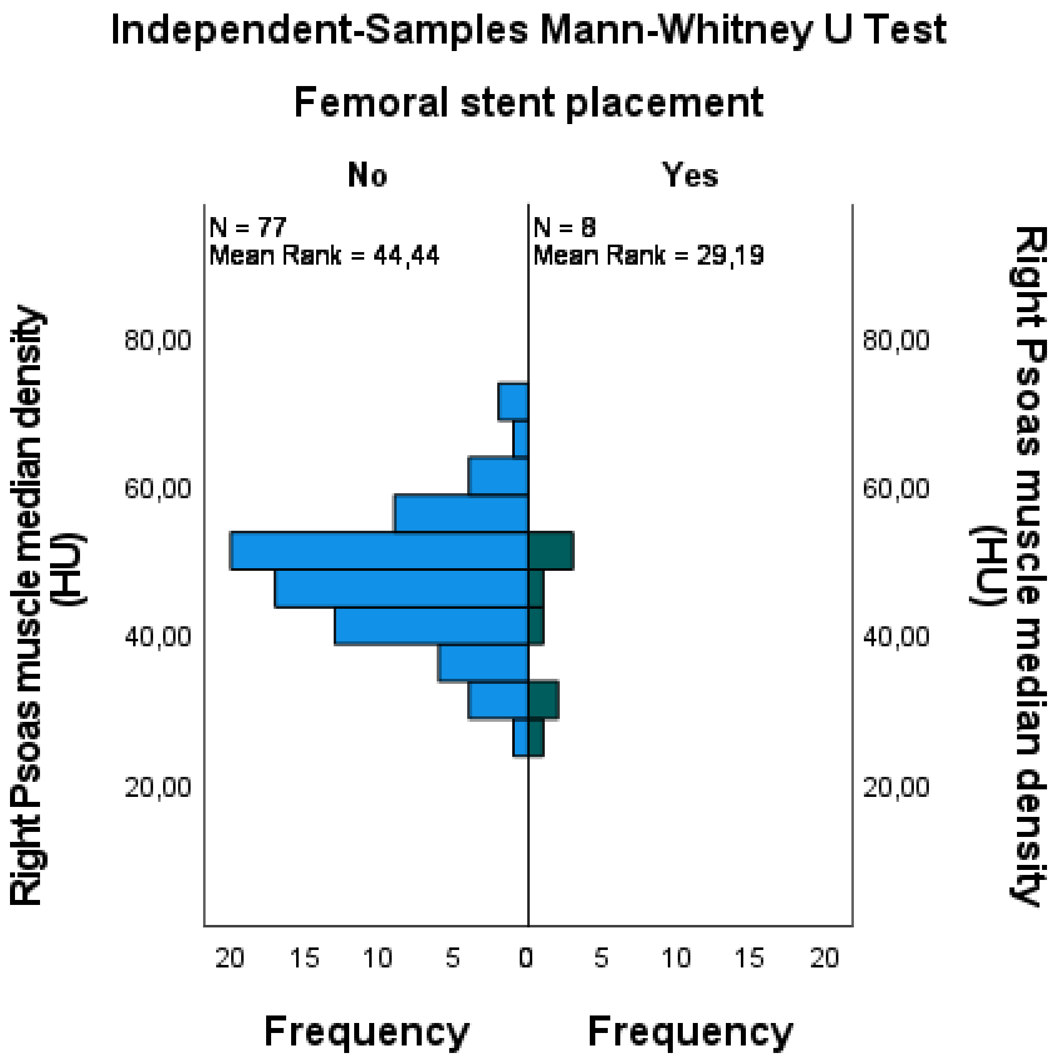

Right Psoas muscle density standard deviation across Femoral stent placement

Independent-Samples Mann-Whitney U Test Summary

Total N	85
Mann-Whitney U	284,000
Wilcoxon W	320,000
Test Statistic	284,000
Standard Error	66,442
Standardized Test Statistic	-,361
Asymptotic Sig.(2-sided test)	,718

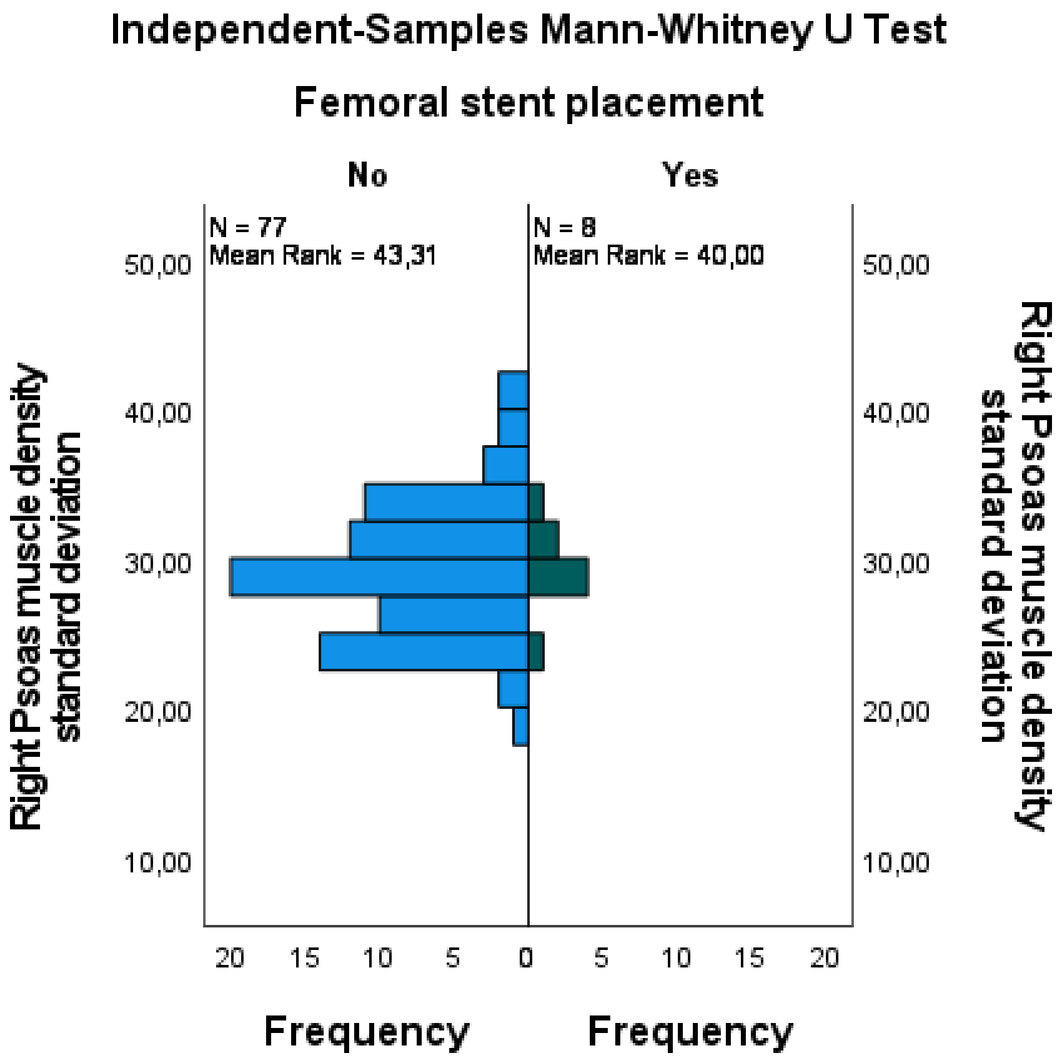

Left Psoas muscle mean density (HU) across Femoral stent placement

Independent-Samples Mann-Whitney U Test Summary

Total N	85
Mann-Whitney U	189,000
Wilcoxon W	225,000
Test Statistic	189,000
Standard Error	66,429
Standardized Test Statistic	-1,791
Asymptotic Sig.(2-sided test)	,073

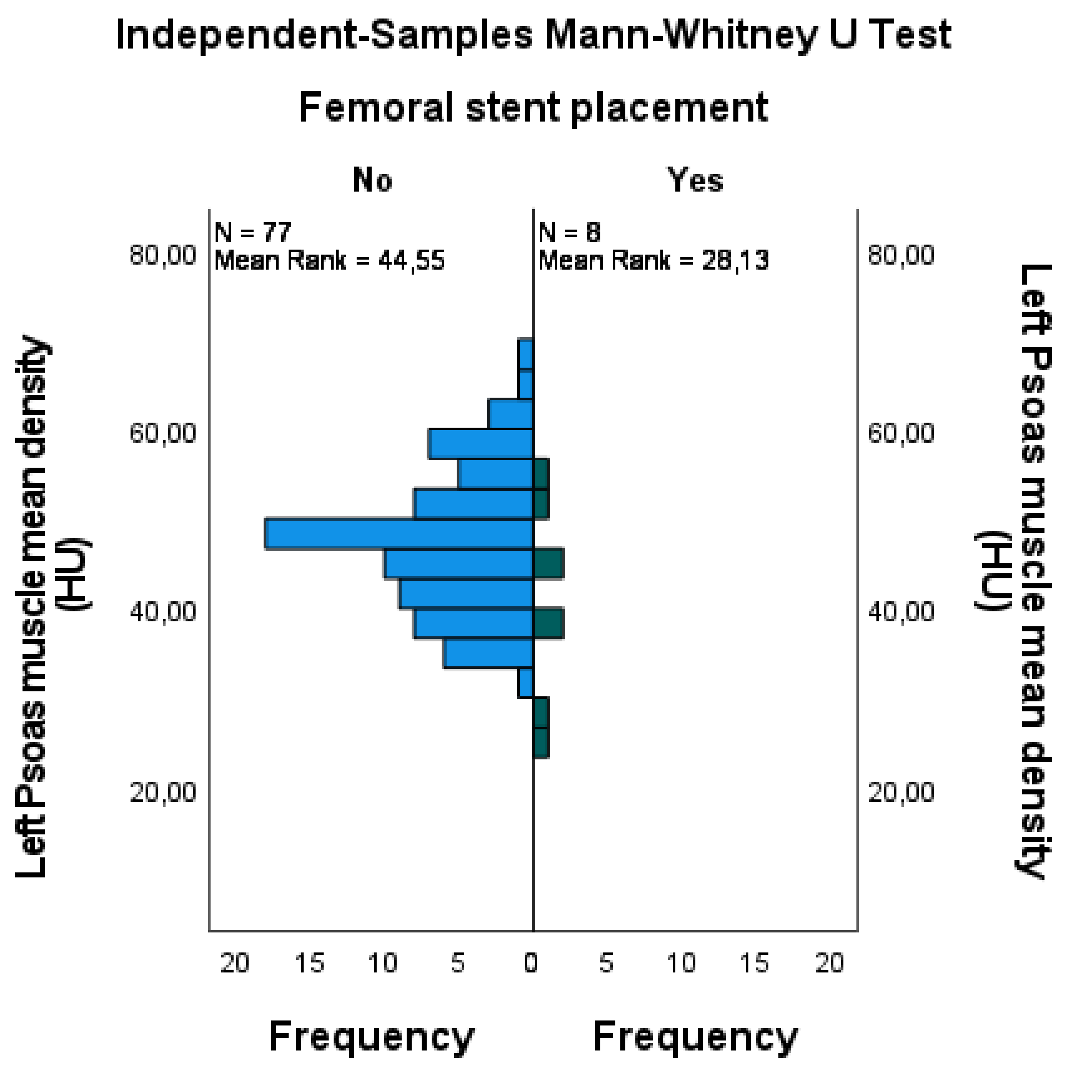

Left Psoas muscle median density (HU) across Femoral stent placement

Independent-Samples Mann-Whitney U Test Summary

Total N	85
Mann-Whitney U	207,000
Wilcoxon W	243,000
Test Statistic	207,000
Standard Error	66,358
Standardized Test Statistic	-1,522
Asymptotic Sig.(2-sided test)	,128

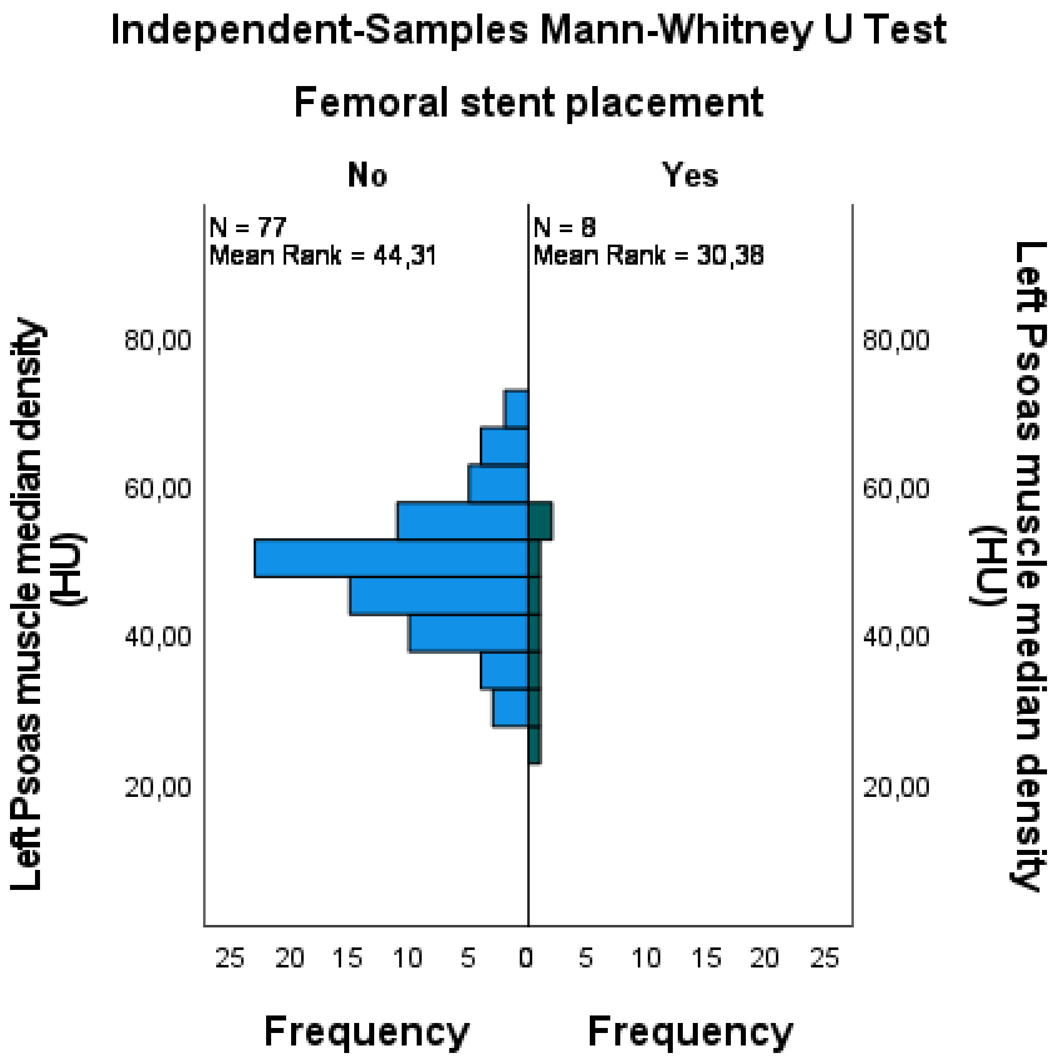

Left Psoas muscle density standard deviation across Femoral stent placement

Independent-Samples Mann-Whitney U Test Summary

Total N	85
Mann-Whitney U	271,000
Wilcoxon W	307,000
Test Statistic	271,000
Standard Error	66,443
Standardized Test Statistic	-,557
Asymptotic Sig.(2-sided test)	,578

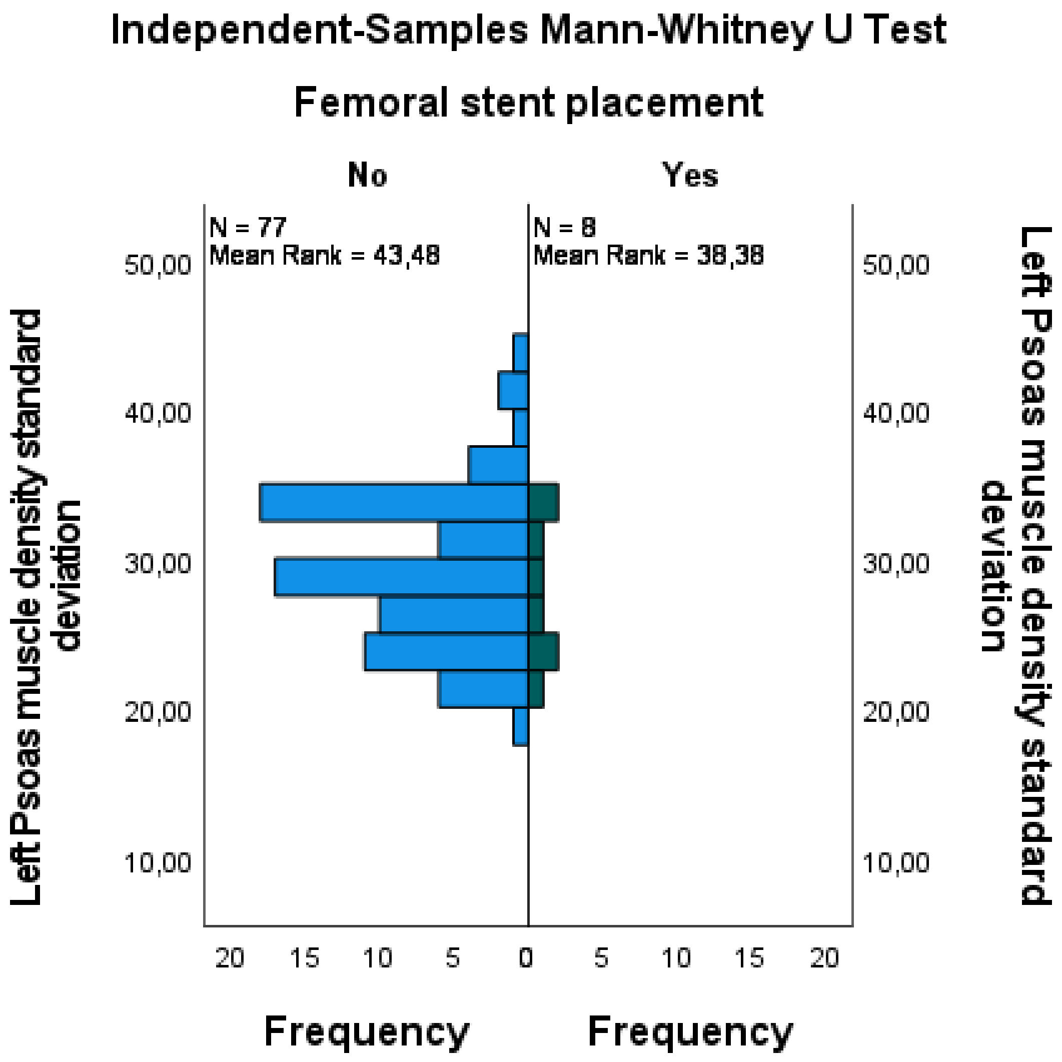

Independent-Samples Mann-Whitney U Test for femoral bleeding

Hypothesis Test Summary

	Null Hypothesis	Test	Sig.[a,b]	Decision
1	The distribution of Psoas/height is the same across categories of Femoral bleeding.	Independent-Samples Mann-Whitney U Test	,381[c]	Retain the null hypothesis.
2	The distribution of Anterior SAT distance is the same across categories of Femoral bleeding.	Independent-Samples Mann-Whitney U Test	,404[c]	Retain the null hypothesis.
3	The distribution of Posterior SAT distance is the same across categories of Femoral bleeding.	Independent-Samples Mann-Whitney U Test	,652[c]	Retain the null hypothesis.
4	The distribution of Anterior+Posterior SAT distance is the same across categories of Femoral bleeding.	Independent-Samples Mann-Whitney U Test	,582[c]	Retain the null hypothesis.
5	The distribution of VAT distance is the same across categories of Femoral bleeding.	Independent-Samples Mann-Whitney U Test	,678[c]	Retain the null hypothesis.
6	The distribution of Right common femoral artery area (mm2) is the same across categories of Femoral bleeding.	Independent-Samples Mann-Whitney U Test	,849[c]	Retain the null hypothesis.
7	The distribution of Left common femoral artery area (mm2) is the same across categories of Femoral bleeding.	Independent-Samples Mann-Whitney U Test	,476[c]	Retain the null hypothesis.
8	The distribution of FAT area (cm2) is the same across categories of Femoral bleeding.	Independent-Samples Mann-Whitney U Test	,451[c]	Retain the null hypothesis.
9	The distribution of SAT area (cm2) is the same across categories of Femoral bleeding.	Independent-Samples Mann-Whitney U Test	,711[c]	Retain the null hypothesis.
10	The distribution of VAT area (cm2) is the same across categories of Femoral bleeding.	Independent-Samples Mann-Whitney U Test	,364[c]	Retain the null hypothesis.

11	The distribution of Right Psoas muscle area (cm2) is the same across categories of Femoral bleeding.	Independent-Samples Mann-Whitney U Test	,392[c]	Retain the null hypothesis.
12	The distribution of Left Psoas muscle area (cm2) is the same across categories of Femoral bleeding.	Independent-Samples Mann-Whitney U Test	,211[c]	Retain the null hypothesis.
13	The distribution of FAT mean density (HU) is the same across categories of Femoral bleeding.	Independent-Samples Mann-Whitney U Test	,439[c]	Retain the null hypothesis.
14	The distribution of FAT median density (HU) is the same across categories of Femoral bleeding.	Independent-Samples Mann-Whitney U Test	,348[c]	Retain the null hypothesis.
15	The distribution of FAT density standard deviation is the same across categories of Femoral bleeding.	Independent-Samples Mann-Whitney U Test	,652[c]	Retain the null hypothesis.
16	The distribution of SAT mean density (HU) is the same across categories of Femoral bleeding.	Independent-Samples Mann-Whitney U Test	,610[c]	Retain the null hypothesis.
17	The distribution of SAT median density (HU) is the same across categories of Femoral bleeding.	Independent-Samples Mann-Whitney U Test	,531[c]	Retain the null hypothesis.
18	The distribution of SAT density standard deviation is the same across categories of Femoral bleeding.	Independent-Samples Mann-Whitney U Test	,738[c]	Retain the null hypothesis.
19	The distribution of VAT mean density (HU) is the same across categories of Femoral bleeding.	Independent-Samples Mann-Whitney U Test	,392[c]	Retain the null hypothesis.
20	The distribution of VAT median density (HU) is the same across categories of Femoral bleeding.	Independent-Samples Mann-Whitney U Test	,288[c]	Retain the null hypothesis.
21	The distribution of VAT density standard deviation is the same across categories of Femoral bleeding.	Independent-Samples Mann-Whitney U Test	,708[c]	Retain the null hypothesis.
22	The distribution of Right Psoas muscle mean density (HU) is the same across categories of Femoral bleeding.	Independent-Samples Mann-Whitney U Test	,174[c]	Retain the null hypothesis.

23	The distribution of Right Psoas muscle median density (HU) is the same across categories of Femoral bleeding.	Independent-Samples Mann-Whitney U Test	,489[c]	Retain the null hypothesis.
24	The distribution of Right Psoas muscle density standard deviation is the same across categories of Femoral bleeding.	Independent-Samples Mann-Whitney U Test	,976[c]	Retain the null hypothesis.
25	The distribution of Left Psoas muscle mean density (HU) is the same across categories of Femoral bleeding.	Independent-Samples Mann-Whitney U Test	,881[c]	Retain the null hypothesis.
26	The distribution of Left Psoas muscle median density (HU) is the same across categories of Femoral bleeding.	Independent-Samples Mann-Whitney U Test	,976[c]	Retain the null hypothesis.
27	The distribution of Left Psoas muscle density standard deviation is the same across categories of Femoral bleeding.	Independent-Samples Mann-Whitney U Test	,624[c]	Retain the null hypothesis.

a. The significance level is ,050.

b. Asymptotic significance is displayed.

c. Exact significance is displayed for this test.

In this case, the hypothesis of equal medians is accepted for all the variables ($p > 0.05$). (The tables and graphs below are the details of the tests in this table: I have highlighted what things you should eventually report, namely test statistic and pvalue).

Psoas/height across Femoral bleeding

Independent-Samples Mann-Whitney U Test Summary

Total N	85
Mann-Whitney U	118,000
Wilcoxon W	128,000
Test Statistic	118,000
Standard Error	48,187
Standardized Test Statistic	-,913
Asymptotic Sig.(2-sided test)	,361
Exact Sig.(2-sided test)	,381

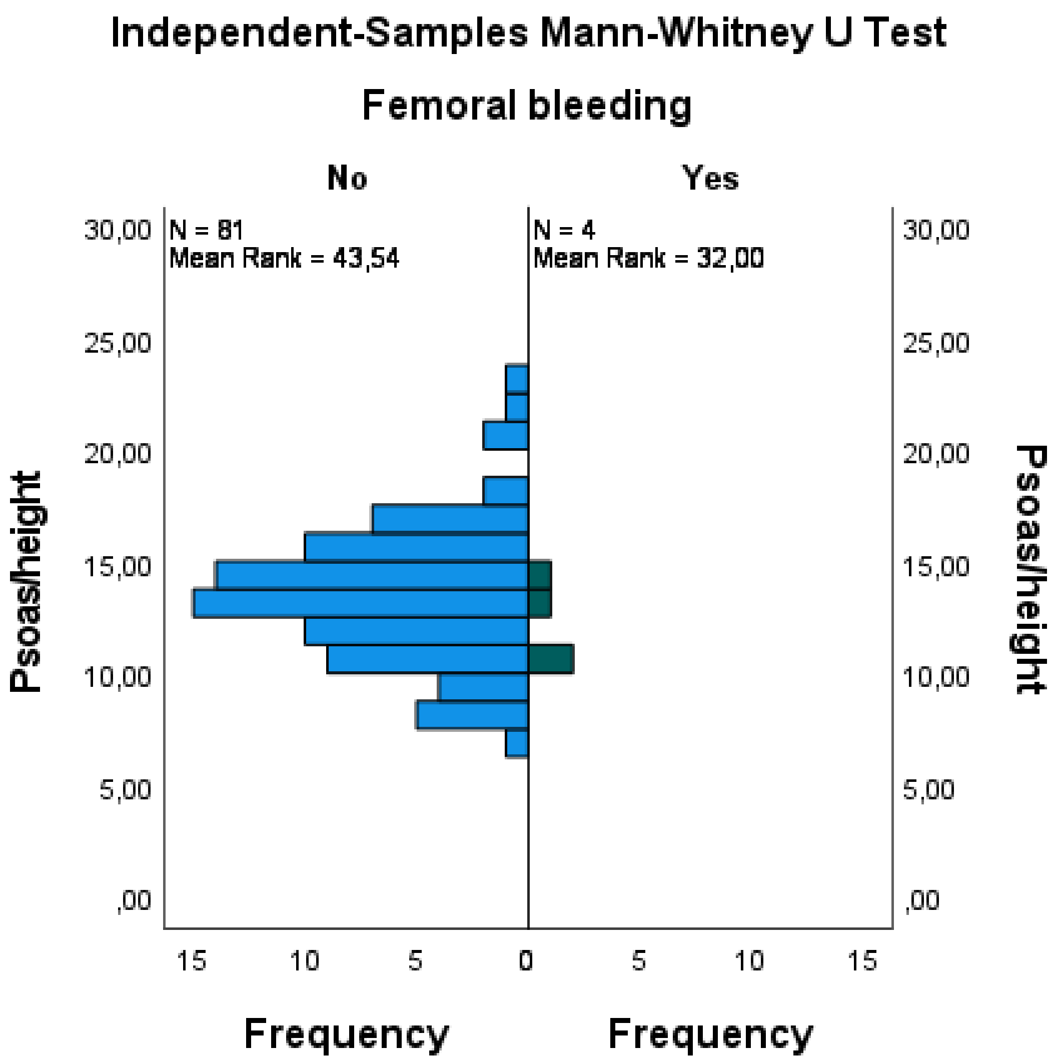

Anterior SAT distance across Femoral bleeding

Independent-Samples Mann-Whitney U Test Summary

Total N	85
Mann-Whitney U	120,500
Wilcoxon W	130,500
Test Statistic	120,500
Standard Error	48,180
Standardized Test Statistic	-,861
Asymptotic Sig.(2-sided test)	,389
Exact Sig.(2-sided test)	,404

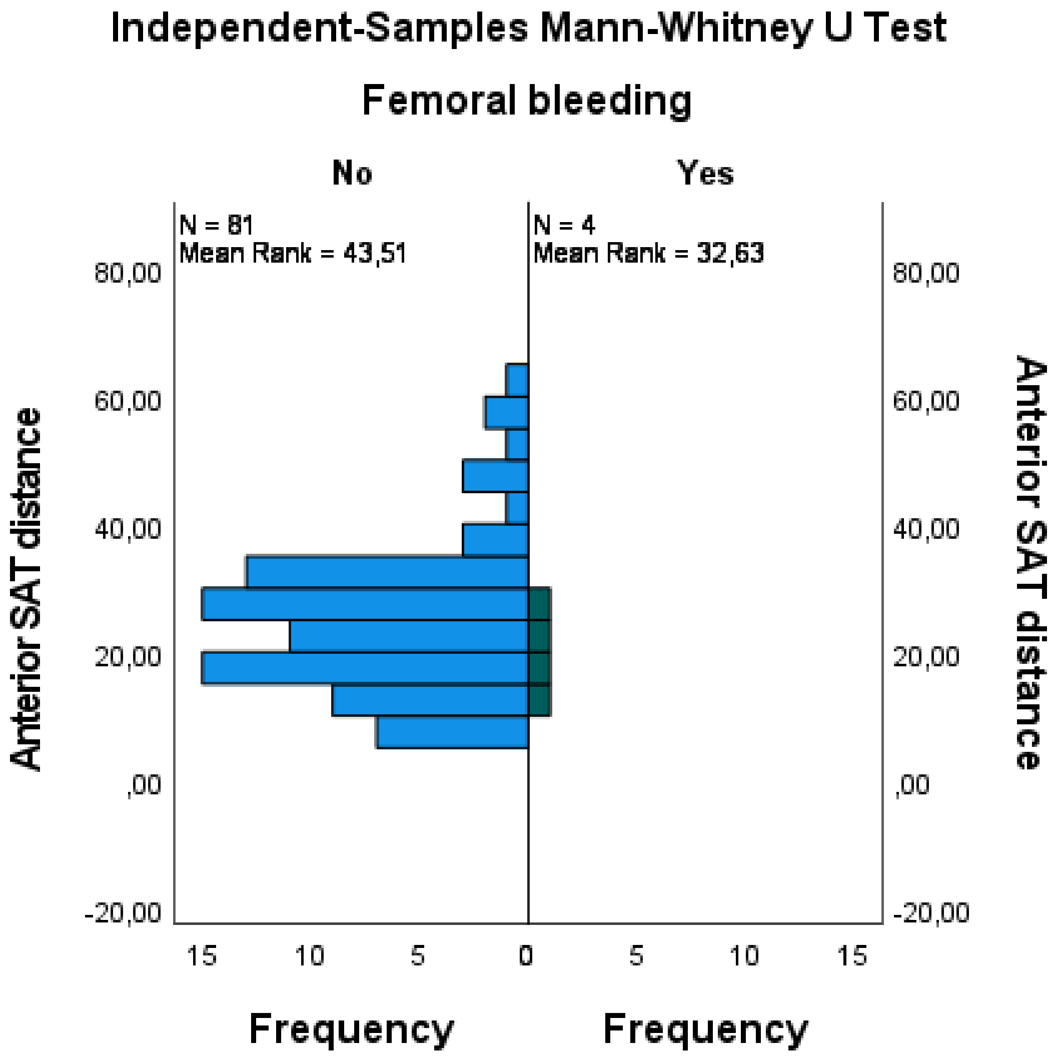

Posterior SAT distance across Femoral bleeding

Independent-Samples Mann-Whitney U Test Summary

Total N	85
Mann-Whitney U	139,500
Wilcoxon W	149,500
Test Statistic	139,500
Standard Error	48,182
Standardized Test Statistic	-,467
Asymptotic Sig.(2-sided test)	,641
Exact Sig.(2-sided test)	,652

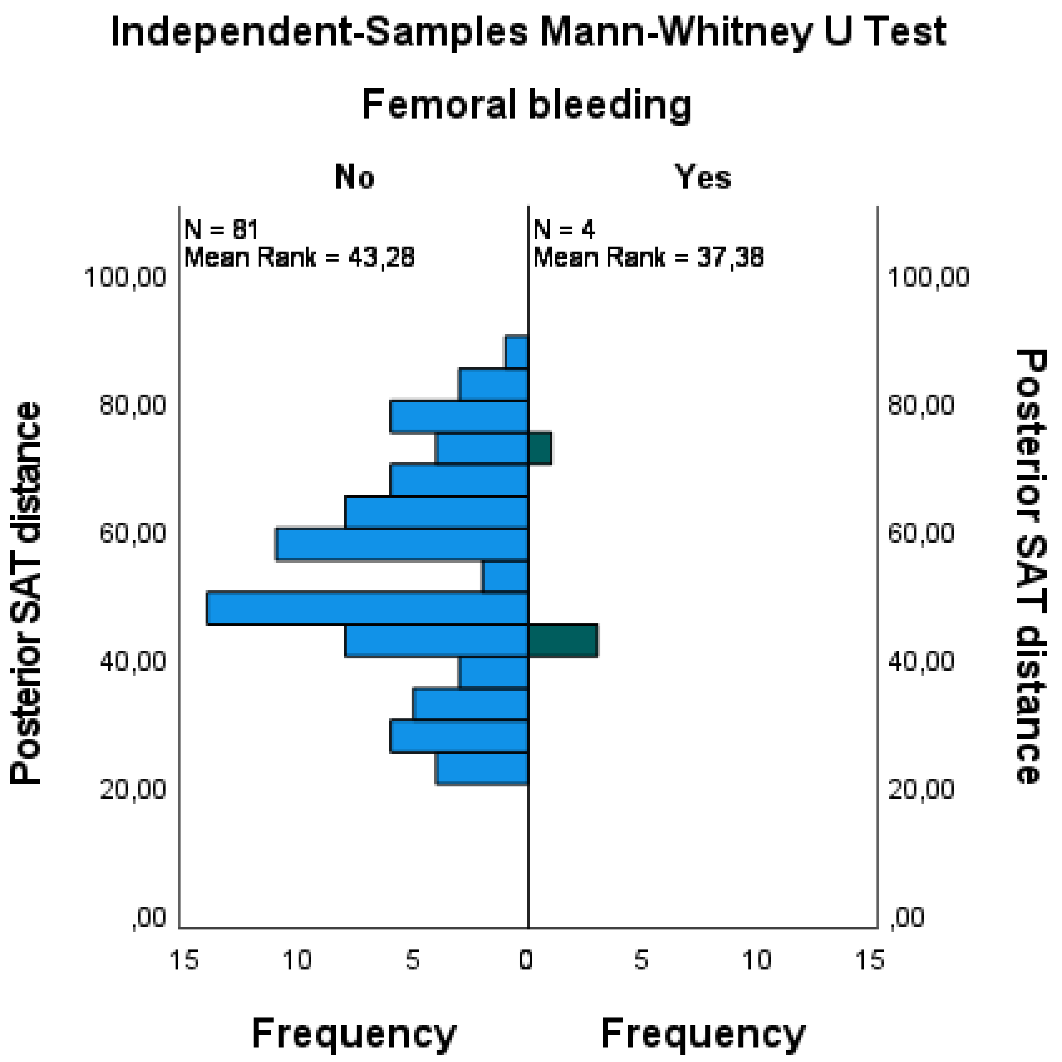

Anterior+Posterior SAT distance across Femoral bleeding

Independent-Samples Mann-Whitney U Test Summary

Total N	85
Mann-Whitney U	134,000
Wilcoxon W	144,000
Test Statistic	134,000
Standard Error	48,185
Standardized Test Statistic	-,581
Asymptotic Sig.(2-sided test)	,561
Exact Sig.(2-sided test)	,582

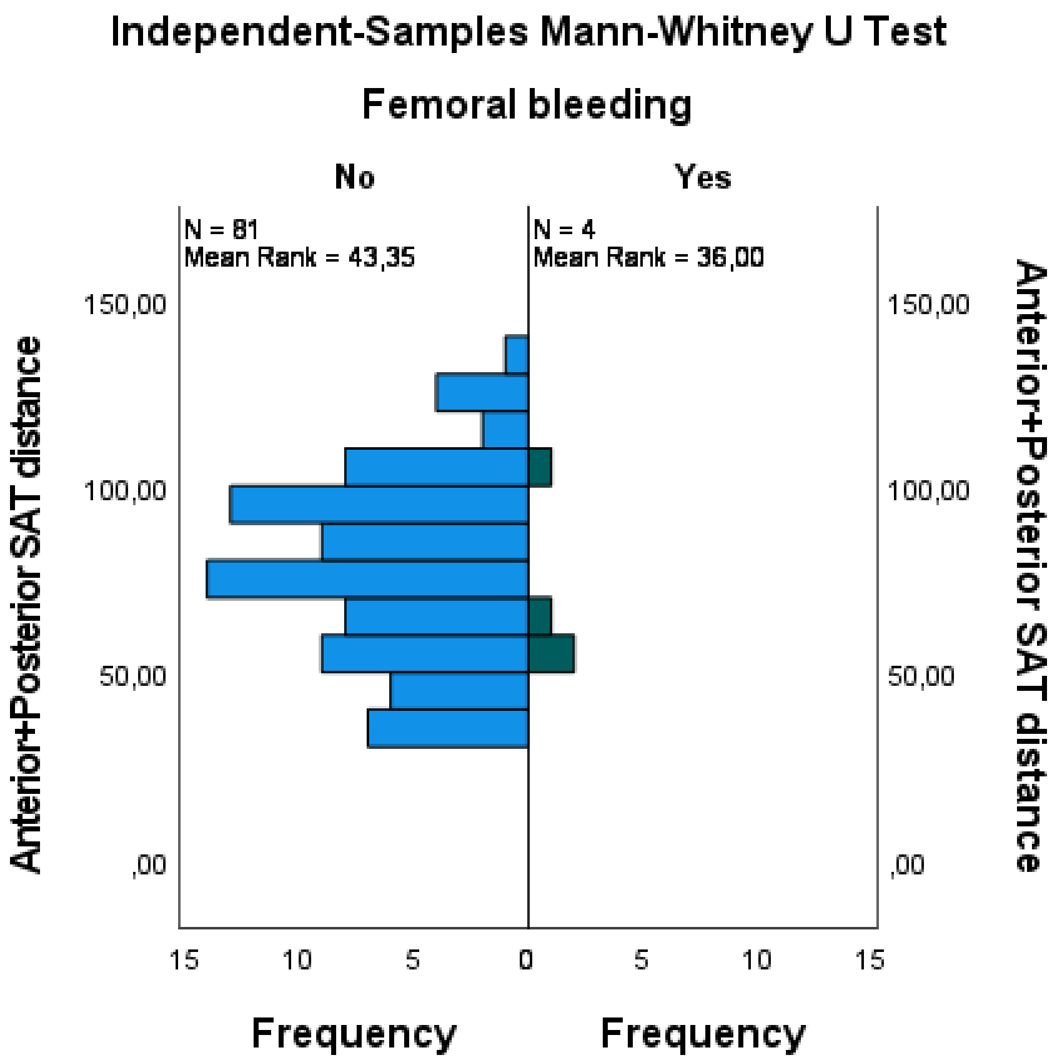

VAT distance across Femoral bleeding

Independent-Samples Mann-Whitney U Test Summary

Total N	84
Mann-Whitney U	139,000
Wilcoxon W	149,000
Test Statistic	139,000
Standard Error	47,600
Standardized Test Statistic	-,441
Asymptotic Sig.(2-sided test)	,659
Exact Sig.(2-sided test)	,678

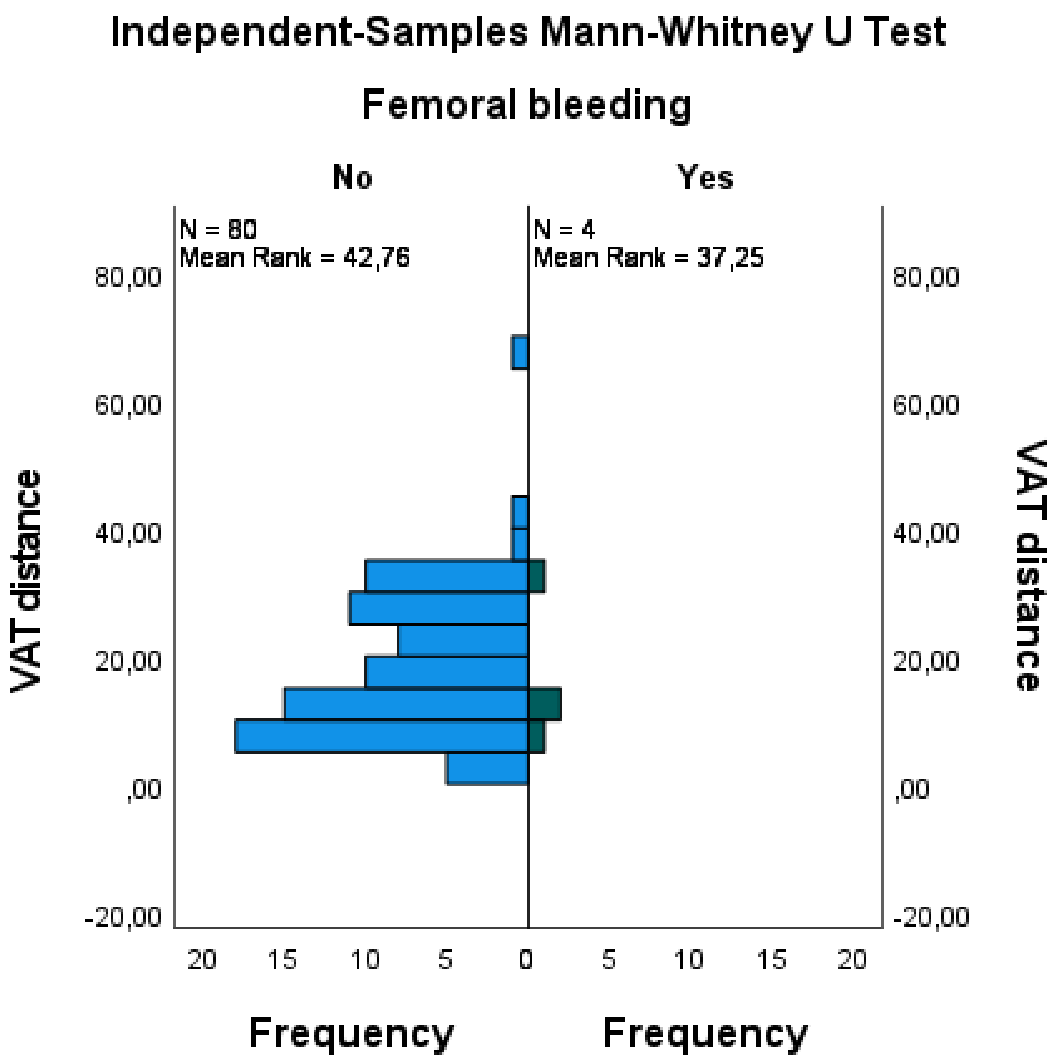

Right common femoral artery area (mm2) across Femoral bleeding

Independent-Samples Mann-Whitney U Test Summary

Total N	85
Mann-Whitney U	171,500
Wilcoxon W	181,500
Test Statistic	171,500
Standard Error	48,179
Standardized Test Statistic	,197
Asymptotic Sig.(2-sided test)	,844
Exact Sig.(2-sided test)	,849

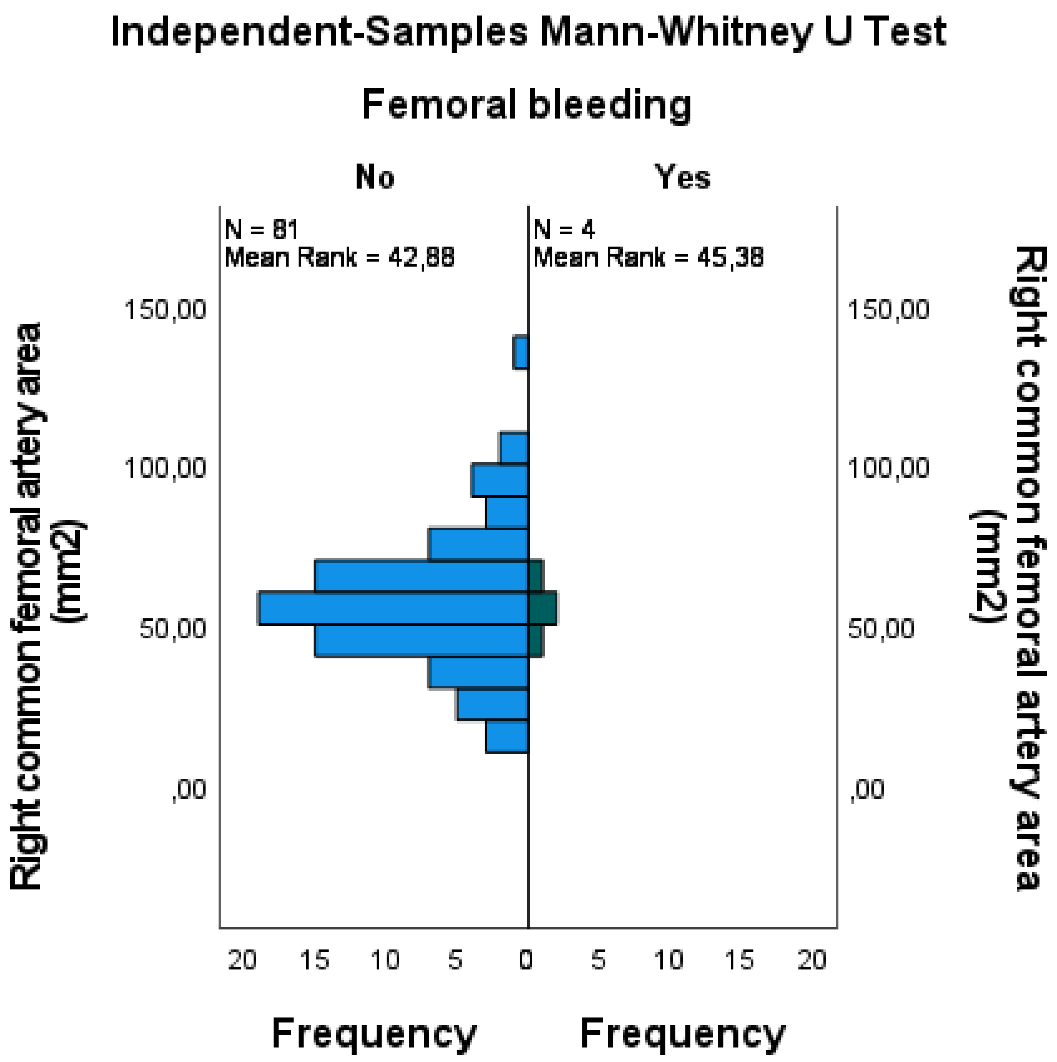

Left common femoral artery area (mm2) across Femoral bleeding

Independent-Samples Mann-Whitney U Test Summary

Total N	85
Mann-Whitney U	198,000
Wilcoxon W	208,000
Test Statistic	198,000
Standard Error	48,175
Standardized Test Statistic	,747
Asymptotic Sig.(2-sided test)	,455
Exact Sig.(2-sided test)	,476

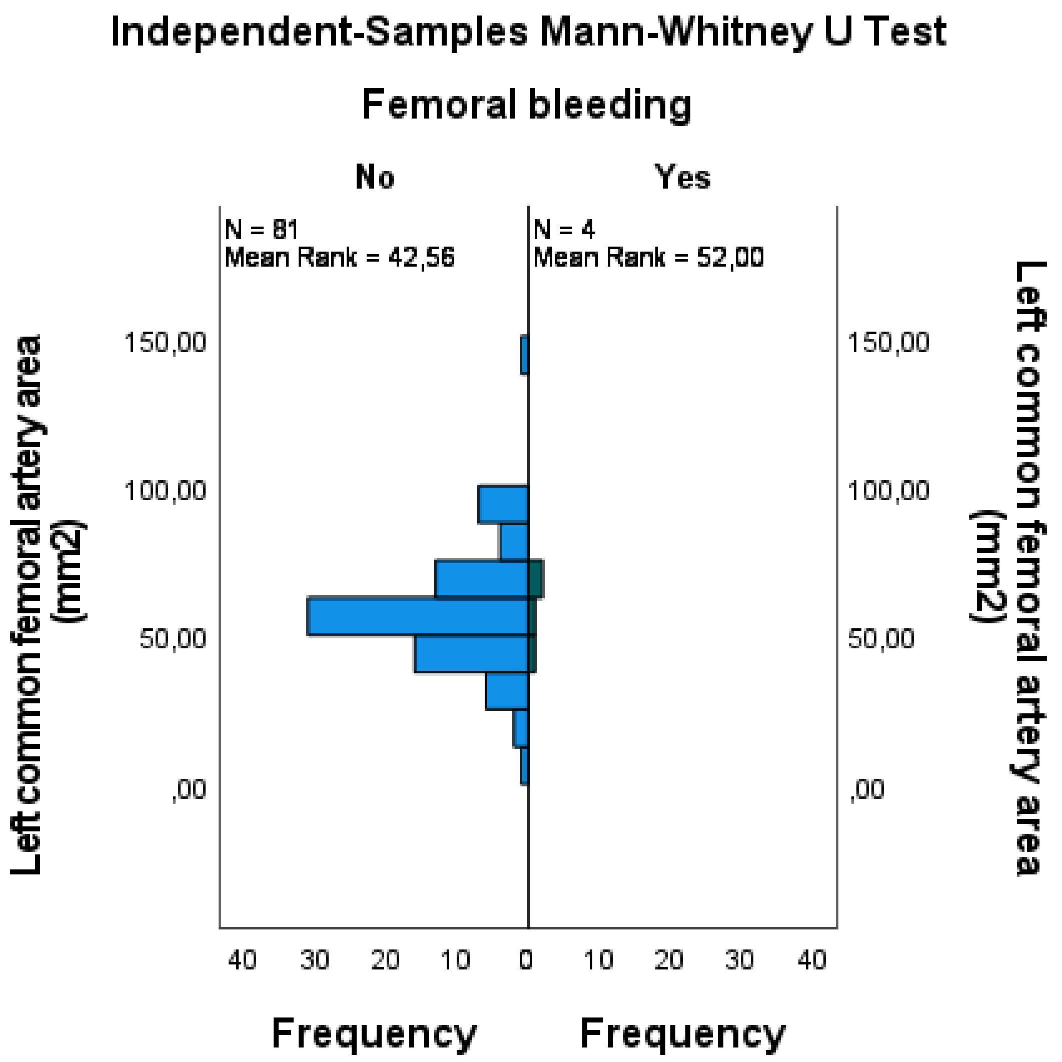

FAT area (cm2) across Femoral bleeding

Independent-Samples Mann-Whitney U Test Summary

Total N	85
Mann-Whitney U	124,500
Wilcoxon W	134,500
Test Statistic	124,500
Standard Error	48,187
Standardized Test Statistic	-,778
Asymptotic Sig.(2-sided test)	,436
Exact Sig.(2-sided test)	,451

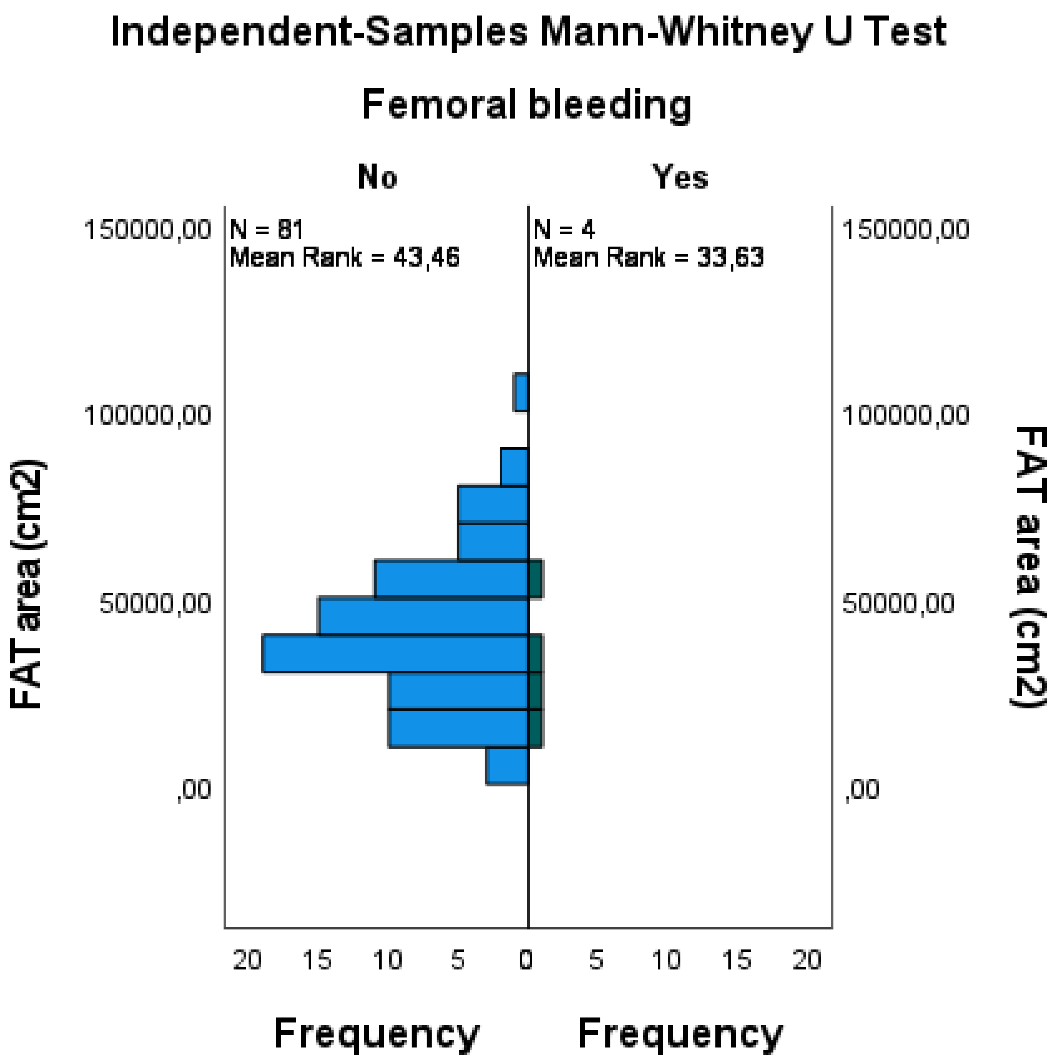

SAT area (cm2) across Femoral bleeding

Independent-Samples Mann-Whitney U Test Summary

Total N	85
Mann-Whitney U	143,000
Wilcoxon W	153,000
Test Statistic	143,000
Standard Error	48,187
Standardized Test Statistic	-,394
Asymptotic Sig.(2-sided test)	,693
Exact Sig.(2-sided test)	,711

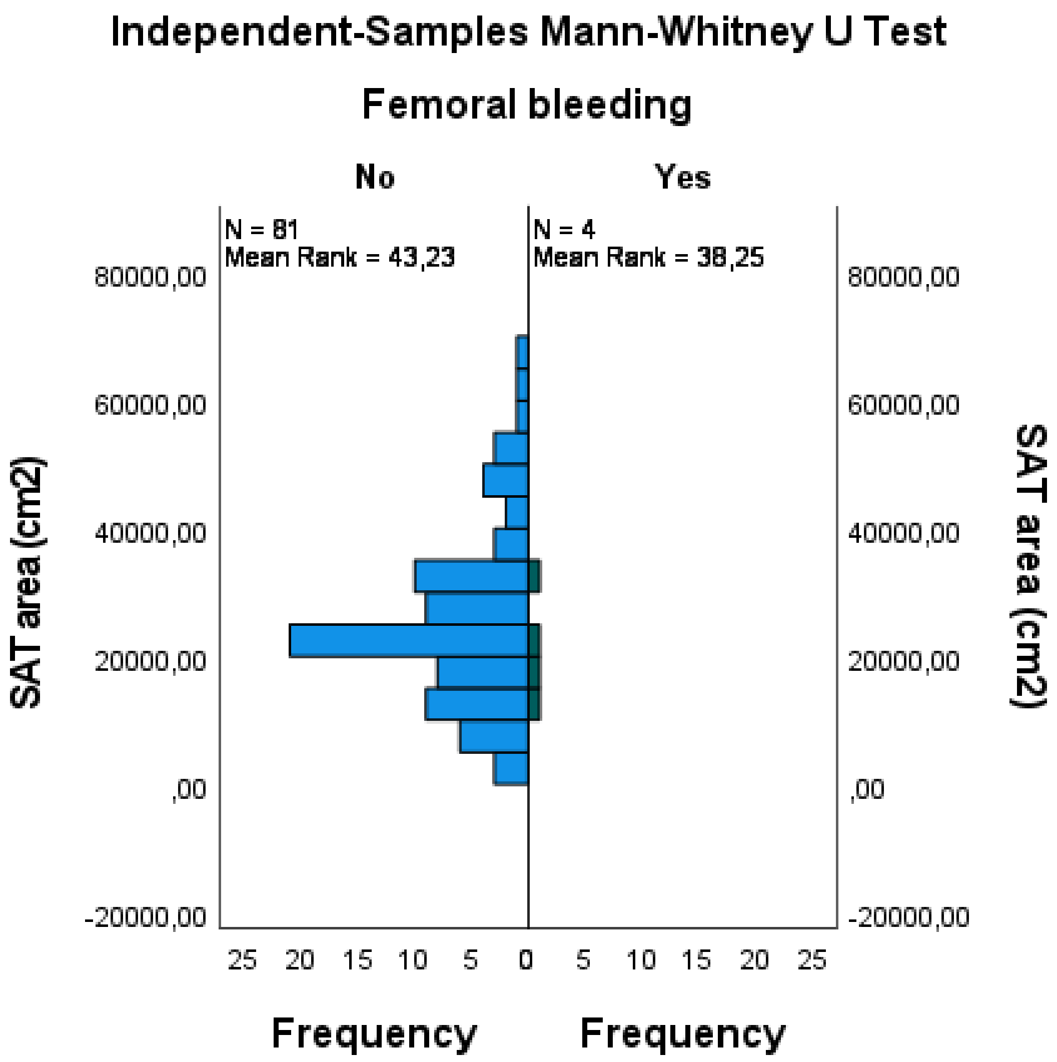

VAT area (cm2) across Femoral bleeding

Independent-Samples Mann-Whitney U Test Summary

Total N	84
Mann-Whitney U	115,000
Wilcoxon W	125,000
Test Statistic	115,000
Standard Error	47,610
Standardized Test Statistic	-,945
Asymptotic Sig.(2-sided test)	,345
Exact Sig.(2-sided test)	,364

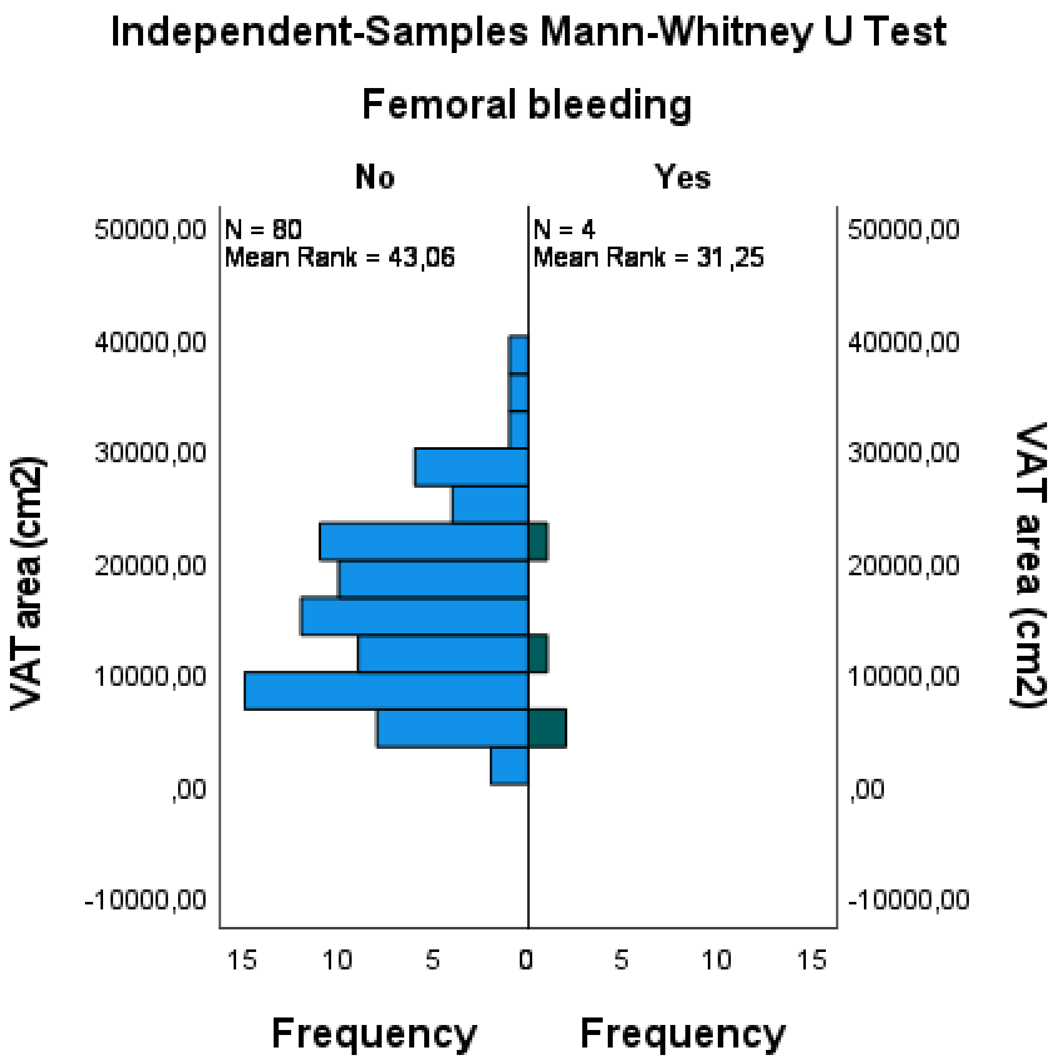

Right Psoas muscle area (cm2) across Femoral bleeding

Independent-Samples Mann-Whitney U Test Summary

Total N	85
Mann-Whitney U	119,000
Wilcoxon W	129,000
Test Statistic	119,000
Standard Error	48,186
Standardized Test Statistic	-,892
Asymptotic Sig.(2-sided test)	,372
Exact Sig.(2-sided test)	,392

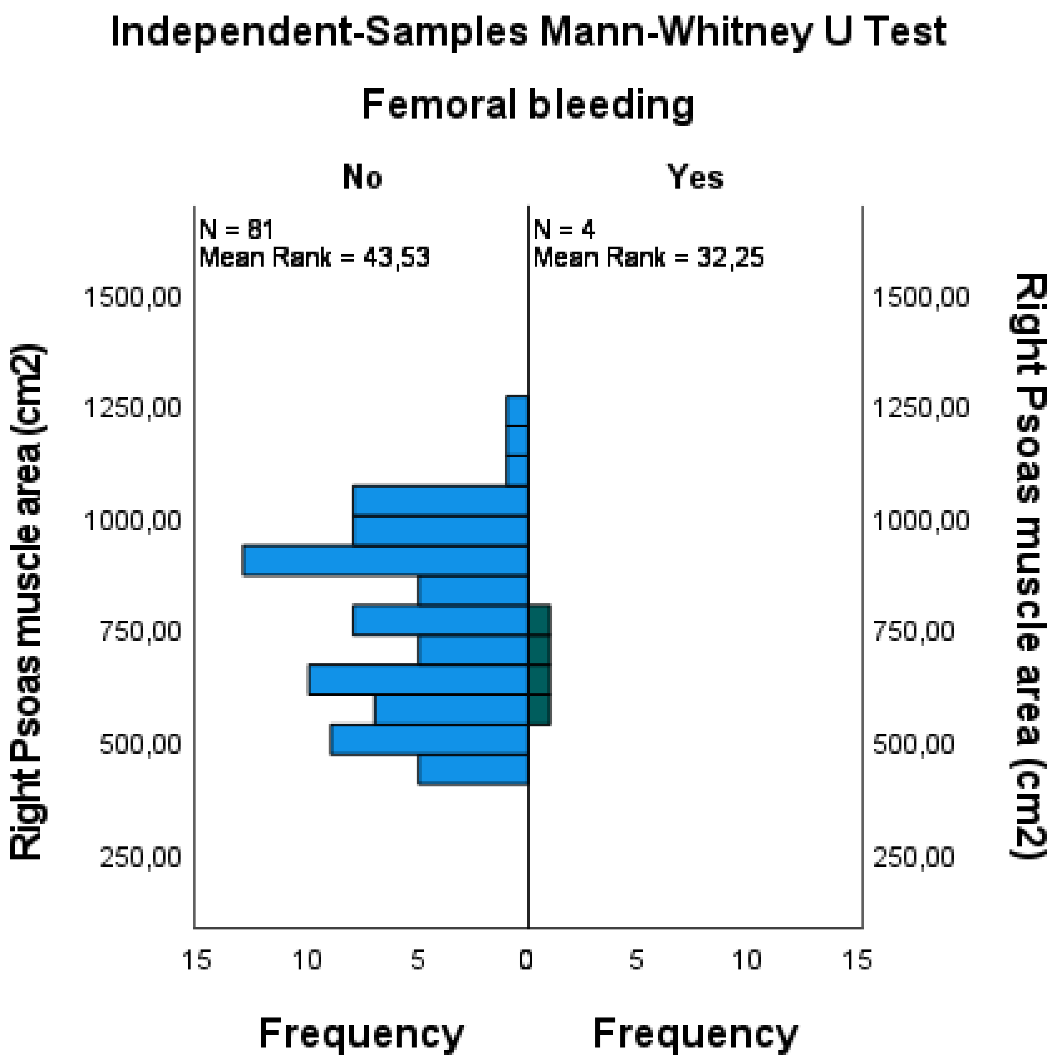

Left Psoas muscle area (cm2) across Femoral bleeding

Independent-Samples Mann-Whitney U Test Summary

Total N	85
Mann-Whitney U	100,000
Wilcoxon W	110,000
Test Statistic	100,000
Standard Error	48,187
Standardized Test Statistic	-1,287
Asymptotic Sig.(2-sided test)	,198
Exact Sig.(2-sided test)	,211

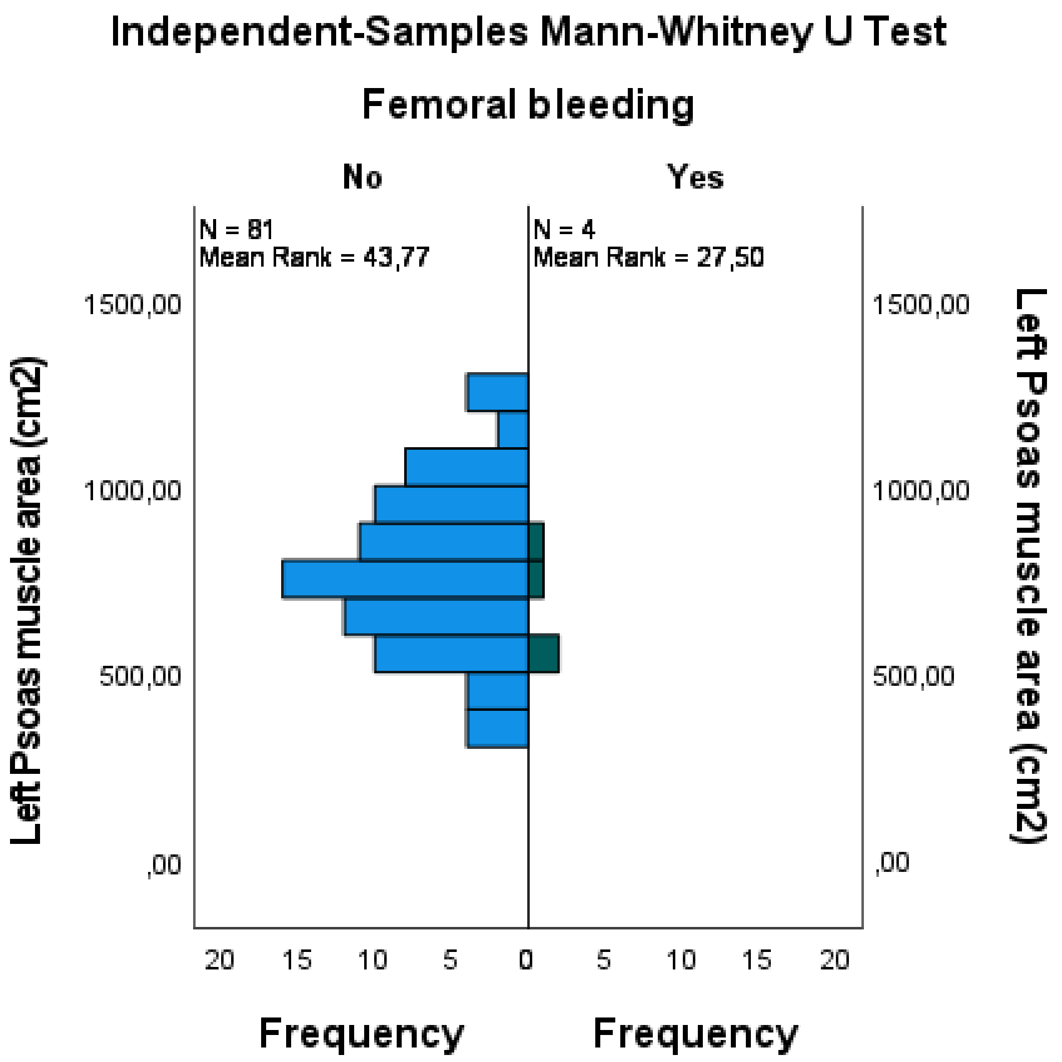

FAT mean density (HU) across Femoral bleeding

Independent-Samples Mann-Whitney U Test Summary

Total N	85
Mann-Whitney U	201,000
Wilcoxon W	211,000
Test Statistic	201,000
Standard Error	48,179
Standardized Test Statistic	,809
Asymptotic Sig.(2-sided test)	,418
Exact Sig.(2-sided test)	,439

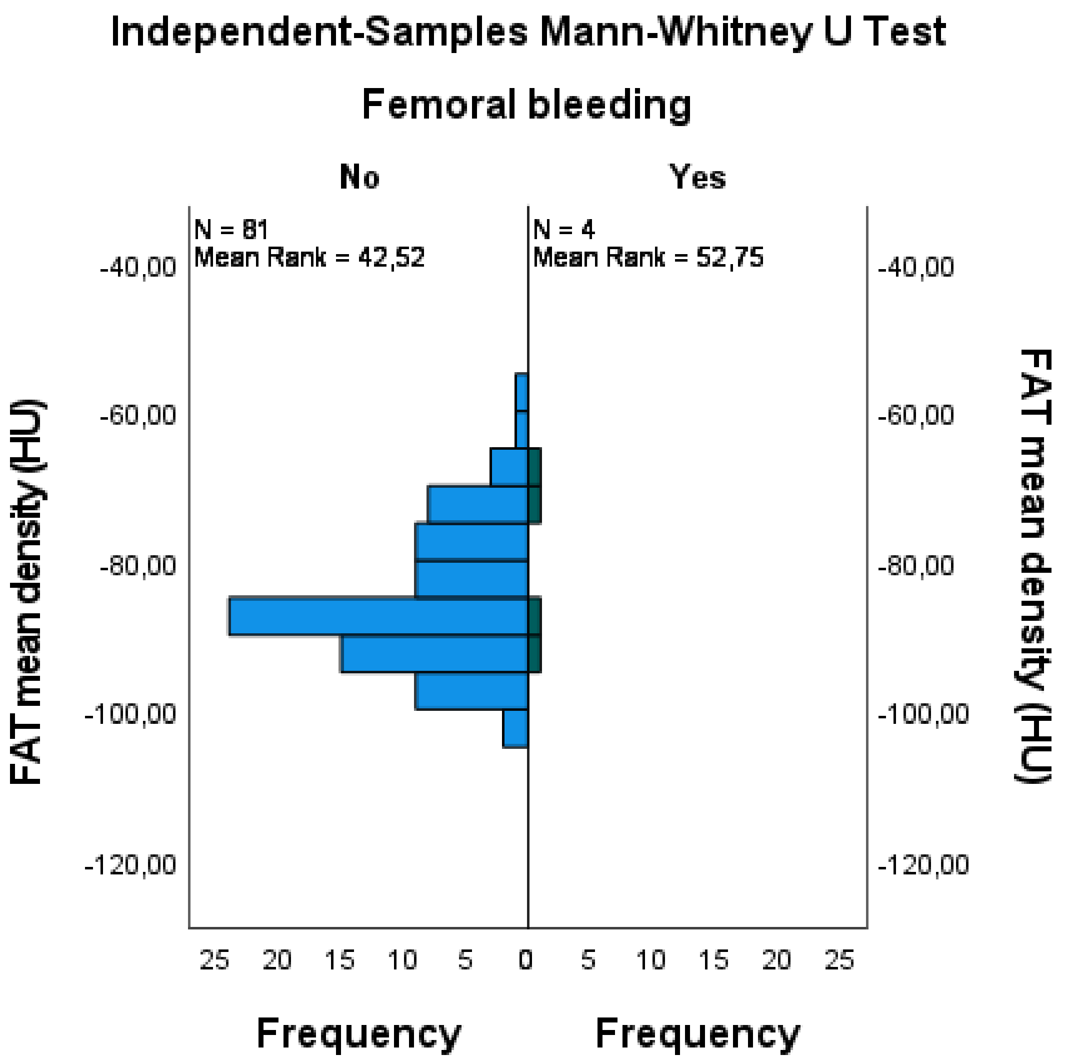

FAT median density (HU) across Femoral bleeding

Independent-Samples Mann-Whitney U Test Summary

Total N	85
Mann-Whitney U	209,000
Wilcoxon W	219,000
Test Statistic	209,000
Standard Error	48,129
Standardized Test Statistic	,977
Asymptotic Sig.(2-sided test)	,329
Exact Sig.(2-sided test)	,348

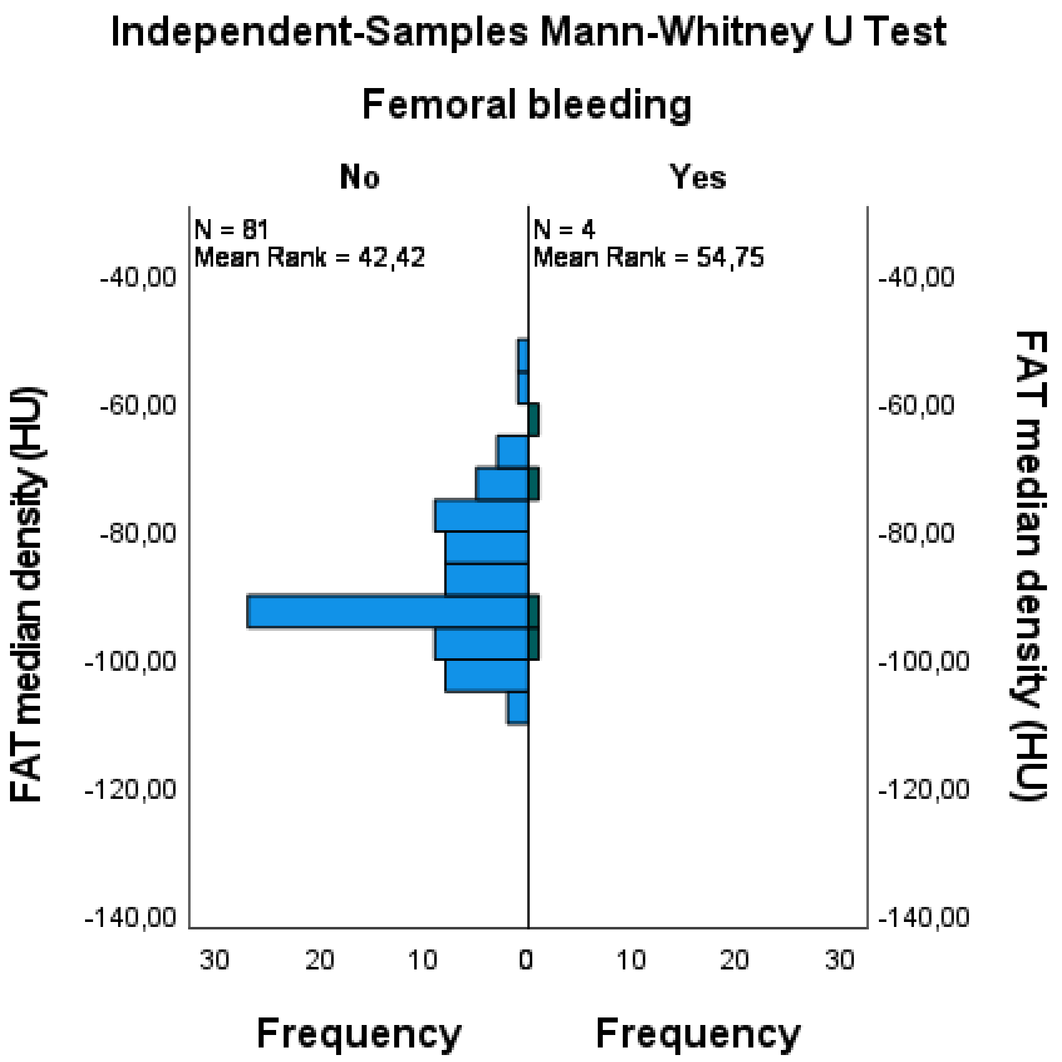

FAT density standard deviation across Femoral bleeding

Independent-Samples Mann-Whitney U Test Summary

Total N	85
Mann-Whitney U	185,000
Wilcoxon W	195,000
Test Statistic	185,000
Standard Error	48,187
Standardized Test Statistic	,477
Asymptotic Sig.(2-sided test)	,633
Exact Sig.(2-sided test)	,652

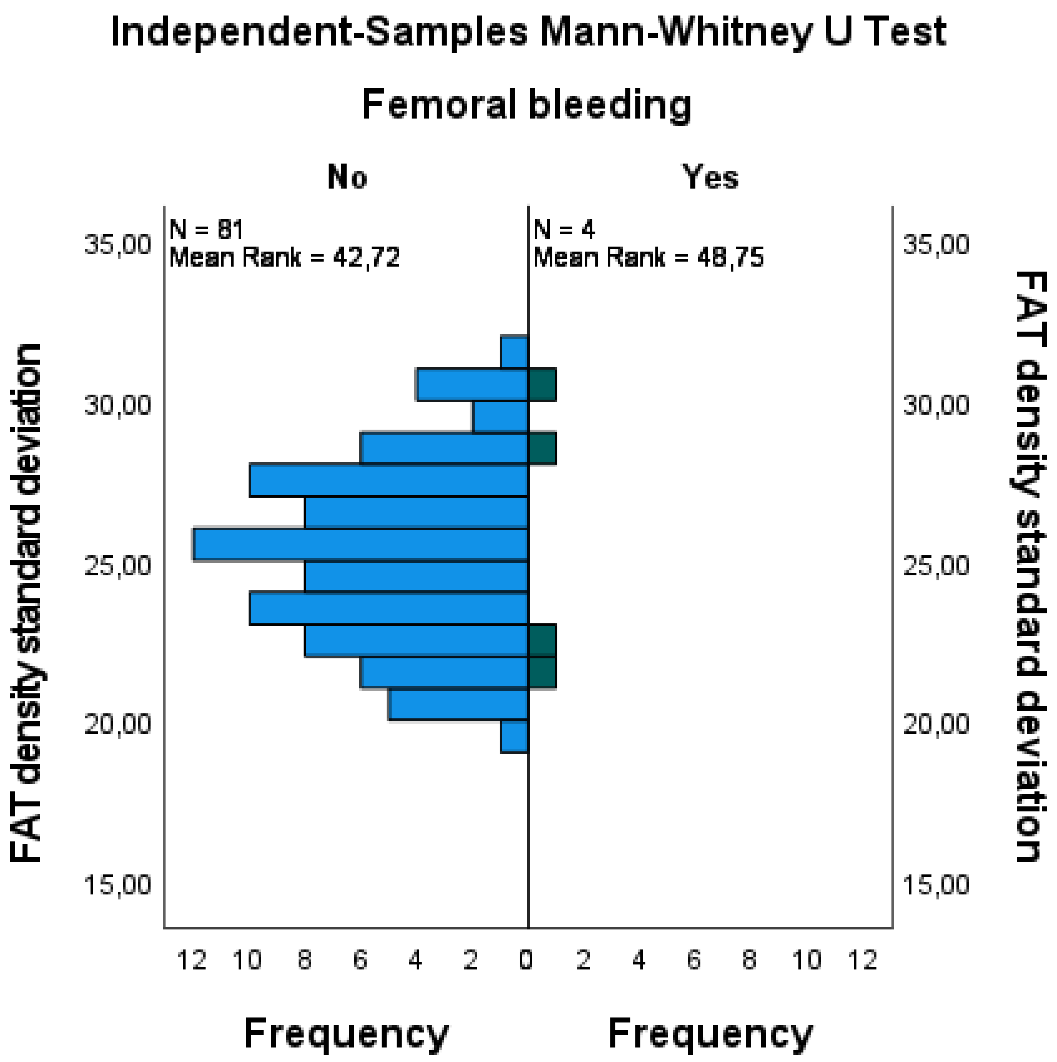

SAT mean density (HU) across Femoral bleeding

Independent-Samples Mann-Whitney U Test Summary

Total N	85
Mann-Whitney U	188,000
Wilcoxon W	198,000
Test Statistic	188,000
Standard Error	48,181
Standardized Test Statistic	,540
Asymptotic Sig.(2-sided test)	,589
Exact Sig.(2-sided test)	,610

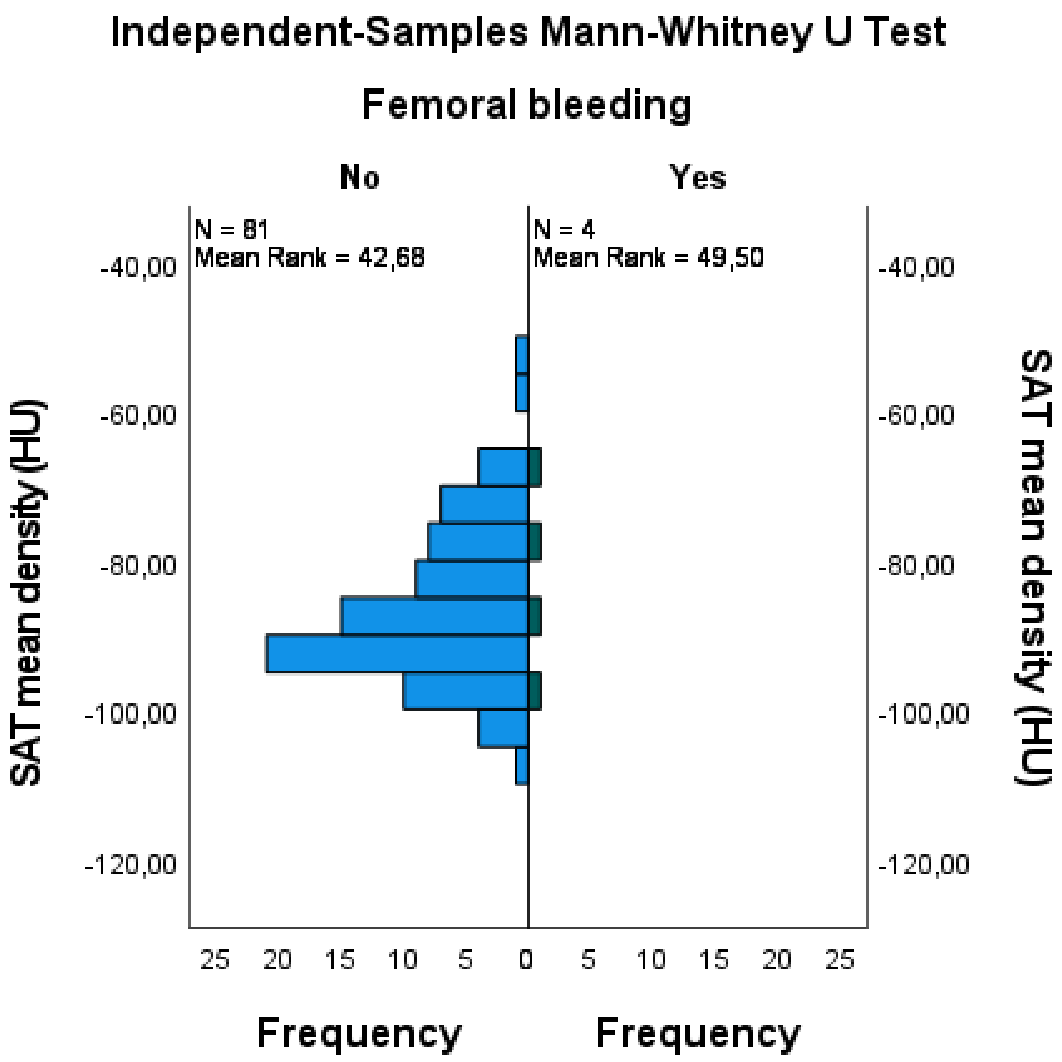

SAT median density (HU) across Femoral bleeding

Independent-Samples Mann-Whitney U Test Summary

Total N	83
Mann-Whitney U	189,000
Wilcoxon W	199,000
Test Statistic	189,000
Standard Error	46,995
Standardized Test Statistic	,660
Asymptotic Sig.(2-sided test)	,509
Exact Sig.(2-sided test)	,531

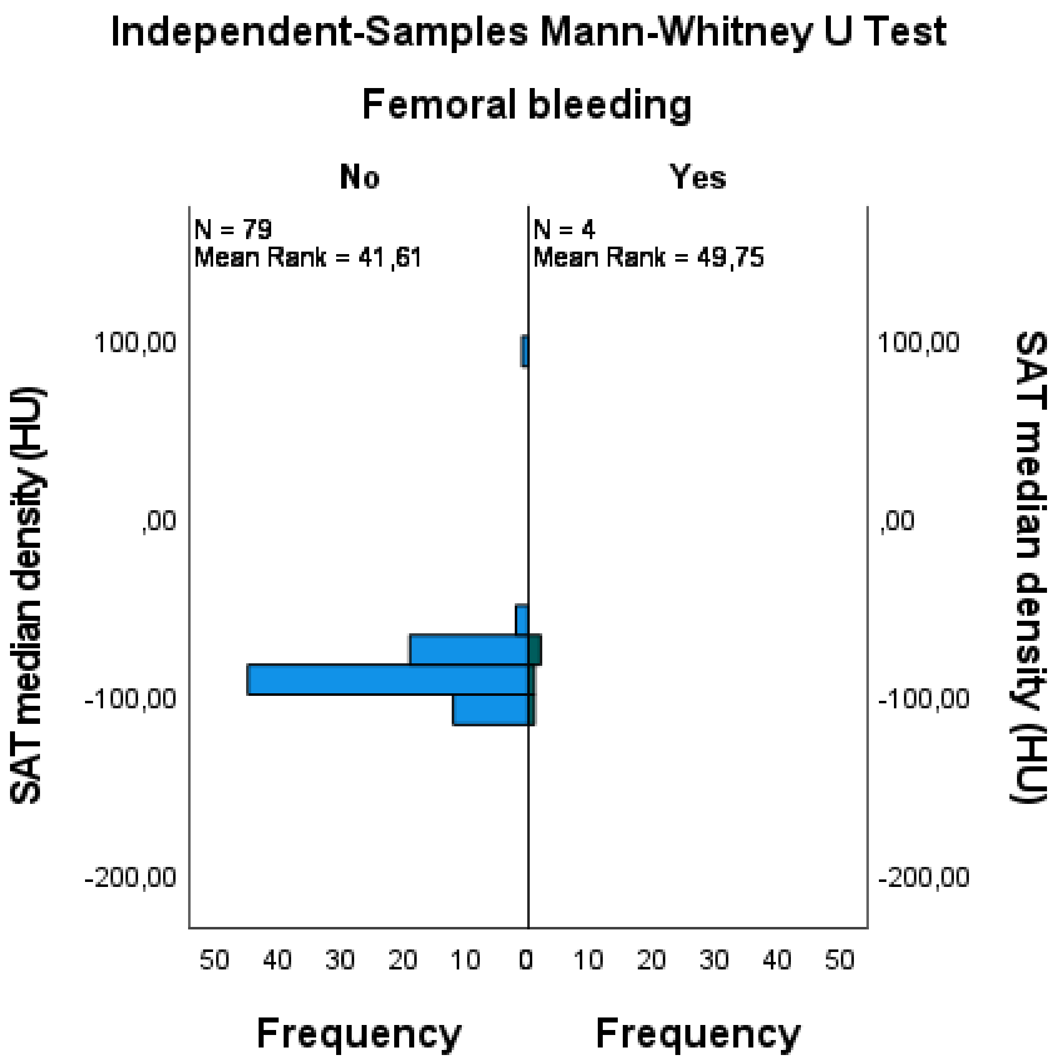

SAT density standard deviation across Femoral bleeding

Independent-Samples Mann-Whitney U Test Summary

Total N	84
Mann-Whitney U	177,000
Wilcoxon W	187,000
Test Statistic	177,000
Standard Error	47,610
Standardized Test Statistic	,357
Asymptotic Sig.(2-sided test)	,721
Exact Sig.(2-sided test)	,738

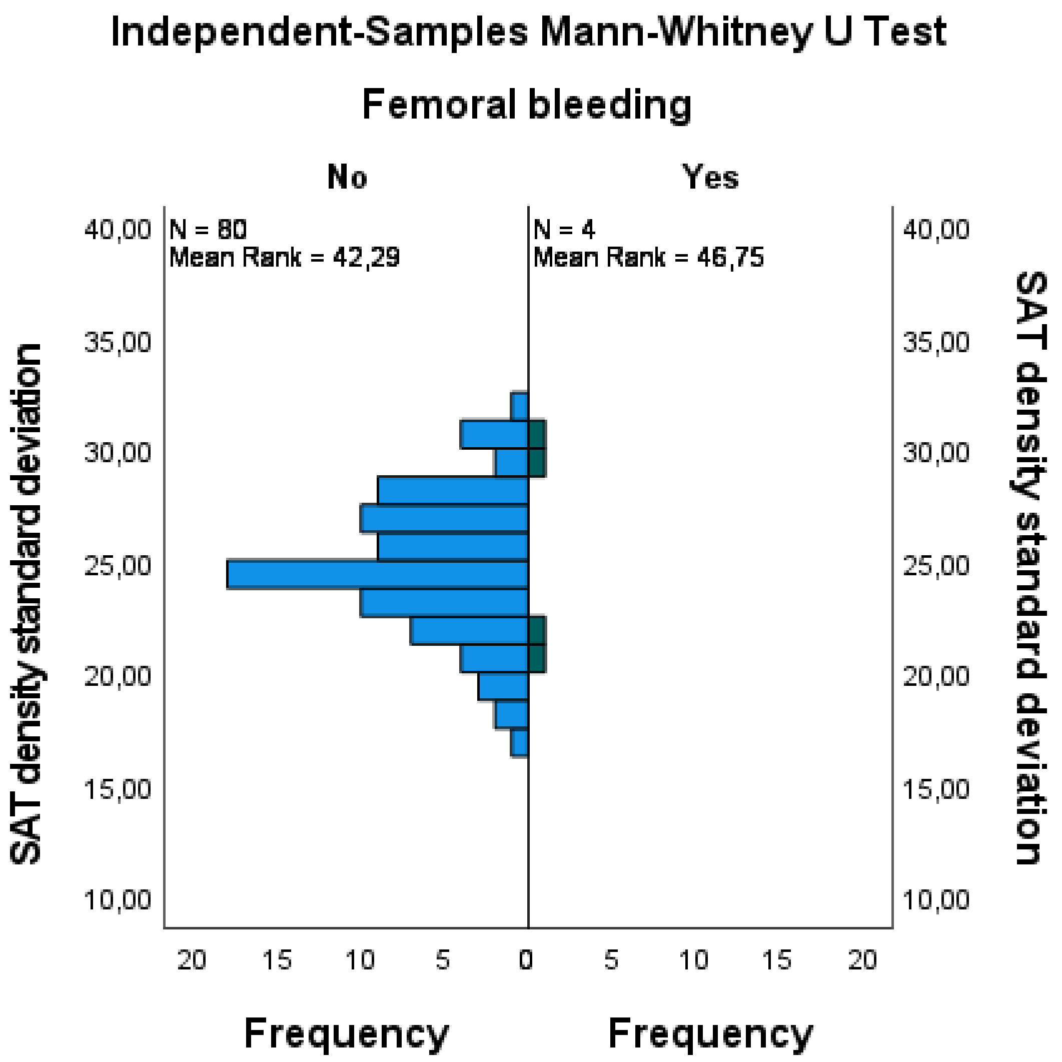

VAT mean density (HU) across Femoral bleeding

Independent-Samples Mann-Whitney U Test Summary

Total N	85
Mann-Whitney U	204,500
Wilcoxon W	214,500
Test Statistic	204,500
Standard Error	48,178
Standardized Test Statistic	,882
Asymptotic Sig.(2-sided test)	,378
Exact Sig.(2-sided test)	,392

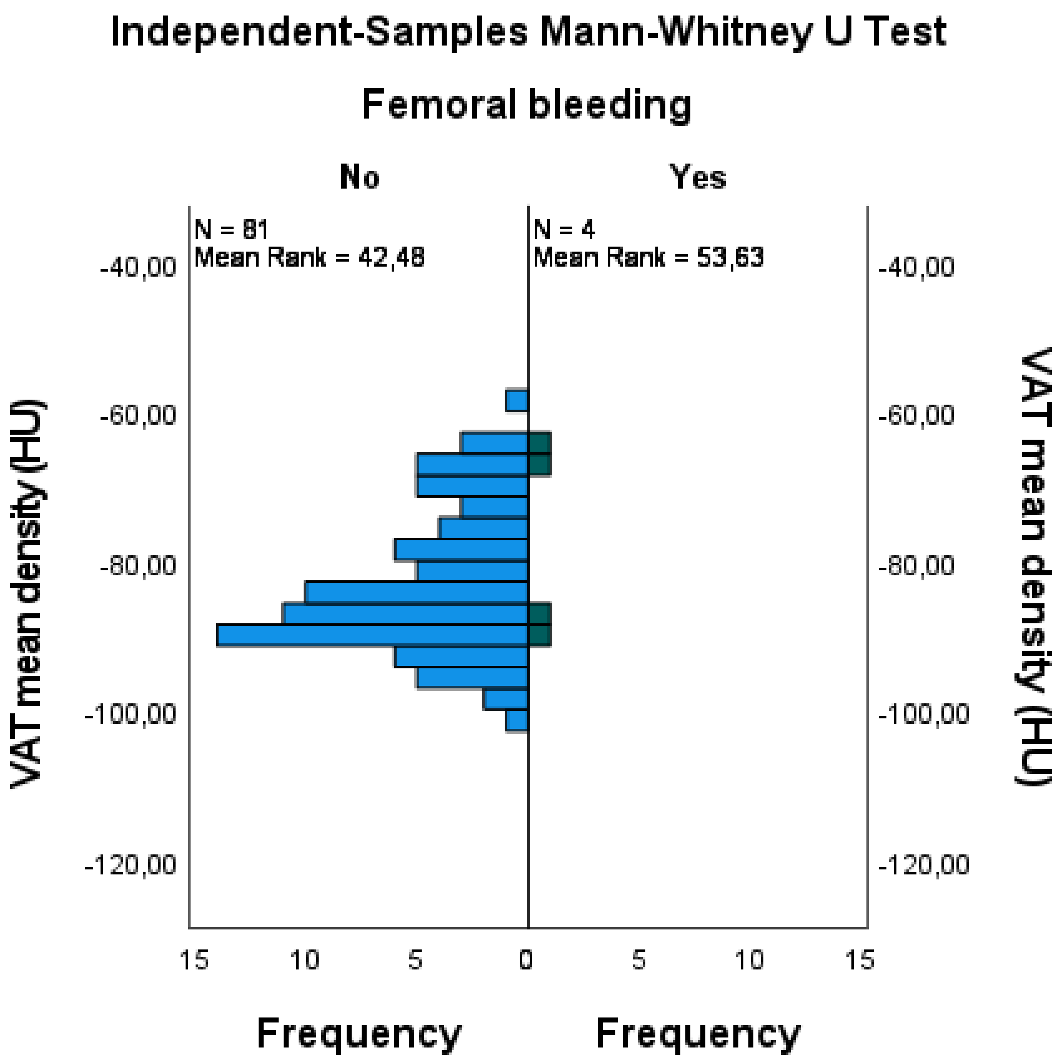

VAT median density (HU) across Femoral bleeding

Independent-Samples Mann-Whitney U Test Summary

Total N	85
Mann-Whitney U	214,500
Wilcoxon W	224,500
Test Statistic	214,500
Standard Error	48,127
Standardized Test Statistic	1,091
Asymptotic Sig.(2-sided test)	,275
Exact Sig.(2-sided test)	,288

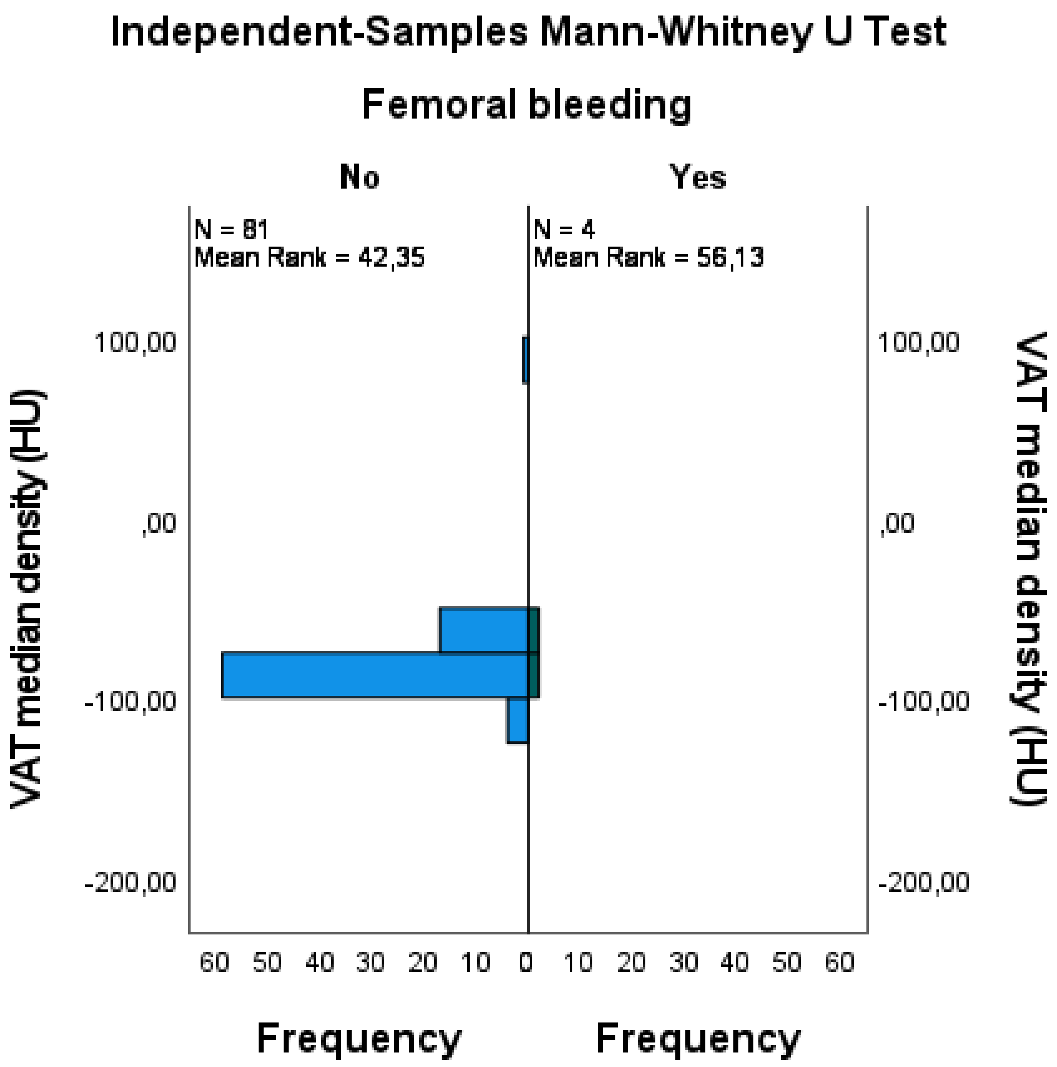

VAT density standard deviation across Femoral bleeding

Independent-Samples Mann-Whitney U Test Summary

Total N	84
Mann-Whitney U	179,000
Wilcoxon W	189,000
Test Statistic	179,000
Standard Error	47,610
Standardized Test Statistic	,399
Asymptotic Sig.(2-sided test)	,690
Exact Sig.(2-sided test)	,708

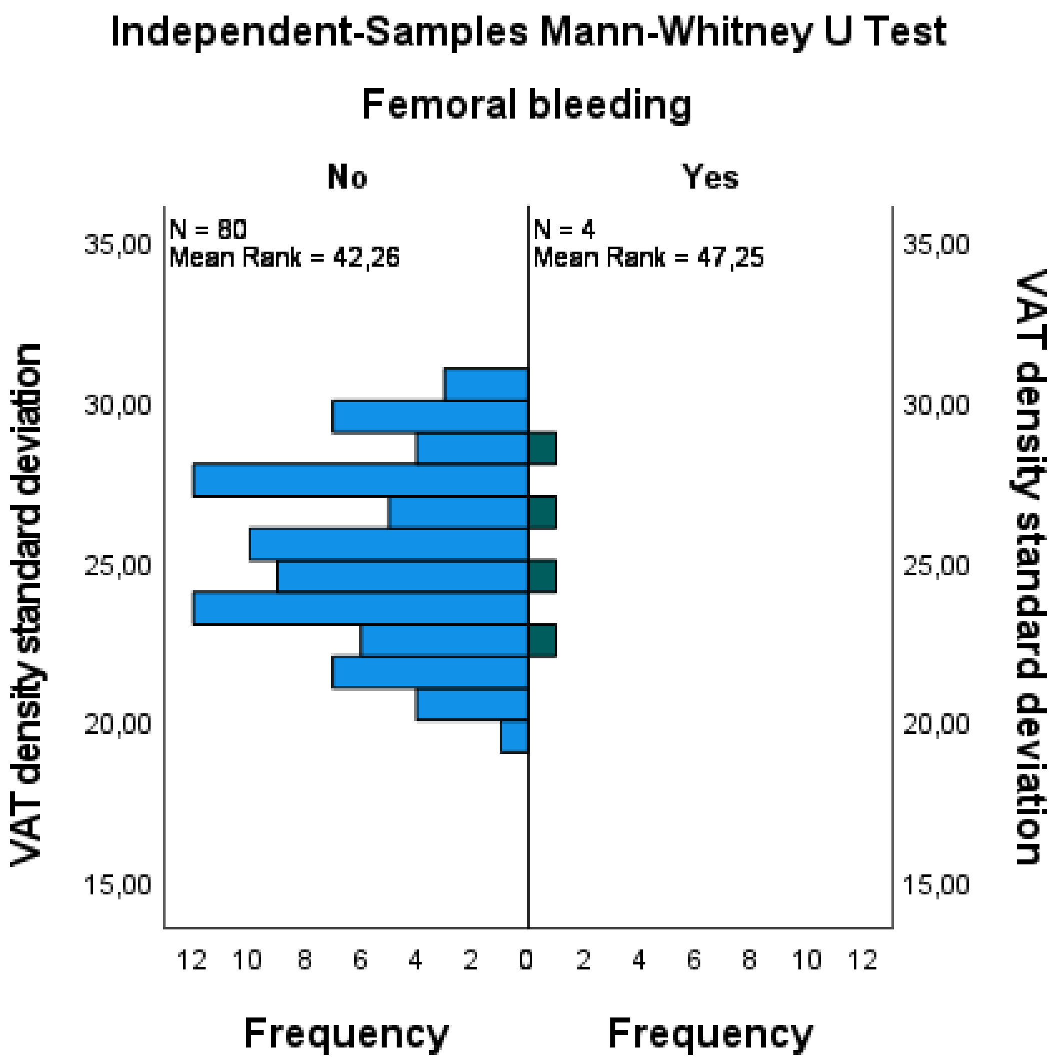

Right Psoas muscle mean density (HU) across Femoral bleeding

Independent-Samples Mann-Whitney U Test Summary

Total N	85
Mann-Whitney U	228,500
Wilcoxon W	238,500
Test Statistic	228,500
Standard Error	48,177
Standardized Test Statistic	1,380
Asymptotic Sig.(2-sided test)	,167
Exact Sig.(2-sided test)	,174

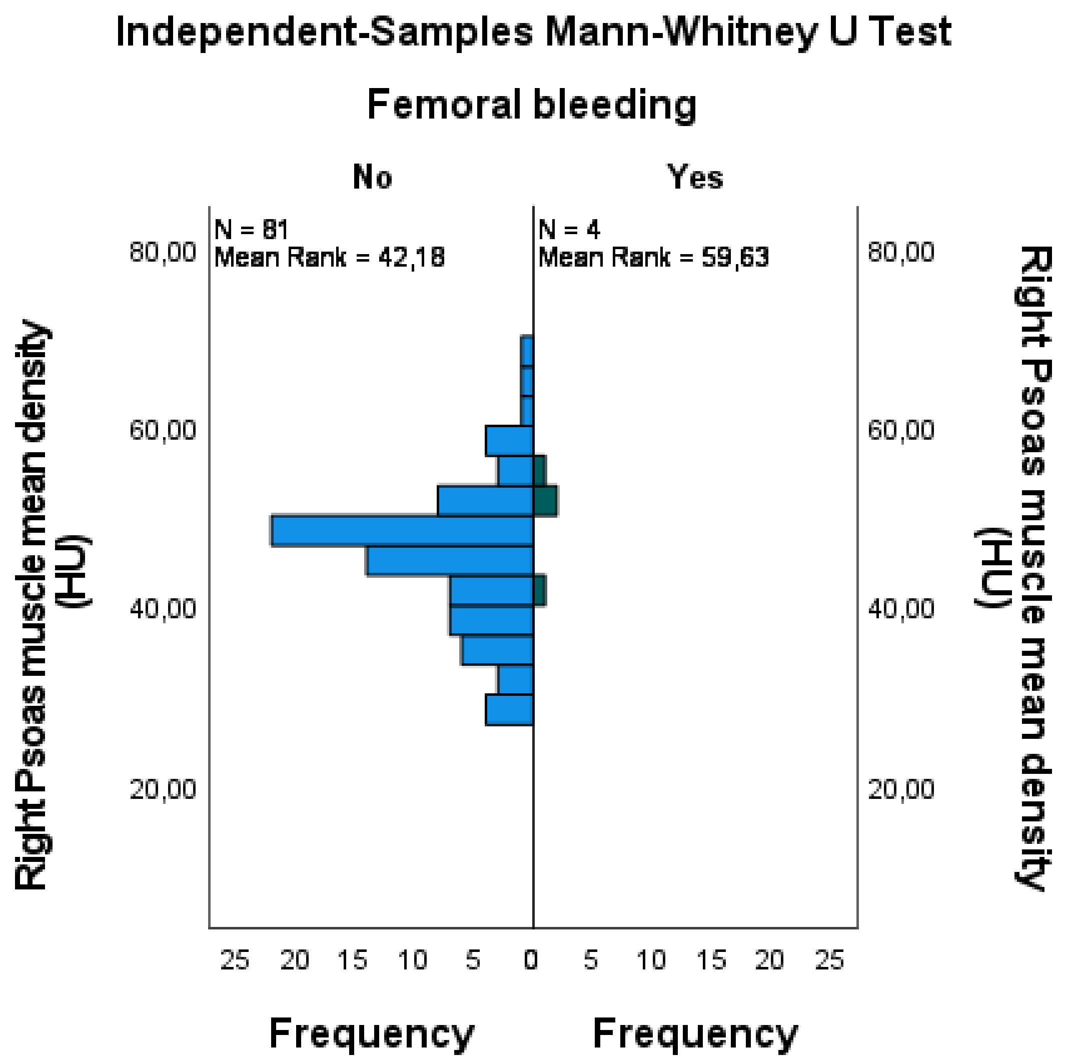

Right Psoas muscle median density (HU) across Femoral bleeding

Independent-Samples Mann-Whitney U Test Summary

Total N	85
Mann-Whitney U	197,000
Wilcoxon W	207,000
Test Statistic	197,000
Standard Error	48,141
Standardized Test Statistic	,727
Asymptotic Sig.(2-sided test)	,467
Exact Sig.(2-sided test)	,489

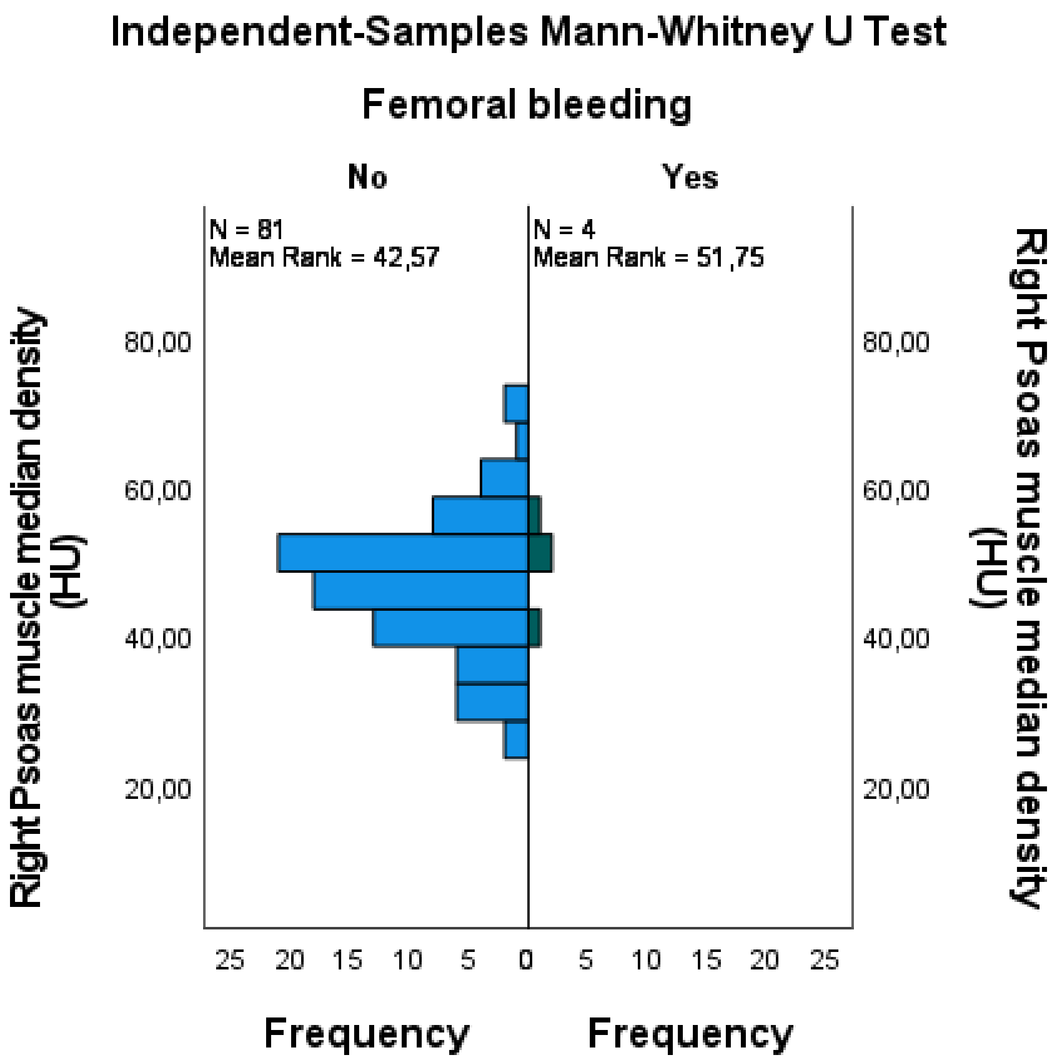

Right Psoas muscle density standard deviation across Femoral bleeding

Independent-Samples Mann-Whitney U Test Summary

Total N	85
Mann-Whitney U	160,500
Wilcoxon W	170,500
Test Statistic	160,500
Standard Error	48,186
Standardized Test Statistic	-,031
Asymptotic Sig.(2-sided test)	,975
Exact Sig.(2-sided test)	,976

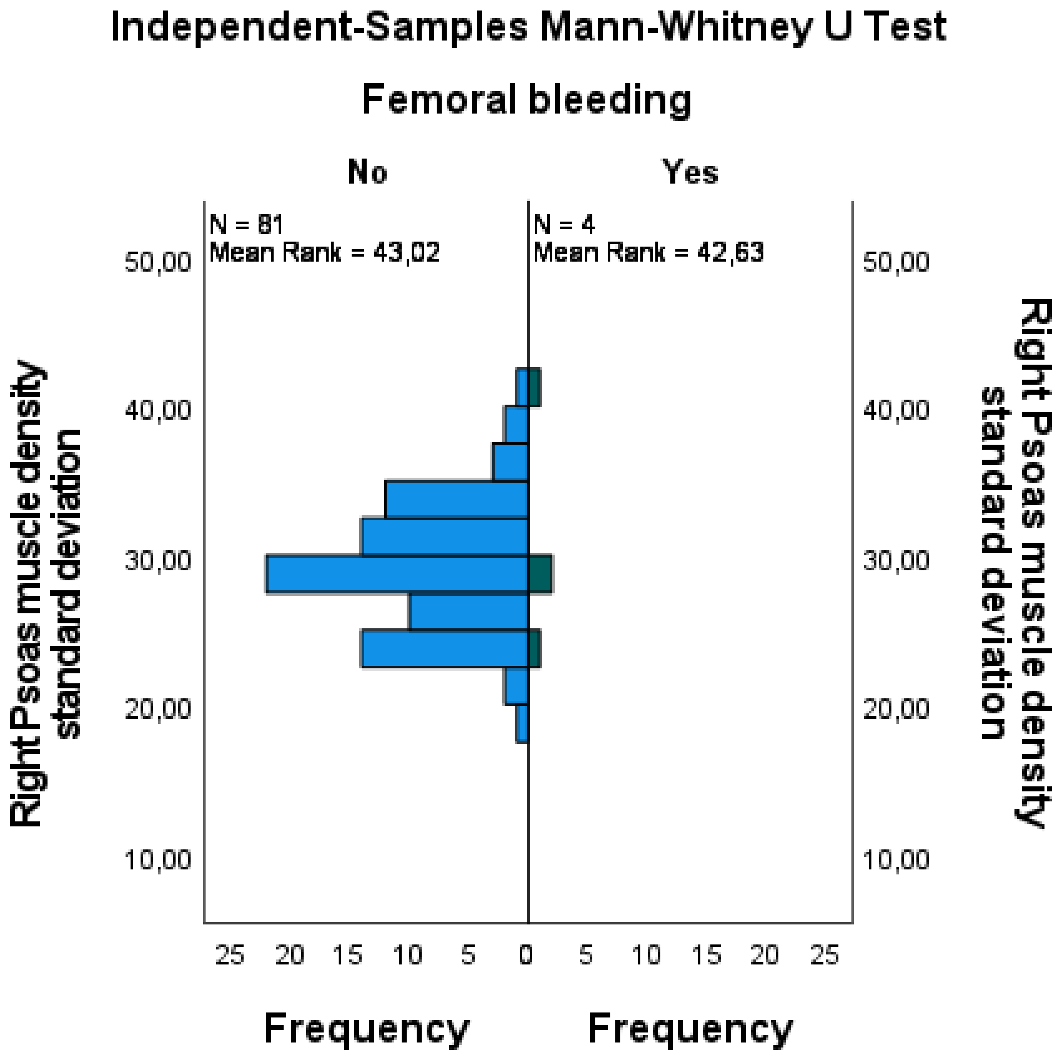

Left Psoas muscle mean density (HU) across Femoral bleeding

Independent-Samples Mann-Whitney U Test Summary

Total N	85
Mann-Whitney U	170,000
Wilcoxon W	180,000
Test Statistic	170,000
Standard Error	48,177
Standardized Test Statistic	,166
Asymptotic Sig.(2-sided test)	,868
Exact Sig.(2-sided test)	,881

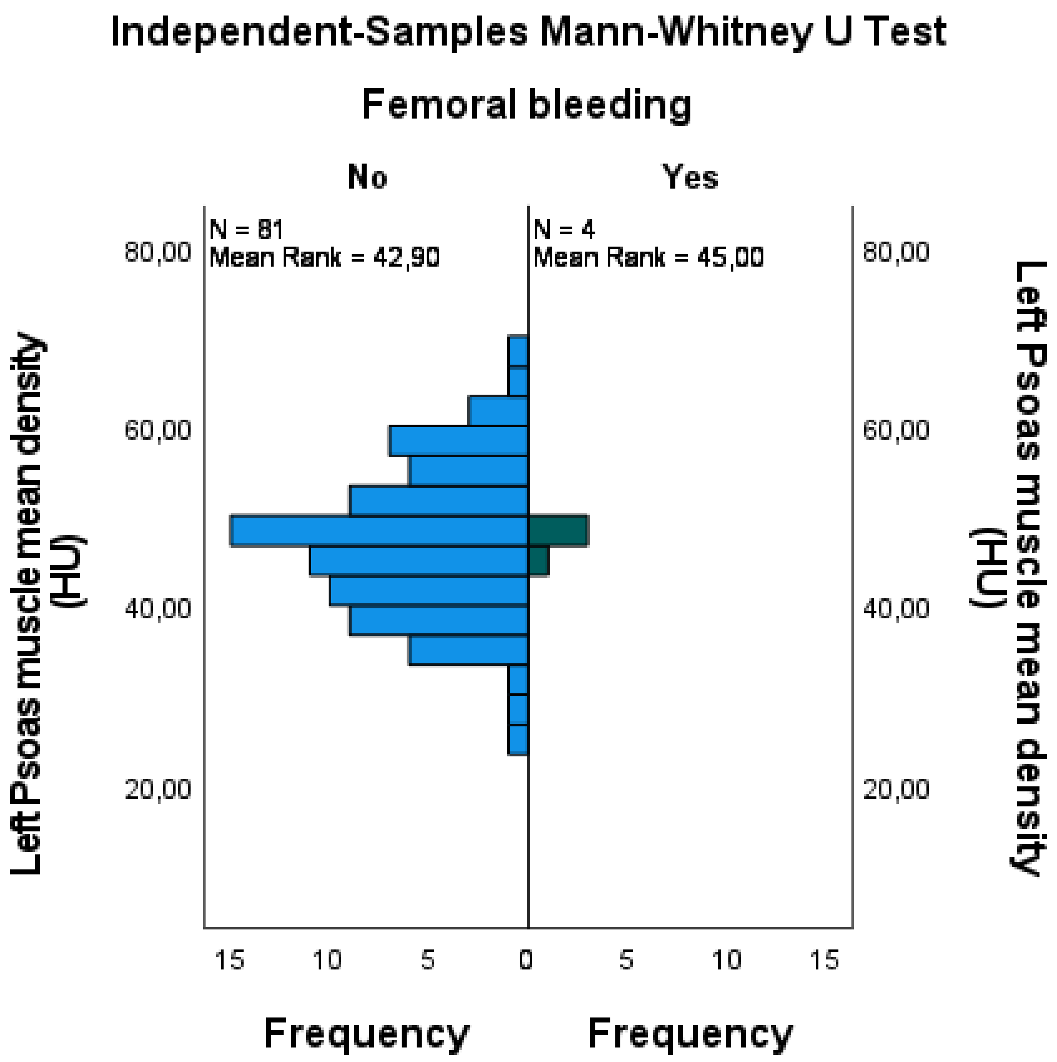

Left Psoas muscle median density (HU) across Femoral bleeding

Independent-Samples Mann-Whitney U Test Summary

Total N	85
Mann-Whitney U	160,000
Wilcoxon W	170,000
Test Statistic	160,000
Standard Error	48,126
Standardized Test Statistic	-,042
Asymptotic Sig.(2-sided test)	,967
Exact Sig.(2-sided test)	,976

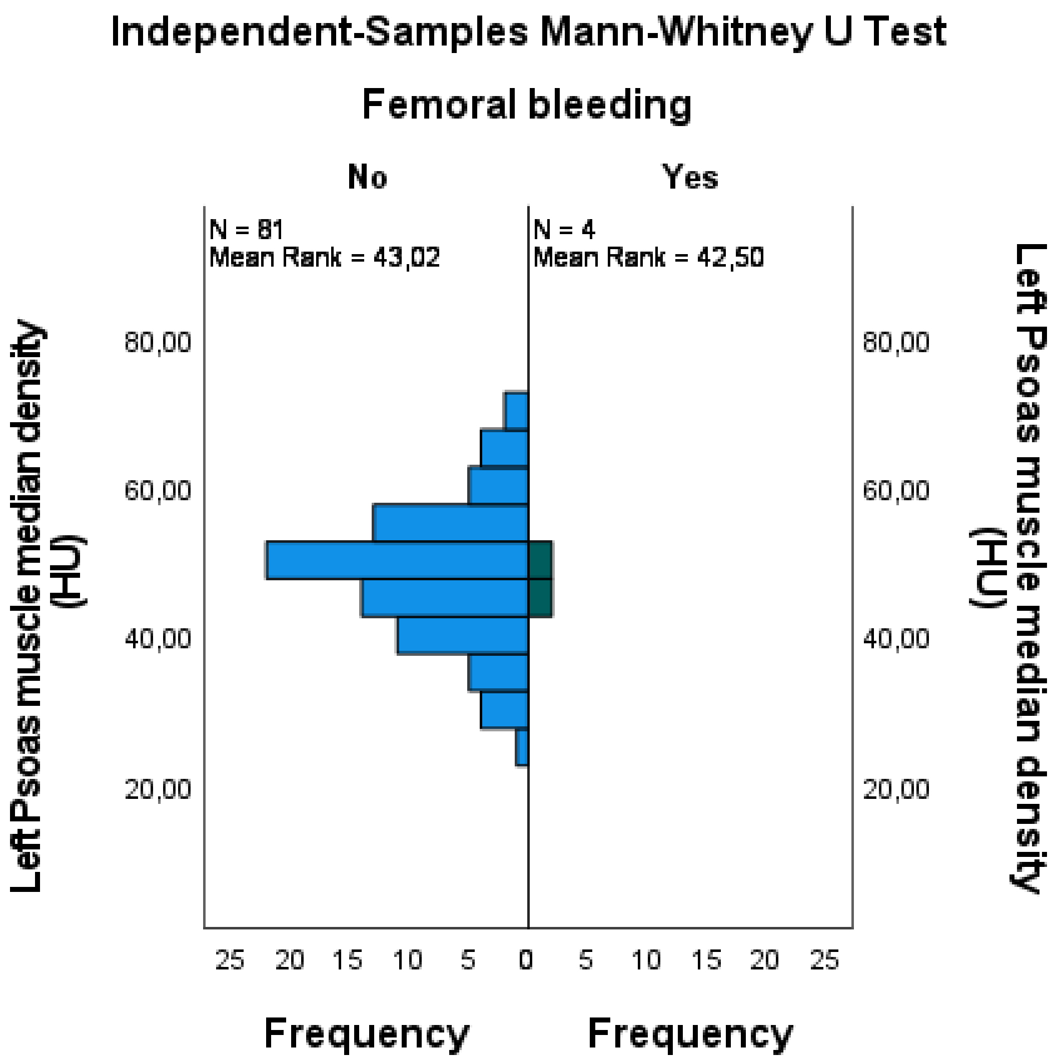

Left Psoas muscle density standard deviation across Femoral bleeding

Independent-Samples Mann-Whitney U Test Summary

Total N	85
Mann-Whitney U	187,000
Wilcoxon W	197,000
Test Statistic	187,000
Standard Error	48,187
Standardized Test Statistic	,519
Asymptotic Sig.(2-sided test)	,604
Exact Sig.(2-sided test)	,624

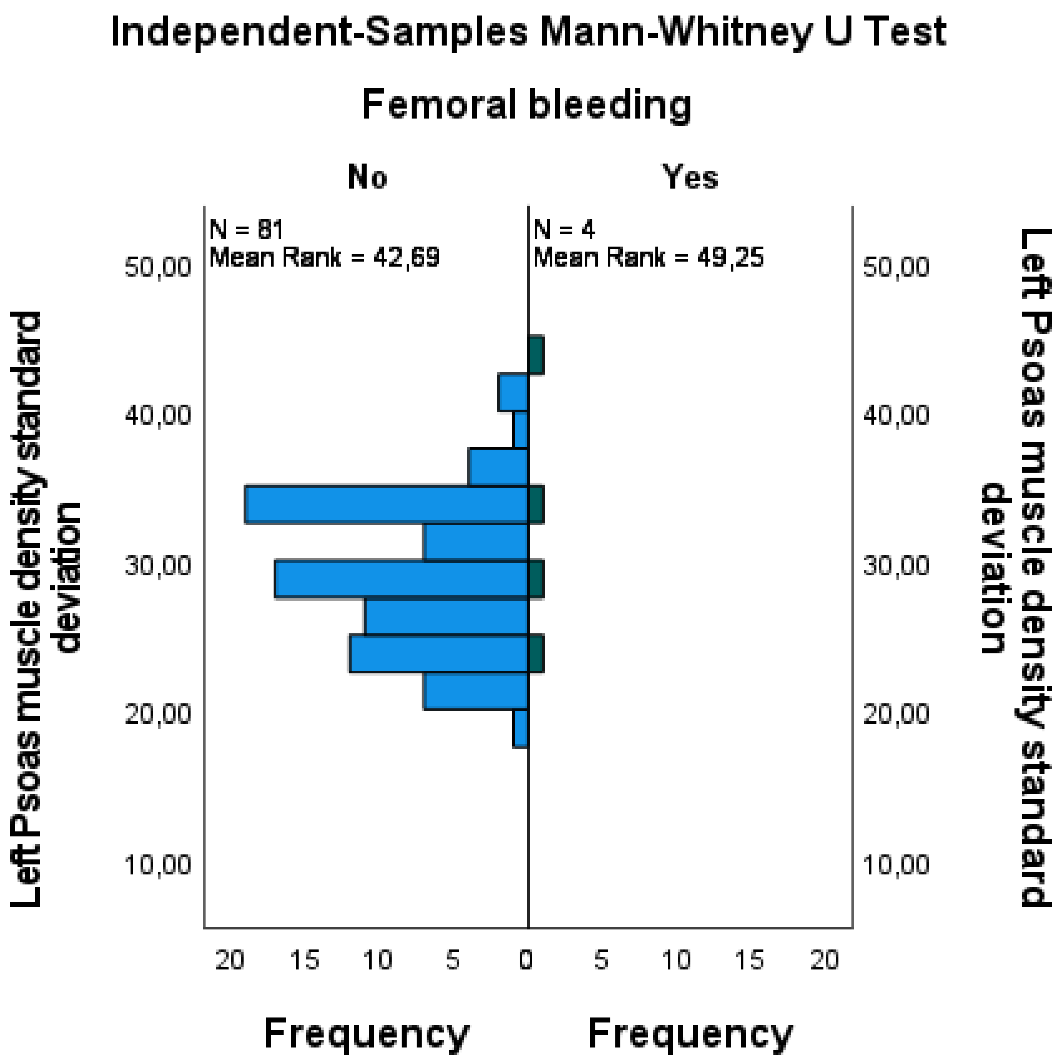

Independent-Samples Mann-Whitney U Test for blood transfusion

Hypothesis Test Summary

	Null Hypothesis	Test	Sig.[a,b]	Decision
1	The distribution of Psoas/height is the same across categories of Blood transfusion.	Independent-Samples Mann-Whitney U Test	,114	Retain the null hypothesis.
2	The distribution of Anterior SAT distance is the same across categories of Blood transfusion.	Independent-Samples Mann-Whitney U Test	<,001	Reject the null hypothesis.
3	The distribution of Posterior SAT distance is the same across categories of Blood transfusion.	Independent-Samples Mann-Whitney U Test	<,001	Reject the null hypothesis.
4	The distribution of Anterior+Posterior SAT distance is the same across categories of Blood transfusion.	Independent-Samples Mann-Whitney U Test	<,001	Reject the null hypothesis.
5	The distribution of VAT distance is the same across categories of Blood transfusion.	Independent-Samples Mann-Whitney U Test	,004	Reject the null hypothesis.
6	The distribution of Right common femoral artery area (mm2) is the same across categories of Blood transfusion.	Independent-Samples Mann-Whitney U Test	,118	Retain the null hypothesis.
7	The distribution of Left common femoral artery area (mm2) is the same across categories of Blood transfusion.	Independent-Samples Mann-Whitney U Test	,399	Retain the null hypothesis.
8	The distribution of FAT area (cm2) is the same across categories of Blood transfusion.	Independent-Samples Mann-Whitney U Test	<,001	Reject the null hypothesis.
9	The distribution of SAT area (cm2) is the same across categories of Blood transfusion.	Independent-Samples Mann-Whitney U Test	<,001	Reject the null hypothesis.
10	The distribution of VAT area (cm2) is the same across categories of Blood transfusion.	Independent-Samples Mann-Whitney U Test	<,001	Reject the null hypothesis.
11	The distribution of Right Psoas muscle area (cm2) is the same across categories of Blood transfusion.	Independent-Samples Mann-Whitney U Test	,195	Retain the null hypothesis.

12	The distribution of Left Psoas muscle area (cm2) is the same across categories of Blood transfusion.	Independent-Samples Mann-Whitney U Test	,071	Retain the null hypothesis.
13	The distribution of FAT mean density (HU) is the same across categories of Blood transfusion.	Independent-Samples Mann-Whitney U Test	,696	Retain the null hypothesis.
14	The distribution of FAT median density (HU) is the same across categories of Blood transfusion.	Independent-Samples Mann-Whitney U Test	,738	Retain the null hypothesis.
15	The distribution of FAT density standard deviation is the same across categories of Blood transfusion.	Independent-Samples Mann-Whitney U Test	,179	Retain the null hypothesis.
16	The distribution of SAT mean density (HU) is the same across categories of Blood transfusion.	Independent-Samples Mann-Whitney U Test	,717	Retain the null hypothesis.
17	The distribution of SAT median density (HU) is the same across categories of Blood transfusion.	Independent-Samples Mann-Whitney U Test	,687	Retain the null hypothesis.
18	The distribution of SAT density standard deviation is the same across categories of Blood transfusion.	Independent-Samples Mann-Whitney U Test	,492	Retain the null hypothesis.
19	The distribution of VAT mean density (HU) is the same across categories of Blood transfusion.	Independent-Samples Mann-Whitney U Test	,958	Retain the null hypothesis.
20	The distribution of VAT median density (HU) is the same across categories of Blood transfusion.	Independent-Samples Mann-Whitney U Test	,886	Retain the null hypothesis.
21	The distribution of VAT density standard deviation is the same across categories of Blood transfusion.	Independent-Samples Mann-Whitney U Test	,043	Reject the null hypothesis.
22	The distribution of Right Psoas muscle mean density (HU) is the same across categories of Blood transfusion.	Independent-Samples Mann-Whitney U Test	,689	Retain the null hypothesis.
23	The distribution of Right Psoas muscle median density (HU) is the same across categories of Blood transfusion.	Independent-Samples Mann-Whitney U Test	,314	Retain the null hypothesis.

24	The distribution of Right Psoas muscle density standard deviation is the same across categories of Blood transfusion.	Independent-Samples Mann-Whitney U Test	,023	Reject the null hypothesis.
25	The distribution of Left Psoas muscle mean density (HU) is the same across categories of Blood transfusion.	Independent-Samples Mann-Whitney U Test	,757	Retain the null hypothesis.
26	The distribution of Left Psoas muscle median density (HU) is the same across categories of Blood transfusion.	Independent-Samples Mann-Whitney U Test	,966	Retain the null hypothesis.
27	The distribution of Left Psoas muscle density standard deviation is the same across categories of Blood transfusion.	Independent-Samples Mann-Whitney U Test	,105	Retain the null hypothesis.

a. The significance level is ,050.

b. Asymptotic significance is displayed.

In this case, the hypothesis of equal medians ($p < 0.05$) is rejected for the variables Anterior SAT distance, Posterior SAT distance, Anterior + Posterior SAT distance, VAT distance, FAT area (cm2), SAT area (cm2), VAT area (cm2),

VAT density standard deviation and Right Psoas muscle density standard deviation, while for the rest we accept the null hypothesis ($p > 0.05$).

(The tables and graphs below are the details of the tests in this table: I have highlighted what things you should eventually report, namely test statistic and pvalue).

Psoas/height across Blood transfusion

Independent-Samples Mann-Whitney U Test Summary

Total N	85
Mann-Whitney U	601,500
Wilcoxon W	952,500
Test Statistic	601,500
Standard Error	104,850
Standardized Test Statistic	-1,578
Asymptotic Sig.(2-sided test)	,114

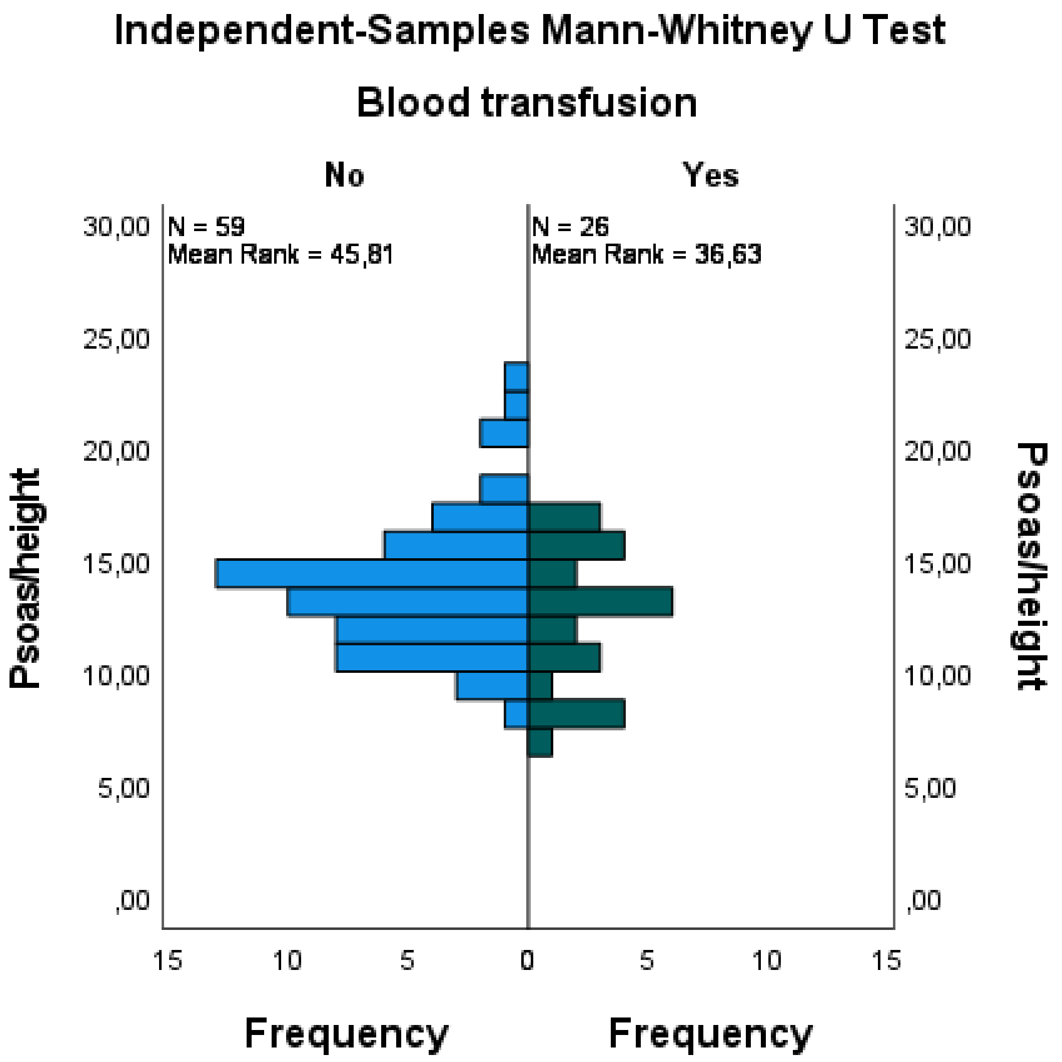

Anterior SAT distance across Blood transfusion

Independent-Samples Mann-Whitney U Test Summary

Total N	85
Mann-Whitney U	312,000
Wilcoxon W	663,000
Test Statistic	312,000
Standard Error	104,836
Standardized Test Statistic	-4,340
Asymptotic Sig.(2-sided test)	<,001

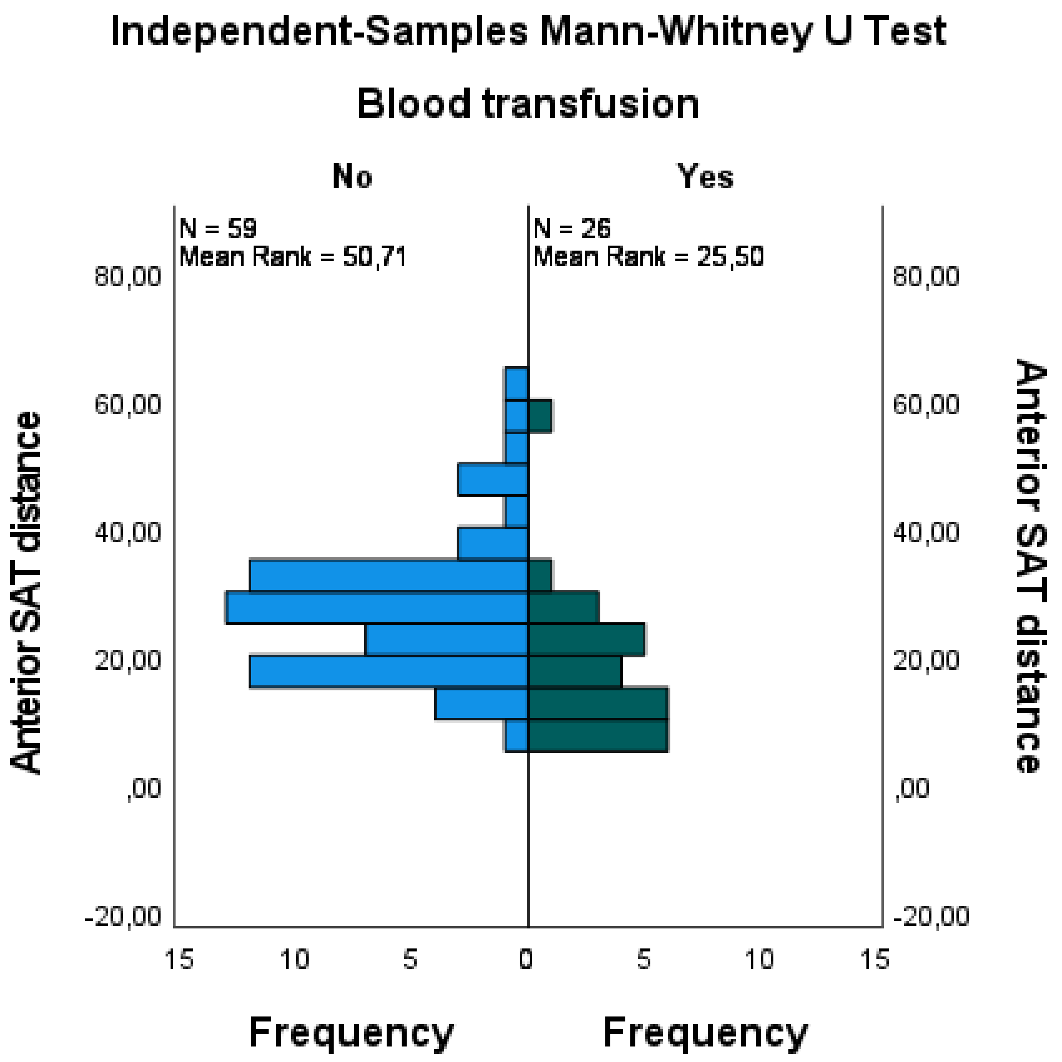

Posterior SAT distance across Blood transfusion

Independent-Samples Mann-Whitney U Test Summary

Total N	85
Mann-Whitney U	196,500
Wilcoxon W	547,500
Test Statistic	196,500
Standard Error	104,839
Standardized Test Statistic	-5,442
Asymptotic Sig.(2-sided test)	<,001

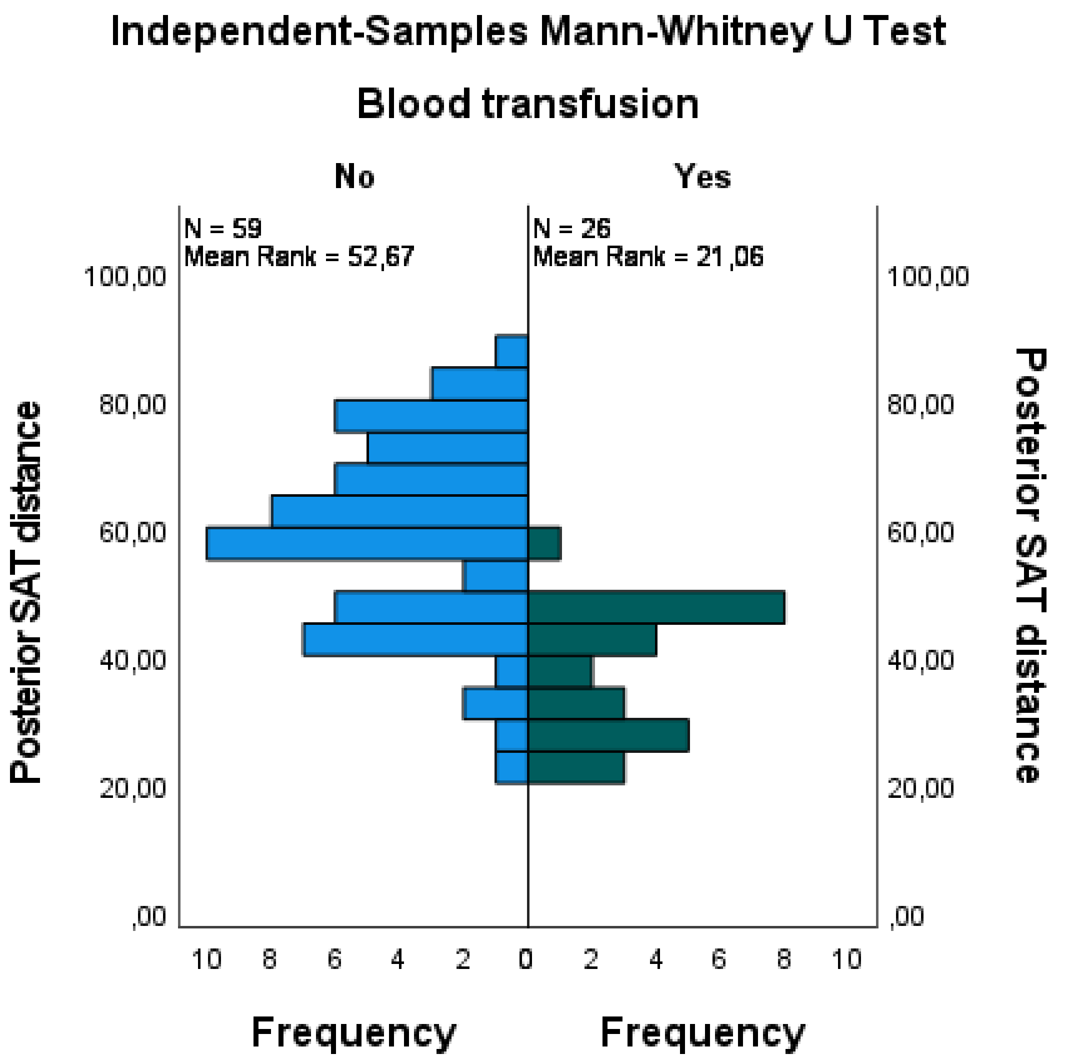

Anterior+Posterior SAT distance across Blood transfusion

Independent-Samples Mann-Whitney U Test Summary

Total N	85
Mann-Whitney U	194,000
Wilcoxon W	545,000
Test Statistic	194,000
Standard Error	104,846
Standardized Test Statistic	-5,465
Asymptotic Sig.(2-sided test)	<,001

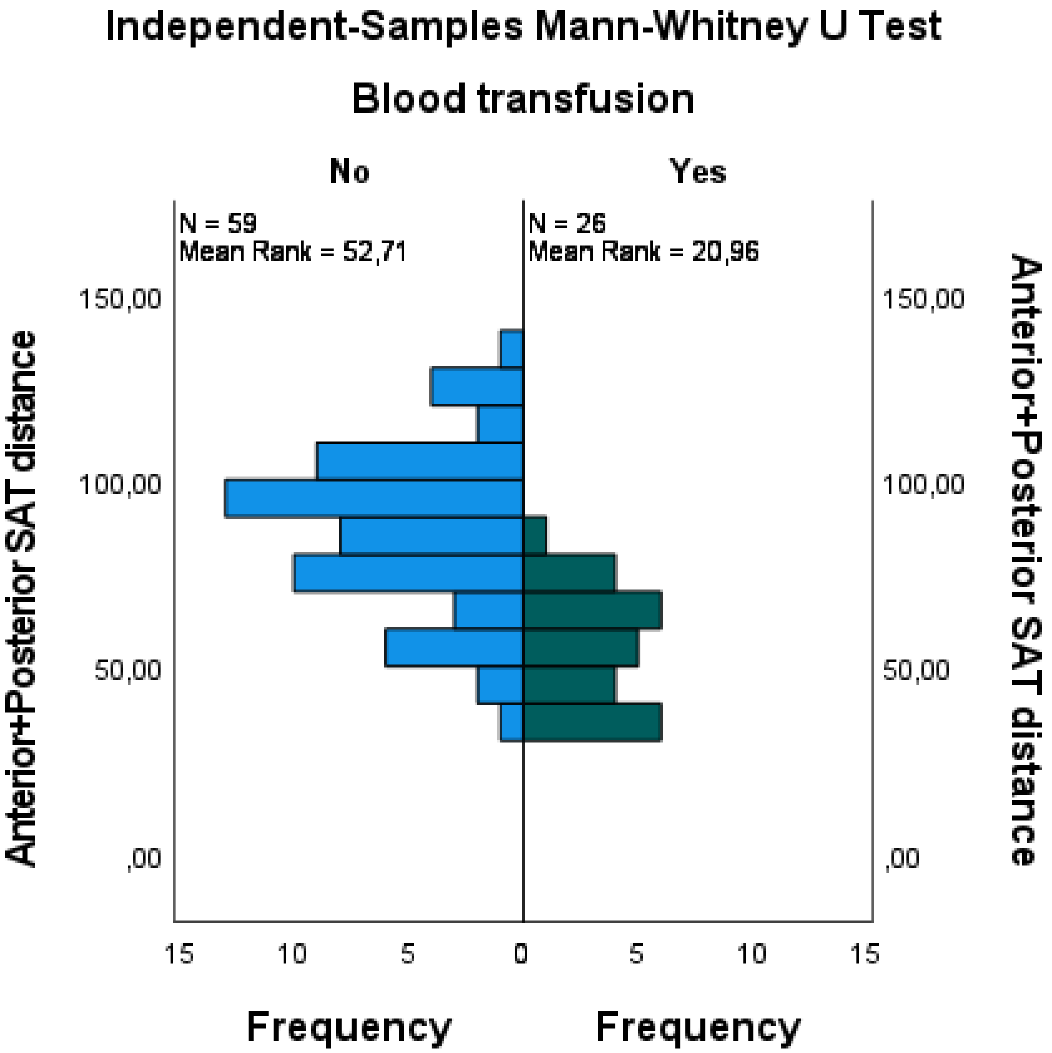

VAT distance across Blood transfusion

Independent-Samples Mann-Whitney U Test Summary

Total N	84
Mann-Whitney U	457,500
Wilcoxon W	808,500
Test Statistic	457,500
Standard Error	103,331
Standardized Test Statistic	-2,869
Asymptotic Sig.(2-sided test)	,004

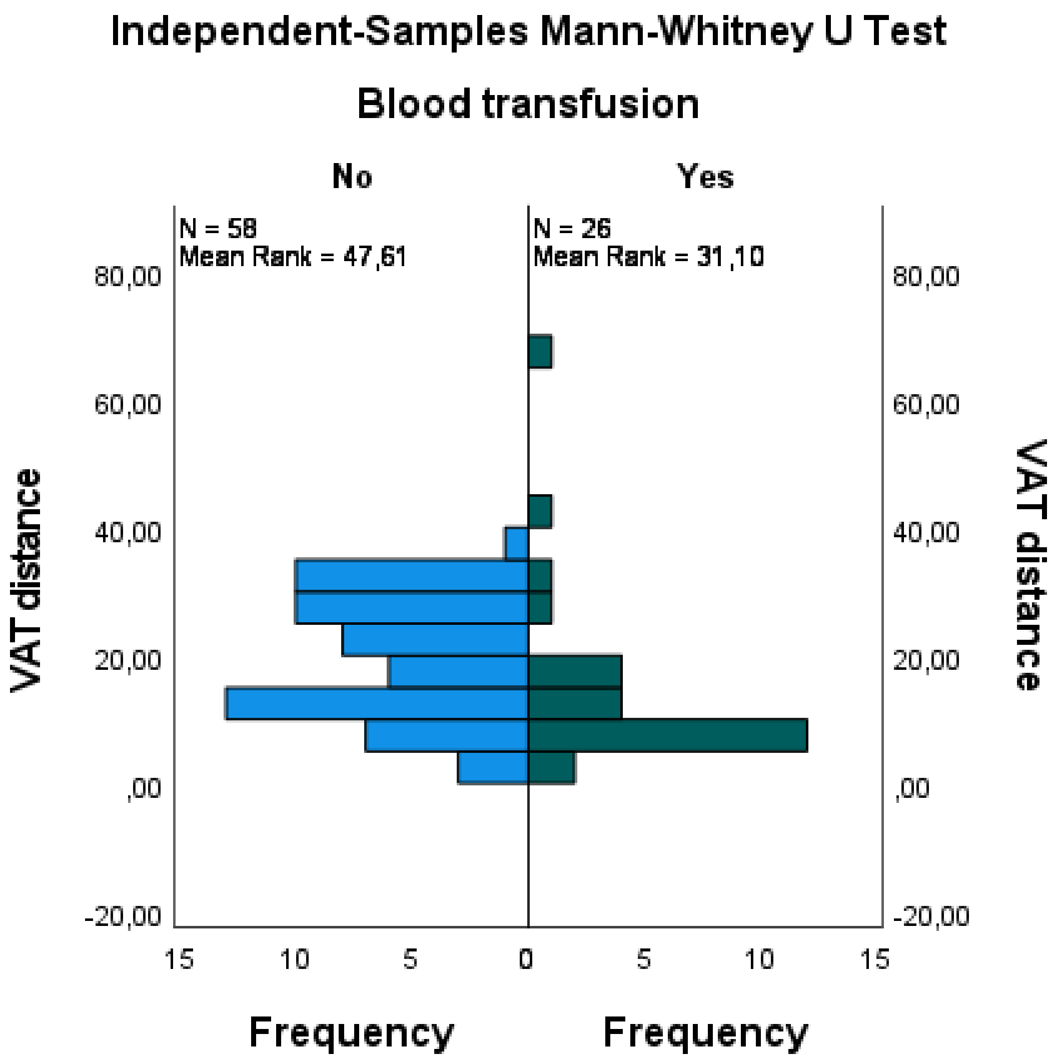

Right common femoral artery area (mm2) across Blood transfusion

Independent-Samples Mann-Whitney U Test Summary

Total N	85
Mann-Whitney U	931,000
Wilcoxon W	1282,000
Test Statistic	931,000
Standard Error	104,833
Standardized Test Statistic	1,564
Asymptotic Sig.(2-sided test)	,118

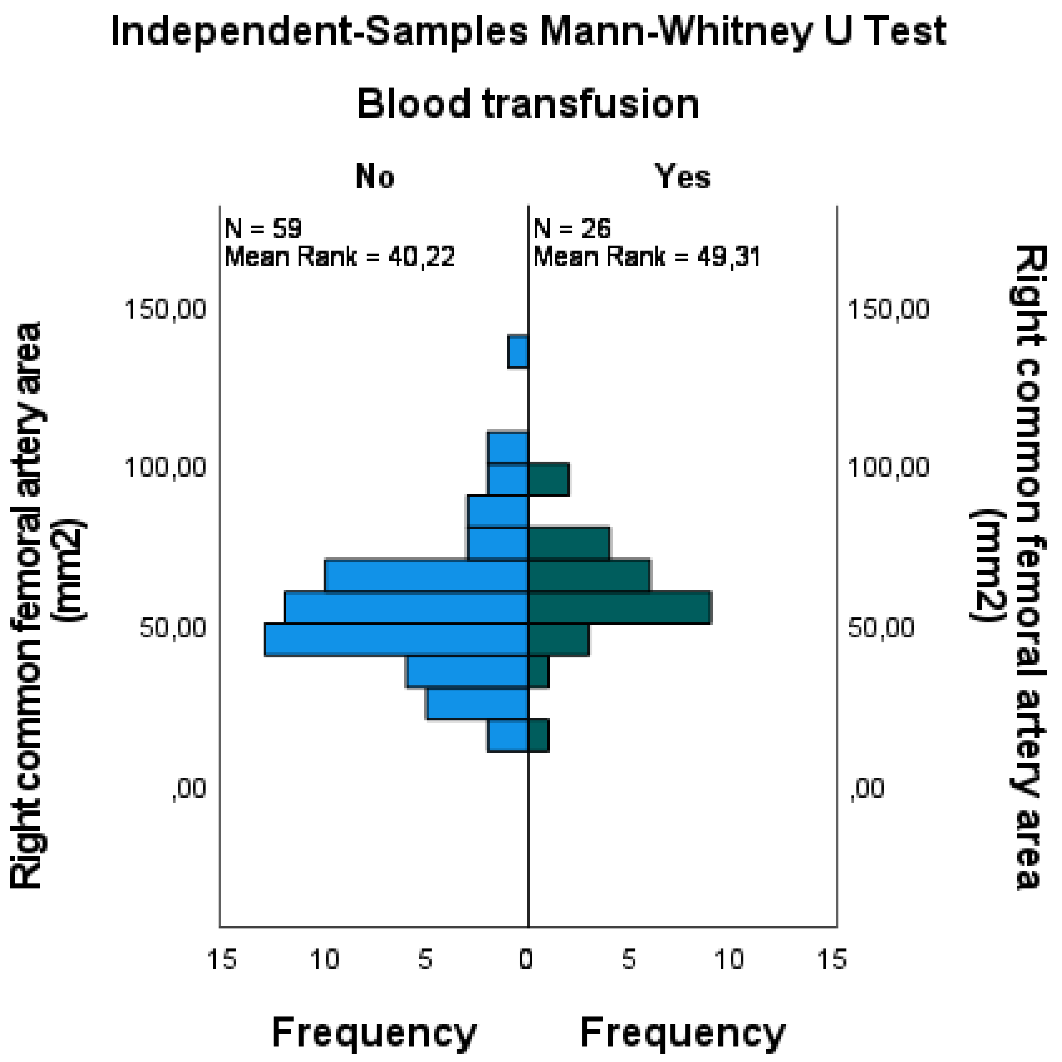

Left common femoral artery area (mm2) across Blood transfusion

Independent-Samples Mann-Whitney U Test Summary

Total N	85
Mann-Whitney U	855,500
Wilcoxon W	1206,500
Test Statistic	855,500
Standard Error	104,825
Standardized Test Statistic	,844
Asymptotic Sig.(2-sided test)	,399

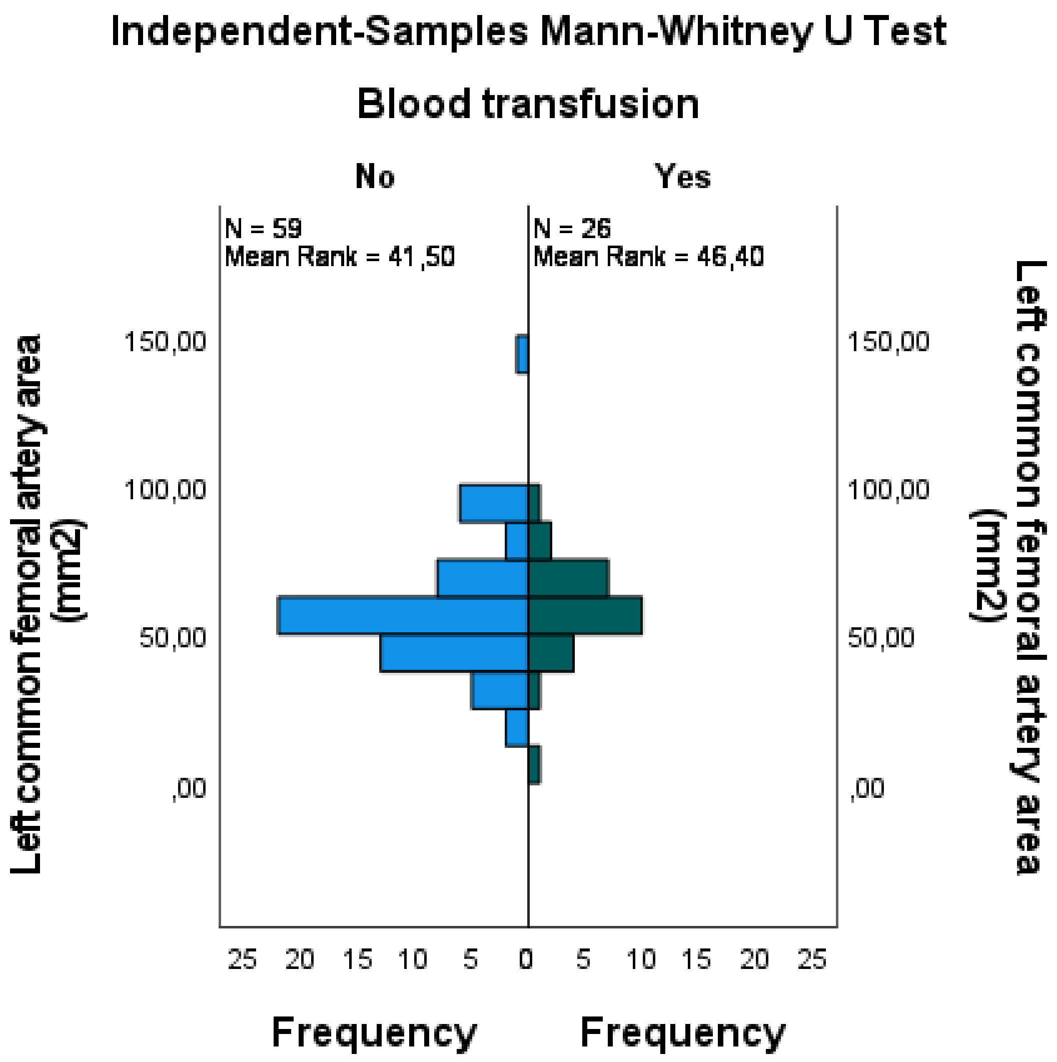

FAT area (cm2) across Blood transfusion

Independent-Samples Mann-Whitney U Test Summary

Total N	85
Mann-Whitney U	369,000
Wilcoxon W	720,000
Test Statistic	369,000
Standard Error	104,850
Standardized Test Statistic	-3,796
Asymptotic Sig.(2-sided test)	<,001

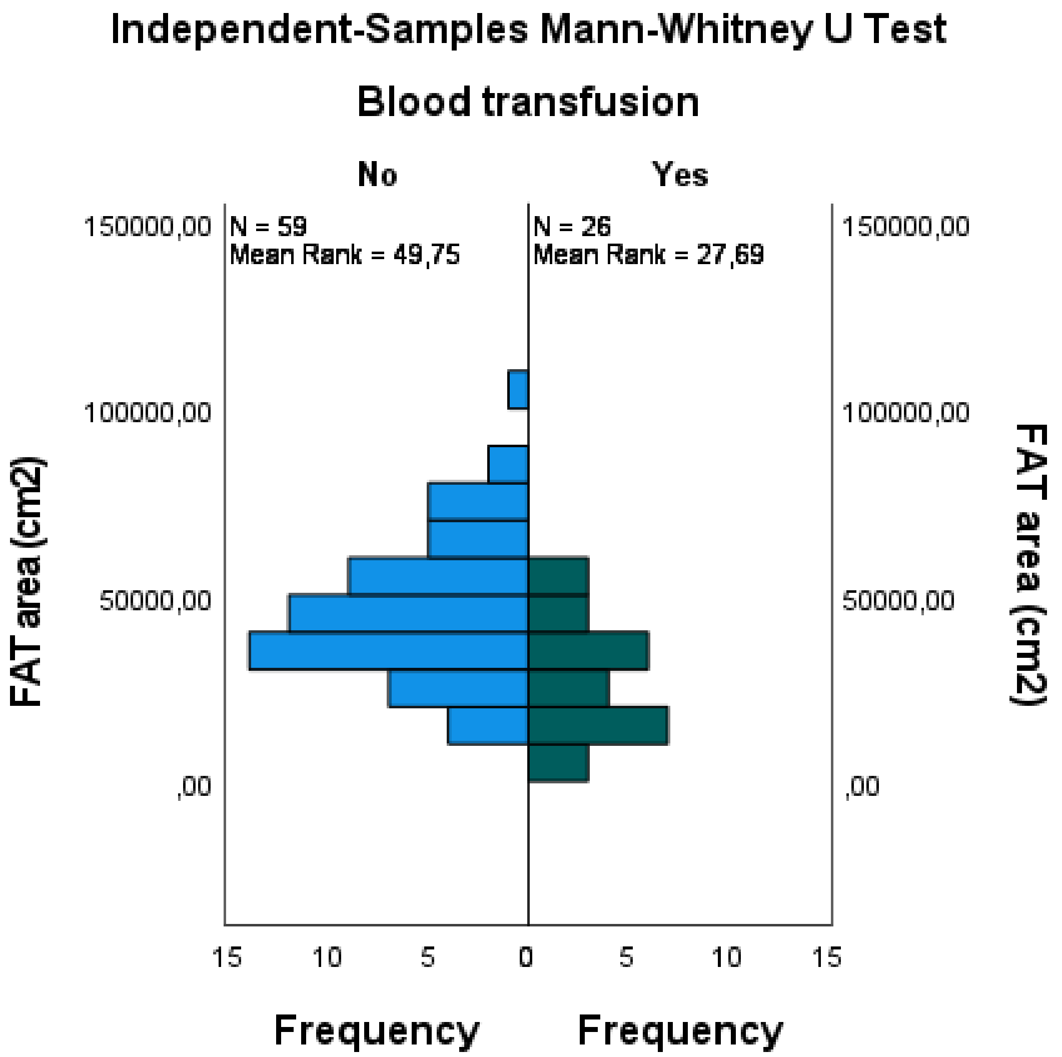

SAT area (cm2) across Blood transfusion

Independent-Samples Mann-Whitney U Test Summary

Total N	85
Mann-Whitney U	384,000
Wilcoxon W	735,000
Test Statistic	384,000
Standard Error	104,850
Standardized Test Statistic	-3,653
Asymptotic Sig.(2-sided test)	<,001

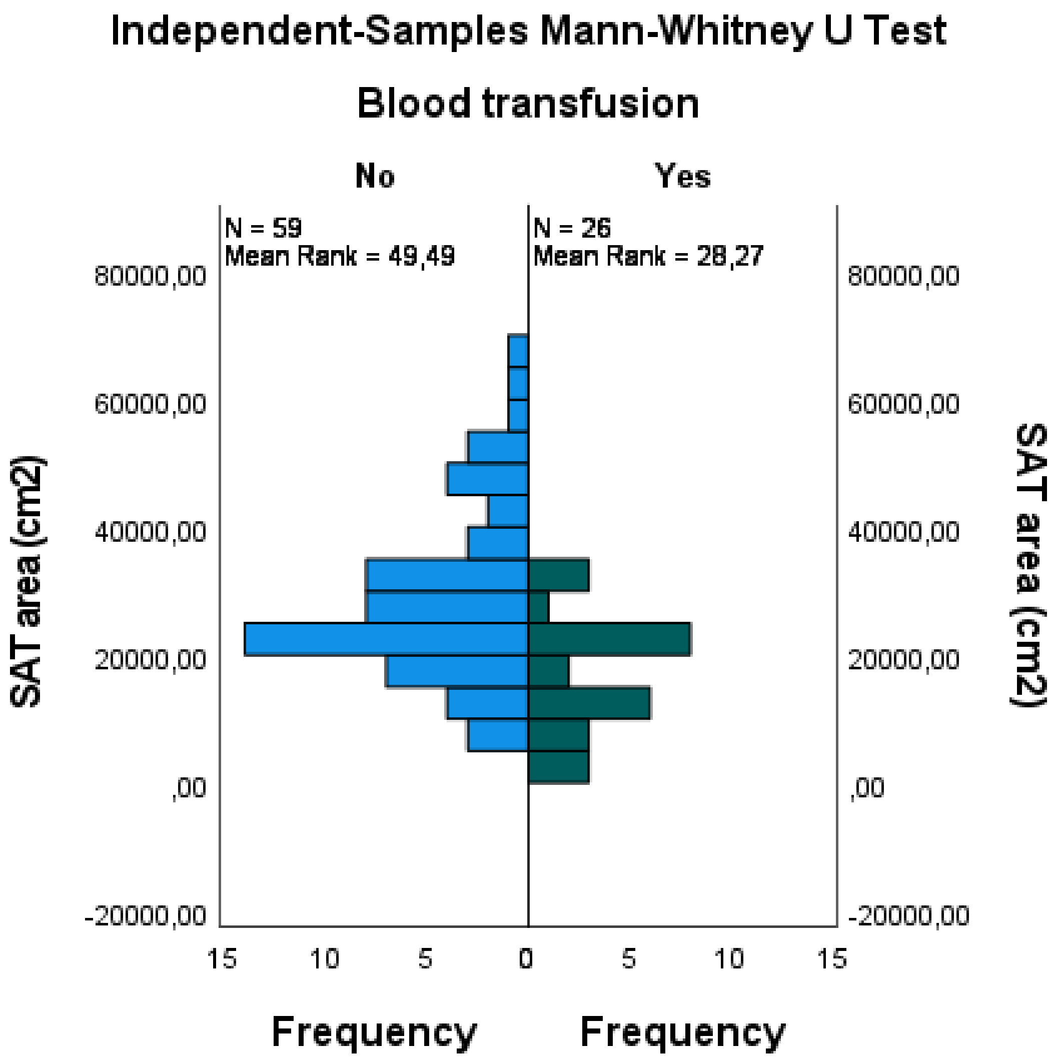

VAT area (cm2) across Blood transfusion

Independent-Samples Mann-Whitney U Test Summary

Total N	84
Mann-Whitney U	393,000
Wilcoxon W	744,000
Test Statistic	393,000
Standard Error	103,352
Standardized Test Statistic	-3,493
Asymptotic Sig.(2-sided test)	<,001

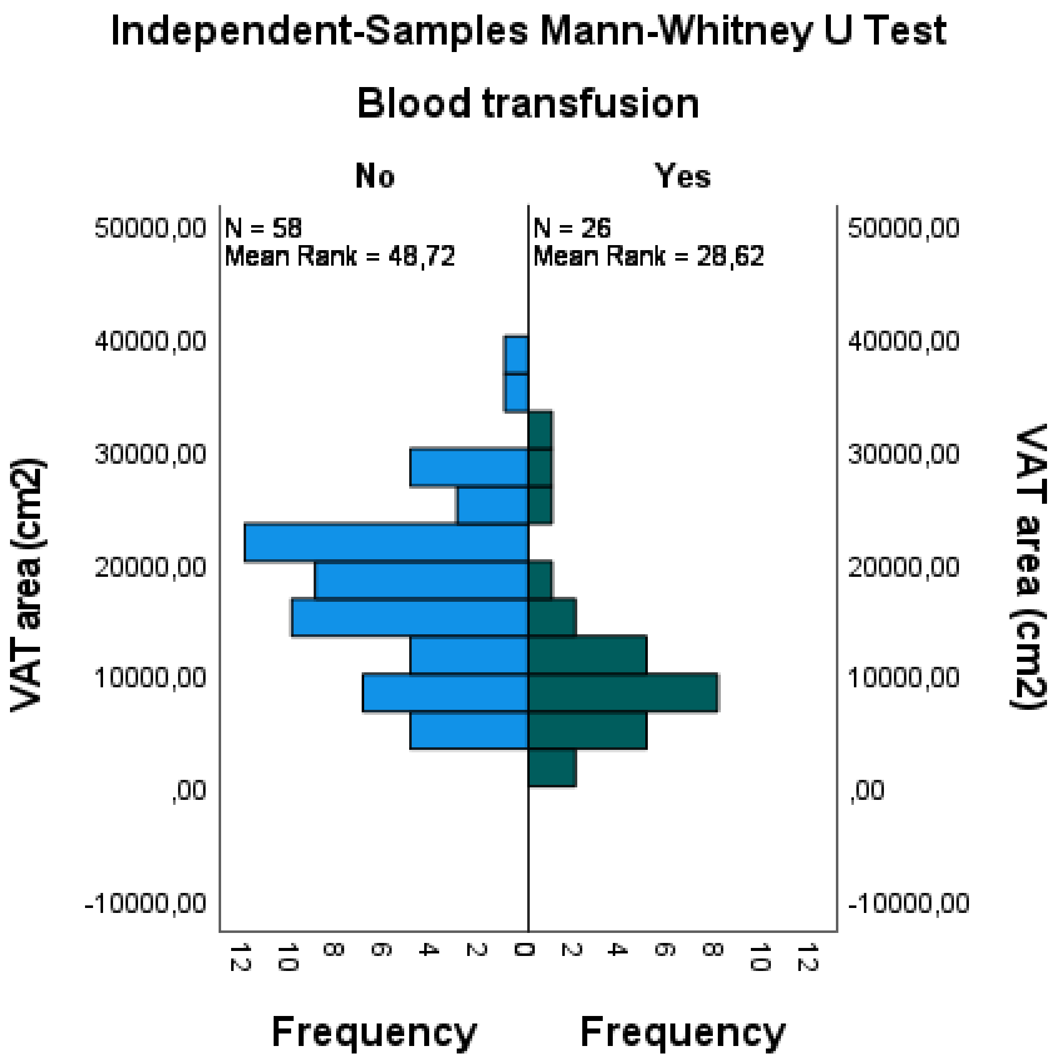

Right Psoas muscle area (cm2) across Blood transfusion

Independent-Samples Mann-Whitney U Test Summary

Total N	85
Mann-Whitney U	631,000
Wilcoxon W	982,000
Test Statistic	631,000
Standard Error	104,848
Standardized Test Statistic	-1,297
Asymptotic Sig.(2-sided test)	,195

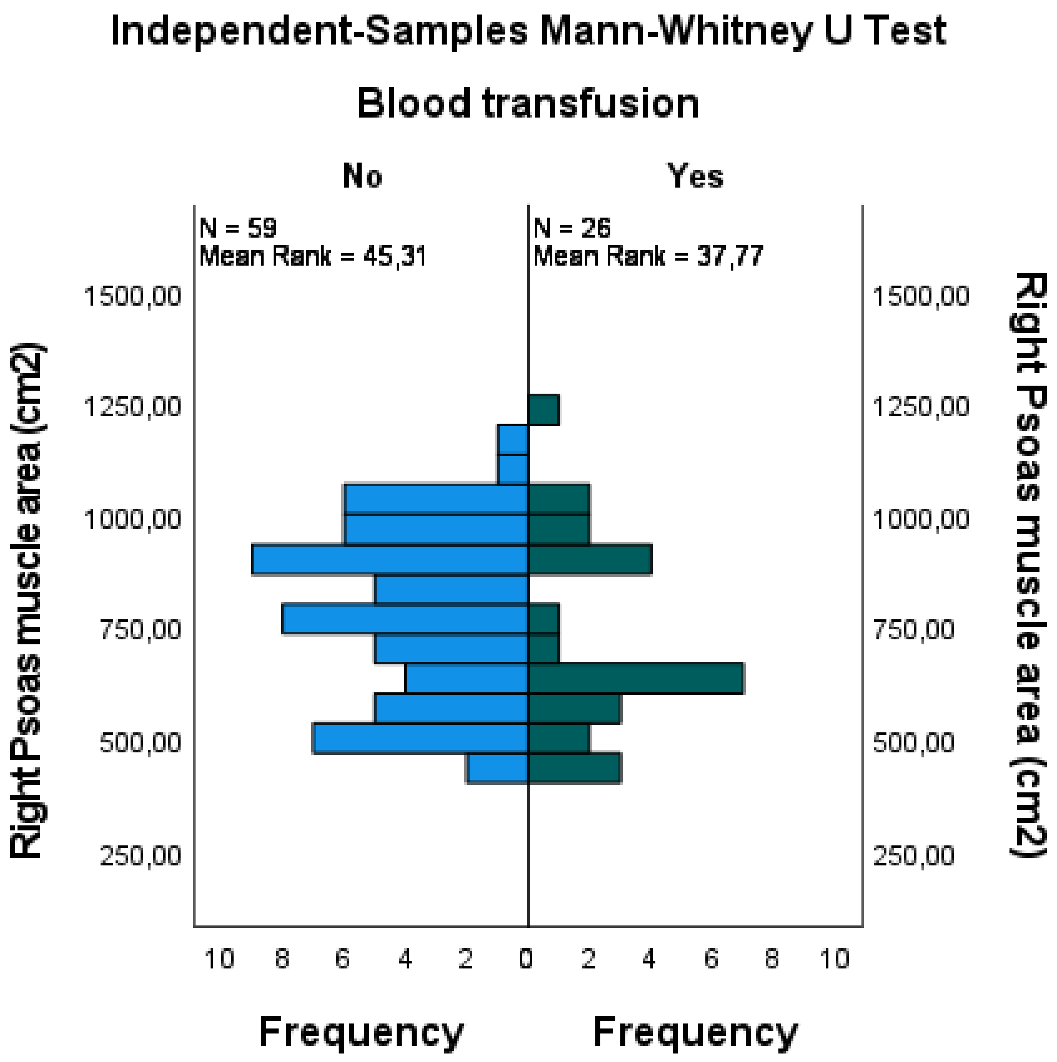

Left Psoas muscle area (cm2) across Blood transfusion

Independent-Samples Mann-Whitney U Test Summary

Total N	85
Mann-Whitney U	578,000
Wilcoxon W	929,000
Test Statistic	578,000
Standard Error	104,850
Standardized Test Statistic	-1,803
Asymptotic Sig.(2-sided test)	,071

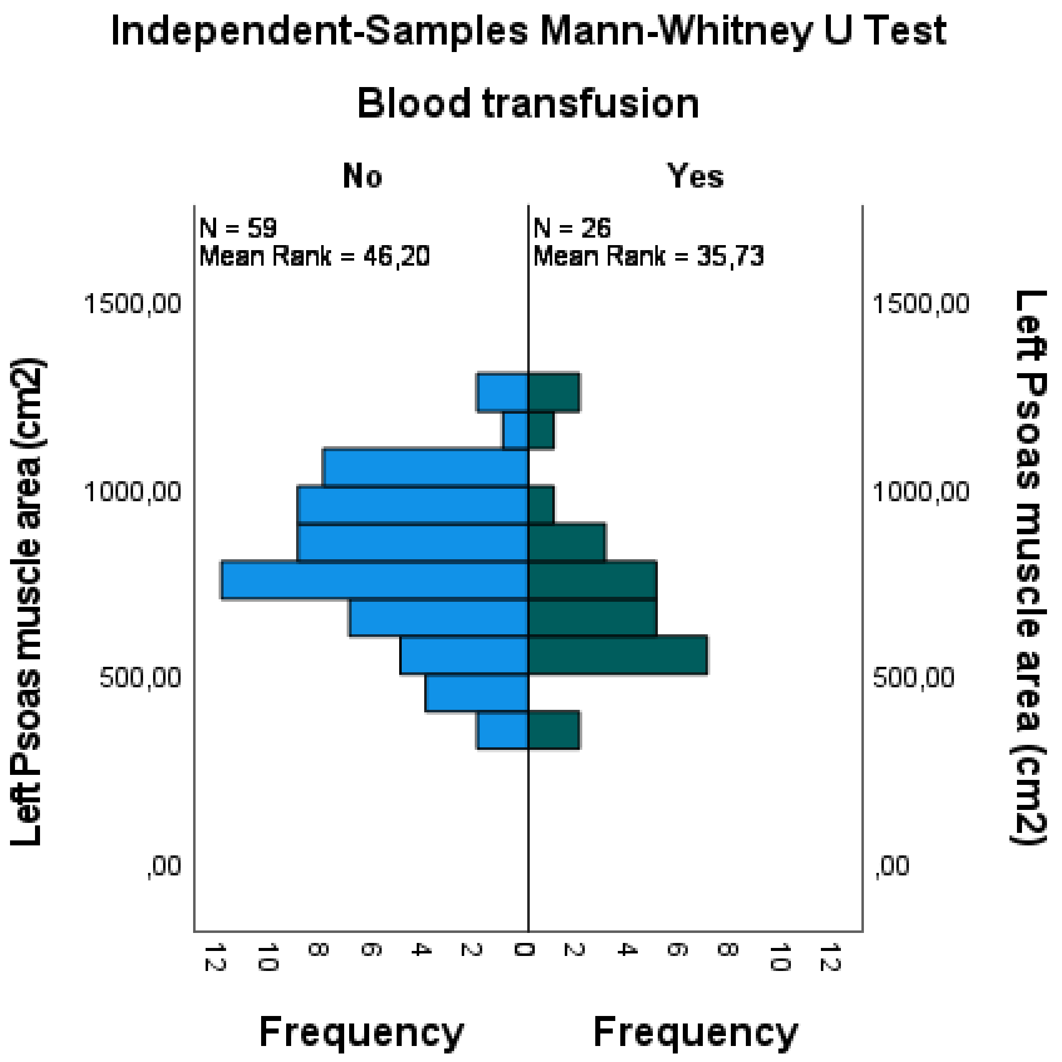

FAT mean density (HU) across Blood transfusion

Independent-Samples Mann-Whitney U Test Summary

Total N	85
Mann-Whitney U	808,000
Wilcoxon W	1159,000
Test Statistic	808,000
Standard Error	104,833
Standardized Test Statistic	,391
Asymptotic Sig.(2-sided test)	,696

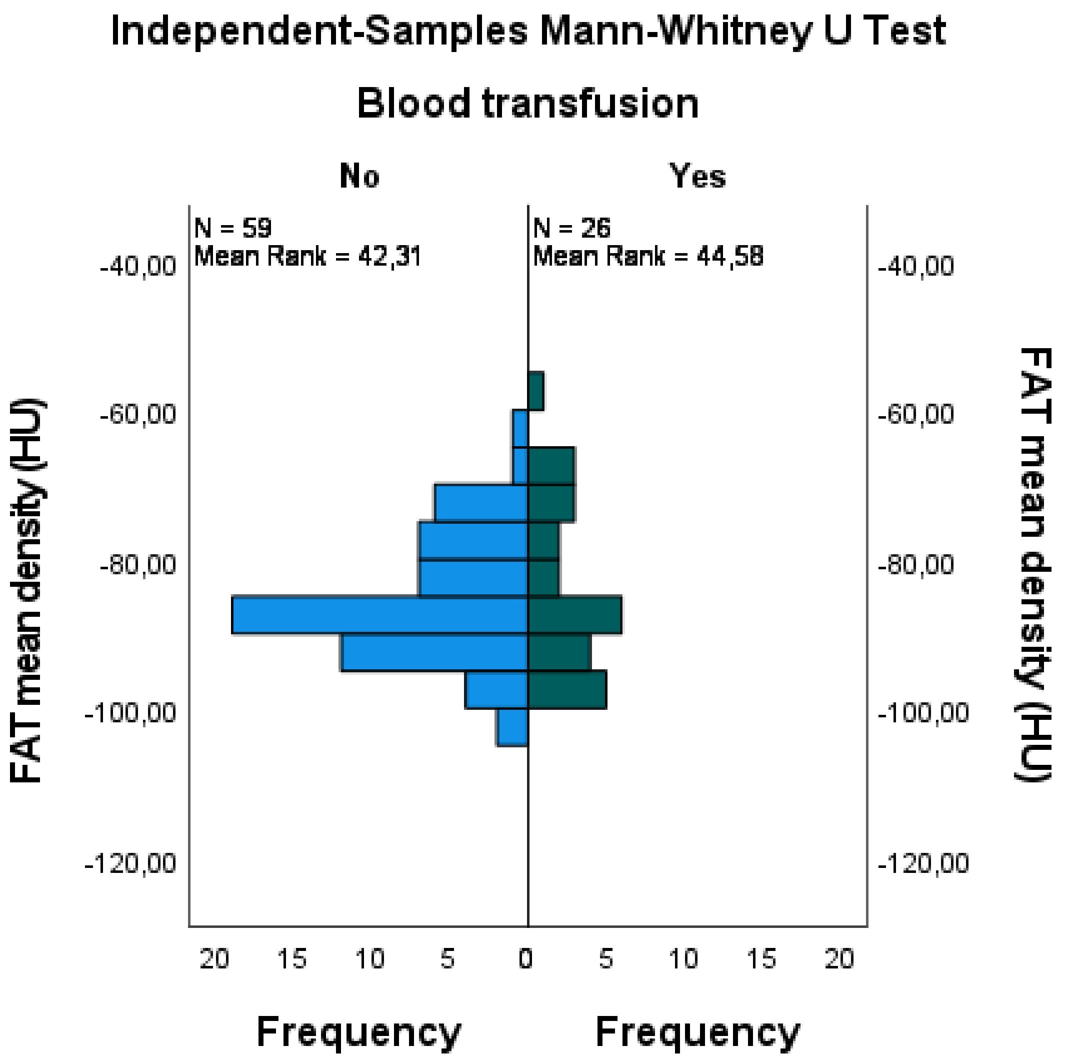

FAT median density (HU) across Blood transfusion

Independent-Samples Mann-Whitney U Test Summary

Total N	85
Mann-Whitney U	802,000
Wilcoxon W	1153,000
Test Statistic	802,000
Standard Error	104,724
Standardized Test Statistic	,334
Asymptotic Sig.(2-sided test)	,738

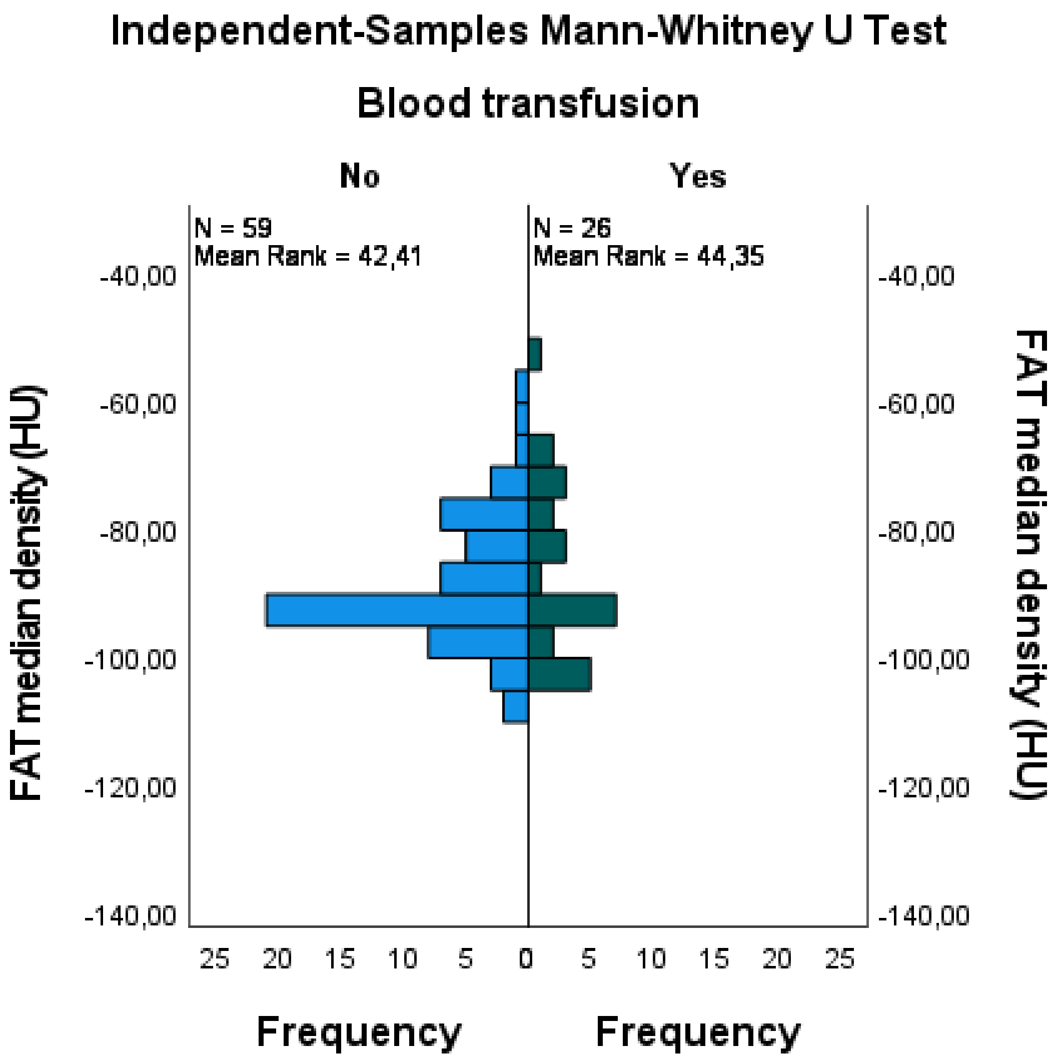

FAT density standard deviation across Blood transfusion

Independent-Samples Mann-Whitney U Test Summary

Total N	85
Mann-Whitney U	626,000
Wilcoxon W	977,000
Test Statistic	626,000
Standard Error	104,850
Standardized Test Statistic	-1,345
Asymptotic Sig.(2-sided test)	,179

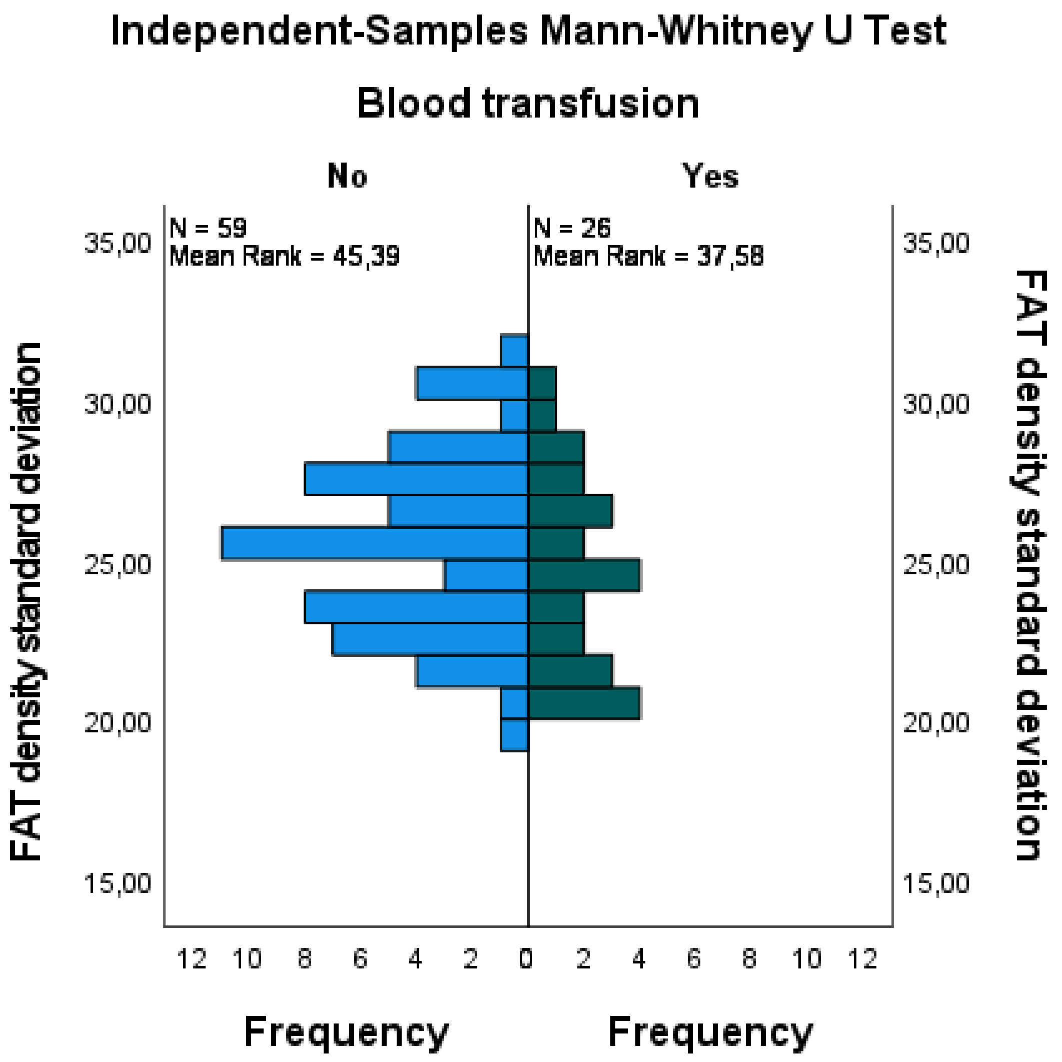

SAT mean density (HU) across Blood transfusion

Independent-Samples Mann-Whitney U Test Summary

Total N	85
Mann-Whitney U	805,000
Wilcoxon W	1156,000
Test Statistic	805,000
Standard Error	104,838
Standardized Test Statistic	,362
Asymptotic Sig.(2-sided test)	,717

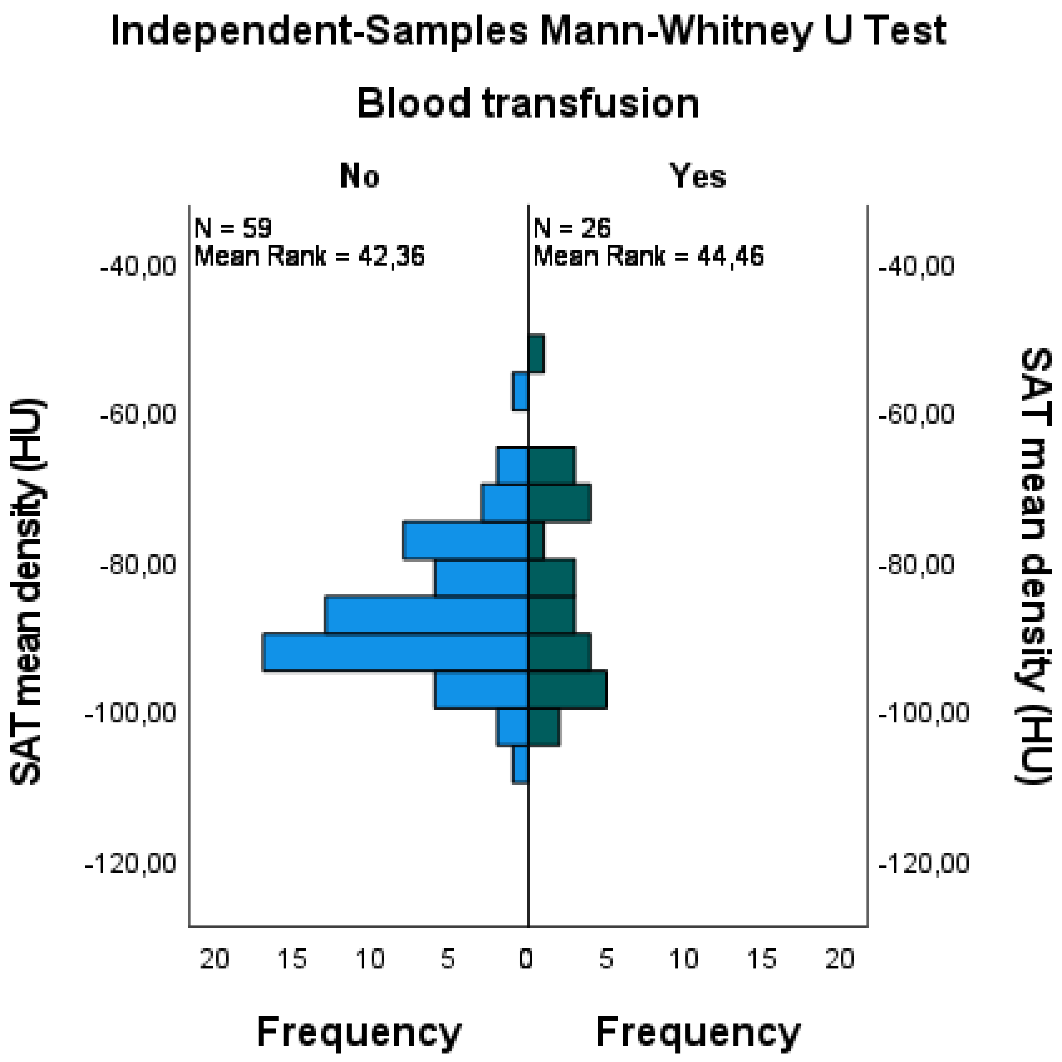

SAT median density (HU) across Blood transfusion

Independent-Samples Mann-Whitney U Test Summary

Total N	83
Mann-Whitney U	684,500
Wilcoxon W	1009,500
Test Statistic	684,500
Standard Error	100,669
Standardized Test Statistic	-,402
Asymptotic Sig.(2-sided test)	,687

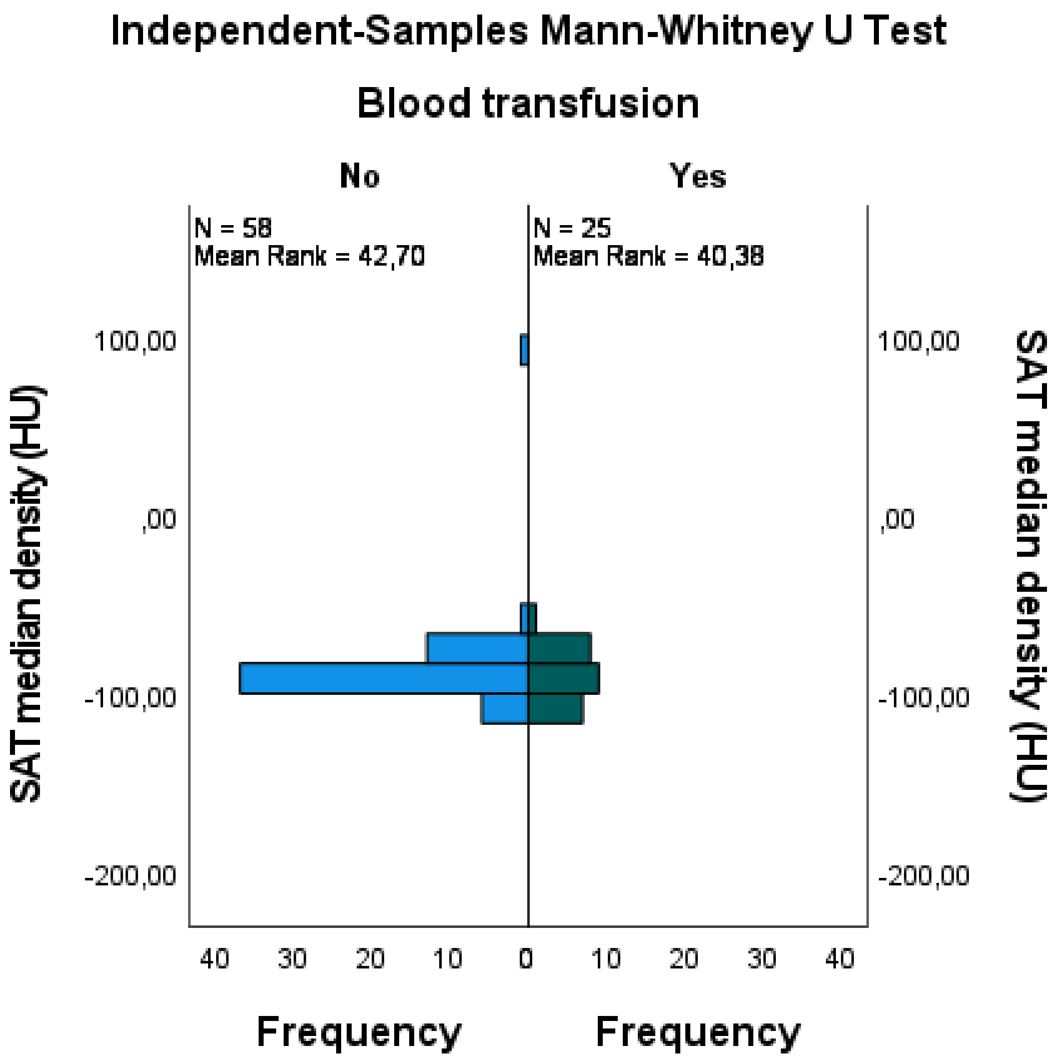

SAT density standard deviation across Blood transfusion

Independent-Samples Mann-Whitney U Test Summary

Total N	84
Mann-Whitney U	683,000
Wilcoxon W	1034,000
Test Statistic	683,000
Standard Error	103,352
Standardized Test Statistic	-,687
Asymptotic Sig.(2-sided test)	,492

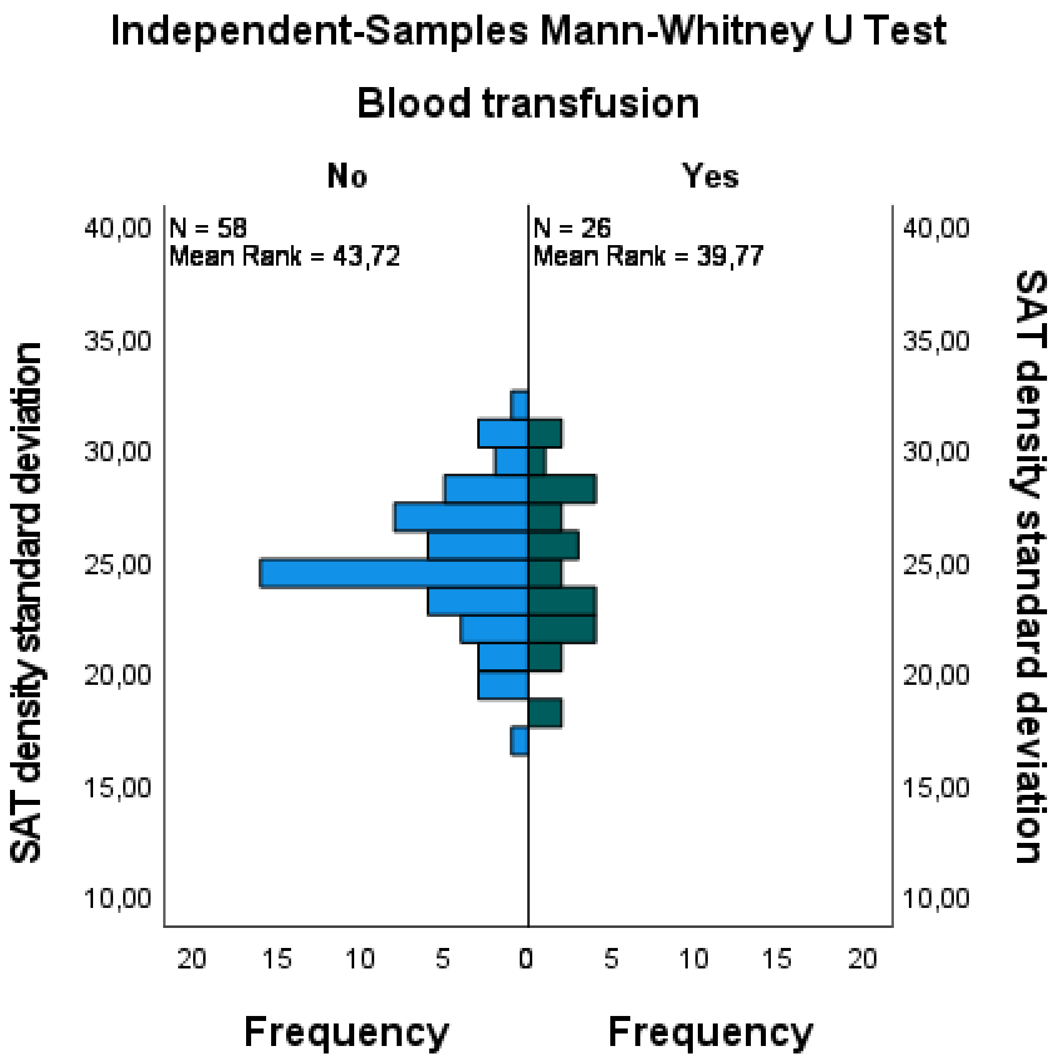

VAT mean density (HU) across Blood transfusion

Independent-Samples Mann-Whitney U Test Summary

Total N	85
Mann-Whitney U	772,500
Wilcoxon W	1123,500
Test Statistic	772,500
Standard Error	104,832
Standardized Test Statistic	,052
Asymptotic Sig.(2-sided test)	,958

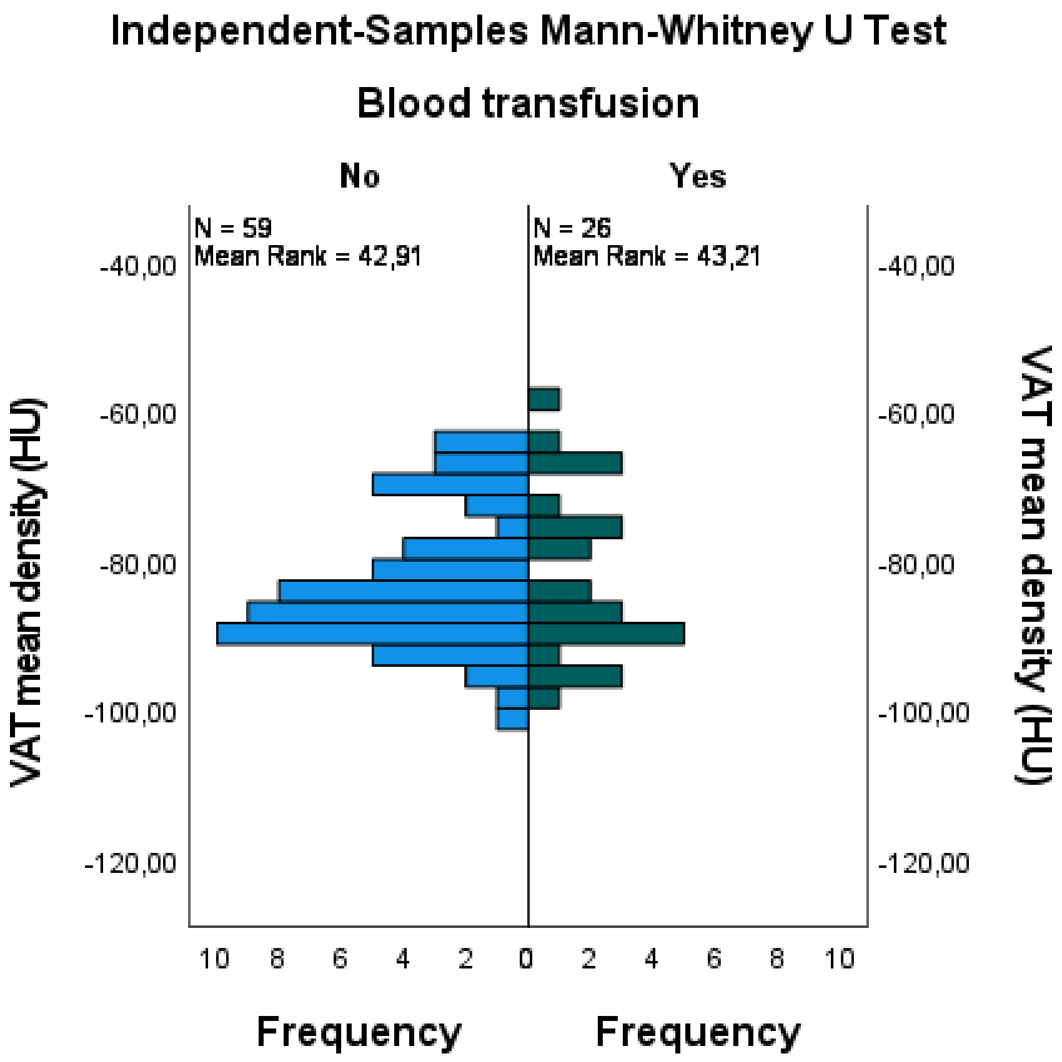

VAT median density (HU) across Blood transfusion

Independent-Samples Mann-Whitney U Test Summary

Total N	85
Mann-Whitney U	752,000
Wilcoxon W	1103,000
Test Statistic	752,000
Standard Error	104,719
Standardized Test Statistic	-,143
Asymptotic Sig.(2-sided test)	,886

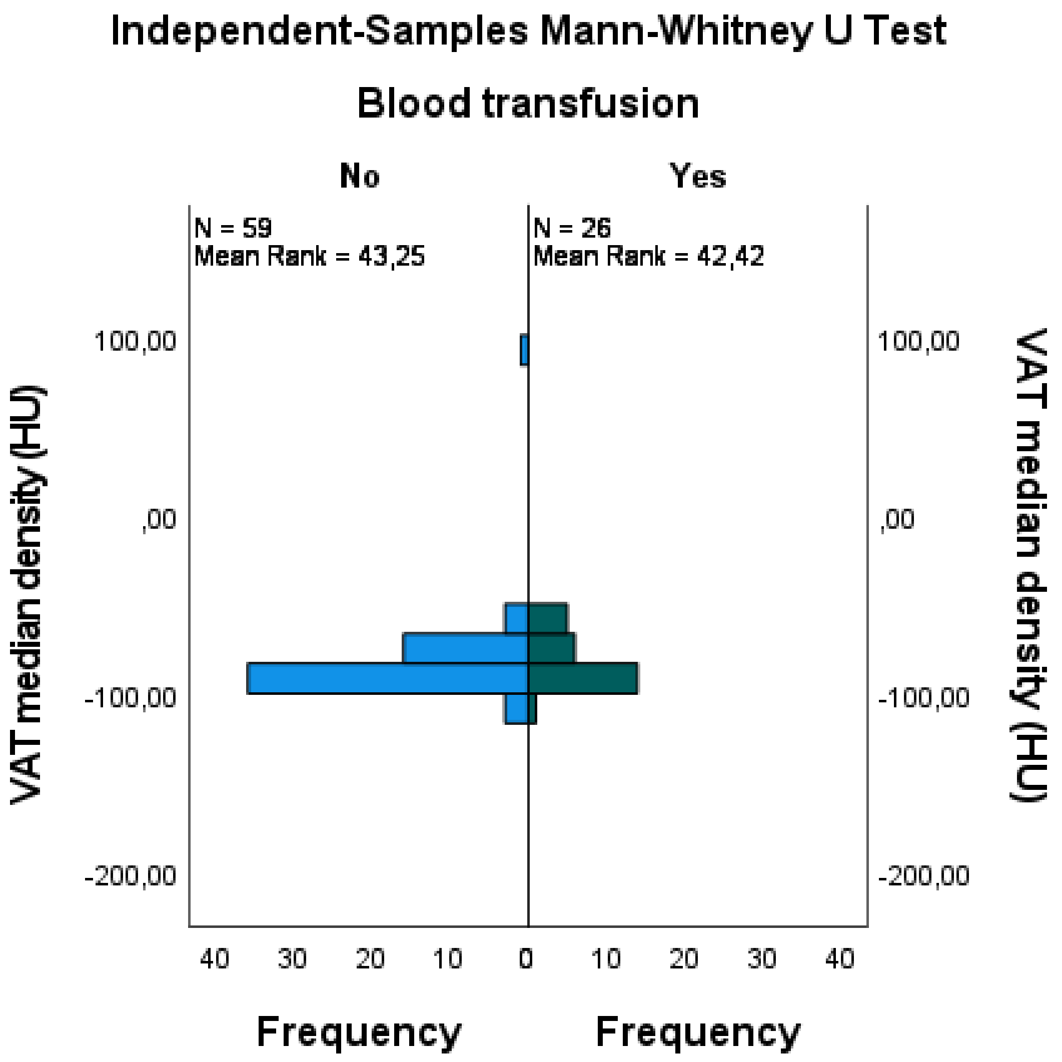

VAT density standard deviation across Blood transfusion

Independent-Samples Mann-Whitney U Test Summary

Total N	84
Mann-Whitney U	963,000
Wilcoxon W	1314,000
Test Statistic	963,000
Standard Error	103,352
Standardized Test Statistic	2,022
Asymptotic Sig.(2-sided test)	,043

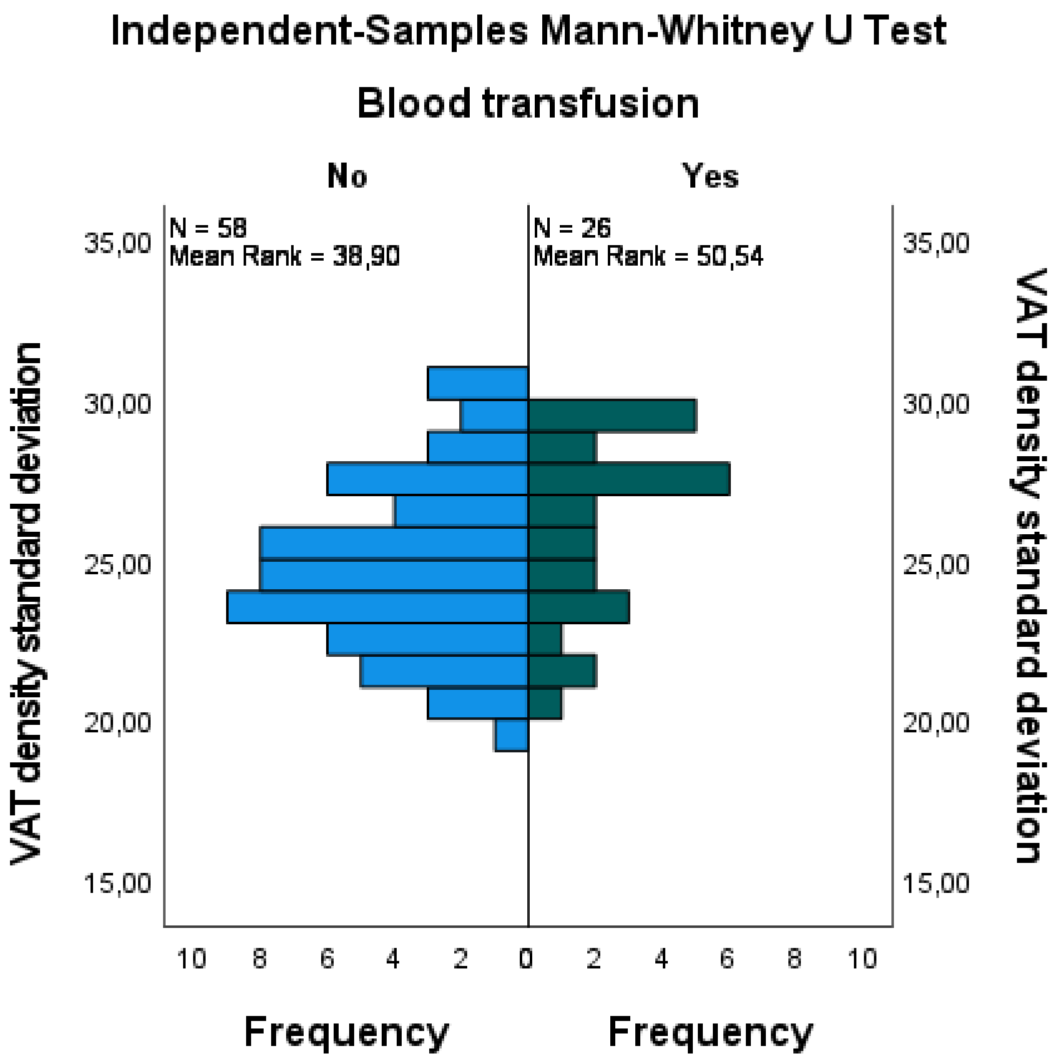

Right Psoas muscle mean density (HU) across Blood transfusion

Independent-Samples Mann-Whitney U Test Summary

Total N	85
Mann-Whitney U	809,000
Wilcoxon W	1160,000
Test Statistic	809,000
Standard Error	104,828
Standardized Test Statistic	,401
Asymptotic Sig.(2-sided test)	,689

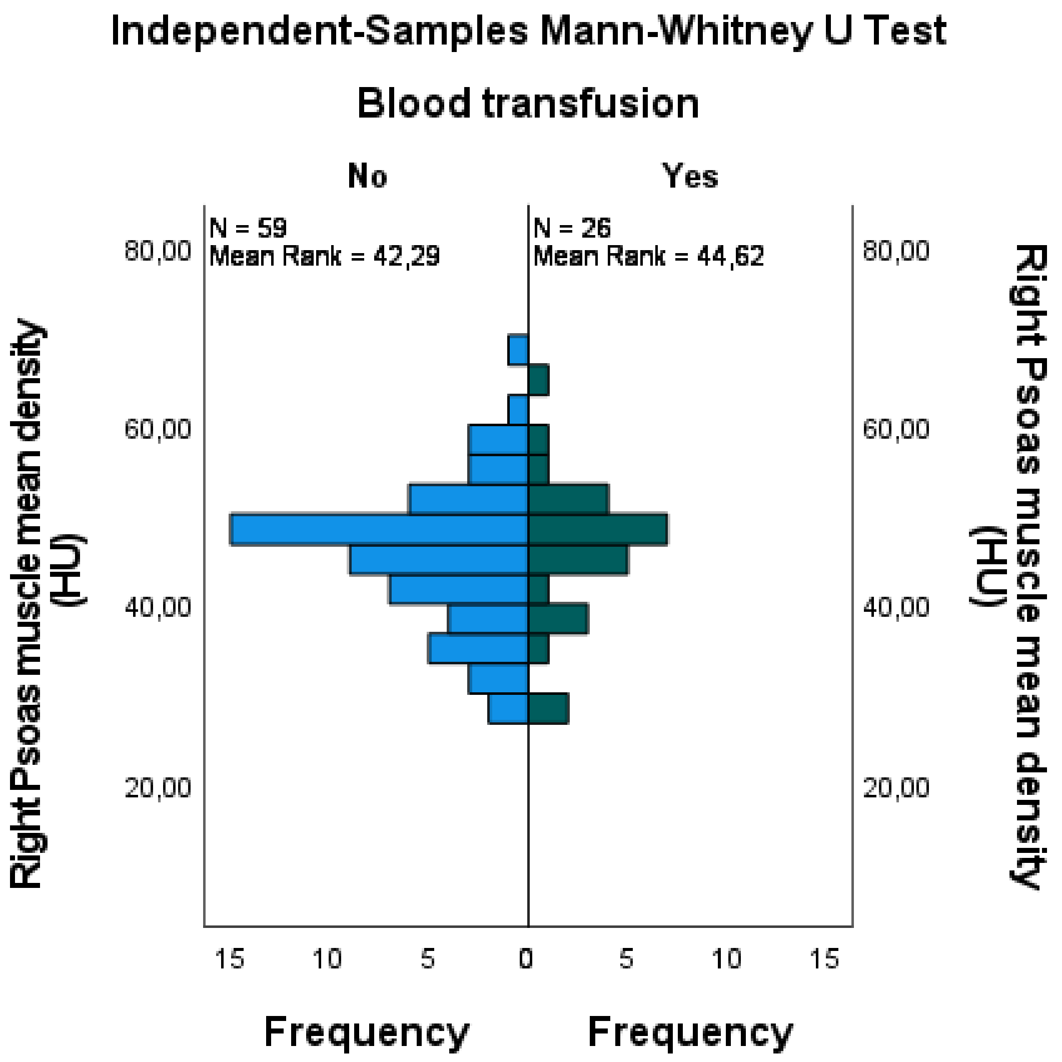

Right Psoas muscle median density (HU) across Blood transfusion

Independent-Samples Mann-Whitney U Test Summary

Total N	85
Mann-Whitney U	872,500
Wilcoxon W	1223,500
Test Statistic	872,500
Standard Error	104,751
Standardized Test Statistic	1,007
Asymptotic Sig.(2-sided test)	,314

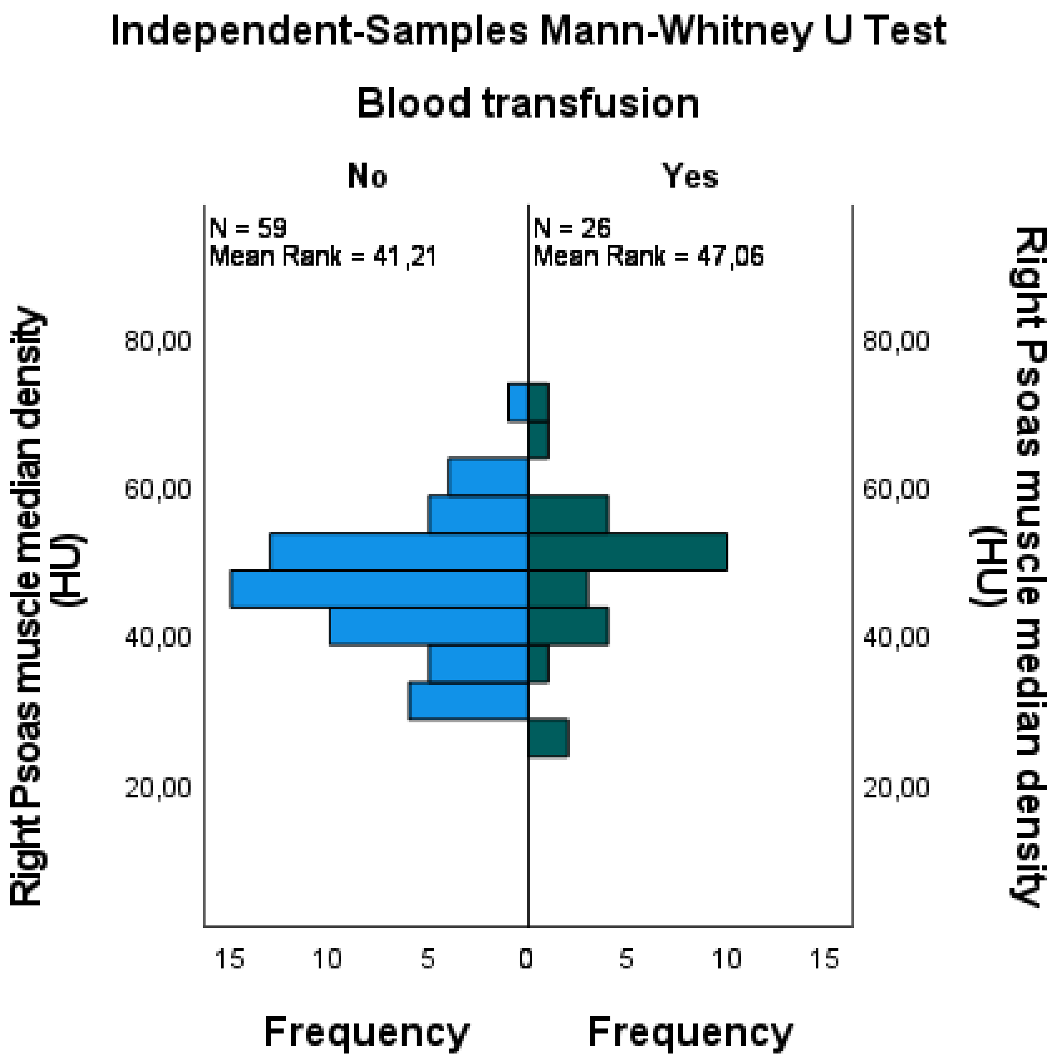

Right Psoas muscle density standard deviation across Blood transfusion

Independent-Samples Mann-Whitney U Test Summary

Total N	85
Mann-Whitney U	528,000
Wilcoxon W	879,000
Test Statistic	528,000
Standard Error	104,849
Standardized Test Statistic	-2,279
Asymptotic Sig.(2-sided test)	,023

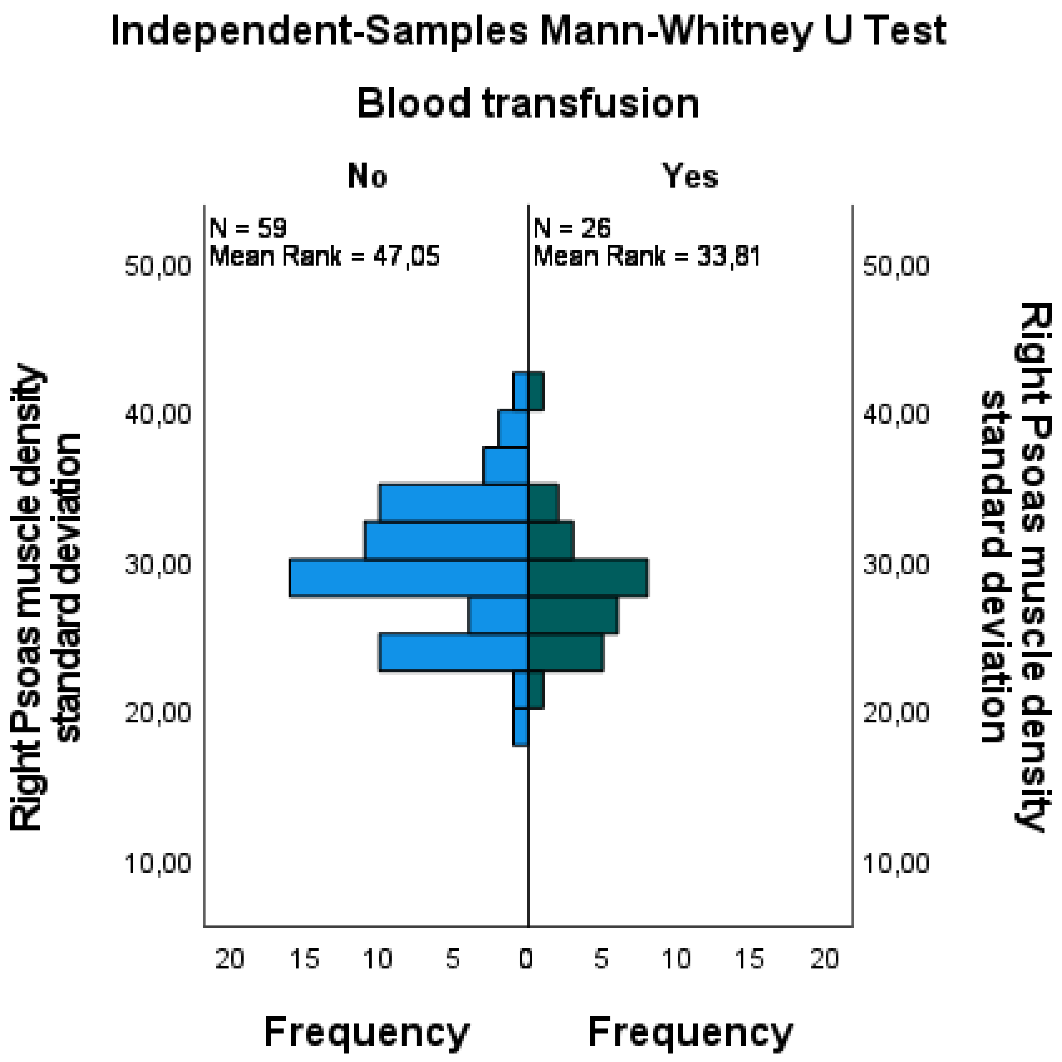

Left Psoas muscle mean density (HU) across Blood transfusion

Independent-Samples Mann-Whitney U Test Summary

Total N	85
Mann-Whitney U	734,500
Wilcoxon W	1085,500
Test Statistic	734,500
Standard Error	104,829
Standardized Test Statistic	-,310
Asymptotic Sig.(2-sided test)	,757

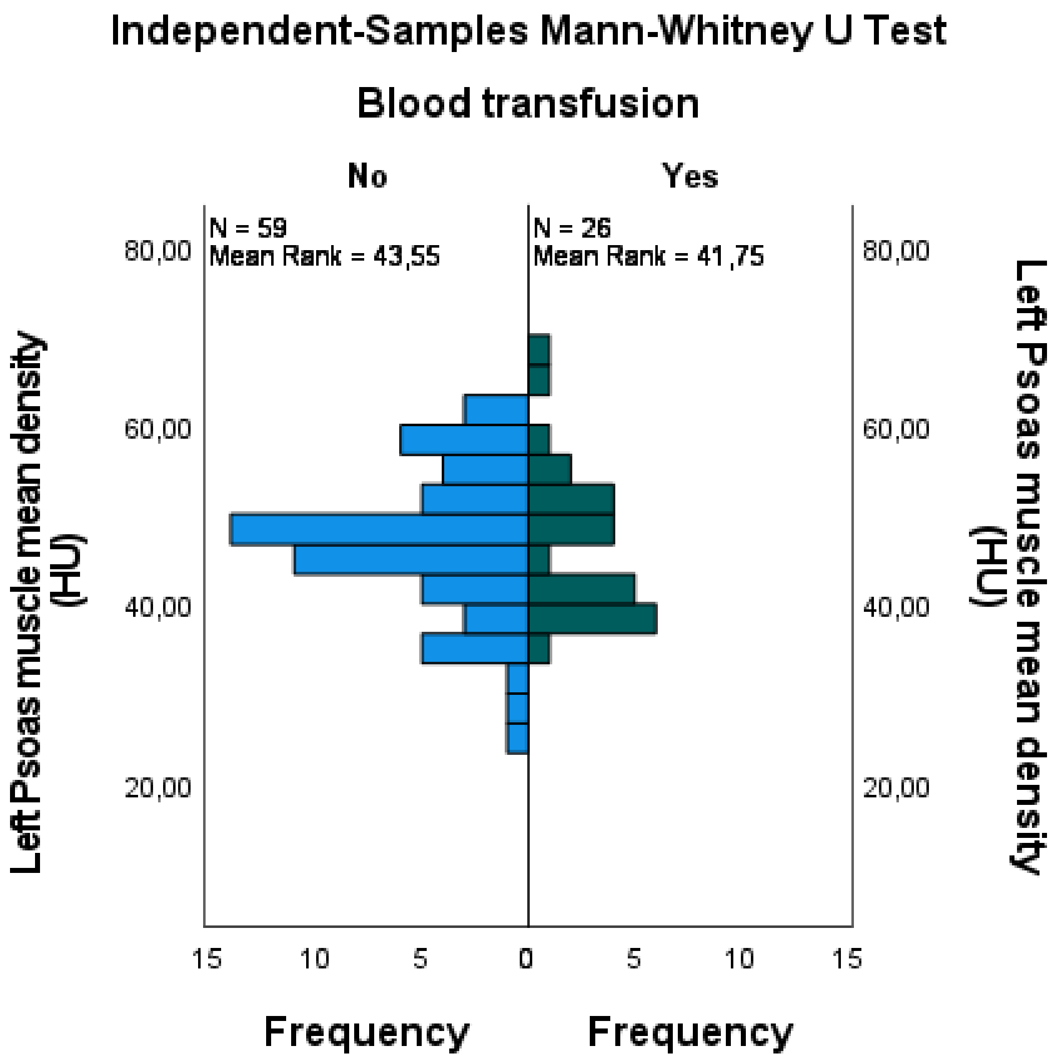

Left Psoas muscle median density (HU) across Blood transfusion

Independent-Samples Mann-Whitney U Test Summary

Total N	85
Mann-Whitney U	762,500
Wilcoxon W	1113,500
Test Statistic	762,500
Standard Error	104,717
Standardized Test Statistic	-,043
Asymptotic Sig.(2-sided test)	,966

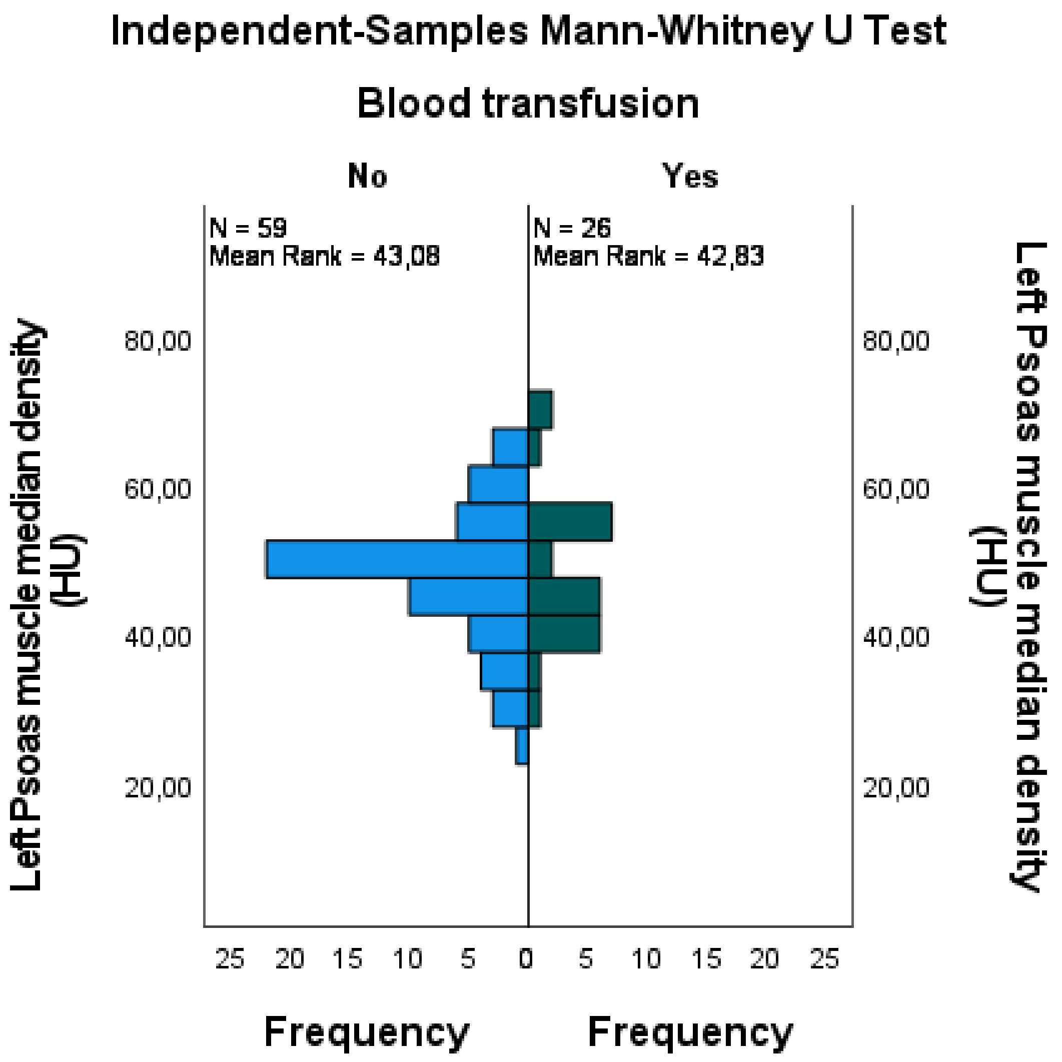

Left Psoas muscle density standard deviation across Blood transfusion

Independent-Samples Mann-Whitney U Test Summary

Total N	85
Mann-Whitney U	597,000
Wilcoxon W	948,000
Test Statistic	597,000
Standard Error	104,851
Standardized Test Statistic	-1,621
Asymptotic Sig.(2-sided test)	,105

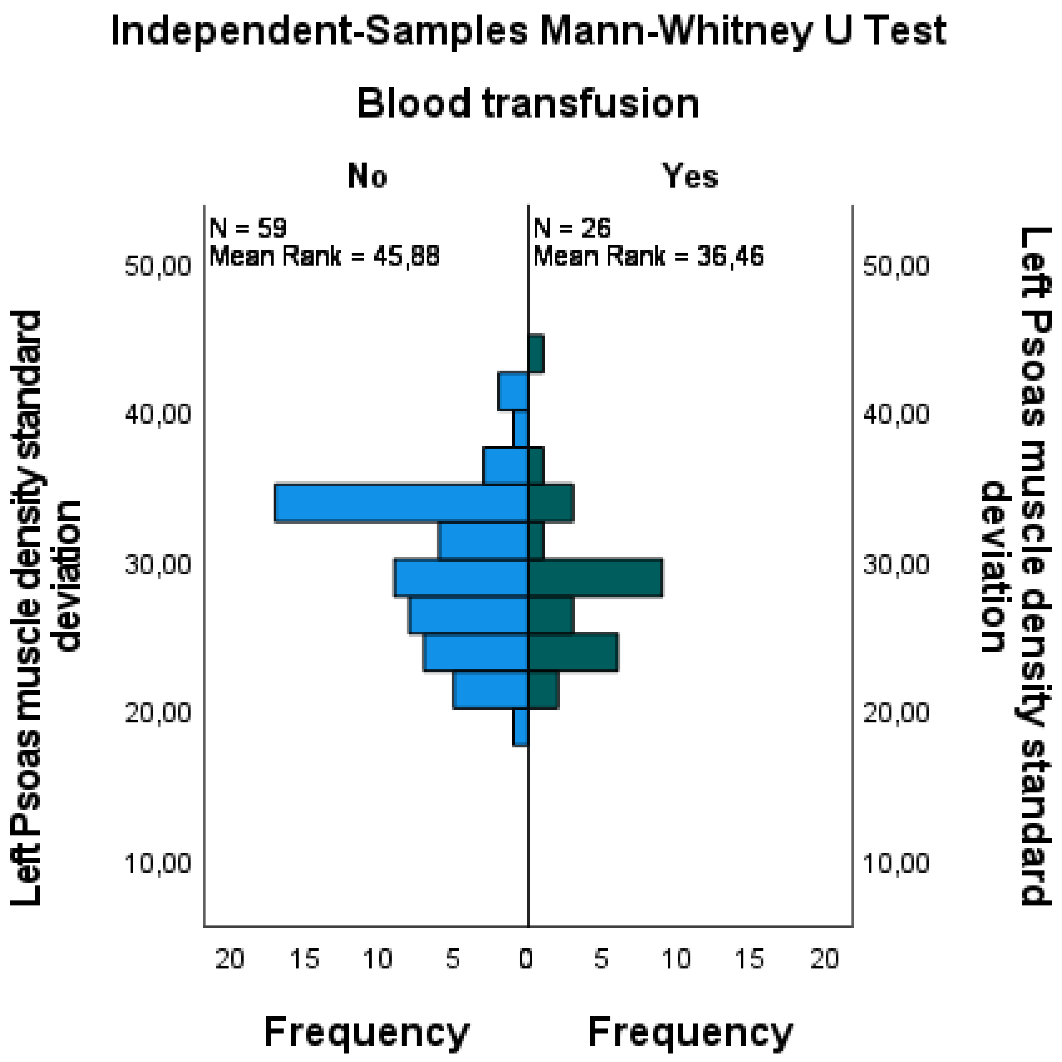

Independent-Samples Mann-Whitney U Test for prolonged hypotension

Hypothesis Test Summary

	Null Hypothesis	Test	Sig.[a,b]	Decision
1	The distribution of Psoas/height is the same across categories of Prolonged hypotension.	Independent-Samples Mann-Whitney U Test	,763	Retain the null hypothesis.
2	The distribution of Anterior SAT distance is the same across categories of Prolonged hypotension.	Independent-Samples Mann-Whitney U Test	,661	Retain the null hypothesis.
3	The distribution of Posterior SAT distance is the same across categories of Prolonged hypotension.	Independent-Samples Mann-Whitney U Test	,534	Retain the null hypothesis.
4	The distribution of Anterior+Posterior SAT distance is the same across categories of Prolonged hypotension.	Independent-Samples Mann-Whitney U Test	,748	Retain the null hypothesis.
5	The distribution of VAT distance is the same across categories of Prolonged hypotension.	Independent-Samples Mann-Whitney U Test	,426	Retain the null hypothesis.
6	The distribution of Right common femoral artery area (mm2) is the same across categories of Prolonged hypotension.	Independent-Samples Mann-Whitney U Test	,231	Retain the null hypothesis.
7	The distribution of Left common femoral artery area (mm2) is the same across categories of Prolonged hypotension.	Independent-Samples Mann-Whitney U Test	,694	Retain the null hypothesis.
8	The distribution of FAT area (cm2) is the same across categories of Prolonged hypotension.	Independent-Samples Mann-Whitney U Test	,656	Retain the null hypothesis.

9	The distribution of SAT area (cm2) is the same across categories of Prolonged hypotension.	Independent-Samples Mann-Whitney U Test	,614	Retain the null hypothesis.
10	The distribution of VAT area (cm2) is the same across categories of Prolonged hypotension.	Independent-Samples Mann-Whitney U Test	,868	Retain the null hypothesis.
11	The distribution of Right Psoas muscle area (cm2) is the same across categories of Prolonged hypotension.	Independent-Samples Mann-Whitney U Test	,719	Retain the null hypothesis.
12	The distribution of Left Psoas muscle area (cm2) is the same across categories of Prolonged hypotension.	Independent-Samples Mann-Whitney U Test	,709	Retain the null hypothesis.
13	The distribution of FAT mean density (HU) is the same across categories of Prolonged hypotension.	Independent-Samples Mann-Whitney U Test	,719	Retain the null hypothesis.
14	The distribution of FAT median density (HU) is the same across categories of Prolonged hypotension.	Independent-Samples Mann-Whitney U Test	,714	Retain the null hypothesis.
15	The distribution of FAT density standard deviation is the same across categories of Prolonged hypotension.	Independent-Samples Mann-Whitney U Test	,547	Retain the null hypothesis.
16	The distribution of SAT mean density (HU) is the same across categories of Prolonged hypotension.	Independent-Samples Mann-Whitney U Test	,534	Retain the null hypothesis.
17	The distribution of SAT median density (HU) is the same across categories of Prolonged hypotension.	Independent-Samples Mann-Whitney U Test	,337	Retain the null hypothesis.
18	The distribution of SAT density standard deviation is the same across categories of Prolonged hypotension.	Independent-Samples Mann-Whitney U Test	,995	Retain the null hypothesis.

19	The distribution of VAT mean density (HU) is the same across categories of Prolonged hypotension.	Independent-Samples Mann-Whitney U Test	,455	Retain the null hypothesis.
20	The distribution of VAT median density (HU) is the same across categories of Prolonged hypotension.	Independent-Samples Mann-Whitney U Test	,609	Retain the null hypothesis.
21	The distribution of VAT density standard deviation is the same across categories of Prolonged hypotension.	Independent-Samples Mann-Whitney U Test	,827	Retain the null hypothesis.
22	The distribution of Right Psoas muscle mean density (HU) is the same across categories of Prolonged hypotension.	Independent-Samples Mann-Whitney U Test	,424	Retain the null hypothesis.
23	The distribution of Right Psoas muscle median density (HU) is the same across categories of Prolonged hypotension.	Independent-Samples Mann-Whitney U Test	,329	Retain the null hypothesis.
24	The distribution of Right Psoas muscle density standard deviation is the same across categories of Prolonged hypotension.	Independent-Samples Mann-Whitney U Test	,788	Retain the null hypothesis.
25	The distribution of Left Psoas muscle mean density (HU) is the same across categories of Prolonged hypotension.	Independent-Samples Mann-Whitney U Test	,814	Retain the null hypothesis.
26	The distribution of Left Psoas muscle median density (HU) is the same across categories of Prolonged hypotension.	Independent-Samples Mann-Whitney U Test	,564	Retain the null hypothesis.
27	The distribution of Left Psoas muscle density standard deviation is the same across categories of Prolonged hypotension.	Independent-Samples Mann-Whitney U Test	,948	Retain the null hypothesis.

a. The significance level is ,050.

b. Asymptotic significance is displayed.

In this case, the hypothesis of equal medians is accepted for all the variables ($p > 0.05$).

(The tables and graphs below are the details of the tests in this table: I have highlighted what things you should eventually report, namely test statistic and pvalue).

Psoas/height across Prolonged hypotension

Independent-Samples Mann-Whitney U Test Summary

Total N	85
Mann-Whitney U	384,000
Wilcoxon W	450,000
Test Statistic	384,000
Standard Error	76,378
Standardized Test Statistic	-,301
Asymptotic Sig.(2-sided test)	,763

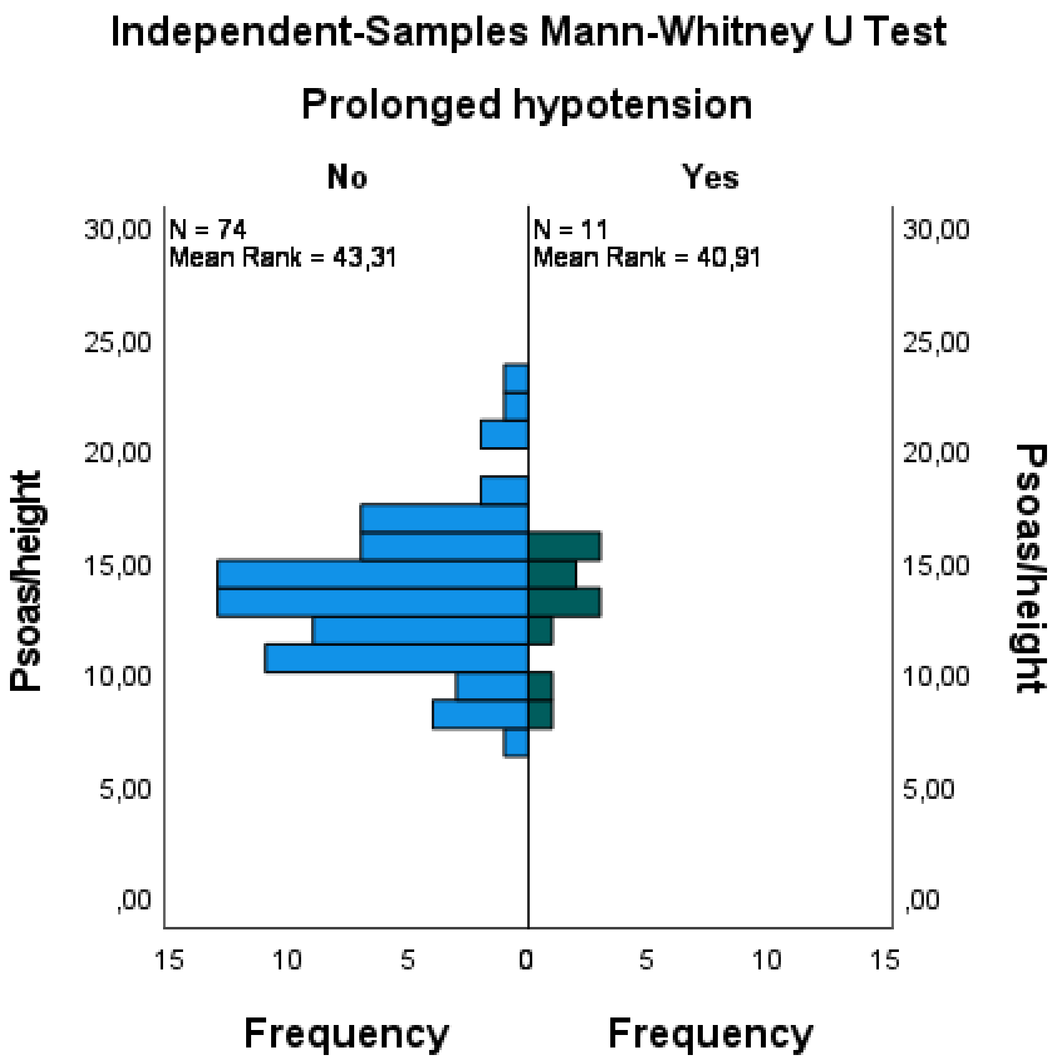

Anterior SAT distance across Prolonged hypotension

Independent-Samples Mann-Whitney U Test Summary

Total N	85
Mann-Whitney U	440,500
Wilcoxon W	506,500
Test Statistic	440,500
Standard Error	76,368
Standardized Test Statistic	,439
Asymptotic Sig.(2-sided test)	,661

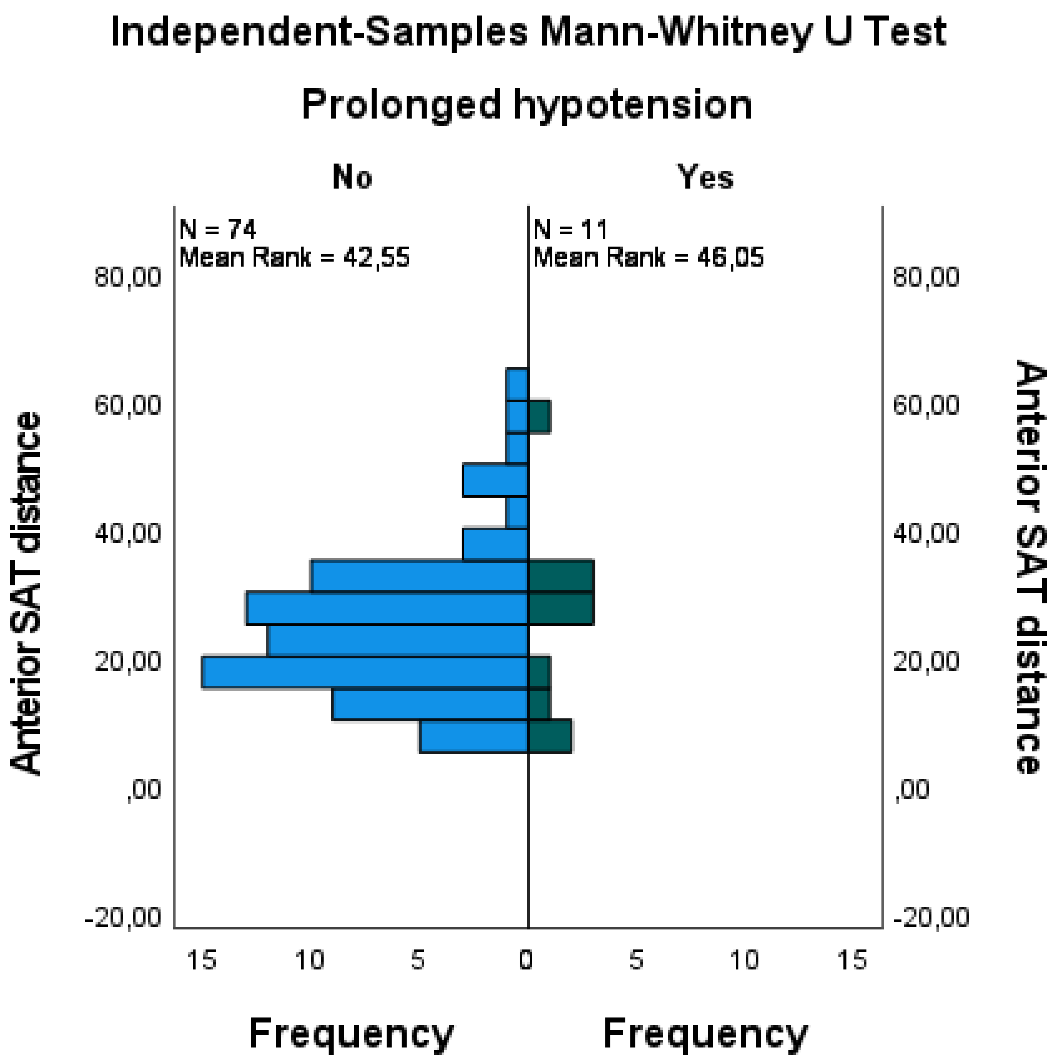

Posterior SAT distance across Prolonged hypotension

Independent-Samples Mann-Whitney U Test Summary

Total N	85
Mann-Whitney U	359,500
Wilcoxon W	425,500
Test Statistic	359,500
Standard Error	76,370
Standardized Test Statistic	-,622
Asymptotic Sig.(2-sided test)	,534

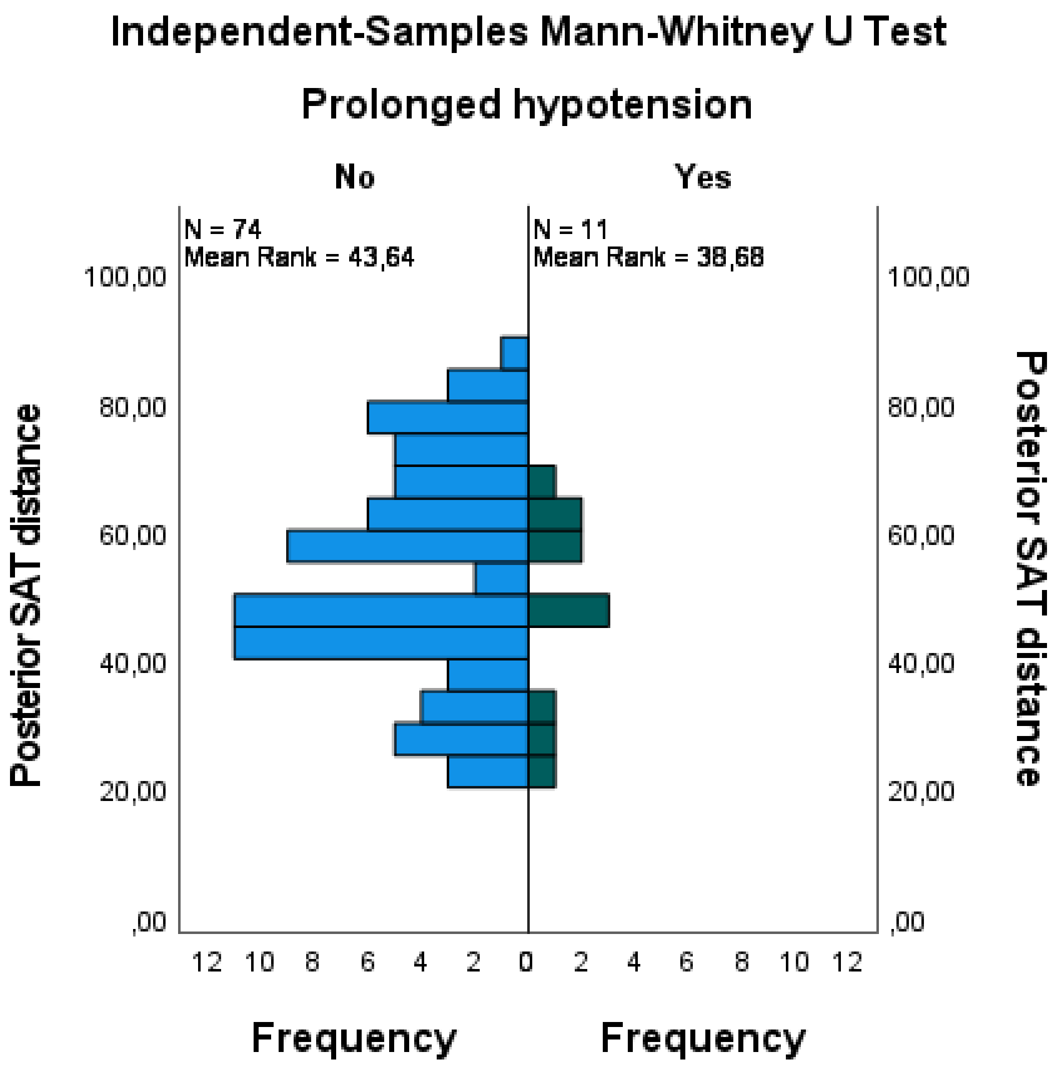

Anterior+Posterior SAT distance across Prolonged hypotension

Independent-Samples Mann-Whitney U Test Summary

Total N	85
Mann-Whitney U	382,500
Wilcoxon W	448,500
Test Statistic	382,500
Standard Error	76,375
Standardized Test Statistic	-,321
Asymptotic Sig.(2-sided test)	,748

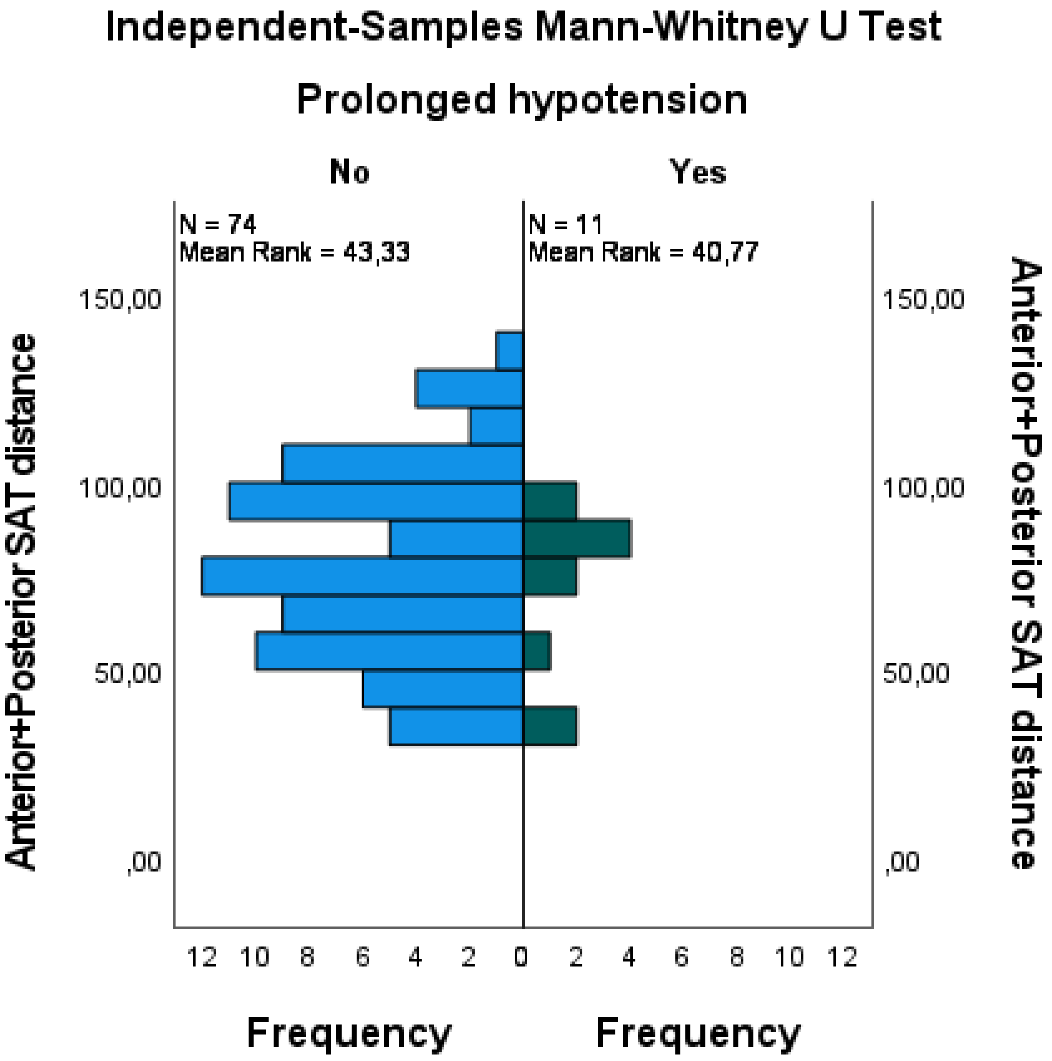

VAT distance across Prolonged hypotension

Independent-Samples Mann-Whitney U Test Summary

Total N	84
Mann-Whitney U	461,500
Wilcoxon W	527,500
Test Statistic	461,500
Standard Error	75,403
Standardized Test Statistic	,796
Asymptotic Sig.(2-sided test)	,426

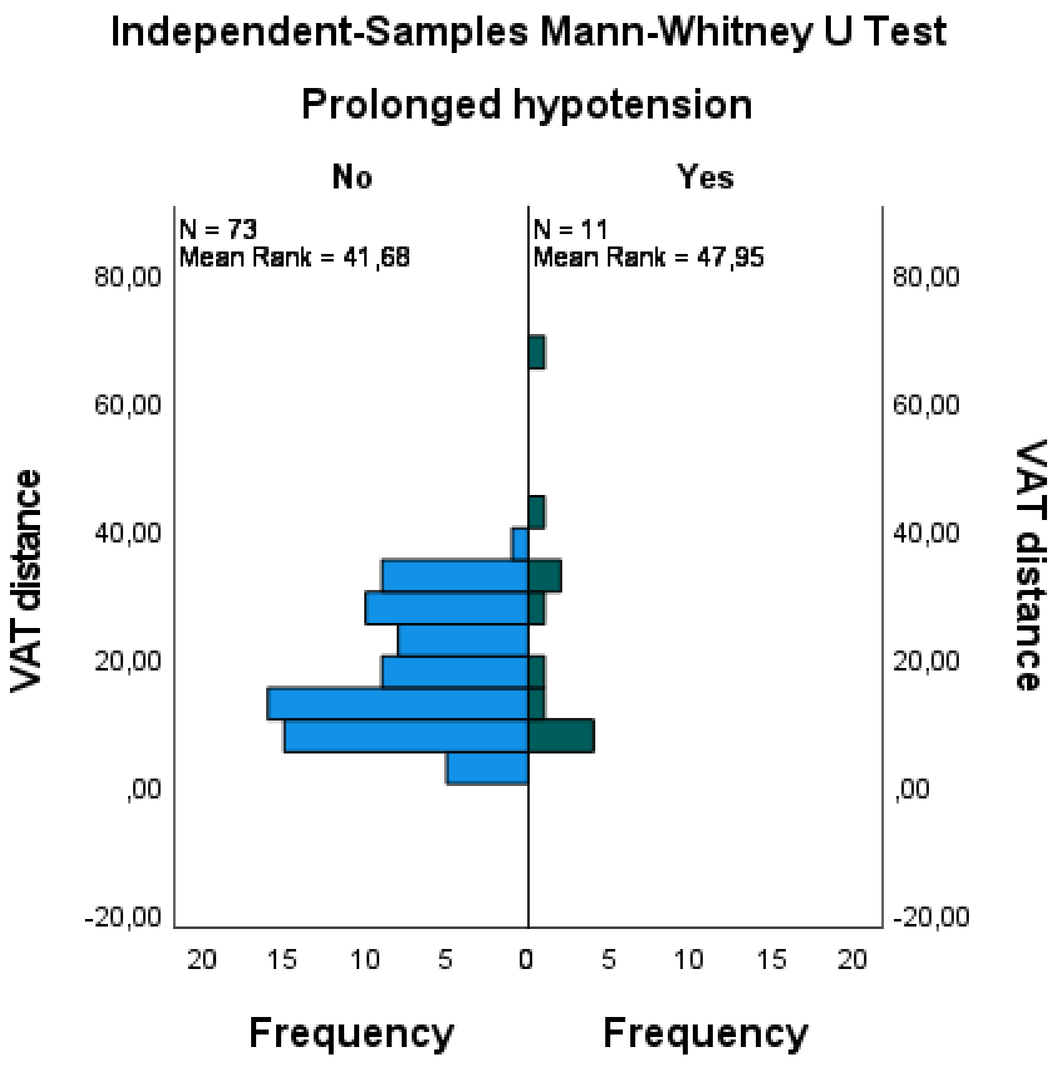

Right common femoral artery area (mm2) across Prolonged hypotension

Independent-Samples Mann-Whitney U Test Summary

Total N	85
Mann-Whitney U	315,500
Wilcoxon W	381,500
Test Statistic	315,500
Standard Error	76,366
Standardized Test Statistic	-1,198
Asymptotic Sig.(2-sided test)	,231

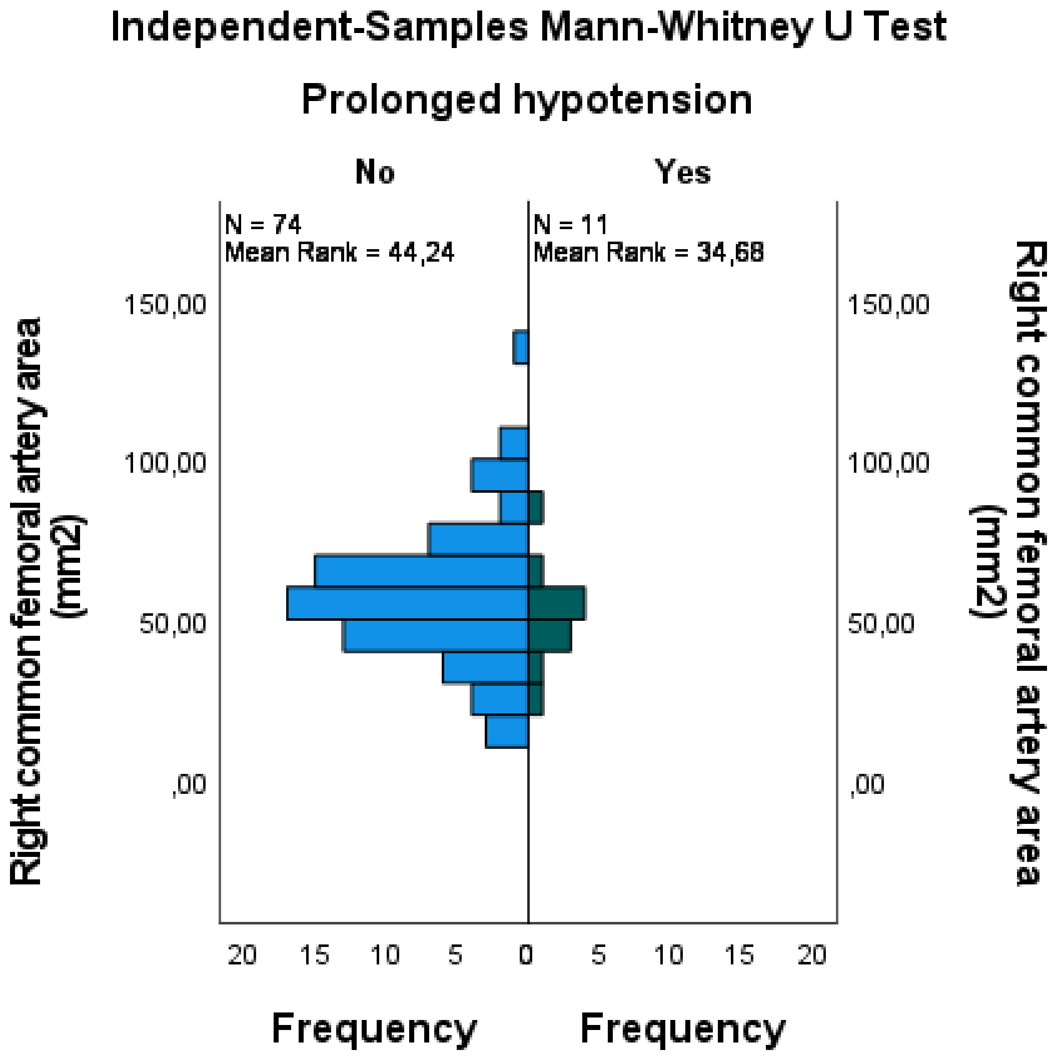

Left common femoral artery area (mm2) across Prolonged hypotension

Independent-Samples Mann-Whitney U Test Summary

Total N	85
Mann-Whitney U	377,000
Wilcoxon W	443,000
Test Statistic	377,000
Standard Error	76,360
Standardized Test Statistic	-,393
Asymptotic Sig.(2-sided test)	,694

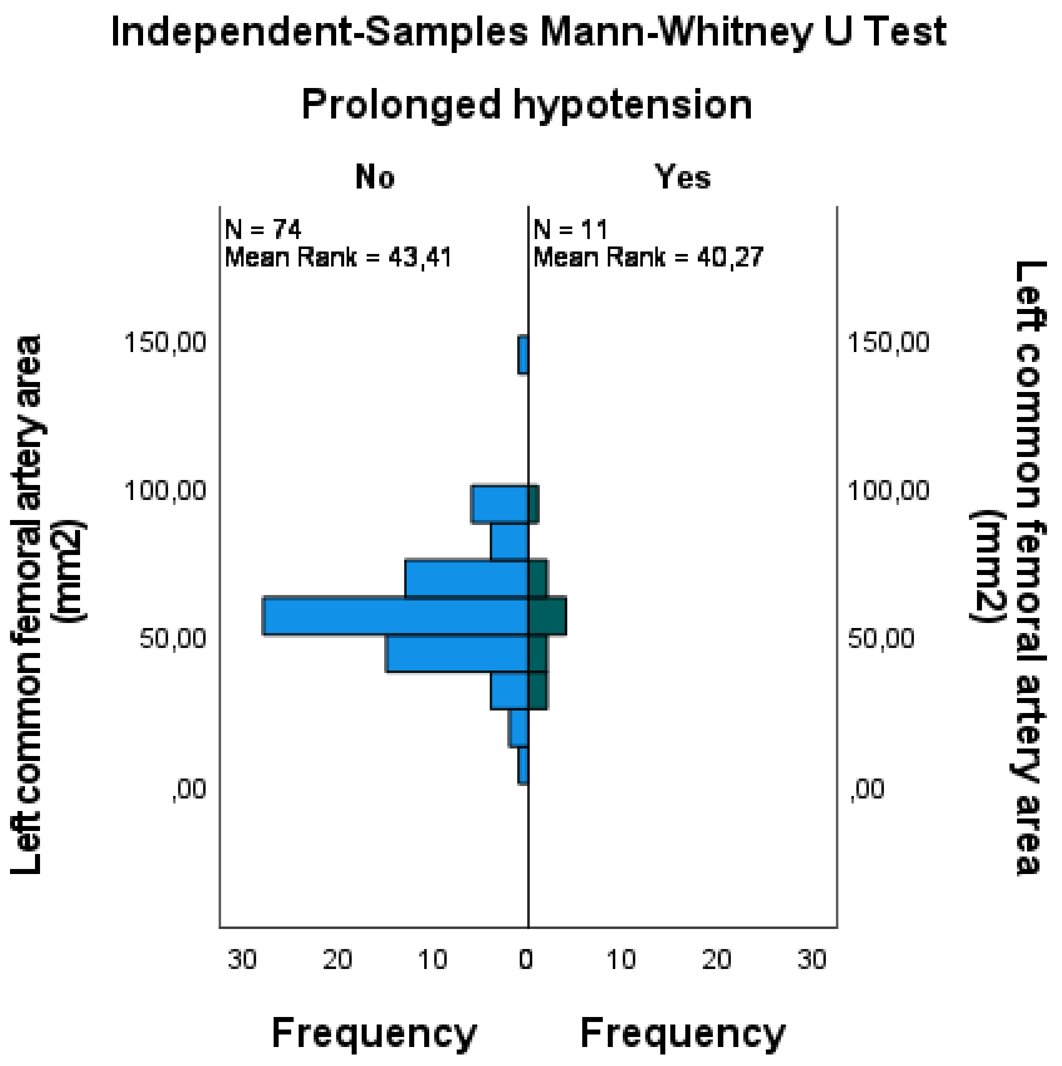

FAT area (cm2) across Prolonged hypotension

Independent-Samples Mann-Whitney U Test Summary

Total N	85
Mann-Whitney U	373,000
Wilcoxon W	439,000
Test Statistic	373,000
Standard Error	76,378
Standardized Test Statistic	-,445
Asymptotic Sig.(2-sided test)	,656

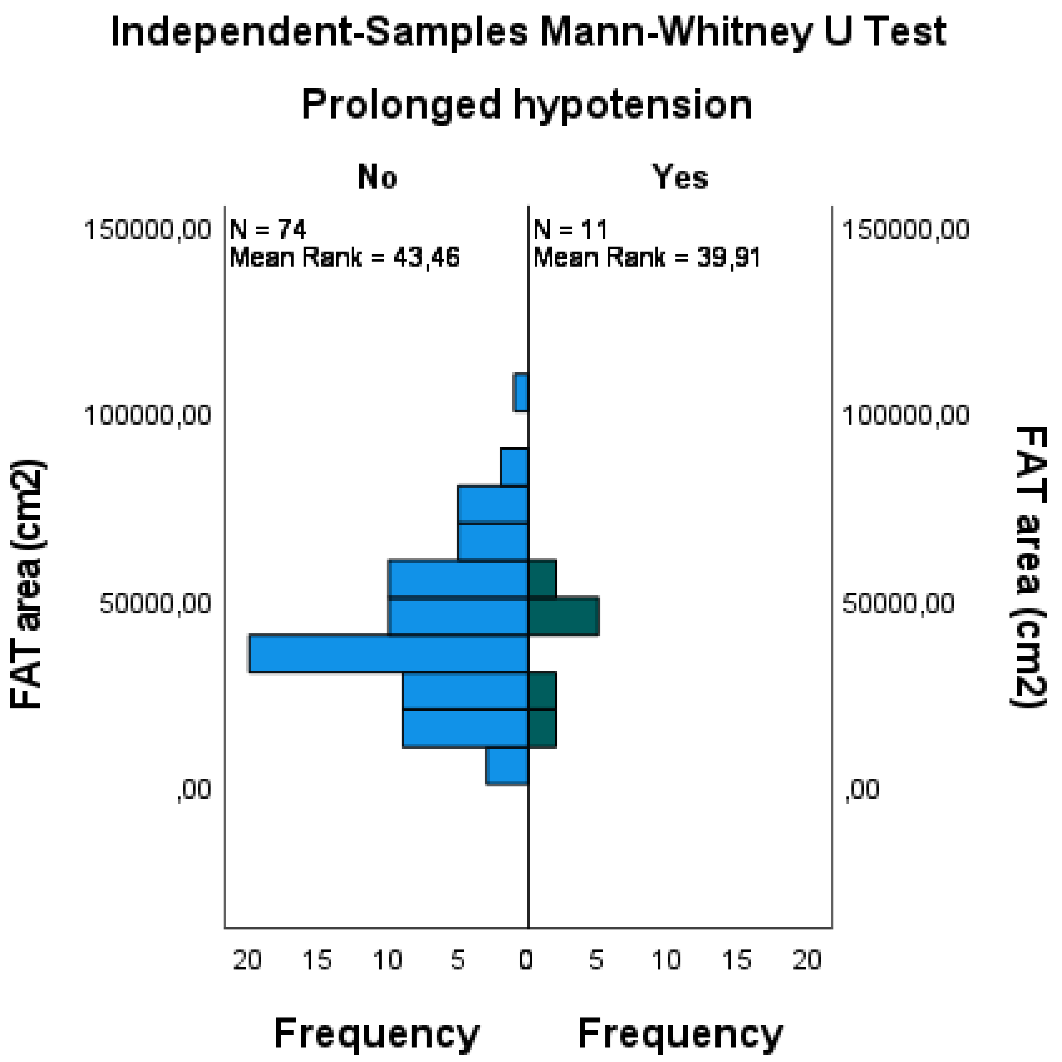

SAT area (cm2) across Prolonged hypotension

Independent-Samples Mann-Whitney U Test Summary

Total N	85
Mann-Whitney U	368,500
Wilcoxon W	434,500
Test Statistic	368,500
Standard Error	76,378
Standardized Test Statistic	-,504
Asymptotic Sig.(2-sided test)	,614

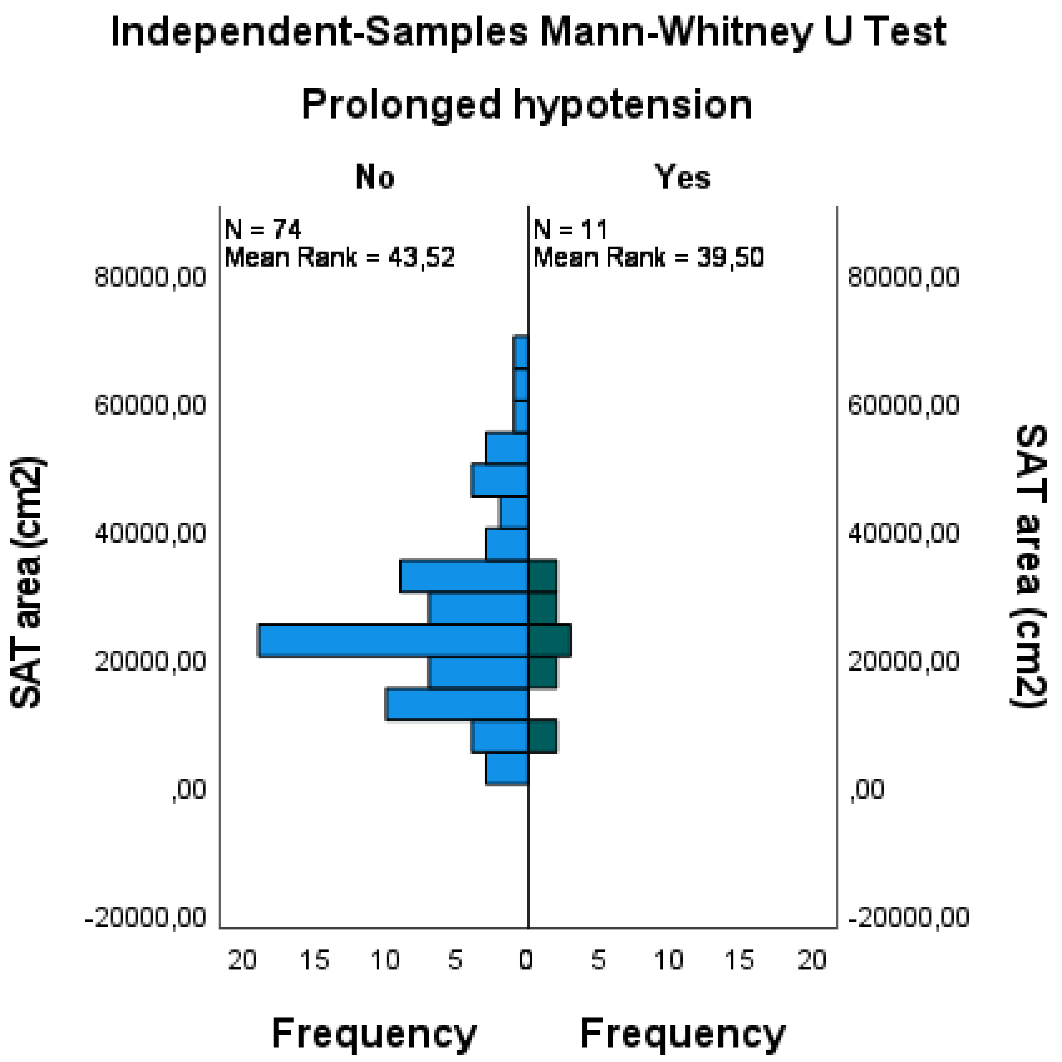

VAT area (cm2) across Prolonged hypotension

Independent-Samples Mann-Whitney U Test Summary

Total N	84
Mann-Whitney U	389,000
Wilcoxon W	455,000
Test Statistic	389,000
Standard Error	75,418
Standardized Test Statistic	-,166
Asymptotic Sig.(2-sided test)	,868

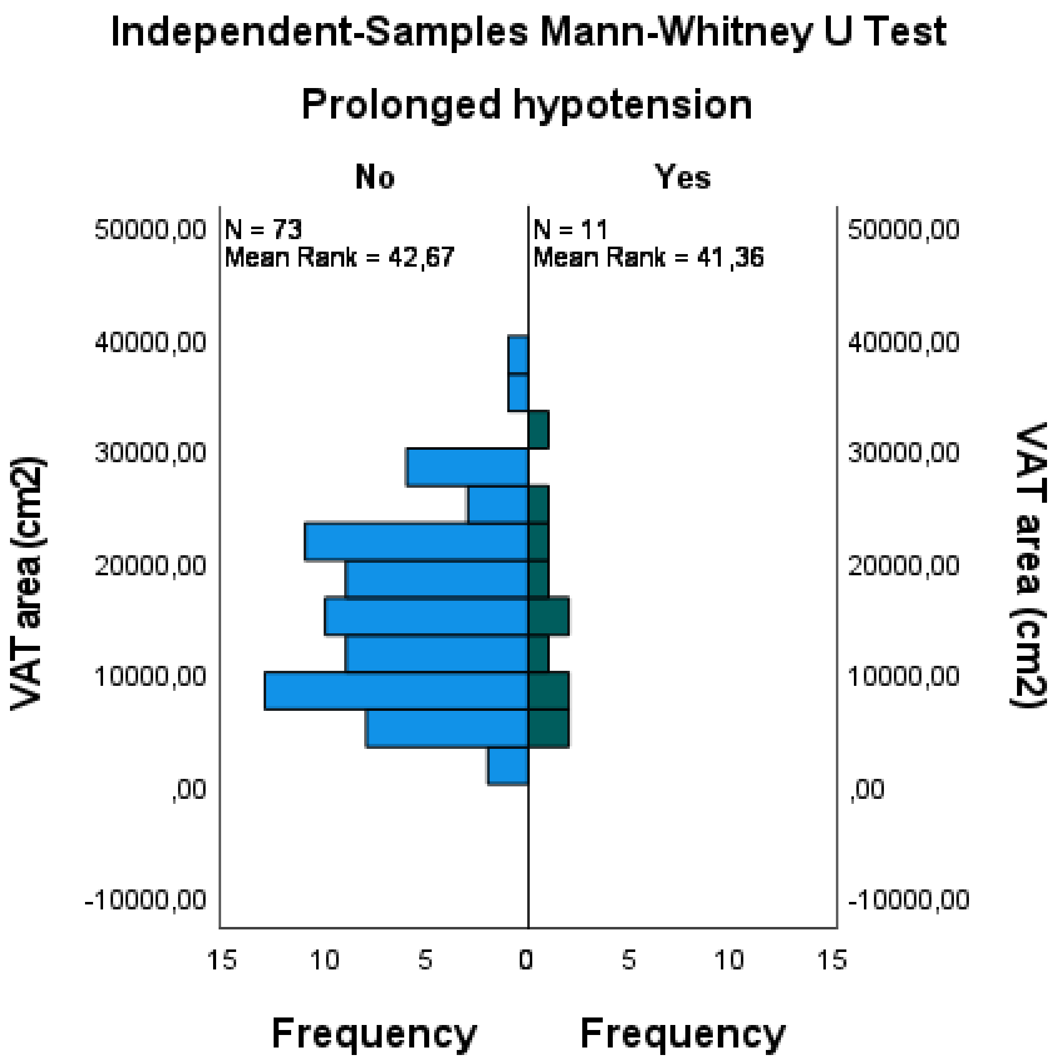

Right Psoas muscle area (cm2) across Prolonged hypotension

Independent-Samples Mann-Whitney U Test Summary

Total N	85
Mann-Whitney U	434,500
Wilcoxon W	500,500
Test Statistic	434,500
Standard Error	76,376
Standardized Test Statistic	,360
Asymptotic Sig.(2-sided test)	,719

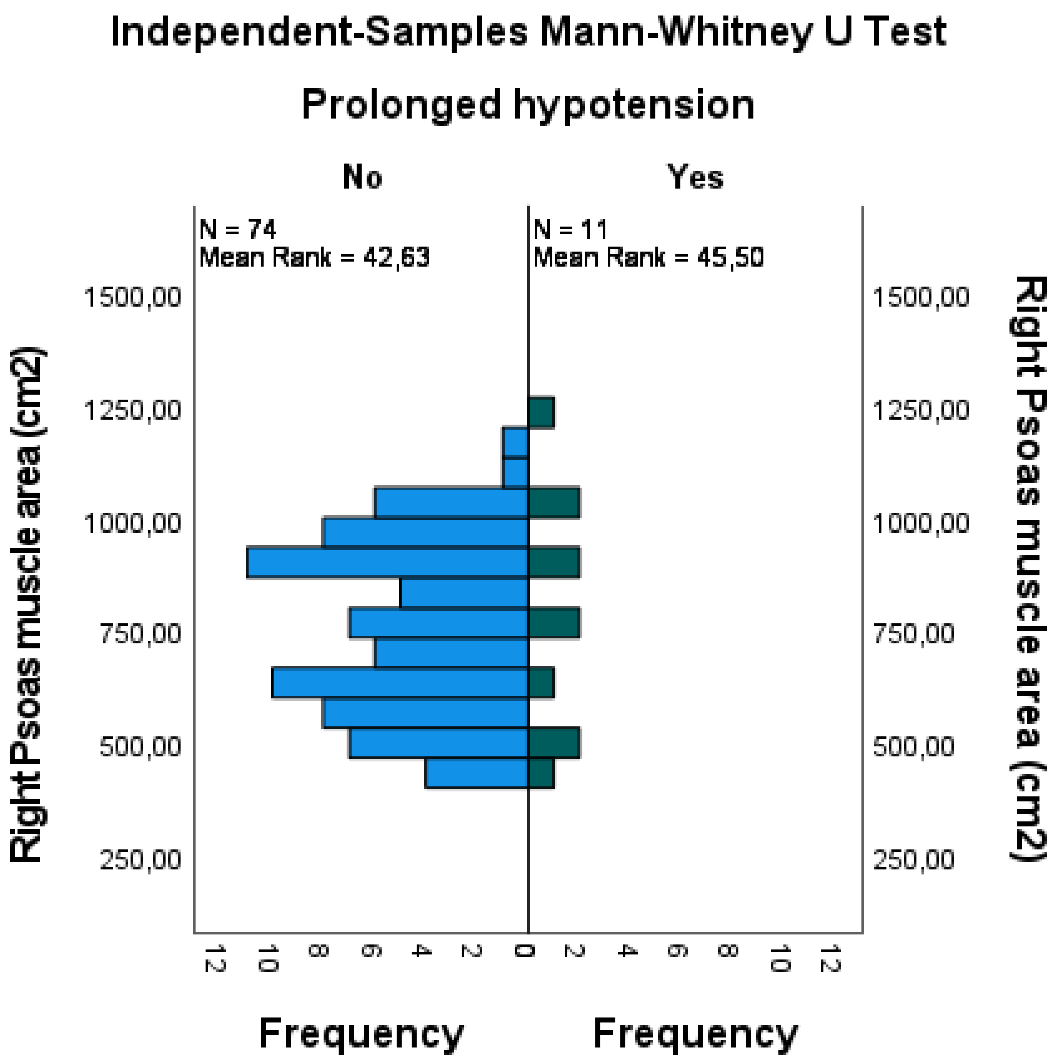

Left Psoas muscle area (cm2) across Prolonged hypotension

Independent-Samples Mann-Whitney U Test Summary

Total N	85
Mann-Whitney U	378,500
Wilcoxon W	444,500
Test Statistic	378,500
Standard Error	76,378
Standardized Test Statistic	-,373
Asymptotic Sig.(2-sided test)	,709

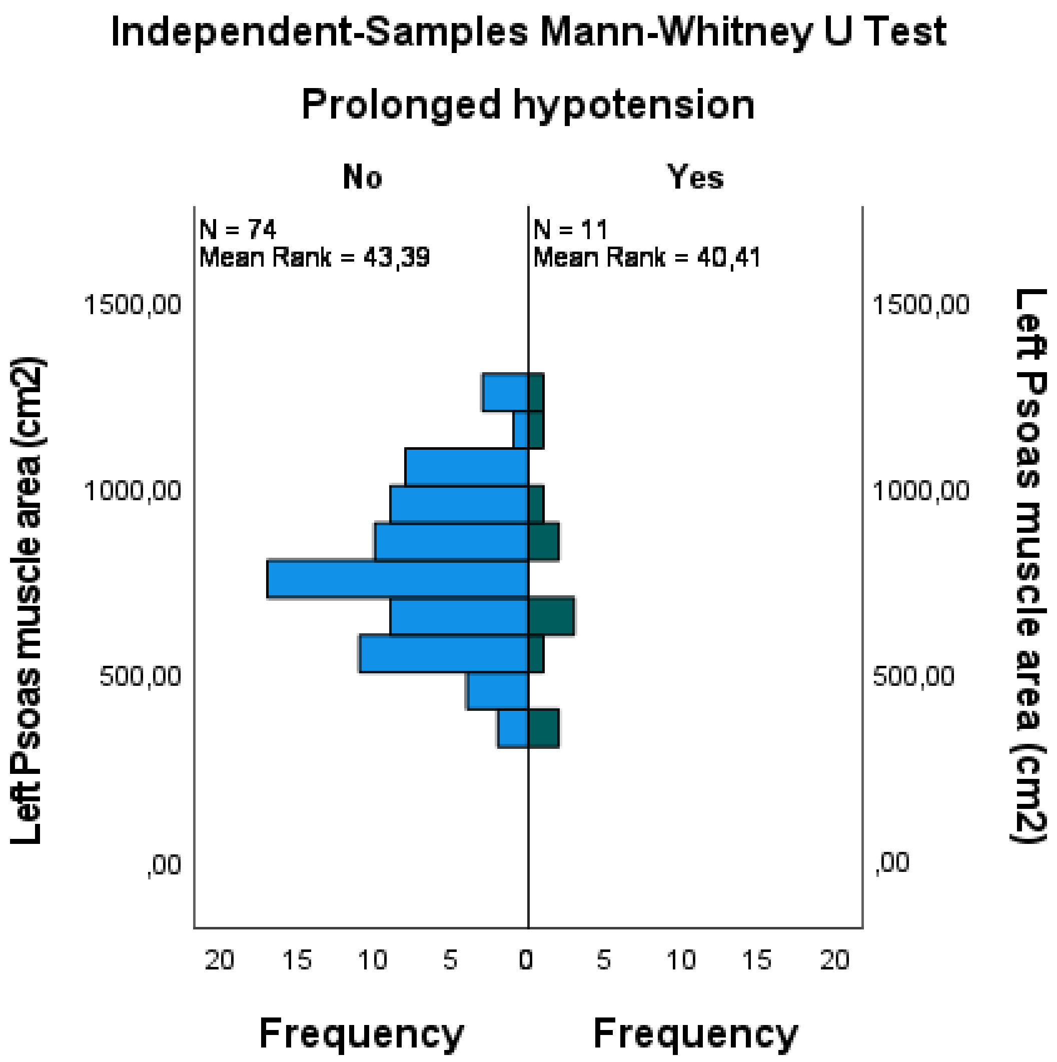

FAT mean density (HU) across Prolonged hypotension

Independent-Samples Mann-Whitney U Test Summary

Total N	85
Mann-Whitney U	434,500
Wilcoxon W	500,500
Test Statistic	434,500
Standard Error	76,366
Standardized Test Statistic	,360
Asymptotic Sig.(2-sided test)	,719

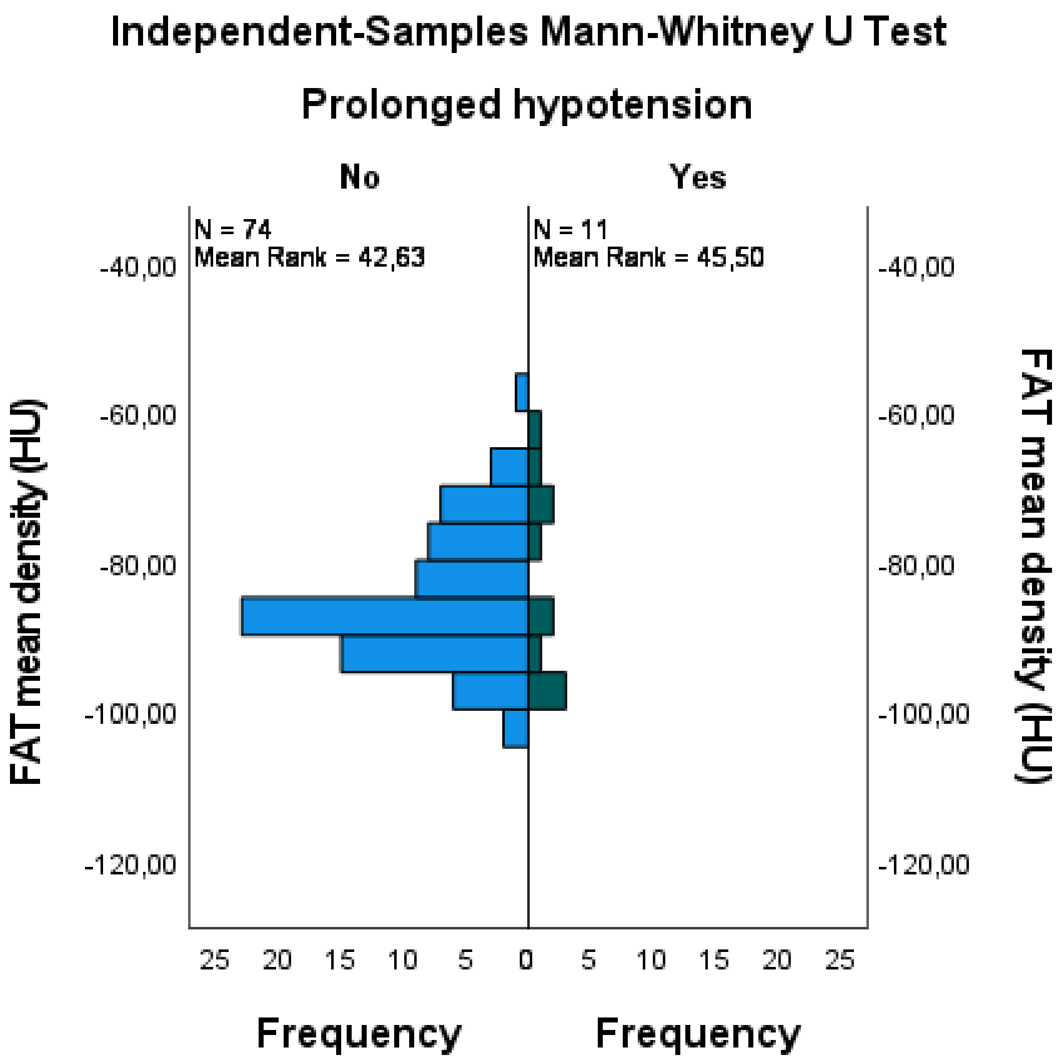

FAT median density (HU) across Prolonged hypotension

Independent-Samples Mann-Whitney U Test Summary

Total N	85
Mann-Whitney U	435,000
Wilcoxon W	501,000
Test Statistic	435,000
Standard Error	76,286
Standardized Test Statistic	,367
Asymptotic Sig.(2-sided test)	,714

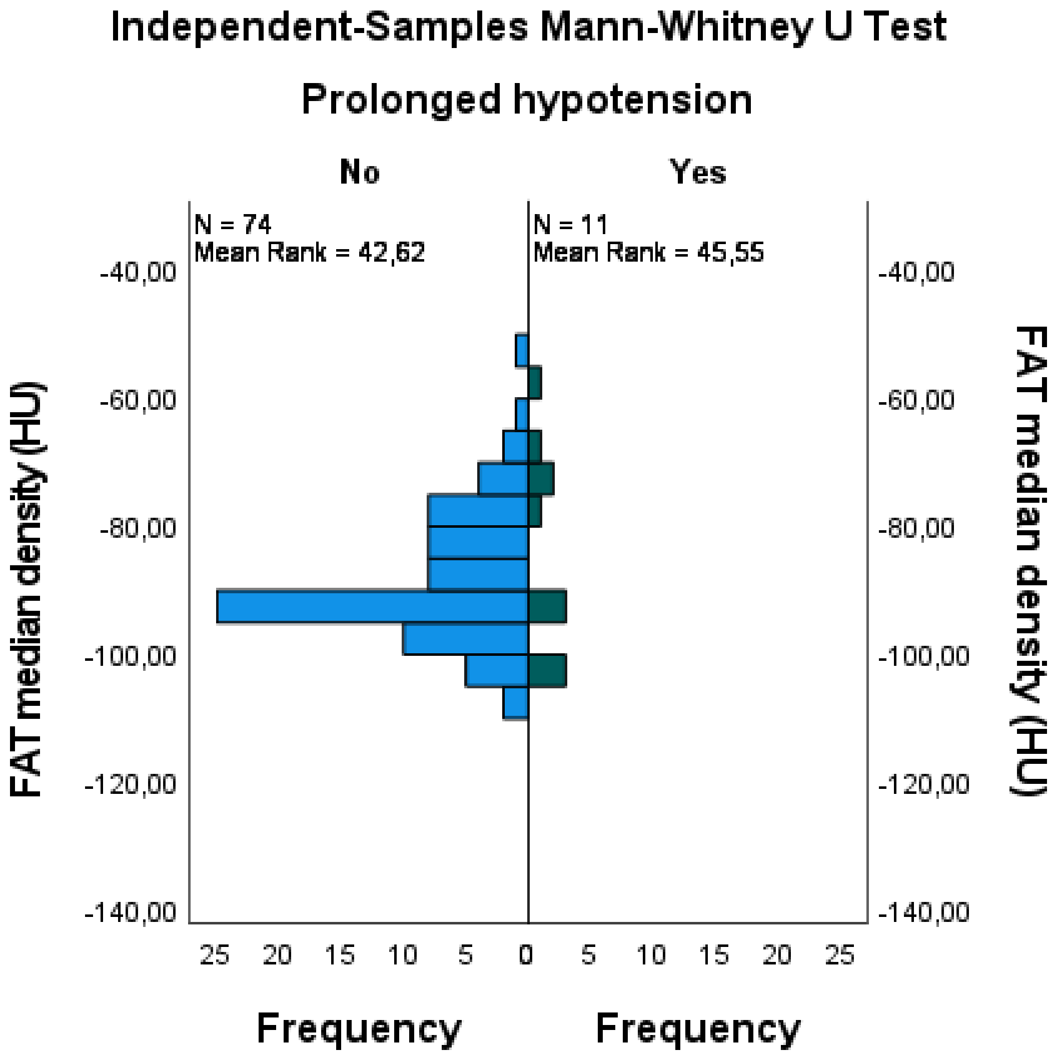

FAT density standard deviation across Prolonged hypotension

Independent-Samples Mann-Whitney U Test Summary

Total N	85
Mann-Whitney U	361,000
Wilcoxon W	427,000
Test Statistic	361,000
Standard Error	76,378
Standardized Test Statistic	-,602
Asymptotic Sig.(2-sided test)	,547

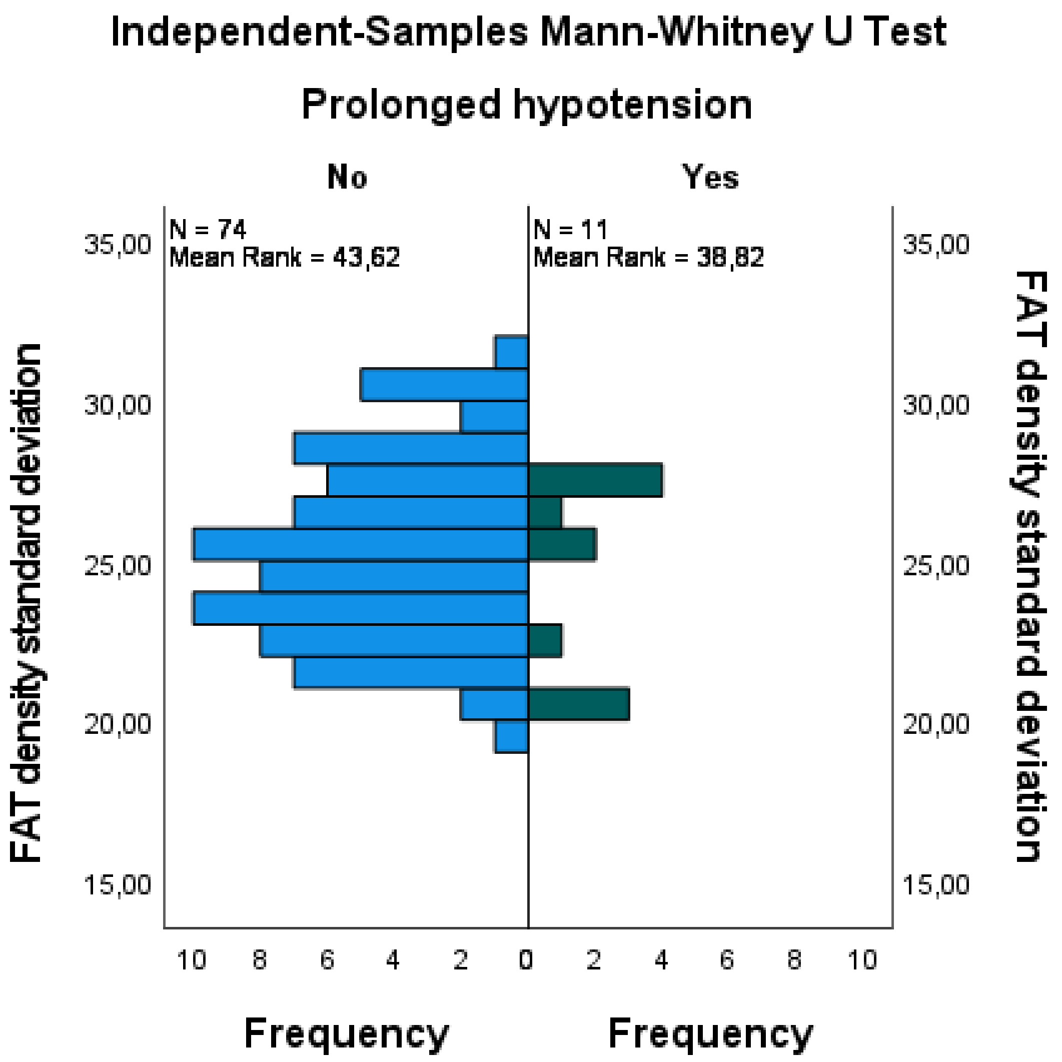

SAT mean density (HU) across Prolonged hypotension

Independent-Samples Mann-Whitney U Test Summary

Total N	85
Mann-Whitney U	454,500
Wilcoxon W	520,500
Test Statistic	454,500
Standard Error	76,369
Standardized Test Statistic	,622
Asymptotic Sig.(2-sided test)	,534

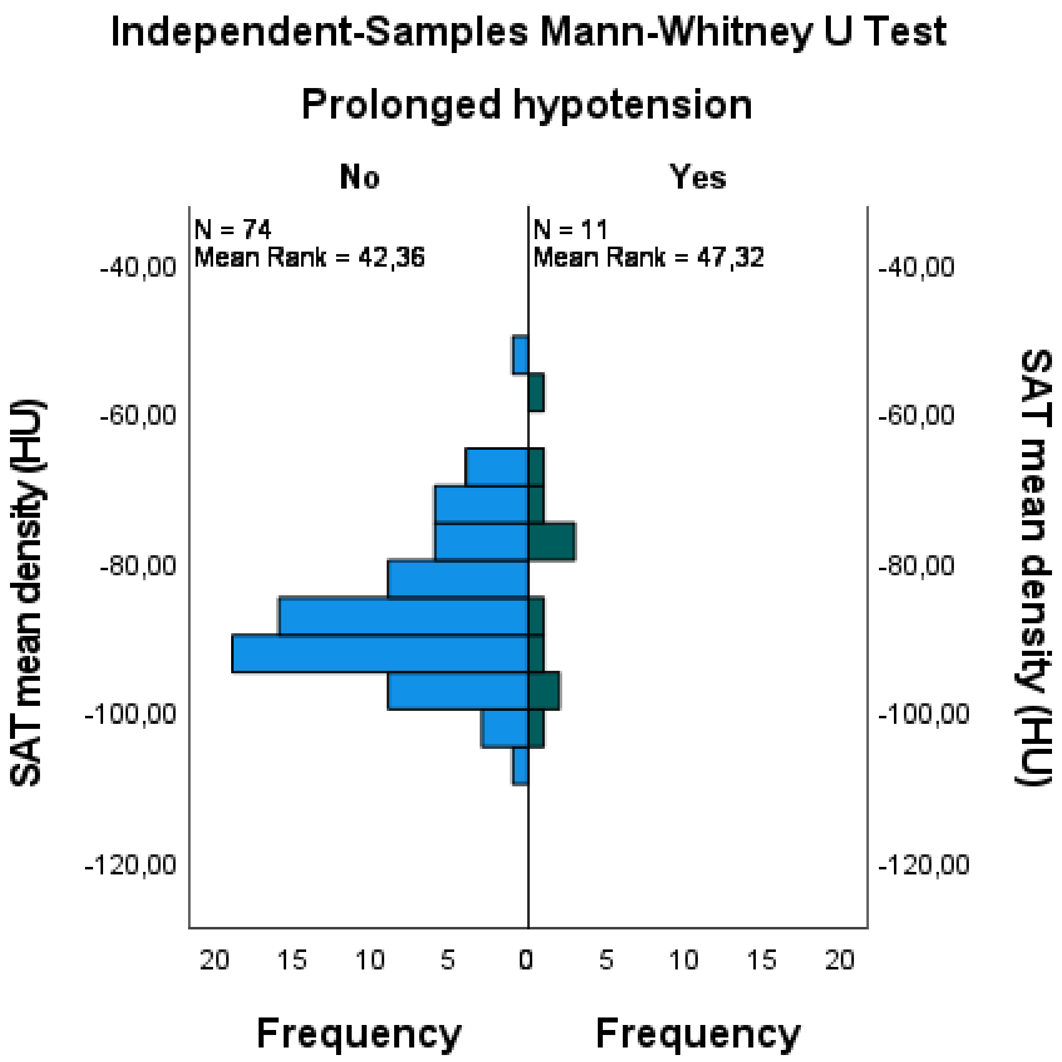

SAT median density (HU) across Prolonged hypotension

Independent-Samples Mann-Whitney U Test Summary

Total N	83
Mann-Whitney U	467,500
Wilcoxon W	533,500
Test Statistic	467,500
Standard Error	74,400
Standardized Test Statistic	,961
Asymptotic Sig.(2-sided test)	,337

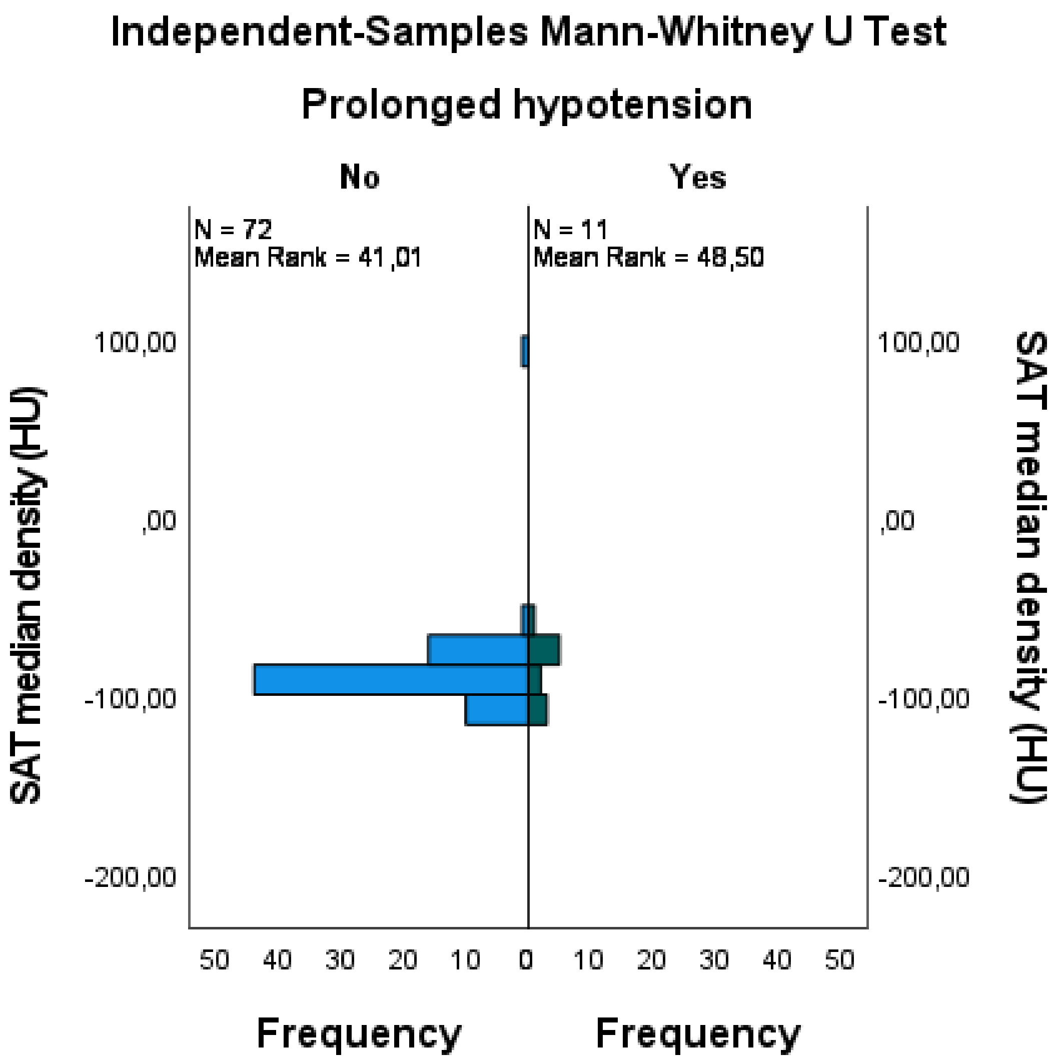

SAT density standard deviation across Prolonged hypotension

Independent-Samples Mann-Whitney U Test Summary

Total N	84
Mann-Whitney U	401,000
Wilcoxon W	467,000
Test Statistic	401,000
Standard Error	75,418
Standardized Test Statistic	-,007
Asymptotic Sig.(2-sided test)	,995

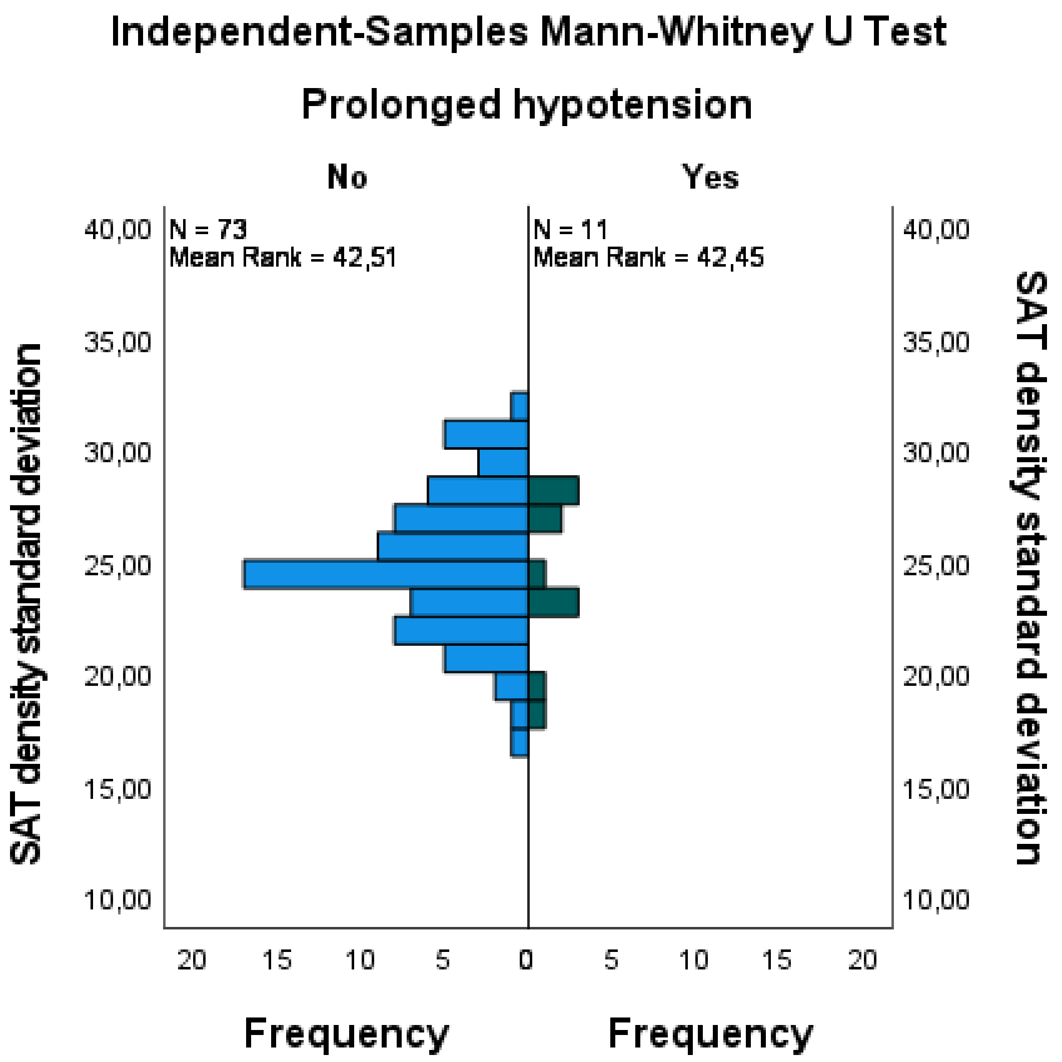

VAT mean density (HU) across Prolonged hypotension

Independent-Samples Mann-Whitney U Test Summary

Total N	85
Mann-Whitney U	464,000
Wilcoxon W	530,000
Test Statistic	464,000
Standard Error	76,365
Standardized Test Statistic	,746
Asymptotic Sig.(2-sided test)	,455

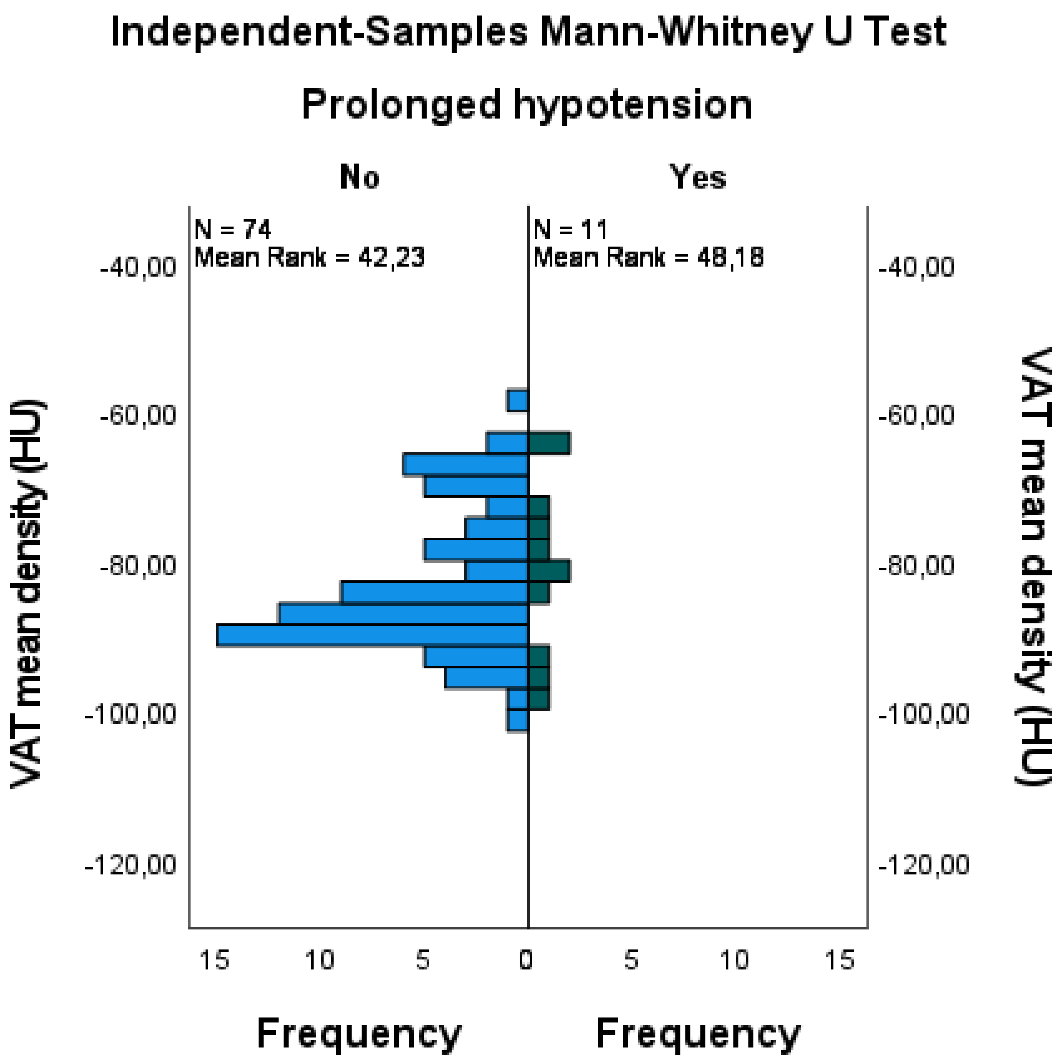

VAT median density (HU) across Prolonged hypotension

Independent-Samples Mann-Whitney U Test Summary

Total N	85
Mann-Whitney U	446,000
Wilcoxon W	512,000
Test Statistic	446,000
Standard Error	76,283
Standardized Test Statistic	,511
Asymptotic Sig.(2-sided test)	,609

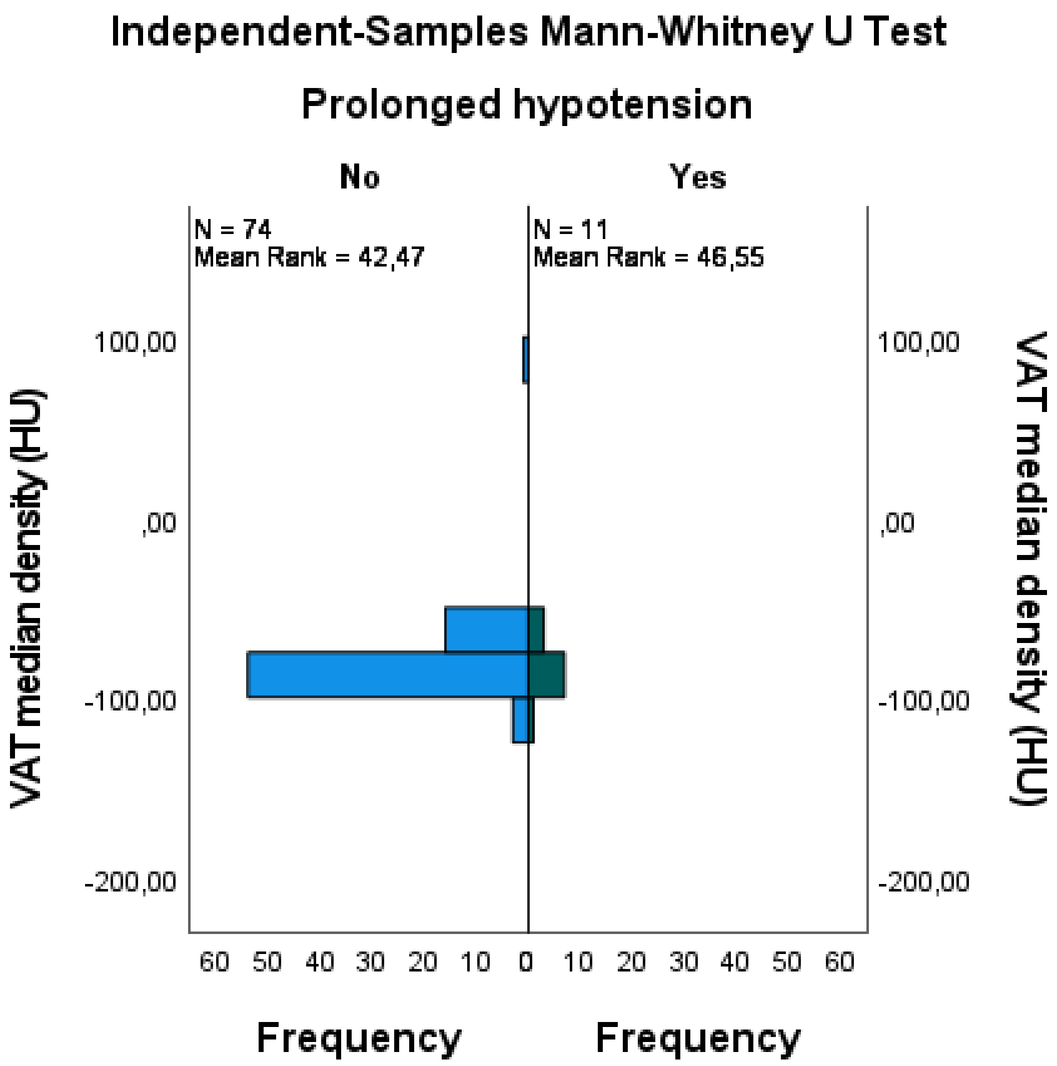

VAT density standard deviation across Prolonged hypotension

Independent-Samples Mann-Whitney U Test Summary

Total N	84
Mann-Whitney U	385,000
Wilcoxon W	451,000
Test Statistic	385,000
Standard Error	75,418
Standardized Test Statistic	-,219
Asymptotic Sig.(2-sided test)	,827

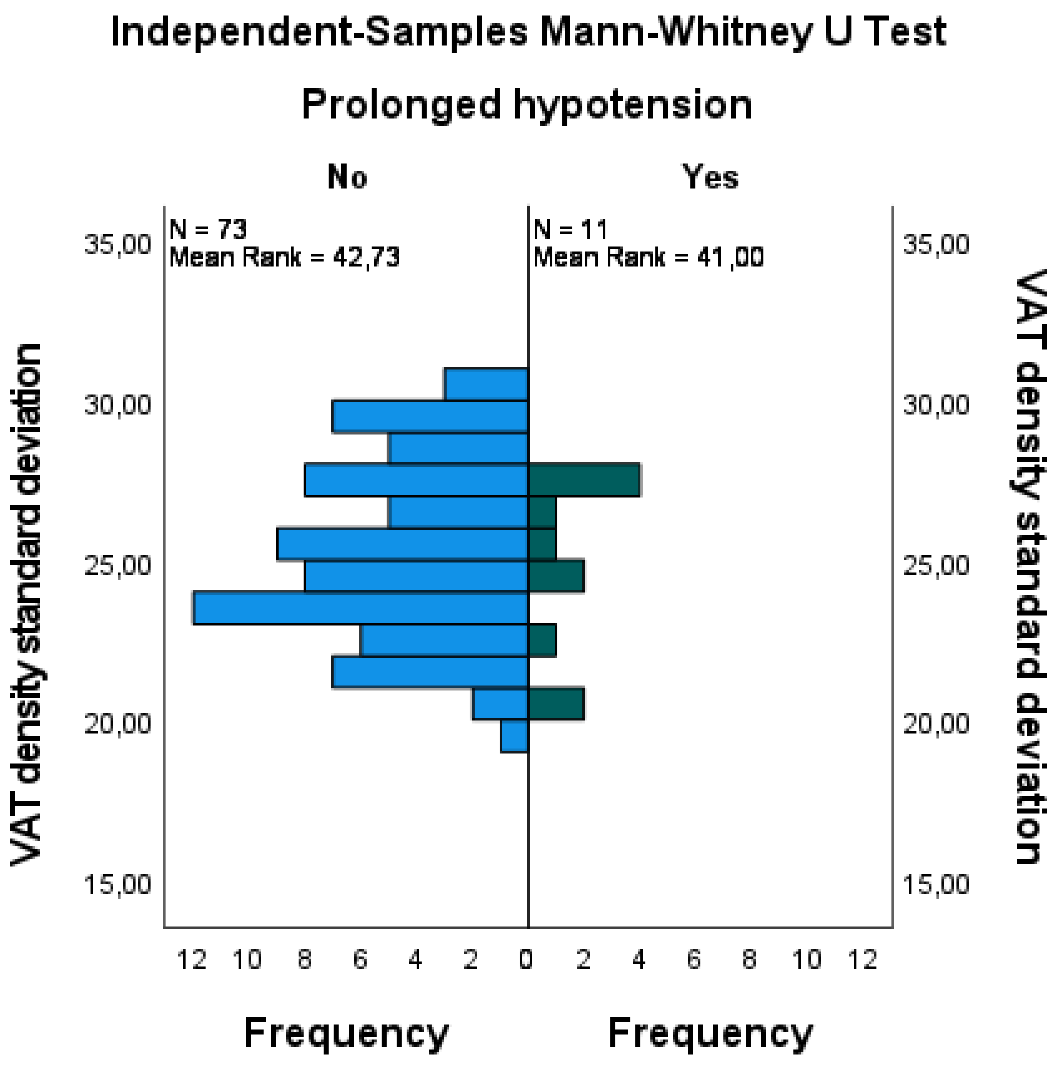

Right Psoas muscle mean density (HU) across Prolonged hypotension

Independent-Samples Mann-Whitney U Test Summary

Total N	85
Mann-Whitney U	346,000
Wilcoxon W	412,000
Test Statistic	346,000
Standard Error	76,362
Standardized Test Statistic	-,799
Asymptotic Sig.(2-sided test)	,424

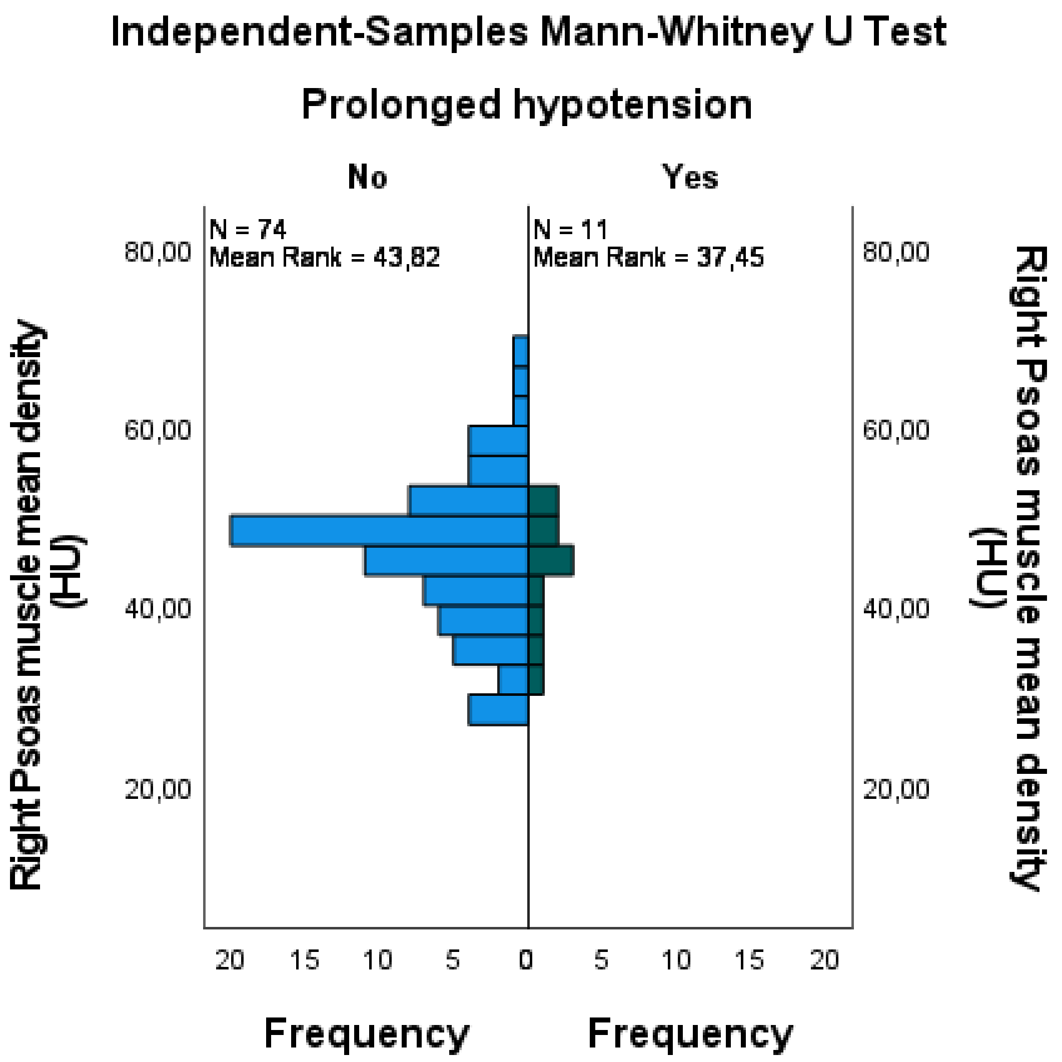

Right Psoas muscle median density (HU) across Prolonged hypotension

Independent-Samples Mann-Whitney U Test Summary

Total N	85
Mann-Whitney U	332,500
Wilcoxon W	398,500
Test Statistic	332,500
Standard Error	76,306
Standardized Test Statistic	-,976
Asymptotic Sig.(2-sided test)	,329

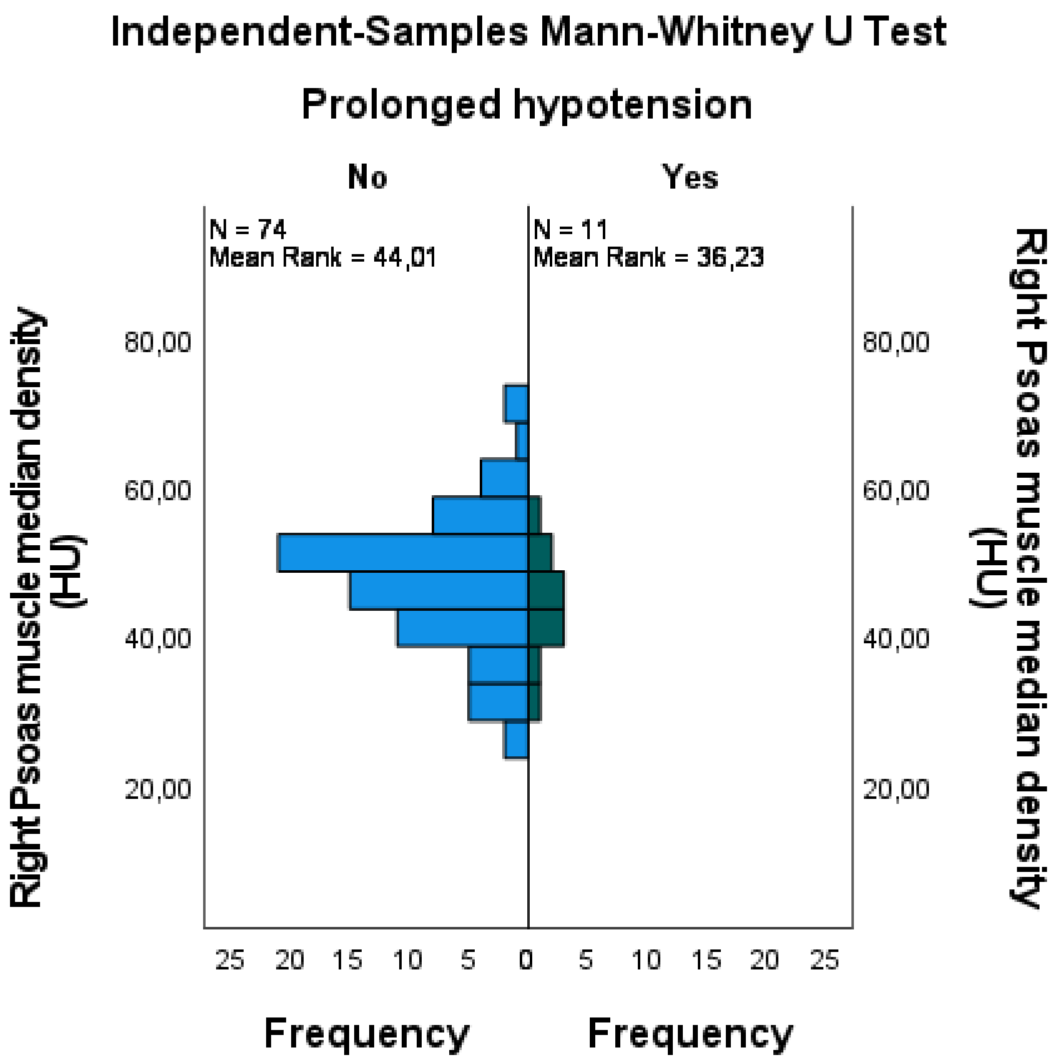

Right Psoas muscle density standard deviation across Prolonged hypotension

Independent-Samples Mann-Whitney U Test Summary

Total N	85
Mann-Whitney U	427,500
Wilcoxon W	493,500
Test Statistic	427,500
Standard Error	76,377
Standardized Test Statistic	,268
Asymptotic Sig.(2-sided test)	,788

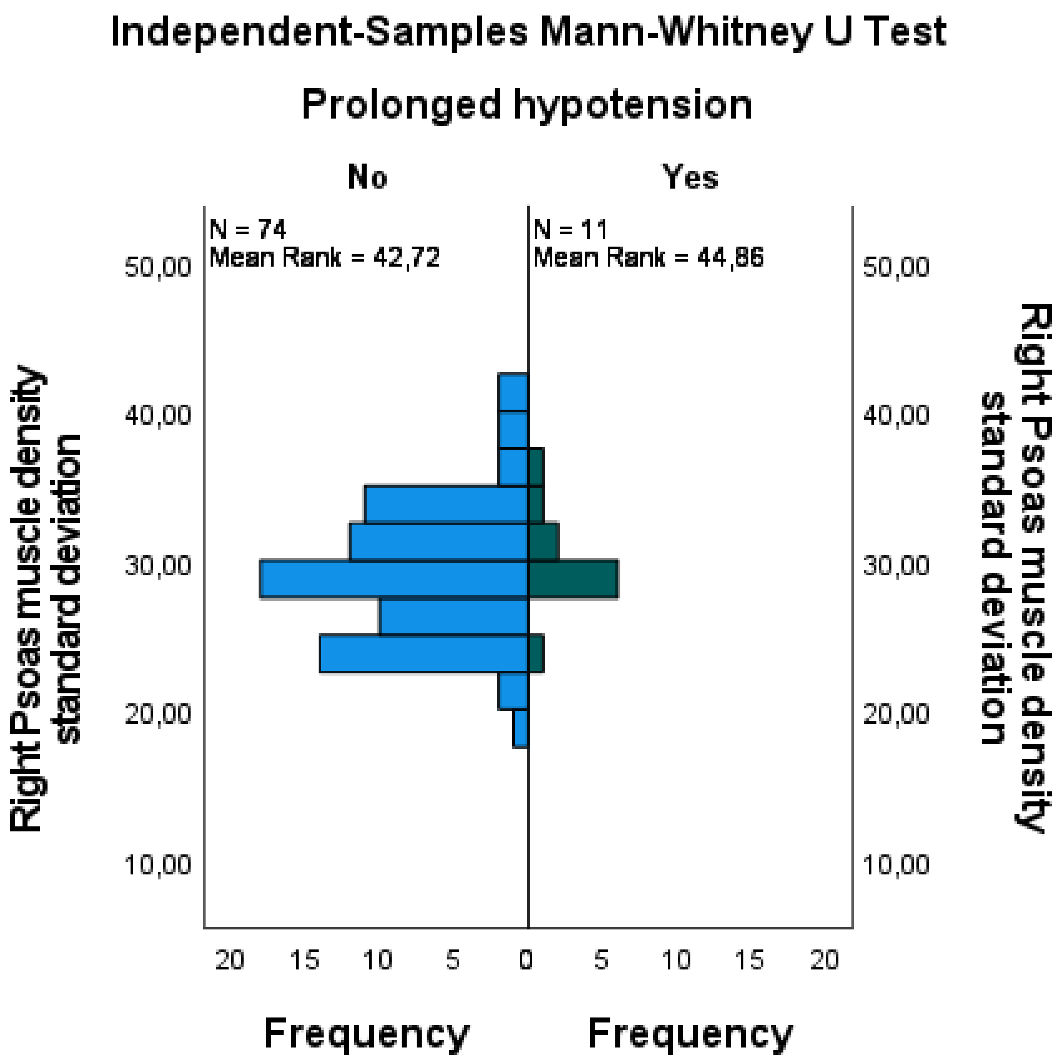

Left Psoas muscle mean density (HU) across Prolonged hypotension

Independent-Samples Mann-Whitney U Test Summary

Total N	85
Mann-Whitney U	389,000
Wilcoxon W	455,000
Test Statistic	389,000
Standard Error	76,362
Standardized Test Statistic	-,236
Asymptotic Sig.(2-sided test)	,814

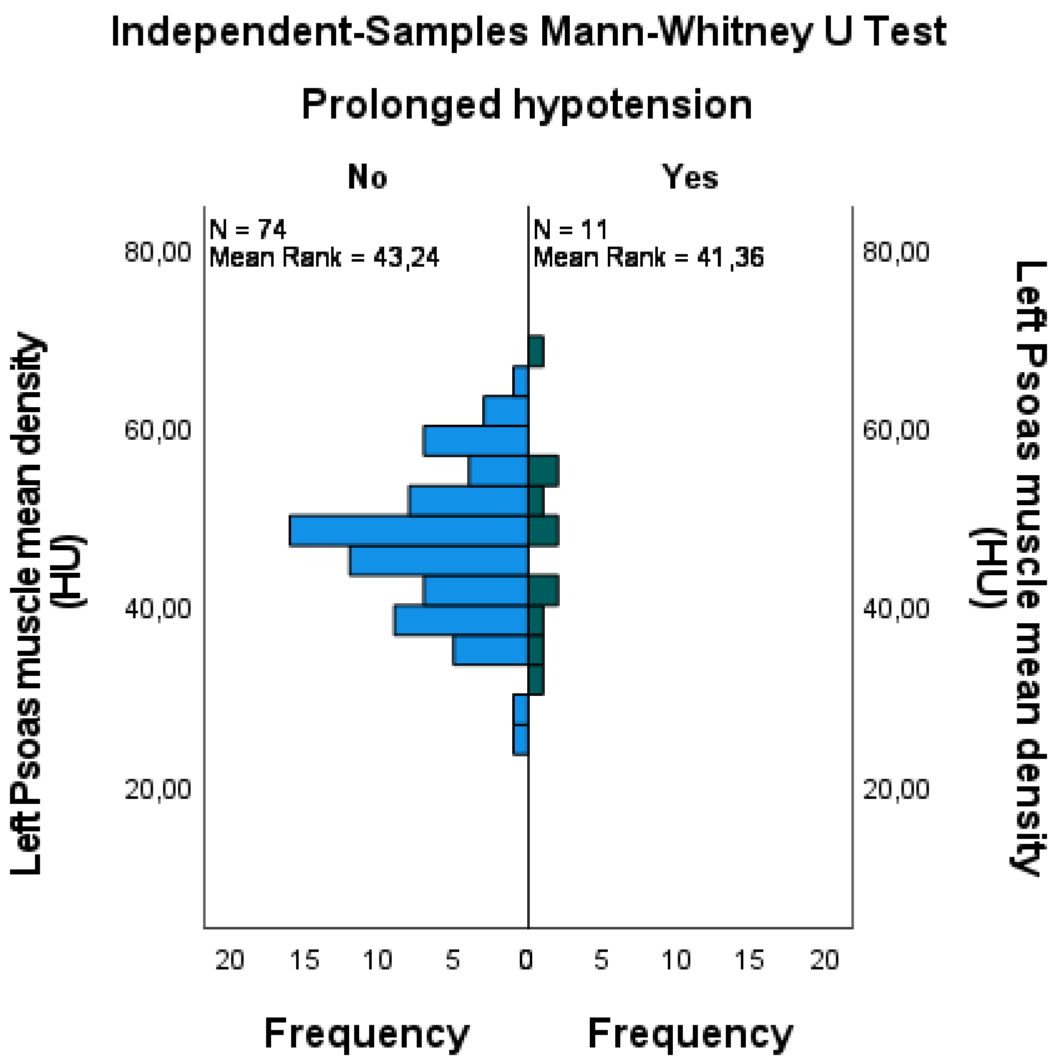

Left Psoas muscle median density (HU) across Prolonged hypotension

Independent-Samples Mann-Whitney U Test Summary

Total N	85
Mann-Whitney U	363,000
Wilcoxon W	429,000
Test Statistic	363,000
Standard Error	76,281
Standardized Test Statistic	-,577
Asymptotic Sig.(2-sided test)	,564

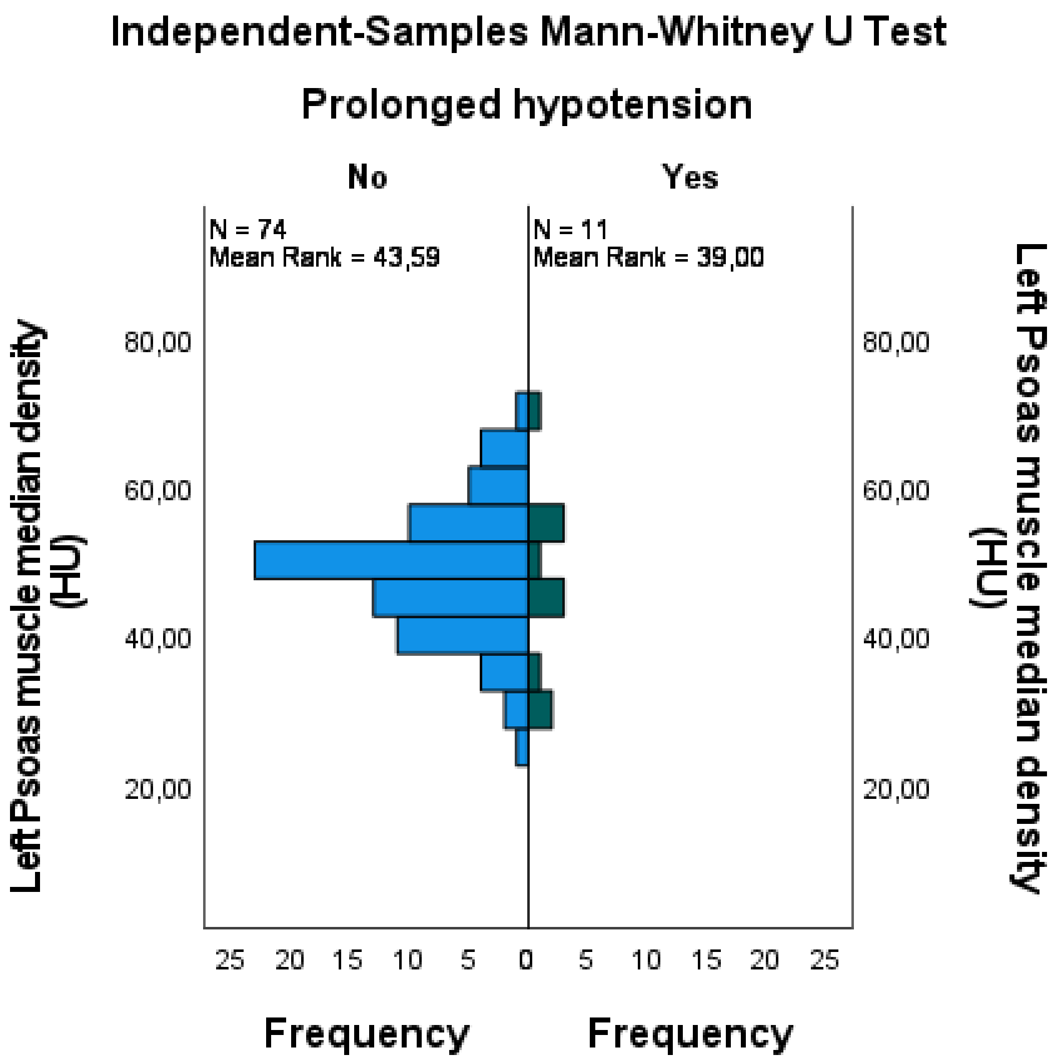

Left Psoas muscle density standard deviation across Prolonged hypotension

Independent-Samples Mann-Whitney U Test Summary

Total N	85
Mann-Whitney U	402,000
Wilcoxon W	468,000
Test Statistic	402,000
Standard Error	76,378
Standardized Test Statistic	-,065
Asymptotic Sig.(2-sided test)	,948

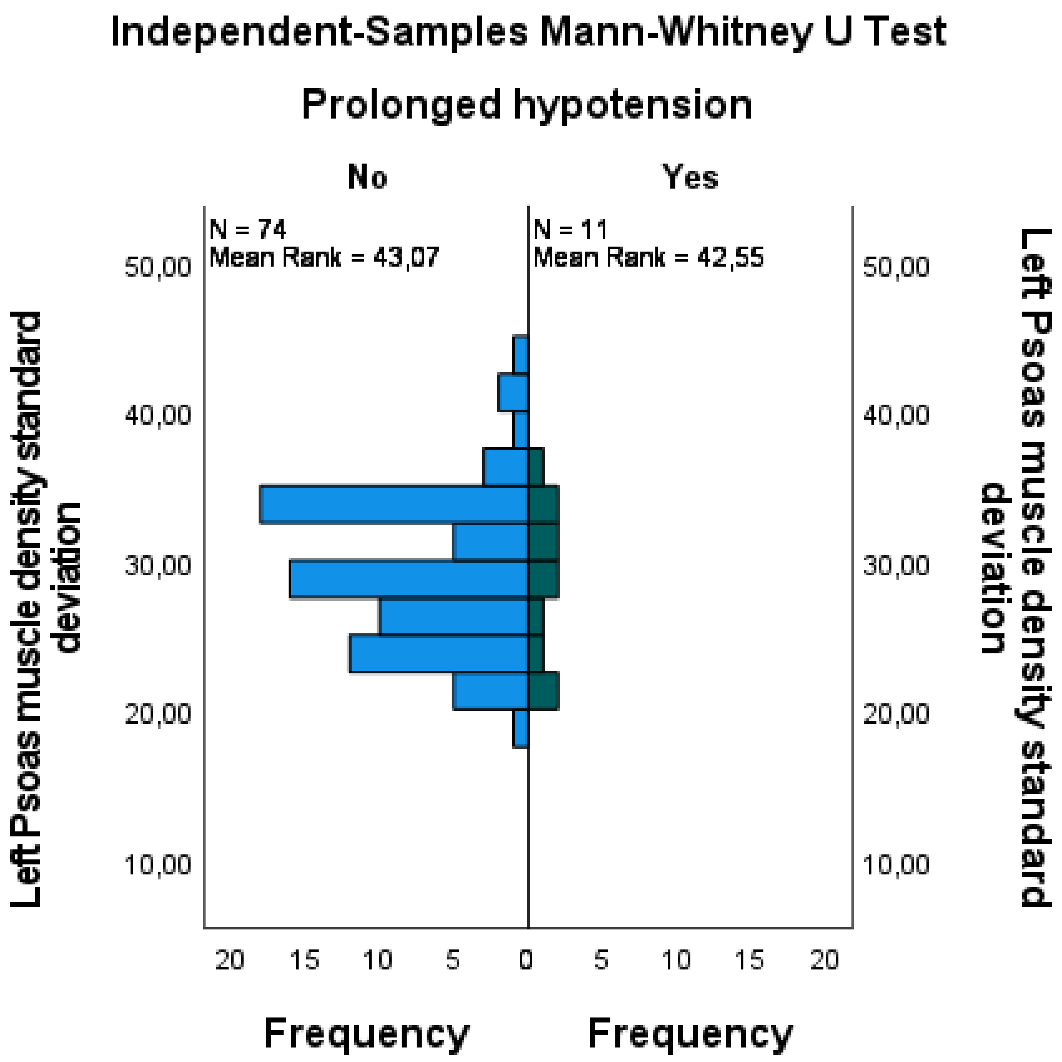

Independent-Samples Mann-Whitney U Test for bundle branch block occurrence

Hypothesis Test Summary

	Null Hypothesis	Test	Sig.[a,b]	Decision
1	The distribution of Psoas/height is the same across categories of Bundle branch block.	Independent-Samples Mann-Whitney U Test	,705	Retain the null hypothesis.

2	The distribution of Anterior SAT distance is the same across categories of Bundle branch block.	Independent-Samples Mann-Whitney U Test	,207	Retain the null hypothesis.
3	The distribution of Posterior SAT distance is the same across categories of Bundle branch block.	Independent-Samples Mann-Whitney U Test	,813	Retain the null hypothesis.
4	The distribution of Anterior+Posterior SAT distance is the same across categories of Bundle branch block.	Independent-Samples Mann-Whitney U Test	,538	Retain the null hypothesis.
5	The distribution of VAT distance is the same across categories of Bundle branch block.	Independent-Samples Mann-Whitney U Test	,595	Retain the null hypothesis.
6	The distribution of Right common femoral artery area (mm2) is the same across categories of Bundle branch block.	Independent-Samples Mann-Whitney U Test	,740	Retain the null hypothesis.
7	The distribution of Left common femoral artery area (mm2) is the same across categories of Bundle branch block.	Independent-Samples Mann-Whitney U Test	,384	Retain the null hypothesis.
8	The distribution of FAT area (cm2) is the same across categories of Bundle branch block.	Independent-Samples Mann-Whitney U Test	,892	Retain the null hypothesis.
9	The distribution of SAT area (cm2) is the same across categories of Bundle branch block.	Independent-Samples Mann-Whitney U Test	,473	Retain the null hypothesis.
10	The distribution of VAT area (cm2) is the same across categories of Bundle branch block.	Independent-Samples Mann-Whitney U Test	,477	Retain the null hypothesis.
11	The distribution of Right Psoas muscle area (cm2) is the same across categories of Bundle branch block.	Independent-Samples Mann-Whitney U Test	,995	Retain the null hypothesis.

12	The distribution of Left Psoas muscle area (cm2) is the same across categories of Bundle branch block.	Independent-Samples Mann-Whitney U Test	,763	Retain the null hypothesis.
13	The distribution of FAT mean density (HU) is the same across categories of Bundle branch block.	Independent-Samples Mann-Whitney U Test	,445	Retain the null hypothesis.
14	The distribution of FAT median density (HU) is the same across categories of Bundle branch block.	Independent-Samples Mann-Whitney U Test	,518	Retain the null hypothesis.
15	The distribution of FAT density standard deviation is the same across categories of Bundle branch block.	Independent-Samples Mann-Whitney U Test	,670	Retain the null hypothesis.
16	The distribution of SAT mean density (HU) is the same across categories of Bundle branch block.	Independent-Samples Mann-Whitney U Test	,431	Retain the null hypothesis.
17	The distribution of SAT median density (HU) is the same across categories of Bundle branch block.	Independent-Samples Mann-Whitney U Test	,789	Retain the null hypothesis.
18	The distribution of SAT density standard deviation is the same across categories of Bundle branch block.	Independent-Samples Mann-Whitney U Test	,259	Retain the null hypothesis.
19	The distribution of VAT mean density (HU) is the same across categories of Bundle branch block.	Independent-Samples Mann-Whitney U Test	,631	Retain the null hypothesis.
20	The distribution of VAT median density (HU) is the same across categories of Bundle branch block.	Independent-Samples Mann-Whitney U Test	,506	Retain the null hypothesis.
21	The distribution of VAT density standard deviation is the same across categories of Bundle branch block.	Independent-Samples Mann-Whitney U Test	,291	Retain the null hypothesis.

22	The distribution of Right Psoas muscle mean density (HU) is the same across categories of Bundle branch block.	Independent-Samples Mann-Whitney U Test	,455	Retain the null hypothesis.
23	The distribution of Right Psoas muscle median density (HU) is the same across categories of Bundle branch block.	Independent-Samples Mann-Whitney U Test	,553	Retain the null hypothesis.
24	The distribution of Right Psoas muscle density standard deviation is the same across categories of Bundle branch block.	Independent-Samples Mann-Whitney U Test	,657	Retain the null hypothesis.
25	The distribution of Left Psoas muscle mean density (HU) is the same across categories of Bundle branch block.	Independent-Samples Mann-Whitney U Test	,526	Retain the null hypothesis.
26	The distribution of Left Psoas muscle median density (HU) is the same across categories of Bundle branch block.	Independent-Samples Mann-Whitney U Test	,390	Retain the null hypothesis.
27	The distribution of Left Psoas muscle density standard deviation is the same across categories of Bundle branch block.	Independent-Samples Mann-Whitney U Test	,507	Retain the null hypothesis.

a. The significance level is ,050.

b. Asymptotic significance is displayed.

In this case, the hypothesis of equal medians is accepted for all the variables ($p > 0.05$). (The tables and graphs below are the details of the tests in this table: I have highlighted what things you should eventually report, namely test statistic and pvalue).

Psoas/height across Bundle branch block

Independent-Samples Mann-Whitney U Test Summary

Total N	85
Mann-Whitney U	465,000
Wilcoxon W	570,000

Test Statistic	465,000
Standard Error	84,401
Standardized Test Statistic	-,379
Asymptotic Sig.(2-sided test)	,705

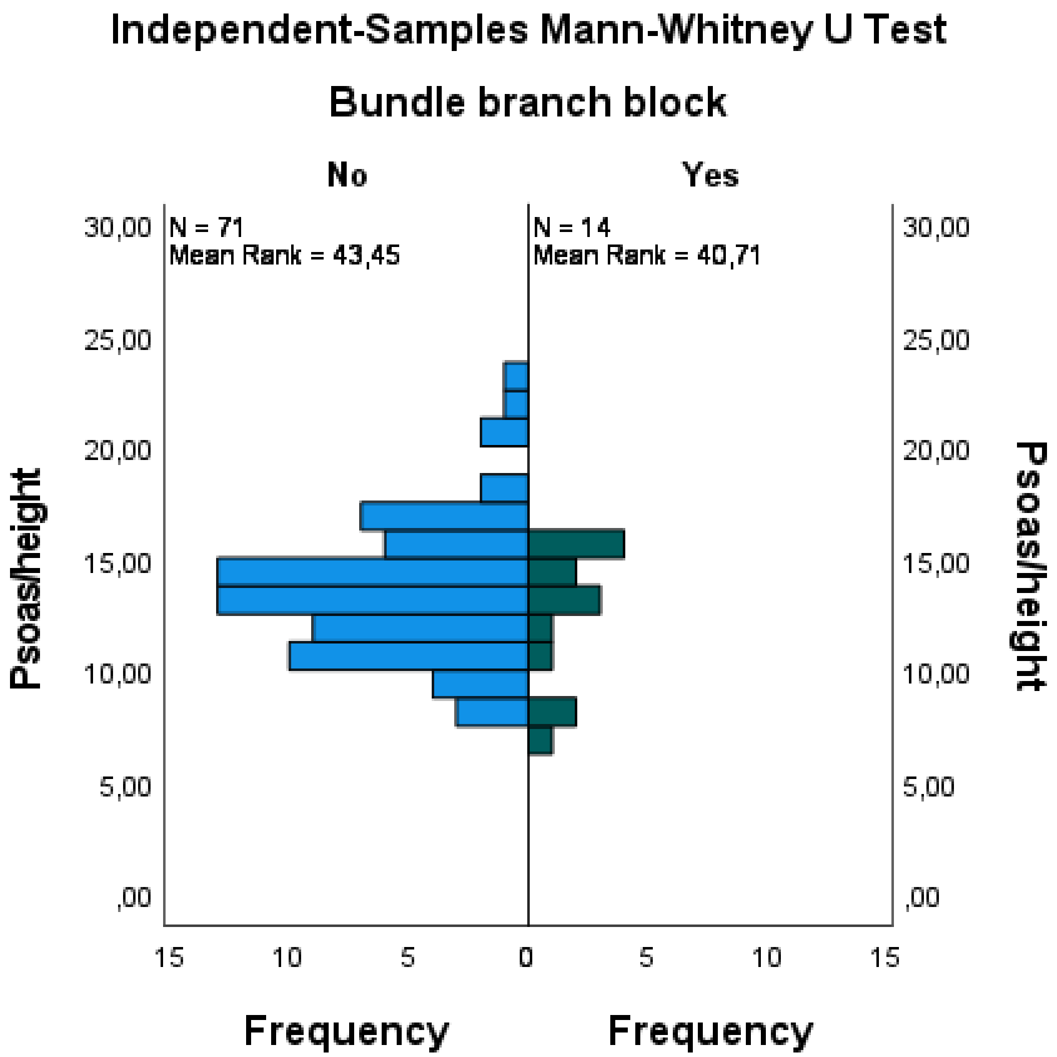

Anterior SAT distance across Bundle branch block

Independent-Samples Mann-Whitney U Test Summary

Total N	85
Mann-Whitney U	390,500
Wilcoxon W	495,500

Test Statistic	390,500
Standard Error	84,390
Standardized Test Statistic	-1,262
Asymptotic Sig.(2-sided test)	,207

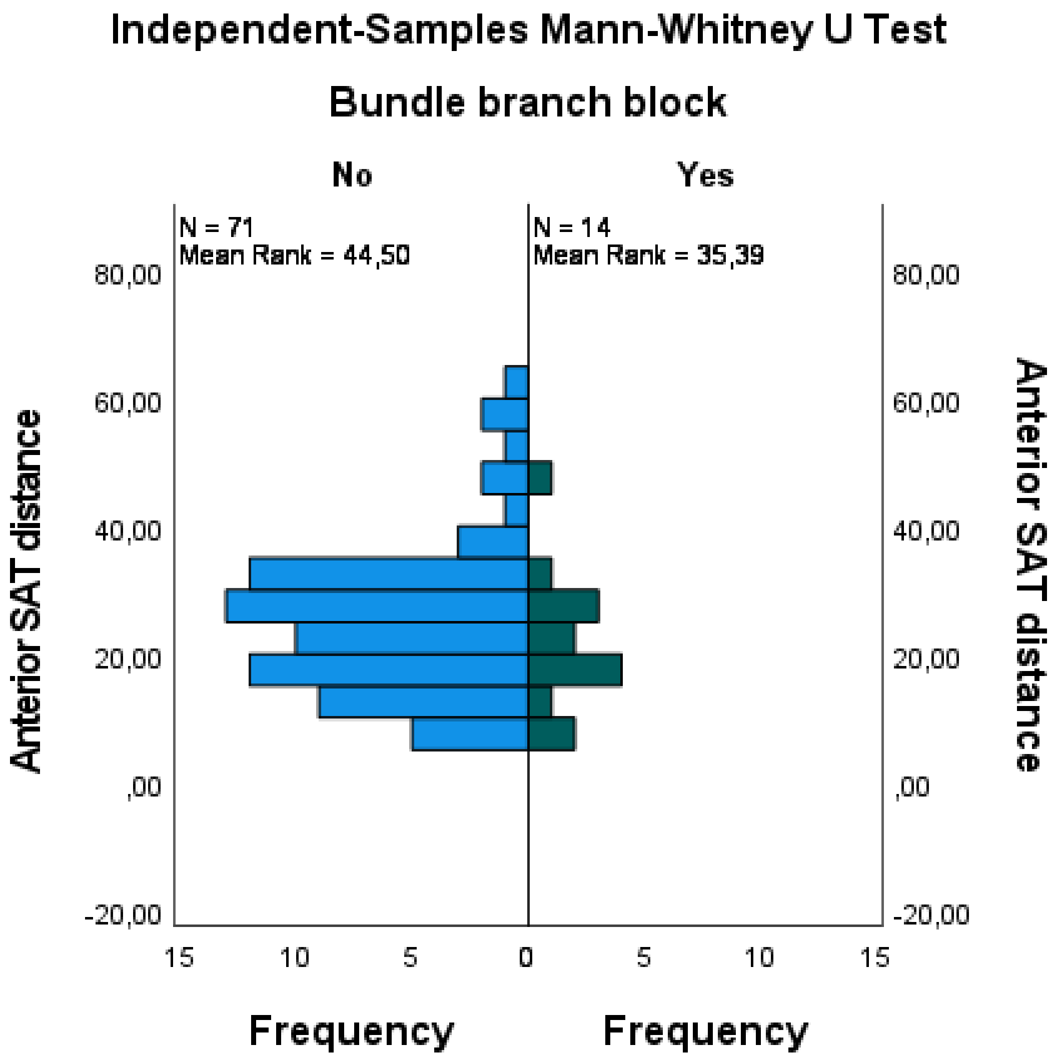

Posterior SAT distance across Bundle branch block

Independent-Samples Mann-Whitney U Test Summary

Total N	85
Mann-Whitney U	477,000
Wilcoxon W	582,000

Test Statistic	477,000
Standard Error	84,392
Standardized Test Statistic	-,237
Asymptotic Sig.(2-sided test)	,813

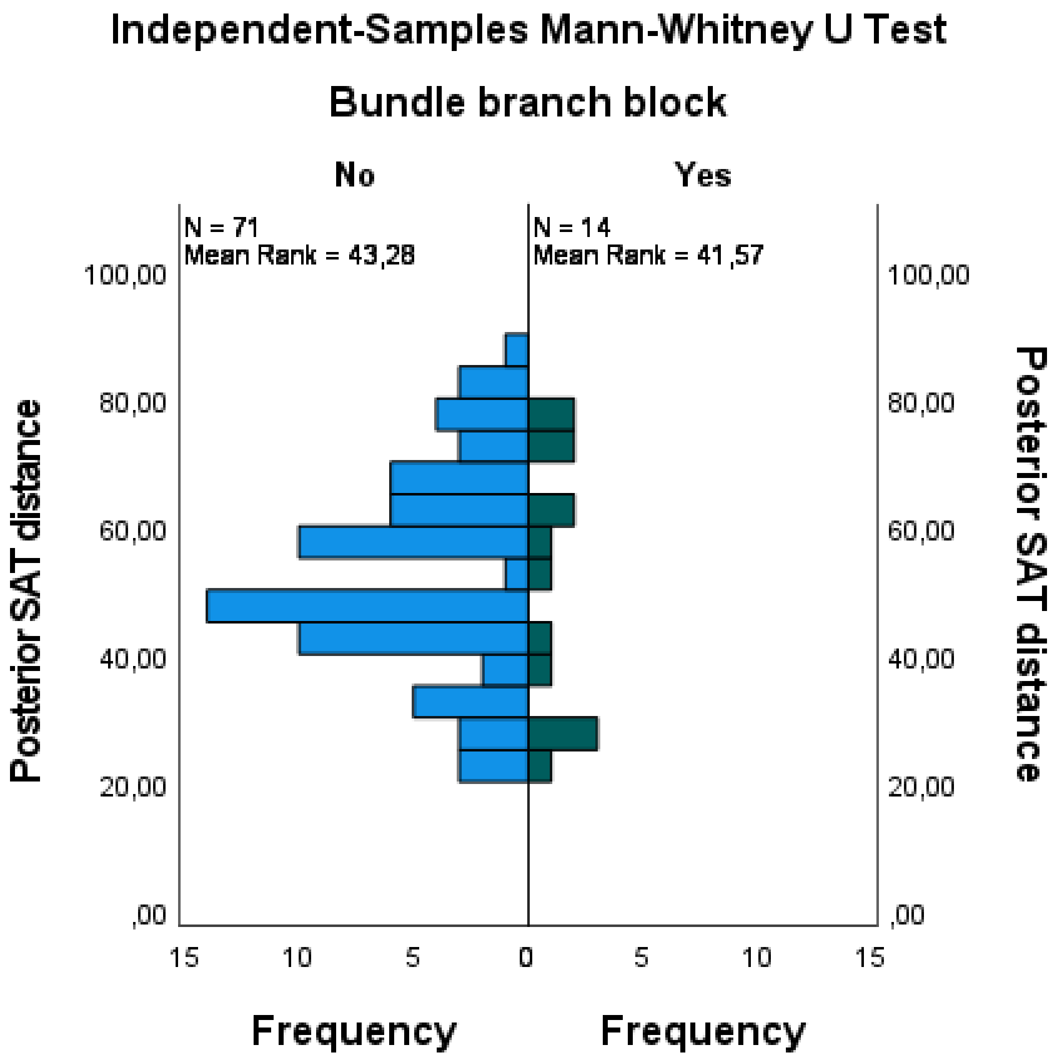

Anterior+Posterior SAT distance across Bundle branch block

Independent-Samples Mann-Whitney U Test Summary

Total N	85
Mann-Whitney U	445,000
Wilcoxon W	550,000

Test Statistic	445,000
Standard Error	84,398
Standardized Test Statistic	-,616
Asymptotic Sig.(2-sided test)	,538

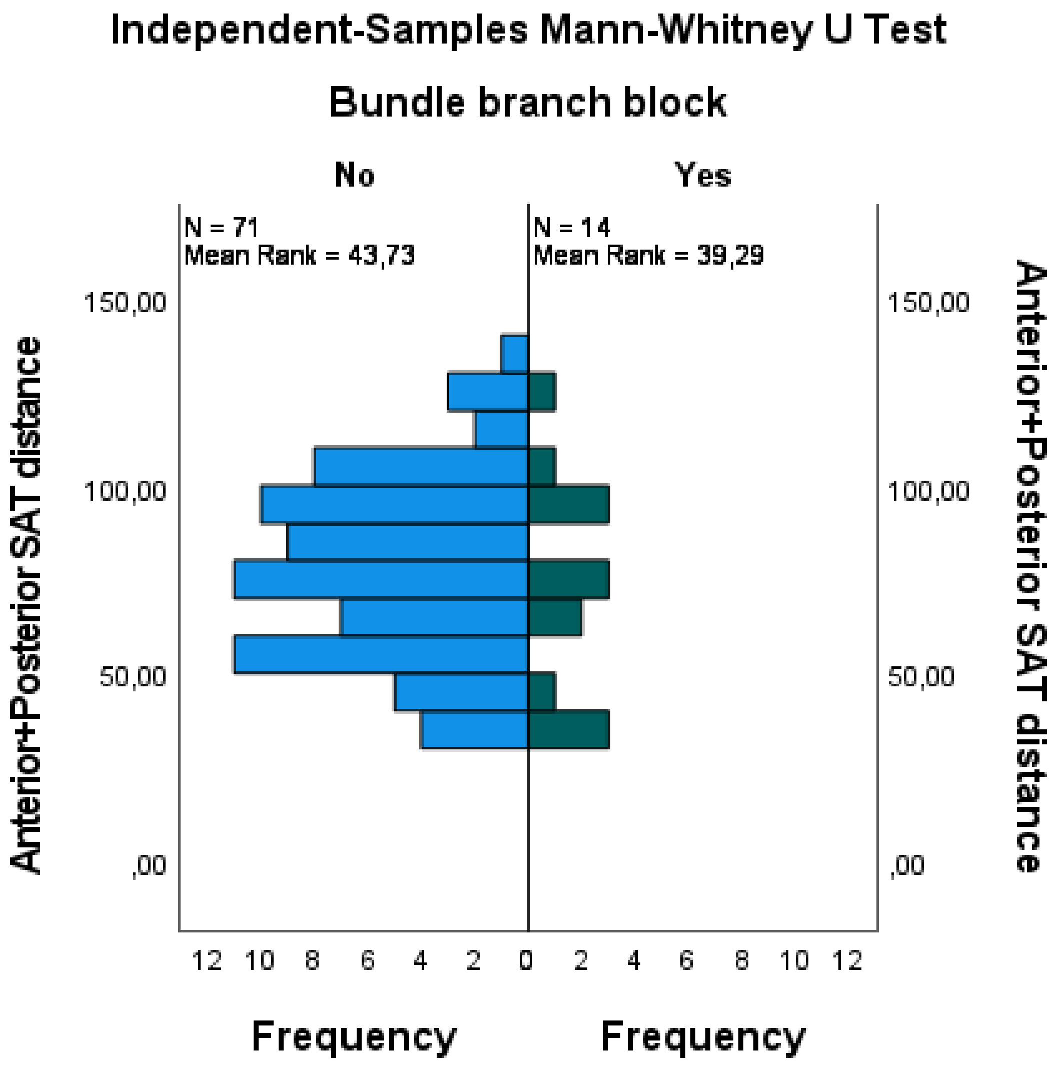

VAT distance across Bundle branch block

Independent-Samples Mann-Whitney U Test Summary

Total N	84
Mann-Whitney U	504,500
Wilcoxon W	595,500

Test Statistic	504,500
Standard Error	80,841
Standardized Test Statistic	,532
Asymptotic Sig.(2-sided test)	,595

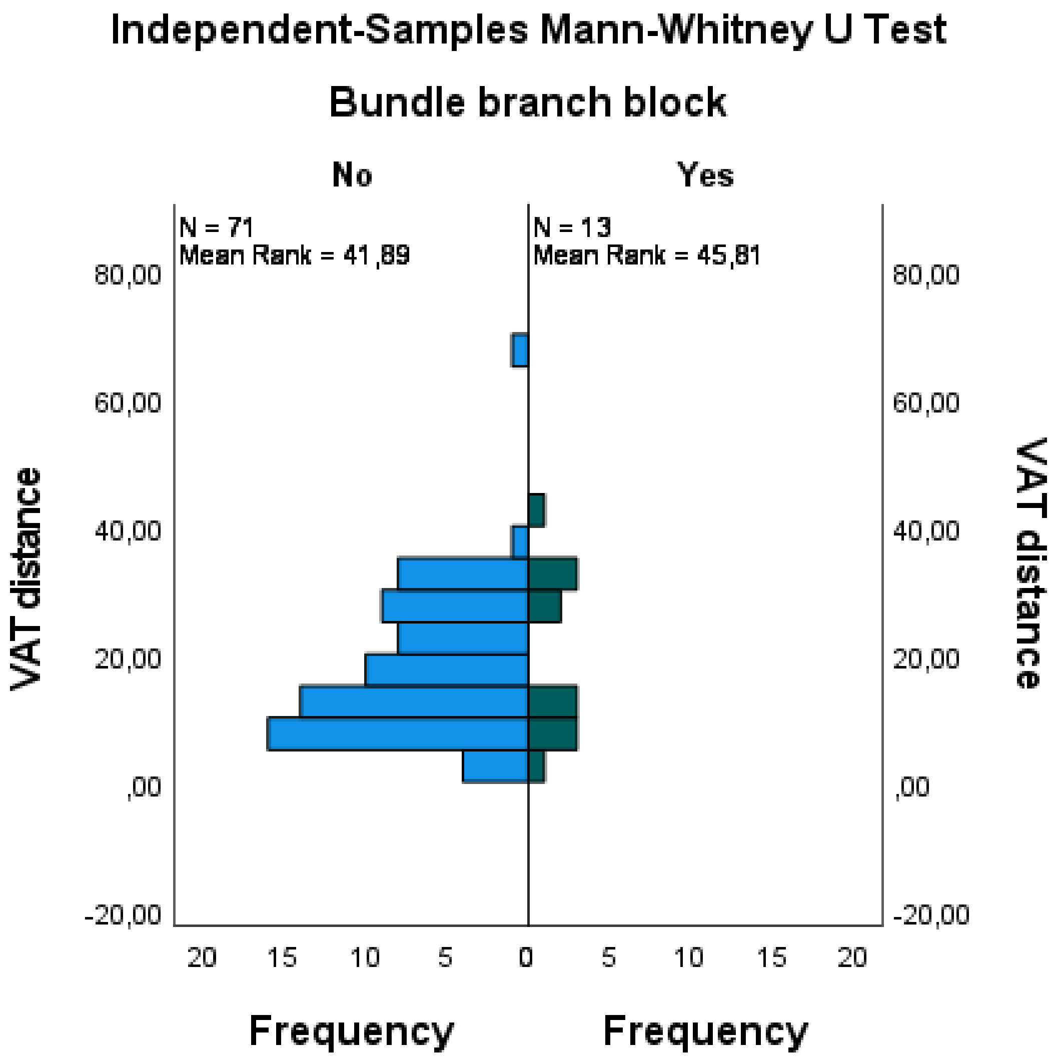

Right common femoral artery area (mm2) across Bundle branch block

Independent-Samples Mann-Whitney U Test Summary

Total N	85
Mann-Whitney U	469,000
Wilcoxon W	574,000

Test Statistic	469,000
Standard Error	84,388
Standardized Test Statistic	-,332
Asymptotic Sig.(2-sided test)	,740

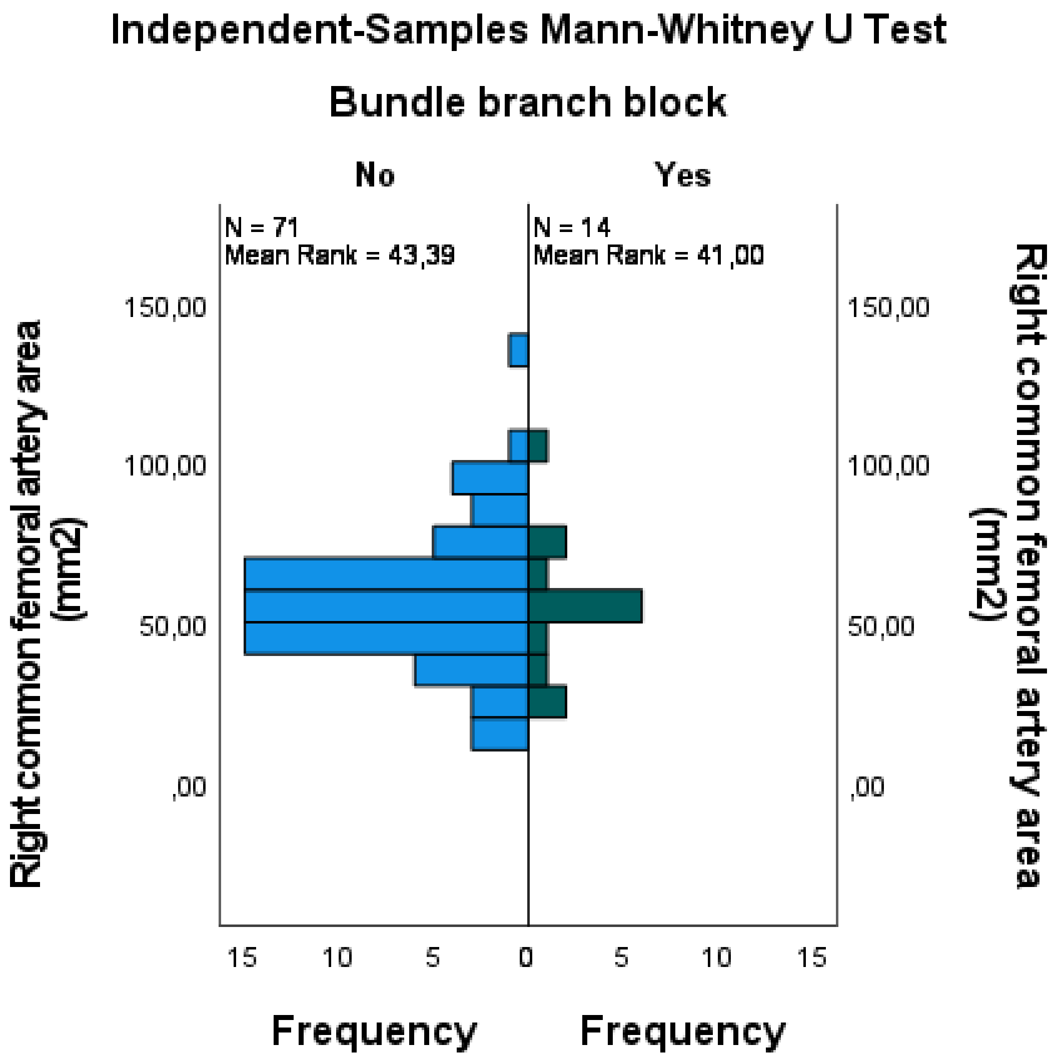

Left common femoral artery area (mm2) across Bundle branch block

Independent-Samples Mann-Whitney U Test Summary

Total N	85
Mann-Whitney U	423,500
Wilcoxon W	528,500

Test Statistic	423,500
Standard Error	84,381
Standardized Test Statistic	-,871
Asymptotic Sig.(2-sided test)	,384

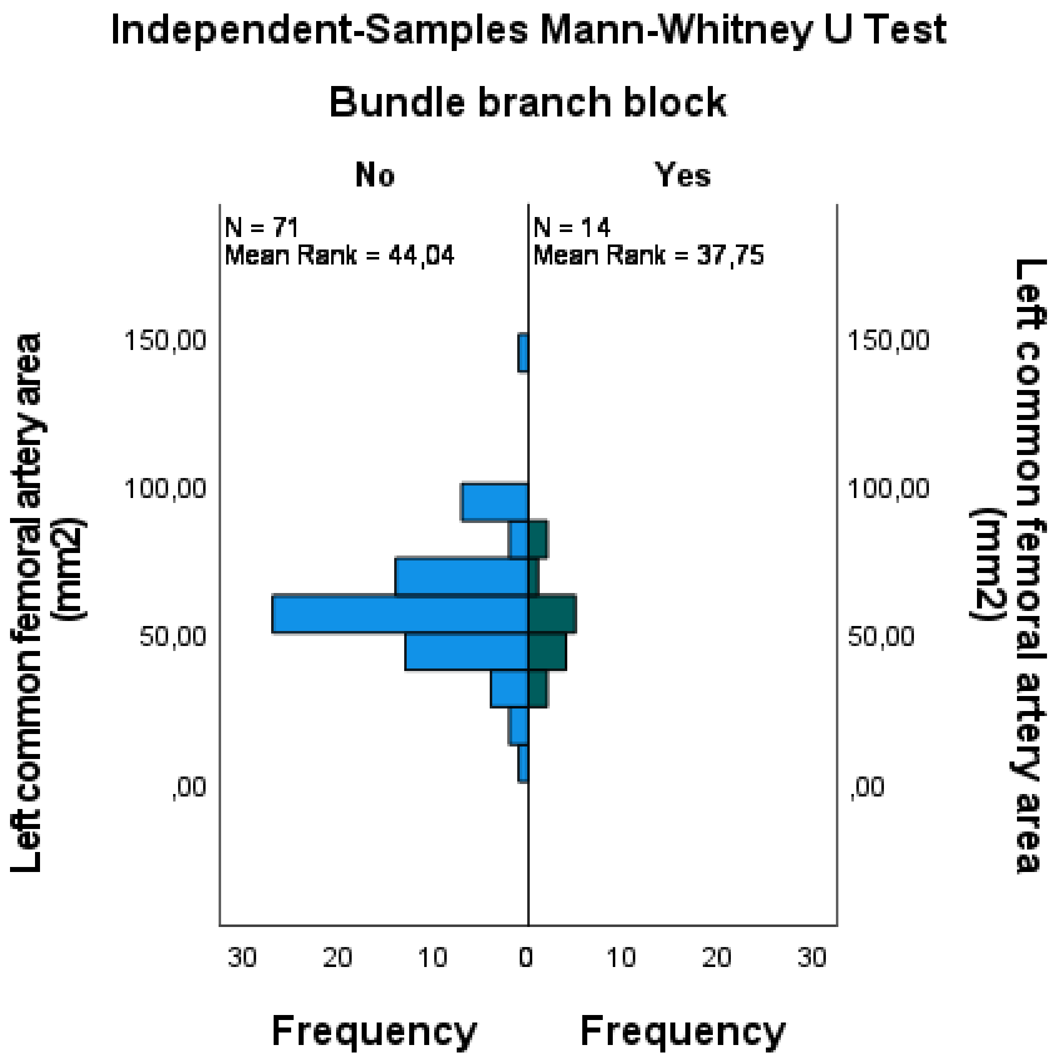

FAT area (cm2) across Bundle branch block

Independent-Samples Mann-Whitney U Test Summary

Total N	85
Mann-Whitney U	508,500
Wilcoxon W	613,500

Test Statistic	508,500
Standard Error	84,401
Standardized Test Statistic	,136
Asymptotic Sig.(2-sided test)	,892

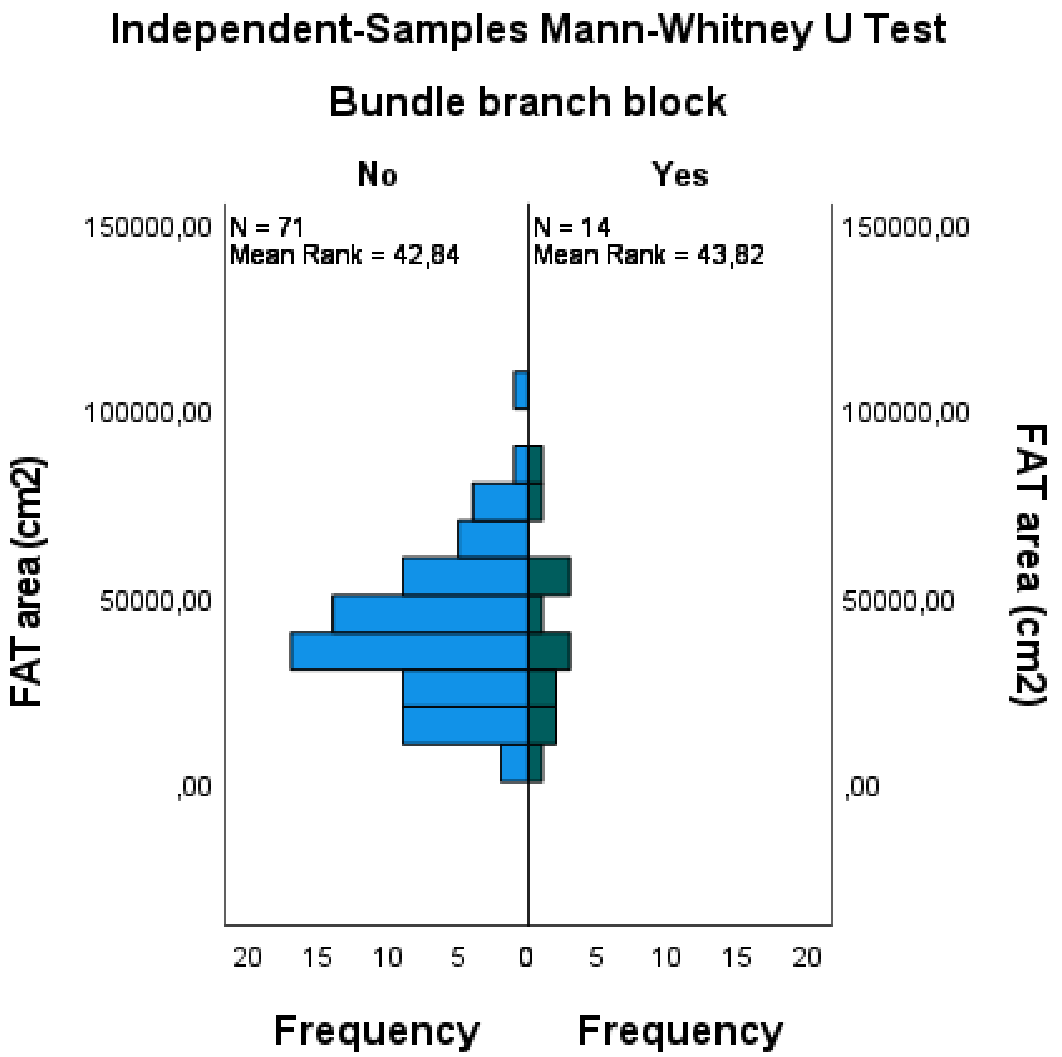

SAT area (cm2) across Bundle branch block

Independent-Samples Mann-Whitney U Test Summary

Total N	85
Mann-Whitney U	436,500
Wilcoxon W	541,500

Test Statistic	436,500
Standard Error	84,401
Standardized Test Statistic	-,717
Asymptotic Sig.(2-sided test)	,473

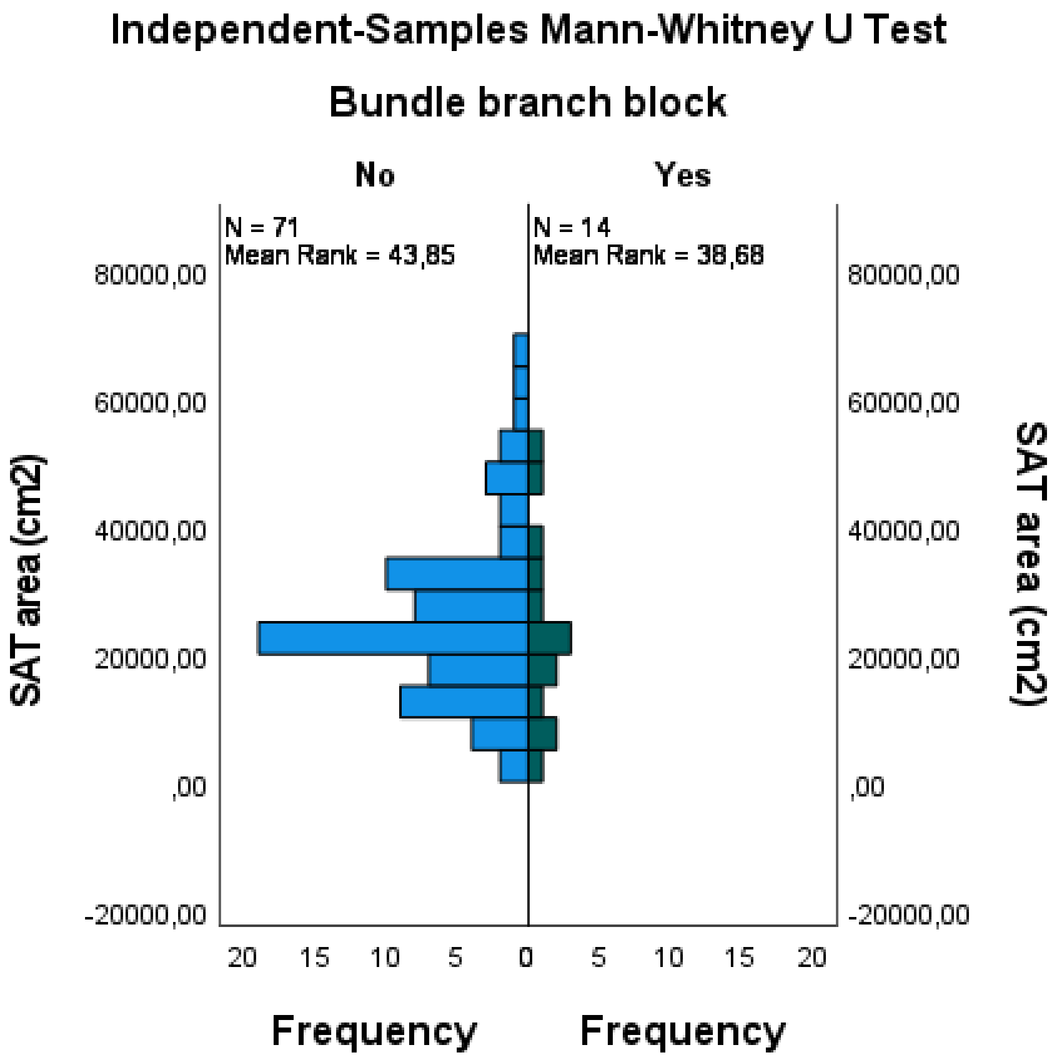

VAT area (cm2) across Bundle branch block

Independent-Samples Mann-Whitney U Test Summary

Total N	84
Mann-Whitney U	519,000
Wilcoxon W	610,000

Test Statistic	519,000
Standard Error	80,857
Standardized Test Statistic	,711
Asymptotic Sig.(2-sided test)	,477

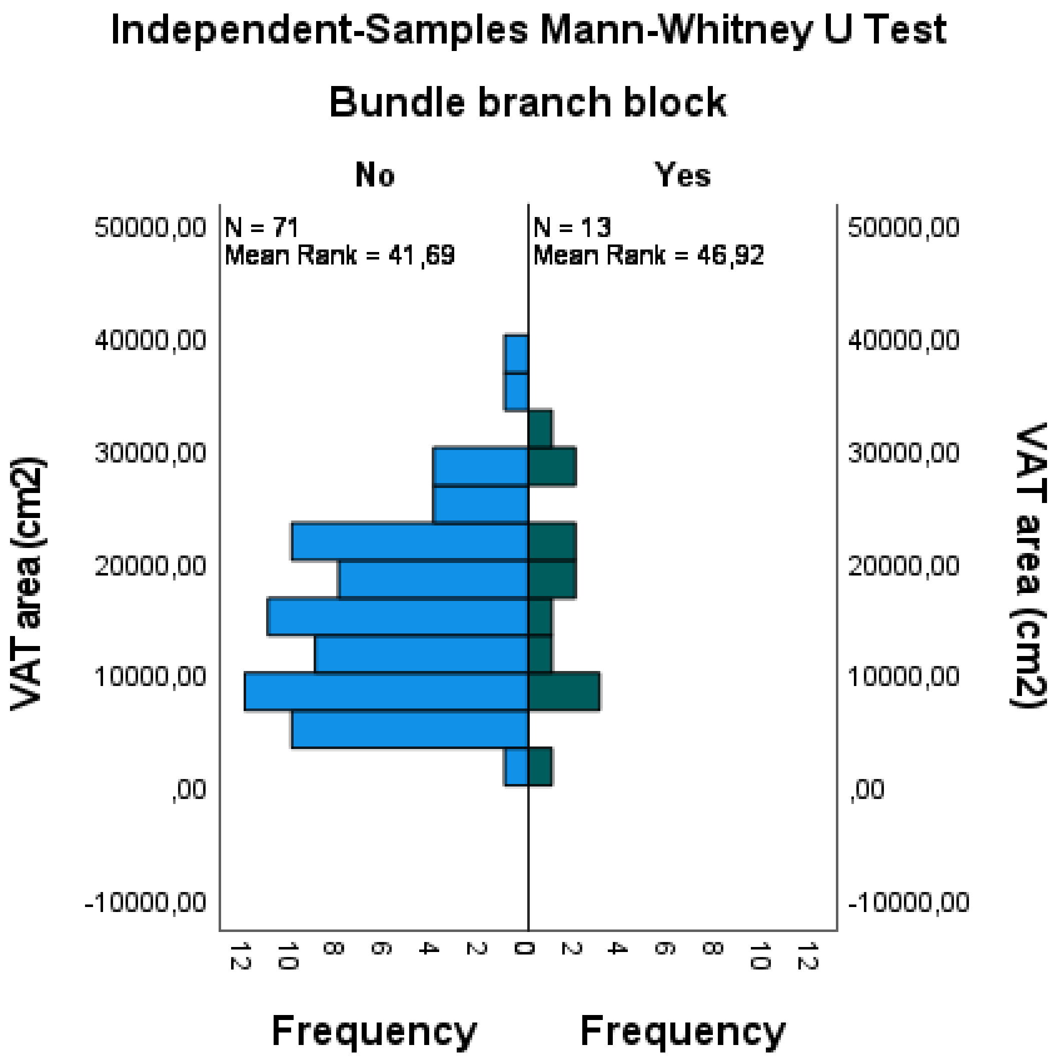

Right Psoas muscle area (cm2) across Bundle branch block

Independent-Samples Mann-Whitney U Test Summary

Total N	85
Mann-Whitney U	497,500
Wilcoxon W	602,500

Test Statistic	497,500
Standard Error	84,399
Standardized Test Statistic	,006
Asymptotic Sig.(2-sided test)	,995

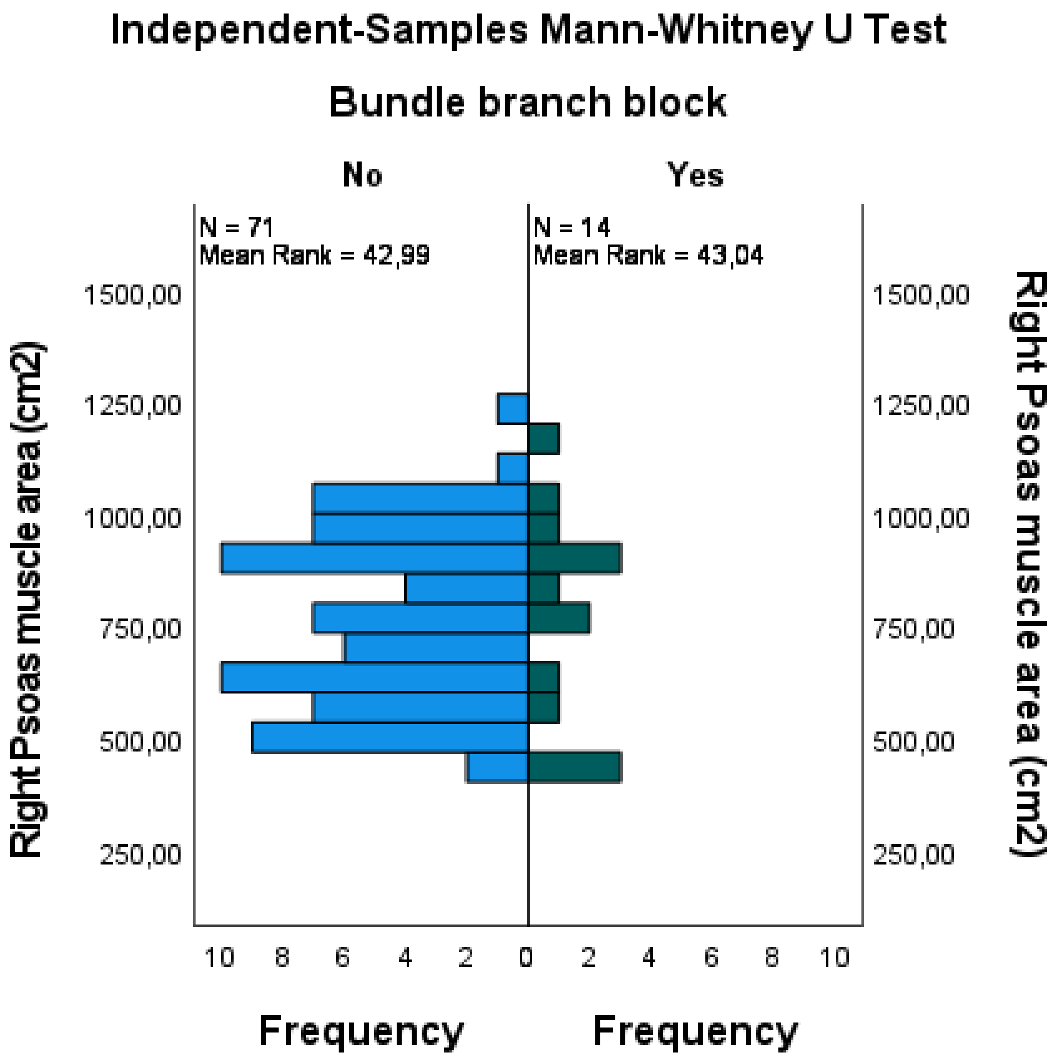

Left Psoas muscle area (cm2) across Bundle branch block

Independent-Samples Mann-Whitney U Test Summary

Total N	85
Mann-Whitney U	522,500
Wilcoxon W	627,500

Test Statistic	522,500
Standard Error	84,401
Standardized Test Statistic	,302
Asymptotic Sig.(2-sided test)	,763

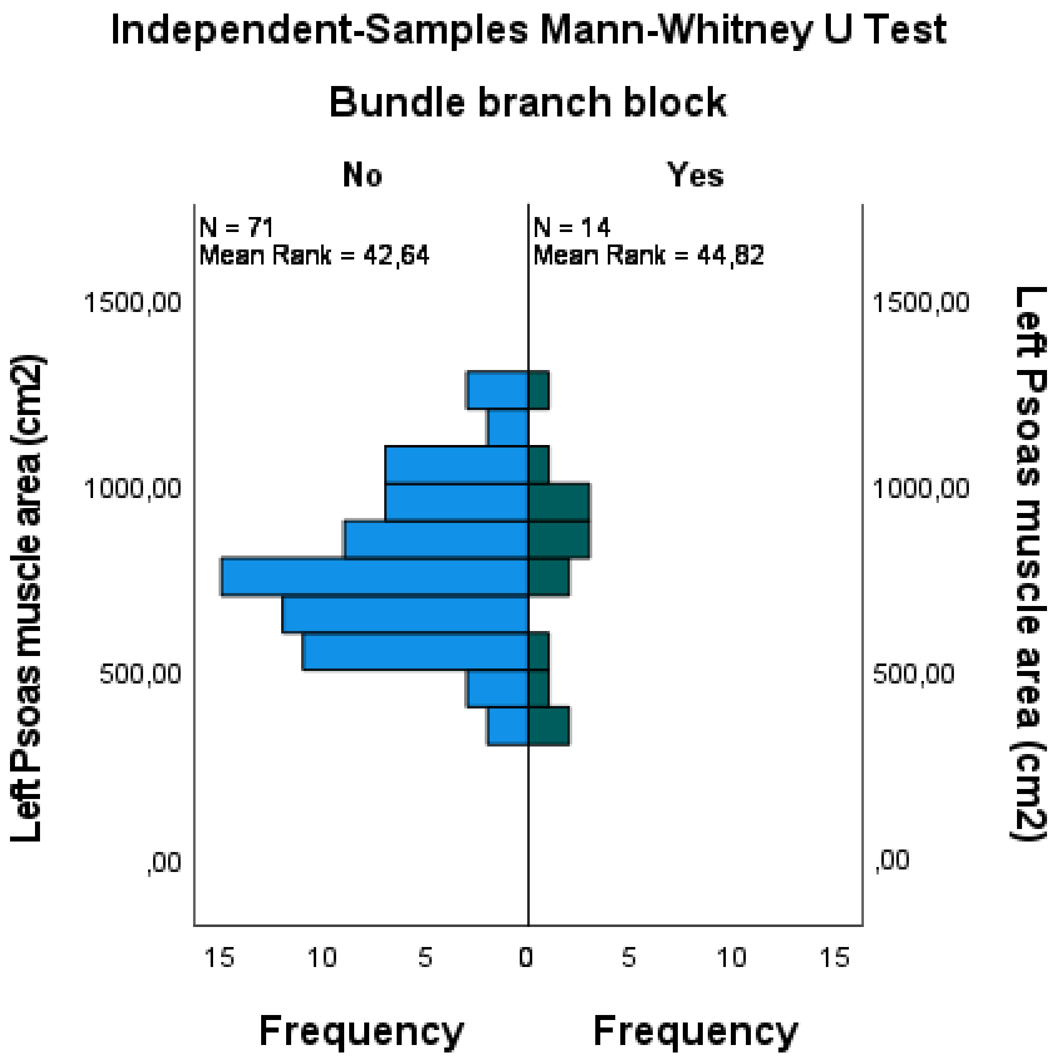

FAT mean density (HU) across Bundle branch block

Independent-Samples Mann-Whitney U Test Summary

Total N	85
Mann-Whitney U	561,500
Wilcoxon W	666,500

Test Statistic	561,500
Standard Error	84,388
Standardized Test Statistic	,764
Asymptotic Sig.(2-sided test)	,445

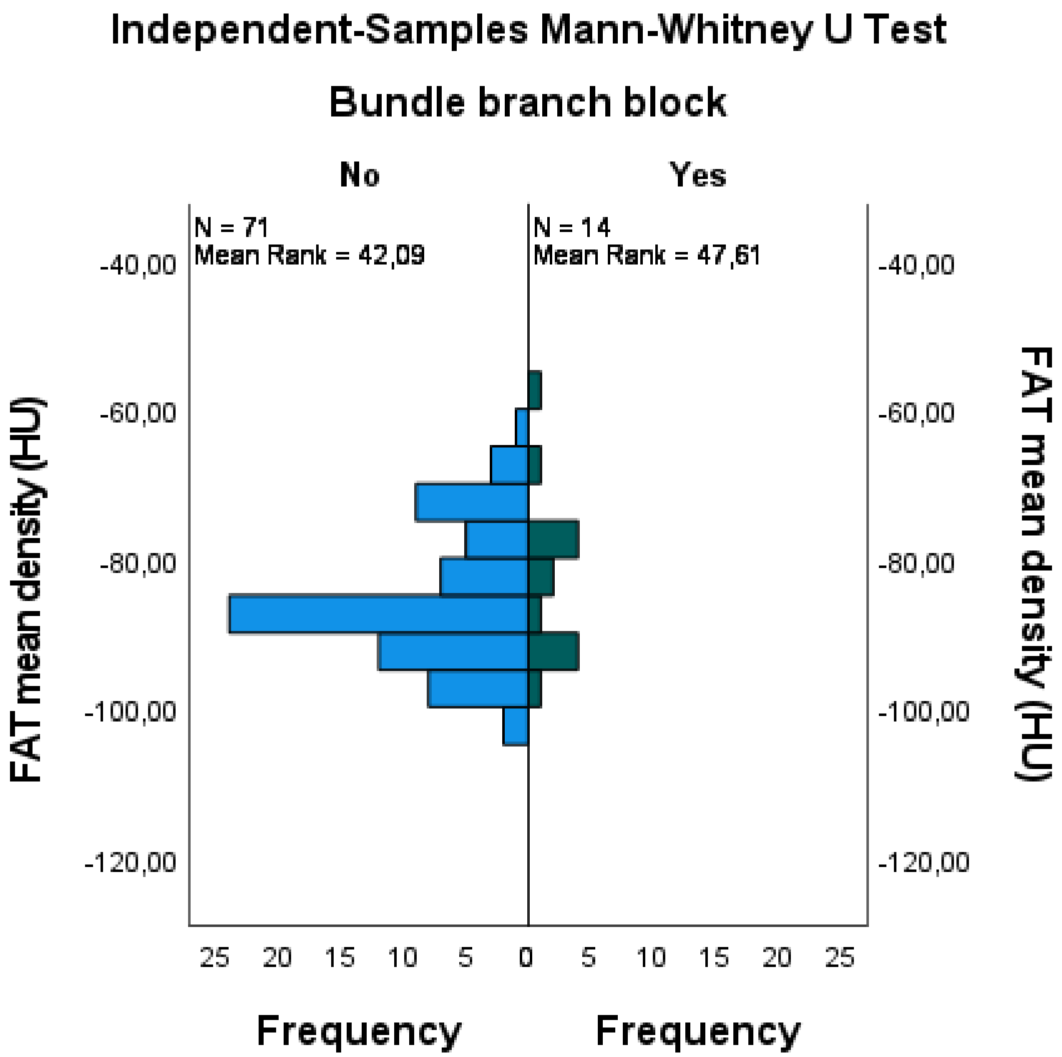

FAT median density (HU) across Bundle branch block

Independent-Samples Mann-Whitney U Test Summary

Total N	85
Mann-Whitney U	551,500
Wilcoxon W	656,500

Test Statistic	551,500
Standard Error	84,300
Standardized Test Statistic	,647
Asymptotic Sig.(2-sided test)	,518

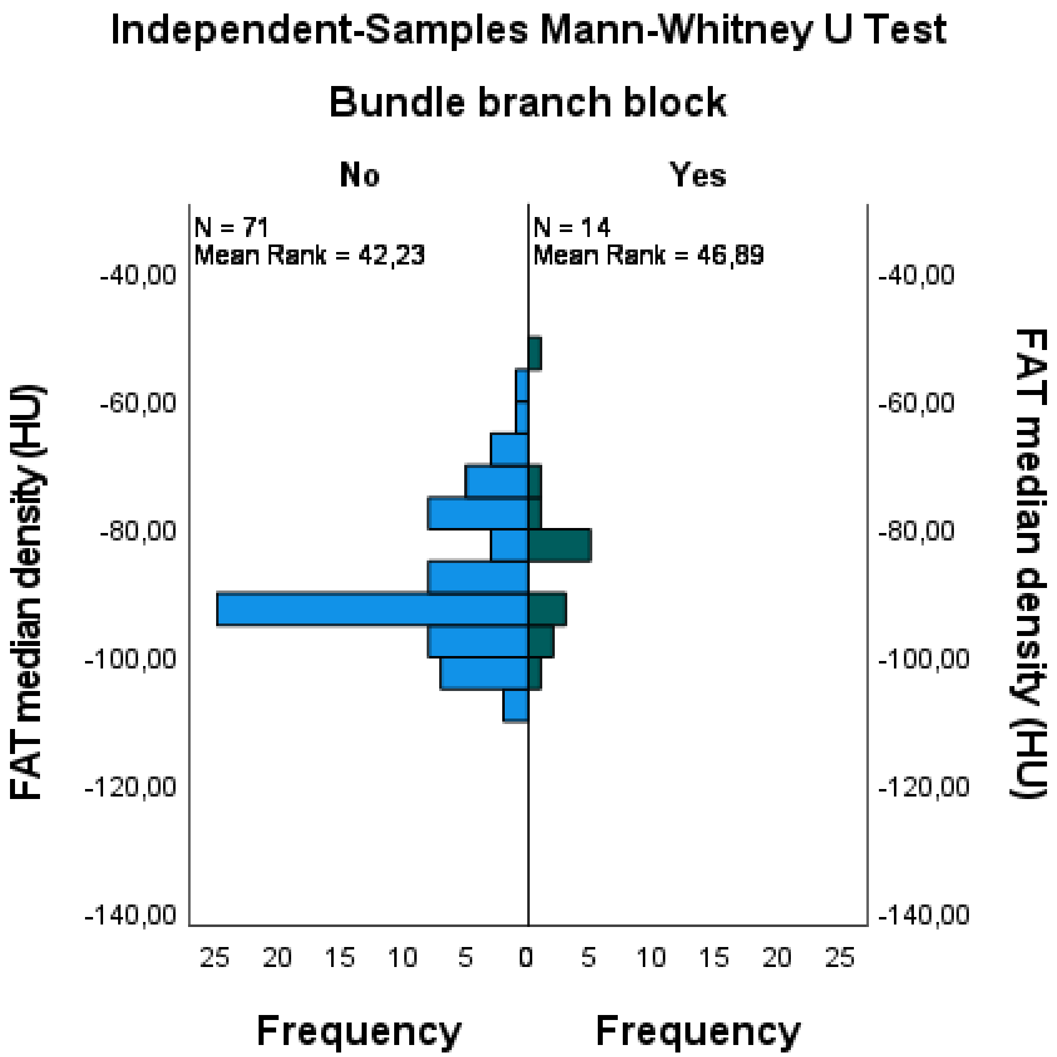

FAT density standard deviation across Bundle branch block

Independent-Samples Mann-Whitney U Test Summary

Total N	85
Mann-Whitney U	461,000
Wilcoxon W	566,000

Test Statistic	461,000
Standard Error	84,401
Standardized Test Statistic	-,427
Asymptotic Sig.(2-sided test)	,670

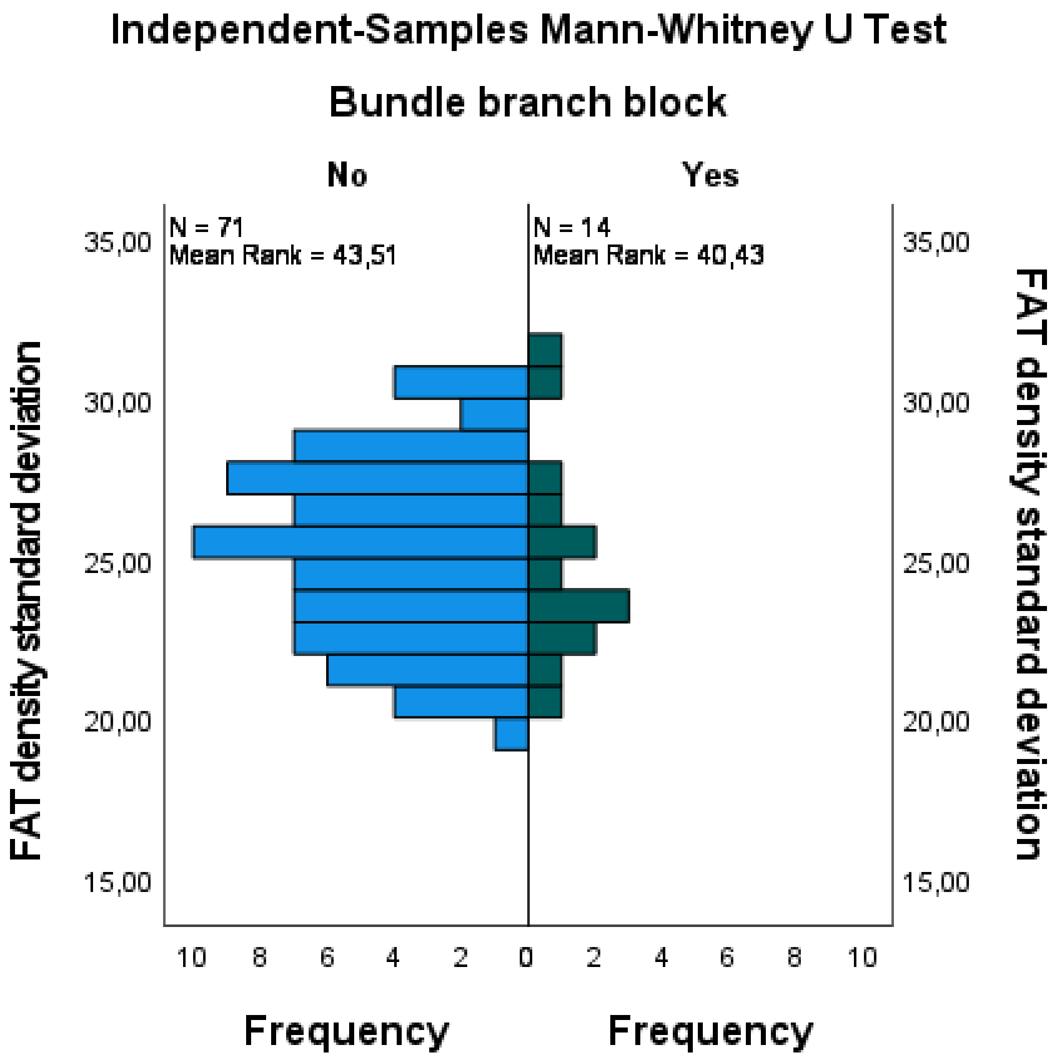

SAT mean density (HU) across Bundle branch block

Independent-Samples Mann-Whitney U Test Summary

Total N	85
Mann-Whitney U	563,500
Wilcoxon W	668,500

Test Statistic	563,500
Standard Error	84,392
Standardized Test Statistic	,788
Asymptotic Sig.(2-sided test)	,431

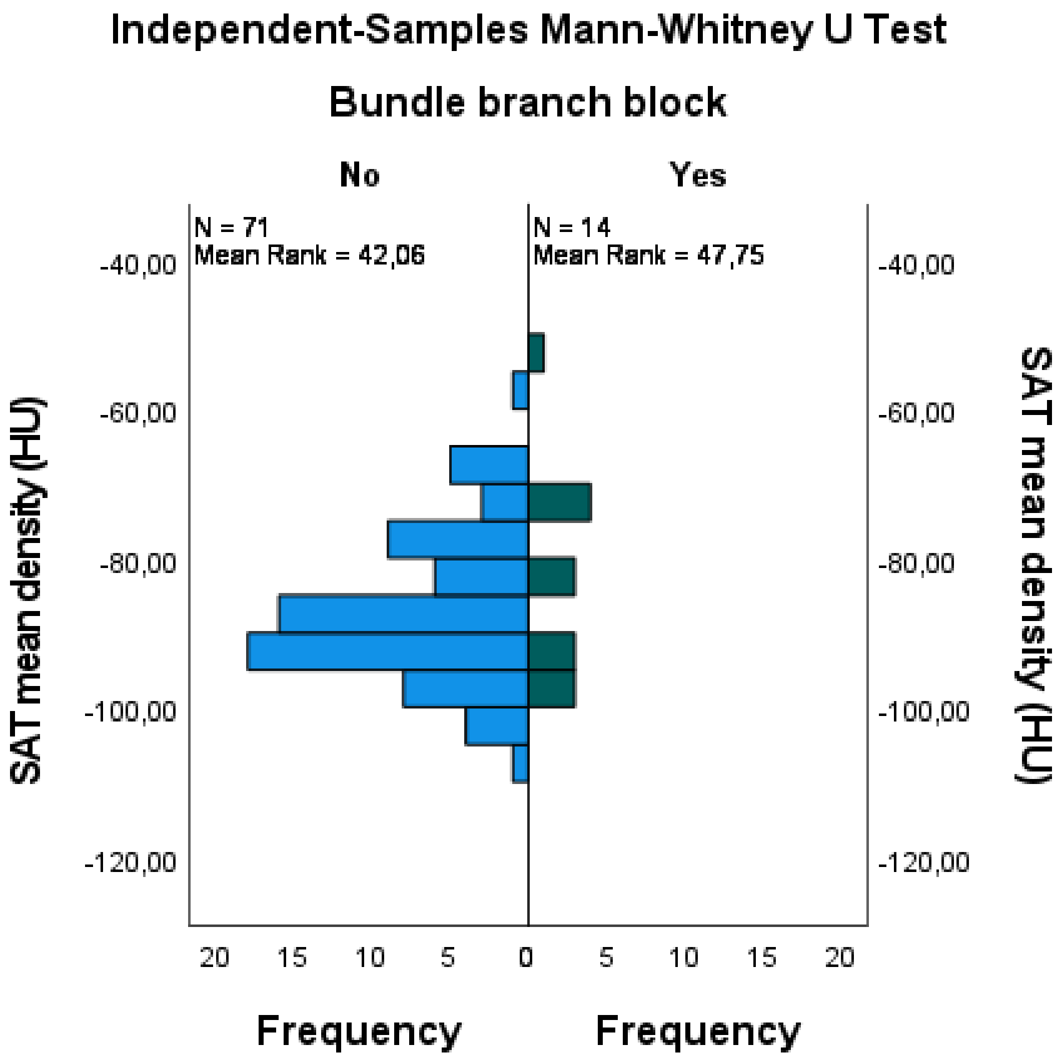

SAT median density (HU) across Bundle branch block

Independent-Samples Mann-Whitney U Test Summary

Total N	83
Mann-Whitney U	505,000
Wilcoxon W	610,000

Test Statistic	505,000
Standard Error	82,167
Standardized Test Statistic	,268
Asymptotic Sig.(2-sided test)	,789

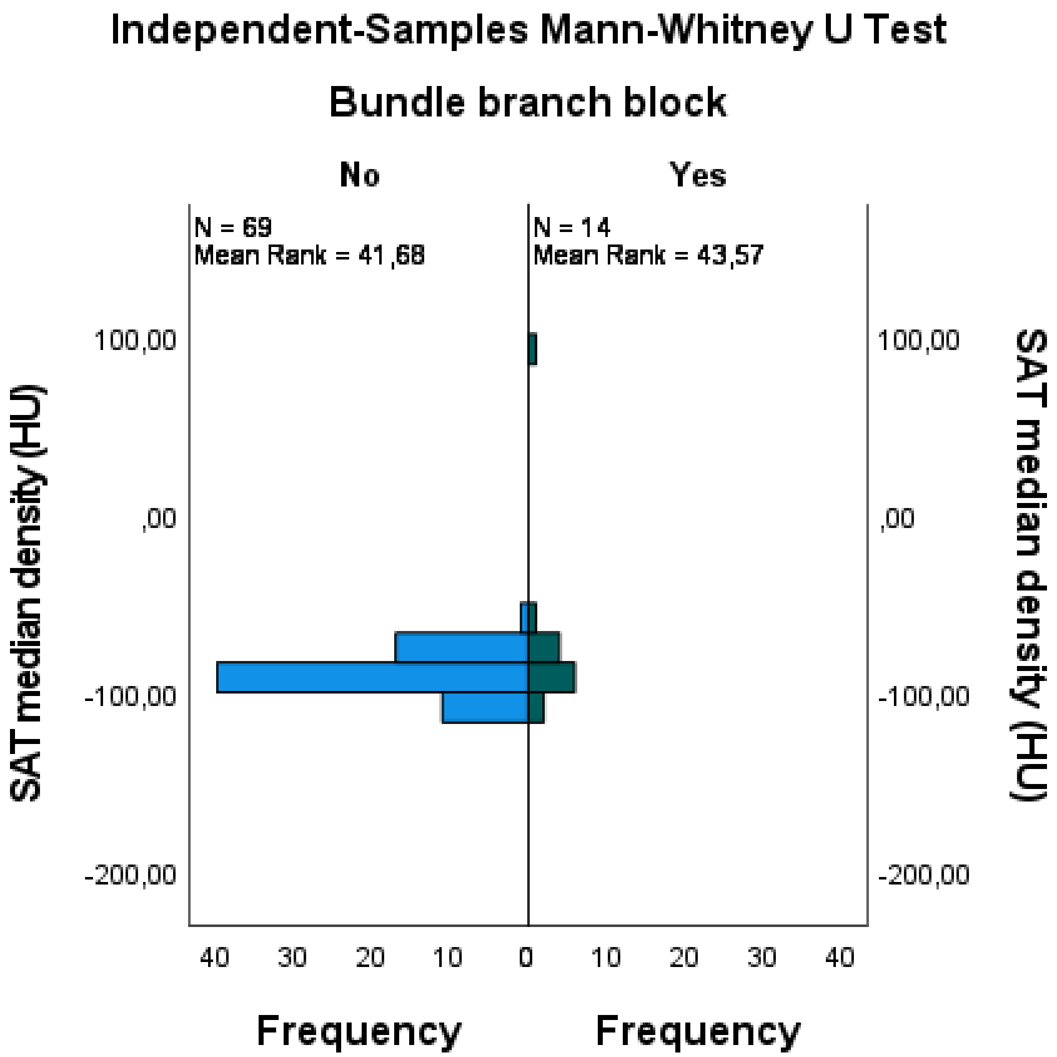

SAT density standard deviation across Bundle branch block

Independent-Samples Mann-Whitney U Test Summary

Total N	84
Mann-Whitney U	584,000

Wilcoxon W	689,000
Test Statistic	584,000
Standard Error	83,317
Standardized Test Statistic	1,128
Asymptotic Sig.(2-sided test)	,259

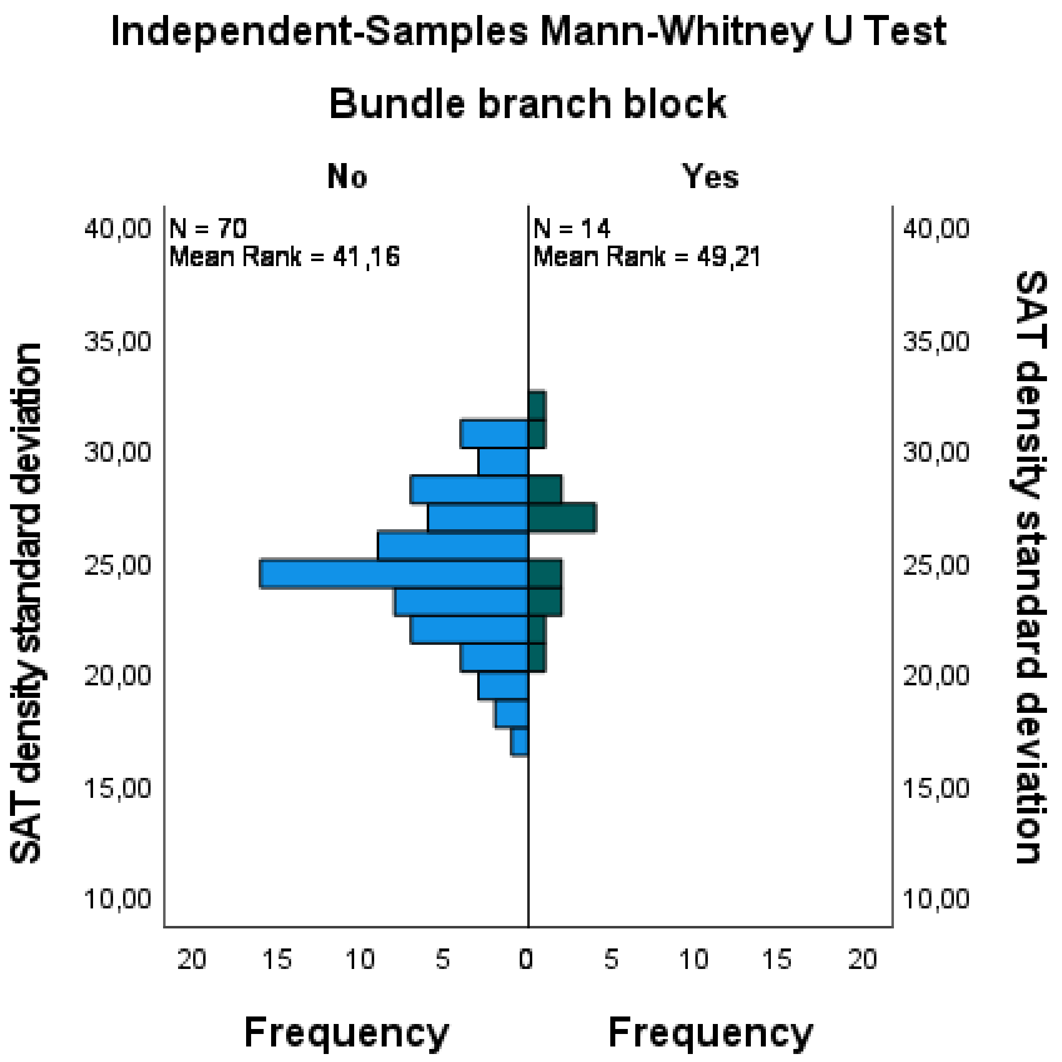

VAT mean density (HU) across Bundle branch block

Independent-Samples Mann-Whitney U Test Summary

Total N	85
Mann-Whitney U	456,500

Wilcoxon W	561,500
Test Statistic	456,500
Standard Error	84,387
Standardized Test Statistic	-,480
Asymptotic Sig.(2-sided test)	,631

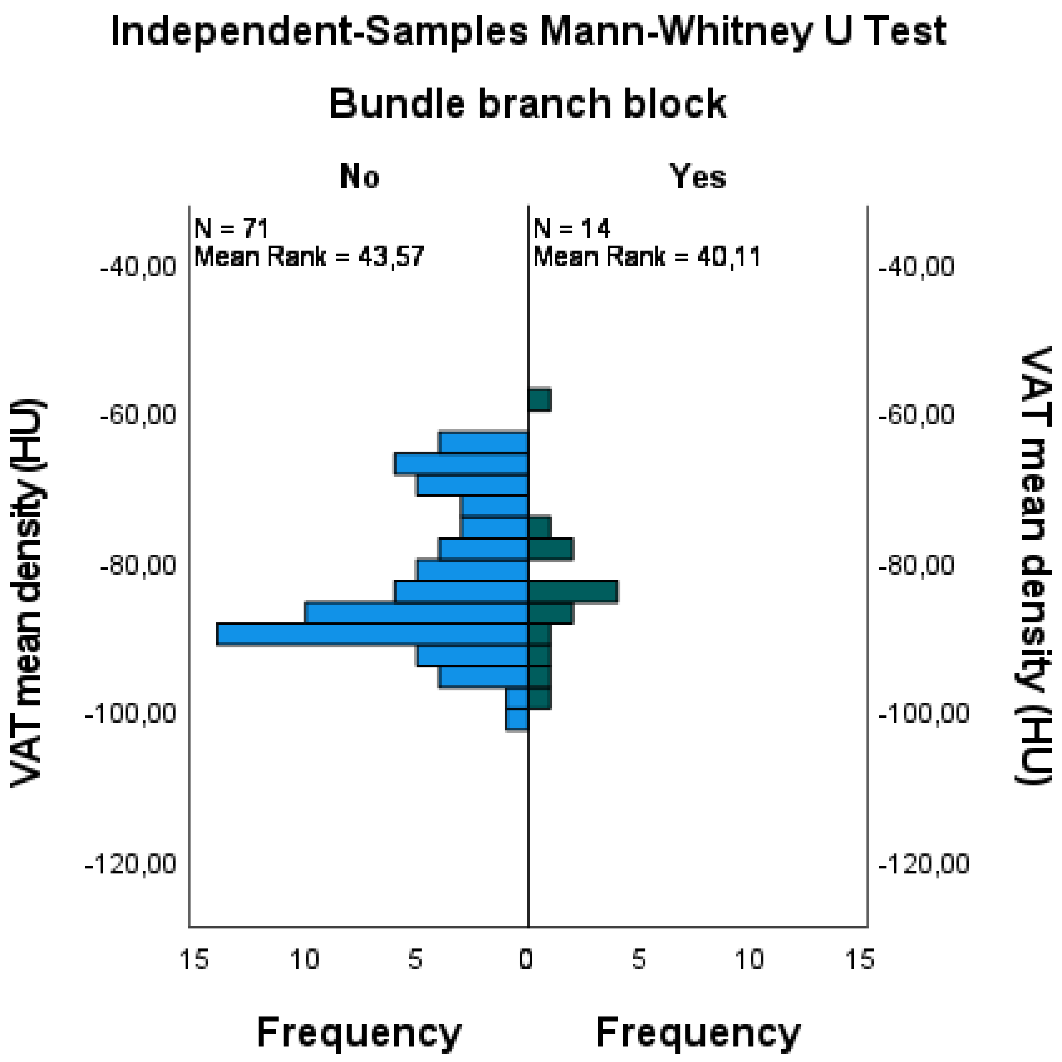

VAT median density (HU) across Bundle branch block

Independent-Samples Mann-Whitney U Test Summary

Total N	85
Mann-Whitney U	441,000

Wilcoxon W	546,000
Test Statistic	441,000
Standard Error	84,296
Standardized Test Statistic	-,664
Asymptotic Sig.(2-sided test)	,506

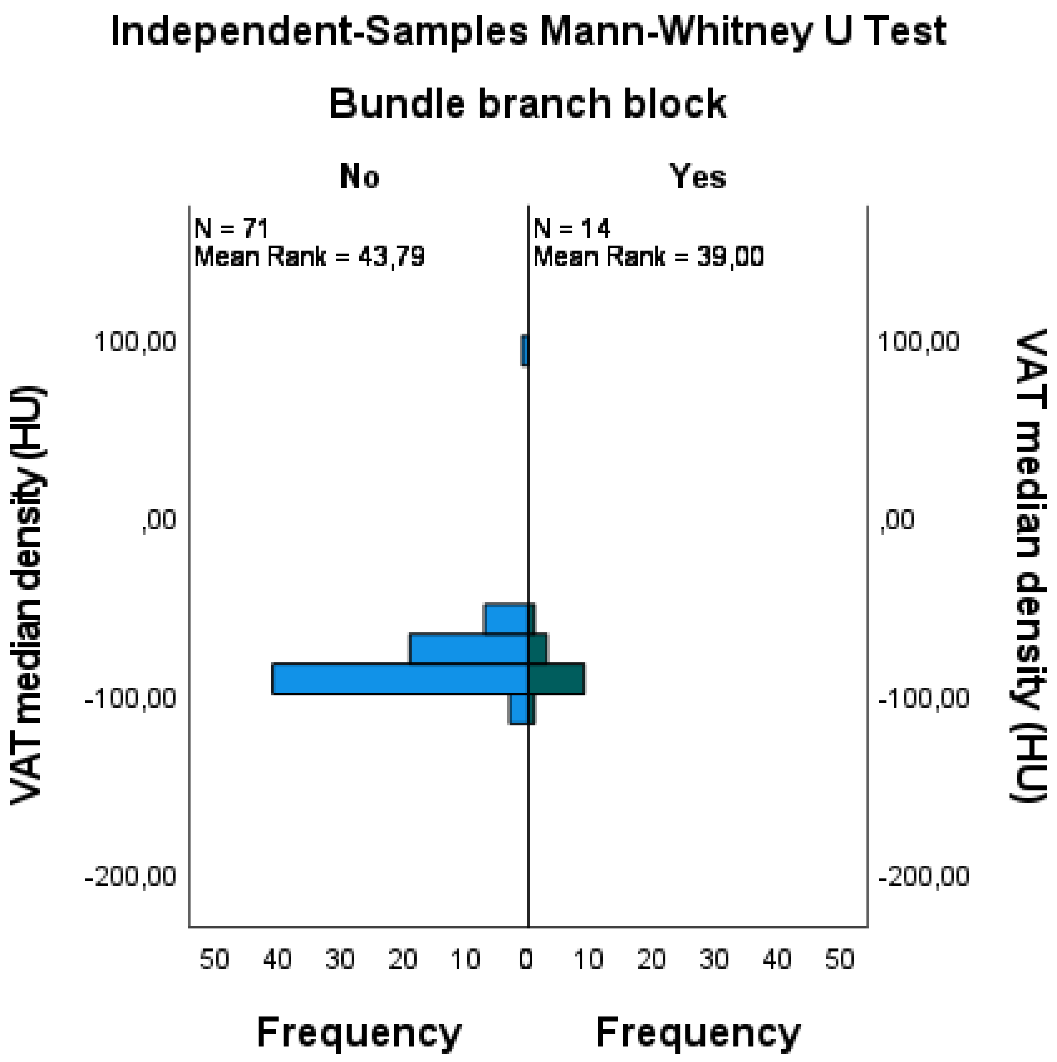

VAT density standard deviation across Bundle branch block

Independent-Samples Mann-Whitney U Test Summary

Total N	84
Mann-Whitney U	578,000

Wilcoxon W	683,000
Test Statistic	578,000
Standard Error	83,317
Standardized Test Statistic	1,056
Asymptotic Sig.(2-sided test)	,291

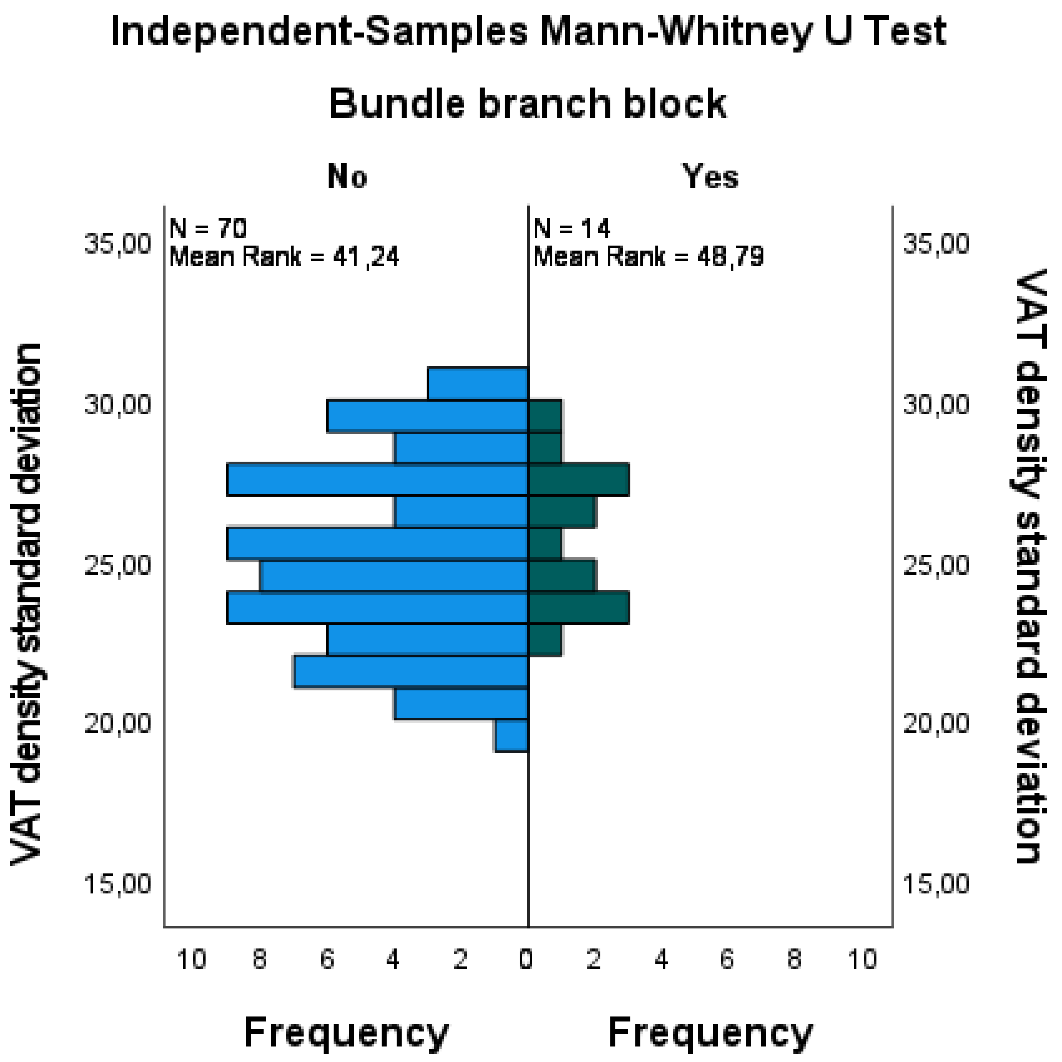

Right Psoas muscle mean density (HU) across Bundle branch block

Independent-Samples Mann-Whitney U Test Summary

Total N	85
Mann-Whitney U	560,000

Wilcoxon W	665,000
Test Statistic	560,000
Standard Error	84,383
Standardized Test Statistic	,747
Asymptotic Sig.(2-sided test)	,455

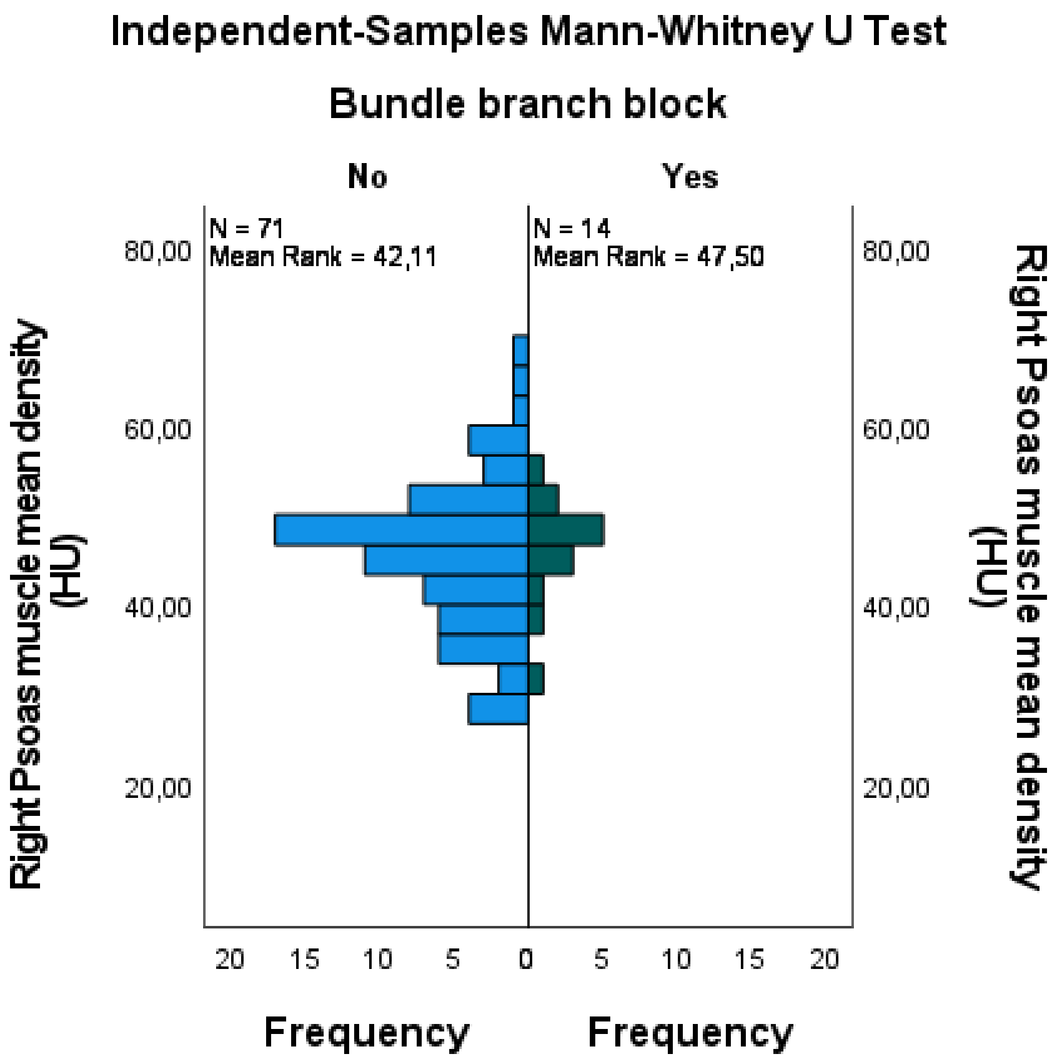

Right Psoas muscle median density (HU) across Bundle branch block

Independent-Samples Mann-Whitney U Test Summary

Total N	85
Mann-Whitney U	547,000

Wilcoxon W	652,000
Test Statistic	547,000
Standard Error	84,322
Standardized Test Statistic	,593
Asymptotic Sig.(2-sided test)	,553

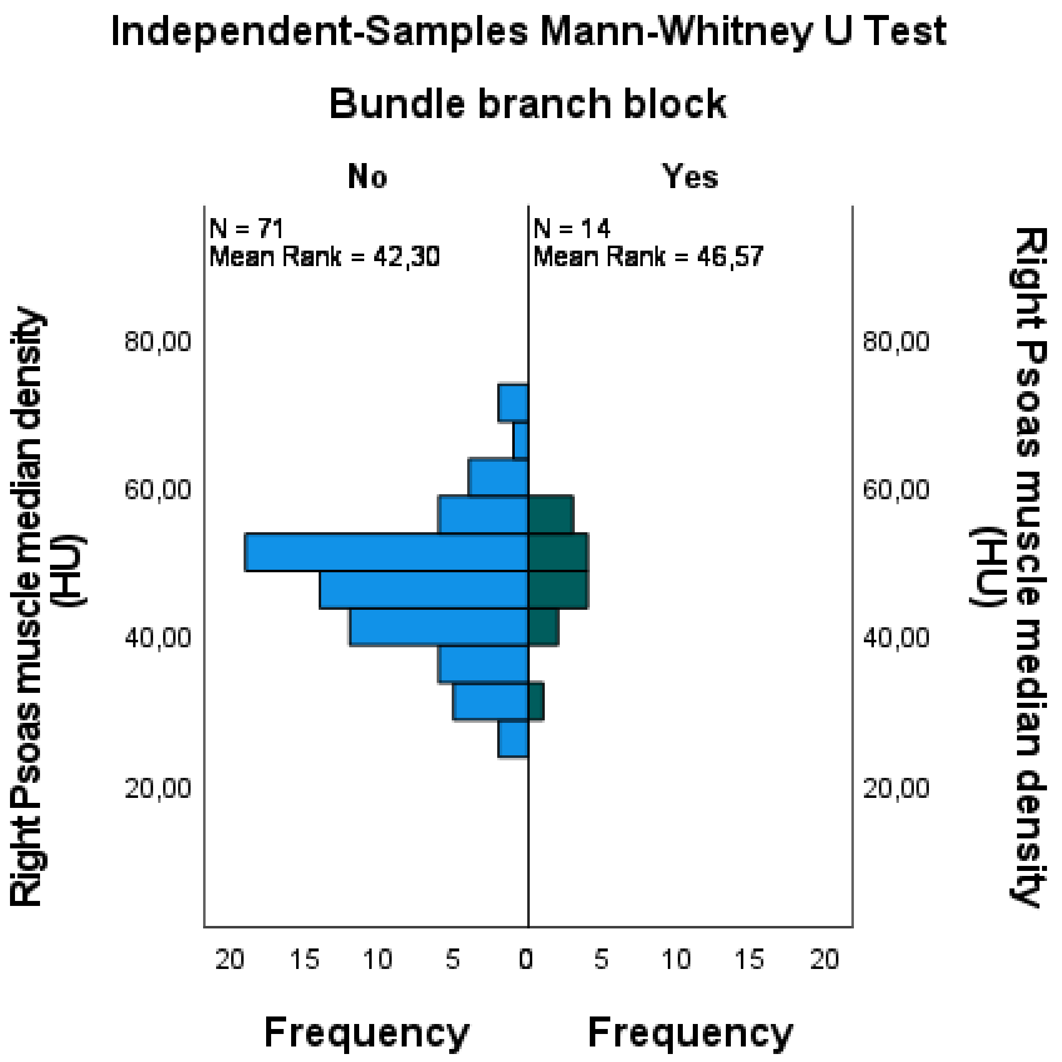

Right Psoas muscle density standard deviation across Bundle branch block

Independent-Samples Mann-Whitney U Test Summary

Total N	85
Mann-Whitney U	459,500

Wilcoxon W	564,500
Test Statistic	459,500
Standard Error	84,401
Standardized Test Statistic	-,444
Asymptotic Sig.(2-sided test)	,657

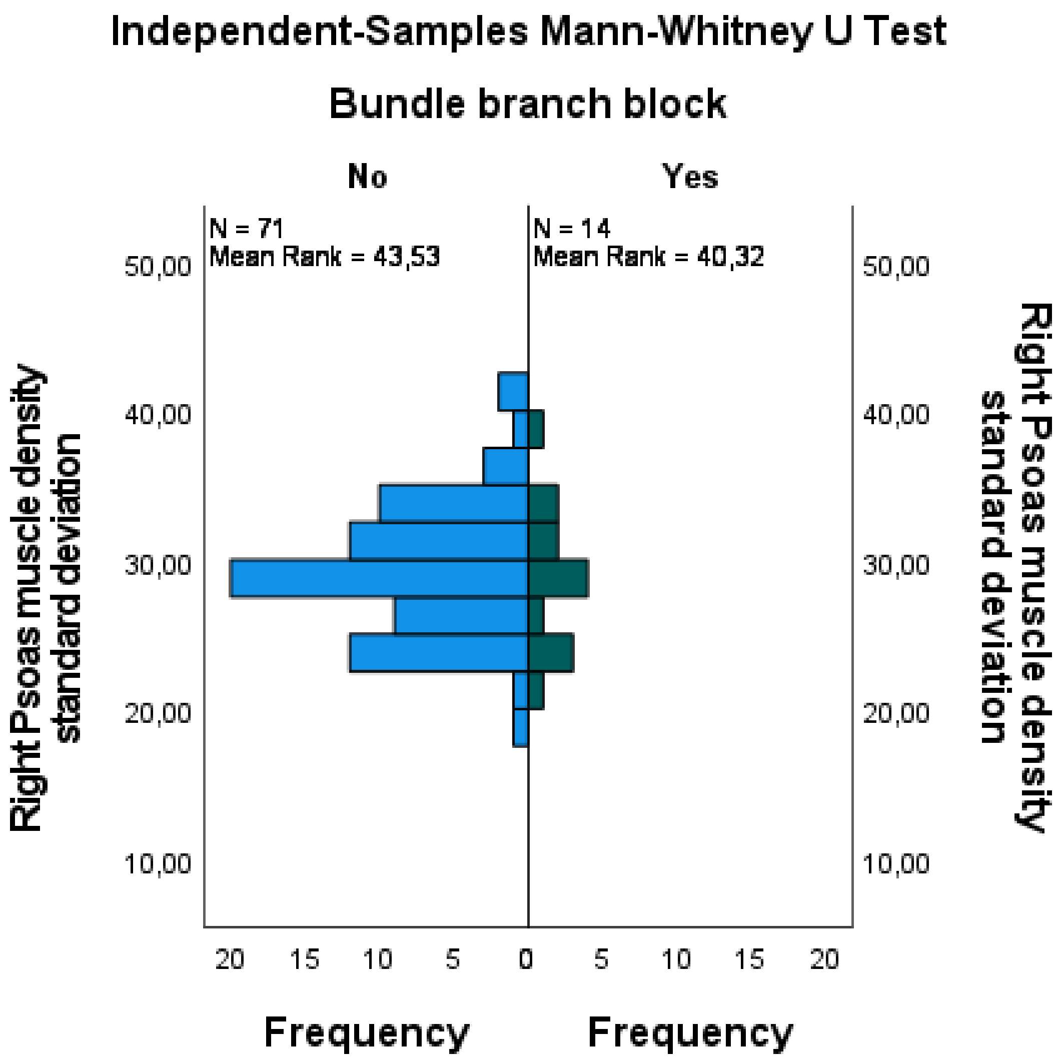

Left Psoas muscle mean density (HU) across Bundle branch block

Independent-Samples Mann-Whitney U Test Summary

Total N	85
Mann-Whitney U	550,500

Wilcoxon W	655,500
Test Statistic	550,500
Standard Error	84,384
Standardized Test Statistic	,634
Asymptotic Sig.(2-sided test)	,526

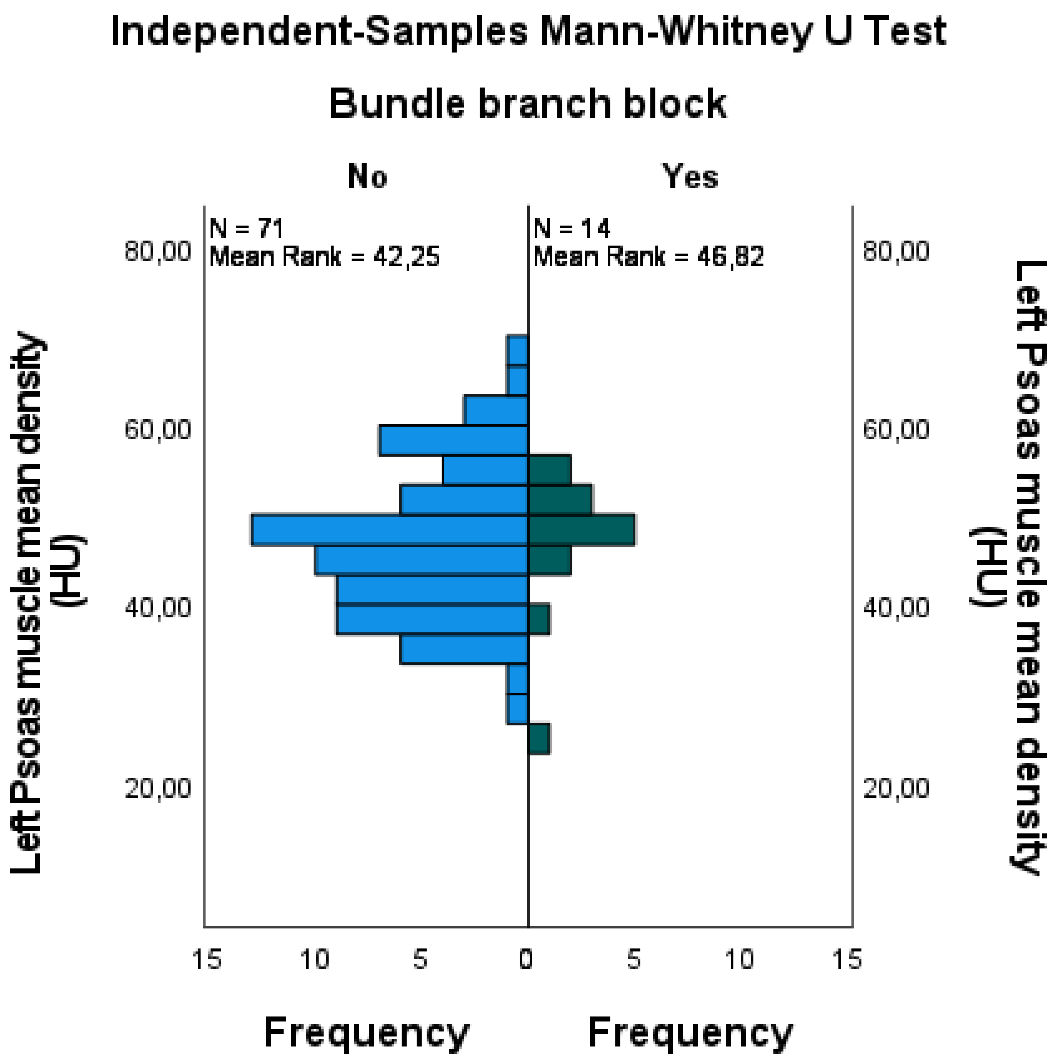

Left Psoas muscle median density (HU) across Bundle branch block

Independent-Samples Mann-Whitney U Test Summary

Total N	85
Mann-Whitney U	569,500

Wilcoxon W	674,500
Test Statistic	569,500
Standard Error	84,294
Standardized Test Statistic	,860
Asymptotic Sig.(2-sided test)	,390

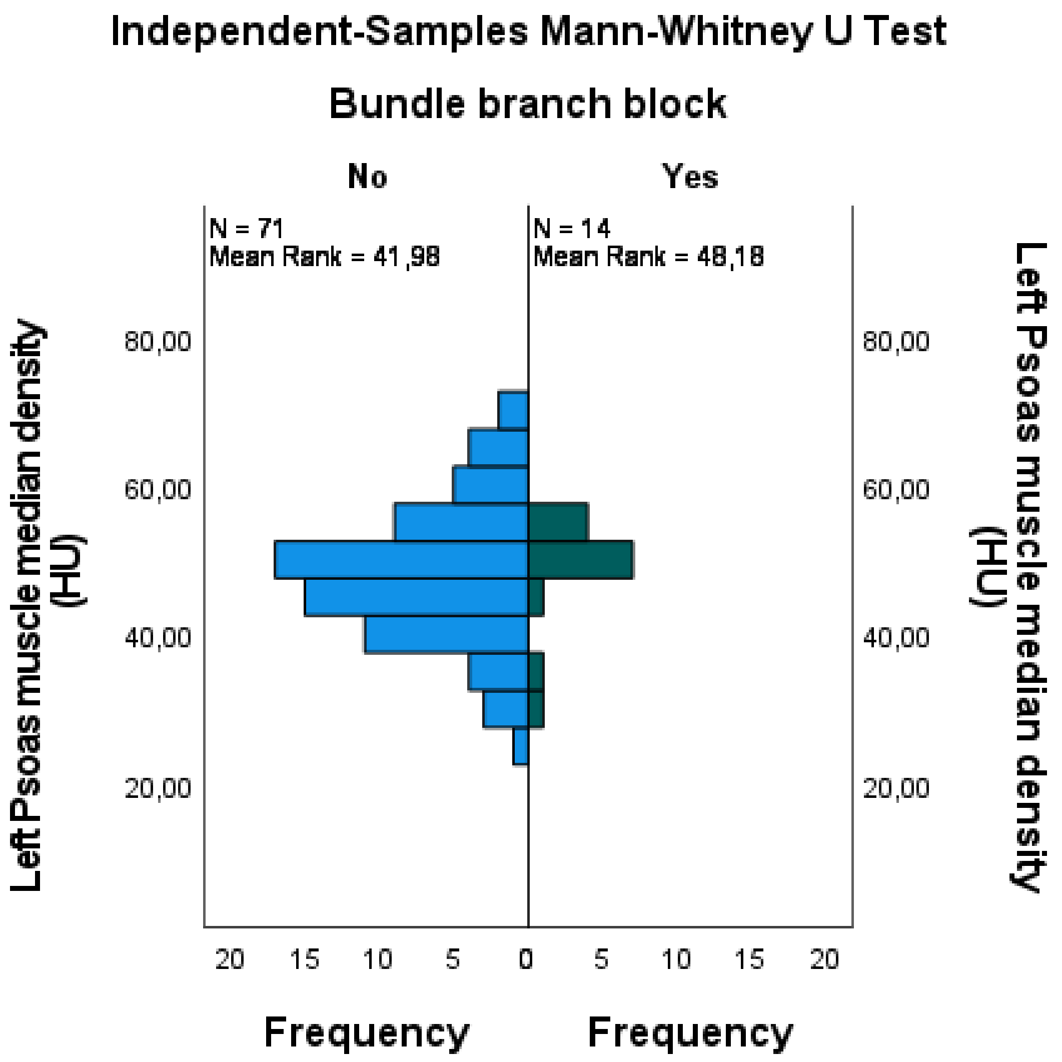

Left Psoas muscle density standard deviation across Bundle branch block

Independent-Samples Mann-Whitney U Test Summary

Total N	85
Mann-Whitney U	441,000

Wilcoxon W	546,000
Test Statistic	441,000
Standard Error	84,402
Standardized Test Statistic	-,663
Asymptotic Sig.(2-sided test)	,507

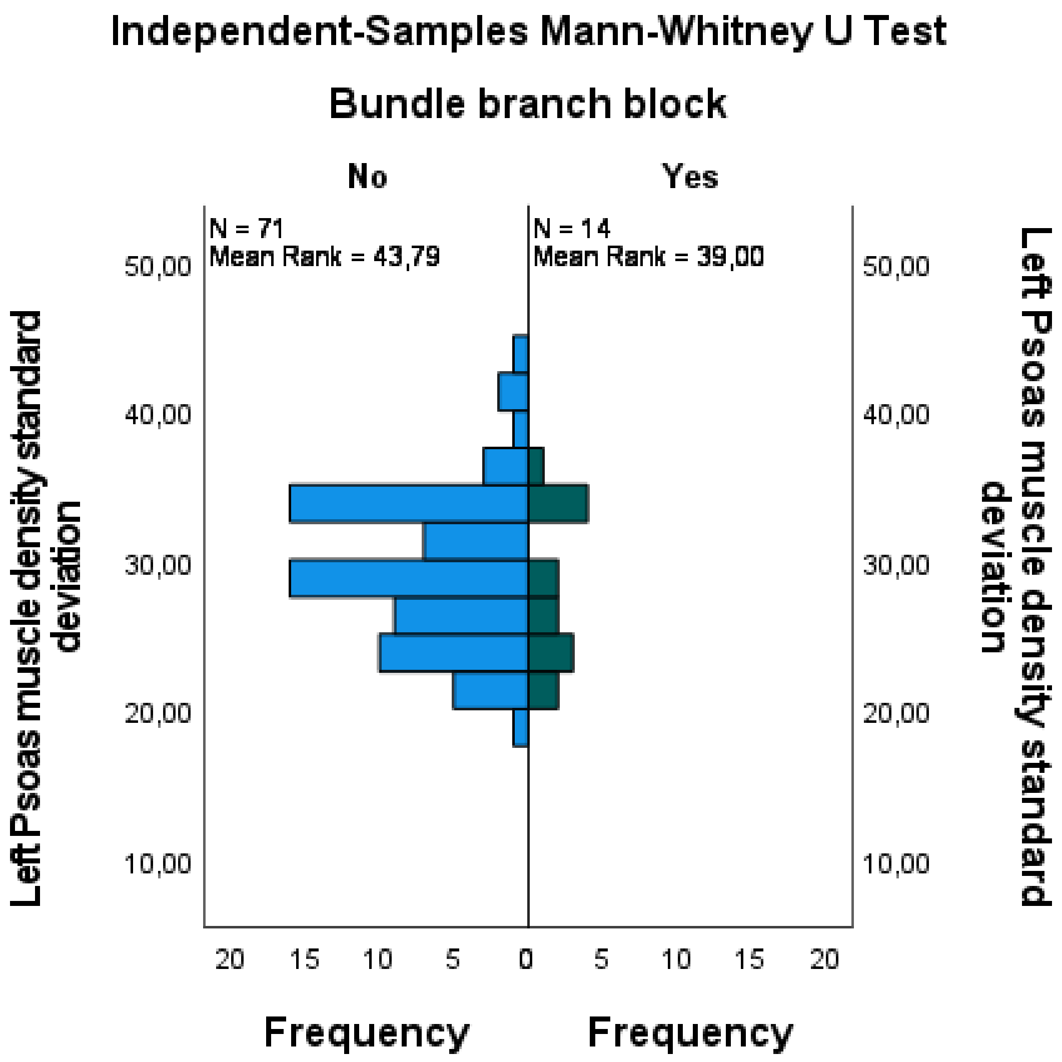

Independent-Samples Mann-Whitney U Test for atrioventricular block type 1 occurrence

Hypothesis Test Summary

	Null Hypothesis	Test	Sig.[a,b]	Decision
1	The distribution of Psoas/height is the same across categories of Atrioventricular block type 1.	Independent-Samples Mann-Whitney U Test	,817	Retain the null hypothesis.
2	The distribution of Anterior SAT distance is the same across categories of Atrioventricular block type 1.	Independent-Samples Mann-Whitney U Test	,984	Retain the null hypothesis.
3	The distribution of Posterior SAT distance is the same across categories of Atrioventricular block type 1.	Independent-Samples Mann-Whitney U Test	,383	Retain the null hypothesis.
4	The distribution of Anterior+Posterior SAT distance is the same across categories of Atrioventricular block type 1.	Independent-Samples Mann-Whitney U Test	,604	Retain the null hypothesis.
5	The distribution of VAT distance is the same across categories of Atrioventricular block type 1.	Independent-Samples Mann-Whitney U Test	,023	Reject the null hypothesis.
6	The distribution of Right common femoral artery area (mm2) is the same across categories of Atrioventricular block type 1.	Independent-Samples Mann-Whitney U Test	,891	Retain the null hypothesis.
7	The distribution of Left common femoral artery area (mm2) is the same across categories of Atrioventricular block type 1.	Independent-Samples Mann-Whitney U Test	,618	Retain the null hypothesis.
8	The distribution of FAT area (cm2) is the same across categories of Atrioventricular block type 1.	Independent-Samples Mann-Whitney U Test	,184	Retain the null hypothesis.
9	The distribution of SAT area (cm2) is the same across categories of Atrioventricular block type 1.	Independent-Samples Mann-Whitney U Test	,886	Retain the null hypothesis.
10	The distribution of VAT area (cm2) is the same across categories of Atrioventricular block type 1.	Independent-Samples Mann-Whitney U Test	,013	Reject the null hypothesis.

11	The distribution of Right Psoas muscle area (cm2) is the same across categories of Atrioventricular block type 1.	Independent-Samples Mann-Whitney U Test	,095	Retain the null hypothesis.
12	The distribution of Left Psoas muscle area (cm2) is the same across categories of Atrioventricular block type 1.	Independent-Samples Mann-Whitney U Test	,284	Retain the null hypothesis.
13	The distribution of FAT mean density (HU) is the same across categories of Atrioventricular block type 1.	Independent-Samples Mann-Whitney U Test	,544	Retain the null hypothesis.
14	The distribution of FAT median density (HU) is the same across categories of Atrioventricular block type 1.	Independent-Samples Mann-Whitney U Test	,432	Retain the null hypothesis.
15	The distribution of FAT density standard deviation is the same across categories of Atrioventricular block type 1.	Independent-Samples Mann-Whitney U Test	,881	Retain the null hypothesis.
16	The distribution of SAT mean density (HU) is the same across categories of Atrioventricular block type 1.	Independent-Samples Mann-Whitney U Test	,517	Retain the null hypothesis.
17	The distribution of SAT median density (HU) is the same across categories of Atrioventricular block type 1.	Independent-Samples Mann-Whitney U Test	,417	Retain the null hypothesis.
18	The distribution of SAT density standard deviation is the same across categories of Atrioventricular block type 1.	Independent-Samples Mann-Whitney U Test	,275	Retain the null hypothesis.
19	The distribution of VAT mean density (HU) is the same across categories of Atrioventricular block type 1.	Independent-Samples Mann-Whitney U Test	,040	Reject the null hypothesis.
20	The distribution of VAT median density (HU) is the same across categories of Atrioventricular block type 1.	Independent-Samples Mann-Whitney U Test	,027	Reject the null hypothesis.

21	The distribution of VAT density standard deviation is the same across categories of Atrioventricular block type 1.	Independent-Samples Mann-Whitney U Test	,571	Retain the null hypothesis.
22	The distribution of Right Psoas muscle mean density (HU) is the same across categories of Atrioventricular block type 1.	Independent-Samples Mann-Whitney U Test	,263	Retain the null hypothesis.
23	The distribution of Right Psoas muscle median density (HU) is the same across categories of Atrioventricular block type 1.	Independent-Samples Mann-Whitney U Test	,150	Retain the null hypothesis.
24	The distribution of Right Psoas muscle density standard deviation is the same across categories of Atrioventricular block type 1.	Independent-Samples Mann-Whitney U Test	,466	Retain the null hypothesis.
25	The distribution of Left Psoas muscle mean density (HU) is the same across categories of Atrioventricular block type 1.	Independent-Samples Mann-Whitney U Test	,774	Retain the null hypothesis.
26	The distribution of Left Psoas muscle median density (HU) is the same across categories of Atrioventricular block type 1.	Independent-Samples Mann-Whitney U Test	,785	Retain the null hypothesis.
27	The distribution of Left Psoas muscle density standard deviation is the same across categories of Atrioventricular block type 1.	Independent-Samples Mann-Whitney U Test	,785	Retain the null hypothesis.

a. The significance level is ,050.

b. Asymptotic significance is displayed.

In this case, the hypothesis of equal medians ($p < 0.05$) is rejected for the variables VAT distance, VAT area (cm2), VAT mean density (HU) and VAT median density (HU), while for the remaining variables the null hypothesis ($p > 0.05$).

(The tables and graphs below are the details of the tests in this table: I have highlighted what things you should eventually report, namely test statistic and pvalue).

Psoas/height across Atrioventricular block type 1

Independent-Samples Mann-Whitney U Test Summary

Total N	85
Mann-Whitney U	358,000
Wilcoxon W	413,000
Test Statistic	358,000
Standard Error	73,314
Standardized Test Statistic	-,232
Asymptotic Sig.(2-sided test)	,817

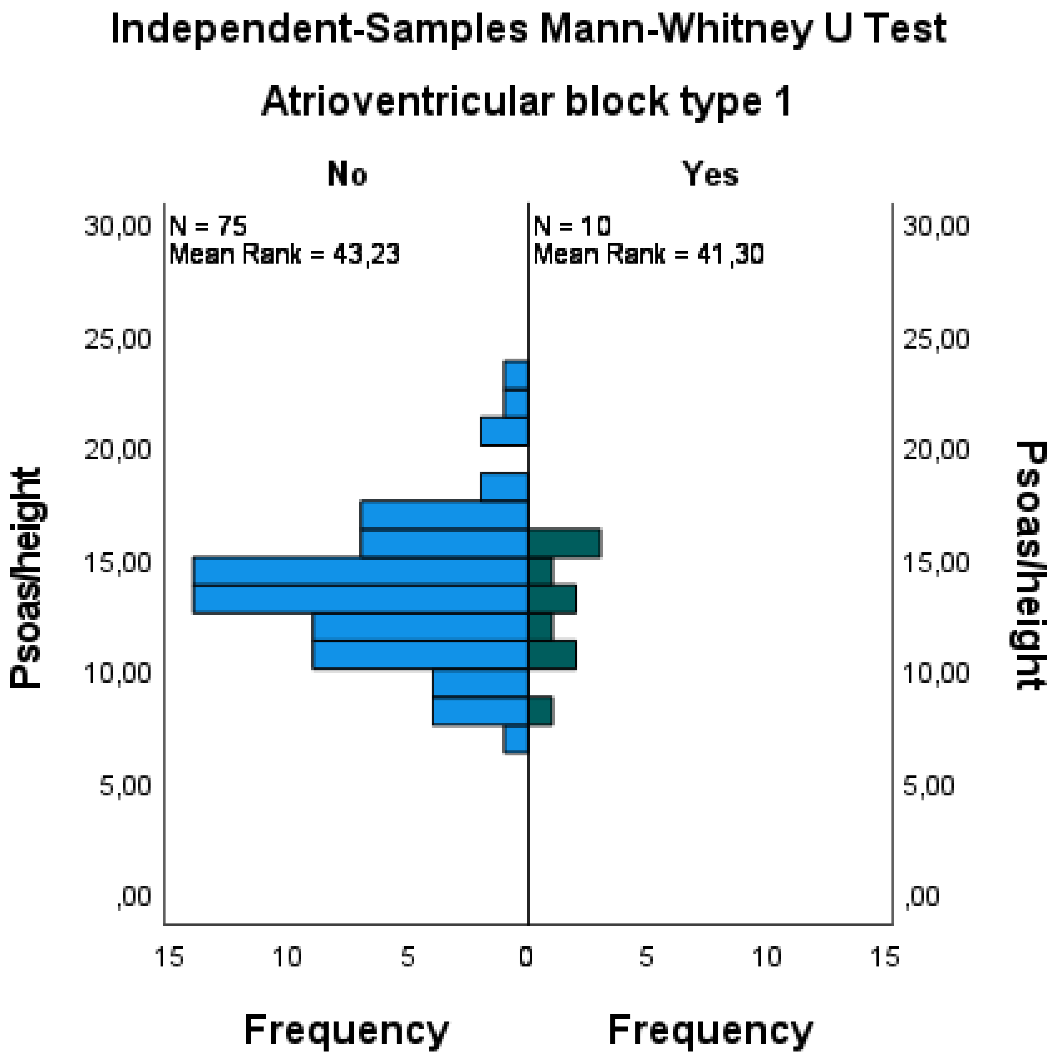

Anterior SAT distance across Atrioventricular block type 1

Independent-Samples Mann-Whitney U Test Summary

Total N	85
Mann-Whitney U	373,500
Wilcoxon W	428,500
Test Statistic	373,500
Standard Error	73,304
Standardized Test Statistic	-,020
Asymptotic Sig.(2-sided test)	,984

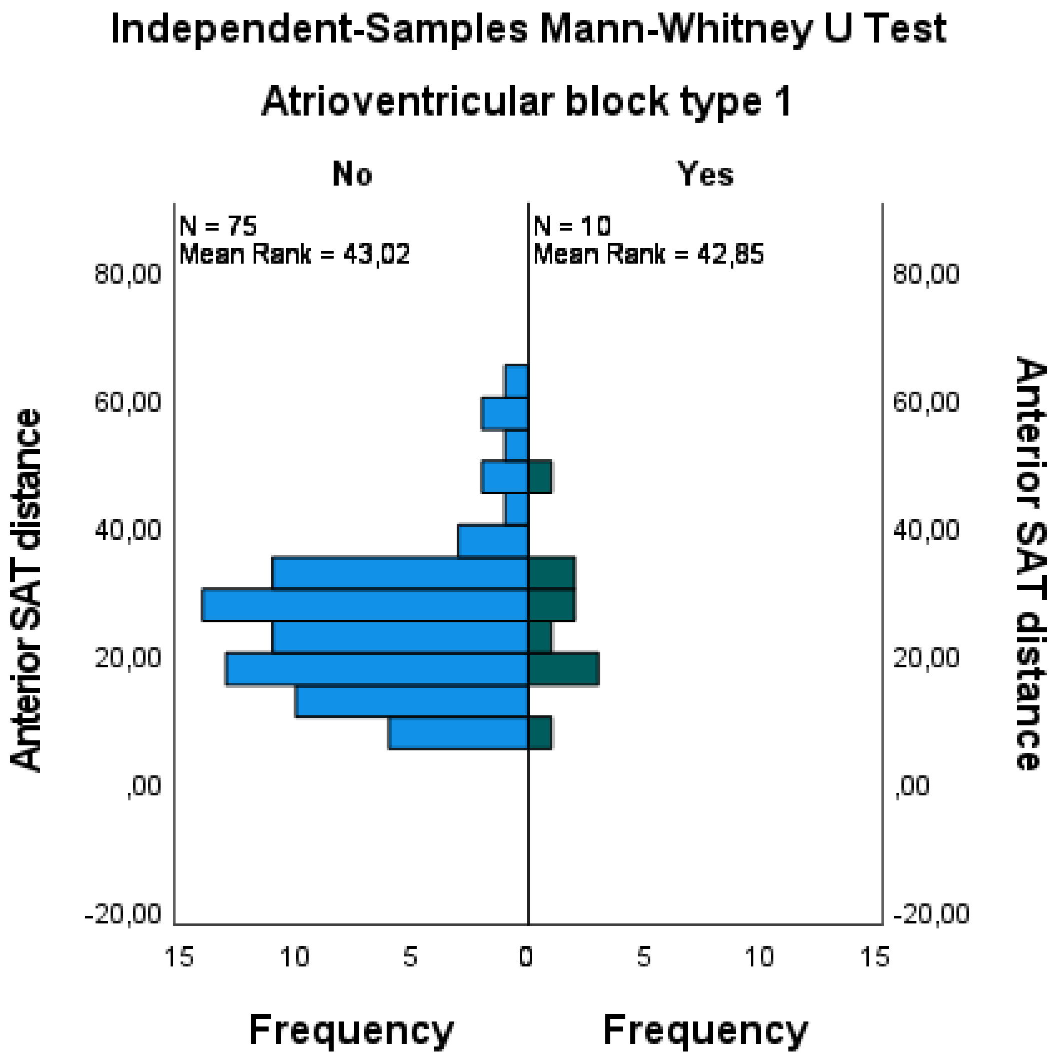

Posterior SAT distance across Atrioventricular block type 1

Independent-Samples Mann-Whitney U Test Summary

Total N	85
Mann-Whitney U	439,000
Wilcoxon W	494,000
Test Statistic	439,000
Standard Error	73,306
Standardized Test Statistic	,873
Asymptotic Sig.(2-sided test)	,383

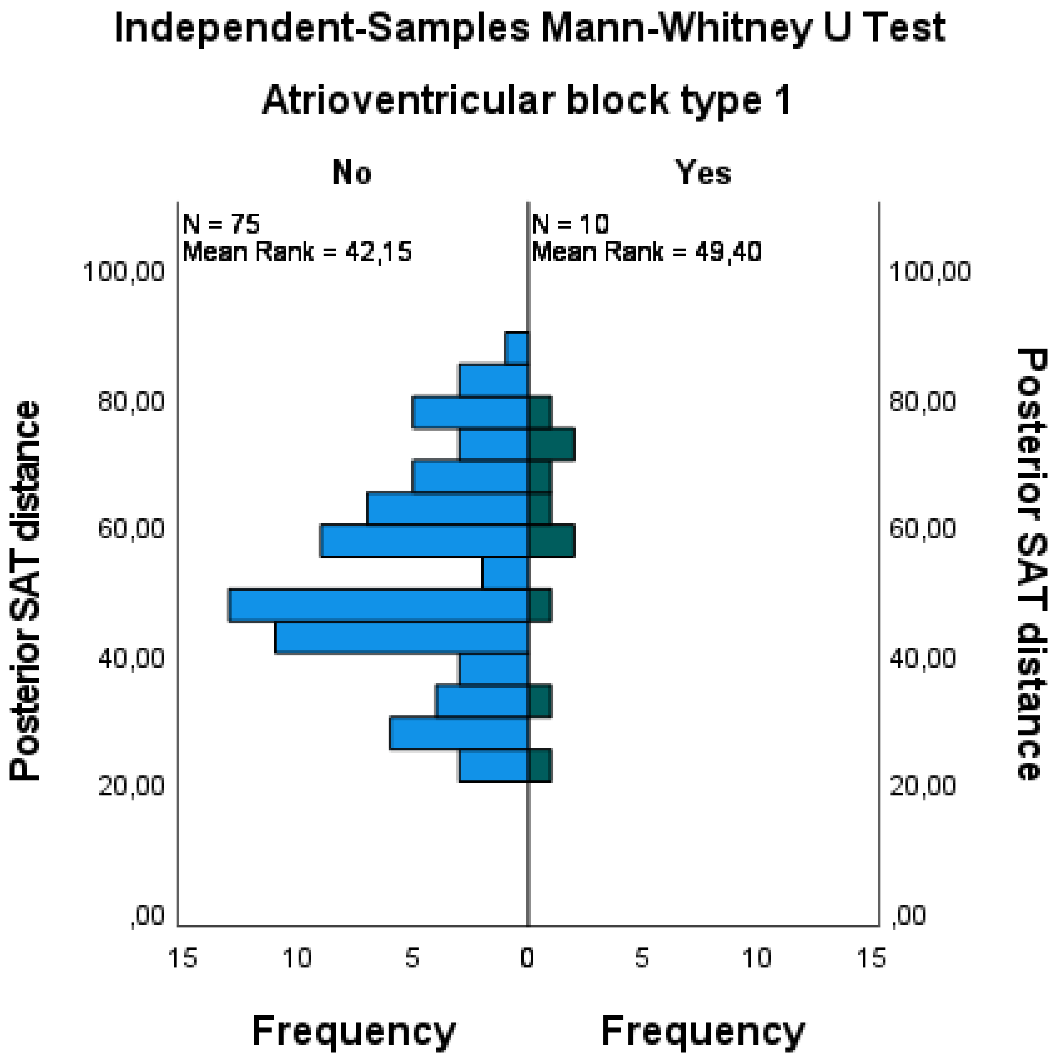

Anterior+Posterior SAT distance across Atrioventricular block type 1

Independent-Samples Mann-Whitney U Test Summary

Total N	85
Mann-Whitney U	413,000
Wilcoxon W	468,000
Test Statistic	413,000
Standard Error	73,311
Standardized Test Statistic	,518
Asymptotic Sig.(2-sided test)	,604

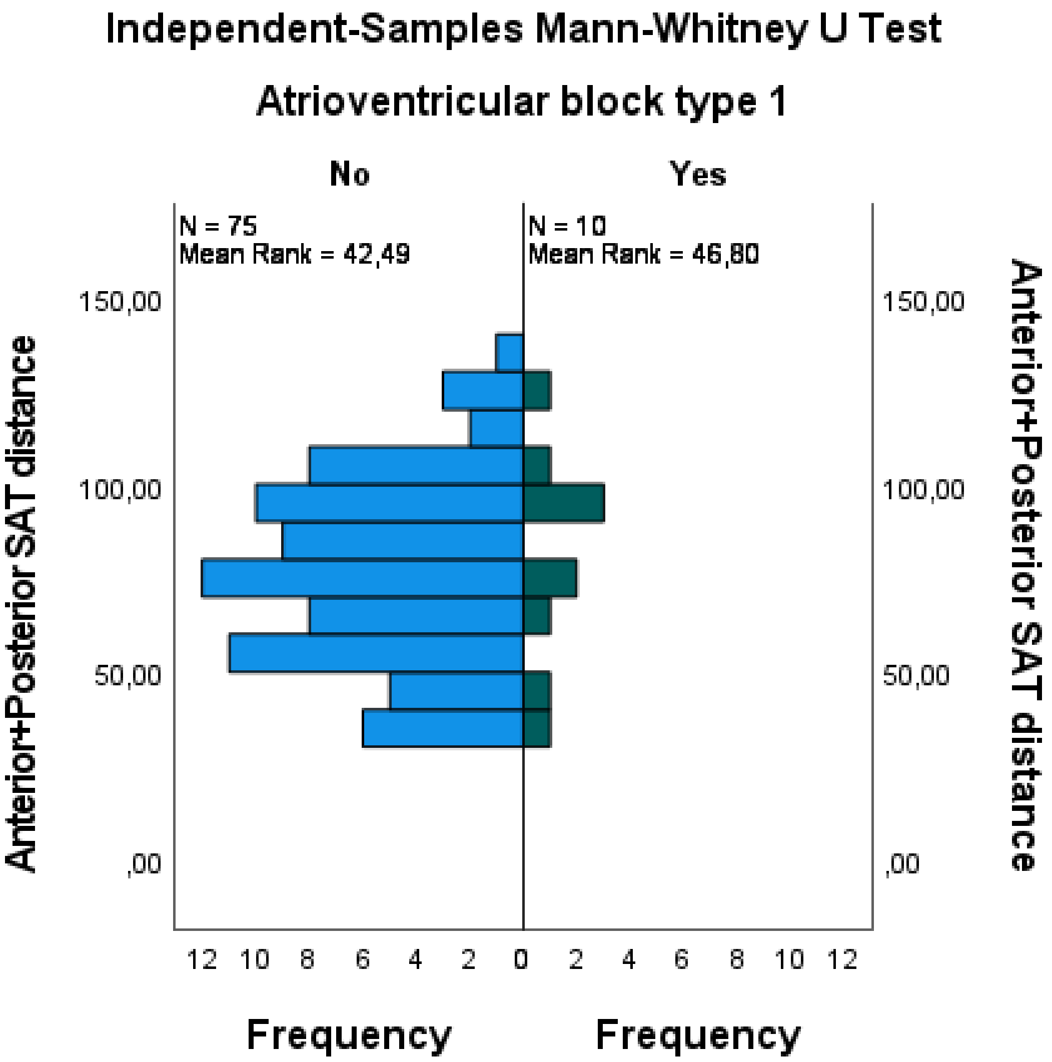

VAT distance across Atrioventricular block type 1

Independent-Samples Mann-Whitney U Test Summary

Total N	84
Mann-Whitney U	535,000
Wilcoxon W	590,000
Test Statistic	535,000
Standard Error	72,385
Standardized Test Statistic	2,279
Asymptotic Sig.(2-sided test)	,023

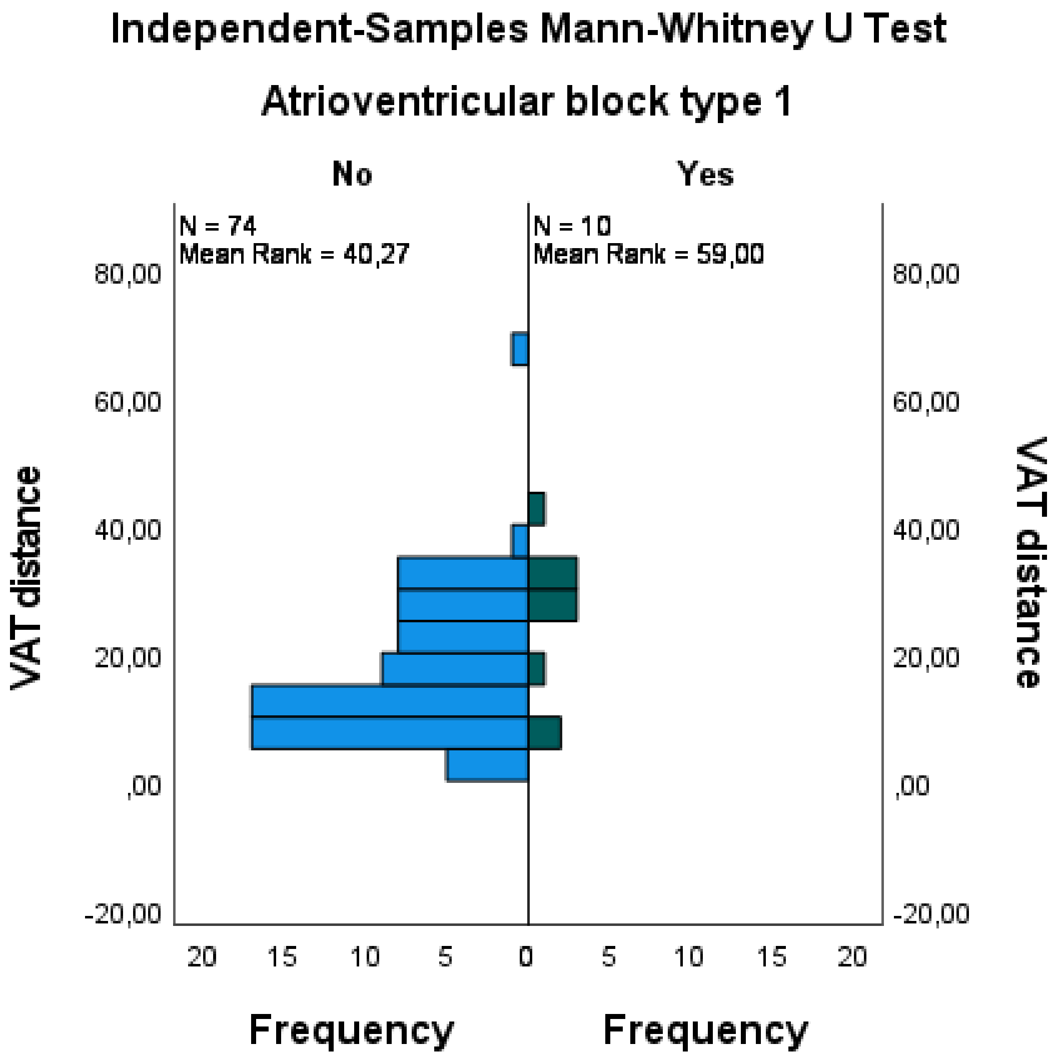

Right common femoral artery area (mm2) across Atrioventricular block type 1

Independent-Samples Mann-Whitney U Test Summary

Total N	85
Mann-Whitney U	385,000
Wilcoxon W	440,000
Test Statistic	385,000
Standard Error	73,302
Standardized Test Statistic	,136
Asymptotic Sig.(2-sided test)	,891

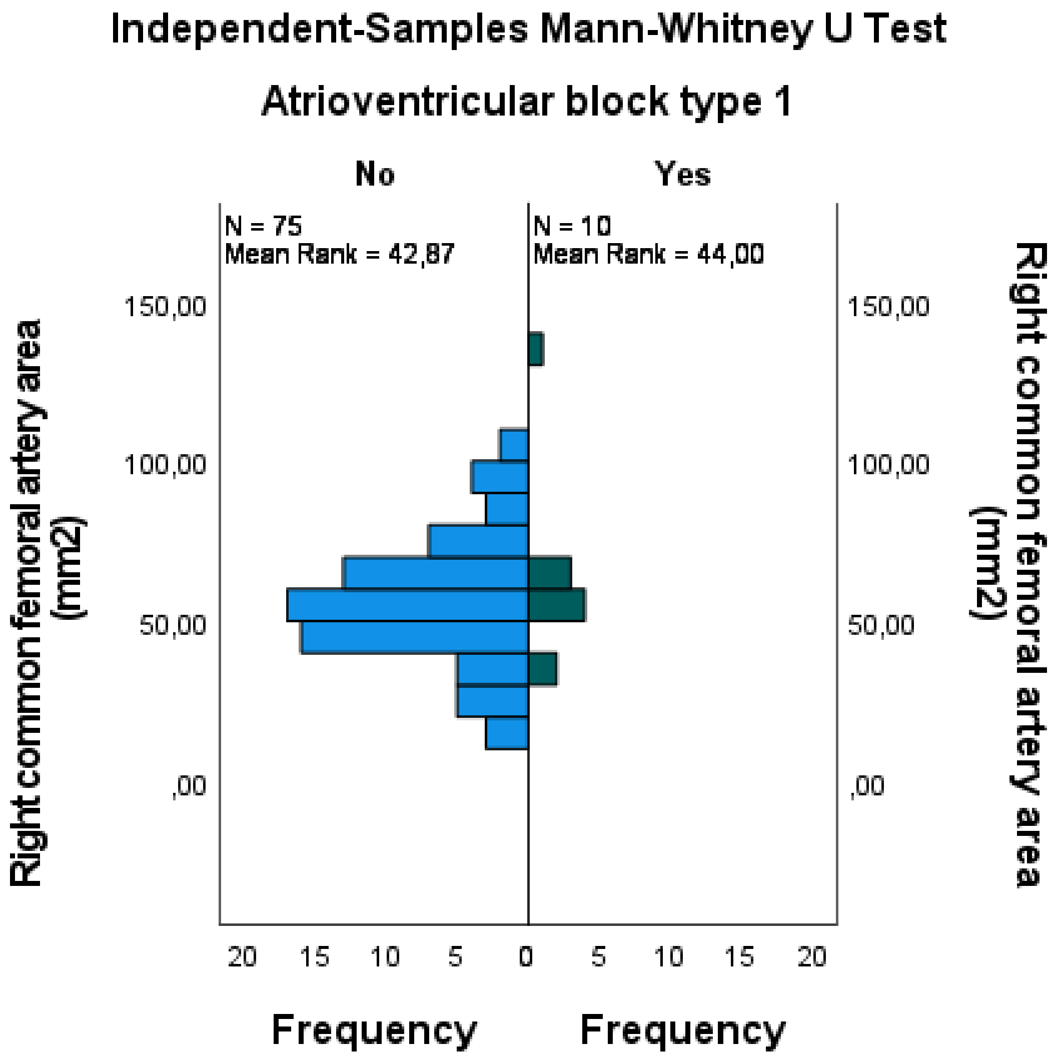

Left common femoral artery area (mm2) across Atrioventricular block type 1

Independent-Samples Mann-Whitney U Test Summary

Total N	85
Mann-Whitney U	411,500
Wilcoxon W	466,500
Test Statistic	411,500
Standard Error	73,296
Standardized Test Statistic	,498
Asymptotic Sig.(2-sided test)	,618

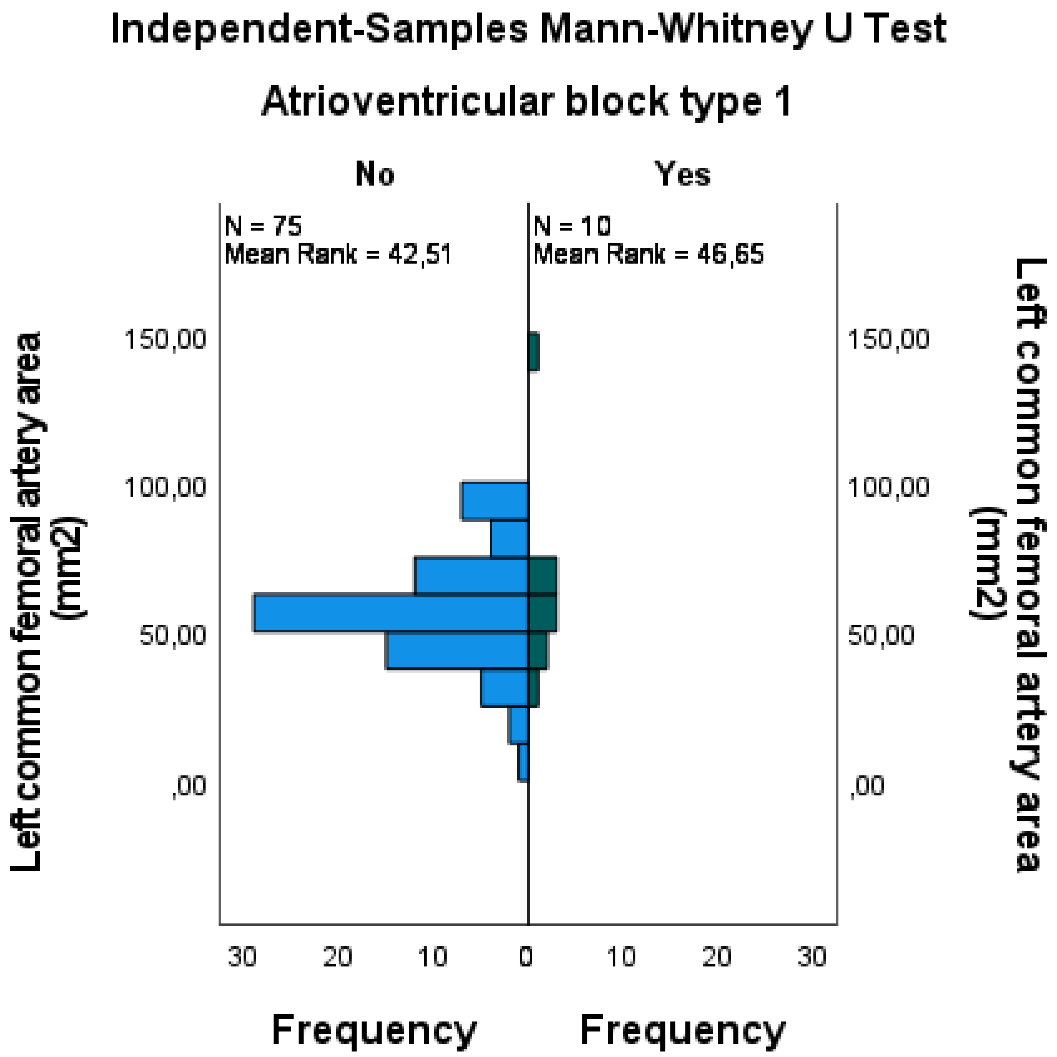

FAT area (cm2) across Atrioventricular block type 1

Independent-Samples Mann-Whitney U Test Summary

Total N	85
Mann-Whitney U	472,500
Wilcoxon W	527,500
Test Statistic	472,500
Standard Error	73,314
Standardized Test Statistic	1,330
Asymptotic Sig.(2-sided test)	,184

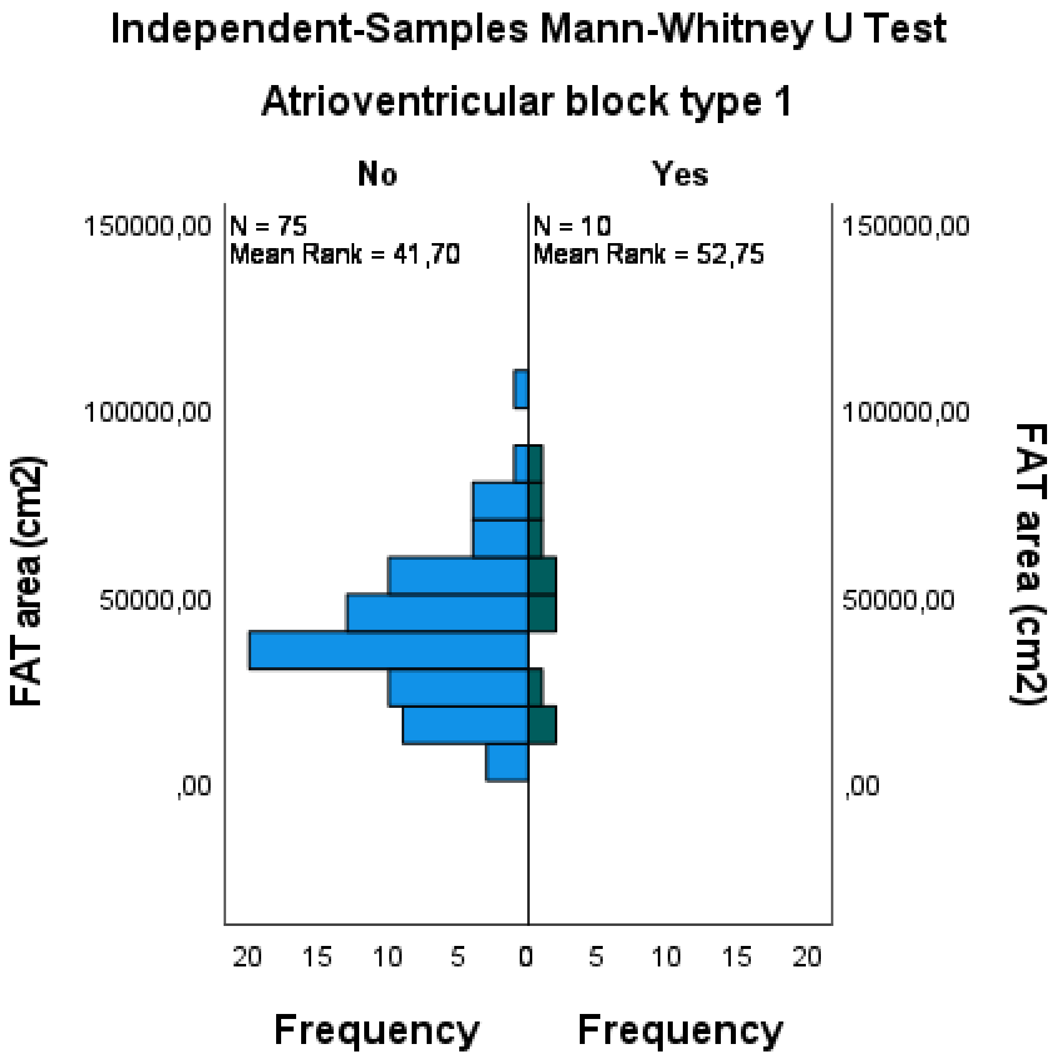

SAT area (cm2) across Atrioventricular block type 1

Independent-Samples Mann-Whitney U Test Summary

Total N	85
Mann-Whitney U	385,500
Wilcoxon W	440,500
Test Statistic	385,500
Standard Error	73,314
Standardized Test Statistic	,143
Asymptotic Sig.(2-sided test)	,886

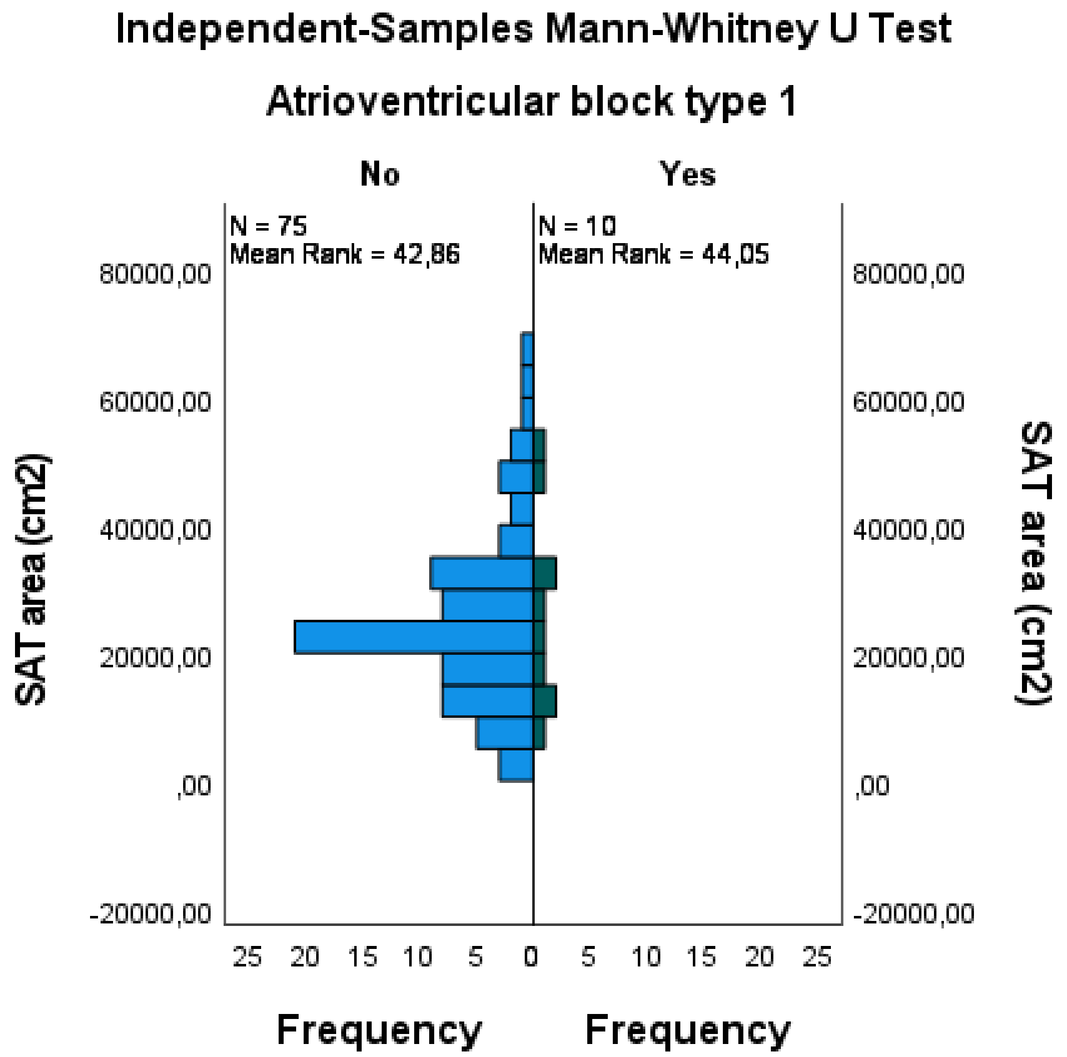

VAT area (cm2) across Atrioventricular block type 1

Independent-Samples Mann-Whitney U Test Summary

Total N	84
Mann-Whitney U	550,000
Wilcoxon W	605,000
Test Statistic	550,000
Standard Error	72,399
Standardized Test Statistic	2,486
Asymptotic Sig.(2-sided test)	,013

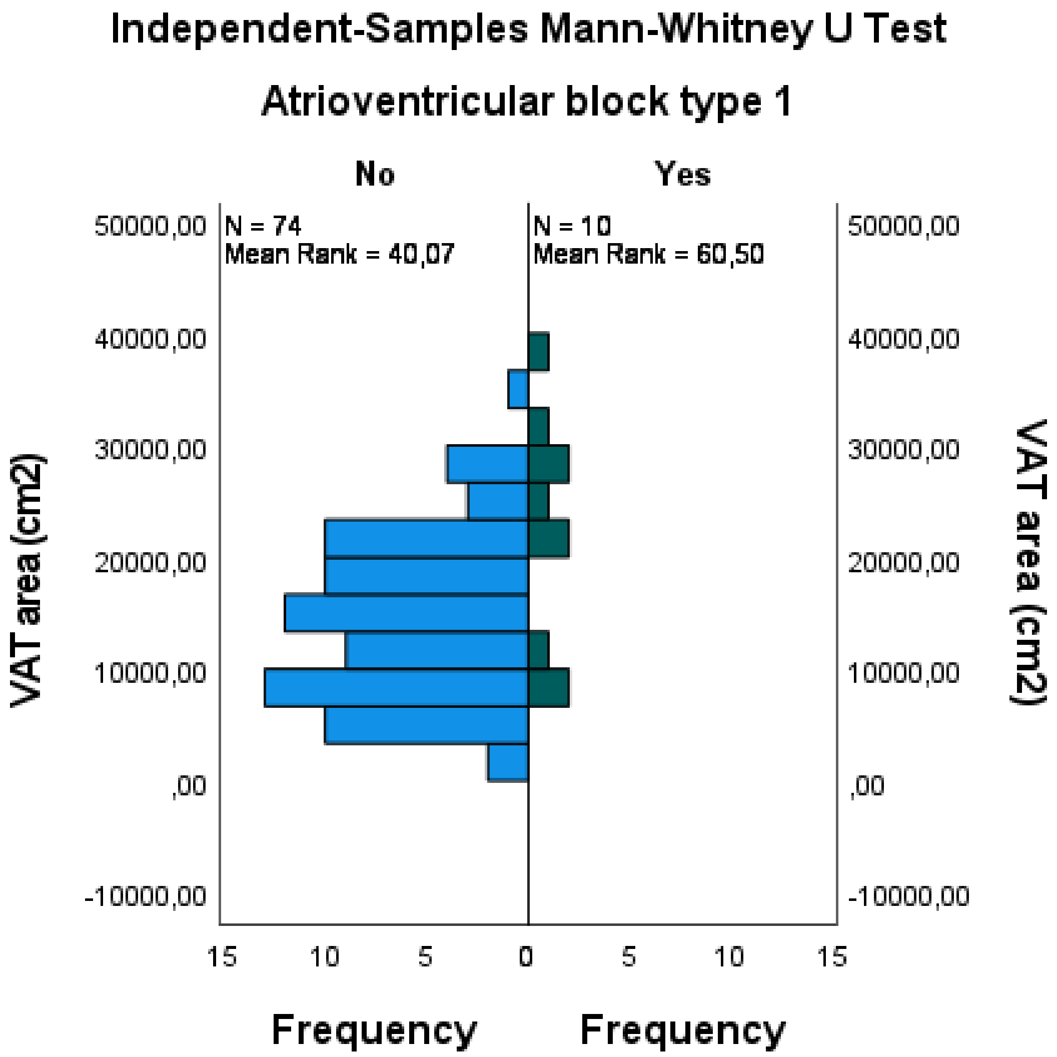

Right Psoas muscle area (cm2) across Atrioventricular block type 1

Independent-Samples Mann-Whitney U Test Summary

Total N	85
Mann-Whitney U	497,500
Wilcoxon W	552,500
Test Statistic	497,500
Standard Error	73,312
Standardized Test Statistic	1,671
Asymptotic Sig.(2-sided test)	,095

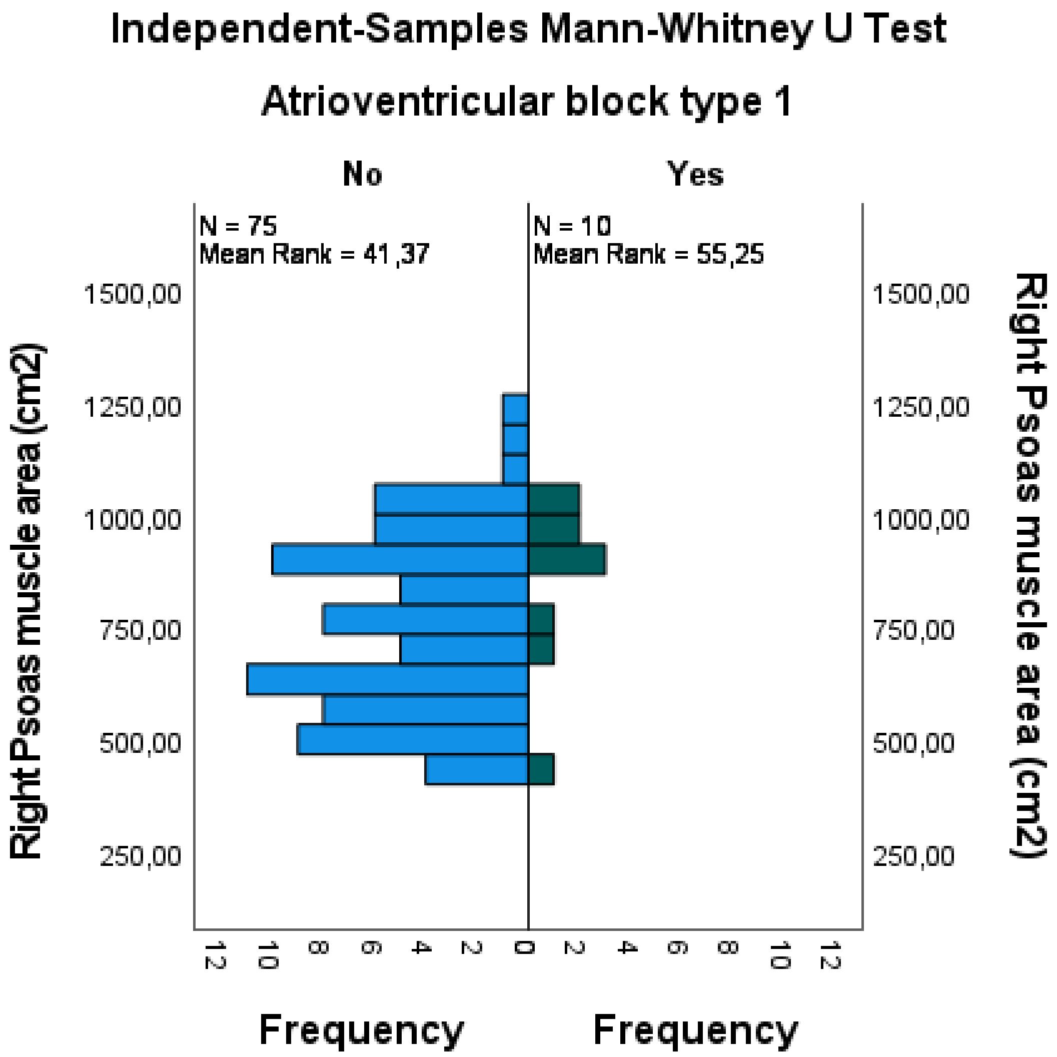

Left Psoas muscle area (cm2) across Atrioventricular block type 1

Independent-Samples Mann-Whitney U Test Summary

Total N	85
Mann-Whitney U	453,500
Wilcoxon W	508,500
Test Statistic	453,500
Standard Error	73,314
Standardized Test Statistic	1,071
Asymptotic Sig.(2-sided test)	,284

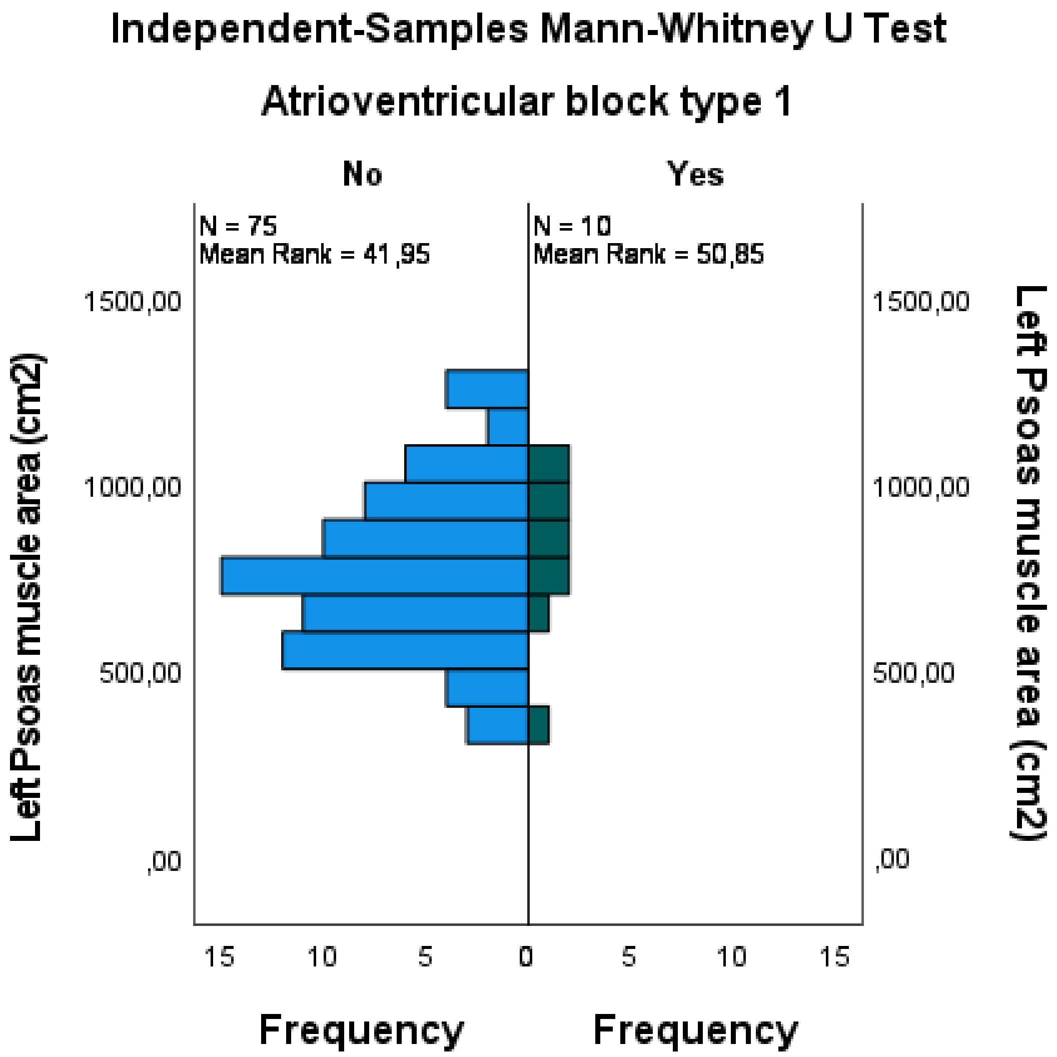

FAT mean density (HU) across Atrioventricular block type 1

Independent-Samples Mann-Whitney U Test Summary

Total N	85
Mann-Whitney U	330,500
Wilcoxon W	385,500
Test Statistic	330,500
Standard Error	73,302
Standardized Test Statistic	-,607
Asymptotic Sig.(2-sided test)	,544

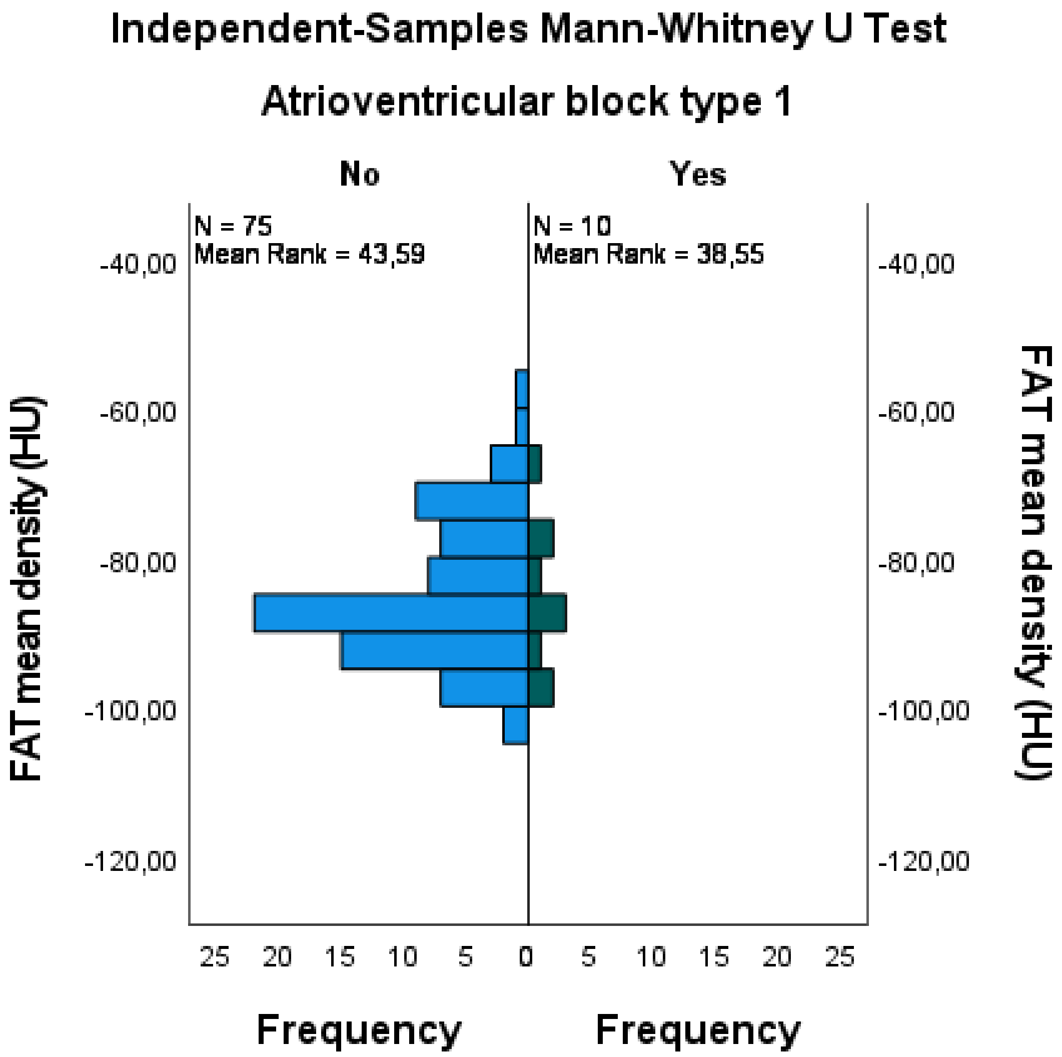

FAT median density (HU) across Atrioventricular block type 1

Independent-Samples Mann-Whitney U Test Summary

Total N	85
Mann-Whitney U	317,500
Wilcoxon W	372,500
Test Statistic	317,500
Standard Error	73,226
Standardized Test Statistic	-,785
Asymptotic Sig.(2-sided test)	,432

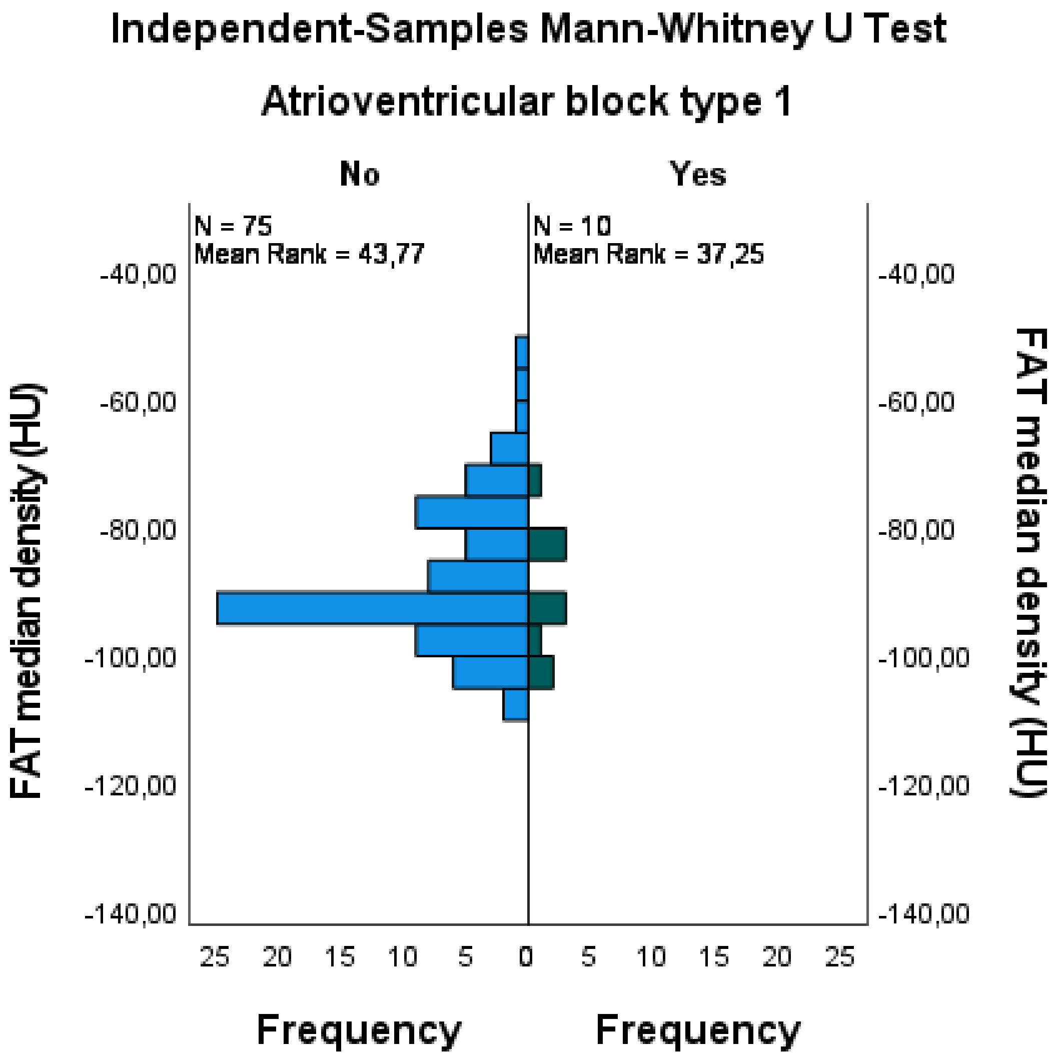

FAT density standard deviation across Atrioventricular block type 1

Independent-Samples Mann-Whitney U Test Summary

Total N	85
Mann-Whitney U	386,000
Wilcoxon W	441,000
Test Statistic	386,000
Standard Error	73,314
Standardized Test Statistic	,150
Asymptotic Sig.(2-sided test)	,881

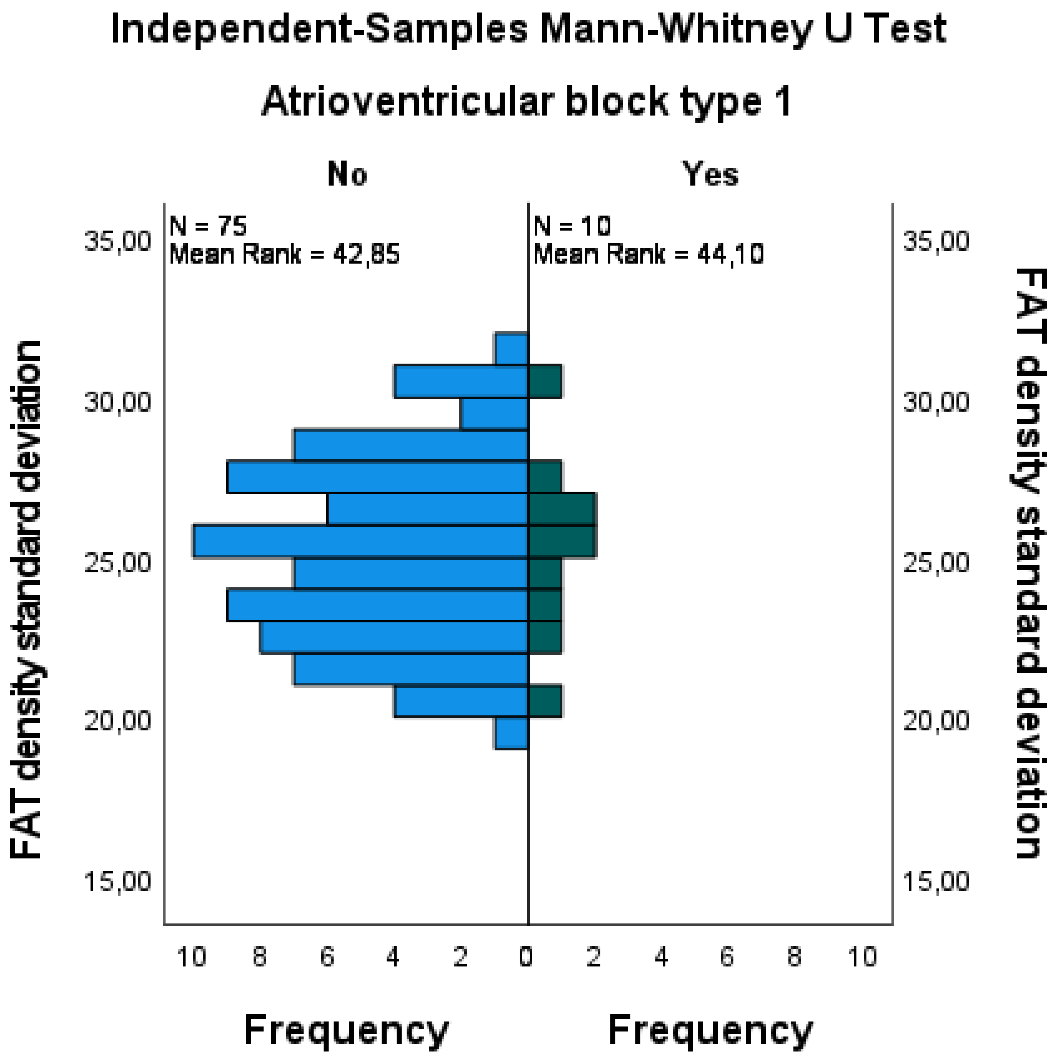

SAT mean density (HU) across Atrioventricular block type 1

Independent-Samples Mann-Whitney U Test Summary

Total N	85
Mann-Whitney U	327,500
Wilcoxon W	382,500
Test Statistic	327,500
Standard Error	73,306
Standardized Test Statistic	-,648
Asymptotic Sig.(2-sided test)	,517

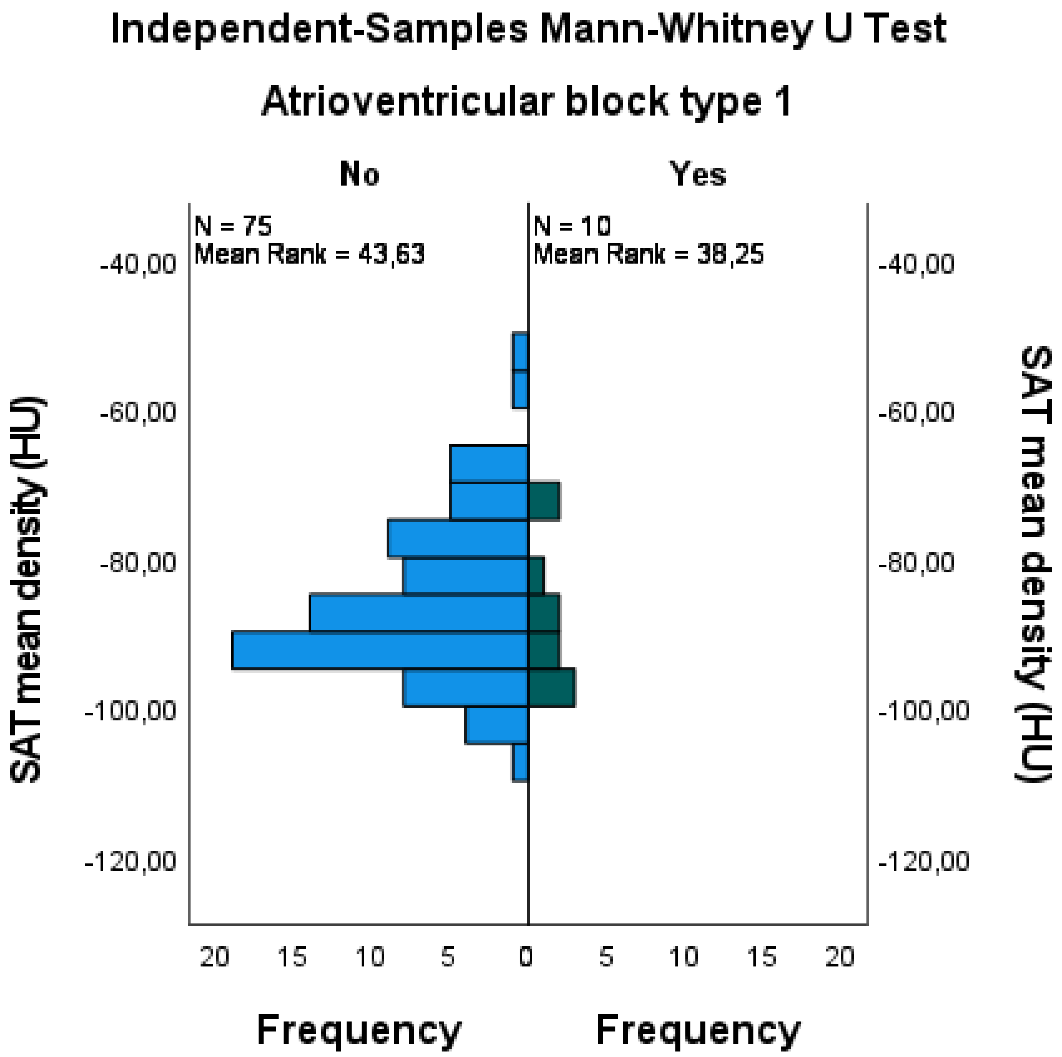

SAT median density (HU) across Atrioventricular block type 1

Independent-Samples Mann-Whitney U Test Summary

Total N	83
Mann-Whitney U	307,000
Wilcoxon W	362,000
Test Statistic	307,000
Standard Error	71,429
Standardized Test Statistic	-,812
Asymptotic Sig.(2-sided test)	,417

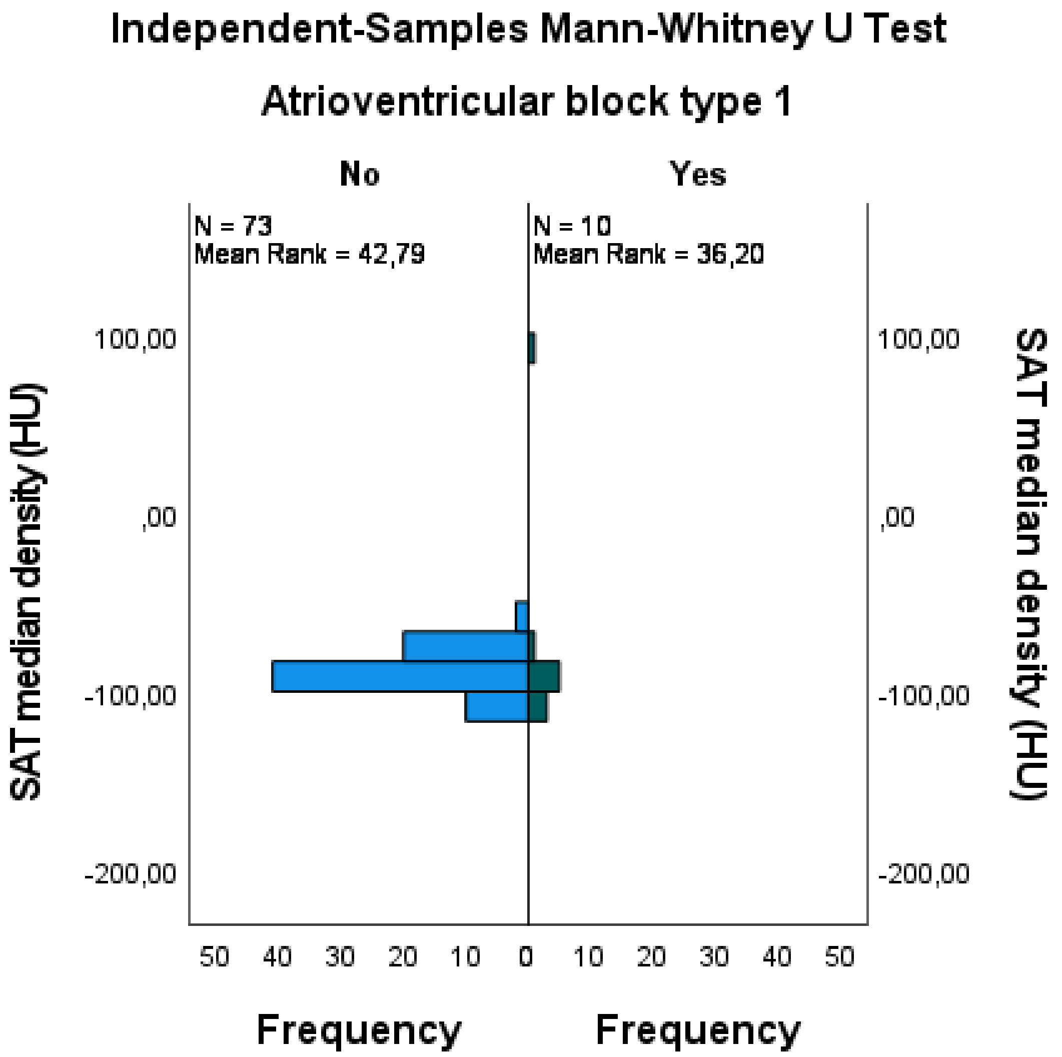

SAT density standard deviation across Atrioventricular block type 1

Independent-Samples Mann-Whitney U Test Summary

Total N	84
Mann-Whitney U	449,000
Wilcoxon W	504,000
Test Statistic	449,000
Standard Error	72,399
Standardized Test Statistic	1,091
Asymptotic Sig.(2-sided test)	,275

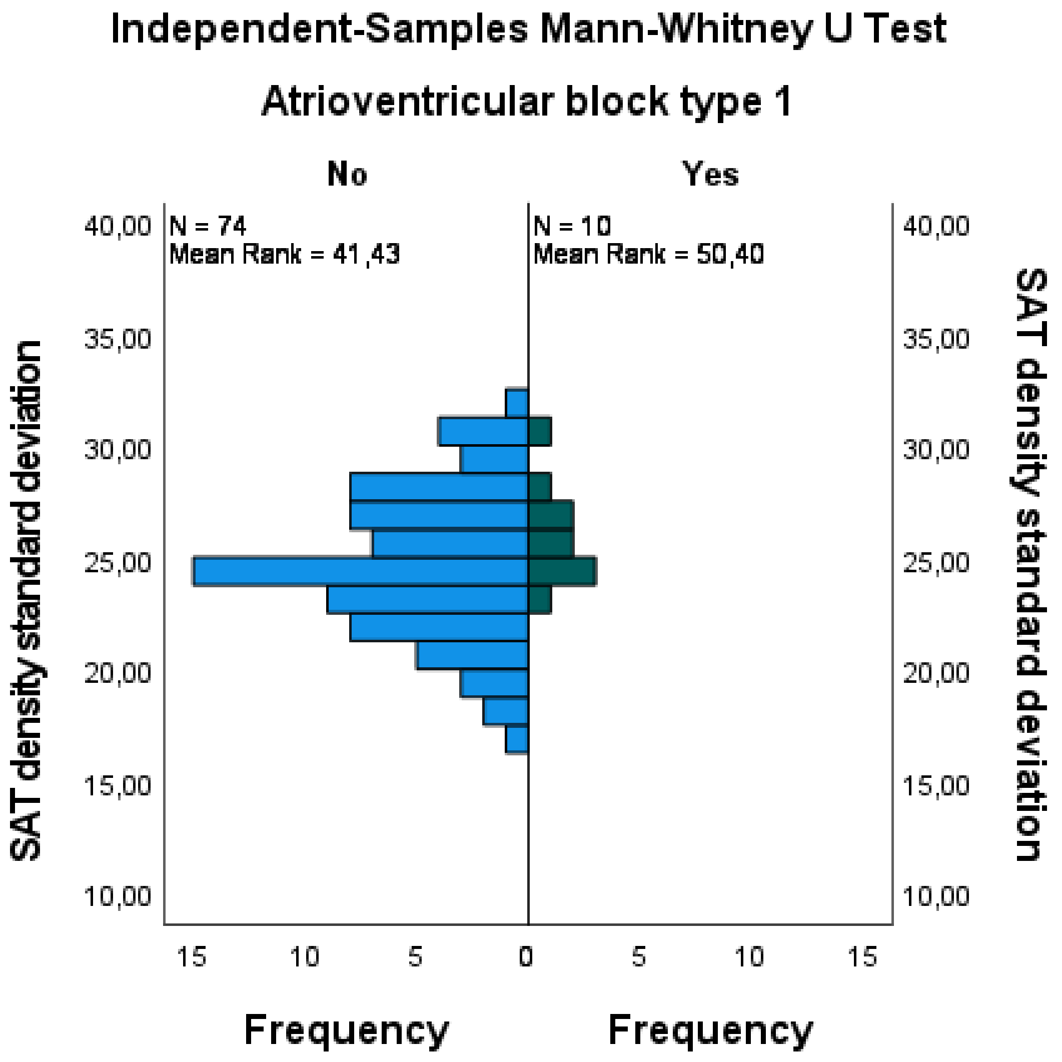

VAT mean density (HU) across Atrioventricular block type 1

Independent-Samples Mann-Whitney U Test Summary

Total N	85
Mann-Whitney U	224,500
Wilcoxon W	279,500
Test Statistic	224,500
Standard Error	73,301
Standardized Test Statistic	-2,053
Asymptotic Sig.(2-sided test)	,040

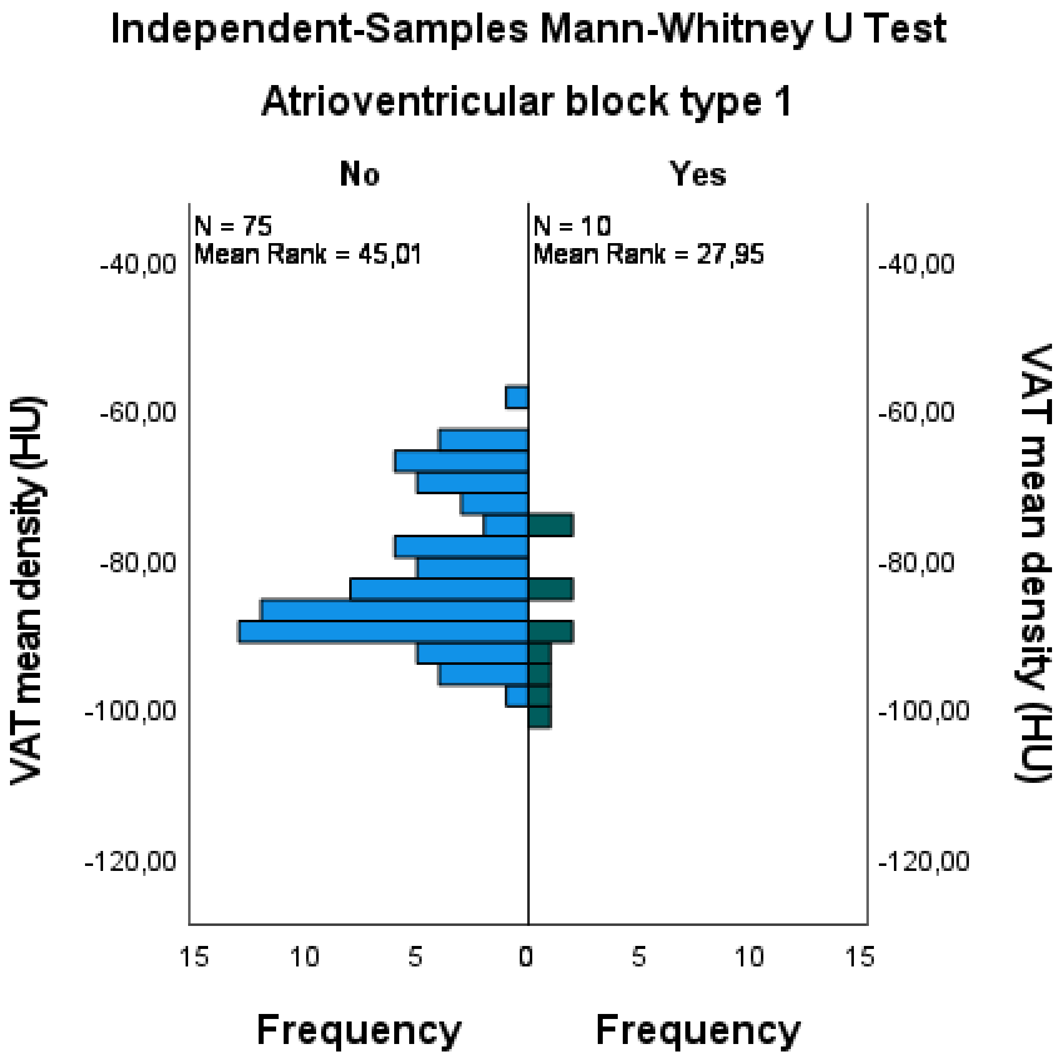

VAT median density (HU) across Atrioventricular block type 1

Independent-Samples Mann-Whitney U Test Summary

Total N	85
Mann-Whitney U	213,500
Wilcoxon W	268,500
Test Statistic	213,500
Standard Error	73,223
Standardized Test Statistic	-2,206
Asymptotic Sig.(2-sided test)	,027

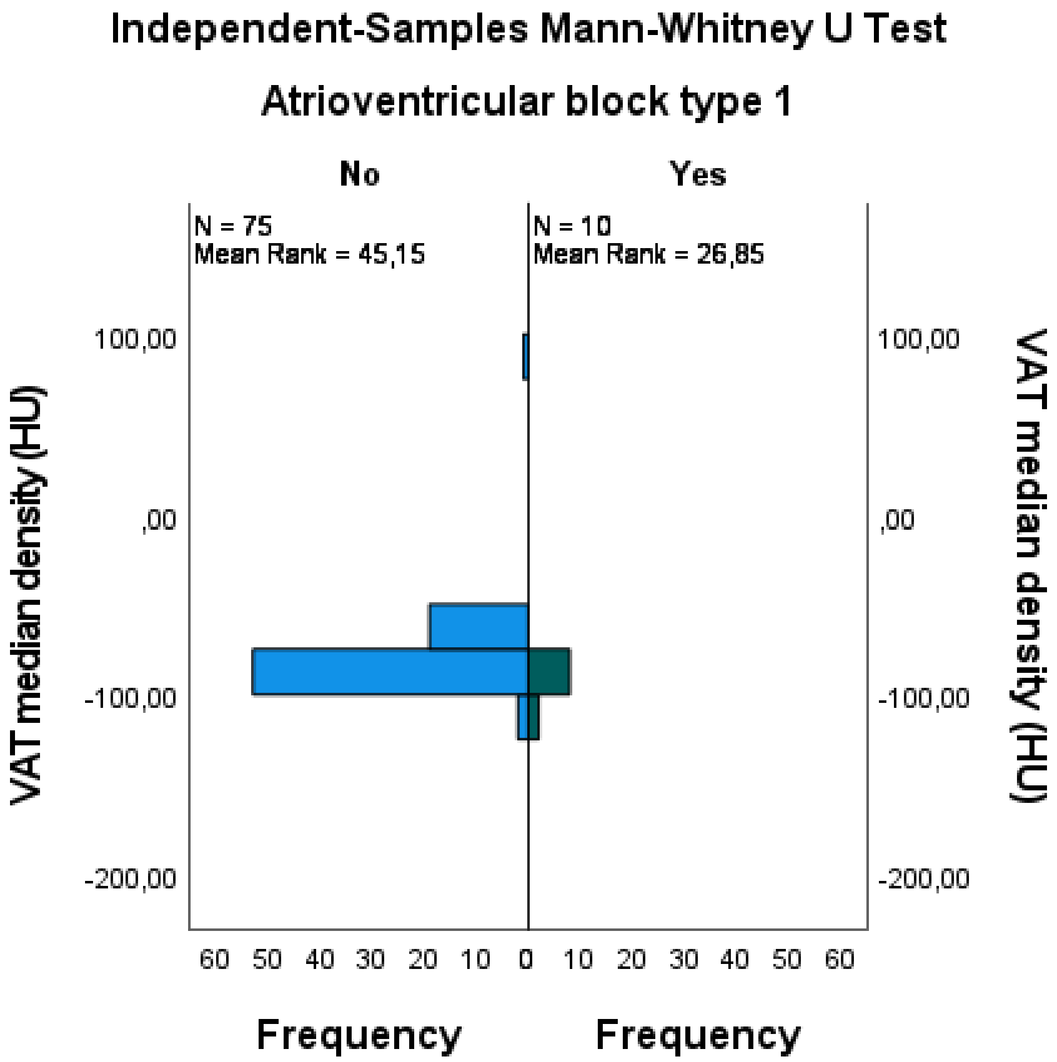

VAT density standard deviation across Atrioventricular block type 1

Independent-Samples Mann-Whitney U Test Summary

Total N	84
Mann-Whitney U	411,000
Wilcoxon W	466,000
Test Statistic	411,000
Standard Error	72,399
Standardized Test Statistic	,566
Asymptotic Sig.(2-sided test)	,571

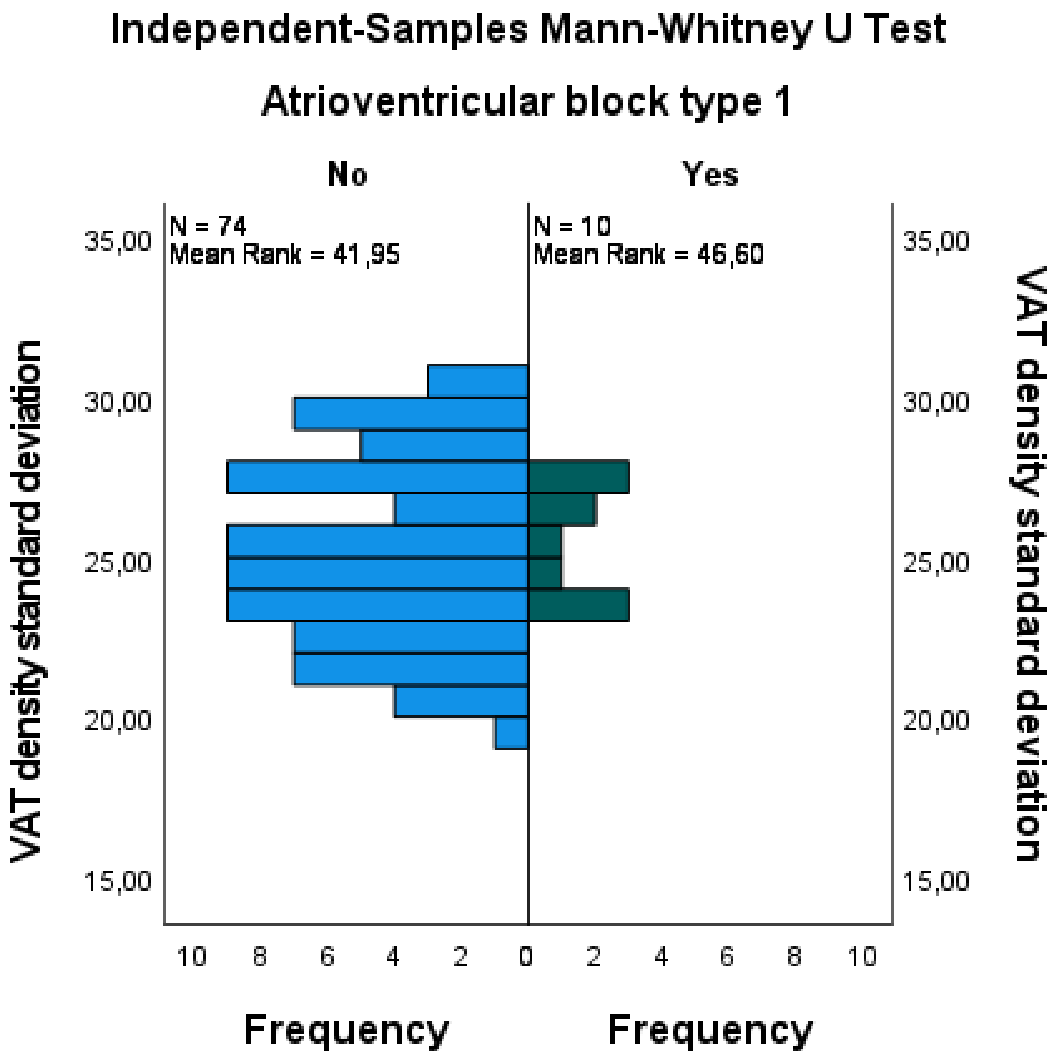

Right Psoas muscle mean density (HU) across Atrioventricular block type 1

Independent-Samples Mann-Whitney U Test Summary

Total N	85
Mann-Whitney U	293,000
Wilcoxon W	348,000
Test Statistic	293,000
Standard Error	73,298
Standardized Test Statistic	-1,119
Asymptotic Sig.(2-sided test)	,263

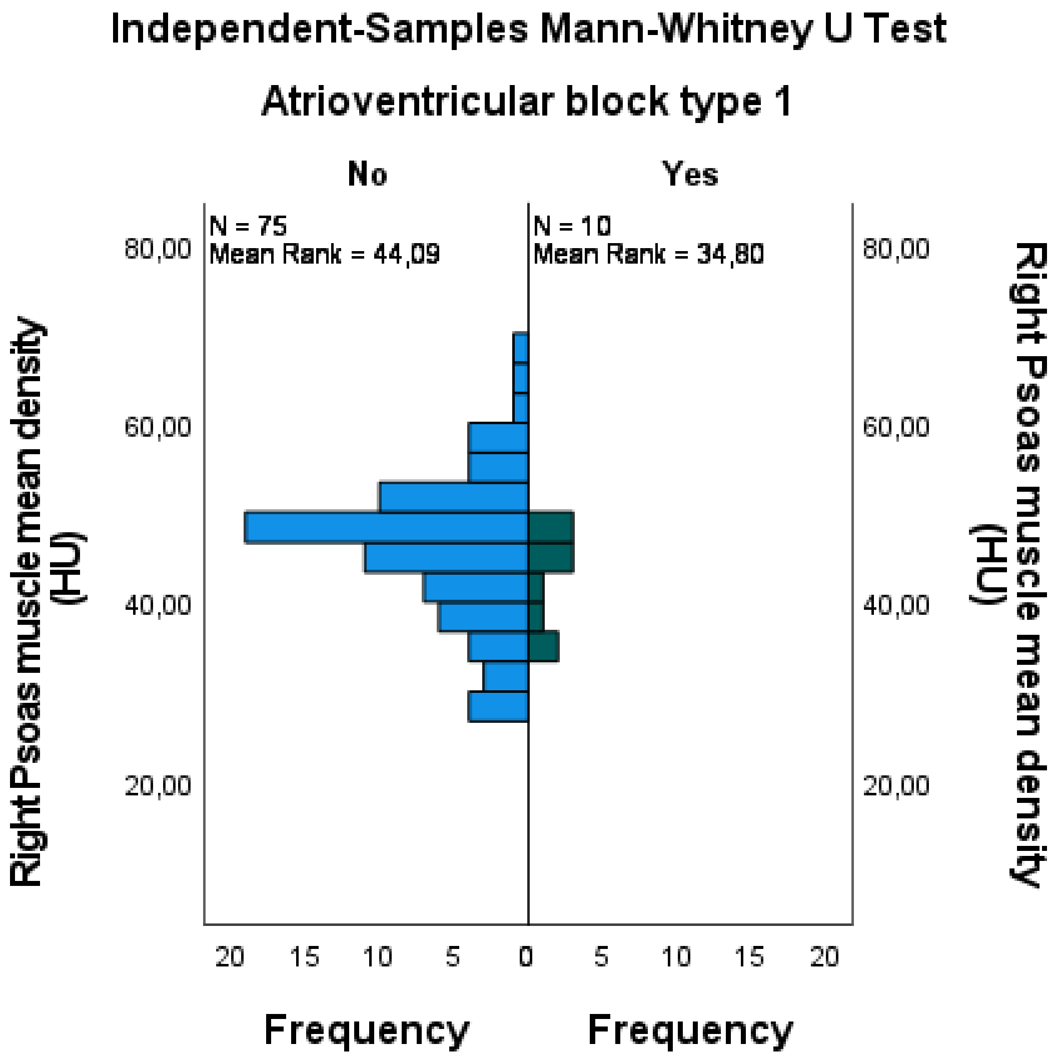

Right Psoas muscle median density (HU) across Atrioventricular block type 1

Independent-Samples Mann-Whitney U Test Summary

Total N	85
Mann-Whitney U	269,500
Wilcoxon W	324,500
Test Statistic	269,500
Standard Error	73,245
Standardized Test Statistic	-1,440
Asymptotic Sig.(2-sided test)	,150

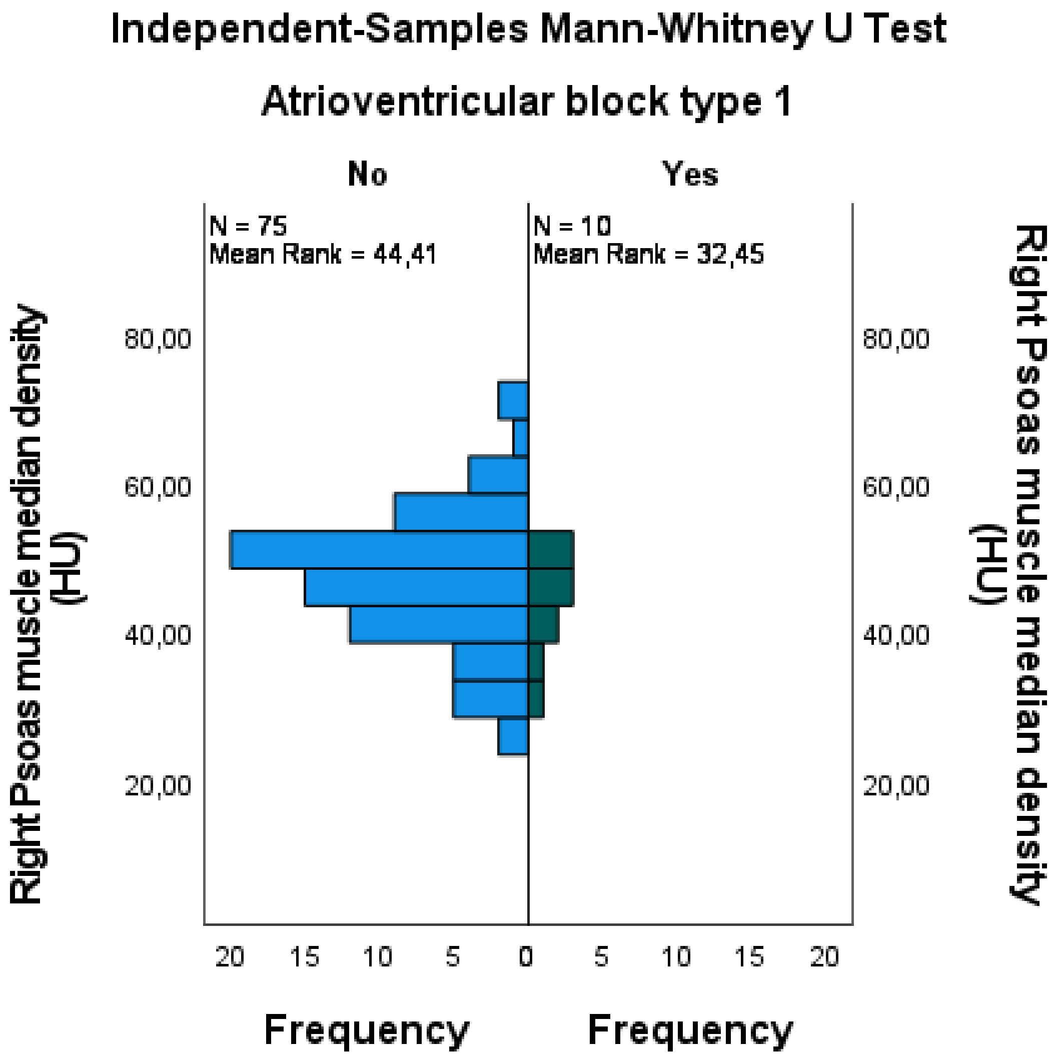

Right Psoas muscle density standard deviation across Atrioventricular block type 1

Independent-Samples Mann-Whitney U Test Summary

Total N	85
Mann-Whitney U	428,500
Wilcoxon W	483,500
Test Statistic	428,500
Standard Error	73,313
Standardized Test Statistic	,730
Asymptotic Sig.(2-sided test)	,466

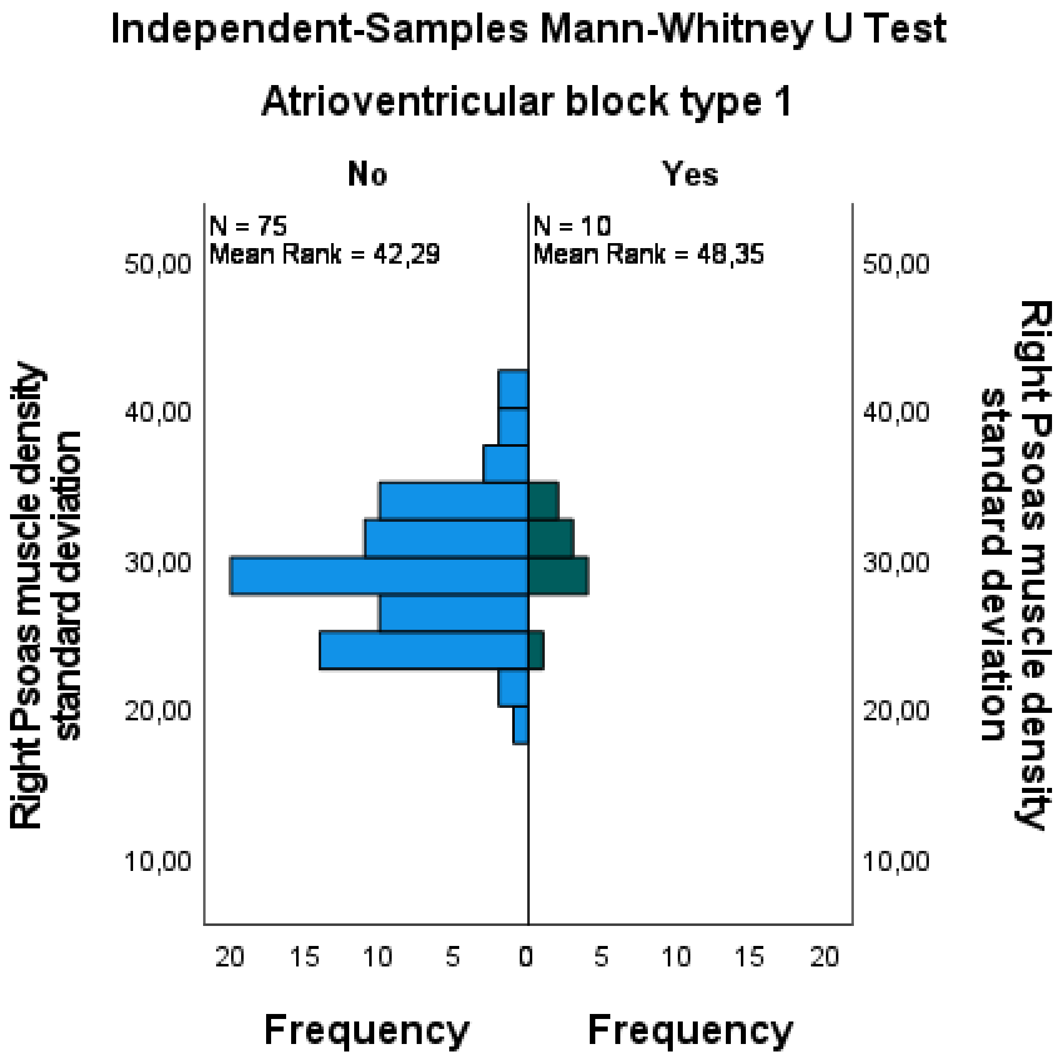

Left Psoas muscle mean density (HU) across Atrioventricular block type 1

Independent-Samples Mann-Whitney U Test Summary

Total N	85
Mann-Whitney U	354,000
Wilcoxon W	409,000
Test Statistic	354,000
Standard Error	73,299
Standardized Test Statistic	-,286
Asymptotic Sig.(2-sided test)	,774

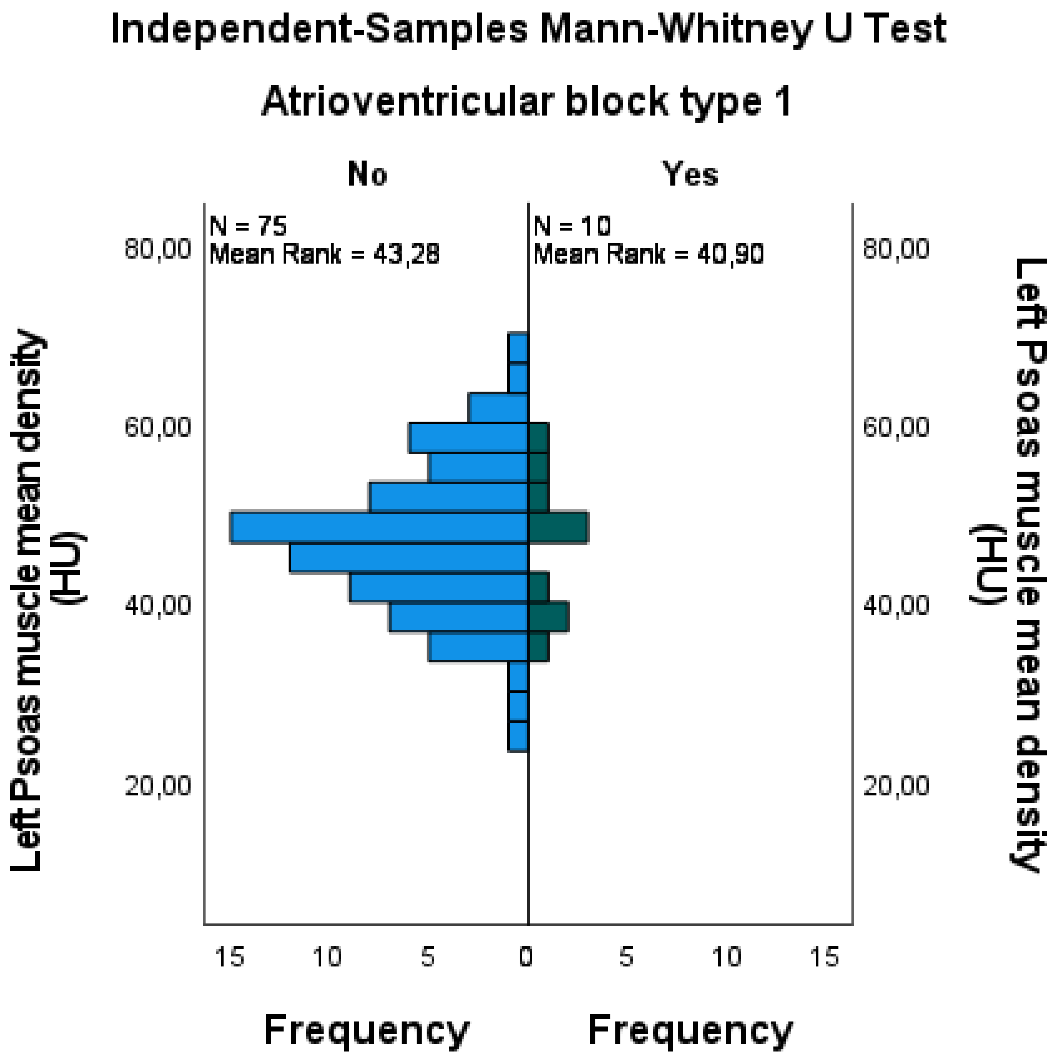

Left Psoas muscle median density (HU) across Atrioventricular block type 1

Independent-Samples Mann-Whitney U Test Summary

Total N	85
Mann-Whitney U	355,000
Wilcoxon W	410,000
Test Statistic	355,000
Standard Error	73,221
Standardized Test Statistic	-,273
Asymptotic Sig.(2-sided test)	,785

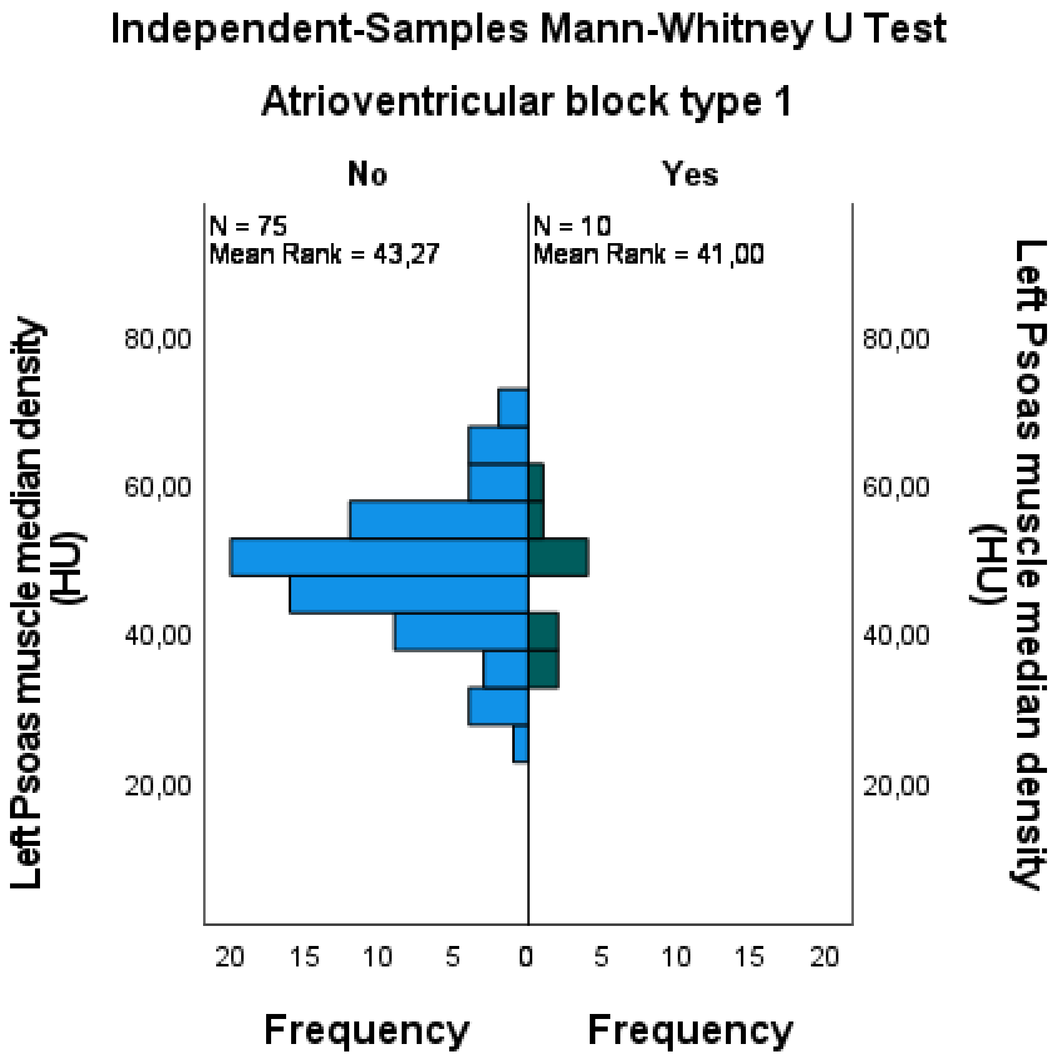

Left Psoas muscle density standard deviation across Atrioventricular block type 1

Independent-Samples Mann-Whitney U Test Summary

Total N	85
Mann-Whitney U	395,000
Wilcoxon W	450,000
Test Statistic	395,000
Standard Error	73,314
Standardized Test Statistic	,273
Asymptotic Sig.(2-sided test)	,785

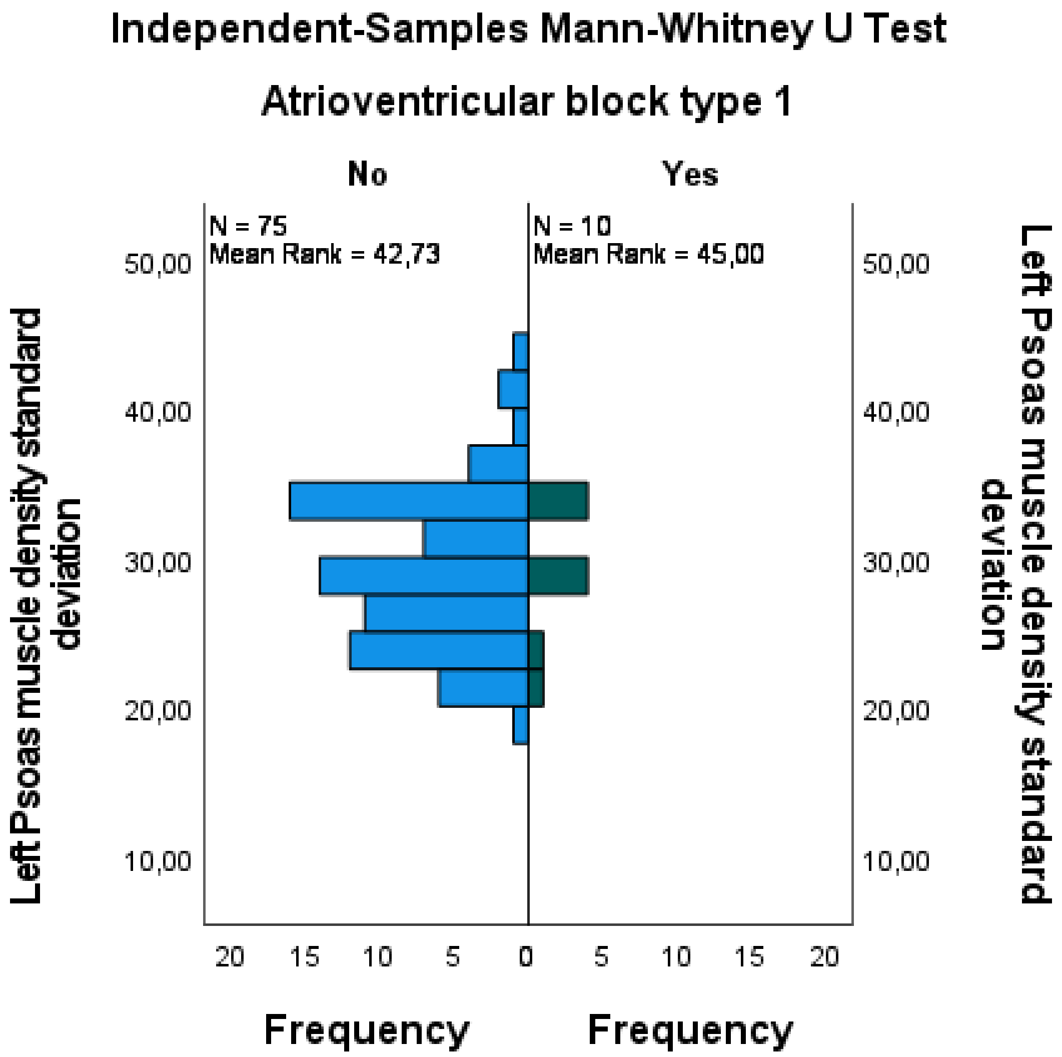

Independent-Samples Mann-Whitney U Test for atrioventricular block type 2 occurrence

Hypothesis Test Summary

	Null Hypothesis	Test	Sig.[a,b]	Decision
1	The distribution of Psoas/height is the same across categories of Atrioventricular block type 2.	Independent-Samples Mann-Whitney U Test	,182[c]	Retain the null hypothesis.
2	The distribution of Anterior SAT distance is the same across categories of Atrioventricular block type 2.	Independent-Samples Mann-Whitney U Test	,965[c]	Retain the null hypothesis.
3	The distribution of Posterior SAT distance is the same across categories of Atrioventricular block type 2.	Independent-Samples Mann-Whitney U Test	,247[c]	Retain the null hypothesis.
4	The distribution of Anterior+Posterior SAT distance is the same across categories of Atrioventricular block type 2.	Independent-Samples Mann-Whitney U Test	,504[c]	Retain the null hypothesis.
5	The distribution of VAT distance is the same across categories of Atrioventricular block type 2.	Independent-Samples Mann-Whitney U Test	,918[c]	Retain the null hypothesis.
6	The distribution of Right common femoral artery area (mm2) is the same across categories of Atrioventricular block type 2.	Independent-Samples Mann-Whitney U Test	,809[c]	Retain the null hypothesis.
7	The distribution of Left common femoral artery area (mm2) is the same across categories of Atrioventricular block type 2.	Independent-Samples Mann-Whitney U Test	,538[c]	Retain the null hypothesis.
8	The distribution of FAT area (cm2) is the same across categories of Atrioventricular block type 2.	Independent-Samples Mann-Whitney U Test	,202[c]	Retain the null hypothesis.
9	The distribution of SAT area (cm2) is the same across categories of Atrioventricular block type 2.	Independent-Samples Mann-Whitney U Test	,706[c]	Retain the null hypothesis.

10	The distribution of VAT area (cm2) is the same across categories of Atrioventricular block type 2.	Independent-Samples Mann-Whitney U Test	,057[c]	Retain the null hypothesis.
11	The distribution of Right Psoas muscle area (cm2) is the same across categories of Atrioventricular block type 2.	Independent-Samples Mann-Whitney U Test	,896[c]	Retain the null hypothesis.
12	The distribution of Left Psoas muscle area (cm2) is the same across categories of Atrioventricular block type 2.	Independent-Samples Mann-Whitney U Test	,767[c]	Retain the null hypothesis.
13	The distribution of FAT mean density (HU) is the same across categories of Atrioventricular block type 2.	Independent-Samples Mann-Whitney U Test	,408[c]	Retain the null hypothesis.
14	The distribution of FAT median density (HU) is the same across categories of Atrioventricular block type 2.	Independent-Samples Mann-Whitney U Test	,350[c]	Retain the null hypothesis.
15	The distribution of FAT density standard deviation is the same across categories of Atrioventricular block type 2.	Independent-Samples Mann-Whitney U Test	,247[c]	Retain the null hypothesis.
16	The distribution of SAT mean density (HU) is the same across categories of Atrioventricular block type 2.	Independent-Samples Mann-Whitney U Test	,336[c]	Retain the null hypothesis.
17	The distribution of SAT median density (HU) is the same across categories of Atrioventricular block type 2.	Independent-Samples Mann-Whitney U Test	,311[c]	Retain the null hypothesis.
18	The distribution of SAT density standard deviation is the same across categories of Atrioventricular block type 2.	Independent-Samples Mann-Whitney U Test	,317[c]	Retain the null hypothesis.
19	The distribution of VAT mean density (HU) is the same across categories of Atrioventricular block type 2.	Independent-Samples Mann-Whitney U Test	,087[c]	Retain the null hypothesis.

20	The distribution of VAT median density (HU) is the same across categories of Atrioventricular block type 2.	Independent-Samples Mann-Whitney U Test	,095[c]	Retain the null hypothesis.
21	The distribution of VAT density standard deviation is the same across categories of Atrioventricular block type 2.	Independent-Samples Mann-Whitney U Test	,265[c]	Retain the null hypothesis.
22	The distribution of Right Psoas muscle mean density (HU) is the same across categories of Atrioventricular block type 2.	Independent-Samples Mann-Whitney U Test	,045[c]	Reject the null hypothesis.
23	The distribution of Right Psoas muscle median density (HU) is the same across categories of Atrioventricular block type 2.	Independent-Samples Mann-Whitney U Test	,056[c]	Retain the null hypothesis.
24	The distribution of Right Psoas muscle density standard deviation is the same across categories of Atrioventricular block type 2.	Independent-Samples Mann-Whitney U Test	,686[c]	Retain the null hypothesis.
25	The distribution of Left Psoas muscle mean density (HU) is the same across categories of Atrioventricular block type 2.	Independent-Samples Mann-Whitney U Test	,102[c]	Retain the null hypothesis.
26	The distribution of Left Psoas muscle median density (HU) is the same across categories of Atrioventricular block type 2.	Independent-Samples Mann-Whitney U Test	,056[c]	Retain the null hypothesis.
27	The distribution of Left Psoas muscle density standard deviation is the same across categories of Atrioventricular block type 2.	Independent-Samples Mann-Whitney U Test	,629[c]	Retain the null hypothesis.

a. The significance level is ,050.

b. Asymptotic significance is displayed.

c. Exact significance is displayed for this test.

In this case, the hypothesis of equal medians ($p < 0.05$) is rejected only for the Right Psoas muscle mean density (HU) variable, while for all the others the null hypothesis is accepted ($p > 0.05$).

(The tables and graphs below are the details of the tests in this table: I have highlighted what things you should eventually report, namely test statistic and pvalue).

Psoas/height across Atrioventricular block type 2

Independent-Samples Mann-Whitney U Test Summary

Total N	85
Mann-Whitney U	34,000
Wilcoxon W	37,000
Test Statistic	34,000
Standard Error	34,491
Standardized Test Statistic	-1,421
Asymptotic Sig.(2-sided test)	,155
Exact Sig.(2-sided test)	,182

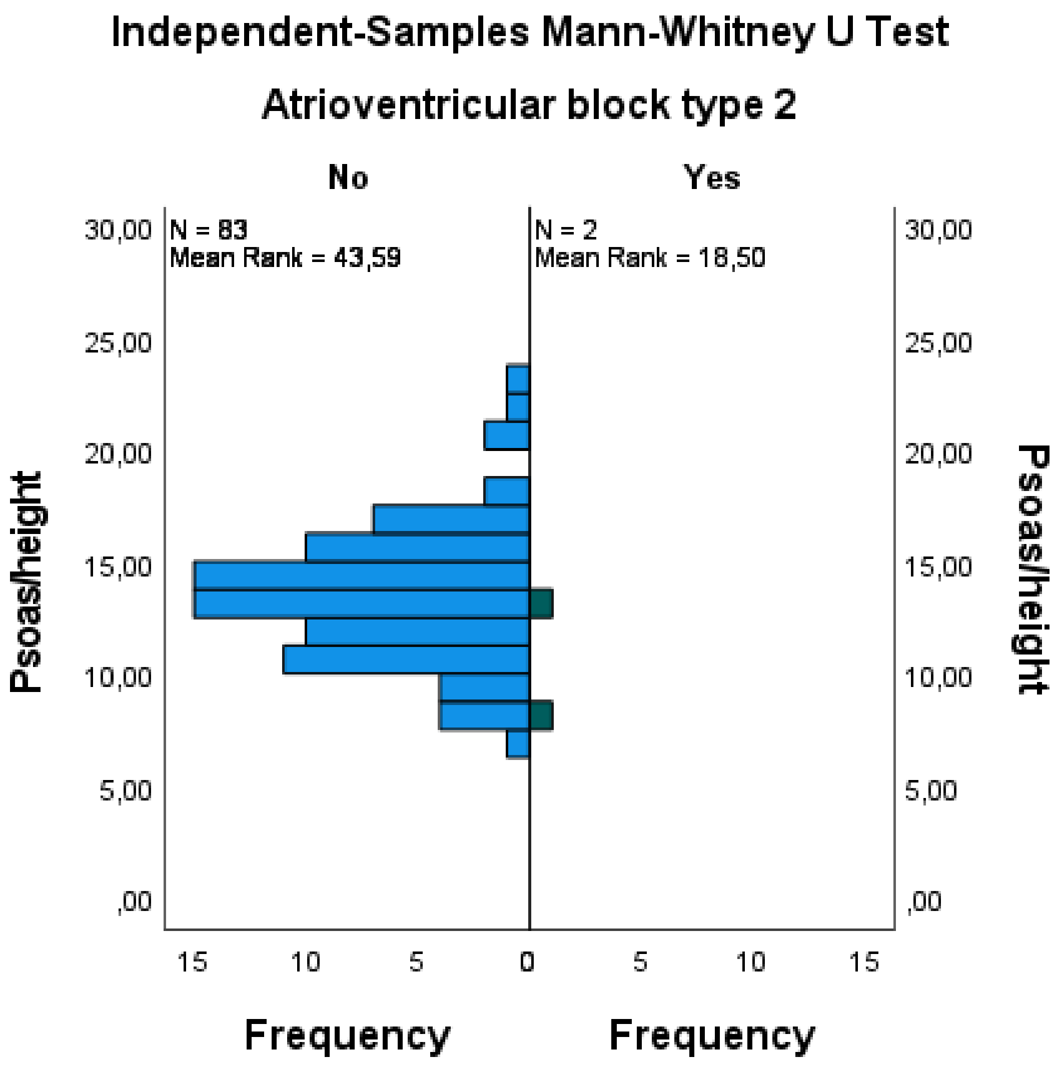

Anterior SAT distance across Atrioventricular block type 2

Independent-Samples Mann-Whitney U Test Summary

Total N	85
Mann-Whitney U	81,500
Wilcoxon W	84,500
Test Statistic	81,500
Standard Error	34,487
Standardized Test Statistic	-,043
Asymptotic Sig.(2-sided test)	,965
Exact Sig.(2-sided test)	,965

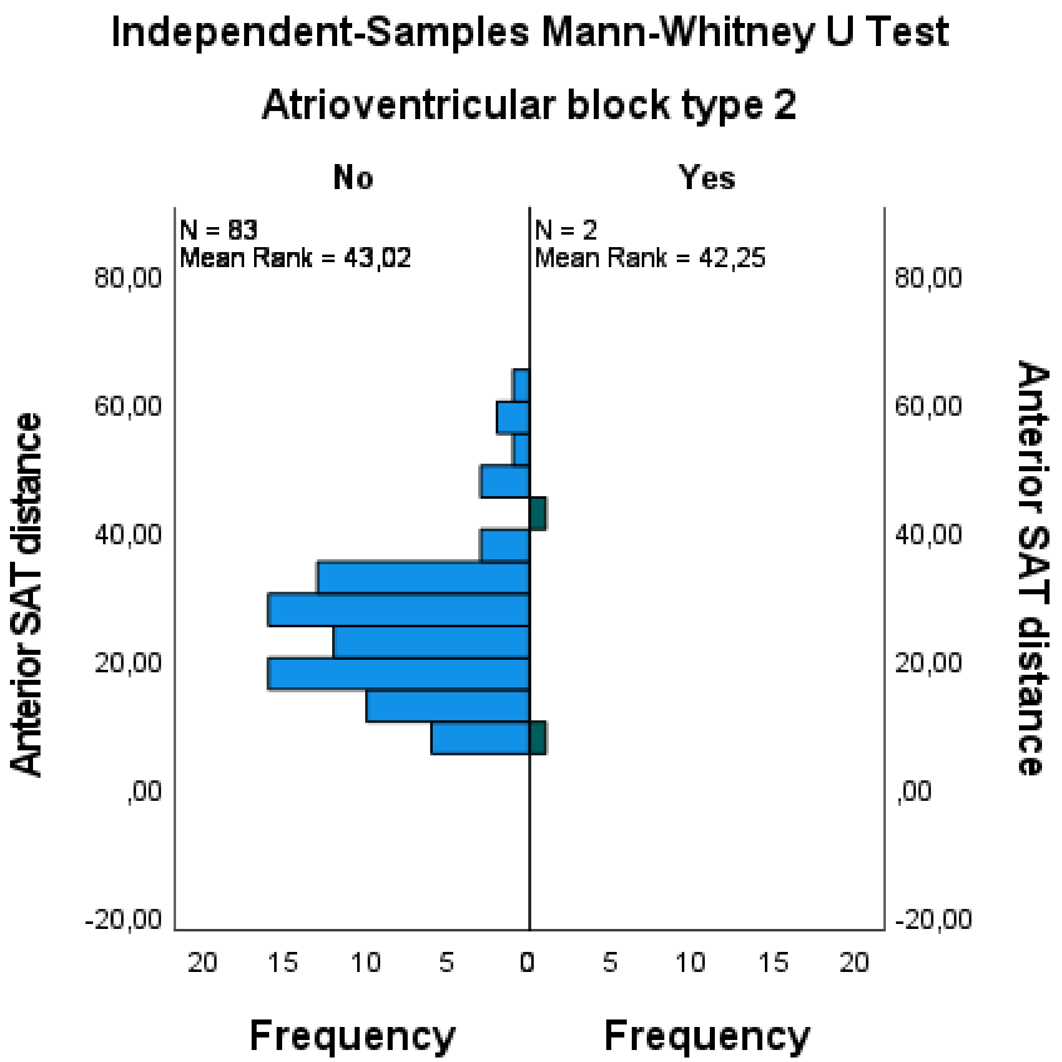

Posterior SAT distance across Atrioventricular block type 2

Independent-Samples Mann-Whitney U Test Summary

Total N	85
Mann-Whitney U	40,000
Wilcoxon W	43,000
Test Statistic	40,000
Standard Error	34,488
Standardized Test Statistic	-1,247
Asymptotic Sig.(2-sided test)	,212
Exact Sig.(2-sided test)	,247

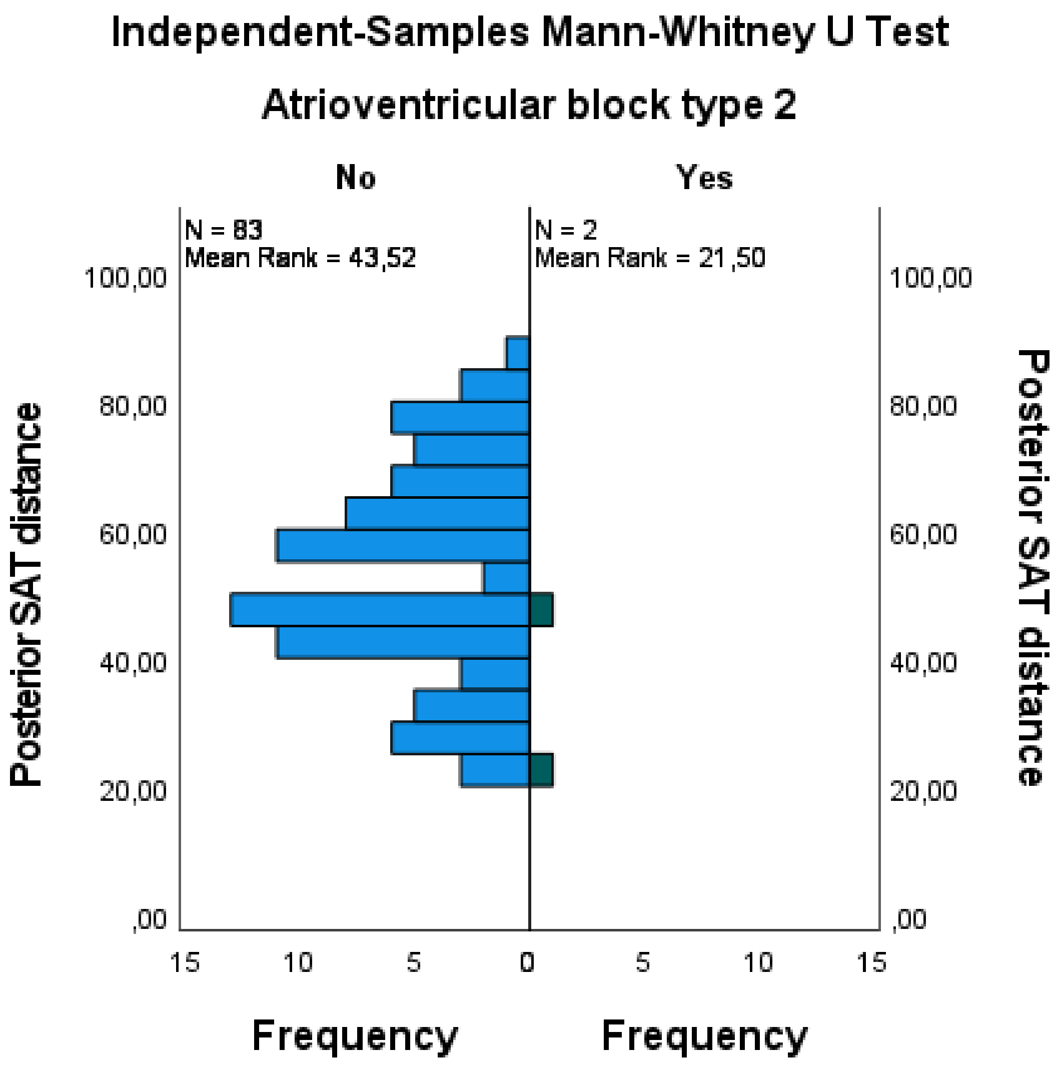

Anterior+Posterior SAT distance across Atrioventricular block type 2

Independent-Samples Mann-Whitney U Test Summary

Total N	85
Mann-Whitney U	58,500
Wilcoxon W	61,500
Test Statistic	58,500
Standard Error	34,490
Standardized Test Statistic	-,710
Asymptotic Sig.(2-sided test)	,477
Exact Sig.(2-sided test)	,504

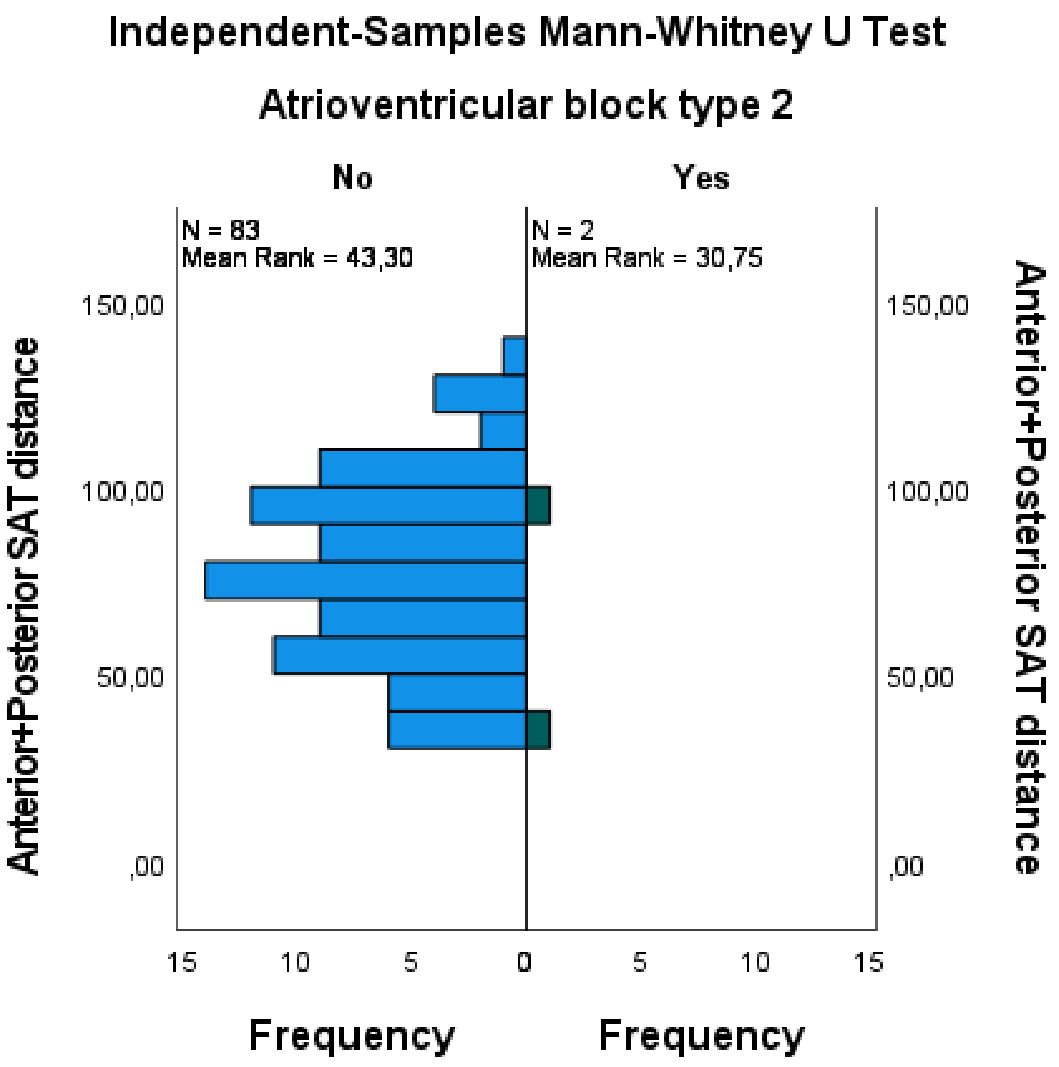

VAT distance across Atrioventricular block type 2

Independent-Samples Mann-Whitney U Test Summary

Total N	84
Mann-Whitney U	78,000
Wilcoxon W	81,000
Test Statistic	78,000
Standard Error	34,076
Standardized Test Statistic	-,117
Asymptotic Sig.(2-sided test)	,907
Exact Sig.(2-sided test)	,918

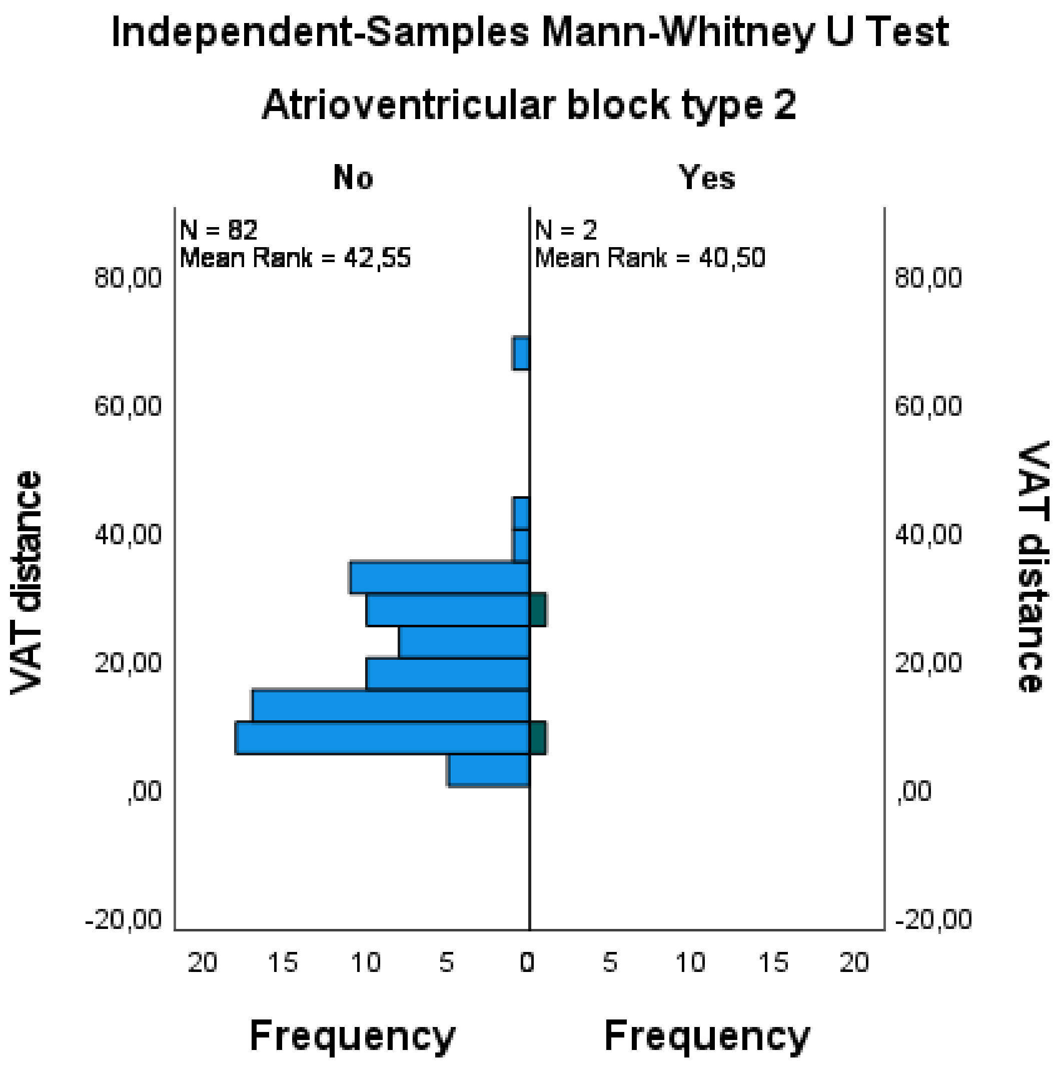

Right common femoral artery area (mm2) across Atrioventricular block type 2

Independent-Samples Mann-Whitney U Test Summary

Total N	85
Mann-Whitney U	74,500
Wilcoxon W	77,500
Test Statistic	74,500
Standard Error	34,486
Standardized Test Statistic	-,246
Asymptotic Sig.(2-sided test)	,805
Exact Sig.(2-sided test)	,809

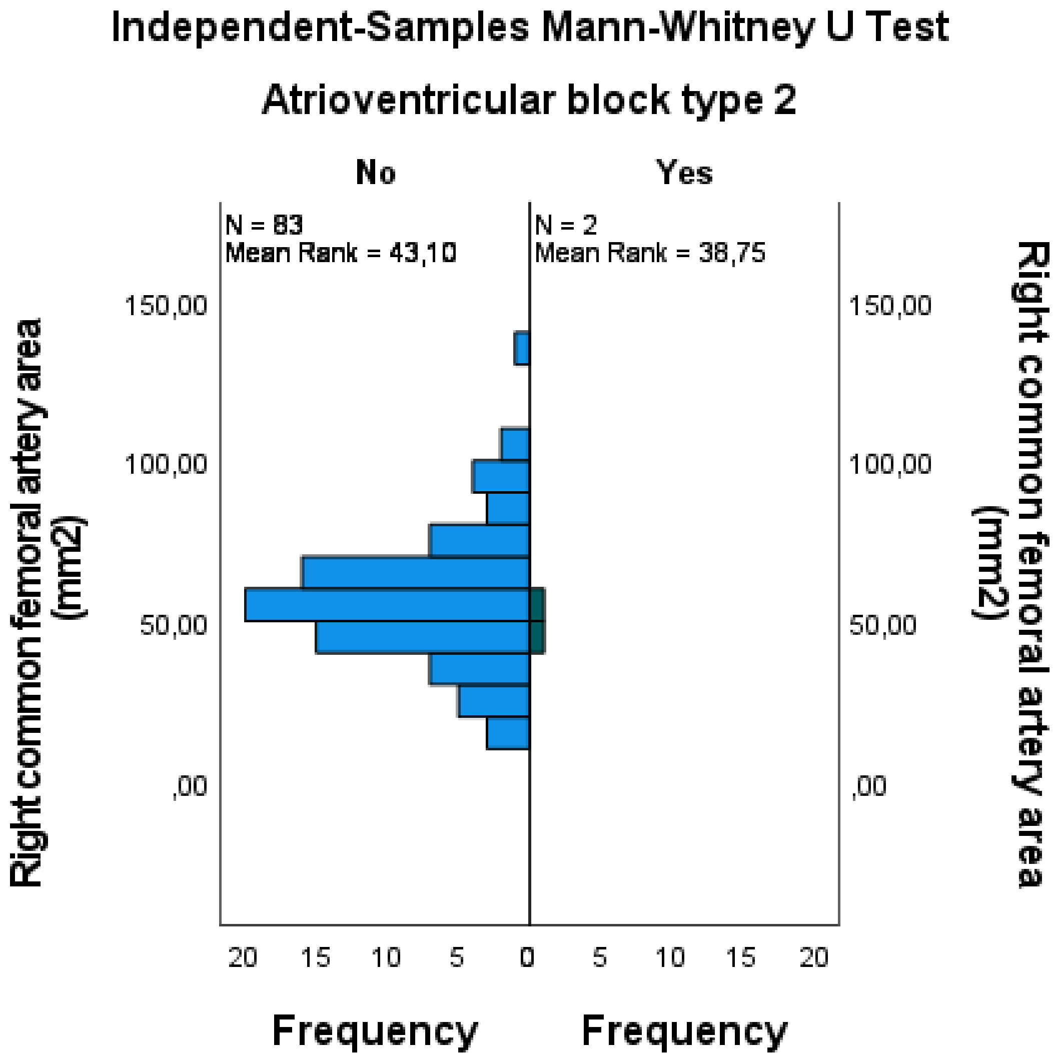

Left common femoral artery area (mm2) across Atrioventricular block type 2

Independent-Samples Mann-Whitney U Test Summary

Total N	85
Mann-Whitney U	106,000
Wilcoxon W	109,000
Test Statistic	106,000
Standard Error	34,483
Standardized Test Statistic	,667
Asymptotic Sig.(2-sided test)	,505
Exact Sig.(2-sided test)	,538

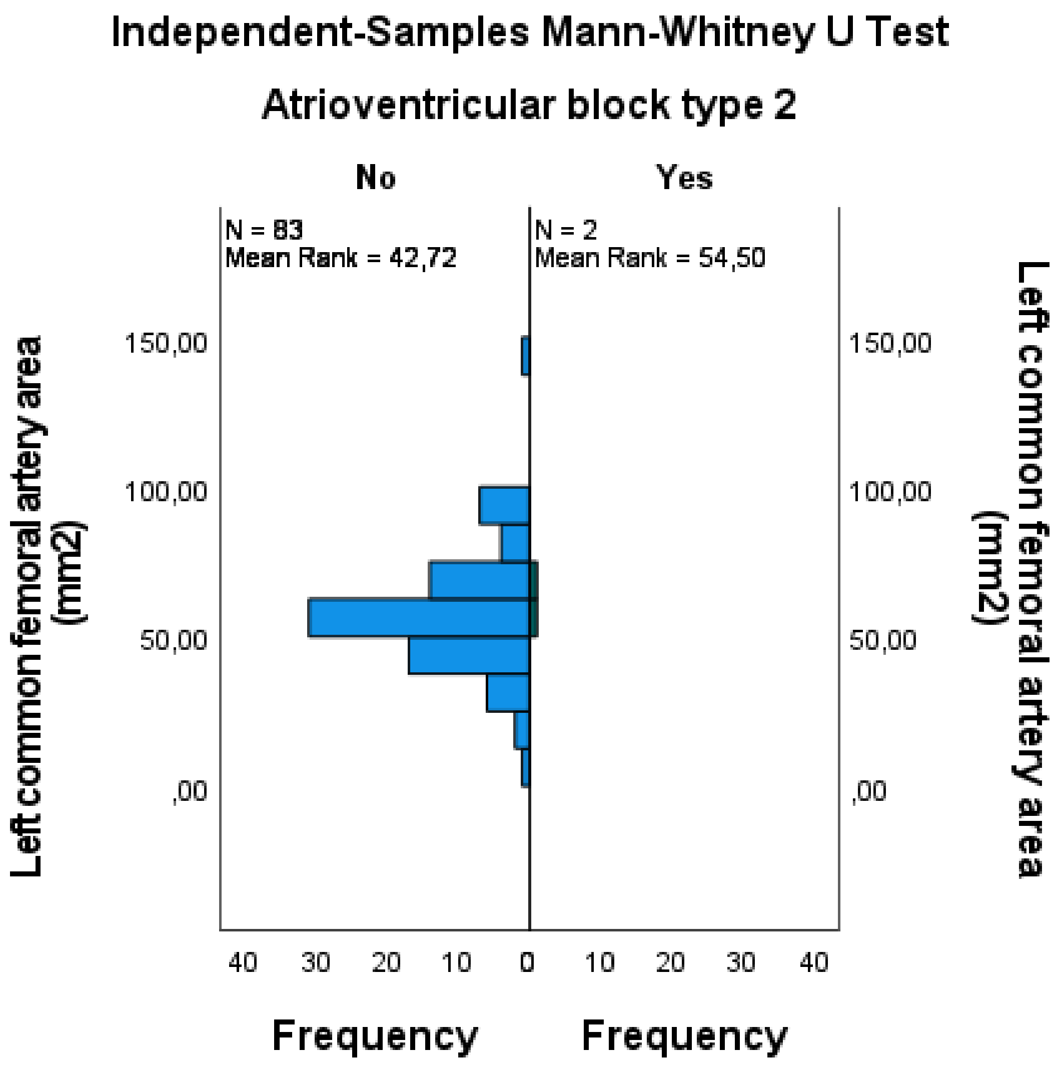

FAT area (cm2) across Atrioventricular block type 2

Independent-Samples Mann-Whitney U Test Summary

Total N	85
Mann-Whitney U	130,000
Wilcoxon W	133,000
Test Statistic	130,000
Standard Error	34,491
Standardized Test Statistic	1,363
Asymptotic Sig.(2-sided test)	,173
Exact Sig.(2-sided test)	,202

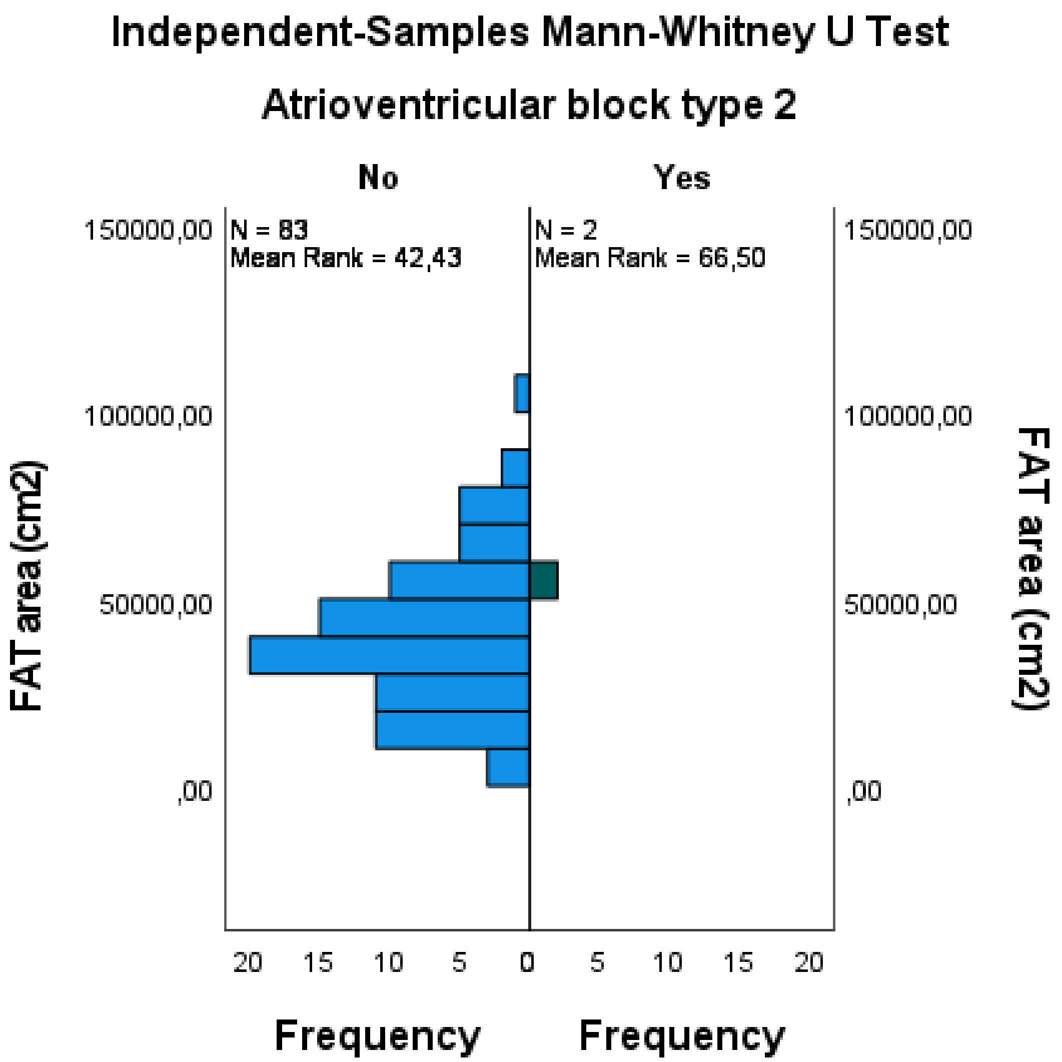

SAT area (cm2) across Atrioventricular block type 2

Independent-Samples Mann-Whitney U Test Summary

Total N	85
Mann-Whitney U	97,000
Wilcoxon W	100,000
Test Statistic	97,000
Standard Error	34,491
Standardized Test Statistic	,406
Asymptotic Sig.(2-sided test)	,685
Exact Sig.(2-sided test)	,706

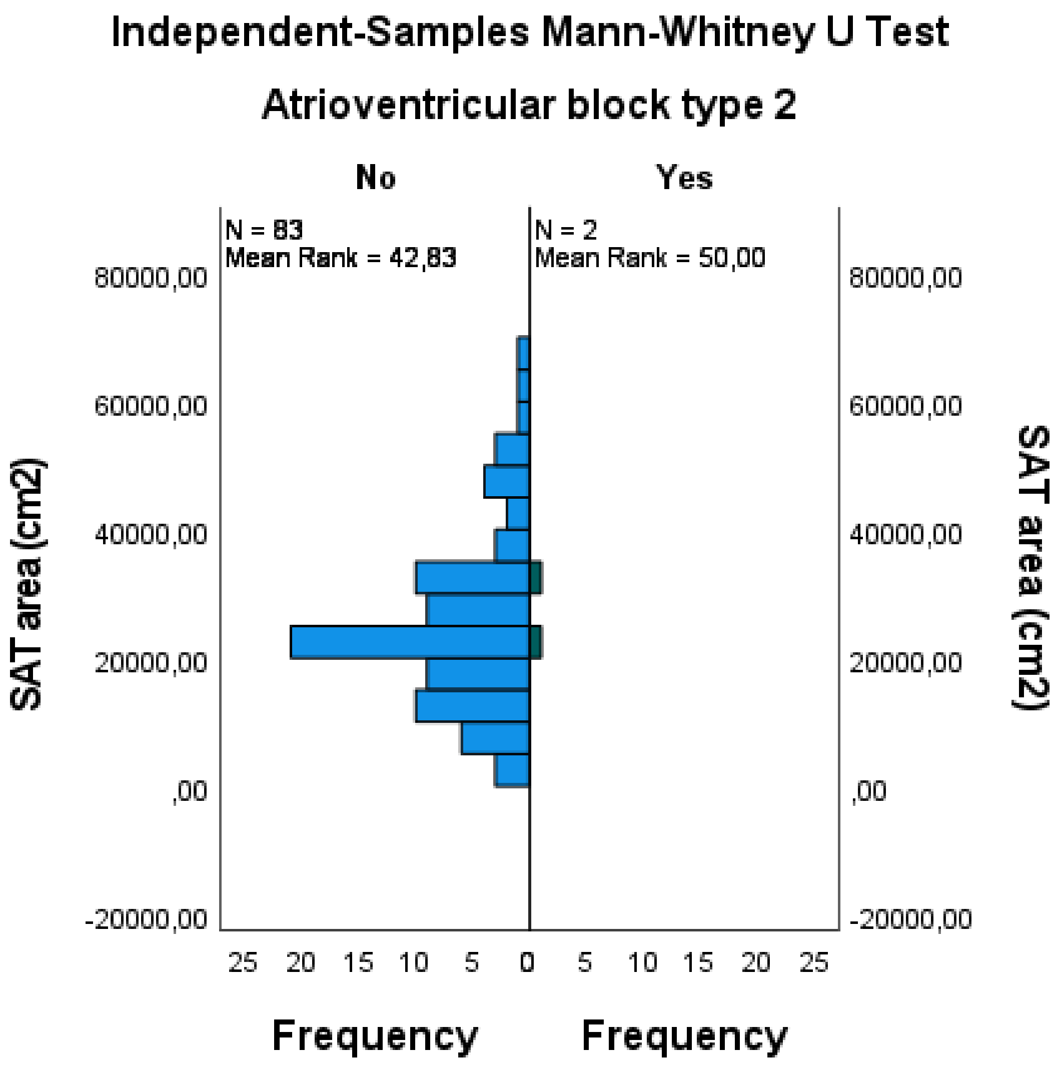

VAT area (cm2) across Atrioventricular block type 2

Independent-Samples Mann-Whitney U Test Summary

Total N	84
Mann-Whitney U	146,000
Wilcoxon W	149,000
Test Statistic	146,000
Standard Error	34,083
Standardized Test Statistic	1,878
Asymptotic Sig.(2-sided test)	,060
Exact Sig.(2-sided test)	,057

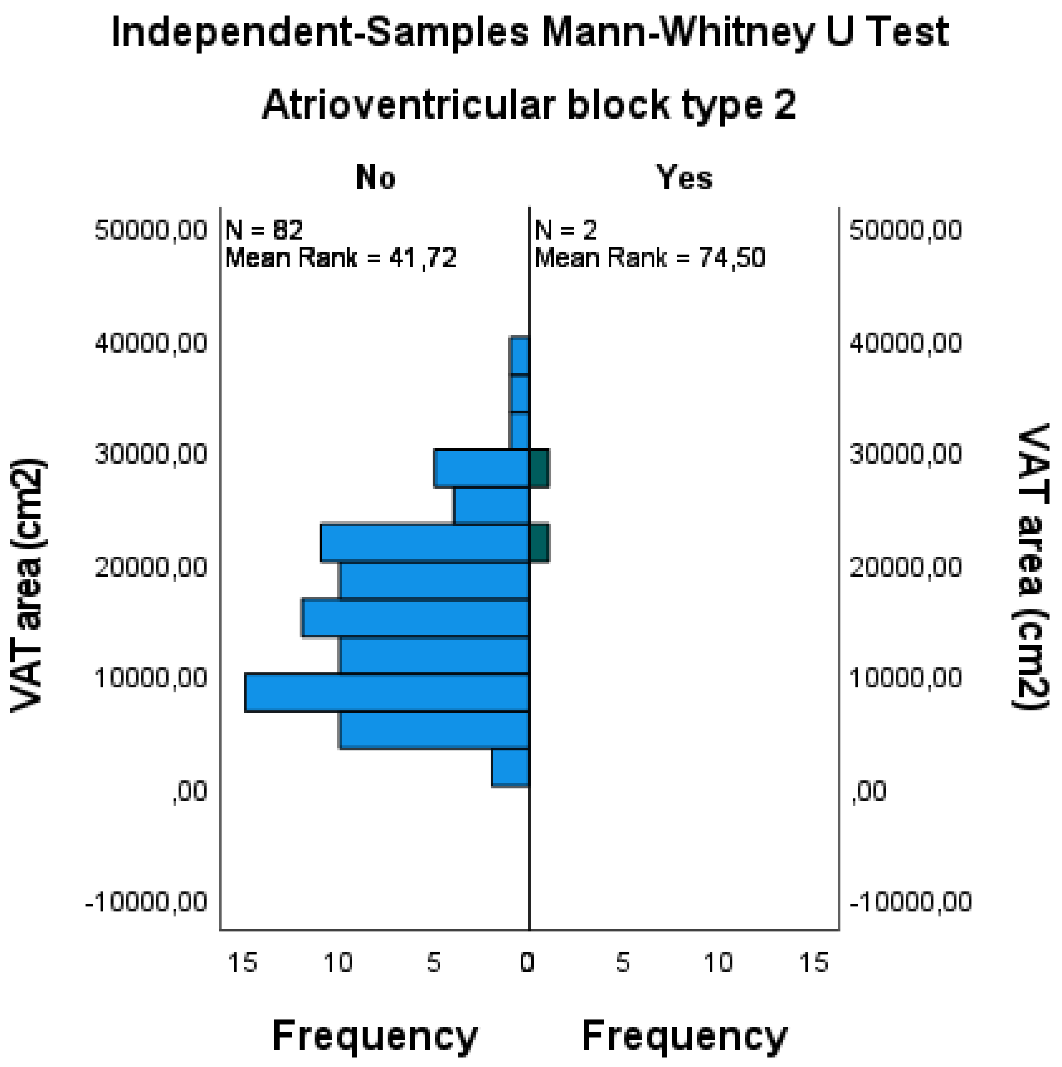

Right Psoas muscle area (cm2) across Atrioventricular block type 2

Independent-Samples Mann-Whitney U Test Summary

Total N	85
Mann-Whitney U	78,000
Wilcoxon W	81,000
Test Statistic	78,000
Standard Error	34,491
Standardized Test Statistic	-,145
Asymptotic Sig.(2-sided test)	,885
Exact Sig.(2-sided test)	,896

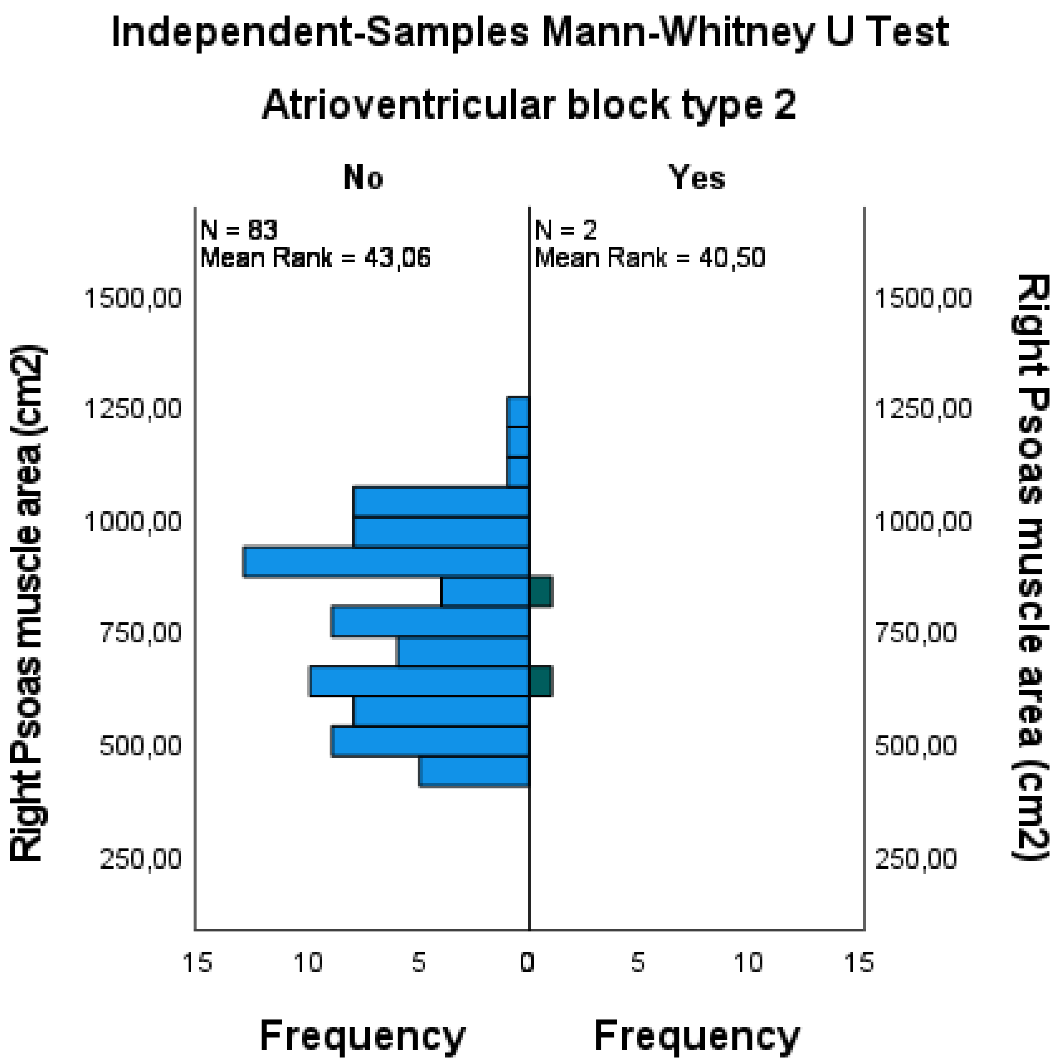

Left Psoas muscle area (cm2) across Atrioventricular block type 2

Independent-Samples Mann-Whitney U Test Summary

Total N	85
Mann-Whitney U	94,000
Wilcoxon W	97,000
Test Statistic	94,000
Standard Error	34,491
Standardized Test Statistic	,319
Asymptotic Sig.(2-sided test)	,750
Exact Sig.(2-sided test)	,767

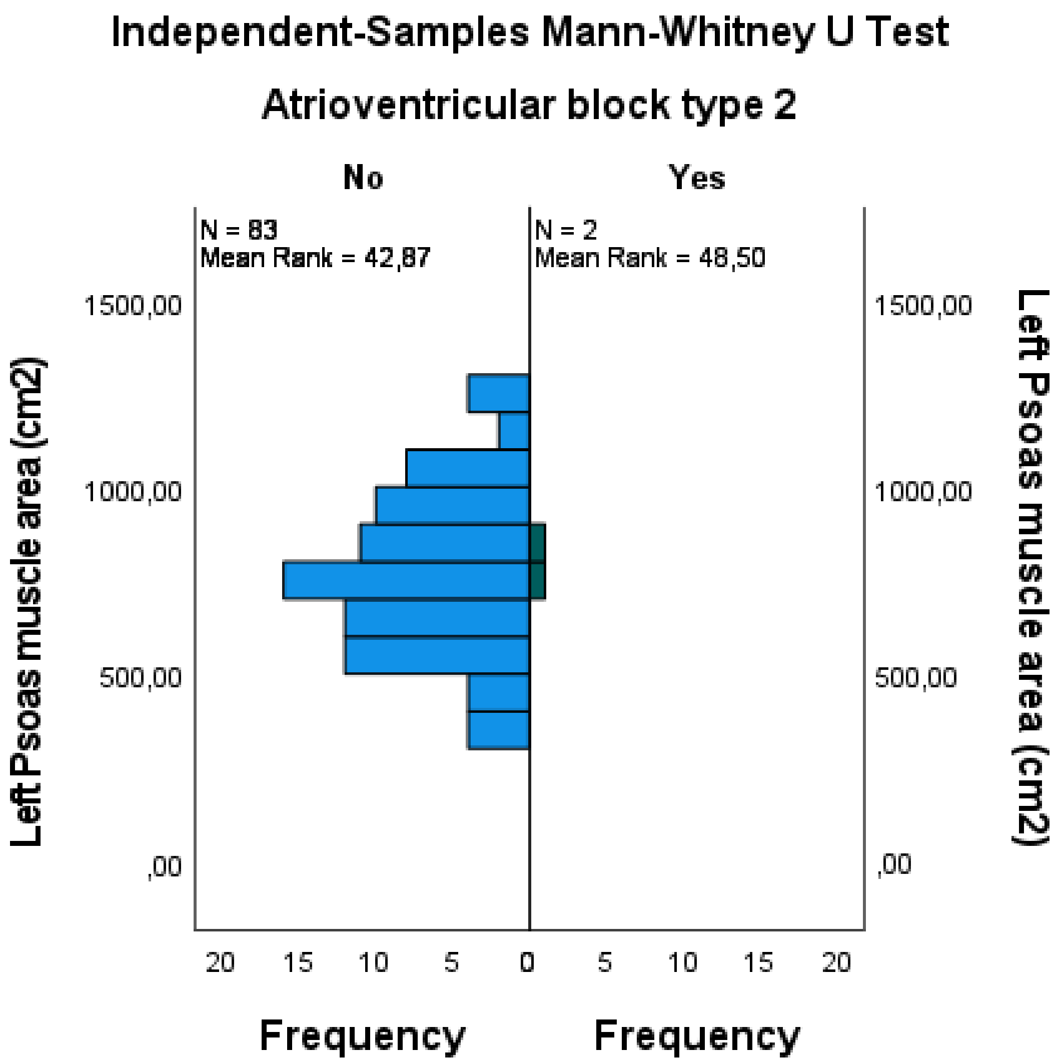

FAT mean density (HU) across Atrioventricular block type 2

Independent-Samples Mann-Whitney U Test Summary

Total N	85
Mann-Whitney U	52,000
Wilcoxon W	55,000
Test Statistic	52,000
Standard Error	34,486
Standardized Test Statistic	-,899
Asymptotic Sig.(2-sided test)	,369
Exact Sig.(2-sided test)	,408

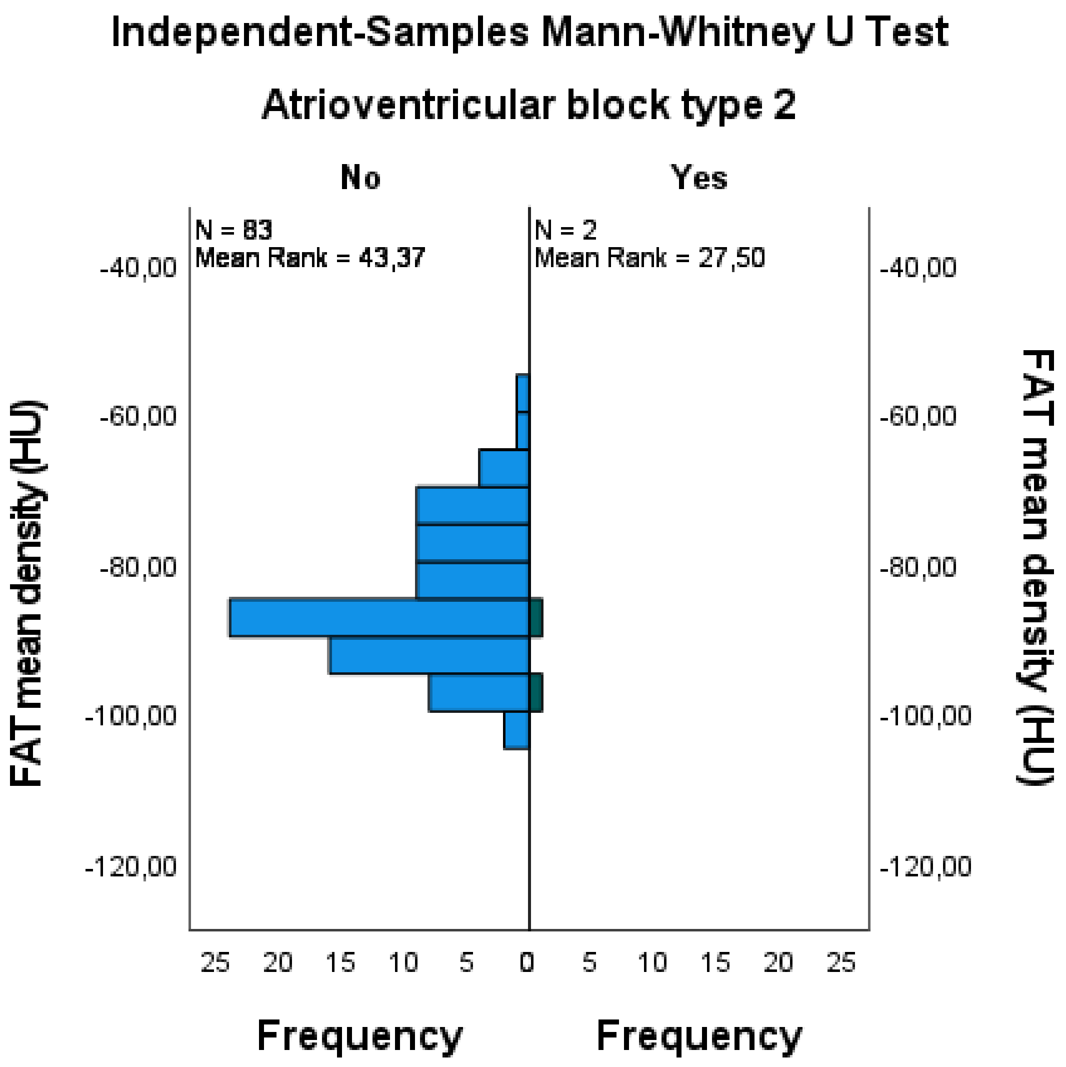

FAT median density (HU) across Atrioventricular block type 2

Independent-Samples Mann-Whitney U Test Summary

Total N	85
Mann-Whitney U	48,500
Wilcoxon W	51,500
Test Statistic	48,500
Standard Error	34,450
Standardized Test Statistic	-1,001
Asymptotic Sig.(2-sided test)	,317
Exact Sig.(2-sided test)	,350

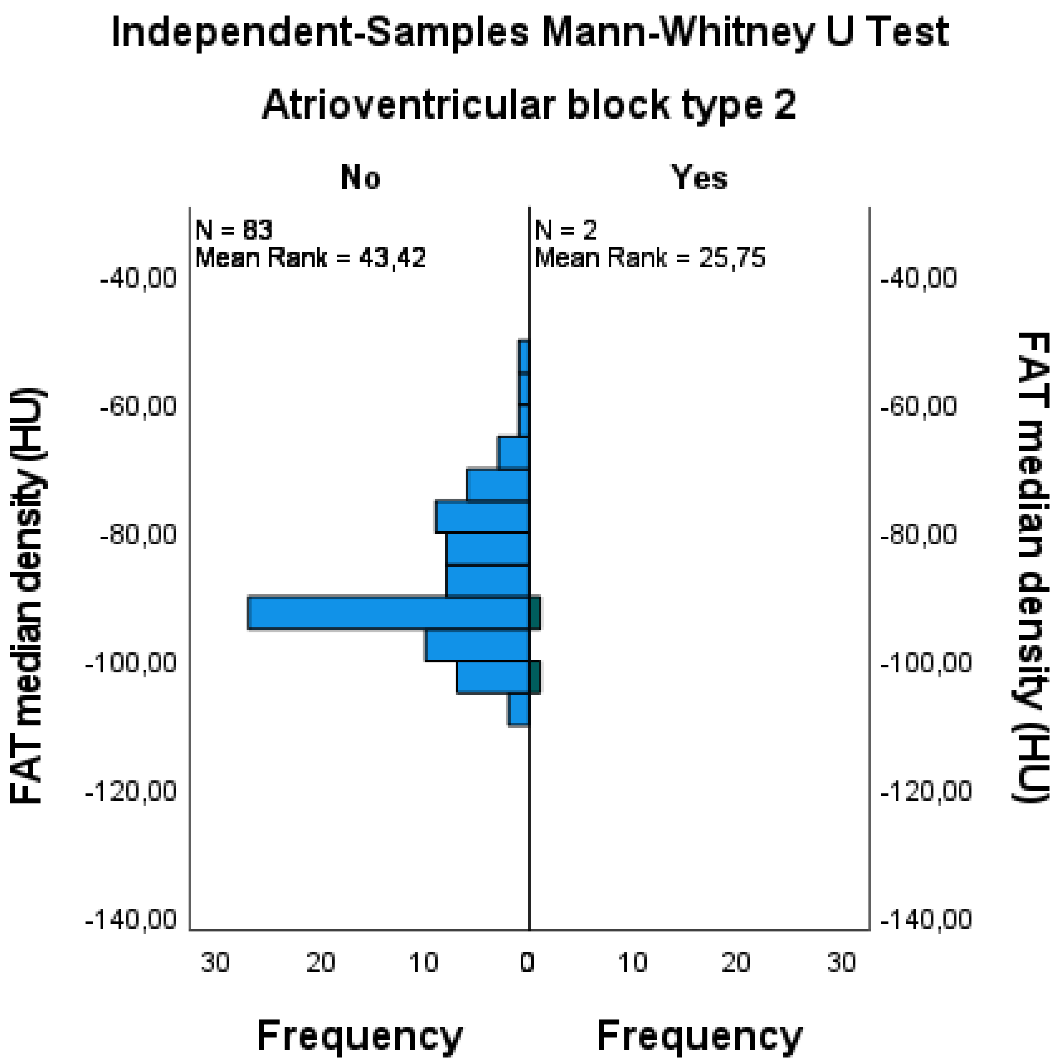

FAT density standard deviation across Atrioventricular block type 2

Independent-Samples Mann-Whitney U Test Summary

Total N	85
Mann-Whitney U	126,000
Wilcoxon W	129,000
Test Statistic	126,000
Standard Error	34,491
Standardized Test Statistic	1,247
Asymptotic Sig.(2-sided test)	,213
Exact Sig.(2-sided test)	,247

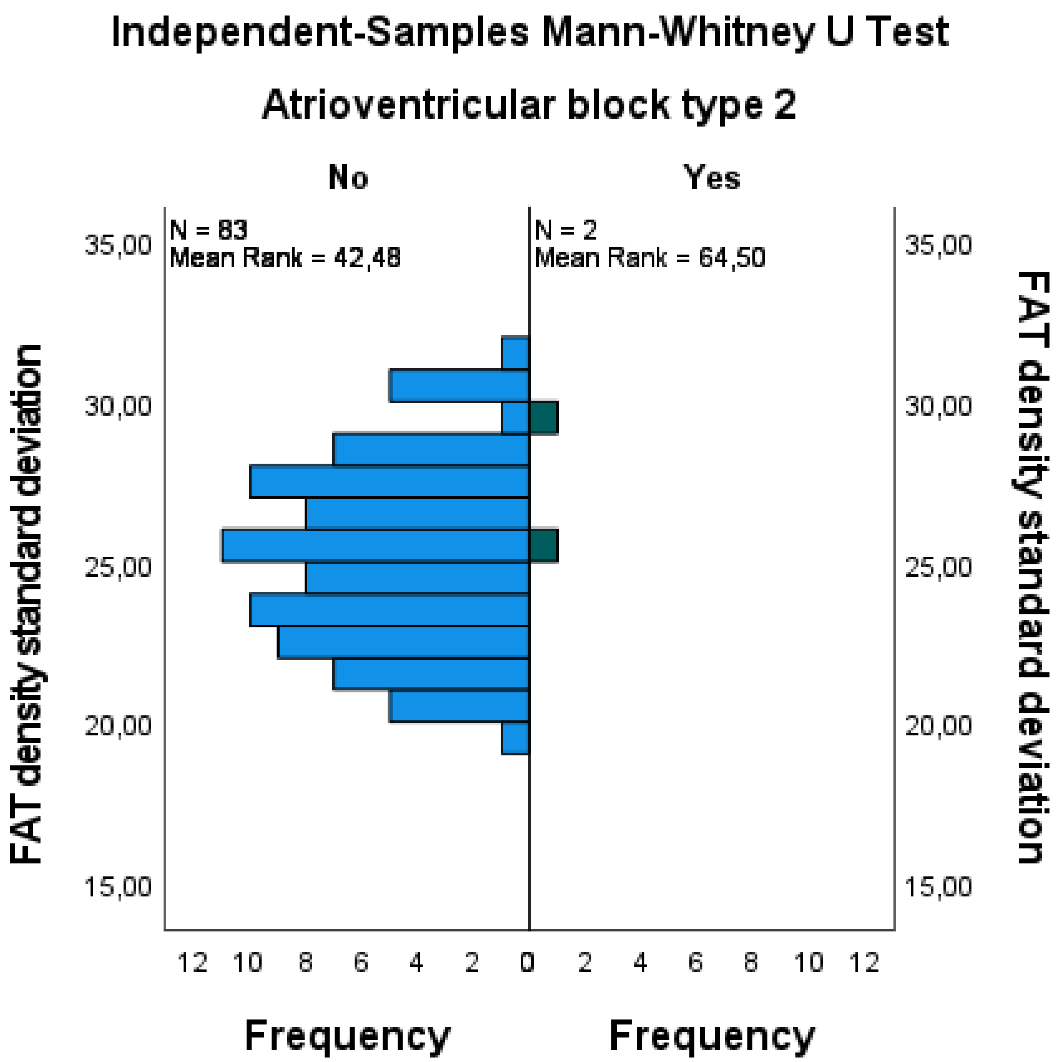

SAT mean density (HU) across Atrioventricular block type 2

Independent-Samples Mann-Whitney U Test Summary

Total N	85
Mann-Whitney U	47,000
Wilcoxon W	50,000
Test Statistic	47,000
Standard Error	34,488
Standardized Test Statistic	-1,044
Asymptotic Sig.(2-sided test)	,297
Exact Sig.(2-sided test)	,336

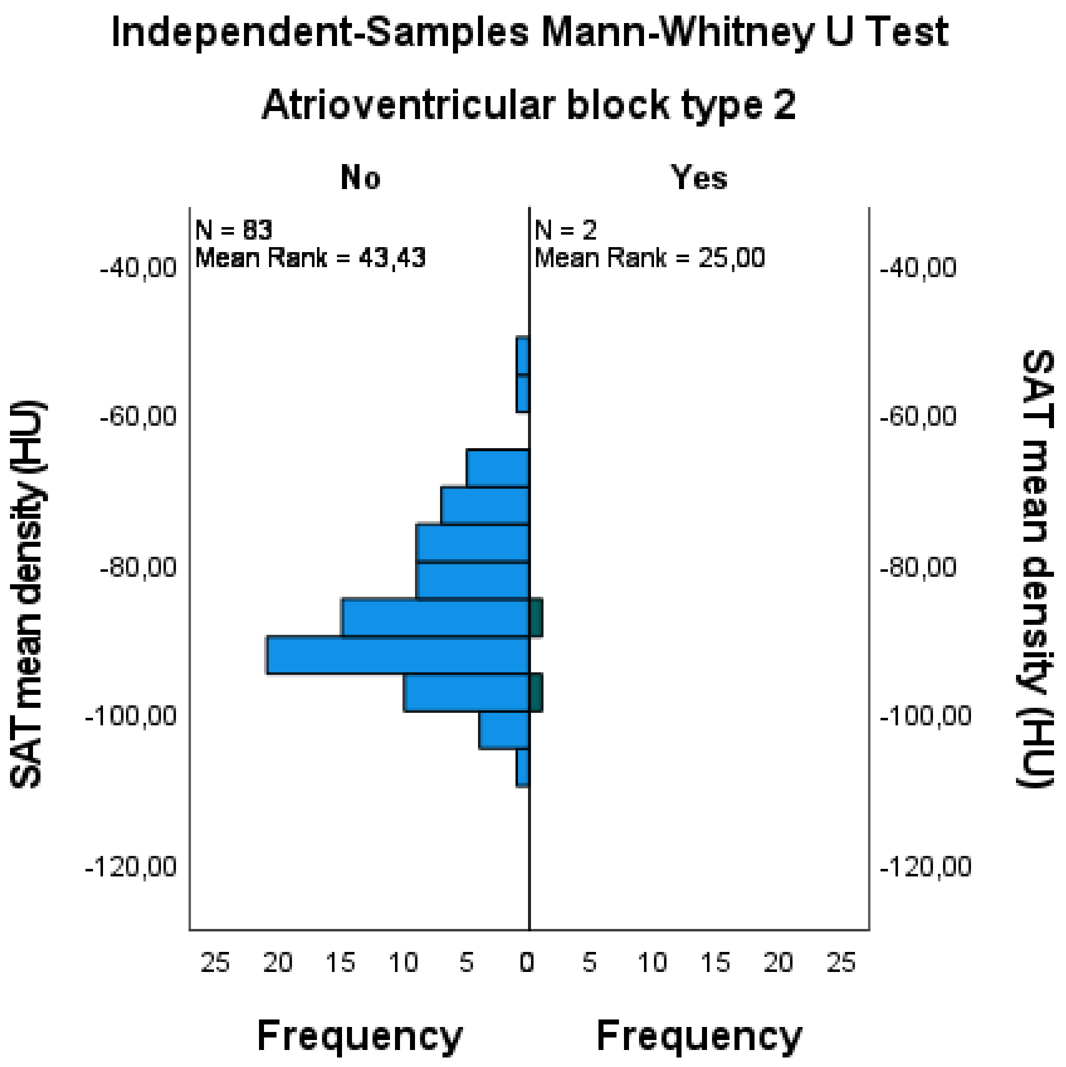

SAT median density (HU) across Atrioventricular block type 2

Independent-Samples Mann-Whitney U Test Summary

Total N	83
Mann-Whitney U	44,500
Wilcoxon W	47,500
Test Statistic	44,500
Standard Error	33,649
Standardized Test Statistic	-1,085
Asymptotic Sig.(2-sided test)	,278
Exact Sig.(2-sided test)	,311

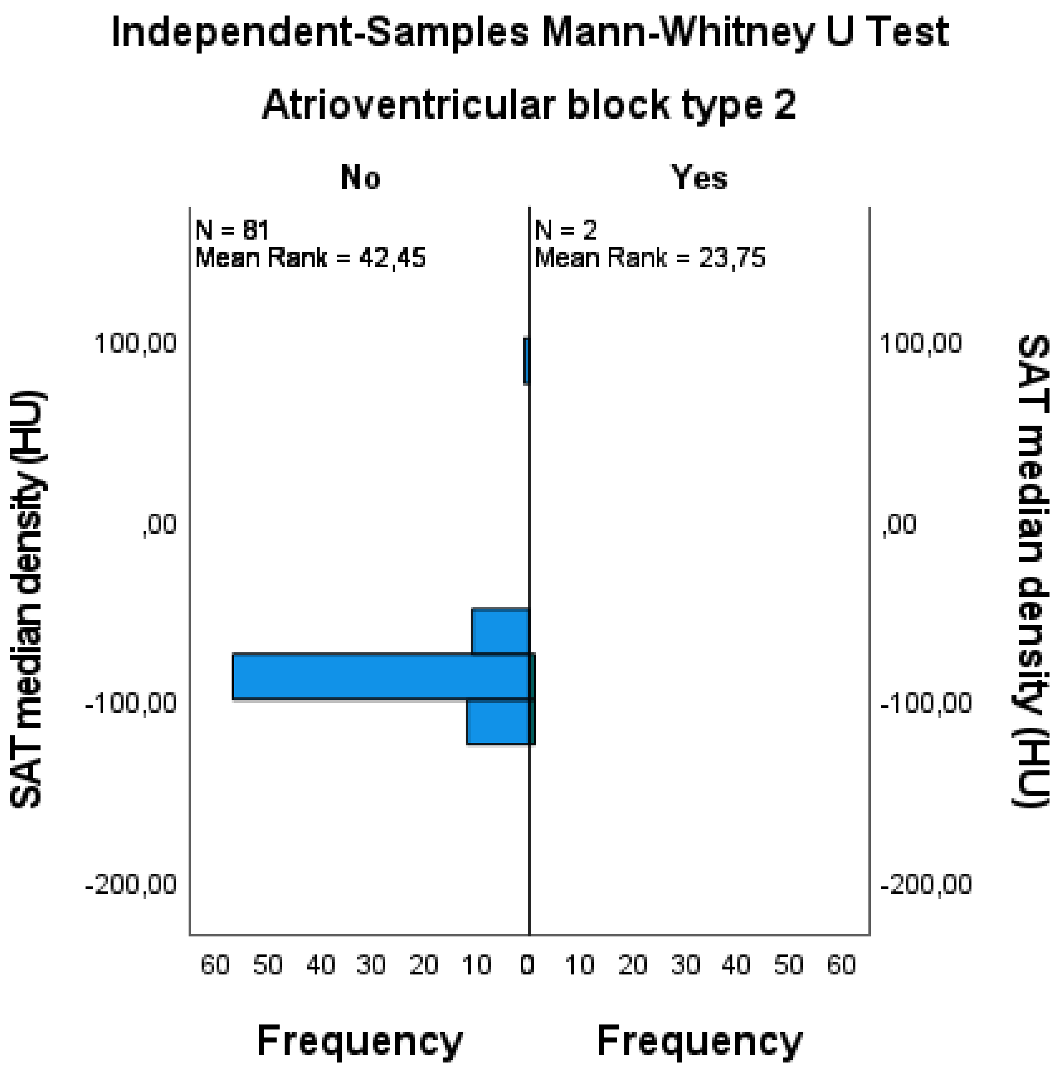

SAT density standard deviation across Atrioventricular block type 2

Independent-Samples Mann-Whitney U Test Summary

Total N	84
Mann-Whitney U	119,000
Wilcoxon W	122,000
Test Statistic	119,000
Standard Error	34,083
Standardized Test Statistic	1,086
Asymptotic Sig.(2-sided test)	,278
Exact Sig.(2-sided test)	,317

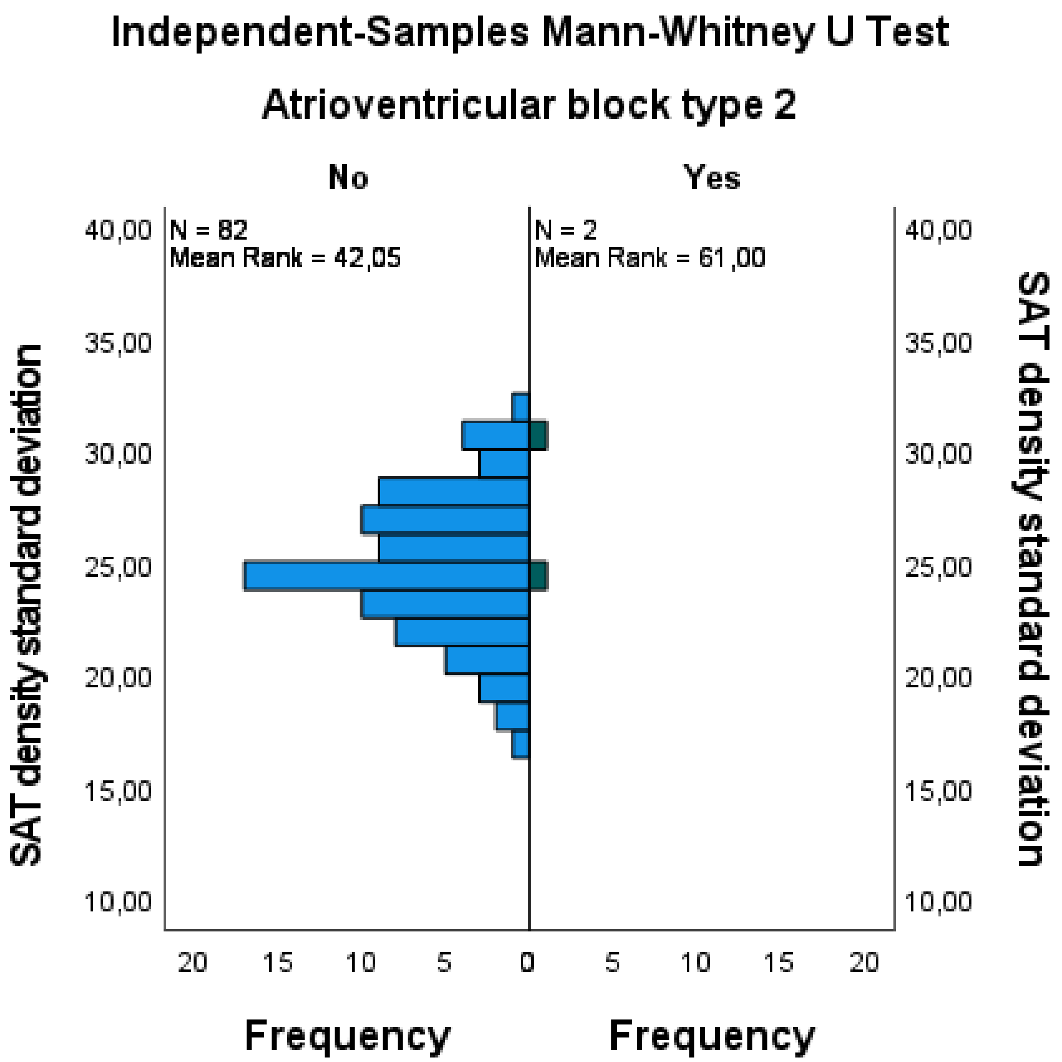

VAT mean density (HU) across Atrioventricular block type 2

Independent-Samples Mann-Whitney U Test Summary

Total N	85
Mann-Whitney U	23,500
Wilcoxon W	26,500
Test Statistic	23,500
Standard Error	34,485
Standardized Test Statistic	-1,725
Asymptotic Sig.(2-sided test)	,084
Exact Sig.(2-sided test)	,087

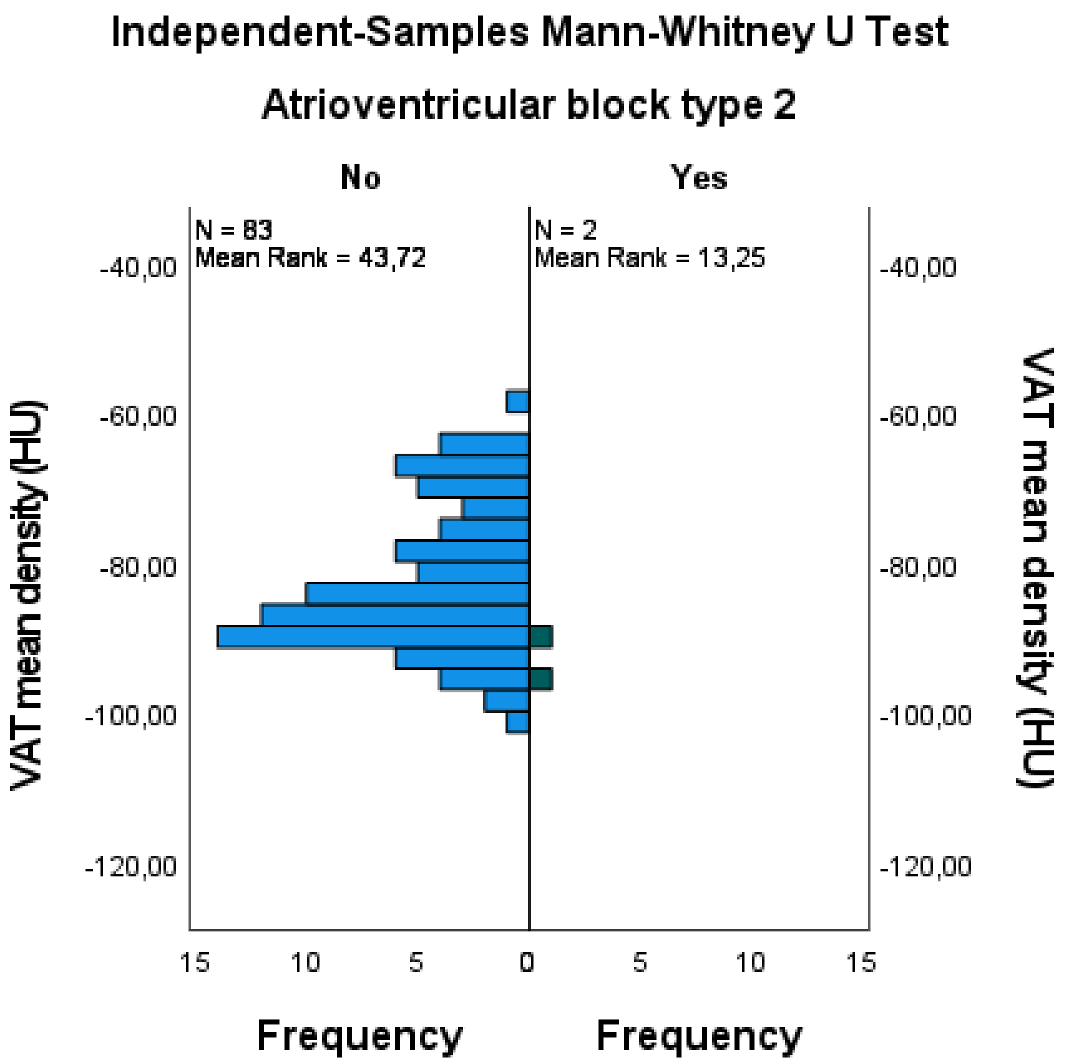

VAT median density (HU) across Atrioventricular block type 2

Independent-Samples Mann-Whitney U Test Summary

Total N	85
Mann-Whitney U	24,000
Wilcoxon W	27,000
Test Statistic	24,000
Standard Error	34,448
Standardized Test Statistic	-1,713
Asymptotic Sig.(2-sided test)	,087
Exact Sig.(2-sided test)	,095

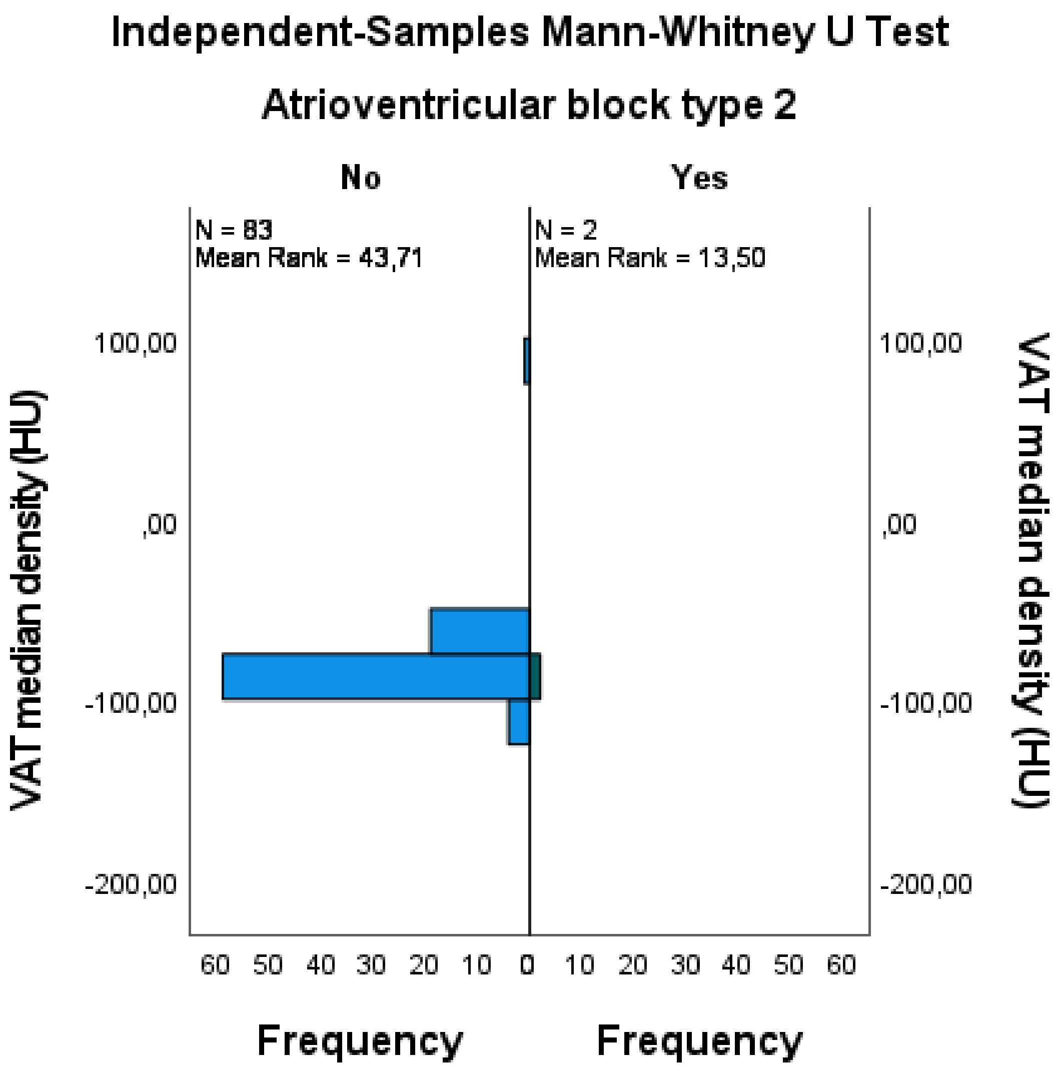

VAT density standard deviation across Atrioventricular block type 2

Independent-Samples Mann-Whitney U Test Summary

Total N	84
Mann-Whitney U	123,000
Wilcoxon W	126,000
Test Statistic	123,000
Standard Error	34,083
Standardized Test Statistic	1,203
Asymptotic Sig.(2-sided test)	,229
Exact Sig.(2-sided test)	,265

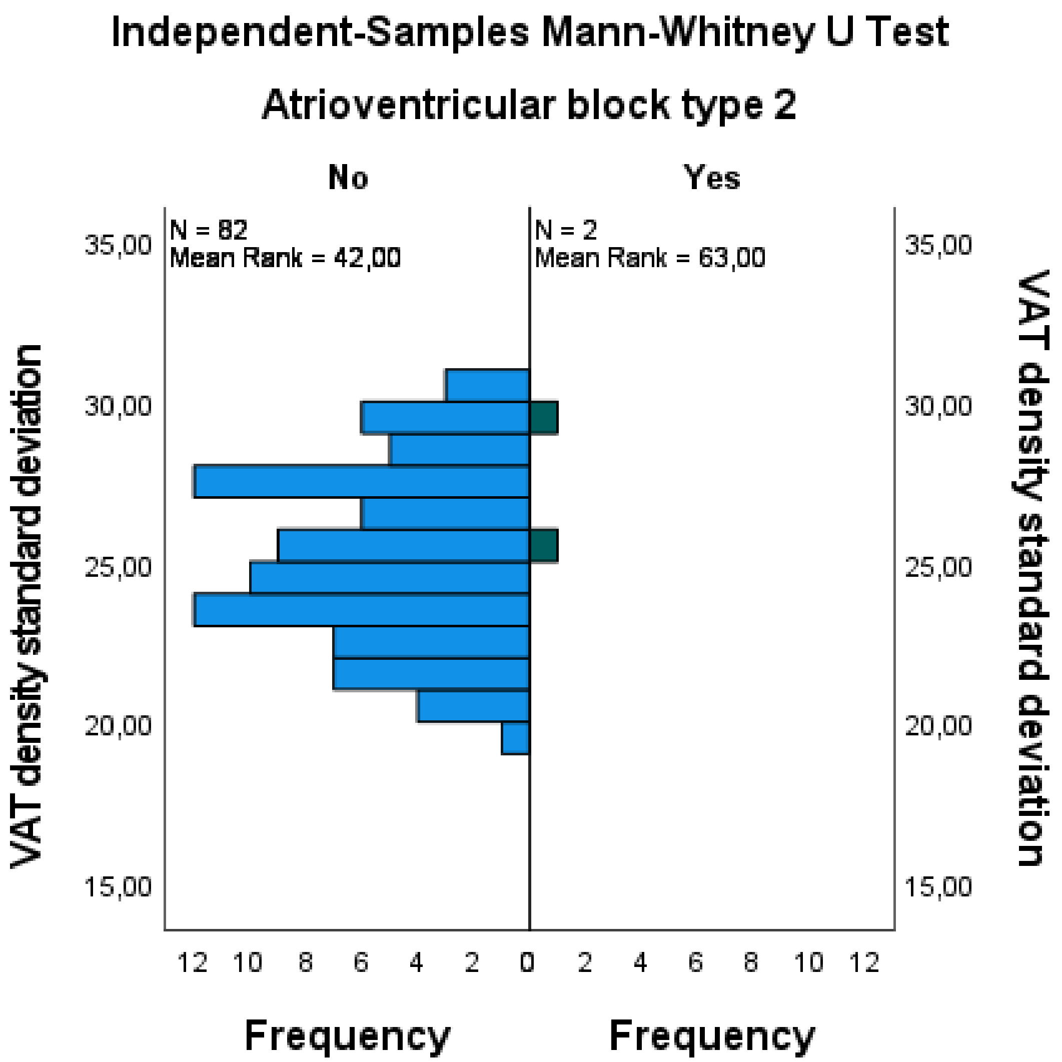

Right Psoas muscle mean density (HU) across Atrioventricular block type 2

Independent-Samples Mann-Whitney U Test Summary

Total N	85
Mann-Whitney U	16,000
Wilcoxon W	19,000
Test Statistic	16,000
Standard Error	34,484
Standardized Test Statistic	-1,943
Asymptotic Sig.(2-sided test)	,052
Exact Sig.(2-sided test)	,045

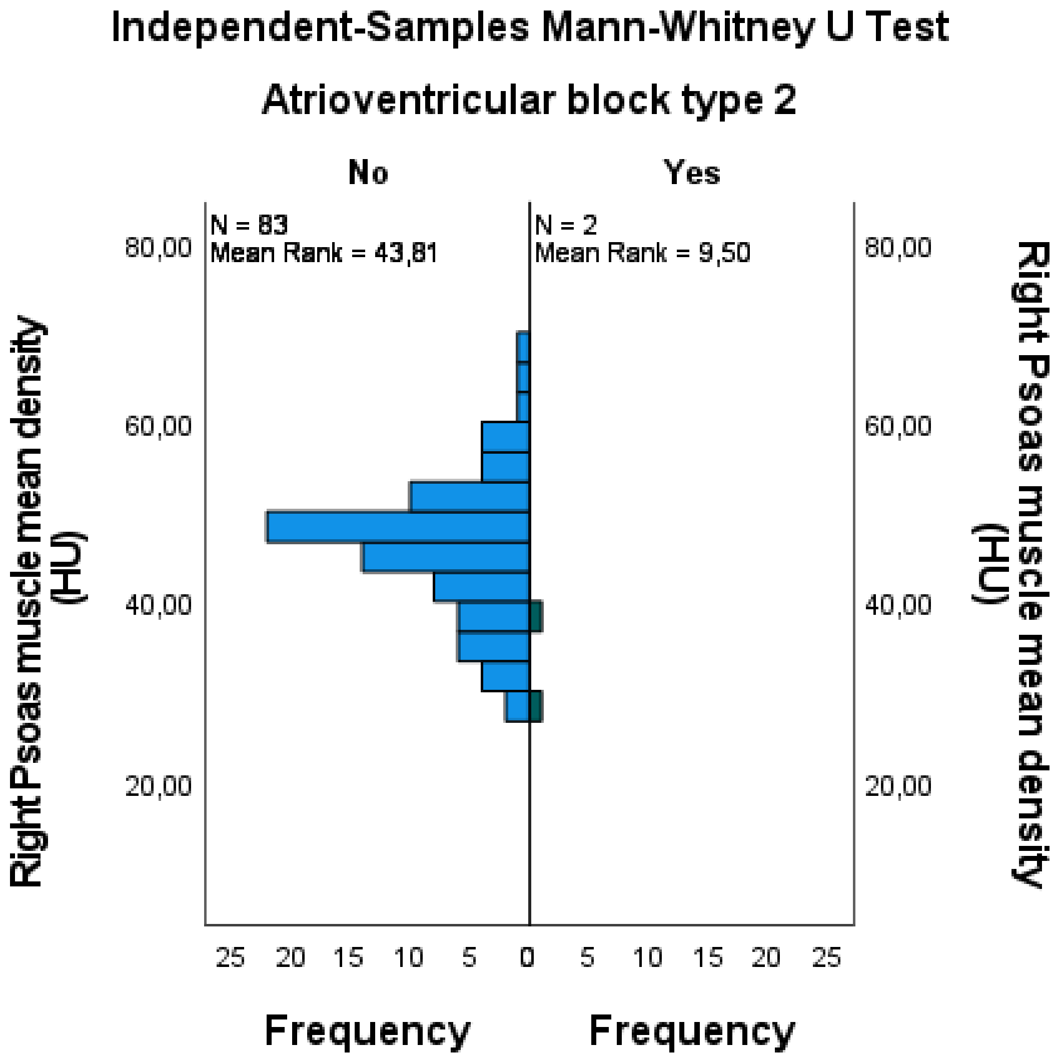

Right Psoas muscle median density (HU) across Atrioventricular block type 2

Independent-Samples Mann-Whitney U Test Summary

Total N	85
Mann-Whitney U	18,500
Wilcoxon W	21,500
Test Statistic	18,500
Standard Error	34,459
Standardized Test Statistic	-1,872
Asymptotic Sig.(2-sided test)	,061
Exact Sig.(2-sided test)	,056

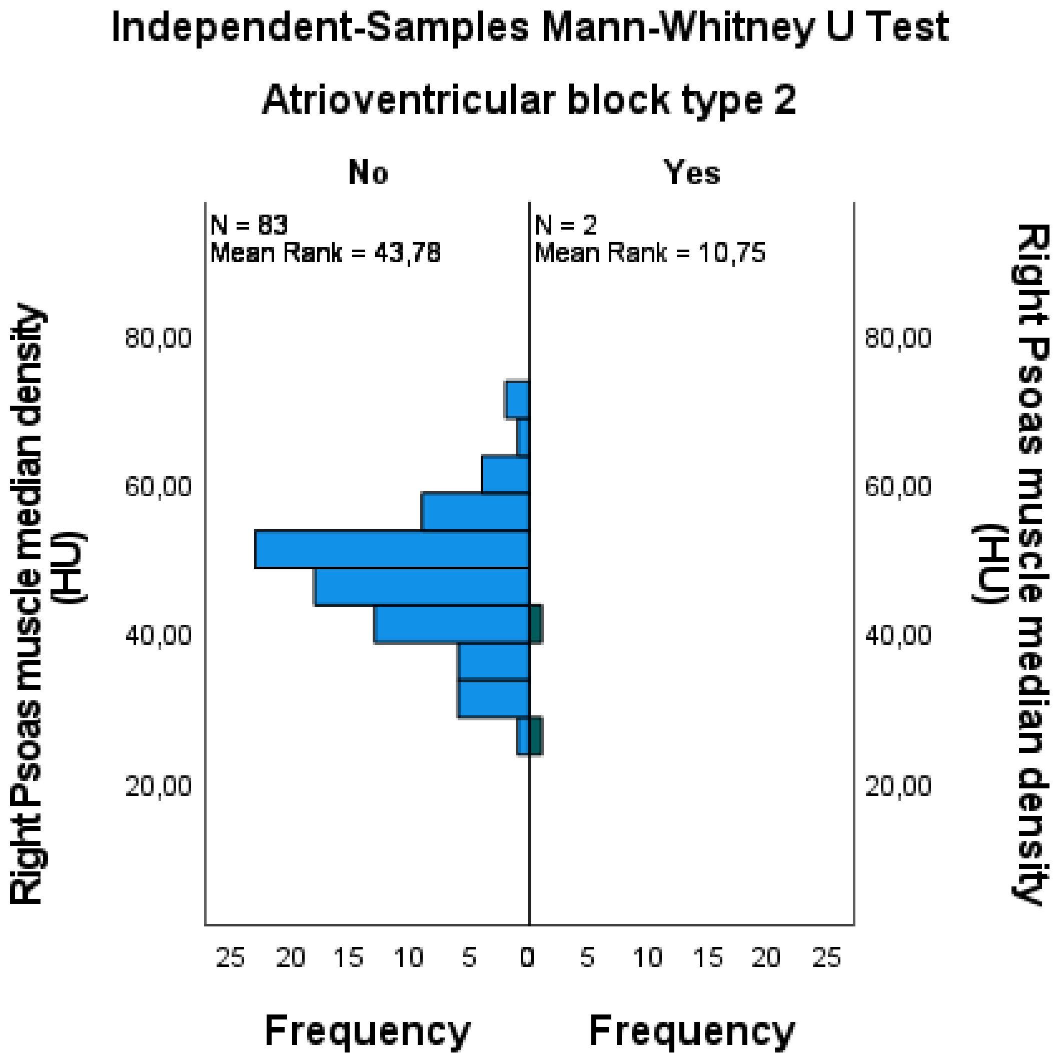

Right Psoas muscle density standard deviation across Atrioventricular block type 2

Independent-Samples Mann-Whitney U Test Summary

Total N	85
Mann-Whitney U	98,000
Wilcoxon W	101,000
Test Statistic	98,000
Standard Error	34,491
Standardized Test Statistic	,435
Asymptotic Sig.(2-sided test)	,664
Exact Sig.(2-sided test)	,686

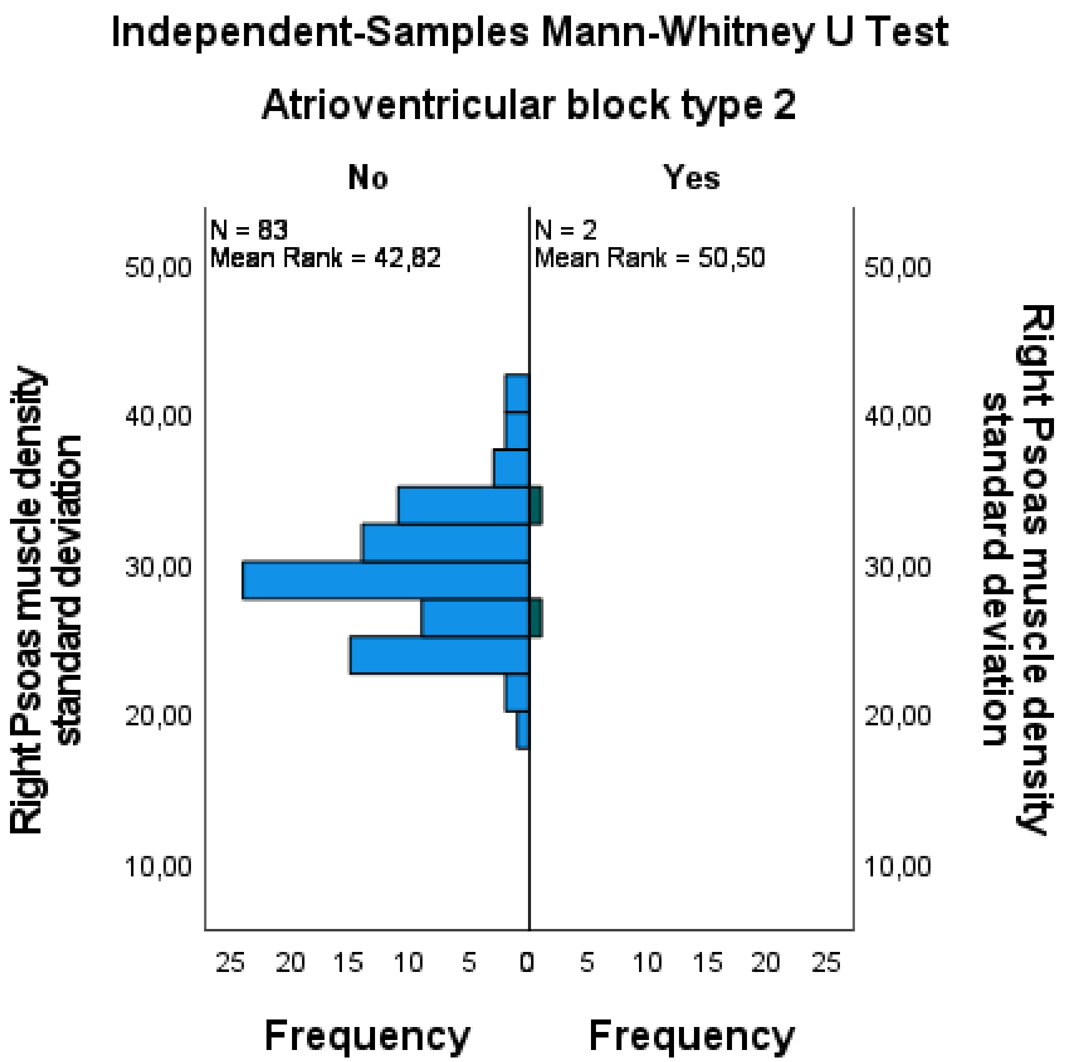

Left Psoas muscle mean density (HU) across Atrioventricular block type 2

Independent-Samples Mann-Whitney U Test Summary

Total N	85
Mann-Whitney U	25,500
Wilcoxon W	28,500
Test Statistic	25,500
Standard Error	34,484
Standardized Test Statistic	-1,667
Asymptotic Sig.(2-sided test)	,095
Exact Sig.(2-sided test)	,102

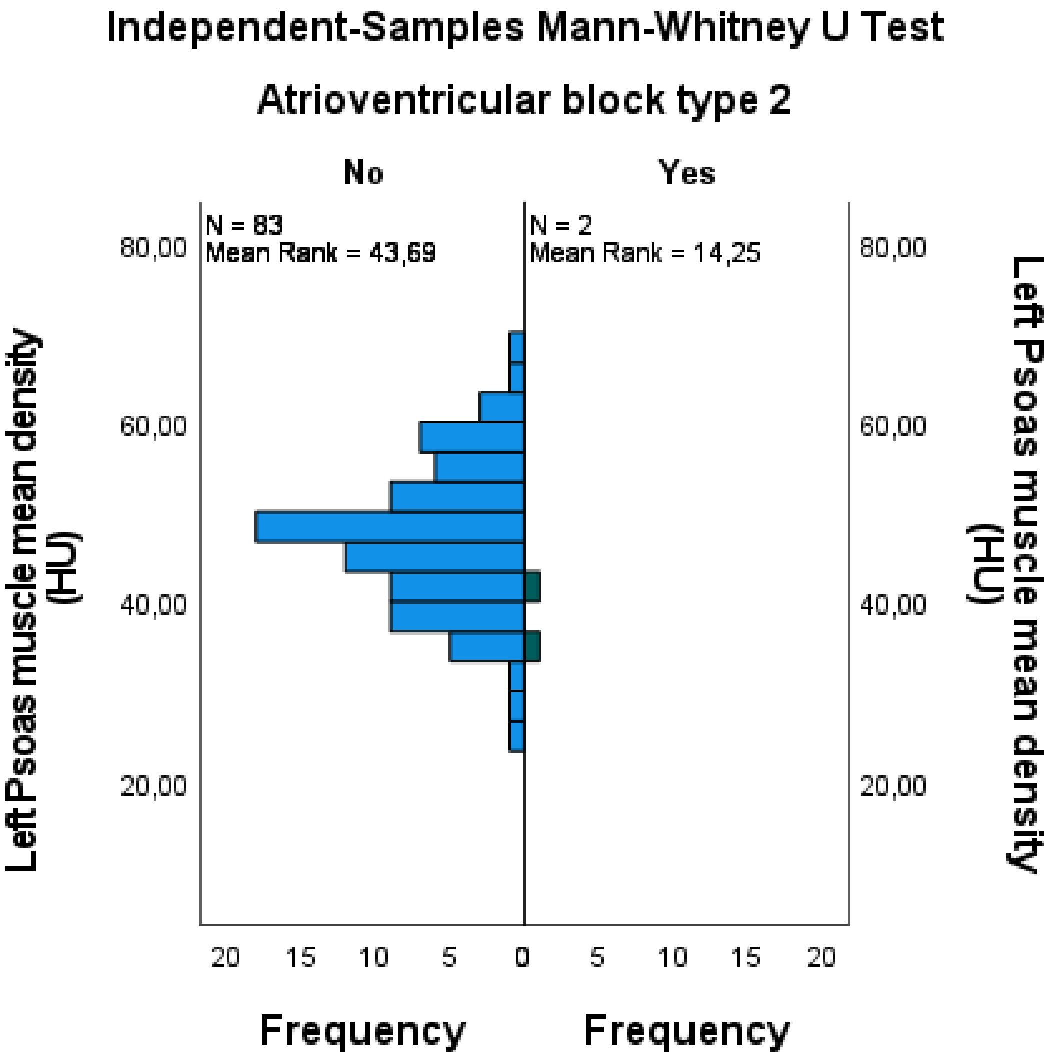

Left Psoas muscle median density (HU) across Atrioventricular block type 2

Independent-Samples Mann-Whitney U Test Summary

Total N	85
Mann-Whitney U	18,000
Wilcoxon W	21,000
Test Statistic	18,000
Standard Error	34,448
Standardized Test Statistic	-1,887
Asymptotic Sig.(2-sided test)	,059
Exact Sig.(2-sided test)	,056

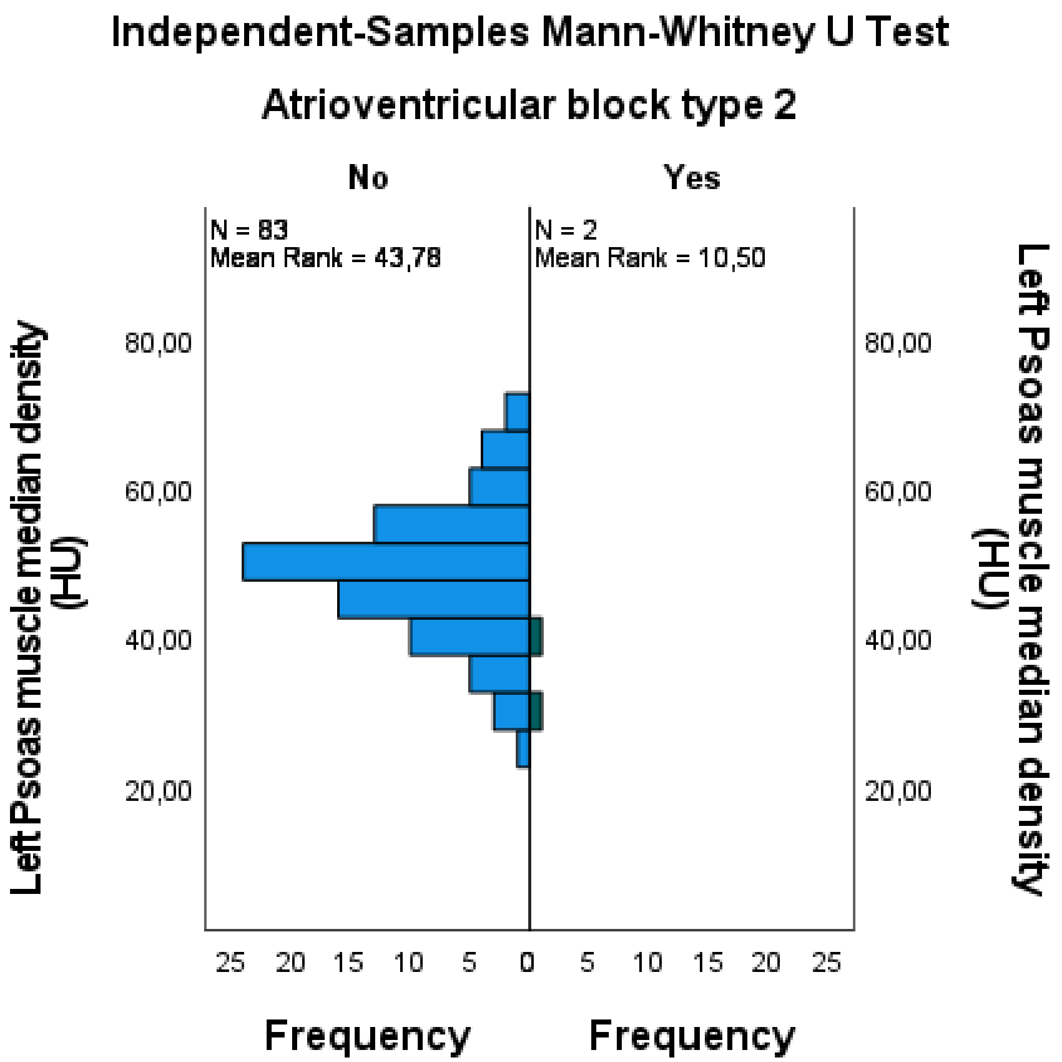

Left Psoas muscle density standard deviation across Atrioventricular block type 2

Independent-Samples Mann-Whitney U Test Summary

Total N	85
Mann-Whitney U	101,000
Wilcoxon W	104,000
Test Statistic	101,000
Standard Error	34,492
Standardized Test Statistic	,522
Asymptotic Sig.(2-sided test)	,602
Exact Sig.(2-sided test)	,629

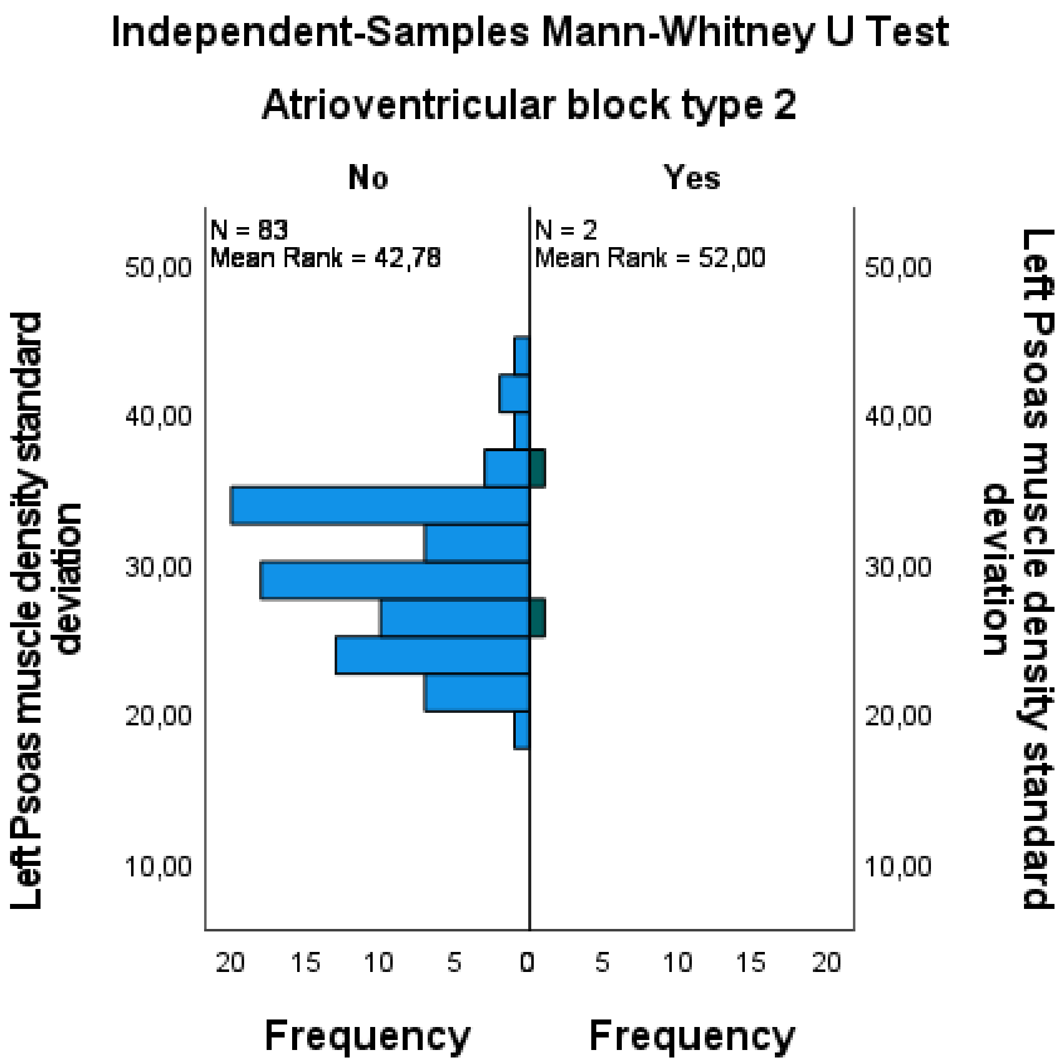

Independent-Samples Mann-Whitney U Test for atrioventricular block type 3 occurrence

Hypothesis Test Summary

	Null Hypothesis	Test	Sig.[a,b]	Decision
1	The distribution of Psoas/height is the same across categories of Atrioventricular block type 3.	Independent-Samples Mann-Whitney U Test	,903	Retain the null hypothesis.

2	The distribution of Anterior SAT distance is the same across categories of Atrioventricular block type 3.	Independent-Samples Mann-Whitney U Test	,507	Retain the null hypothesis.
3	The distribution of Posterior SAT distance is the same across categories of Atrioventricular block type 3.	Independent-Samples Mann-Whitney U Test	,177	Retain the null hypothesis.
4	The distribution of Anterior+Posterior SAT distance is the same across categories of Atrioventricular block type 3.	Independent-Samples Mann-Whitney U Test	,637	Retain the null hypothesis.
5	The distribution of VAT distance is the same across categories of Atrioventricular block type 3.	Independent-Samples Mann-Whitney U Test	,520	Retain the null hypothesis.
6	The distribution of Right common femoral artery area (mm2) is the same across categories of Atrioventricular block type 3.	Independent-Samples Mann-Whitney U Test	,357	Retain the null hypothesis.
7	The distribution of Left common femoral artery area (mm2) is the same across categories of Atrioventricular block type 3.	Independent-Samples Mann-Whitney U Test	,134	Retain the null hypothesis.
8	The distribution of FAT area (cm2) is the same across categories of Atrioventricular block type 3.	Independent-Samples Mann-Whitney U Test	,539	Retain the null hypothesis.
9	The distribution of SAT area (cm2) is the same across categories of Atrioventricular block type 3.	Independent-Samples Mann-Whitney U Test	,578	Retain the null hypothesis.
10	The distribution of VAT area (cm2) is the same across categories of Atrioventricular block type 3.	Independent-Samples Mann-Whitney U Test	,767	Retain the null hypothesis.
11	The distribution of Right Psoas muscle area (cm2) is the same across categories of Atrioventricular block type 3.	Independent-Samples Mann-Whitney U Test	,184	Retain the null hypothesis.

12	The distribution of Left Psoas muscle area (cm2) is the same across categories of Atrioventricular block type 3.	Independent-Samples Mann-Whitney U Test	,130	Retain the null hypothesis.
13	The distribution of FAT mean density (HU) is the same across categories of Atrioventricular block type 3.	Independent-Samples Mann-Whitney U Test	,411	Retain the null hypothesis.
14	The distribution of FAT median density (HU) is the same across categories of Atrioventricular block type 3.	Independent-Samples Mann-Whitney U Test	,436	Retain the null hypothesis.
15	The distribution of FAT density standard deviation is the same across categories of Atrioventricular block type 3.	Independent-Samples Mann-Whitney U Test	,307	Retain the null hypothesis.
16	The distribution of SAT mean density (HU) is the same across categories of Atrioventricular block type 3.	Independent-Samples Mann-Whitney U Test	,573	Retain the null hypothesis.
17	The distribution of SAT median density (HU) is the same across categories of Atrioventricular block type 3.	Independent-Samples Mann-Whitney U Test	,655	Retain the null hypothesis.
18	The distribution of SAT density standard deviation is the same across categories of Atrioventricular block type 3.	Independent-Samples Mann-Whitney U Test	,659	Retain the null hypothesis.
19	The distribution of VAT mean density (HU) is the same across categories of Atrioventricular block type 3.	Independent-Samples Mann-Whitney U Test	,684	Retain the null hypothesis.
20	The distribution of VAT median density (HU) is the same across categories of Atrioventricular block type 3.	Independent-Samples Mann-Whitney U Test	,841	Retain the null hypothesis.
21	The distribution of VAT density standard deviation is the same across categories of Atrioventricular block type 3.	Independent-Samples Mann-Whitney U Test	,329	Retain the null hypothesis.

22	The distribution of Right Psoas muscle mean density (HU) is the same across categories of Atrioventricular block type 3.	Independent-Samples Mann-Whitney U Test	,012	Reject the null hypothesis.
23	The distribution of Right Psoas muscle median density (HU) is the same across categories of Atrioventricular block type 3.	Independent-Samples Mann-Whitney U Test	,014	Reject the null hypothesis.
24	The distribution of Right Psoas muscle density standard deviation is the same across categories of Atrioventricular block type 3.	Independent-Samples Mann-Whitney U Test	,026	Reject the null hypothesis.
25	The distribution of Left Psoas muscle mean density (HU) is the same across categories of Atrioventricular block type 3.	Independent-Samples Mann-Whitney U Test	,300	Retain the null hypothesis.
26	The distribution of Left Psoas muscle median density (HU) is the same across categories of Atrioventricular block type 3.	Independent-Samples Mann-Whitney U Test	,310	Retain the null hypothesis.
27	The distribution of Left Psoas muscle density standard deviation is the same across categories of Atrioventricular block type 3.	Independent-Samples Mann-Whitney U Test	,107	Retain the null hypothesis.

a. The significance level is ,050.

b. Asymptotic significance is displayed.

In this case, the hypothesis of equal medians ($p < 0.05$) is rejected for the Right Psoas muscle mean density (HU), Right Psoas muscle median density (HU) and Right Psoas muscle density standard deviation variables, while for all the others the null hypothesis is accepted ($p > 0.05$).

(The tables and graphs below are the details of the tests in this table: I have highlighted what things you should eventually report, namely test statistic and pvalue).

Psoas/height across Atrioventricular block type 3

Independent-Samples Mann-Whitney U Test Summary

Total N	85
Mann-Whitney U	333,500
Wilcoxon W	378,500

Test Statistic	333,500
Standard Error	70,014
Standardized Test Statistic	-,121
Asymptotic Sig.(2-sided test)	,903

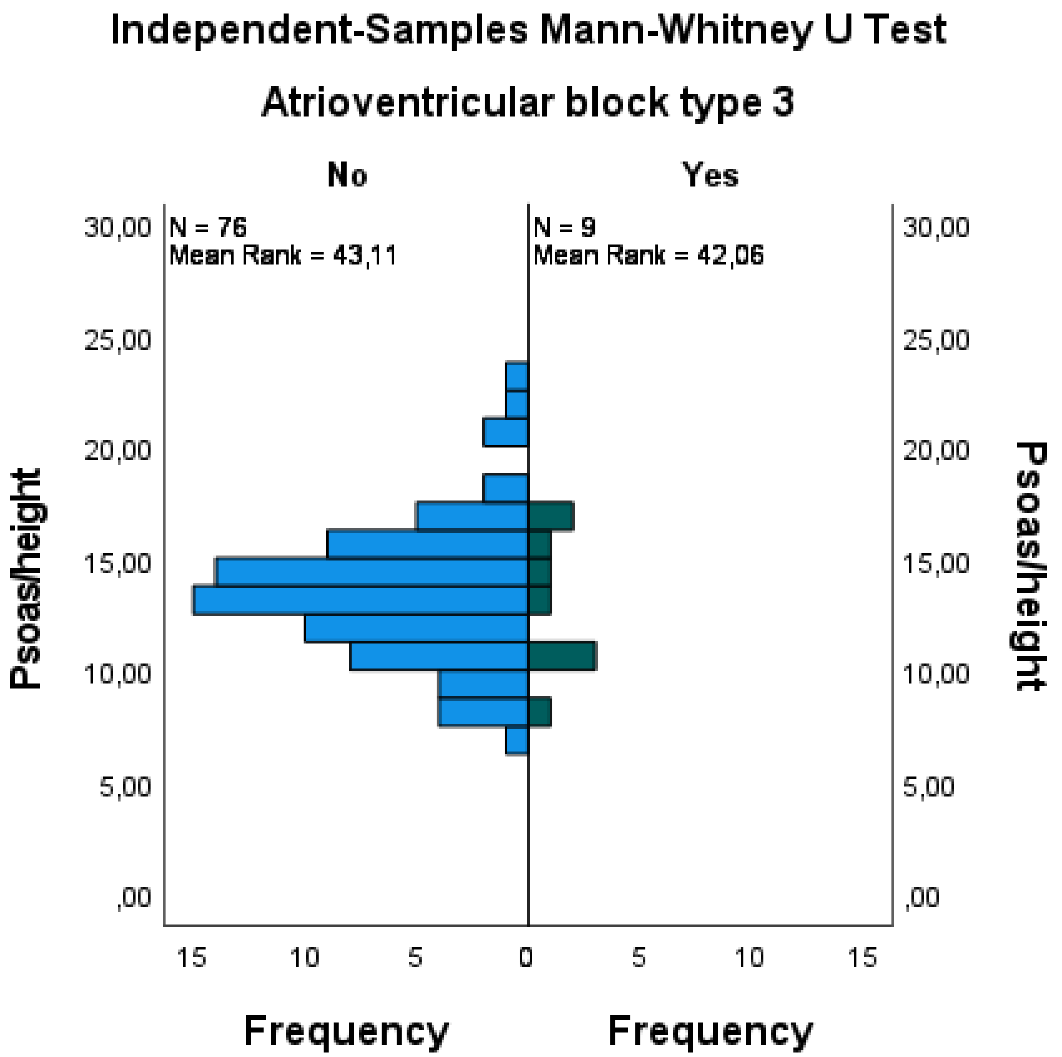

Anterior SAT distance across Atrioventricular block type 3

Independent-Samples Mann-Whitney U Test Summary

Total N	85
Mann-Whitney U	388,500
Wilcoxon W	433,500

Test Statistic	388,500
Standard Error	70,004
Standardized Test Statistic	,664
Asymptotic Sig.(2-sided test)	,507

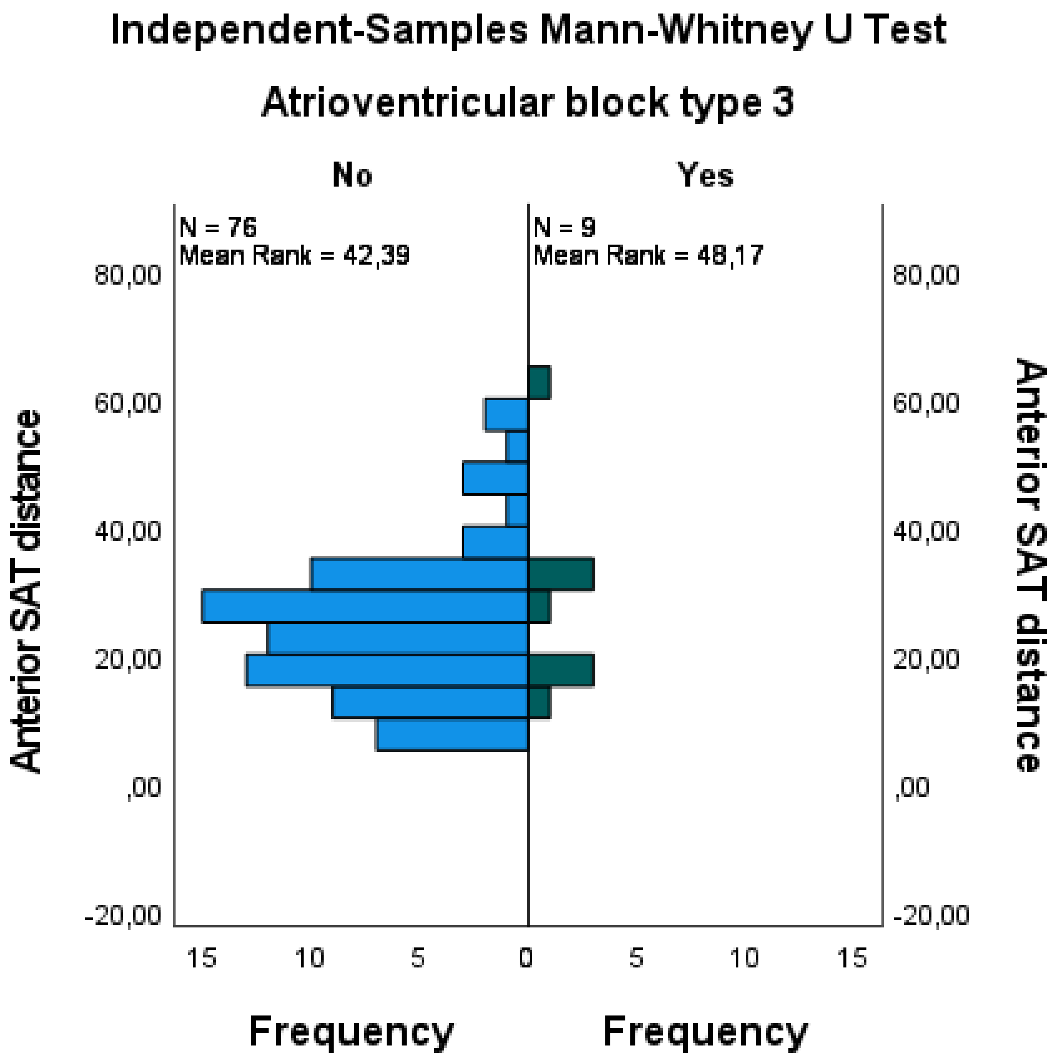

Posterior SAT distance across Atrioventricular block type 3

Independent-Samples Mann-Whitney U Test Summary

Total N	85
Mann-Whitney U	247,500
Wilcoxon W	292,500

Test Statistic	247,500
Standard Error	70,006
Standardized Test Statistic	-1,350
Asymptotic Sig.(2-sided test)	,177

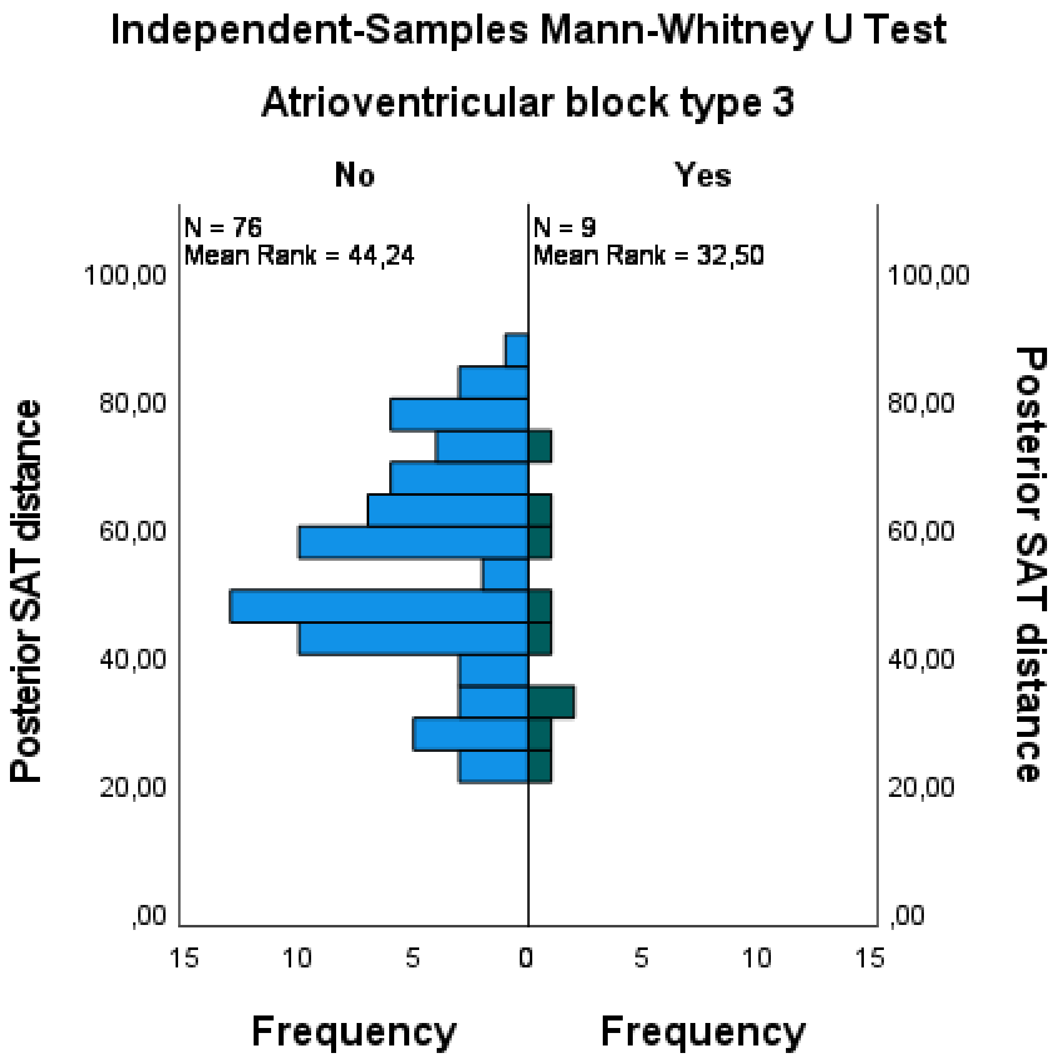

Anterior+Posterior SAT distance across Atrioventricular block type 3

Independent-Samples Mann-Whitney U Test Summary

Total N	85
Mann-Whitney U	309,000
Wilcoxon W	354,000

Test Statistic	309,000
Standard Error	70,011
Standardized Test Statistic	-,471
Asymptotic Sig.(2-sided test)	,637

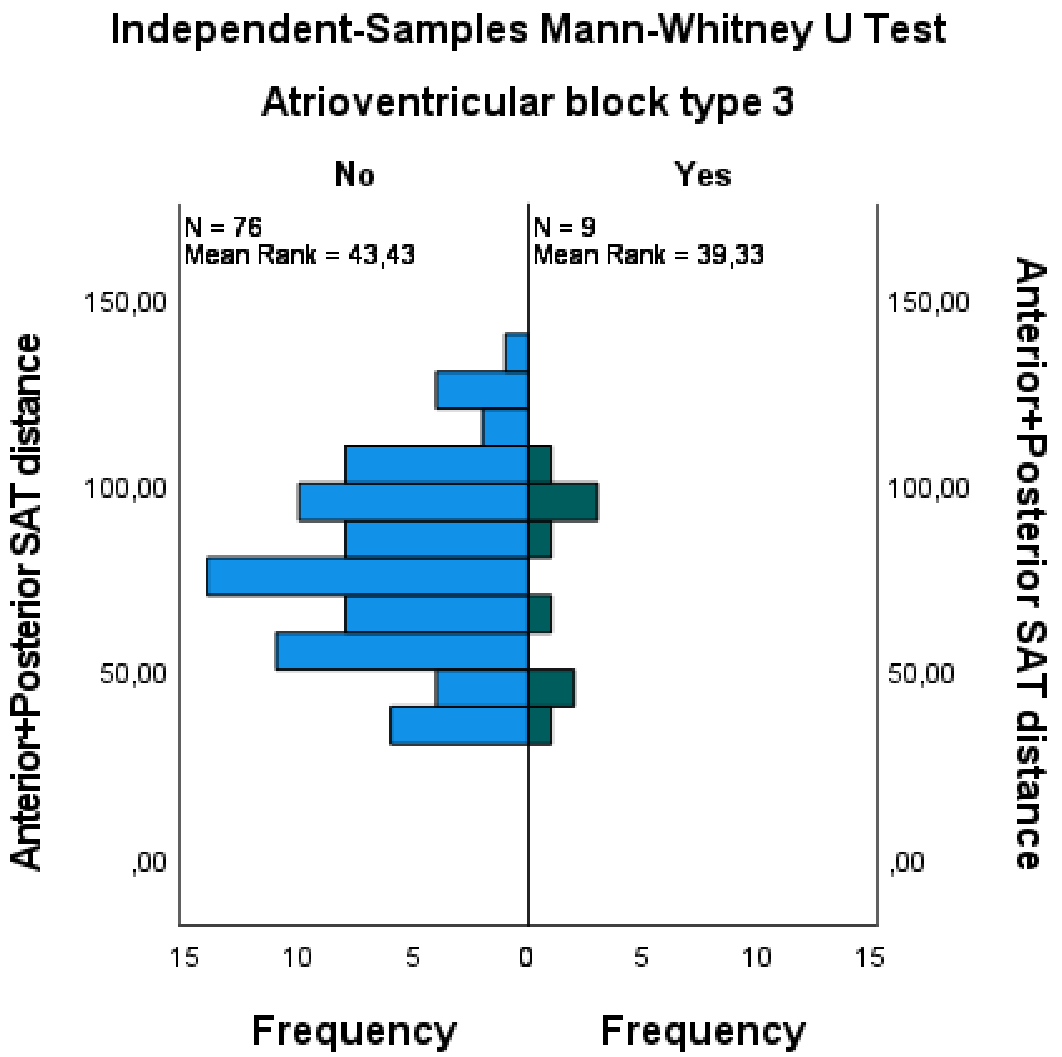

VAT distance across Atrioventricular block type 3

Independent-Samples Mann-Whitney U Test Summary

Total N	84
Mann-Whitney U	293,000
Wilcoxon W	338,000

Test Statistic	293,000
Standard Error	69,133
Standardized Test Statistic	-,644
Asymptotic Sig.(2-sided test)	,520

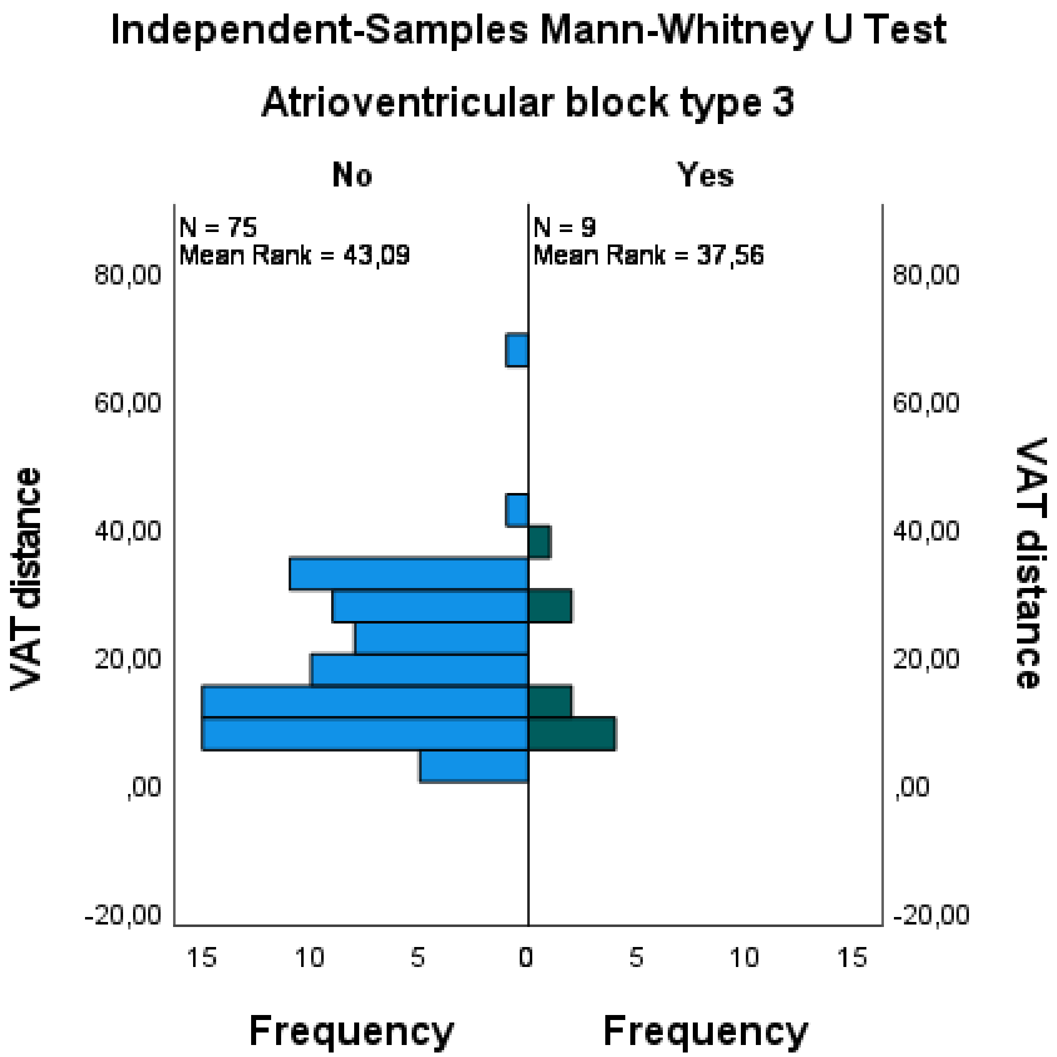

Right common femoral artery area (mm2) across Atrioventricular block type 3

Independent-Samples Mann-Whitney U Test Summary

Total N	85
Mann-Whitney U	277,500
Wilcoxon W	322,500

Test Statistic	277,500
Standard Error	70,003
Standardized Test Statistic	-,921
Asymptotic Sig.(2-sided test)	,357

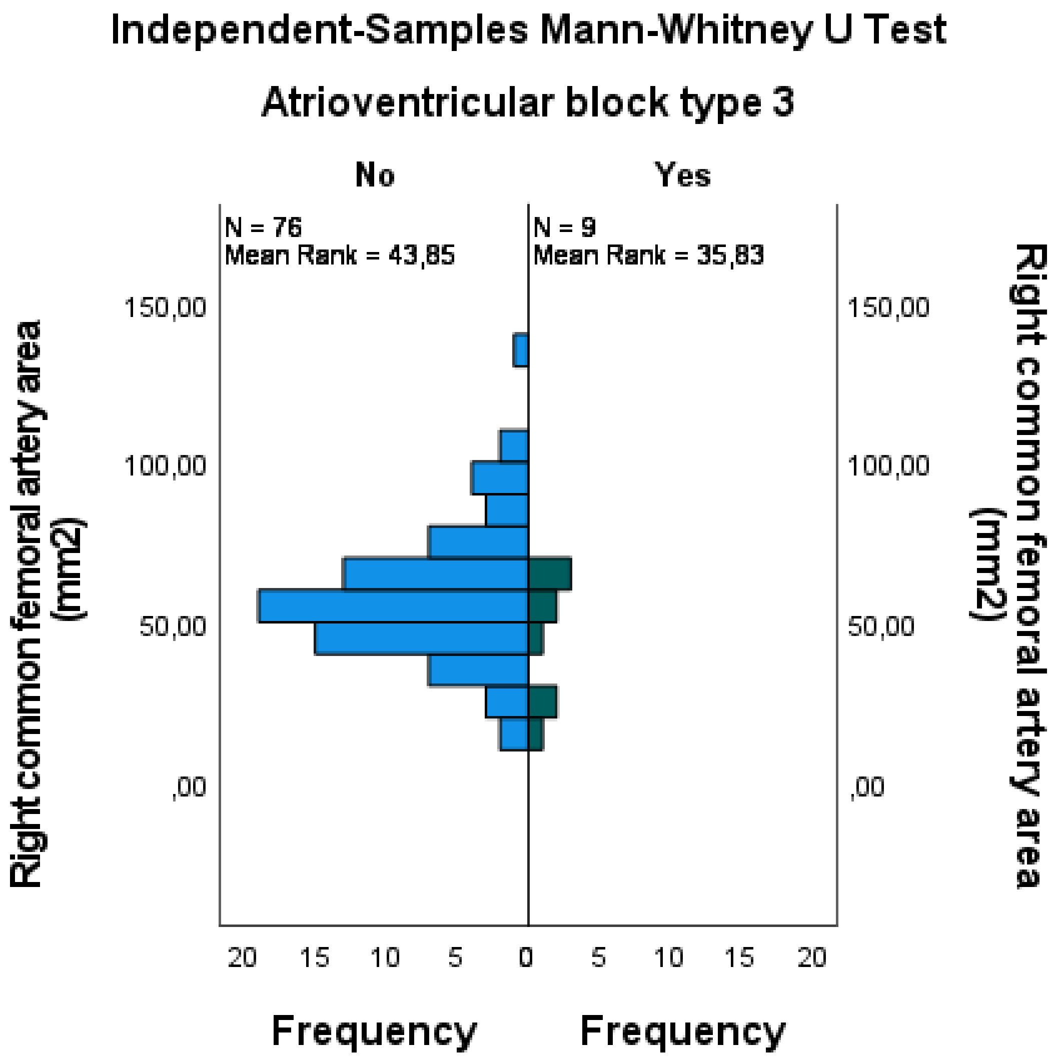

Left common femoral artery area (mm2) across Atrioventricular block type 3

Independent-Samples Mann-Whitney U Test Summary

Total N	85
Mann-Whitney U	237,000
Wilcoxon W	282,000

Test Statistic	237,000
Standard Error	69,997
Standardized Test Statistic	-1,500
Asymptotic Sig.(2-sided test)	,134

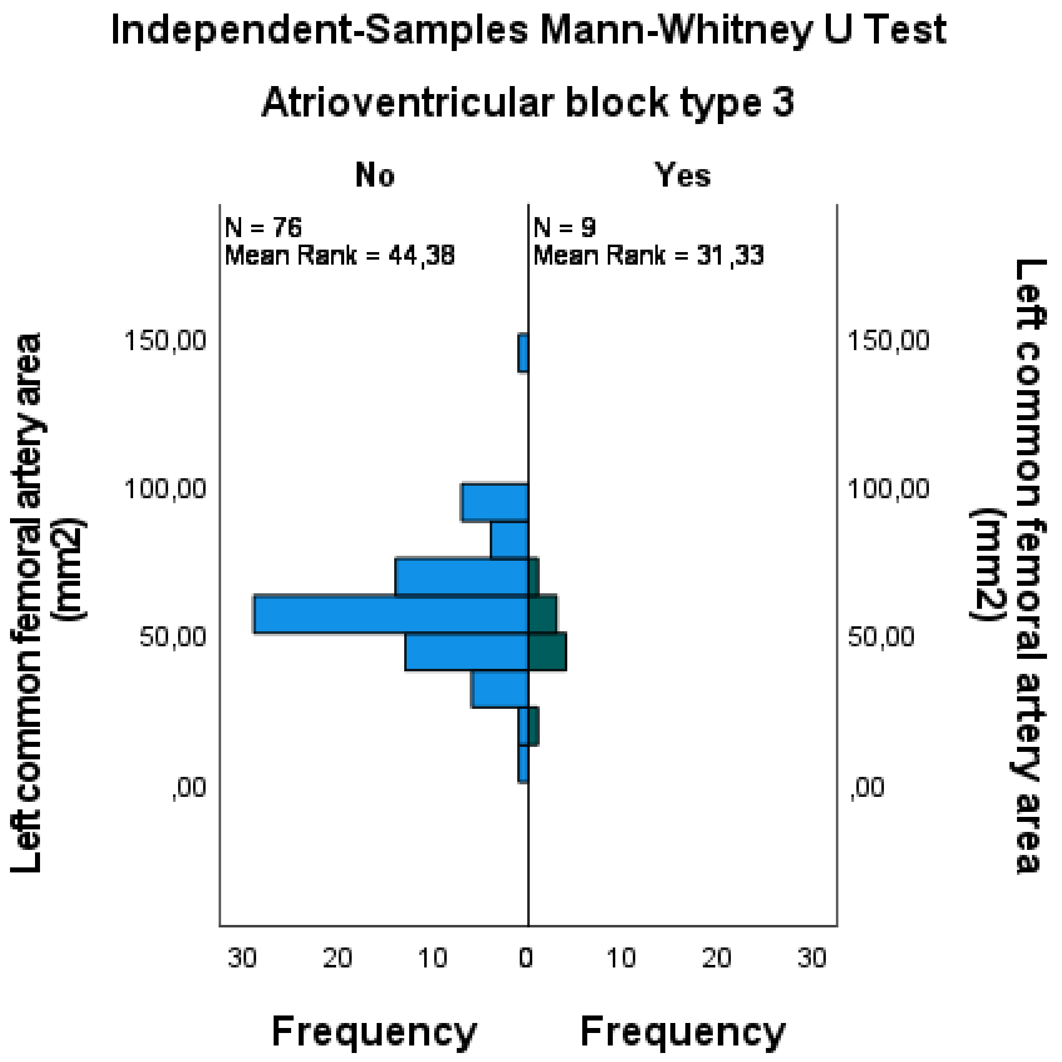

FAT area (cm2) across Atrioventricular block type 3

Independent-Samples Mann-Whitney U Test Summary

Total N	85
Mann-Whitney U	299,000
Wilcoxon W	344,000

Test Statistic	299,000
Standard Error	70,014
Standardized Test Statistic	-,614
Asymptotic Sig.(2-sided test)	,539

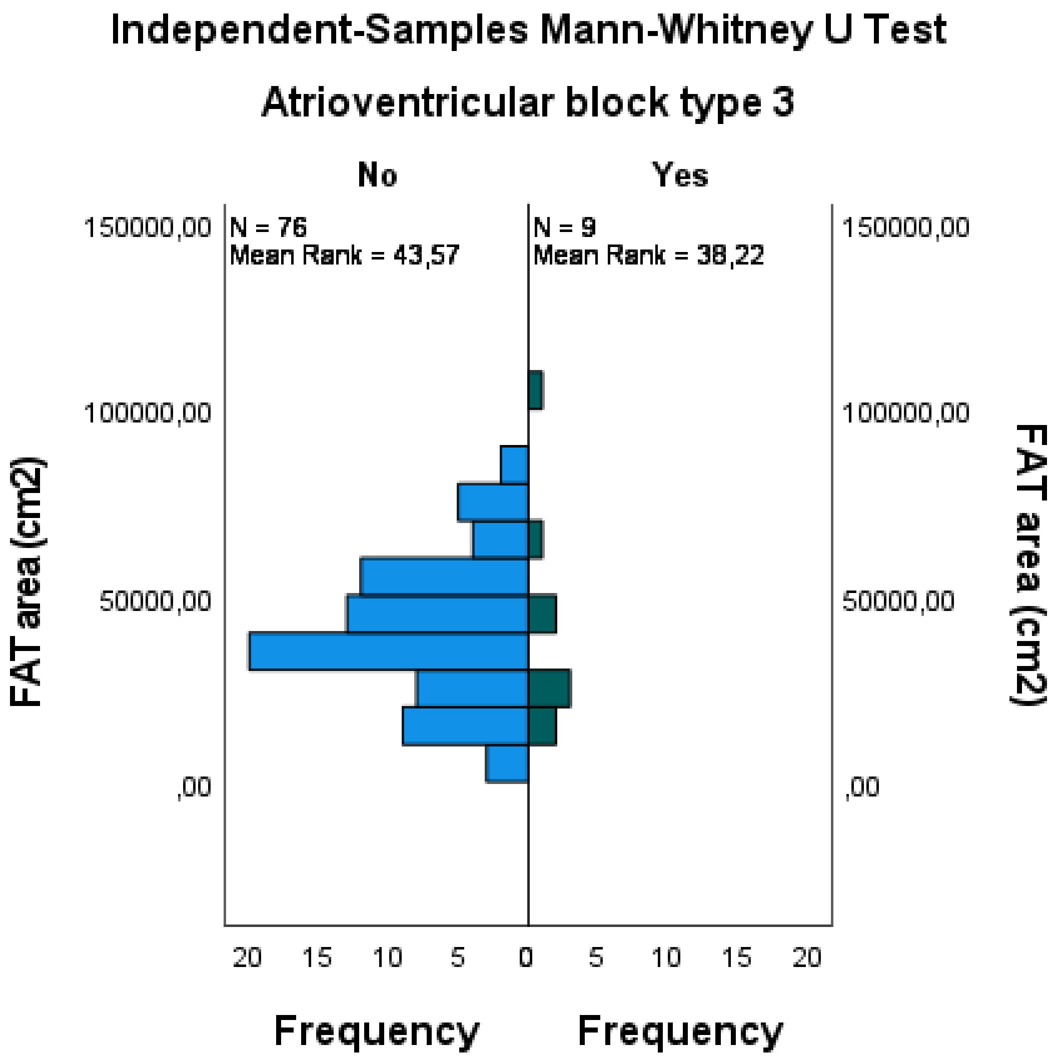

SAT area (cm2) across Atrioventricular block type 3

Independent-Samples Mann-Whitney U Test Summary

Total N	85
Mann-Whitney U	303,000
Wilcoxon W	348,000

Test Statistic	303,000
Standard Error	70,014
Standardized Test Statistic	-,557
Asymptotic Sig.(2-sided test)	,578

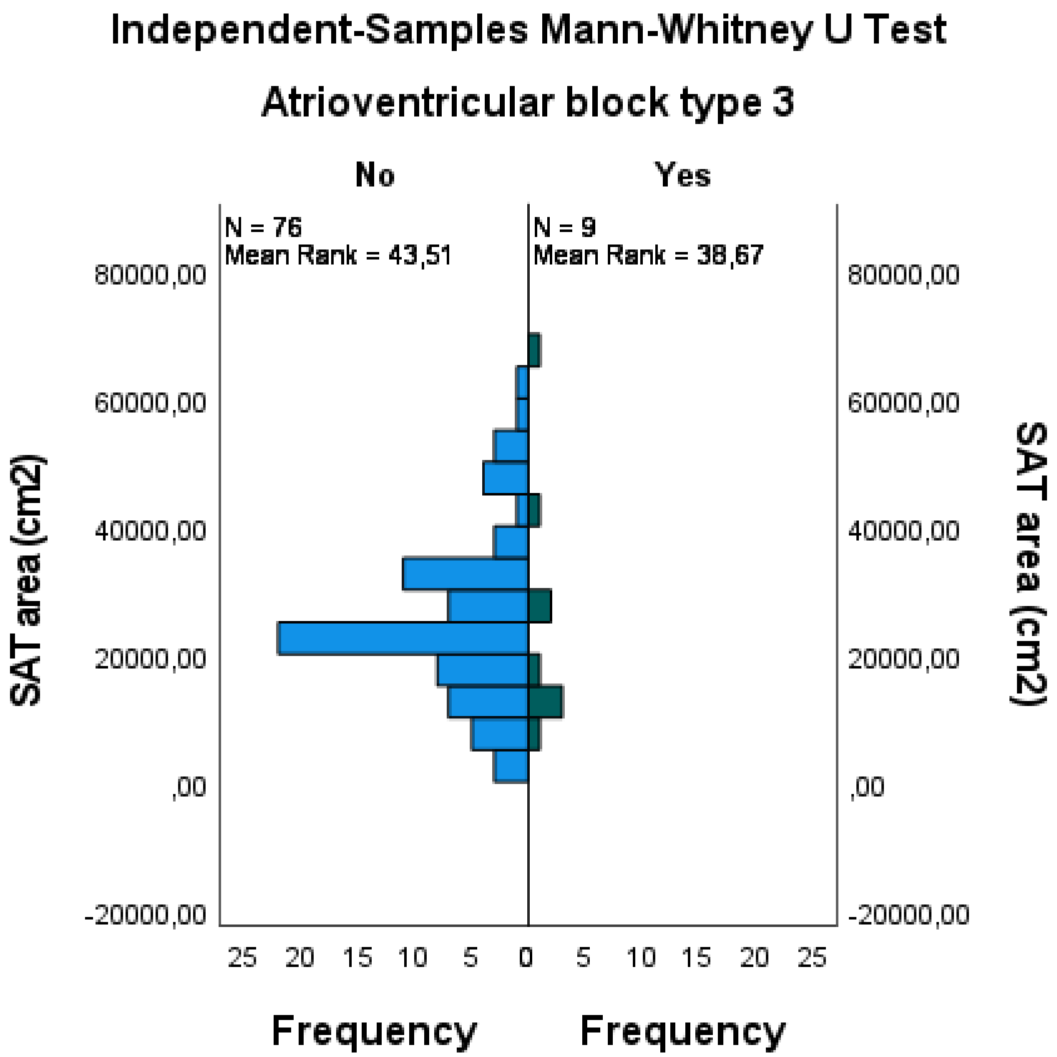

VAT area (cm2) across Atrioventricular block type 3

Independent-Samples Mann-Whitney U Test Summary

Total N	84
Mann-Whitney U	317,000
Wilcoxon W	362,000

Test Statistic	317,000
Standard Error	69,147
Standardized Test Statistic	-,296
Asymptotic Sig.(2-sided test)	,767

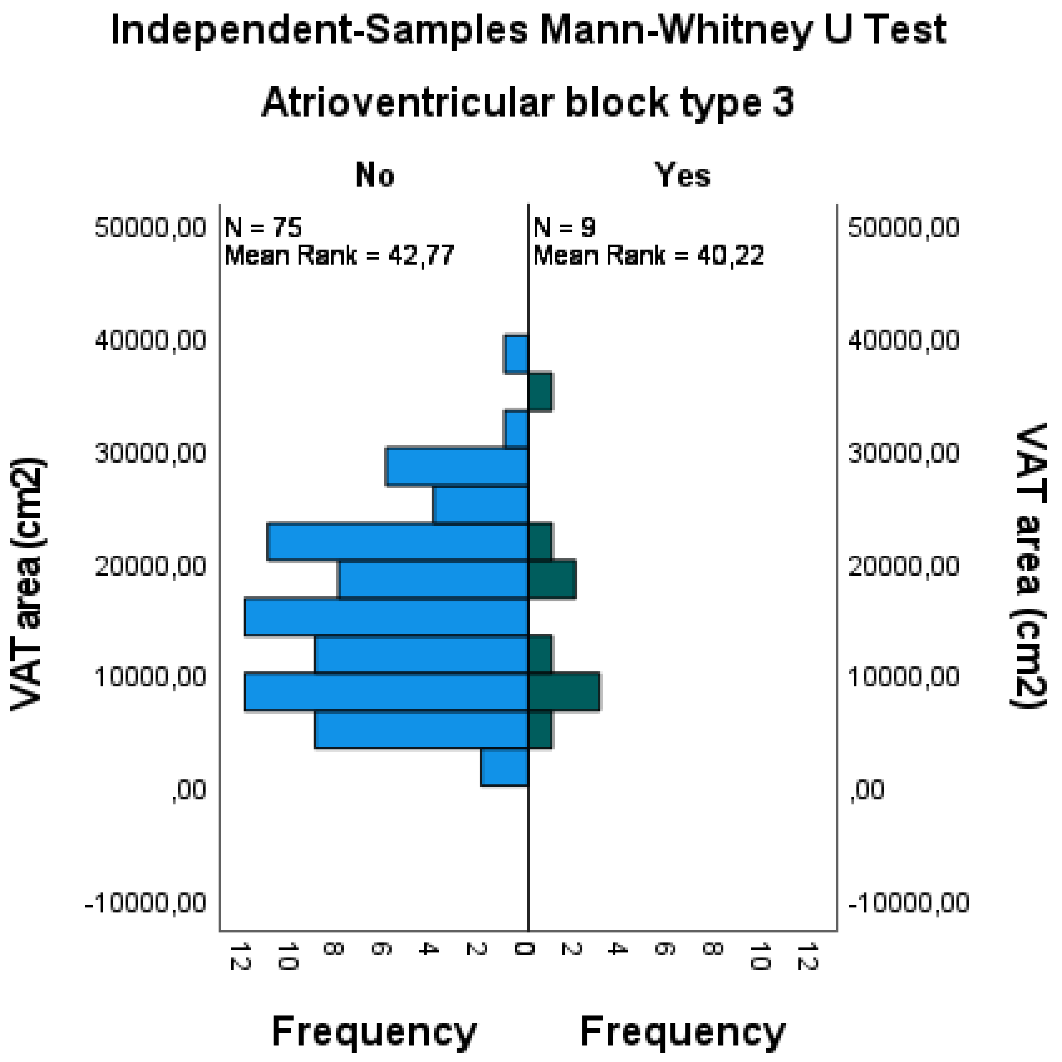

Right Psoas muscle area (cm2) across Atrioventricular block type 3

Independent-Samples Mann-Whitney U Test Summary

Total N	85
Mann-Whitney U	249,000
Wilcoxon W	294,000

Test Statistic	249,000
Standard Error	70,012
Standardized Test Statistic	-1,328
Asymptotic Sig.(2-sided test)	,184

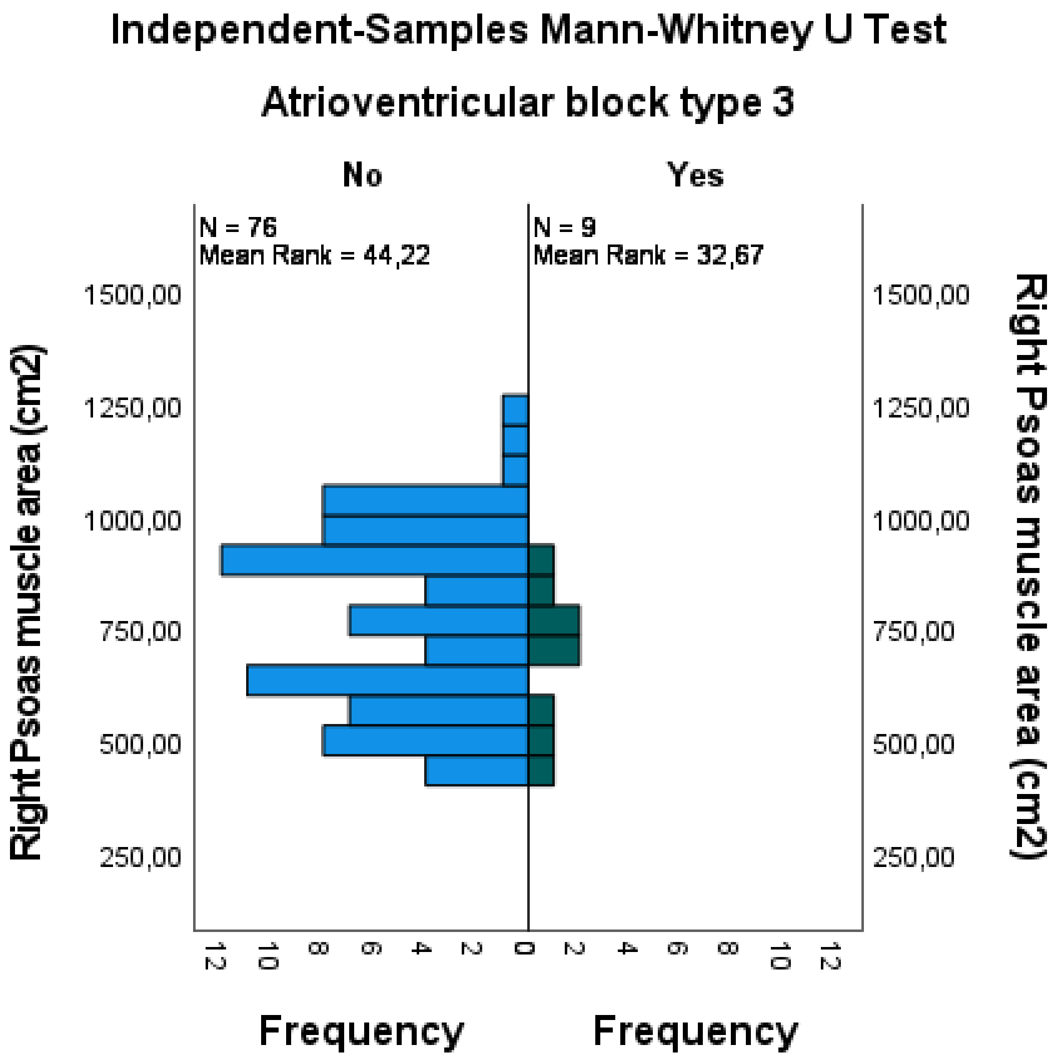

Left Psoas muscle area (cm2) across Atrioventricular block type 3

Independent-Samples Mann-Whitney U Test Summary

Total N	85
Mann-Whitney U	236,000
Wilcoxon W	281,000

Test Statistic	236,000
Standard Error	70,014
Standardized Test Statistic	-1,514
Asymptotic Sig.(2-sided test)	,130

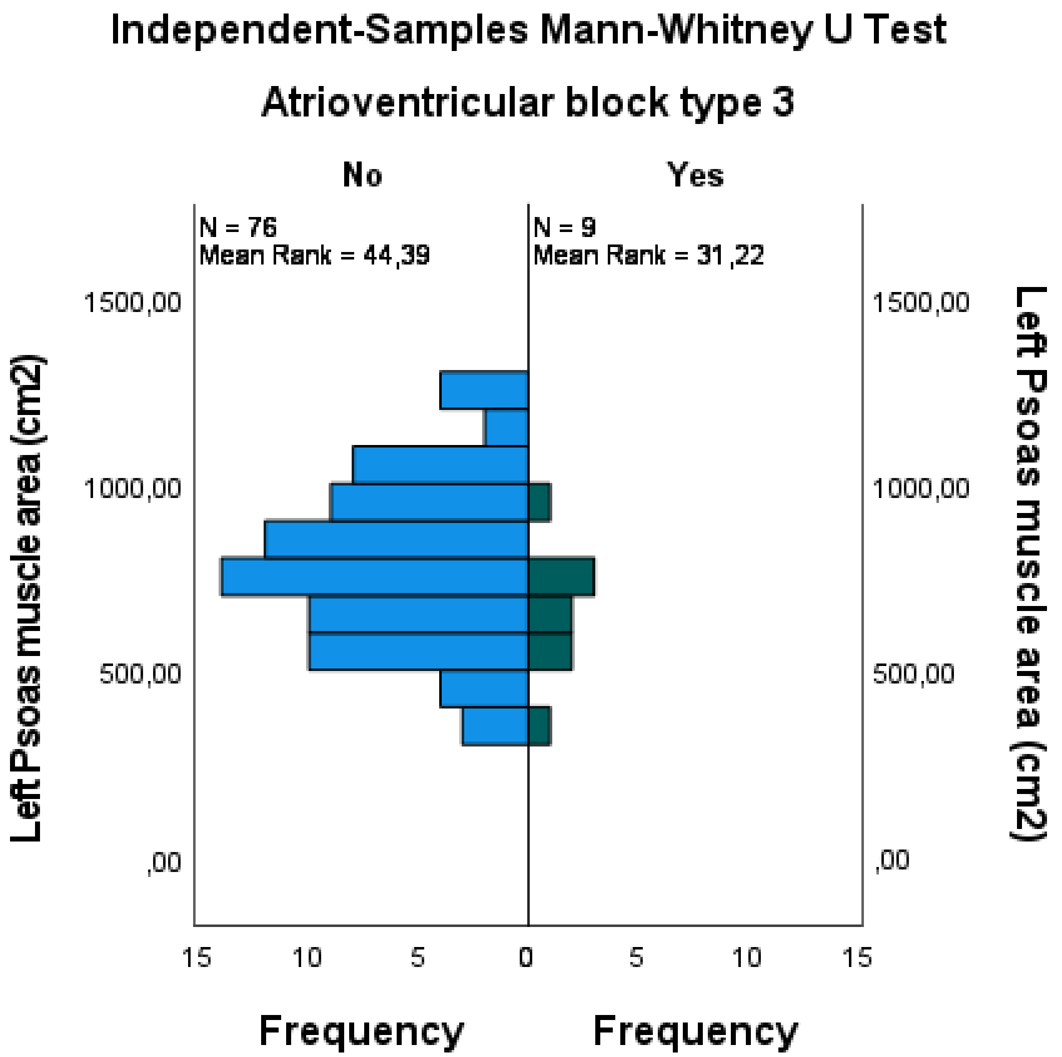

FAT mean density (HU) across Atrioventricular block type 3

Independent-Samples Mann-Whitney U Test Summary

Total N	85
Mann-Whitney U	399,500
Wilcoxon W	444,500

Test Statistic	399,500
Standard Error	70,003
Standardized Test Statistic	,821
Asymptotic Sig.(2-sided test)	,411

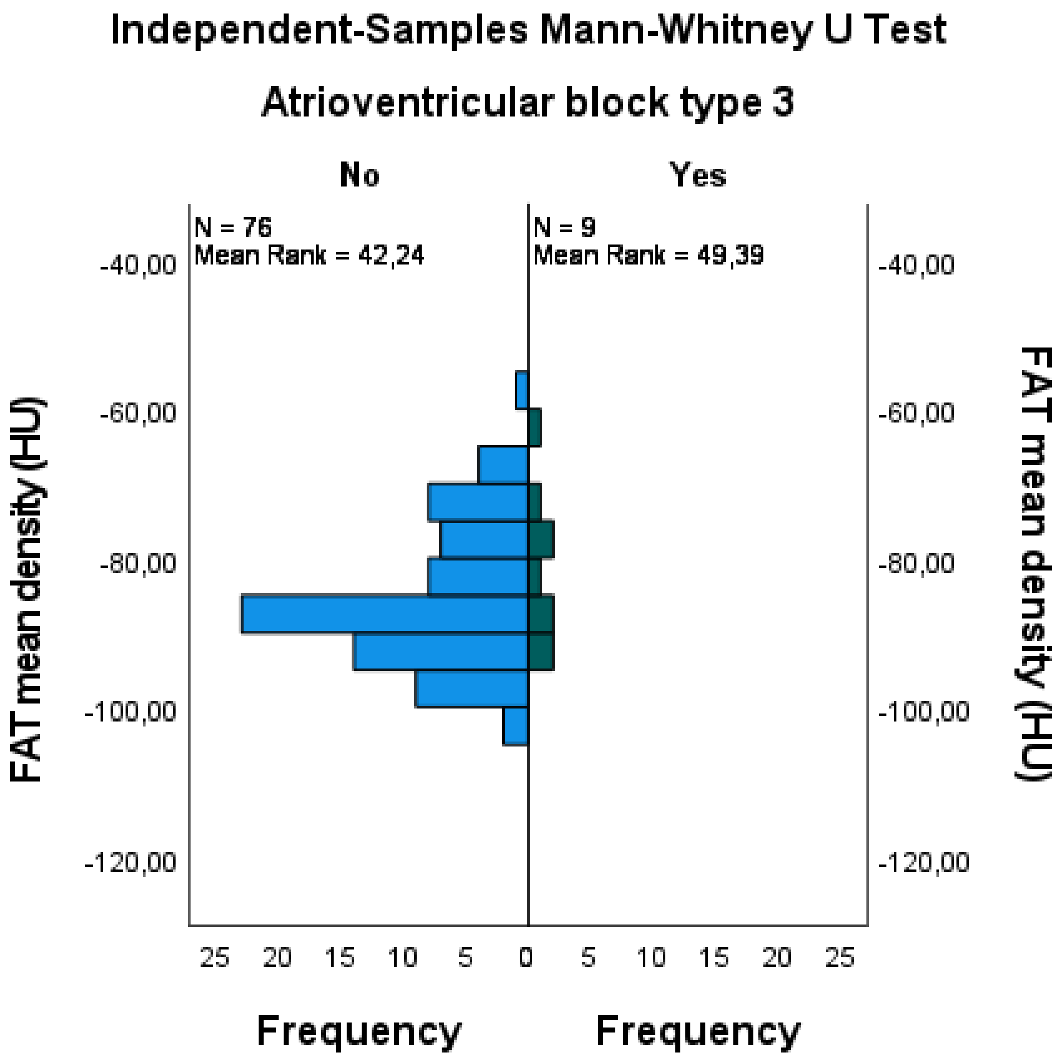

FAT median density (HU) across Atrioventricular block type 3

Independent-Samples Mann-Whitney U Test Summary

Total N	85
Mann-Whitney U	396,500
Wilcoxon W	441,500

Test Statistic	396,500
Standard Error	69,930
Standardized Test Statistic	,779
Asymptotic Sig.(2-sided test)	,436

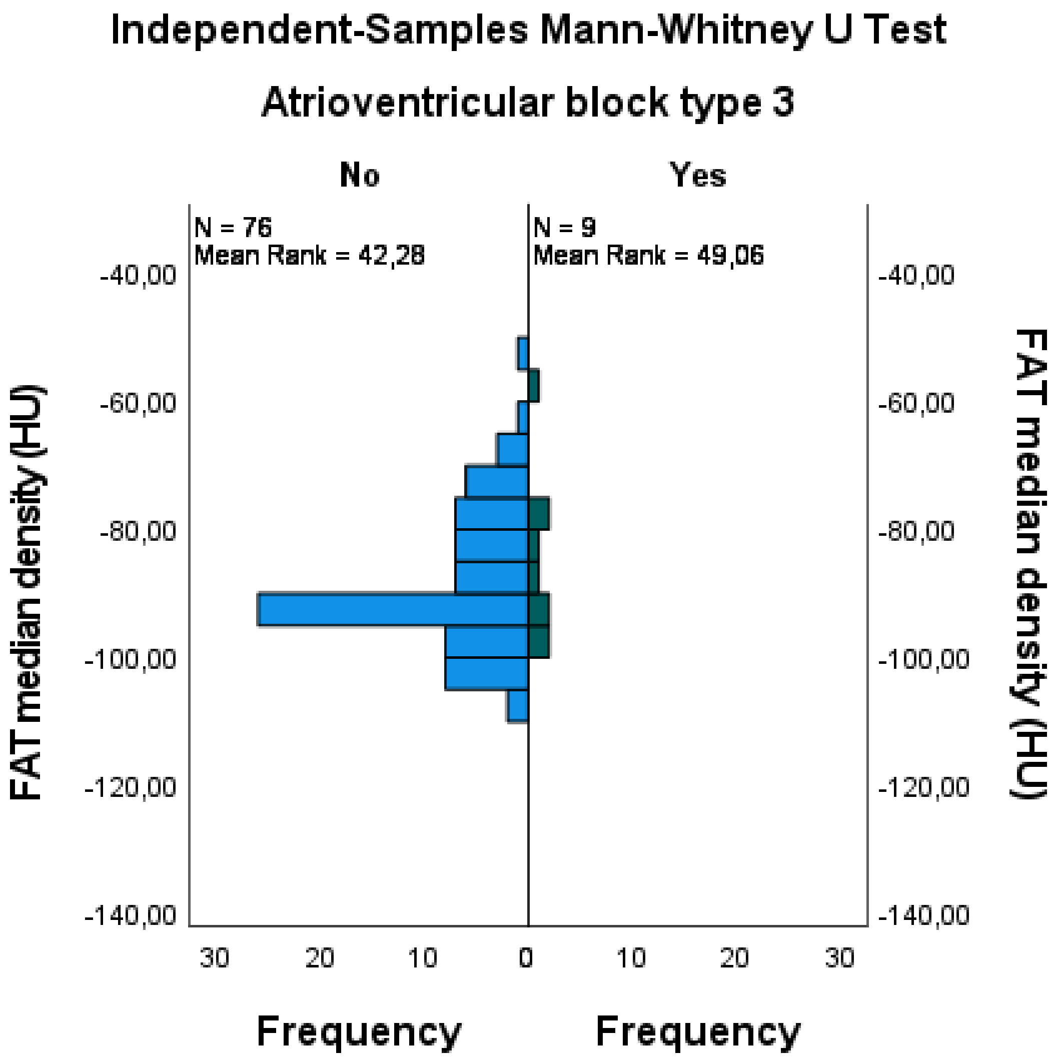

FAT density standard deviation across Atrioventricular block type 3

Independent-Samples Mann-Whitney U Test Summary

Total N	85
Mann-Whitney U	270,500
Wilcoxon W	315,500

Test Statistic	270,500
Standard Error	70,014
Standardized Test Statistic	-1,021
Asymptotic Sig.(2-sided test)	,307

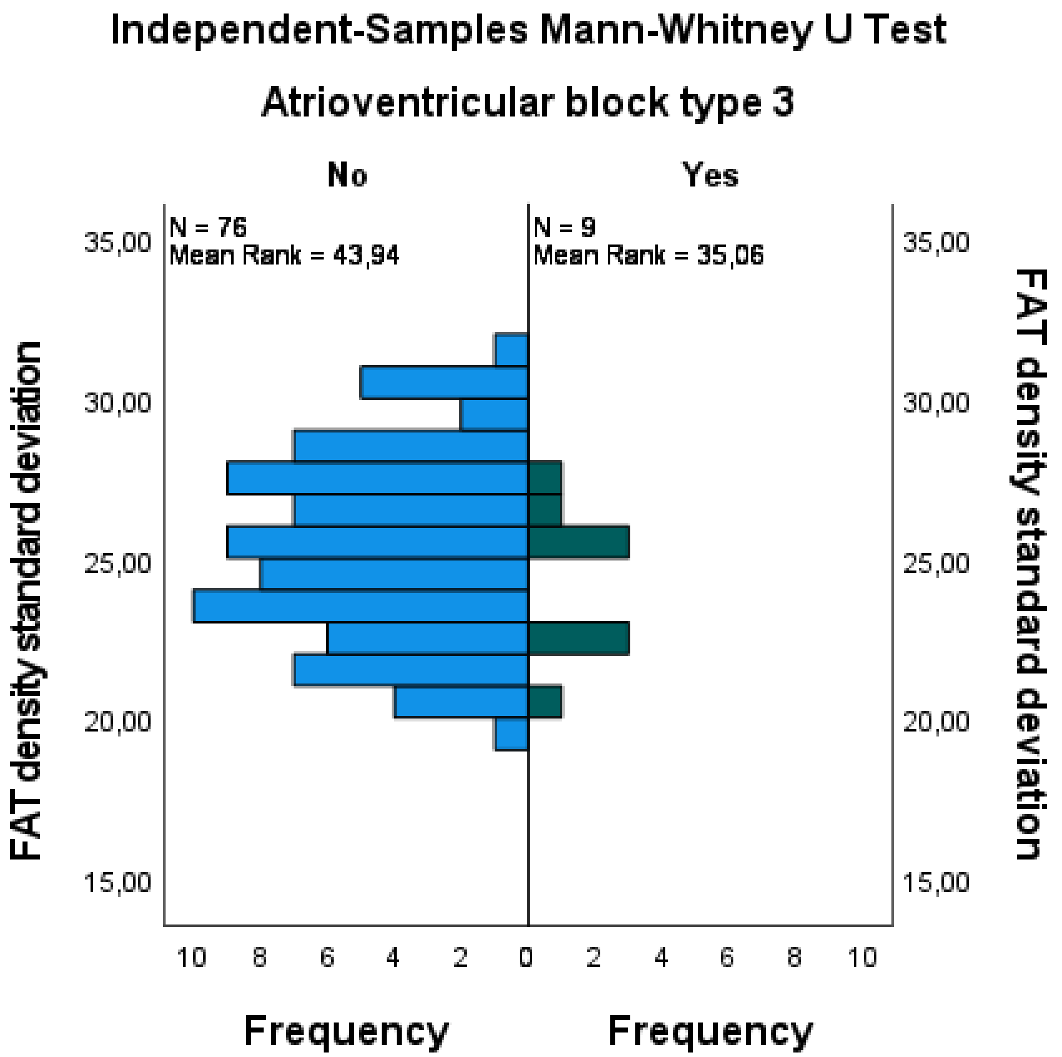

SAT mean density (HU) across Atrioventricular block type 3

Independent-Samples Mann-Whitney U Test Summary

Total N	85
Mann-Whitney U	381,500
Wilcoxon W	426,500

Test Statistic	381,500
Standard Error	70,006
Standardized Test Statistic	,564
Asymptotic Sig.(2-sided test)	,573

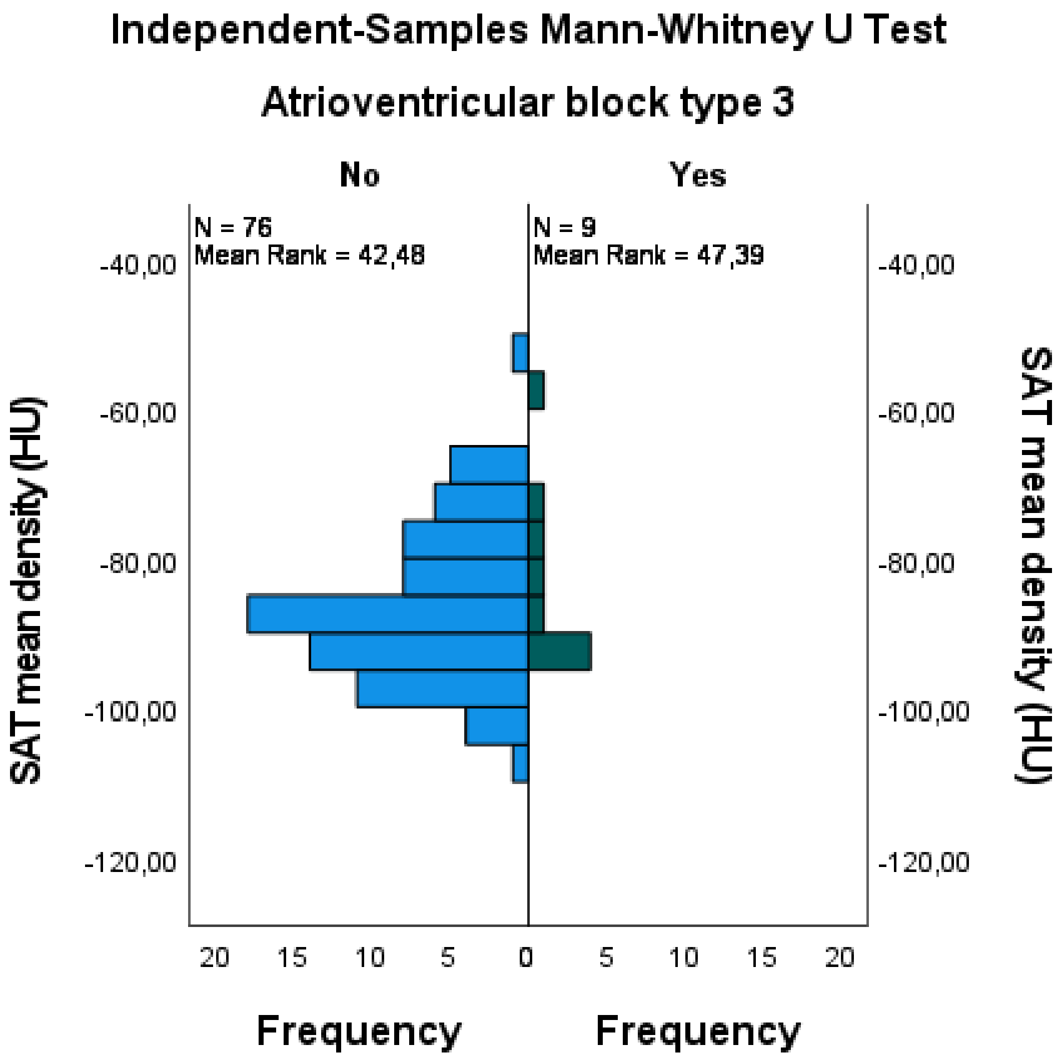

SAT median density (HU) across Atrioventricular block type 3

Independent-Samples Mann-Whitney U Test Summary

Total N	83
Mann-Whitney U	363,500
Wilcoxon W	408,500

Test Statistic	363,500
Standard Error	68,226
Standardized Test Statistic	,447
Asymptotic Sig.(2-sided test)	,655

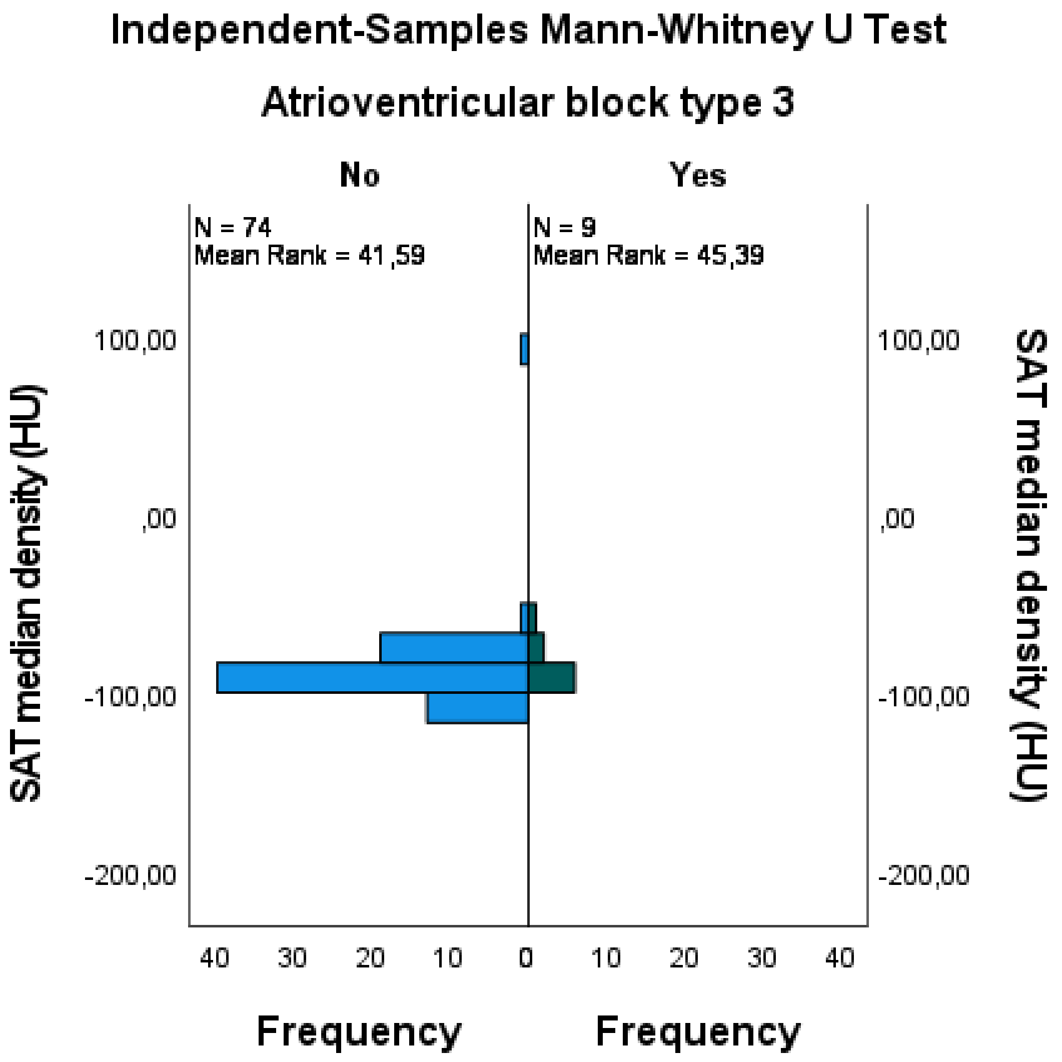

SAT density standard deviation across Atrioventricular block type 3

Independent-Samples Mann-Whitney U Test Summary

Total N	84
Mann-Whitney U	307,000
Wilcoxon W	352,000

Test Statistic	307,000
Standard Error	69,147
Standardized Test Statistic	-,441
Asymptotic Sig.(2-sided test)	,659

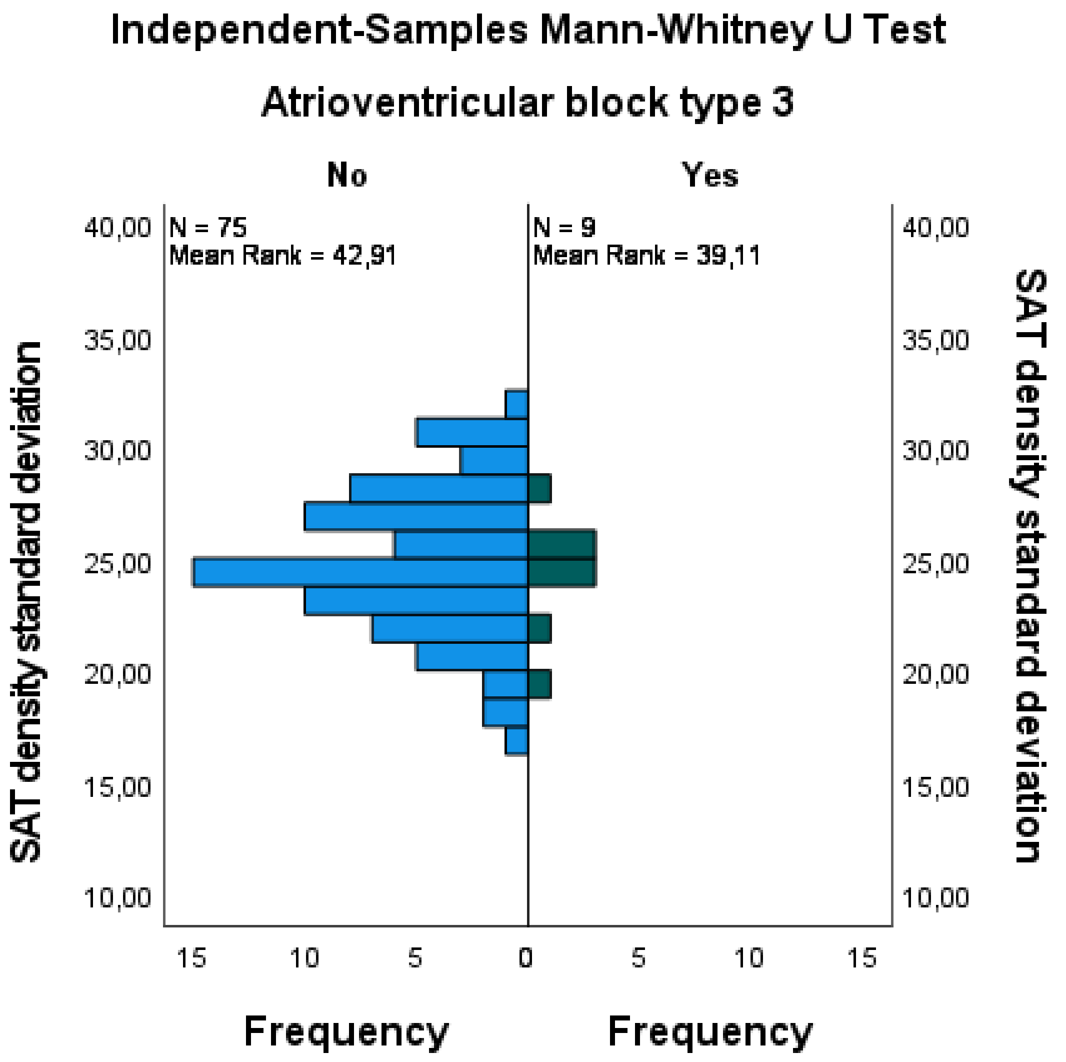

VAT mean density (HU) across Atrioventricular block type 3

Independent-Samples Mann-Whitney U Test Summary

Total N	85
Mann-Whitney U	370,500

Wilcoxon W	415,500
Test Statistic	370,500
Standard Error	70,002
Standardized Test Statistic	,407
Asymptotic Sig.(2-sided test)	,684

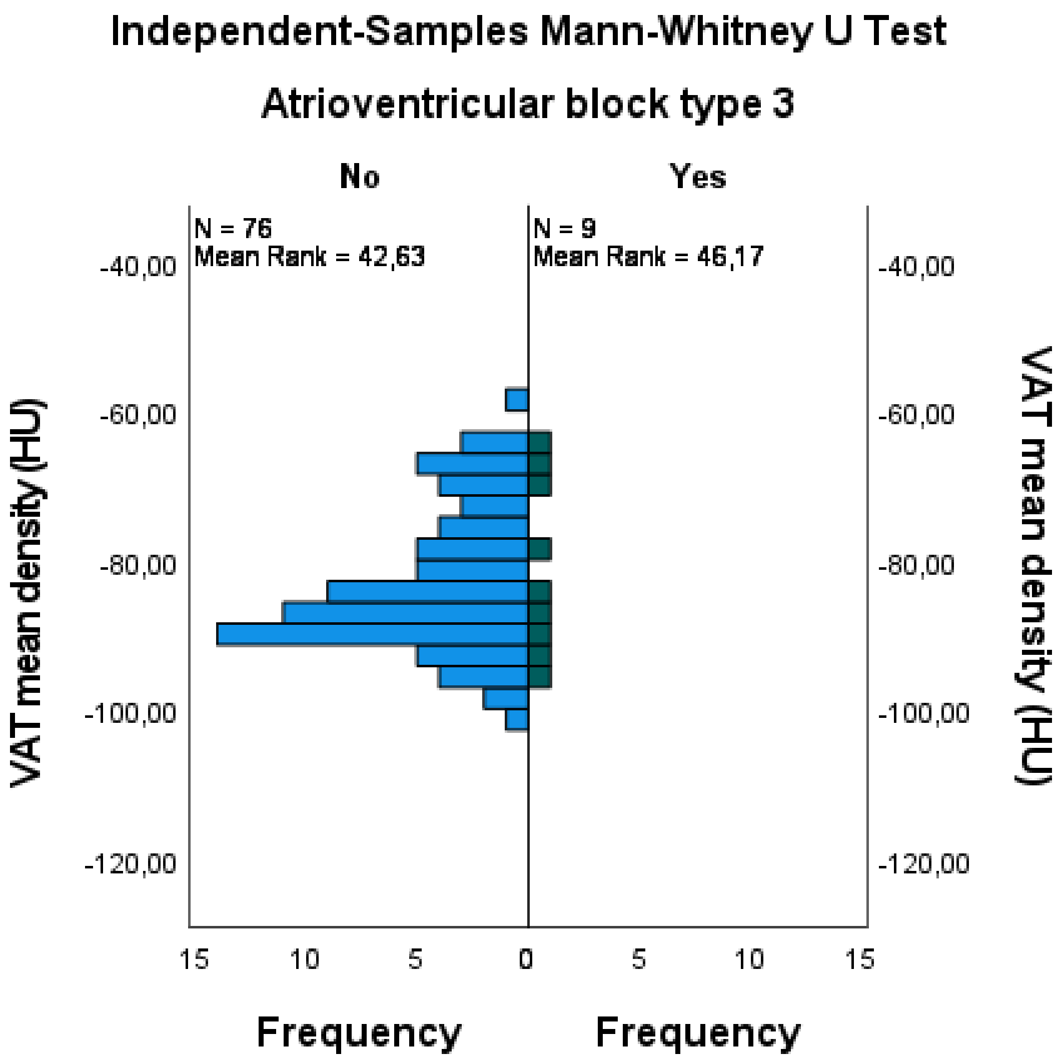

VAT median density (HU) across Atrioventricular block type 3

Independent-Samples Mann-Whitney U Test Summary

Total N	85
Mann-Whitney U	356,000

Wilcoxon W	401,000
Test Statistic	356,000
Standard Error	69,927
Standardized Test Statistic	,200
Asymptotic Sig.(2-sided test)	,841

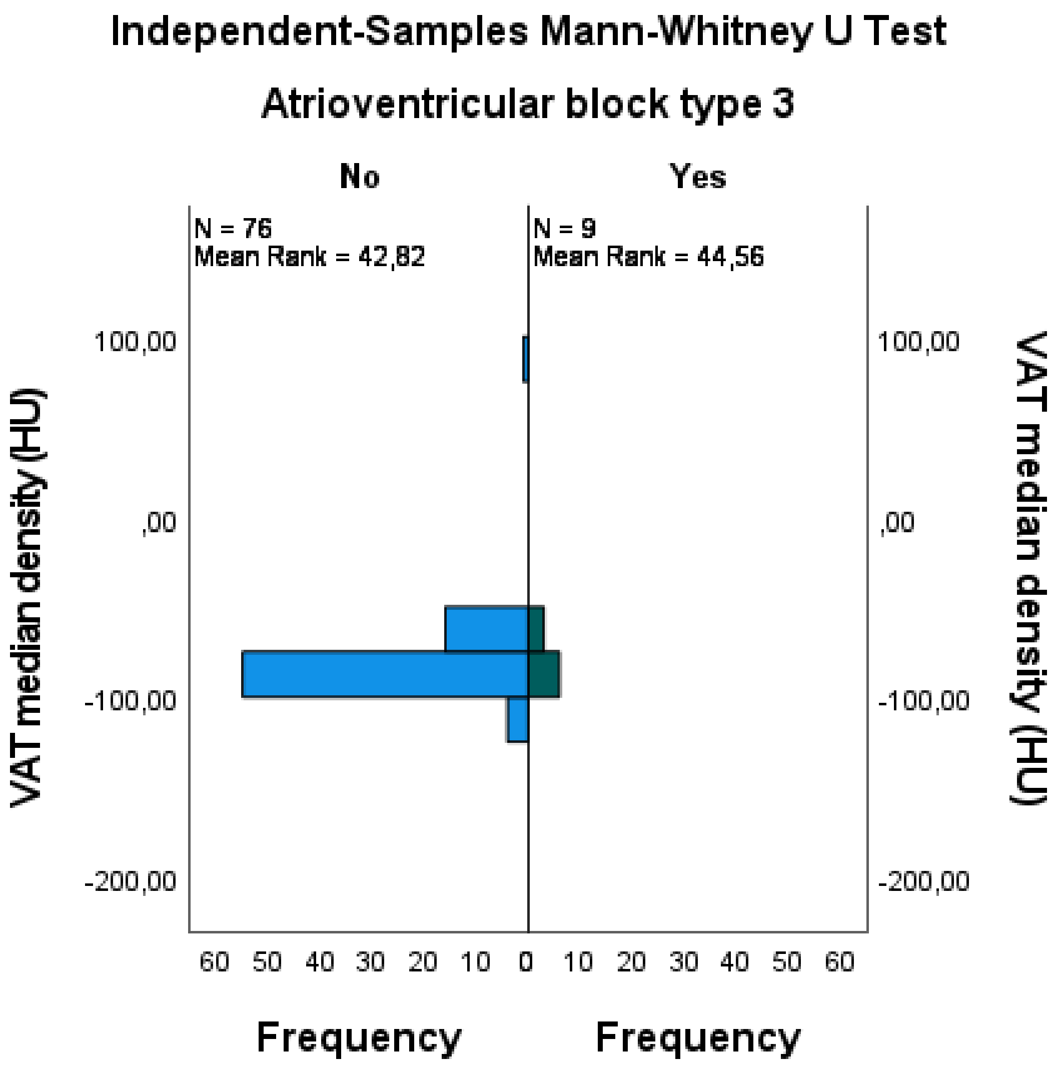

VAT density standard deviation across Atrioventricular block type 3

Independent-Samples Mann-Whitney U Test Summary

Total N	84
Mann-Whitney U	270,000

Wilcoxon W	315,000
Test Statistic	270,000
Standard Error	69,147
Standardized Test Statistic	-,976
Asymptotic Sig.(2-sided test)	,329

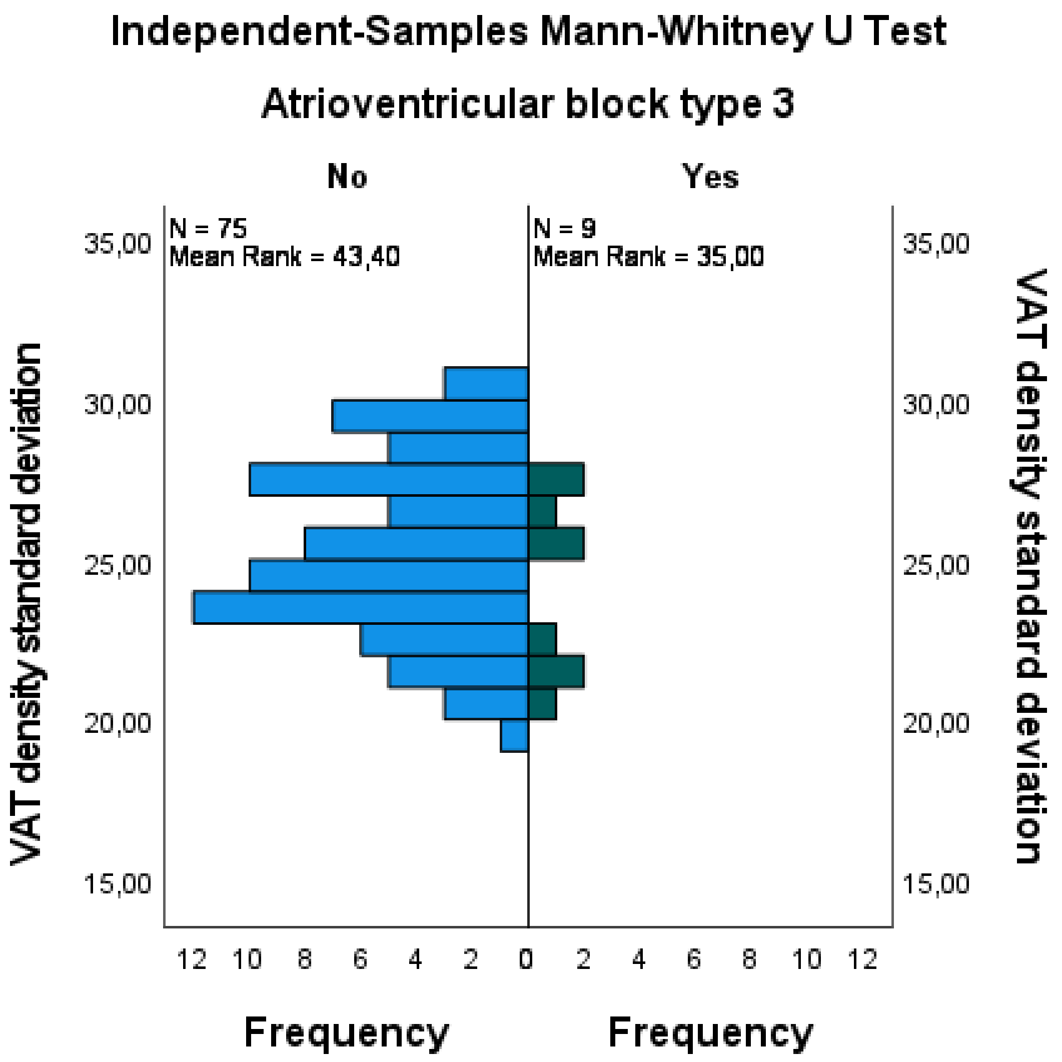

Right Psoas muscle mean density (HU) across Atrioventricular block type 3

Independent-Samples Mann-Whitney U Test Summary

Total N	85
Mann-Whitney U	165,500

Wilcoxon W	210,500
Test Statistic	165,500
Standard Error	69,999
Standardized Test Statistic	-2,521
Asymptotic Sig.(2-sided test)	,012

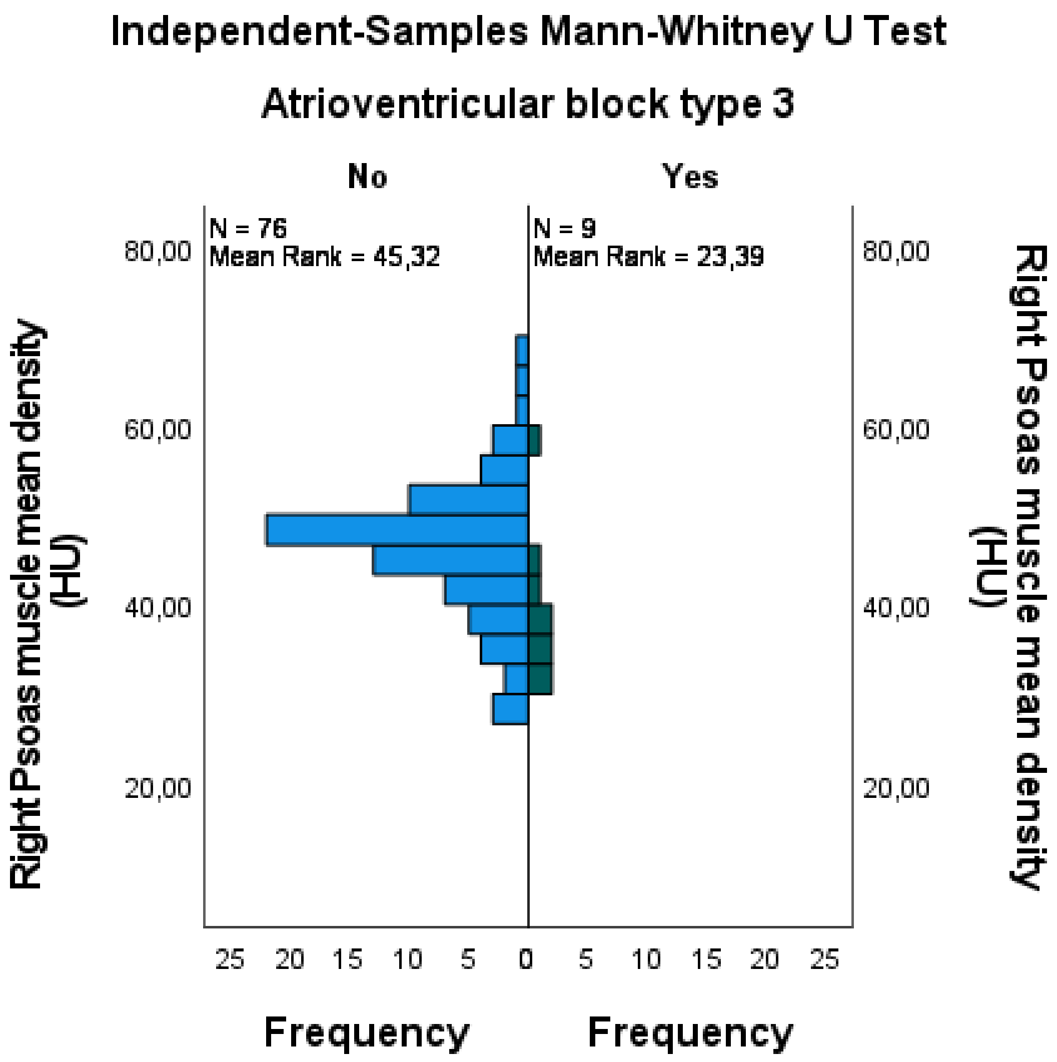

Right Psoas muscle median density (HU) across Atrioventricular block type 3

Independent-Samples Mann-Whitney U Test Summary

Total N	85
Mann-Whitney U	170,500

Wilcoxon W	215,500
Test Statistic	170,500
Standard Error	69,948
Standardized Test Statistic	-2,452
Asymptotic Sig.(2-sided test)	,014

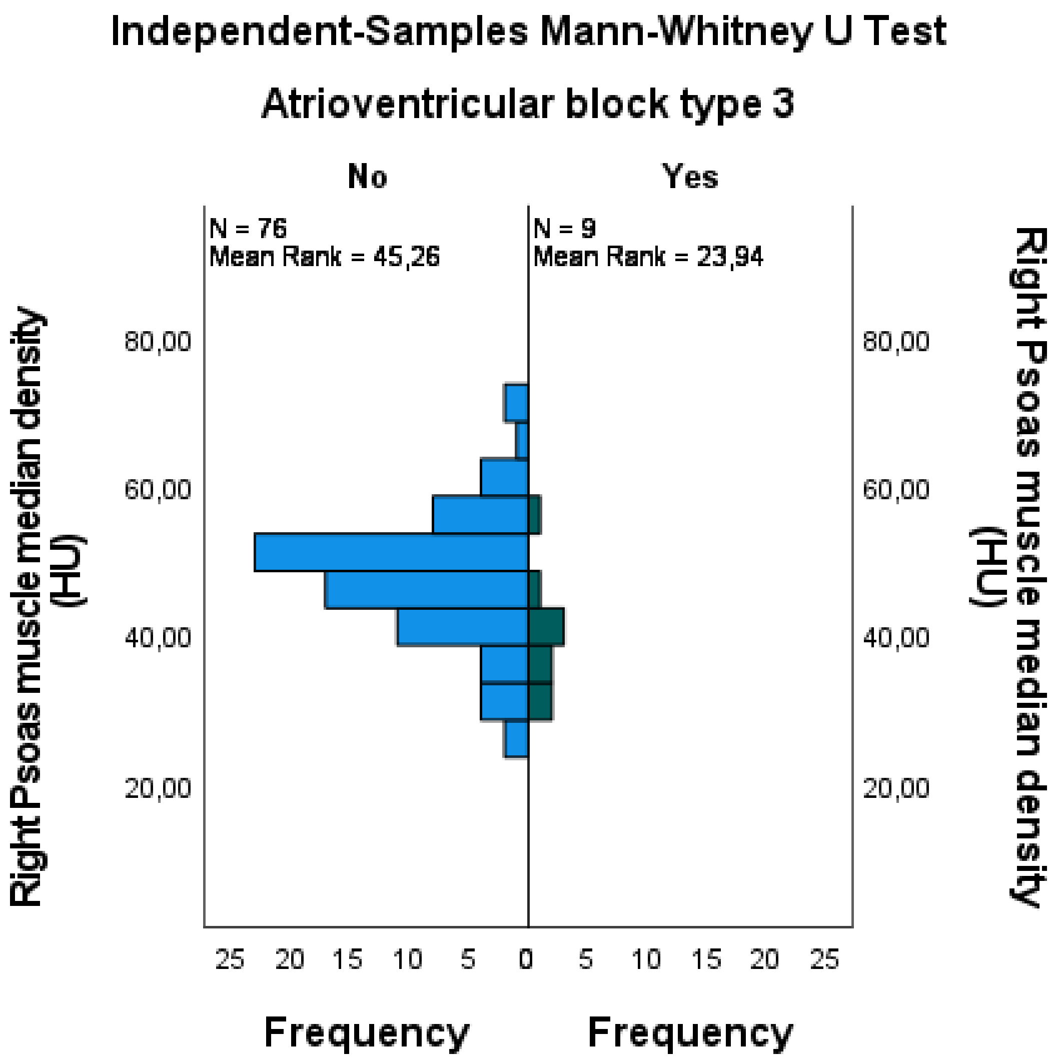

Right Psoas muscle density standard deviation across Atrioventricular block type 3

Independent-Samples Mann-Whitney U Test Summary

Total N	85
Mann-Whitney U	186,000

Wilcoxon W	231,000
Test Statistic	186,000
Standard Error	70,013
Standardized Test Statistic	-2,228
Asymptotic Sig.(2-sided test)	,026

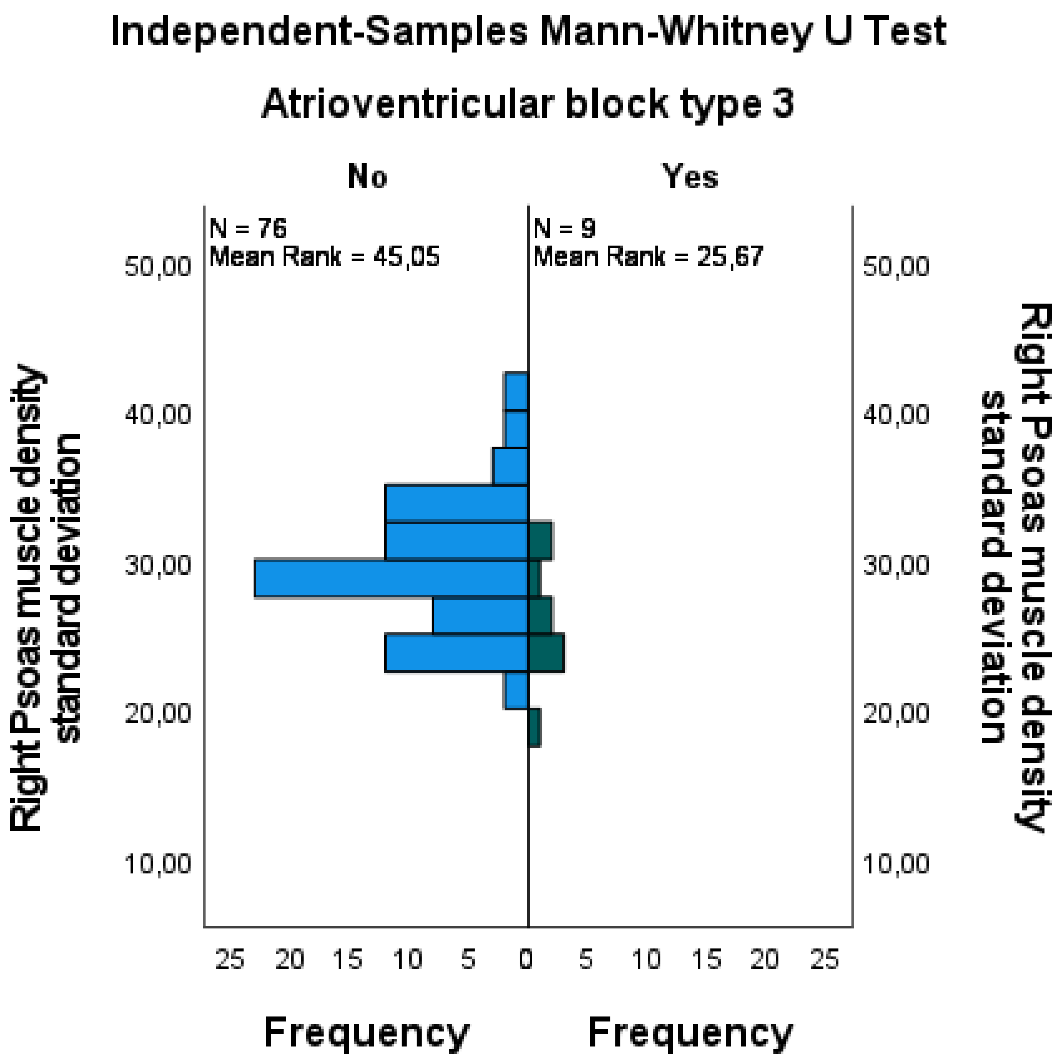

Left Psoas muscle mean density (HU) across Atrioventricular block type 3

Independent-Samples Mann-Whitney U Test Summary	
Total N	85
Mann-Whitney U	269,500

Wilcoxon W	314,500
Test Statistic	269,500
Standard Error	70,000
Standardized Test Statistic	-1,036
Asymptotic Sig.(2-sided test)	,300

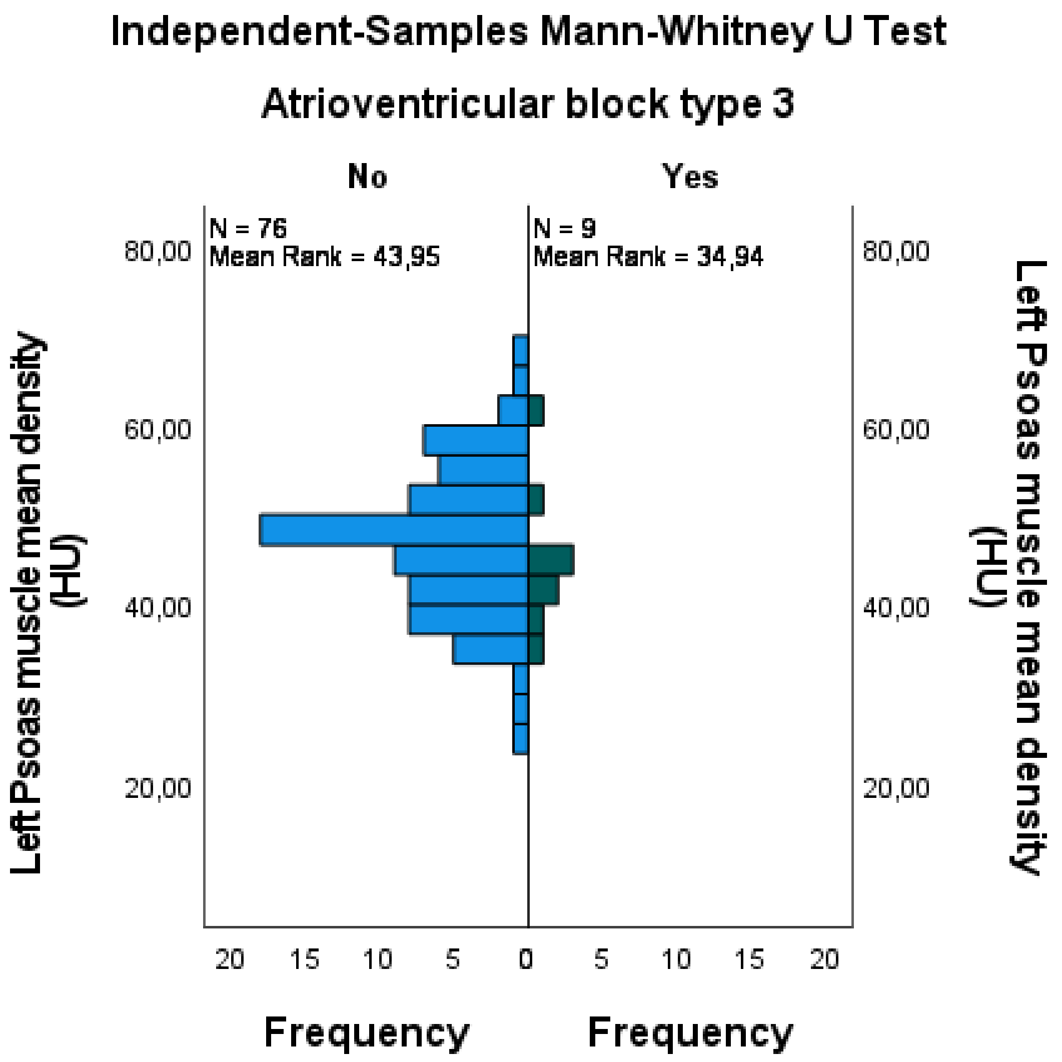

Left Psoas muscle median density (HU) across Atrioventricular block type 3

Independent-Samples Mann-Whitney U Test Summary

Total N	85
Mann-Whitney U	271,000

Wilcoxon W	316,000
Test Statistic	271,000
Standard Error	69,925
Standardized Test Statistic	-1,015
Asymptotic Sig.(2-sided test)	,310

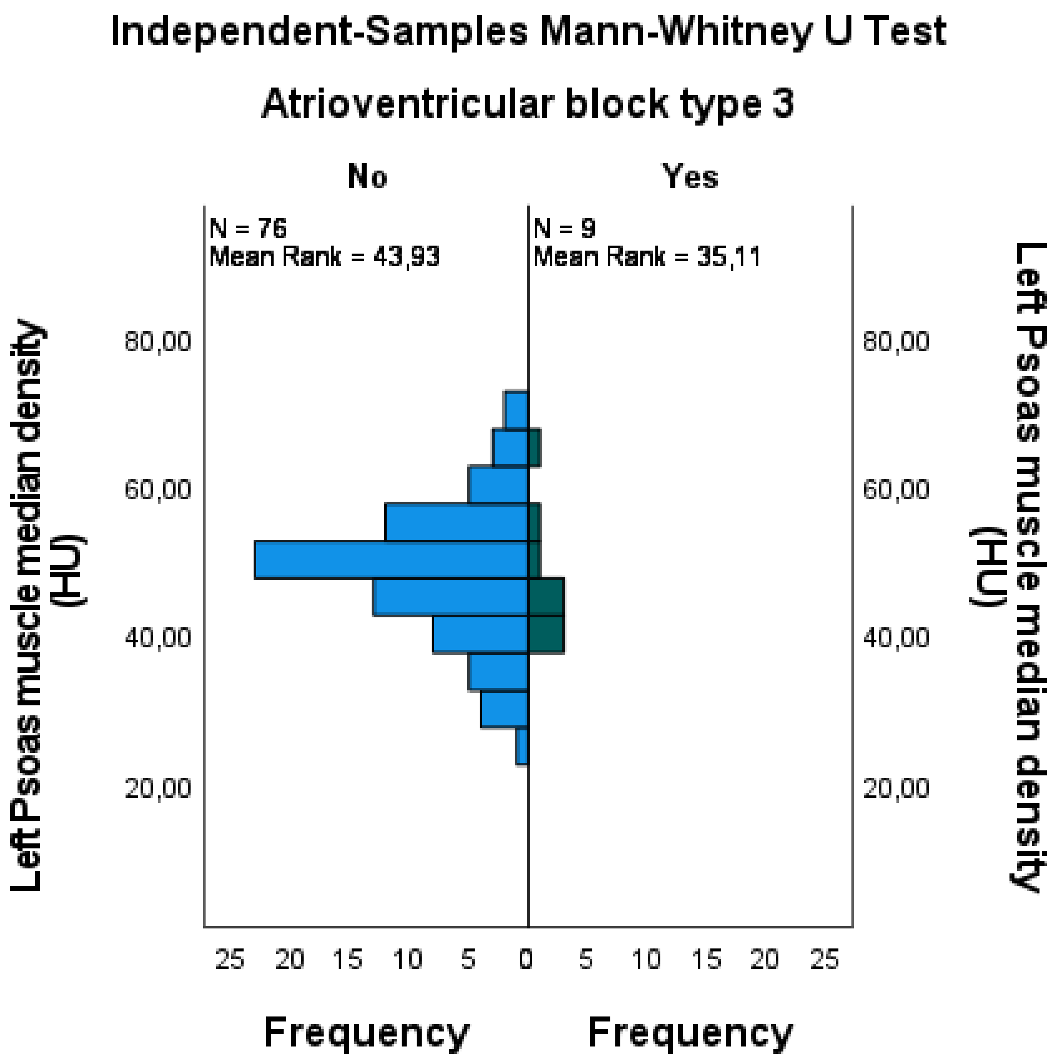

Left Psoas muscle density standard deviation across Atrioventricular block type 3

Independent-Samples Mann-Whitney U Test Summary

Total N	85
Mann-Whitney U	229,000

Wilcoxon W	274,000
Test Statistic	229,000
Standard Error	70,014
Standardized Test Statistic	-1,614
Asymptotic Sig.(2-sided test)	,107

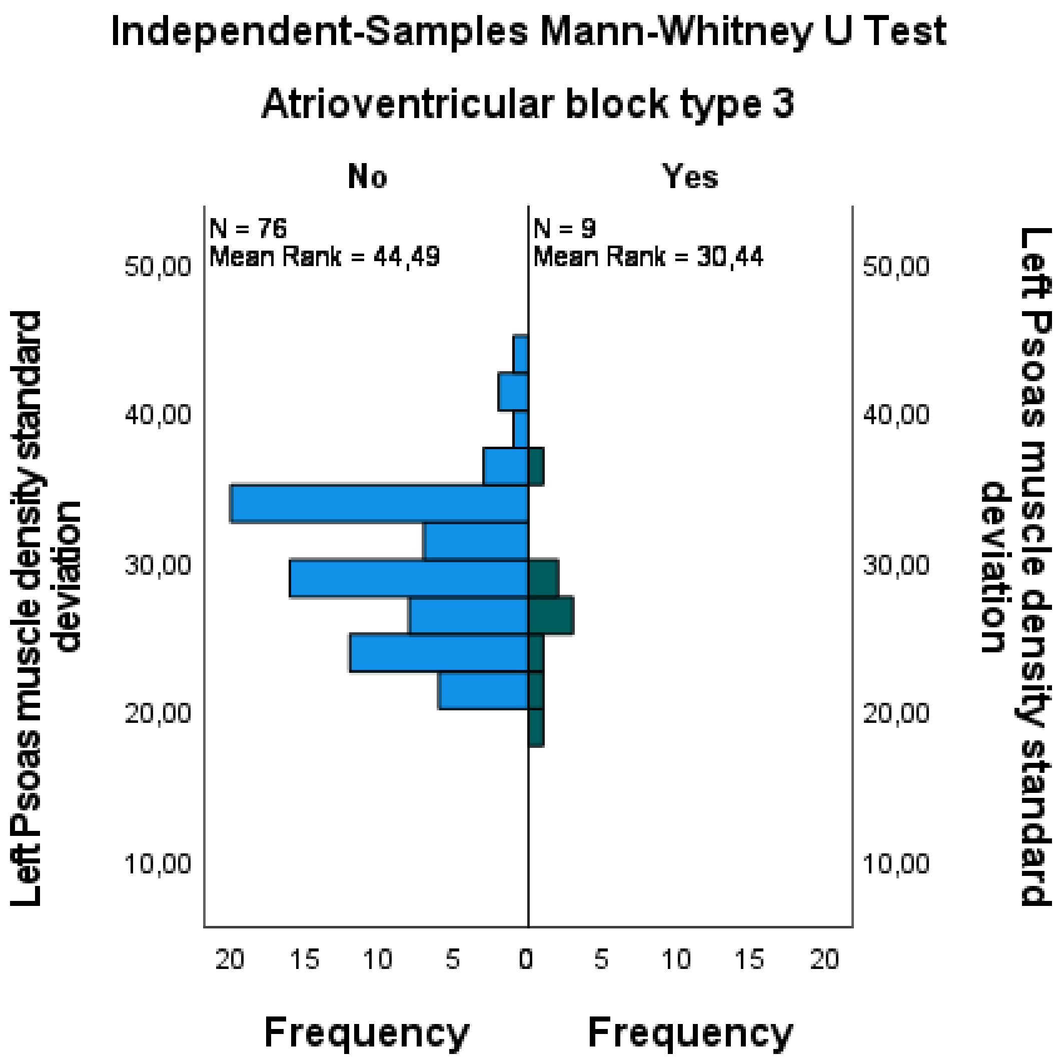

Independent-Samples Mann-Whitney U Test for hospitalization

Group Statistics

Hospitalization days after TAVI	N	Mean	Std. Deviation	Std. Error Mean

Psoas/height	<=5	50	13,66	3,18	,45
	>5	35	13,06	3,02	,51
Anterior SAT distance	<=5	50	26,16	11,78	1,67
	>5	35	23,09	12,20	2,06
Posterior SAT distance	<=5	50	56,59	16,01	2,26
	>5	35	45,83	15,92	2,69
Anterior+Posterior SAT distance	<=5	50	82,76	24,78	3,50
	>5	35	68,92	23,60	3,99
VAT distance	<=5	50	18,25	11,42	1,62
	>5	34	16,84	10,51	1,80
Right common femoral artery area (mm2)	<=5	50	57,48	22,84	3,23
	>5	35	55,14	19,25	3,25
Left common femoral artery area (mm2)	<=5	50	61,06	21,71	3,07
	>5	35	55,13	17,49	2,96
FAT area (cm2)	<=5	50	42759,98	18160,68	2568,31
	>5	35	38701,45	21092,35	3565,26
SAT area (cm2)	<=5	50	26725,85	12611,60	1783,55
	>5	35	23877,36	14004,87	2367,26
VAT area (cm2)	<=5	50	16089,47	7478,07	1057,56
	>5	34	14533,15	9026,27	1547,99
Right Psoas muscle area (cm2)	<=5	50	769,73	205,36	29,04
	>5	35	741,15	202,36	34,21
Left Psoas muscle area (cm2)	<=5	50	797,12	216,72	30,65
	>5	35	742,79	220,67	37,30
FAT mean density (HU)	<=5	50	-86,64	9,12	1,29
	>5	35	-83,29	10,22	1,73
FAT median density (HU)	<=5	50	-90,12	10,45	1,48
	>5	35	-85,89	11,64	1,97
FAT density standard deviation	<=5	50	24,99	2,71	,38
	>5	35	25,51	3,18	,54
SAT mean density (HU)	<=5	50	-87,74	10,21	1,44
	>5	35	-84,69	10,57	1,79
SAT median density (HU)	<=5	48	-87,69	29,14	4,21
	>5	35	-87,80	12,09	2,04
SAT density standard deviation	<=5	49	24,72	2,91	,42
	>5	35	24,92	3,51	,59
VAT mean density (HU)	<=5	50	-84,20	8,95	1,27
	>5	35	-80,71	10,83	1,83
VAT median density (HU)	<=5	50	-87,40	10,41	1,47
	>5	35	-77,26	31,74	5,36
VAT density standard deviation	<=5	49	24,98	2,82	,40
	>5	35	25,89	2,71	,46

Right Psoas muscle mean density (HU)	<=5	50	46,57	7,52	1,06
	>5	35	44,43	8,13	1,38
Right Psoas muscle median density (HU)	<=5	50	47,74	8,79	1,24
	>5	35	45,46	9,55	1,61
Right Psoas muscle density standard deviation	<=5	50	29,46	4,04	,57
	>5	35	29,09	5,05	,85
Left Psoas muscle mean density (HU)	<=5	50	47,52	7,82	1,11
	>5	35	45,87	8,92	1,51
Left Psoas muscle median density (HU)	<=5	50	48,82	8,22	1,16
	>5	35	46,80	10,19	1,72
Left Psoas muscle density standard deviation	<=5	50	29,13	4,78	,68
	>5	35	29,46	5,558	,94

Independent Samples Test

		Levene's Test for Equality of Variances		t-test for Equality of Means							
						Significance				95% Confidence Interval of the Difference	
		F	Sig.	t	df	One-Sided p	Two-Sided p	Mean Difference	Std. Error Difference	Lower	Upper
Psoas/height	Equal variances assumed	,796	,375	,866	83	,194	,389	,60	,69	-,77	1,96
	Equal variances not assumed			,874	75,623	,192	,385	,60	,68	-,76	1,95
Anterior SAT distance	Equal variances assumed	,012	,914	1,169	83	,123	,246	3,08	2,63	-2,16	8,32
	Equal variances not assumed			1,161	71,665	,125	,249	3,08	2,65	-2,21	8,36
Posterior SAT distance	Equal variances assumed	,022	,883	3,057	83	,002	,003	10,76	3,52	3,76	17,76

	Equal variances not assumed			3,060	73,589	,002	,003	10,76	3,52	3,75	17,77
Anterior+Posterior SAT distance	Equal variances assumed	,082	,775	2,584	83	,006	,012	13,84	5,36	3,19	24,49
	Equal variances not assumed			2,606	75,524	,006	,011	13,84	5,31	3,26	24,41
VAT distance	Equal variances assumed	,001	,976	,573	82	,284	,568	1,41	2,46	-3,48	6,30
	Equal variances not assumed			,582	74,804	,281	,562	1,41	2,42	-3,41	6,23
Right common femoral artery area (mm2)	Equal variances assumed	,551	,460	,496	83	,311	,621	2,34	4,73	-7,06	11,74
	Equal variances not assumed			,511	80,070	,305	,611	2,34	4,58	-6,78	11,47
Left common femoral artery area (mm2)	Equal variances assumed	,257	,613	1,338	83	,092	,185	5,92	4,43	-2,88	14,73
	Equal variances not assumed			1,390	81,281	,084	,168	5,92	4,26	-2,56	14,40
FAT area (cm2)	Equal variances assumed	,434	,512	,948	83	,173	,346	4058,54	4278,90	- 4452,03	12569,10
	Equal variances not assumed			,924	66,094	,180	,359	4058,549	4394,00	- 4714,14	12831,21
SAT area (cm2)	Equal variances assumed	,047	,829	,979	83	,165	,330	2848,49	2909,17	- 2937,73	8634,70

	Equal variances not assumed			,961	68,288	,170	,340	2848,49	2963,94	- 3065,52	8762,49
VAT area (cm2)	Equal variances assumed	2,241	,138	,860	82	,196	,392	1556,33	1808,67	- 2041,70	5154,35
	Equal variances not assumed			,830	61,910	,205	,410	1556,33	1874,76	- 2191,37	5304,02
Right Psoas muscle area (cm2)	Equal variances assumed	,185	,668	,635	83	,264	,527	28,58	44,99	-60,90	118,06
	Equal variances not assumed			,637	74,005	,263	,526	28,5	44,87	-60,83	117,99
Left Psoas muscle area (cm2)	Equal variances assumed	,003	,958	1,129	83	,131	,262	54,33	48,12	-41,38	150,04
	Equal variances not assumed			1,125	72,484	,132	,264	54,33	48,28	-41,90	150,56
FAT mean density (HU)	Equal variances assumed	1,401	,240	- 1,584	83	,059	,117	-3,35	2,11229	-7,55	,86
	Equal variances not assumed			- 1,552	67,835	,063	,125	-3,35	2,16	-7,65	,96
FAT median density (HU)	Equal variances assumed	1,200	,276	- 1,754	83	,042	,083	-4,23	2,41	-9,04	,57
	Equal variances not assumed			- 1,721	68,145	,045	,090	-4,23	2,46	-9,14	,68
FAT density standard deviation	Equal variances assumed	1,438	,234	-,796	83	,214	,428	-,51	,64	-1,79	,77

	Equal variances not assumed			-,774	65,612	,221	,442	-,51	6	-1,83	,81
SAT mean density (HU)	Equal variances assumed	,315	,576	-1,339	83	,092	,184	-3,06	2,28	-7,60	1,48
	Equal variances not assumed			-1,331	71,721	,094	,188	-3,06	2,30	-7,64	1,52
SAT median density (HU)	Equal variances assumed	,768	,383	,022	81	,491	,983	,11	5,23	-10,30	10,52
	Equal variances not assumed			,024	66,671	,490	,981	,11	4,68	-9,22	9,45
SAT density standard deviation	Equal variances assumed	2,050	,156	-,292	82	,385	,771	-,205	,70	-1,60	1,19
	Equal variances not assumed			-,283	64,557	,389	,778	-,205	,73	-1,65	1,24
VAT mean density (HU)	Equal variances assumed	3,997	,049	-1,619	83	,055	,109	-3,48	2,15	-7,77	,80
	Equal variances not assumed			-1,565	64,112	,061	,122	-3,48	2,23	-7,93	,96
VAT median density (HU)	Equal variances assumed	4,496	,037	-2,108	83	,019	,038	-10,14	4,81	-19,71	-,57
	Equal variances not assumed			-1,823	39,161	,038	,076	-10,14	5,56	-21,39	1,11
VAT density standard deviation	Equal variances assumed	,059	,809	-1,478	82	,072	,143	-,91	,61	-2,13	,31

	Equal variances not assumed			-1,488	75,147	,070	,141	-,91	,61	-2,12	,31
Right Psoas muscle mean density (HU)	Equal variances assumed	,601	,440	1,246	83	,108	,216	2,13513	1,71	-1,27	5,54
	Equal variances not assumed			1,229	69,564	,112	,223	2,14	1,74	-1,33	5,60
Right Psoas muscle median density (HU)	Equal variances assumed	,622	,433	1,137	83	,129	,259	2,28	2,01	-1,71	6,28
	Equal variances not assumed			1,121	69,386	,133	,266	2,28	2,04	-1,78	6,35
Right Psoas muscle density standard deviation	Equal variances assumed	1,901	,172	,371	83	,356	,712	,37	,99	-1,60	2,33
	Equal variances not assumed			,357	62,619	,361	,723	,37	1,03	-1,69	2,42
Left Psoas muscle mean density (HU)	Equal variances assumed	,441	,509	,906	83	,184	,368	1,65	1,83	-1,98	5,29
	Equal variances not assumed			,885	67,019	,190	,379	1,65	1,87	-2,08	5,39
Left Psoas muscle median density (HU)	Equal variances assumed	1,422	,236	1,009	83	,158	,316	2,02	2,00	-1,96	6,00
	Equal variances not assumed			,972	62,987	,167	,335	2,02	2,08	-2,13	6,17
Left Psoas muscle density standard deviation	Equal variances assumed	,316	,575	-,299	83	,383	,766	-,34	1,13	-2,58	1,90

	Equal variances not assumed			-,291	65,988	,386	,772	-,34	1,16	-2,65	1,97

The Levene test is accepted ($p > 0.05$) in almost all cases, except ($p < 0.05$) for the VAT mean density (HU) and VAT median density (HU) variables, for which the robust t-test will be performed.

The difference between the means of the two groups is statistically different ($p < 0.05$) only for the Posterior SAT distance, Anterior + Posterior SAT density and VAT median density (HU) variables. For the other variables, however, there are no statistically significant differences ($p > 0.05$) between the two groups.

With regard to the three variables for which a difference was found, the mean is higher in the group <= 5 for the Posterior SAT distance and Anterior + Posterior SAT distance variables, while for the VAT median density (HU) variable the mean is highest in the> 5 group.

Independent-Samples Mann-Whitney U Test for peri-prosthetic endoleak

Group Statistics

	Peri-prosthetic endoleak	N	Mean	Std. Deviation	Std. Error Mean
Psoas/height	No	49	13,64	3,49	,50
	Yes	36	13,11	2,51	,42
Anterior SAT distance	No	49	25,44	12,70	1,82
	Yes	36	24,16	11,05	1,84
Posterior SAT distance	No	49	53,44	17,08	2,44
	Yes	36	50,43	16,35	2,72
Anterior+Posterior SAT distance	No	49	78,87	25,50	3,64
	Yes	36	74,60	24,71	4,12
VAT distance	No	48	19,17	12,05	1,74
	Yes	36	15,69	9,26	1,54
Right common femoral artery area (mm2)	No	49	55,15	19,75	2,82
	Yes	36	58,38	23,49	3,92
Left common femoral artery area (mm2)	No	49	56,79	17,52	2,50
	Yes	36	61,11	23,37	3,89
FAT area (cm2)	No	49	43124,26	20301,86	2900,27
	Yes	36	38318,36	18018,02	3003,00
SAT area (cm2)	No	49	27119,50	13674,27	1953,47
	Yes	36	23420,69	12386,66	2064,44
VAT area (cm2)	No	48	15883,93	8818,26	1272,81
	Yes	36	14893,67	7177,00	1196,17
Right Psoas muscle area (cm2)	No	49	735,11	210,45	30,06
	Yes	36	789,06	191,92	31,99
Left Psoas muscle area (cm2)	No	49	757,10	224,80	32,12
	Yes	36	798,78	210,85	35,14
FAT mean density (HU)	No	49	-85,67	9,17	1,31
	Yes	36	-84,69	10,42	1,74
FAT median density (HU)	No	49	-88,88	10,38	1,48
	Yes	36	-87,69	12,10	2,02
FAT density standard deviation	No	49	25,41	2,95	,42
	Yes	36	24,93	2,86	,48
SAT mean density (HU)	No	49	-86,87	9,96	1,42
	Yes	36	-85,96	11,11	1,85
SAT median density (HU)	No	47	-90,28	11,21	1,64
	Yes	36	-84,42	33,12	5,52
SAT density standard deviation	No	49	25,23	3,26	,47
	Yes	35	24,21	2,95	,50

VAT mean density (HU)	No	49	-83,35	9,65	1,38
	Yes	36	-81,97	10,22	1,70
VAT median density (HU)	No	49	-85,98	11,32	1,62
	Yes	36	-79,47	31,46	5,24
VAT density standard deviation	No	48	25,65	2,73	,40
	Yes	36	24,98	2,86	,48
Right Psoas muscle mean density (HU)	No	49	44,53	8,34	1,19
	Yes	36	47,26	6,81	1,14
Right Psoas muscle median density (HU)	No	49	45,39	10,06	1,44
	Yes	36	48,72	7,39	1,23
Right Psoas muscle density standard deviation	No	49	30,28	3,97	,57
	Yes	36	27,98	4,80	,80
Left Psoas muscle mean density (HU)	No	49	46,00	9,37	1,34
	Yes	36	47,99	6,46	1,08
Left Psoas muscle median density (HU)	No	49	46,82	10,61	1,52
	Yes	36	49,58	6,24	1,04
Left Psoas muscle density standard deviation	No	49	29,76	4,98	,71
	Yes	36	28,59	5,21	,87

Independent Samples Test

		Levene's Test for Equality of Variances		t-test for Equality of Means							
						Significance				95% Confidence Interval of the Difference	
		F	Sig.	t	df	One-Sided p	Two-Sided p	Mean Difference	Std. Error Difference	Lower	Upper
	Equal variances assumed	3,328	,072	,764	83	,224	,447	,52	,69	-,84	1,88
	Equal variances not assumed			,802	82,975	,212	,425	,52	,65	-,77	1,82
Anterior SAT distance	Equal variances assumed	,410	,524	,481	83	,316	,632	1,27	2,64	-3,98	6,52

	Equal variances not assumed			,492	80,580	,312	,624	1,27	2,59	-3,87	6,42
Posterior SAT distance	Equal variances assumed	,116	,735	,815	83	,209	,417	3,00	3,68	-4,32	10,33
	Equal variances not assumed			,821	77,378	,207	,414	3,00	3,66	-4,28	10,28
Anterior+Posterior SAT distance	Equal variances assumed	,015	,902	,773	83	,221	,442	4,27	5,53	-6,72	15,26
	Equal variances not assumed			,777	76,886	,220	,440	4,27	5,50	-6,68	15,22
VAT distance	Equal variances assumed	1,613	,208	1,442	82	,077	,153	3,48	2,41	-1,32	8,28
	Equal variances not assumed			1,497	81,938	,069	,138	3,48	2,33	-1,15	8,10
Right common femoral artery area (mm2)	Equal variances assumed	,051	,822	-,688	83	,247	,494	-3,23	4, 70	-12,58	6,12
	Equal variances not assumed			-,670	67,513	,253	,505	-3,23	4,83	-12,86	6,40
Left common femoral artery area (mm2)	Equal variances assumed	,206	,651	-,974	83	,166	,333	-4,32	4,43	-13,13	4,50
	Equal variances not assumed			-,933	62,140	,177	,354	-4,32	4,63	-13,57	4,93
FAT area (cm2)	Equal variances assumed	,336	,564	1,130	83	,131	,262	4805,90	4252,33	-3651,81	13263,61

	Equal variances not assumed			1,151	79,995	,127	,253	4805,90	4174,87	-3502,37	13114,17
SAT area (cm2)	Equal variances assumed	,333	,565	1,282	83	,102	,204	3698,81	2885,87	-2041,07	9438,68
	Equal variances not assumed			1,301	79,350	,098	,197	3698,81	2842,18	-1958,01	9355,63
VAT area (cm2)	Equal variances assumed	1,327	,253	,551	82	,292	,583	990,27	1798,72	-2587,96	4568,49
	Equal variances not assumed			,567	81,409	,286	,572	990,27	1746,67	-2484,79	4465,32
Right Psoas muscle area (cm2)	Equal variances assumed	1,838	,179	- 1,212	83	,115	,229	-53,95	44,53	-142,51	34,62
	Equal variances not assumed			- 1,229	79,126	,111	,223	-53,95	43,90	-141,32	33,43
Left Psoas muscle area (cm2)	Equal variances assumed	,000	,992	-,867	83	,194	,388	-41,68	48,08	-137,31	53,95
	Equal variances not assumed			-,876	78,134	,192	,384	-41,68	47,61	-136,45	53,09
FAT mean density (HU)	Equal variances assumed	,094	,760	-,459	83	,324	,647	-,98	2,13	-5,22	3,26
	Equal variances not assumed			-,450	69,694	,327	,654	-,98	2,18	-5,32	3,36
FAT median density (HU)	Equal variances assumed	,136	,714	-,484	83	,315	,630	-1,18	2,45	-6,05	3,68

	Equal variances not assumed			-,473	68,454	,319	,638	-1,18	2,50	-6,18	3,81
FAT density standard deviation	Equal variances assumed	,092	,763	,764	83	,223	,447	,49	,64	-,78	1,76
	Equal variances not assumed			,768	76,851	,222	,445	,49	,64	-,78	1,76
SAT mean density (HU)	Equal variances assumed	,004	,949	-,395	83	,347	,694	-,91	2,30	-5,47	3,66
	Equal variances not assumed			-,389	70,589	,349	,699	-,91	2,34	-5,56	3,75
SAT median density (HU)	Equal variances assumed	2,488	,119	-1,133	81	,130	,261	-5,86	5,17	-16,15	4,43
	Equal variances not assumed			-1,018	41,170	,157	,315	-5,86	5,76	-17,49	5,77
SAT density standard deviation	Equal variances assumed	,362	,549	1,475	82	,072	,144	1,02	,69	-,36	2,40
	Equal variances not assumed			1,499	77,345	,069	,138	1,02	,68	-,34	2,38
VAT mean density (HU)	Equal variances assumed	,251	,618	-,637	83	,263	,526	-1,38	2,17	-5,70	2,94
	Equal variances not assumed			-,631	73,014	,265	,530	-1,38	2,19	-5,75	2,99
VAT median density (HU)	Equal variances assumed	3,013	,086	-1,337	83	,092	,185	-6,51	4,87	-16,19	3,17

	Equal variances not assumed			-1,186	41,706	,121	,242	-6,51	5,49	-17,58	4,57
VAT density standard deviation	Equal variances assumed	,016	,900	1,096	82	,138	,276	,67	,62	-,55	1,90
	Equal variances not assumed			1,089	73,638	,140	,280	,67	,62	-,56	1,91
Right Psoas muscle mean density (HU)	Equal variances assumed	,658	,419	-1,607	83	,056	,112	-2,73	1, 70	-6,10	,65
	Equal variances not assumed			-1,657	82,007	,051	,101	-2,73	1,65	-5,99	,55
Right Psoas muscle median density (HU)	Equal variances assumed	1,801	,183	-1,683	83	,048	,096	-3,34	1,98	-7,28	,61
	Equal variances not assumed			-1,763	82,999	,041	,082	-3,34	1,89	-7,10	,43
Right Psoas muscle density standard deviation	Equal variances assumed	1,222	,272	2,421	83	,009	,018	2,30	,95	,41	4,20
	Equal variances not assumed			2,351	66,680	,011	,022	2,30	,98	,35	4,26
Left Psoas muscle mean density (HU)	Equal variances assumed	4,909	,029	-1,092	83	,139	,278	-1,98	1,81519	-5,59	1,63
	Equal variances not assumed			-1,154	82,715	,126	,252	-1,98	1,72	-5,40	1,44
Left Psoas muscle median density (HU)	Equal variances assumed	6,151	,015	-1,396	83	,083	,166	-2,77	1,98	-6,71	1,18

	Equal variances not assumed			-1,505	79,624	,068	,136	-2,77	1,84	-6,43	,89
Left Psoas muscle density standard deviation	Equal variances assumed	,162	,689	1,043	83	,150	,300	1,16	1,12	-1,06	3,38
	Equal variances not assumed			1,036	73,591	,152	,304	1,16	1,12	-1,07	3,40

The Levene test is accepted ($p > 0.05$) in almost all cases, except ($p < 0.05$) for the Left Psoas muscle mean density (HU) and Left Psoas muscle median density (HU) variables, for which the robust t-test will be performed.

The difference between the means of the two groups is statistically different ($p < 0.05$) only for the Right Psoas muscle density standard deviation variable, in particular the mean is higher in the group No. For the other variables, however, there are no statistically significant differences ($p > 0.05$) between the two groups.

Pearson's correlations of days of intensive care with other variables

Correlations

		Days of intensive care	Psoas/height	Anterior SAT distance	Posterior SAT distance	Anterior+Posterior SAT distance	VAT distance	Right common femoral artery area (mm2)	Left common femoral artery area (mm2)
Days of intensive care	Pearson Correlation	--							
	N	85							
Psoas/height	Pearson Correlation	-,125	--						
	Sig. (2-tailed)	,253							
	N	85	85						
Anterior SAT distance	Pearson Correlation	-,214*	,343**	--					
	Sig. (2-tailed)	,049	,001						
	N	85	85	85					
Posterior SAT distance	Pearson Correlation	-,157	,338**	,515**	--				
	Sig. (2-tailed)	,152	,002	<,001					
	N	85	85	85	85				
Anterior+Posterior SAT distance	Pearson Correlation	-,206	,389**	,821**	,913**	--			
	Sig. (2-tailed)	,058	<,001	<,001	<,001				
	N	85	85	85	85	85			
VAT distance	Pearson Correlation	-,153	,298**	,582**	,288**	,470**	--		
	Sig. (2-tailed)	,164	,006	<,001	,008	<,001			

	N	84	84	84	84	84	84		
Right common femoral artery area (mm2)	Pearson Correlation	-,017	,113	-,146	-,005	-,073	,113	--	
	Sig. (2-tailed)	,874	,303	,182	,962	,506	,306		
	N	85	85	85	85	85	84	85	
Left common femoral artery area (mm2)	Pearson Correlation	,043	,116	-,091	,066	,000	,175	,856**	--
	Sig. (2-tailed)	,695	,289	,408	,551	,998	,111	<,001	
	N	85	85	85	85	85	84	85	85

*. Correlation is significant at the 0.05 level (2-tailed).

**. Correlation is significant at the 0.01 level (2-tailed).

Correlations

		Days of intensive care	FAT area (cm2)	SAT area (cm2)	VAT area (cm2)	Right Psoas muscle area (cm2)	Left Psoas muscle area (cm2)	FAT mean density (HU)	FAT median density (HU)	FAT density standard deviation
Days of intensive care	Pearson Correlation	--								
	N	85								
FAT area (cm2)	Pearson Correlation	-,139	--							
	Sig. (2-tailed)	,205								
	N	85	85							
SAT area (cm2)	Pearson Correlation	-,099	,947**	--						
	Sig. (2-tailed)	,367	<,001							
	N	85	85	85						
VAT area (cm2)	Pearson Correlation	-,171	,850**	,638**	--					
	Sig. (2-tailed)	,120	<,001	<,001						
	N	84	84	84	84					
	Pearson Correlation	-,163	,233*	,128	,365**	--				

Right Psoas muscle area (cm2)	Sig. (2-tailed)	,135	,032	,245	<,001					
	N	85	85	85	84	85				
Left Psoas muscle area (cm2)	Pearson Correlation	-,163	,207	,096	,344**	,880**	--			
	Sig. (2-tailed)	,137	,058	,382	,001	<,001				
	N	85	85	85	84	85	85			
FAT mean density (HU)	Pearson Correlation	,168	-,491**	-,416**	-,508**	-,211	-,205	--		
	Sig. (2-tailed)	,124	<,001	<,001	<,001	,052	,059			
	N	85	85	85	84	85	85	85		
FAT median density (HU)	Pearson Correlation	,169	-,481**	-,414**	-,488**	-,209	-,208	,991**	--	
	Sig. (2-tailed)	,122	<,001	<,001	<,001	,055	,056	<,001		
	N	85	85	85	84	85	85	85	85	
FAT density standard deviation	Pearson Correlation	-,196	,372**	,296**	,416**	,260*	,169	-,563**	-,518**	--
	Sig. (2-tailed)	,073	<,001	,006	<,001	,016	,122	<,001	<,001	
	N	85	85	85	84	85	85	85	85	85

**. Correlation is significant at the 0.01 level (2-tailed).

*. Correlation is significant at the 0.05 level (2-tailed).

Correlations

		Days of intensive care	SAT mean density (HU)	SAT median density (HU)	SAT density standard deviation	VAT mean density (HU)	VAT median density (HU)	VAT density standard deviation
Days of intensive care	Pearson Correlation	--						
	N	85						
SAT mean density (HU)	Pearson Correlation	,172	--					
	Sig. (2-tailed)	,115						
	N	85	85					
SAT median density (HU)	Pearson Correlation	,049	,591**	--				
	Sig. (2-tailed)	,659	<,001					
	N	83	83	83				

SAT density standard deviation	Pearson Correlation	-,262*	-,454**	-,160	--			
	Sig. (2-tailed)	,016	<,001	,150				
	N	84	84	82	84			
VAT mean density (HU)	Pearson Correlation	,116	,822**	,399**	-,553**	--		
	Sig. (2-tailed)	,292	<,001	<,001	<,001			
	N	85	85	83	84	85		
VAT median density (HU)	Pearson Correlation	,067	,321**	,175	-,269*	,493**	--	
	Sig. (2-tailed)	,542	,003	,113	,013	<,001		
	N	85	85	83	84	85	85	
VAT density standard deviation	Pearson Correlation	,106	-,313**	-,223*	,609**	-,484**	-,263*	--
	Sig. (2-tailed)	,337	,004	,043	<,001	<,001	,016	
	N	84	84	83	83	84	84	84

*. Correlation is significant at the 0.05 level (2-tailed).

**. Correlation is significant at the 0.01 level (2-tailed).

Correlations

		Days of intensive care	Right Psoas muscle mean density (HU)	Right Psoas muscle median density (HU)	Right Psoas muscle density standard deviation	Left Psoas muscle mean density (HU)	Left Psoas muscle median density (HU)	Left Psoas muscle density standard deviation
Days of intensive care	Pearson Correlation	--						
	N	85						
Right Psoas muscle mean density (HU)	Pearson Correlation	,096	--					
	Sig. (2-tailed)	,385						
	N	85	85					
Right Psoas muscle median density (HU)	Pearson Correlation	,149	,973**	--				
	Sig. (2-tailed)	,173	<,001					
	N	85	85	85				
Right Psoas muscle density standard deviation	Pearson Correlation	-,095	-,123	-,227*	--			
	Sig. (2-tailed)	,385	,262	,037				
	N	85	85	85	85			

Left Psoas muscle mean density (HU)	Pearson Correlation	,264*	,763**	,766**	-,039	--		
	Sig. (2-tailed)	,014	<,001	<,001	,722			
	N	85	85	85	85	85		
Left Psoas muscle median density (HU)	Pearson Correlation	,284**	,765**	,800**	-,167	,969**	--	
	Sig. (2-tailed)	,008	<,001	<,001	,127	<,001		
	N	85	85	85	85	85	85	
Left Psoas muscle density standard deviation	Pearson Correlation	-,125	-,155	-,274*	,855**	-,099	-,212	--
	Sig. (2-tailed)	,252	,156	,011	<,001	,366	,052	
	N	85	85	85	85	85	85	85

*. Correlation is significant at the 0.05 level (2-tailed).

**. Correlation is significant at the 0.01 level (2-tailed).

From the correlation matrix it can be observed that there is a negative, statistically significant and low relationship ($p < 0.05$) between the variable Days of intensive care and the variables Anterior SAT distance and SAT density standard deviation: as one decreases, the other increases and vice versa.

There is a positive, statistically significant and low relationship ($p < 0.05$) between the variable Days of intensive care and the variables Left Psoas muscle mean density (HU) and Left Psoas muscle median density (HU): as one increases, the other increases and vice versa.

There are no statistically significant ($p > 0.05$) relationships with Days of intensive care and the other variables, so they are linearly independent, i.e. it is as if the correlation were 0.
